# Supplementary material for: Enantiotopic-group-selective coupling for unified access to carbazole atropisomers as versatile chiral chromophores
Source: Nat Commun. 2025 Nov 21;16:10313. doi: 10.1038/s41467-025-65243-8 (PMC12638793; doi:10.1038/s41467-025-65243-8)
Supplement: Supplementary file 1 — Supplementary Information [file 41467_2025_65243_MOESM1_ESM.pdf]

Supplementary Information

Enantiotopic-Group-Selective Coupling for Unified Access to Carbazole Atropisomers as Versatile Chiral Chromophores

Junqiang Wei[1], Zhuoer Wang[2], Pengyao Xing*[,2] and Ye Zhu*[,1]

[1]Department of Chemistry, Faculty of Science, National University of Singapore, 3 Science Drive 3, Singapore 117543.

*Correspondence: chmzhu@nus.edu.sg

[2]School of Chemistry and Chemical Engineering, Shandong University, Jinan 250100, People's Republic of China.

*Correspondence: xingpengyao@sdu.edu.cn.

Table of Contents

List of acronyms and abbreviations 3
General information 5
Preparation of ligands 7
Preparation of substrates 13
Side chain parameters for amino acids 33
General procedure for Suzuki cross-coupling desymmetrization reactions of N–C and N–N carbazole acid substrates. 34
Characterization data of desymmetrizating products from N–C and N–N carbazole acid substrates 35
General procedure for Suzuki cross-coupling desymmetrization reactions of N–N carbazole ester substrates. 66
Characterization data of desymmetrizating products from N–N carbazole ester substrates 67
Buchwald-hartwig amination and Sonogashira cross-coupling desymmetrization reaction 72
Suzuki cross-coupling desymmetrization reactions of ethyl ester substrates. 77
Racemization of N–C and N–N carbazole products. 77
Suzuki cross-coupling desymmetrization reactions of 2-(3,6-dichloro-9*H*-carbazol-9-yl)-3-methylbenzoic acid 78
Synthetic utilities 79
Chiroptical properties 102
NMR Spectra 112
HPLC Traces 229
X-Ray Crystallography Data 307
References 310

List of acronyms and abbreviations

**Ac**	Acetyl
**Bu**	Butyl
**BINAP**	2,2′-Bis(diphenylphosphino)-1,1′-binaphthyl
**Bn**	Benzyl
**Cy**	Cyclohexyl
**Bpin**	Pinacol boronic ester
**dan**	Naphthalene-1,8-diaminato
**dba**	Dibenzylideneacetone
**DCE**	1,2-Dichloroethane
**DCM**	Dichloromethane
**DIBAL-H**	Diisobutylaluminum hydride
**DIPEA**	*N,N*-Diisopropylethylamine
**DMEDA**	*N,N′*-Dimethylethylenediamine
**DMF**	*N,N*-Dimethylformamide
**DMSO**	Dimethyl sulfoxide
**DPPA**	Diphenylphosphoryl azide
**dr**	Diastereomeric ratio
**EA**	Ethyl acetate
**EDC**	*N*-(3-dimethylaminopropyl)-*N*’-ethylcarbodiimide
**er**	Enantiomeric ratio
**Et**	Ethyl

**HOBt**	1-Hydroxybenzotriazole
**HOMO**	Highest occupied molecular orbital
**LUMO**	Lowest unoccupied molecular orbital
**Me**	Methyl
**NCS**	*N*-Chlorosuccinimide
**Ph**	Phenyl
**pin**	Pinacolato
**PMP**	*p*-Methoxyphenyl
**Pr**	Propyl
**RuPhos**	2-Dicyclohexylphosphino-2′,6′-diisopropoxybiphenyl
**SPhos**	2-Dicyclohexylphosphino-2′,6′-dimethoxybiphenyl
**TBS**	*tert*-Butyldimethylsilyl
**TCNB**	1,2,4,5-Tetrachloro-3-nitrobenzene
**TFA**	Trifluoroacetic acid
**THF**	Tetrahydrofuran
**TMS**	Trimethylsilyl
**Ts**	Tosyl
**XPhos**	2-Dicyclohexylphosphino-2′,4′,6′-triisopropylbiphenyl

General information

Materials: Commercially available reagents and solvents were used as received. Bis[2-(diphenylphosphino)phenyl]ether oxide (DPEPO) was purchased from Bide pharm. Poly(methyl methacrylate (PMMA, m.w. =350,000 Da) were purchased from Shanghai Xiensi Biotechnology Co., Ltd. Commercial dry solvents (Aldrich Sure/Seal^{TM}) were sparged with nitrogen before being used in catalytic reactions. Solvents used for column chromatography were analytical grade.

Methods: Unless otherwise noted, all experiments were set up under an atmosphere of nitrogen in a glovebox or using standard Schlenk techniques. Reactions were monitored by thin-layer chromatography (TLC), gas chromatography (GC), or nuclear magnetic resonance (NMR) analysis. Flash column chromatography was performed using Tsingdao silica gel (60, particle size 300-400 mesh). Yields refer to isolated yields after flash column chromatography purification.

Instrumentation: Circularly polarized luminescence (CPL), Circular dichroism (CD) were measured with an Applied Photophysics ChirascanV100 model. Fluorescence lifetime and quantum yield (QY) were measured by Steady State-Transient-Fluorescence Spectrometer (Edinburgh FLS920). UV-Visible absorption spectra at room temperature was recorded on a UV-1900 Shimadzu spectrophotometer. Fluorescence spectra was made on a RF-6000 Shimadzu fluorophotometer.

Characterization: Products were characterized utilizing nuclear magnetic resonance (NMR), mass spectrometry (MS), high-performance liquid chromatography (HPLC), and optical rotation. NMR spectra were recorded on a Bruker DPX 400 spectrometer at 300 MHz for ^{1}H NMR, 101 MHz for ^{13}C NMR, and 162 MHz for ^{31}P NMR or on a Bruker DPX 500 spectrometer at 500 MHz for ^{1}H

NMR, 126 MHz for ^{13}C NMR, 202 MHz for ^{31}P NMR and 471 MHz for ^{19}F NMR in $CDCl_3$ with tetramethylsilane as internal standard. Chemical shifts were reported relative to Chloroform-*d* (7.26 ppm) for ^{1}H NMR and relative to Chloroform-*d* (77.0 ppm) for ^{13}C NMR. ^{19}F spectra were calibrated from the external standard ($CFCl_3$: 0 ppm). ^{31}P spectra were calibrated from the external standard (85 wt% phosphoric acid: 0 ppm). NMR data are reported as chemical shift (parts per million, ppm), multiplicity (s = singlet, d = doublet, t = triplet, q = quartet, m = multiplet), coupling constant (Hz), and integration. HPLC analysis was performed on a Shimadzu i-series HPLC system equipped with a photodiode array (PDA) detector and Chiralcel and Chiralpak columns (0.46 cm∅× 25 cm). The wavelength that is an apex in the spectrum was selected for analysis of the enantiomeric ratio (er) of each compound. Optical rotation ($[\alpha]_D^T$, deg•cm^3•g^{-1}•dm^{-1}) was measured on a Jasco DIP-1000 Digital Polarimeter at λ=589 nm in the given solvent at the indicated concentration (c, g/100 mL) and temperature (T, °C). X-ray diffraction was performed on Bruker D8 Venture single crystal X-ray diffractometer.

Preparation of ligands

1. General Remarks

L1 to L4 was synthesized from RuPhos by the previously reported kinds of literature.[1-4] All amino acid residues and coupling reagents were purchased from commercial suppliers. Yields are not optimized.

2. The general procedure of representative amino acid-drived ligands[2,5]

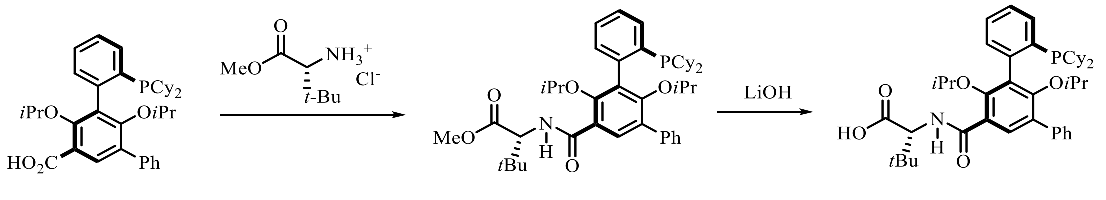

L9-((*S*)-2-(dicyclohexylphosphaneyl)-2', 6'-diisopropoxy-[1, 1':3', 1''-terphenyl]-5'-carbonyl)-*D*-tert-leucine

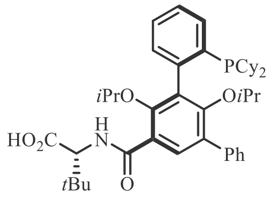

Under N_2 atmosphere, **L4**-(*S*)-2-(dicyclohexylphosphaneyl)-2',6'-diisopropoxy-[1,1':3',1''-terphenyl]-5'-carboxylic acid (58.6 mg, 0.1 mmol), DIPEA (51.6 mg, 0.4 mmol), HOBt·H_2O (27 mg, 0.2 mmol), and *D*-Tert-Leucine Methyl ester hydrochloride (18 mg, 0.1 mmol) were added DCM (1 mL). The mixture was cooled to 0 °C and EDC·HCl (38.4 mg, 0.2 mmol) was then added to the solution. After that, the resulting reaction mixture was stirred at room temperature for 24 h. The reaction was quenched with H_2O, then the mixture was extracted with EA (5 mL X 3) three times. The combined organic phases were washed with brine, dried over Na_2SO_4, and concentrated in vacuo. The crude product **SI-L9** was used directly in the next step without further purification.

Under the N_2 atmosphere, **SI-L9** (crude material) was dissolved in 0.5 mL THF and 0.5 mL MeOH, followed by the addition of 2 M LiOH $_{(aq.)}$ (0.5 mL, 1 mmol). The resulting reaction mixture was stirred at 45 °C for 24 h. After the reaction was completed, removal of the organic phase was performed under reduced pressure, and water was added and extracted with DCM (5 mL X 3) three times. The combined organic phases were washed with brine, dried over Na_2SO_4, and concentrated in vacuo. The crude materials were purified by flash column chromatography with hexane and acetone (5:1 – 2:1) to give the desired product **L9**, 40 mg in 57% yield over two steps as a white solid. ^{1}H NMR (500 MHz, Acetone-d_6) δ 8.48 (d, J = 8.8 Hz, 1H), 8.09 (s, 1H), 7.81 – 7.78 (m, 1H), 7.67 – 7.63 (m, 2H), 7.49 – 7.44 (m, 5H), 7.40 – 7.35 (m, 1H), 4.69 – 4.61 (m, 1H), 4.00 – 3.92 (m, 1H), 3.67 – 3.62 (m, 1H), 2.34 – 2.24 (m, 2H), 2.21 – 2.15 (m, 1H), 2.05 – 1.96 (m, 2H), 1.87 (d, J = 10.3 Hz, 2H), 1.75 (dd, J = 28.4, 11.6 Hz, 2H), 1.66 (t, J = 16.0 Hz, 2H), 1.51 – 1.37 (m, 5H), 1.32 – 1.20 (m, 6H), 1.11 (s, 9H), 1.04 (d, J = 6.2 Hz, 3H), 0.90 (d, J = 6.2 Hz, 3H), 0.67 (d, J = 6.1 Hz, 3H), 0.56 (d, J = 6.1 Hz, 3H). ^{13}C NMR (101 MHz, Acetone-d_6) δ 165.0, 153.2, 142.3 (d, J = 32.5 Hz), 139.0, 138.3 (d, J = 18.8 Hz), 132.4 (d, J = 6.0 Hz), 132.3 (d, J = 3.2 Hz), 131.9, 130.9, 129.1, 128.2, 127.7, 127.1 (d, J = 2.8 Hz), 123.8, 75.8, 75.7, 60.3, 34.7 (d, J = 16.5 Hz), 33.6 (d, J = 23.9 Hz), 32.7 (d, J = 14.6 Hz), 30.6 (d, J = 18.8 Hz), 30.3 (d, J = 13.9 Hz), 27.9 (d, J = 15.6 Hz), 27.6 (d, J = 4.5 Hz), 27.2, 26.9 (d, J = 9.7 Hz), 26.6 (d, J = 10.9 Hz), 26.4, 26.3, 26.2, 21.8, 21.7, 21.0, 19.8. ^{31}P NMR (202 MHz, Acetone-d_6) δ -8.02. HRMS (m/z, ESI): Calcd. for Chemical Formula: $C_{43}H_{59}NO_5P^+$ $[M+H]^+$: 700.4125, Found: 700.4120.

3. Characterization and Spectra of amino acid-drived ligands L5 to *ent*-L9

L5-((*S*)-2-(dicyclohexylphosphaneyl)-2', 6'-diisopropoxy-[1, 1':3', 1''-terphenyl]-5'-carbonyl)-*D*-alanine

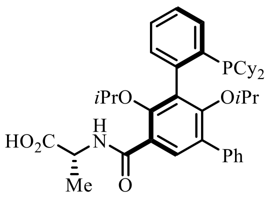

Prepared from *D*-alanine Methyl Ester Hydrochloride and (*S*)-**L4**, two steps, 43 mg in 66% yield as a white solid. ^{1}H NMR (500 MHz, Acetone-d_6) δ 8.57 (t, *J* = 7.1 Hz, 1H), 8.09 (d, *J* = 7.3 Hz, 1H), 7.78 – 7.74 (m, 1H), 7.65 – 7.60 (m, 2H), 7.49 – 7.41 (m, 5H), 7.34 (td, *J* = 7.2, 1.4 Hz, 1H), 4.75 – 4.69 (m, 1H), 3.99 – 3.93 (m, 1H), 3.69 – 3.62 (m, 1H), 2.27 – 2.17 (m, 2H), 2.12 – 1.98 (m, 2H), 1.94 – 1.86 (m, 1H), 1.83 – 1.74 (m, 3H), 1.71 – 1.61 (m, 3H), 1.52 (t, *J* = 7.4 Hz, 3H), 1.42 – 1.17 (m, 11H), 1.01 (dd, *J* = 6.1, 1.3 Hz, 3H), 0.94 (dd, *J* = 16.8, 6.2 Hz, 3H), 0.63 (dd, *J* = 10.5, 6.1 Hz, 3H), 0.56 (d, *J* = 6.1 Hz, 3H). ^{13}C NMR (126 MHz, Acetone-d_6) δ 175.7, 162.1, 154.1, 151.4, 140.3 (d, *J* = 33.0 Hz), 137.2, 136.2 (d, *J* = 19.0 Hz), 130.5, 129.8 (d, *J* = 25.4 Hz), 128.5, 127.1, 126.1, 125.5, 124.9, 122.5, 73.7 (d, *J* = 63.4 Hz), 66.3, 49.0, 32.5 (d, *J* = 16.5 Hz), 31.5 (d, *J* = 24.1 Hz), 30.7 (d, *J* = 15.2 Hz), 28.3 (d, *J* = 16.7 Hz), 26.3 (d, *J* = 19.3 Hz), 26.0 (d, *J* = 19.0 Hz), 25.7, 25.6, 25.4, 25.1, 24.8, 24.6 (d, *J* = 10.6 Hz), 24.3 (d, *J* = 17.8 Hz), 19.7, 19.6, 19.2, 18.1, 17.0. ^{31}P NMR (202 MHz, Acetone-d_6) δ -8.40. HRMS (m/z, ESI): Calcd. for Chemical Formula: $C_{40}H_{53}NO_5P^+$ $[M+H]^+$: 658.3656, Found: 658.3659.

L6-((*S*)-2-(dicyclohexylphosphaneyl)-2', 6'-diisopropoxy-[1, 1':3', 1''-terphenyl]-5'-carbonyl)-*D*-leucine

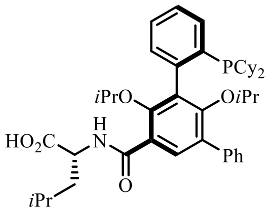

Prepared from *D*-Leucine Methyl Ester Hydrochloride and (*S*)-**L4**, two steps, 40 mg in 56% yield as a white solid. ^{1}H NMR (500 MHz, Acetone-d_6) δ 8.39 (d, *J* = 7.7 Hz, 1H), 8.06 (s, 1H), 7.79 – 7.74 (m, 1H), 7.65 – 7.61 (m, 2H), 7.48 – 7.42 (m, 5H), 7.37 – 7.33 (m, 1H), 4.83 – 4.76 (m, 1H), 3.98 – 3.91 (m, 1H), 3.65 (p, *J* = 6.1 Hz, 1H), 2.27 – 2.20 (m, 2H), 2.12 – 2.07 (m, 1H), 1.99 – 1.96 (m, 1H), 1.93 – 1.90

(m, 1H), 1.86 – 1.74 (m, 5H), 1.74 – 1.69 (m, 1H), 1.68 – 1.62 (m, 2H), 1.49 (d, *J* = 13.0 Hz, 1H), 1.43 – 1.17 (m, 11H), 1.05 – 0.99 (m, 9H), 0.92 (d, *J* = 6.2 Hz, 3H), 0.64 (d, *J* = 6.2 Hz, 3H), 0.56 (d, *J* = 6.2 Hz, 3H). ^{13}C NMR (126 MHz, Acetone-*d*$_6$) δ 174.1, 165.6, 157.4, 154.2, 143.0 (d, *J* = 32.6 Hz), 139.9, 139.0 (d, *J* = 19.2 Hz), 133.0 (d, *J* = 5.9 Hz), 132.6, 131.8, 129.9, 128.9, 128.4, 127.8, 124.4, 76.6, 76.2, 51.6, 42.9, 35.3 (d, *J* = 16.2 Hz), 33.8 (d, *J* = 10.7 Hz), 33.7 (d, *J* = 17.7 Hz), 31.4 (d, *J* = 15.1 Hz), 31.0, 28.6 (d, *J* = 14.1 Hz), 28.5, 28.2 (d, *J* = 5.7 Hz), 27.8 (d, *J* = 10.4 Hz), 27.5, 27.1 (d, *J* = 17.5 Hz), 25.6, 23.0, 22.5, 22.4 (d, *J* = 7.0 Hz), 21.9, 21.3. ^{31}P NMR (202 MHz, Acetone-*d*$_6$) δ -8.37. HRMS (m/z, ESI): Calcd. for Chemical Formula: $C_{43}H_{59}NO_5P^+$ $[M+H]^+$: 700.4125, Found: 700.4129.

L7-((*S*)-2-(dicyclohexylphosphaneyl)-2', 6'-diisopropoxy-[1, 1':3', 1''-terphenyl]-5'-carbonyl)-*D*-valine

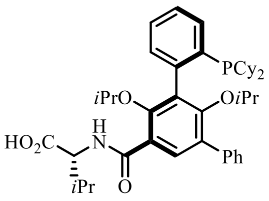

Prepared from *D*-valine Methyl Ester Hydrochloride and (*S*)-**L4**, two steps, 41 mg in 60% yield as a white solid. ^{1}H NMR (500 MHz, Acetone-*d*$_6$) δ 8.45 (d, *J* = 8.0 Hz, 1H), 8.07 (s, 1H), 7.79 – 7.76 (m, 1H), 7.65 – 7.61 (m, 2H), 7.48 – 7.42 (m, 5H), 7.37 – 7.33 (m, 1H), 4.70 (dd, *J* = 8.0, 4.8 Hz, 1H), 3.98 – 3.92 (m, 1H), 3.68 – 3.60 (m, 1H), 2.32 – 2.21 (m, 3H), 2.16 – 2.11 (m, 1H), 1.98 – 1.96 (m, 2H), 1.85 – 1.75 (m, 3H), 1.72 – 1.60 (m, 3H), 1.47 – 1.34 (m, 5H), 1.32 – 1.18 (m, 6H), 1.05 (t, *J* = 6.6 Hz, 9H), 0.90 (d, *J* = 6.3 Hz, 3H), 0.65 (d, *J* = 6.2 Hz, 3H), 0.55 (d, *J* = 6.1 Hz, 3H). ^{13}C NMR (126 MHz, Acetone-*d*$_6$) δ 173.1, 165.8, 157.5, 154.1, 143.1 (d, *J* = 32.6 Hz), 139.8, 139.1 (d, *J* = 19.2 Hz), 133.1 (d, *J* = 6.1 Hz), 133.0 (d, *J* = 3.1 Hz), 132.6, 131.7, 129.9, 129.0, 128.5, 127.9, 124.5, 76.5, 76.4, 58.2, 35.4 (d, *J* = 16.3 Hz), 34.1 (d, *J* = 23.0 Hz), 33.6 (d, *J* = 14.6 Hz), 32.1, 31.2 (d, *J*

= 3.5 Hz), 31.1, 30.0, 29.8, 28.6 (d, J = 15.1 Hz), 28.3 (d, J = 5.0 Hz), 28.2, 27.7 (d, J = 10.1 Hz), 27.4 (d, J = 10.8 Hz), 27.1 (d, J = 21.6 Hz), 22.5, 22.4, 21.8, 20.9, 19.3, 18.6. ^{31}P NMR (202 MHz, Acetone-d_6) δ -8.17. HRMS (m/z, ESI): Calcd. for Chemical Formula: $C_{42}H_{57}NO_5P^+$ $[M+H]^+$: 686.3969, Found: 686.3979.

L8-(*R*)-2'-(dicyclohexylphosphaneyl)-2,6-diisopropoxy-5-methyl-[1,1'-biphenyl]-3-carbonyl-*L*-*iso*-leucine

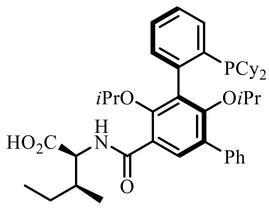

Prepared from Methyl *L*-isoleucine hydrochloride and (*R*)-**L4**, two steps, 11 mg in 17% yield as a white solid. ^{1}H NMR (500 MHz, Acetone-d_6) δ 8.09 (s, 1H), 7.73 (d, J = 5.4 Hz, 1H), 7.60 (d, J = 7.7 Hz, 2H), 7.46 – 7.24 (m, 6H), 4.38 (s, 1H), 3.83 (s, 1H), 3.63 – 3.57 (m, 1H), 2.30 (d, J = 7.9 Hz, 1H), 2.23 – 2.13 (m, 2H), 1.98 – 1.87 (m, 2H), 1.83 (s, 2H), 1.71 (d, J = 15.8 Hz, 2H), 1.64 – 1.52 (m, 3H), 1.48 – 1.06 (m, 13H), 0.97 – 0.70 (m, 13H), 0.63 (d, J = 6.2 Hz, 3H), 0.52 (d, J = 6.2 Hz, 3H). ^{13}C NMR (126 MHz, Acetone-d_6) δ 177.0, 165.2, 156.5, 153.4, 144.8, 142.4 (d, J = 33.1 Hz), 139.2, 138.3 (d, J = 19.5 Hz), 132.5, 132.0 (d, J = 24.0 Hz), 130.6, 129.2, 128.1, 127.6, 127.0, 124.2, 116.2, 76.1, 75.7, 68.4, 53.8 (dt, J = 38.8, 19.4 Hz), 37.7, 34.6 (d, J = 16.8 Hz), 33.7 (d, J = 24.5 Hz), 32.7 (d, J = 14.8 Hz), 30.5 (d, J = 18.7 Hz), 30.3, 28.2 (d, J = 19.0 Hz), 27.9 (d, J = 19.4 Hz), 27.6 (d, J = 12.2 Hz), 27.1, 26.9 (d, J = 9.7 Hz), 26.6 (d, J = 10.8 Hz), 26.4 (d, J = 19.4 Hz), 25.3 (d, J = 12.2 Hz), 21.8 (d, J = 15.3 Hz), 21.4, 20.2, 15.6, 11.5. ^{31}P NMR (202 MHz, Acetone-d_6) δ -8.49. HRMS (m/z, ESI): Calcd. for Chemical Formula: $C_{43}H_{59}NO_5P^+$ $[M+H]^+$: 700.4125, Found: 700.4125.

***ent*-L9-((*R*)-2-(dicyclohexylphosphaneyl)-2',6'-diisopropoxy-[1,1':3',1''-terphenyl]-5'-carbon**

-yl)-*L*-*tert*-leucine

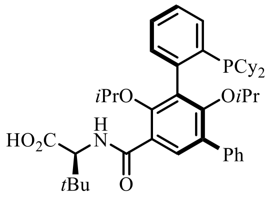

Prepared from *L*-Tert-Leucine Methyl ester hydrochloride and(*R*)-**L4**, two steps, 35 mg in 50% yield as a white solid. NMR was same with **L9**. HRMS (m/z, ESI): Calcd. for Chemical Formula: $C_{43}H_{59}NO_5P^+$ $[M+H]^+$: 700.4125, Found:700.4123.

Preparation of substrates

1. The general procedure A for the synthesis of substrates.[6, 7]

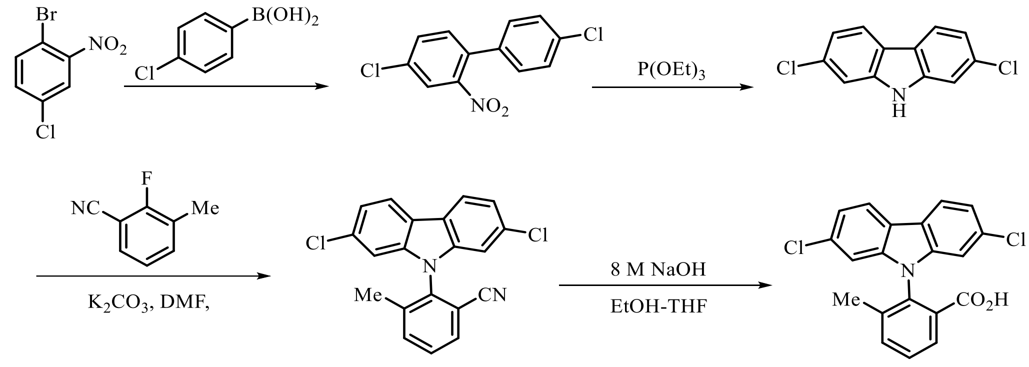

2-(2,7-dichloro-9*H*-carbazol-9-yl)-3-methylbenzoic acid (1)

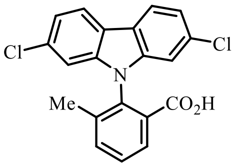

Under N_2 atmosphere, 1-Bromo-4-chloro-2-nitrobenzene (2.36 g, 10 mmol), 4-chlorophenylboronic acid (1.56 g, 10 mmol), K_2CO_3 (4.14 g, 30 mmol), and $Pd(PPh_3)_4$ (231 mg, 0.2 mmol) were dissolved in 20 mL 1,4-dioxane and 5 mL H_2O, then the reaction mixture was stirred for 18 h at 100 °C. The reaction was quenched with H_2O and extracted with EA (40 mL X 3) three times. The combined organic phases were washed with brine, dried over Na_2SO_4, and concentrated in vacuo. The crude product was rapidly filtered through column chromatography and directly uesd for next step.

Under the N_2 atmosphere, the above mixture was dissolved in 15 mL $P(OEt)_3$, then the reaction mixture was heated to 145 °C and stirred at this temperature for 15 h. Then, the majority of $P(OEt)_3$ was removed by vacuum distillation. After that, the crude materials were purified by flash column chromatography with hexane and acetone (150:1 – 20:1) to give the desired product, 2,7-dichloro-9H-carbazole, 1.43 g in 61% yield as a white solid over two steps. ^{1}H NMR (500 MHz, Chloroform-*d*) δ 8.01 (s, 1H), 7.90 (d, J = 8.3 Hz, 2H), 7.37 (d, J = 1.9 Hz, 2H), 7.21 (dt, J = 8.3, 1.4 Hz, 2H). MS (m/z, ESI): Calcd. for Chemical Formula: $C_{12}H_6Cl_2N^-$ $[M-H]^-$: 233.99, Found: 233.94.

Under the N_2 atmosphere, 2,7-dichloro-9*H*-carbazole (1.0 g, 4.3 mmol), 2-Fluoro-3-methyl-benzonitrile (574 mg, 4.3 mmol), and K_2CO_3 (1.78 g, 12.9 mmol) were added to 15 mL DMF, then the mixture was heated to 120 °C, and stirred overnight at this temperature. The reaction was quenched with H_2O and extracted with EA (40 mL X 3) three times. The combined organic phases were washed with brine, dried over Na_2SO_4, and concentrated in vacuo. The crude product was used directly in the next step without further purification.

Under the N_2 atmosphere, the above crude product was dissolved in 10 mL THF, 10 mL MeOH, and 8 M NaOH $_{(aq.)}$ (5 mL, 40 mmol). The resulting reaction mixture was heated to reflux and stirred at this temperature for 4 d. Then, the reaction was cooled to room temperature, the mixture was neutralized with 1 M HCl $_{(aq.)}$ to pH 2 – 3 and extracted with EA (20 mL X 3) three times. The combined organic phases were washed with brine, dried over Na_2SO_4, and concentrated in vacuo. The crude materials were purified by flash column chromatography with hexane and acetone (15:1 – 2:1) to give the desired product, 2.25 g in 92% yield as a white solid. ^{1}H NMR (500 MHz, Chloroform-*d*) δ 8.01 (d, *J* = 8.3 Hz, 2H), 7.90 (t, *J* = 4.7 Hz, 1H), 7.61 – 7.58 (m, 2H), 7.28 (dd, *J* = 8.3, 1.8 Hz, 2H), 6.97 (d, *J* = 1.8 Hz, 2H), 1.86 (s, 3H). ^{13}C NMR (126 MHz, Chloroform-*d*) δ 168.9, 142.0, 139.8, 136.4, 134.6, 132.0, 130.7, 129.5, 121.3, 121.2, 120.6, 109.5, 17.4. HRMS (m/z, ESI): Calcd. for Chemical Formula: $C_{20}H_{12}Cl_2NO_2^-$ [M-H]$^-$: 368.0251, Found: 368.0252.

Ethyl 2-(2,7-dichloro-9*H*-carbazol-9-yl)-3-methylbenzoate (SI-1)

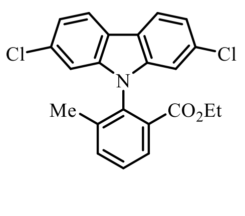

2-(2,7-dichloro-9*H*-carbazol-9-yl)-3-methylbenzoic acid **1** (0.25 mmol) was dissolved in DMF (1.0 mL), and treated with EtI (0.5 mmol) and K_2CO_3 (1.0 mmol). The reaction mixture was stirred overnight at room temperature.

Then the mixture was diluted with ethyl acetate (20.0 mL) and washed with water, brine, and dried over Na_2SO_4. The organic layer was concentrated in vacuo and purified by flash column chromatography with hexane and acetone as the eluents to give the desired product. 99 mg in 99% yield as a white solid. ^{1}H NMR (500 MHz, Chloroform-*d*) δ 7.99 (d, *J* = 8.3 Hz, 3H), 7.68 – 7.62 (m, 1H), 7.58 (t, *J* = 7.7 Hz, 1H), 7.23 (dd, *J* = 8.3, 1.8 Hz, 2H), 6.89 (d, *J* = 1.8 Hz, 2H), 3.73 (q, *J* = 7.1 Hz, 2H), 1.98 (s, 3H), 0.51 (t, *J* = 7.1 Hz, 3H). ^{13}C NMR (101 MHz, Chloroform-*d*) δ 165.6, 142.2, 139.3, 135.2, 133.6, 132.1, 131.6, 129.9, 129.5, 121.1, 120.6, 109.7, 61.1, 17.5, 13.0. HRMS (m/z, ESI): Calcd. for Chemical Formula: $C_{22}H_{18}Cl_2NO_2^+$ $[M+H]^+$: 398.0709, Found: 398.0701.

2-(2,7-dichloro-3,6-dimethoxy-9*H*-carbazol-9-yl)-3-methylbenzoic acid

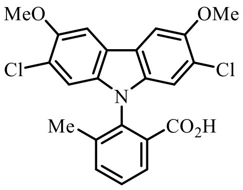

170 mg in 35% yield as a yellow solid. ^{1}H NMR (500 MHz, Chloroform-*d*) δ 7.99 (dd, *J* = 7.8, 1.6 Hz, 1H), 7.68 – 7.62 (m, 1H), 7.55 (d, *J* = 8.5 Hz, 3H), 6.87 (s, 2H), 4.03 (s, 6H), 1.87 (s, 3H). ^{13}C NMR (101 MHz, Chloroform-*d*) δ 168.3, 149.5, 139.9, 136.3, 136.1, 134.8, 130.4, 129.9, 129.3, 122.2, 121.5, 111.1, 103.3, 57.0, 17.3. HRMS (m/z, ESI): Calcd. for Chemical Formula: $C_{22}H_{16}Cl_2NO_4^-$ $[M-H]^-$: 428.0462, Found: 428.0464.

2-(2,7-dichloro-9*H*-carbazol-9-yl)-3'-methoxy-[1,1'-biphenyl]-3-carboxylic acid

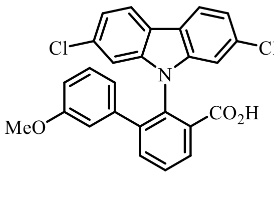

210 mg in 44% yield as a white solid. ^{1}H NMR (500 MHz, Chloroform-*d*) δ 8.16 (dd, *J* = 7.8, 1.7 Hz, 1H), 7.85 – 7.78 (m, 3H), 7.72 (t, *J* = 7.8 Hz, 1H), 7.11 (dd, *J* = 8.3, 1.8 Hz, 2H), 6.95 – 6.90 (m, 1H), 6.87 (d, *J* = 1.8 Hz, 2H), 6.61 – 6.59 (m, 1H), 6.56 – 6.53 (m, 1H), 6.37 (dd, *J* = 2.6, 1.6 Hz, 1H), 3.22 (s, 3H).

^{13}C NMR (126 MHz, Chloroform-*d*) δ 167.9, 158.9, 143.9, 142.6, 138.3, 136.1, 133.3, 131.9, 131.8, 129.9, 129.6, 129.2, 121.1, 120.9, 120.5, 120.3, 115.0, 111.9, 109.8, 54.7. HRMS (m/z, ESI): Calcd. for Chemical Formula: $C_{26}H_{16}Cl_2NO_3^-$ [M-H]$^-$: 460.0513, Found: 460.0514.

2-(2,7-dichloro-9*H*-carbazol-9-yl)-3'-nitro-[1,1'-biphenyl]-3-carboxylic acid

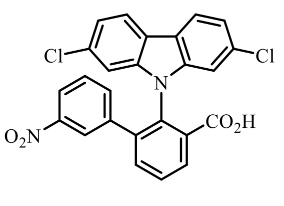

210 mg in 52% yield as a white solid. ^1H NMR (500 MHz, Chloroform-*d*) δ 8.22 (dd, *J* = 7.8, 1.7 Hz, 1H), 7.90 – 7.82 (m, 3H), 7.78 (dd, *J* = 8.1, 1.6 Hz, 3H), 7.14 – 7.09 (m, 1H), 7.14 – 7.09 (m, 3H), 6.83 (d, *J* = 1.8 Hz, 2H). ^{13}C NMR (126 MHz, Chloroform-*d*) δ 168.5, 147.7, 142.3, 141.3, 138.6, 135.9, 133.7, 133.6, 133.0, 132.1, 130.3, 130.1, 129.1, 123.0, 122.9, 121.2, 121.0, 109.5. HRMS (m/z, ESI): Calcd. for Chemical Formula: $C_{25}H_{13}Cl_2N_2O_4^-$ [M-H]$^-$: 475.0258, Found: 475.0259.

2-(2,7-dichloro-9*H*-carbazol-9-yl)-2',3',4',5'-tetrahydro-[1,1'-biphenyl]-3-carboxylic acid

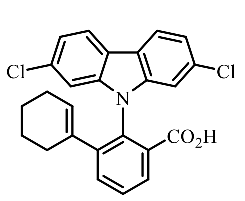

210 mg in 33% yield as a white solid. ^1H NMR (500 MHz, Chloroform-*d*) δ 8.00 (dd, *J* = 7.5, 2.0 Hz, 1H), 7.91 (d, *J* = 8.3 Hz, 2H), 7.63 – 7.55 (m, 2H), 7.18 (dd, *J* = 8.3, 1.8 Hz, 2H), 6.88 (d, *J* = 1.8 Hz, 2H), 5.41 – 5.38 (m, 1H), 1.69 – 1.65 (m, 2H), 1.56 – 1.52 (m, 2H), 1.15 – 1.07 (m, 4H). ^{13}C NMR (126 MHz, Chloroform-*d*) δ 168.5, 146.3, 142.5, 135.0, 133.0, 131.7, 130.8, 129.6, 129.3, 128.7, 121.2, 120.9, 120.5, 110.3, 28.6, 25.3, 22.4, 21.3. HRMS (m/z, ESI): Calcd. for Chemical Formula: $C_{25}H_{18}Cl_2NO_2^-$ [M-H]$^-$: 434.0720, Found: 434.0721.

2-(2,7-dichloro-9*H*-carbazol-9-yl)-3-(3,6-dihydro-2*H*-pyran-4-yl)benzoic acid

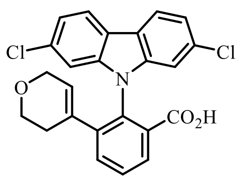

206 mg in 39% yield as a white solid. ^{1}H NMR (500 MHz, Chloroform-*d*) δ 8.06 (dd, *J* = 6.6, 2.8 Hz, 1H), 7.91 (d, *J* = 8.3 Hz, 2H), 7.66 – 7.60 (m, 2H), 7.20 (dd, *J* = 8.4, 1.8 Hz, 2H), 6.87 (d, *J* = 1.8 Hz, 2H), 5.44 – 5.42 (m, 1H), 3.79 (q, *J* = 2.7 Hz, 2H), 3.19 (t, *J* = 5.3 Hz, 2H), 1.68 – 1.65 (m, 2H). ^{13}C NMR (126 MHz, Chloroform-*d*) δ 167.8, 143.9, 142.5, 134.6, 132.8, 131.9, 131.5, 130.0, 129.6, 126.6, 121.2, 121.1, 120.8, 110.0, 65.1, 63.7, 28.3. HRMS (m/z, ESI): Calcd. for Chemical Formula: $C_{24}H_{16}Cl_2NO_3^-$ [M-H]$^-$: 436.0513, Found: 436.0516.

2-(2,7-dichloro-9*H*-carbazol-9-yl)-3-(naphthalen-1-yl)benzoic acid

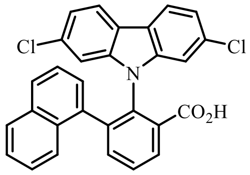

350 mg in 42% yield as a white solid. ^{1}H NMR (500 MHz, MeOD) δ 8.26 (dd, *J* = 6.8, 2.7 Hz, 1H), 7.90 – 7.84 (m, 2H), 7.78 (d, *J* = 8.7 Hz, 1H), 7.75 – 7.72 (m, 1H), 7.65 – 7.61 (m, 2H), 7.51 – 7.48 (m, 1H), 7.40 – 7.33 (m, 2H), 7.13 – 7.11 (m, 2H), 7.00 – 6.94 (m, 2H), 6.84 – 6.79 (m, 2H).^{13}C NMR (126 MHz, MeOD) δ 167.3, 143.1, 142.0, 141.8, 136.2, 134.4, 133.9, 133.4, 133.0, 131.3, 131.1, 130.9, 130.8, 129.0, 127.9, 127.9, 125.8, 125.7, 125.4, 124.9, 123.9, 121.1, 120.9, 120.6, 120.1, 119.8, 119.5, 110.3, 109.8. HRMS (m/z, ESI): Calcd. for Chemical Formula: $C_{29}H_{16}Cl_2NO_2^-$ [M-H]$^-$: 480.0564, Found: 480.0561.

3-chloro-2-(2,7-dichloro-9*H*-carbazol-9-yl)benzoic acid (63)

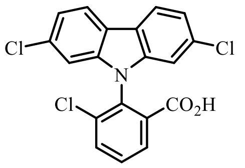

300 mg in 44% yield as a white solid. ^{1}H NMR (500 MHz, Acetone-d_6) δ 8.20 (dd, *J* = 8.1, 2.6 Hz, 3H), 8.04 (dd, *J* = 8.2, 1.5 Hz, 1H), 7.83 (t, *J* = 8.0 Hz, 1H), 7.29 (dd, *J* = 8.3, 1.8 Hz, 2H), 7.07 (d, *J* = 1.9 Hz, 2H). ^{13}C NMR

(126 MHz, Acetone-d_6) δ 164.7, 142.2, 135.4, 134.5, 133.7, 132.5, 131.7, 131.3, 130.7, 121.7, 121.6, 120.8, 109.8. HRMS (m/z, ESI): Calcd. for Chemical Formula: $C_{19}H_9Cl_3NO_2^-$ $[M-H]^-$: 387.9704, Found: 387.9705.

2-(2,7-dichloro-9*H*-carbazol-9-yl)benzoic acid (SI-2)

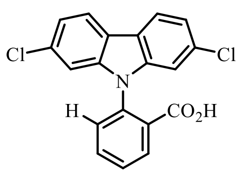

350 mg in 55% yield as a white solid. ^{1}H NMR (500 MHz, Acetone-d_6) δ 8.31 – 8.09 (m, 3H), 7.92 (t, J = 7.7 Hz, 1H), 7.78 (t, J = 7.7 Hz, 1H), 7.67 (d, J = 7.8 Hz, 1H), 7.36 – 7.05 (m, 4H).^{13}C NMR (126 MHz, Acetone-d_6) δ 165.5, 142.8, 135.4, 134.1, 132.3, 131.6, 130.5, 130.4, 129.6, 121.6, 121.5, 120.5, 109.6. HRMS (m/z, ESI): Calcd. for Chemical Formula: $C_{19}H_{12}Cl_2NO_2^+$ $[M+H]^+$: 356.0240, Found: 356.0243.

2-(2,7-dibromo-9*H*-carbazol-9-yl)-3-methylbenzoic acid (80)

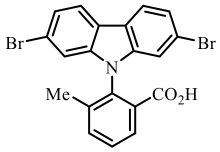

350 mg in 60% yield as a white solid.^{1}H NMR (500 MHz, Chloroform-d) δ 8.02 (dd, J = 7.8, 1.6 Hz, 1H), 7.92 (d, J = 8.3 Hz, 2H), 7.70 – 7.66 (m, 1H), 7.57 (t, J = 7.8 Hz, 1H), 7.36 (dd, J = 8.3, 1.7 Hz, 2H), 6.97 (d, J = 1.6 Hz, 2H), 1.91 (s, 3H). ^{13}C NMR (126 MHz, Chloroform-d) δ 168.0, 142.1, 139.8, 136.3, 134.5, 130.7, 129.5, 129.4, 123.4, 121.6, 121.6, 120.0, 112.4, 17.4. HRMS (m/z, ESI): Calcd. for Chemical Formula: $C_{20}H_{14}Br_2NO_2^+$ $[M+H]^+$: 457.9386, Found: 457.9381.

2-(3,6-dichloro-9*H*-carbazol-9-yl)-3-methylbenzoic acid (SI-3)

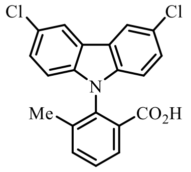

380 mg in 65% yield as a white solid.^{1}H NMR (500 MHz, Chloroform-*d*) δ 8.04 (d, *J* = 2.0 Hz, 2H), 7.96 (dd, *J* = 7.8, 1.6 Hz, 1H), 7.66 (dt, *J* = 7.6, 1.3 Hz, 1H), 7.54 (t, *J* = 7.8 Hz, 1H), 7.30 (dd, *J* = 8.6, 2.1 Hz, 2H), 6.76 (d, *J* = 8.7 Hz, 2H), 1.89 (s, 3H). ^{13}C NMR (126 MHz, Chloroform-*d*) δ 168.7, 139.9, 139.7, 136.1, 134.8, 130.4, 129.6, 129.3, 126.8, 125.4, 123.4, 120.4, 110.5, 17.3. HRMS (m/z, ESI): Calcd. for Chemical Formula: $C_{20}H_{14}Cl_2NO_2^+$ $[M+H]^+$: 370.0396, Found: 370.0393.

2. The procedure for the synthesis of 3-chloro-2-(3,6-dichloro-9,9-dimethylacridin-10(9*H*)-yl)benzoic acid.[7, 8]

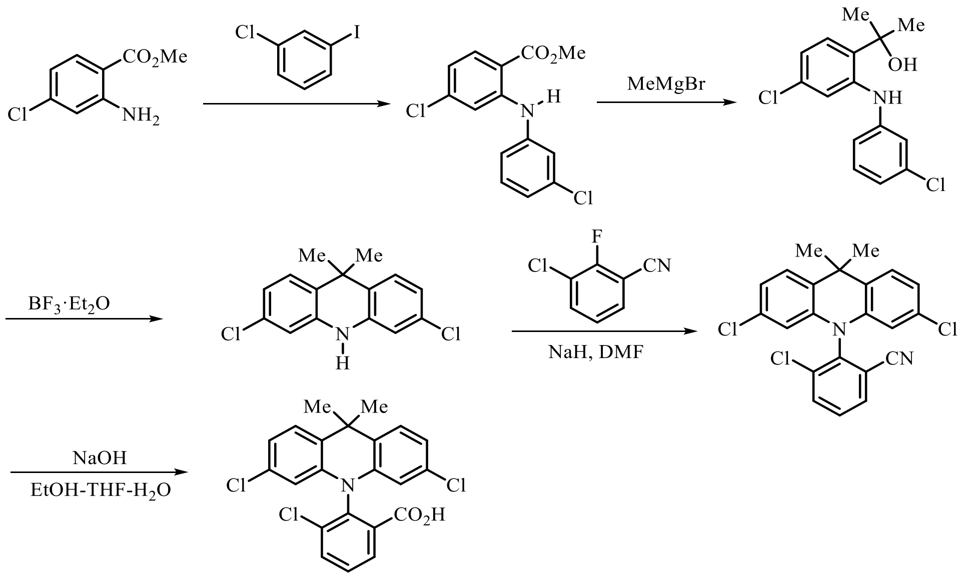

Under N_2 atmosphere, methyl 2-amino-4-chlorobenzoate (1.85 g, 10 mmol), 1-chloro-3-iodobenzene (2.38 g, 10 mmol), Pd_2dba_3 (183 mg, 0.2 mmol), $[(t\text{-Bu})_3P]HBF_4$ (116 mg, 0.4 mmol), and *t*-BuONa (2.88 g, 30 mmol) were added in 50 mL toluene. The resulting reaction mixture was stirred at 110 °C. After stirring overnight, the reaction was quenched with saturated $NH_4Cl_{(aq.)}$ at 0 °C and stirred for another 0.5 h. The mixture was extracted with EA (35 mL X 3) three times. The combined organic phases were washed with brine, dried over Na_2SO_4, and concentrated in vacuo.

The crude materials were purified by flash column chromatography with hexane and acetone (45:1 – 10:1) to give the desired product, 2.33 g in 79% yield as a white solid.

The above product (1.77 g, 6 mmol) was dissolved in 25 mL THF, followed by the addition of MeMgBr (3.0 M in diethyl ether, 8 mL, 24 mmol) at 0 °C. The resulting reaction mixture was warmed to room temperature and stirred at this temperature for 24 h. After the reaction was completed, the mixture was quenched with $NH_4Cl_{(aq.)}$ and extracted with EA (50 mL X 3) three times. The combined organic phases were washed with water and brine, dried over Na_2SO_4, and concentrated in vacuo. The crude product was directly used for next without further purification.

The above crude product was dissolved in 100 mL DCM, followed by the addition of $BF_3{\cdot}Et_2O$ (2.13 g, 15 mmol) at 0 °C over 2 h. The resulting reaction mixture was warmed to room temperature and stirred at this temperature for 1 h. After the reaction was completed, the mixture was quenched with $NH_4Cl_{(aq.)}$ and extracted with DCM (50 mL X 3) three times. The combined organic phases were washed with water and brine, dried over Na_2SO_4, and concentrated in vacuo. The crude product was purified by flash column chromatography with hexane to give product, 3,6-dichloro-9,9-dimethyl-9,10-dihydroacridine, 492 mg in 30% yield as a white solid. ^{1}H NMR (400 MHz, Chloroform-*d*) δ 7.27 (d, *J* = 8.3 Hz, 2H), 6.91 (d, *J* = 2.1 Hz, 1H), 6.89 (d, *J* = 2.1 Hz, 1H), 6.68 (d, *J* = 2.2 Hz, 2H), 6.14 (s, 1H), 1.53 (s, 6H). ^{13}C NMR (101 MHz, Chloroform-*d*) δ 138.9, 132.3, 127.6, 126.9, 121.0, 113.4, 35.9, 30.8.

3,6-dichloro-9,9-dimethyl-9,10-dihydroacridine (492 mg, 1.78 mmol) was added into the mixture of NaH (60% dispersion in mineral oil, 107 mg, 2.67 mmol) and 6 mL DMF at 0 °C, and stirred for 1 h at room temperature. After that, 3-chloro-2-fluorobenzonitrile (414 mg, 2.67 mmol) was added to the above reaction mixture. Then the resulting reaction mixture was heated to 120 °C and stirred

overnight at this temperature. After the reaction was completed, the mixture was quenched with NH_4Cl (aq.) and extracted with EA (15 mL X 3) three times. The combined organic phases were washed with water and brine, dried over Na_2SO_4, and concentrated in vacuo. The crude material was directly used for next without further purification.

Under the N_2 atmosphere, the above crude product was dissolved in 3 mL THF, 3 mL MeOH, and 8 M NaOH (aq.) (3 mL, 24 mmol). The resulting reaction mixture was heated to reflux and stirred at this temperature for 2 d. Then, the reaction was cooled to room temperature, the mixture was neutralized with 1 M HCl (aq.) to pH 2 – 3 and extracted with EA (20 mL X 3) three times. The combined organic phases were washed with brine, dried over Na_2SO_4, and concentrated in vacuo. The crude materials were purified by flash column chromatography with hexane and acetone (15:1 – 2:1) to give the desired product, 450 mg in 59% yield as a yellowish solid. ^{1}H NMR (500 MHz, Chloroform-*d*) δ 8.16 (dd, *J* = 7.9, 1.5 Hz, 1H), 7.92 (dd, *J* = 8.1, 1.6 Hz, 1H), 7.62 (t, *J* = 8.0 Hz, 1H), 7.32 (d, *J* = 8.4 Hz, 2H), 6.87 (dd, *J* = 8.3, 2.1 Hz, 2H), 5.82 (d, *J* = 2.0 Hz, 2H), 1.70 (s, 3H), 1.45 (s, 3H). ^{13}C NMR (126 MHz, Chloroform-*d*) δ 168.3, 139.2, 137.9, 137.0, 136.5, 132.4, 132.3, 132.2, 130.3, 128.3, 127.7, 121.2, 112.5, 35.4, 34.5, 32.7. HRMS (m/z, ESI): Calcd. for Chemical Formula: $C_{22}H_{15}Cl_3NO_2^-$ [M-H]$^-$: 430.0174, Found: 430.0175.

3. The general procedure for the synthesis of 3-chloro-2-(2,8-dichloro-10*H*-phenoxazin-10-yl)benzoic acid.[7, 9]

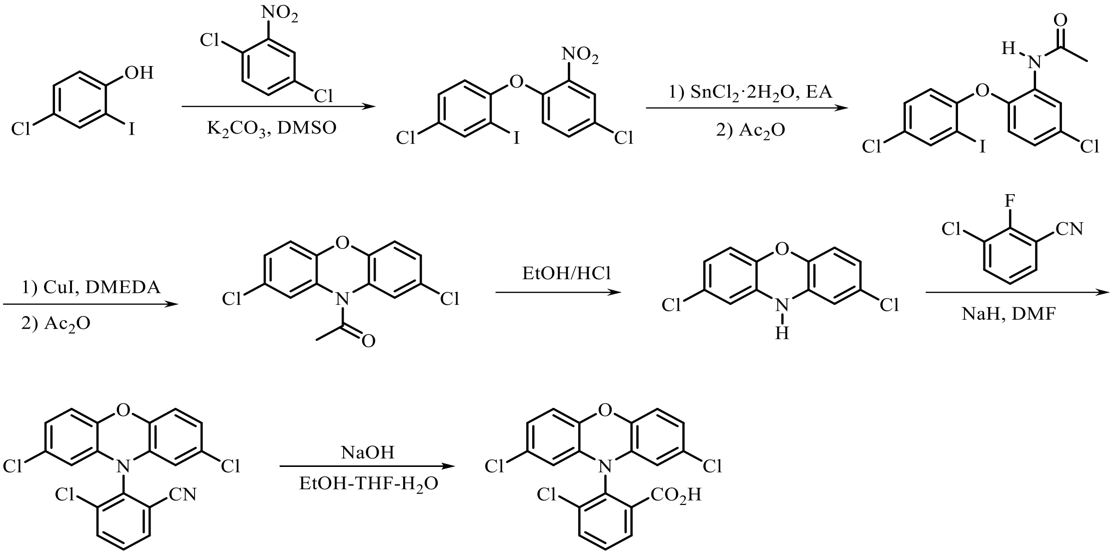

Under the N_2 atmosphere, 4-chloro-2-iodophenol (4.82 g, 18.94 mmol), 2.5-Dichloronitrobenzene (3.64 g, 18.94 mmol), and K_2CO_3 (5.12 g, 37.08 mmol) were added in 30 mL DMSO, then the resulting reaction mixture was heated to 100 ºC, and stirred for 1.5 h at this temperature. After that, it was cooled to room temperature and quenched with saturated NH_4Cl (aq.) at 0 ºC. The mixture was extracted with EA (35 mL X 3) three times. The combined organic phase was washed with brine, dried over Na_2SO_4, and concentrated in vacuo. The crude product was used directly without further purification.

Under the N_2 atmosphere, the above crude product and $SnCl_2 \cdot 2H_2O$ (20.92 g, 92.7 mmol) were added in 100 mL EA. The resulting reaction mixture was stirred overnight at room temperature. After that, the reaction was added to 500 mL H_2O and the mixture was extracted with EA (45 mL X 3) three times. The combined organic phase was washed with H_2O, and brine, dried over Na_2SO_4, and concentrated in vacuo. The crude product was used directly without further purification.

Under the N_2 atmosphere, the above crude product was added in 20 mL Ac_2O, and stirred overnight at room temperature. After that, the reaction was added to 100 mL H_2O and the mixture was extracted with EA (45 mL X 3) three times. The combined organic phase was washed with H_2O,

and brine, dried over Na_2SO_4, and concentrated in vacuo. The crude materials were purified by flash column chromatography with hexane and acetone (60:1 – 10:1) to give the desired product, *N*-(5-chloro-2-(4-chloro-2-iodophenoxy)phenyl)acetamide, 4.22 g in 52% yield as a white solid.

Under the N_2 atmosphere, *N*-(5-chloro-2-(4-chloro-2-iodophenoxy)phenyl)acetamide (2.1 g, 5 mmol), K_2CO_3 (2.07 g, 15 mmol), CuI (47.5 mg, 0.25 mmol), and DMEDA (69 mg, 0.5 mmol) were added in 15 mL toluene. After that, the reaction mixture was heated to 120 °C and stirred overnight at this temperature. After that, the reaction was quenched with H_2O. The mixture was extracted with EA (30 mL X 3) three times. The combined organic phase was washed with H_2O, and brine, dried over Na_2SO_4, and concentrated in vacuo. The crude materials were purified by flash column chromatography with hexane and acetone (30:1 – 10:1) to give the desired product, 1-(2,8-dichloro-10H-phenoxazin-10-yl)ethan-1-one, 952 mg in 65% yield as a white solid.

Under the N_2 atmosphere, 1-(2,8-dichloro-10*H*-phenoxazin-10-yl)ethan-1-one (640 mg, 2.18 mmol) was discovered in EtOH (20 mL) and Conc.HCl $_{(aq.)}$ (5 mL), then the mixture was heated to 75 °C, and stirred overnight at this temperature. After that the mixture was cooled to room temperature, quenched with H_2O. The mixture was extracted with EA (30 mL X 3) three times. The combined organic phase was washed with H_2O, and brine, dried over Na_2SO_4, and concentrated in vacuo. The crude materials were purified by flash column chromatography with hexane and acetone (30:1 – 10:1) to give the desired product, 2,8-dichloro-10*H*-phenoxazine, 530 mg in 97% yield as a white solid. ^{1}H NMR (400 MHz, Acetonitrile-d_3) δ 6.69 (s, 1H), 6.63 (dd, J = 8.5, 2.3 Hz, 2H), 6.58 (d, J = 8.5 Hz, 2H), 6.47 (d, J = 2.3 Hz, 2H). ^{13}C NMR (101 MHz, Acetonitrile-d_3) δ 142.5, 133.2, 128.7, 121.2, 117.0, 113.7.

Under the N_2 atmosphere, 2,8-dichloro-10*H*-phenoxazine (503 mg, 2 mmol) was added into the

mixture of NaH (60% dispersion in mineral oil, 120 mg, 3 mmol) and 5 mL DMF at 0 °C, and stirred for 1 h at room temperature. After that, 3-chloro-2-fluorobenzonitrile (465 mg, 3 mmol) was added to the above reaction mixture. Then the resulting reaction mixture was heated to 120 °C and stirred overnight at this temperature. After the reaction was completed, the mixture was quenched with $NH_4Cl_{(aq.)}$ and extracted with EA (15 mL X 3) three times. The combined organic phases were washed with water and brine, dried over Na_2SO_4, and concentrated in vacuo. The crude material was directly used for next without further purification.

Under the N_2 atmosphere, the above crude product was dissolved in 3 mL THF, 3 mL MeOH, and 8M $NaOH_{(aq.)}$ (3 mL, 24 mmol). The resulting reaction mixture was heated to reflux and stirred at this temperature for 2 d. Then, the reaction was cooled to room temperature, the mixture was neutralized with 1 M $HCl_{(aq.)}$ to pH 2 – 3 and extracted with EA (20 mL X 3) three times. The combined organic phases were washed with brine, dried over Na_2SO_4, and concentrated in vacuo. The crude materials were purified by flash column chromatography with hexane and acetone (30:1 – 2:1) to give the desired product, 527 mg in 65% yield as a white solid. ^1H NMR (500 MHz, Methanol-*d*) δ 8.13 (dd, *J* = 7.9, 1.5 Hz, 1H), 7.98 (dd, *J* = 8.2, 1.5 Hz, 1H), 7.71 (t, *J* = 8.0 Hz, 1H), 6.65 (d, *J* = 1.3 Hz, 4H), 5.63 (d, *J* = 1.5 Hz, 2H). ^{13}C NMR (126 MHz, Tetrahydrofuran-d_8) δ 164.2, 142.6, 137.0, 135.1, 134.3, 133.7, 133.1, 131.8, 130.8, 128.1, 121.2, 116.3, 112.4. HRMS (m/z, ESI): Calcd. for Chemical Formula: $C_{19}H_9Cl_3NO_3^-$ $[M-H]^-$: 403.9653, Found: 403.9653.

4. The procedure for the synthesis of 1-(2,7-dichloro-9*H*-carbazol-9-yl)-5-ethyl-1*H*-pyrrole-2-carboxylic acid (34).[10, 11]

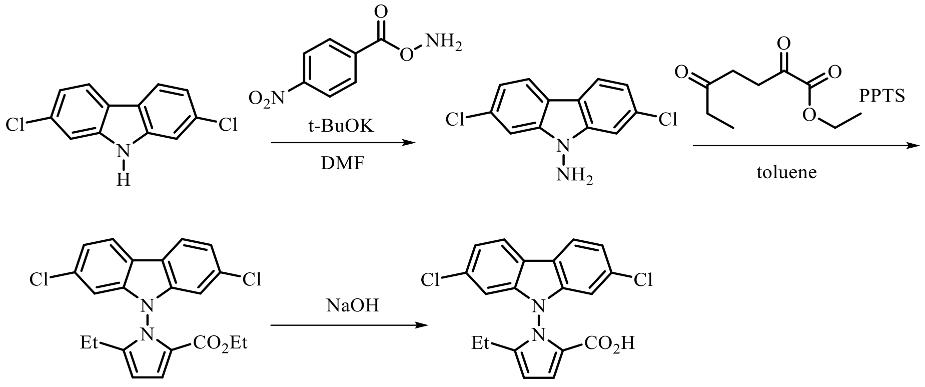

Under the N_2 atmosphere, 2,7-dichloro-9*H*-carbazole (470 mg, 2 mmol) was added in 10 mL DMF, then *t*-BuOK (272 mg, in 4 mL DMF, 2.4 mmol) was added dropwise at 0 ℃. The reaction mixture was stirred for 1 h at room temperature. After that, O-(4-nitrobenzoyl)hydroxylamine (400 mg, in 4 mL DMF, 2.4 mmol) was added dropwise into the reaction mixture at 0 ℃, and then warmed to room temperature. After stirring for 24 h, the reaction was quenched with saturated $NH_4Cl_{(aq.)}$ at 0 ℃. The mixture was extracted with EA (20 mL X 3) three times. The combined organic phase was washed with brine, dried over Na_2SO_4, and concentrated in vacuo. The crude materials were purified by flash column chromatography with hexane and EA (50:1 – 10:1) to give the desired product, 2,7-dichloro-9*H*-carbazol-9-amine, 410 mg in 82% yield as a white solid.

Under the N_2 atmosphere, 2,7-dichloro-9*H*-carbazol-9-amine (250 mg, 1 mmol), ethyl 2,5-dioxoheptanoate[11] (186 mg, 1 mmol), and *p*-methoxybenzenesulfonic acid (50 mg, 0.2 mmol) were added in 10 mL toluene, then the reaction mixture stirred overnight at 80 ℃. After that, quenched the reaction with H_2O, and extracted with EA (15 mL X 3) three times. The combined organic phases were washed with water and brine, dried over Na_2SO_4, and concentrated in vacuo. The crude materials were purified by flash column chromatography with hexane and acetone (350:1 – 50:1) to give the desired product, 280 mg in 70% yield as a white solid.

The above product (280 mg, 0.7 mmol) was dissolved in 2 mL THF, 2 mL EtOH and 2 mL H_2O,

followed by the addition of NaOH (126 mg, 3.15 mmol). The resulting reaction mixture was stirred at room temperature for 24 h. After that, the reaction mixture was neutralized with 1 M HCl $_{(aq.)}$ to pH 2 – 3 and extracted with EA (8 mL X 3) three times. The combined organic phases were washed with water and brine, dried over Na_2SO_4, and concentrated in vacuo. The crude materials were purified by flash column chromatography with hexane and acetone (25:1 – 5:1) to give the desired product, 214 mg in 82% yield as a pink solid. ^{1}H NMR (500 MHz, Chloroform-*d*) δ 7.93 (d, *J* = 8.3 Hz, 2H), 7.27 (d, *J* = 1.8 Hz, 1H), 7.25 (d, *J* = 1.8 Hz, 1H), 7.18 (d, *J* = 4.4 Hz, 1H), 6.84 (d, *J* = 1.8 Hz, 2H), 6.23 (d, *J* = 4.4 Hz, 1H), 2.25 (q, *J* = 7.5 Hz, 2H), 1.11 (t, *J* = 7.6 Hz, 3H). ^{13}C NMR (126 MHz, Chloroform-*d*) δ 161.9, 144.8, 141.4, 132.8, 122.0, 121.4, 120.3, 119.8, 119.7, 108.6, 106.1, 18.5, 12.2. HRMS (m/z, ESI): Calcd. for Chemical Formula: $C_{19}H_{13}Cl_2N_2O_2^-$ [M-H]$^-$: 371.0360, Found: 371.0362.

5. The general procedure B for the synthesis substrates.[10]

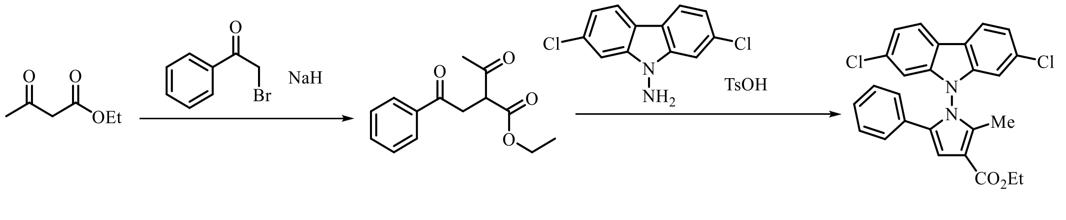

Ethyl 1-(2,7-dichloro-9*H*-carbazol-9-yl)-2-methyl-5-phenyl-1*H*-pyrrole-3-carboxylate (SI-4)

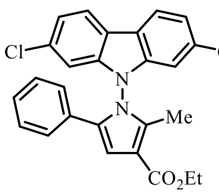

Under the N_2 atmosphere, ethyl acetoacetate (572 mg, 4 mmol) was added dropwise in the mixture of NaH (60% dispersion in mineral oil, 176 mg, 4.4 mmol) and THF (8 mL) at room temperature, then the reaction mixture was stirred for 1 h at this temperature. After that, 2-bromoacetophenone (in 2 mL THF, 871 mg, 4.4 mmol) was added dropwise into the above mixture. The resulting reaction mixture was stirred at room temperature for 14 h. After that, quenched the reaction with H_2O, and extracted with EA (15

mL X 3) three times.The combined organic phases were washed with brine, dried over Na_2SO_4, and concentrated in vacuo. The crude materials were purified by flash column chromatography with hexane and acetone (50:1 – 10:1) to give the desired product, ethyl 2-acetyl-4-oxo-4-phenylbutanoate, 493 mg in 50 % yield as a colorless oil.

Under the N_2 atmosphere, ethyl 2-acetyl-4-oxo-4-phenylbutanoate (333 mg, 1.34 mmol), 2,7-dichloro-9*H*-carbazol-9-amine (336 mg, 1.34 mmol), and TsOH (23 mg, 0.134 mmol) were dissolved in 8 mL toluene. The resulting reaction mixture was stirred at 85 °C for 1 d. After that, the reaction mixture was quenched with H_2O and extracted with EA (10 mL X 3) three times. The combined organic phase was washed with water and brine, dried over Na_2SO_4, and concentrated in vacuo. The crude materials were purified slowly and carefully by flash column chromatography with hexane and acetone (250:1 – 30:1) to give the desired product, 402 mg in 65% yield as a white solid. ^{1}H NMR (500 MHz, Chloroform-*d*) δ 7.94 (d, *J* = 8.4 Hz, 2H), 7.29 (dd, *J* = 8.3, 1.8 Hz, 2H), 7.11 – 7.05 (m, 5H), 7.03 (d, *J* = 1.8 Hz, 2H), 6.97 (s, 1H), 4.37 (q, *J* = 7.1 Hz, 2H), 2.08 (s, 3H), 1.41 (t, *J* = 7.1 Hz, 3H). ^{13}C NMR (126 MHz, Chloroform-*d*) δ 164.7, 140.9, 138.1, 133.7, 133.3, 129.8, 128.6, 127.7, 126.6, 122.6, 121.7, 119.5, 112.8, 109.0, 108.6, 60.1, 14.5, 10.2. HRMS (m/z, ESI): Calcd. for Chemical Formula: $C_{26}H_{21}Cl_2N_2O_2^+$ $[M+H]^+$: 463.0975, Found: 463.0973.

Ethyl 1-(2,7-dichloro-9*H*-carbazol-9-yl)-2-ethyl-5-phenyl-1*H*-pyrrole-3-carboxylate

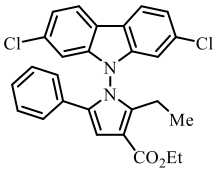

228 mg in 55% yield as a white solid. ^{1}H NMR (500 MHz, Chloroform-*d*) δ 7.93 (d, *J* = 8.4 Hz, 2H), 7.28 (dd, *J* = 8.4, 1.8 Hz, 2H), 7.09 – 6.99 (m, 7H), 6.98 (s, 1H), 4.38 (q, *J* = 7.1 Hz, 2H), 2.52 (q, *J* = 7.5 Hz, 2H), 1.42 (t, *J* = 7.1 Hz, 3H), 0.90 (t, *J* = 7.5 Hz, 3H). ^{13}C NMR (126 MHz, Chloroform-*d*) δ 164.4, 144.1, 141.2, 133.6,

133.2, 128.5, 127.7, 126.7, 122.5, 121.6, 119.3, 112.2, 109.1, 109.1, 60.1, 18.3, 14.5, 14.2. HRMS (m/z, ESI): Calcd. for Chemical Formula: $C_{27}H_{23}Cl_2N_2O_2^+$ $[M+H]^+$: 477.1131, Found: 477.1133.

Methyl 2-butyl-1-(2,7-dichloro-9*H*-carbazol-9-yl)-5-phenyl-1*H*-pyrrole-3-carboxylate

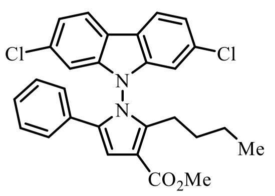

262 mg in 52% yield as a white solid. ^{1}H NMR (500 MHz, Chloroform-*d*) δ 7.92 (d, *J* = 8.3 Hz, 2H), 7.27 (d, *J* = 8.3 Hz, 2H), 7.08 – 6.99 (m, 7H), 6.96 (s, 1H), 3.90 (s, 3H), 2.52 – 2.45 (m, 2H), 1.29 – 1.20 (m, 2H), 1.02 (h, *J* = 7.4 Hz, 2H), 0.53 (t, *J* = 7.3 Hz, 3H). ^{13}C NMR (126 MHz, Chloroform-*d*) δ 164.8, 143.2, 141.2, 133.8, 133.2, 129.7, 128.6, 127.8, 126.7, 122.5, 121.6, 119.3, 112.2, 109.2, 108.9, 51.3, 31.5, 24.4, 22.2, 13.2. HRMS (m/z, ESI): Calcd. for Chemical Formula: $C_{28}H_{25}Cl_2N_2O_2^+$ $[M+H]^+$: 491.1288, Found: 491.1280.

Ethyl 1-(2,7-dichloro-9*H*-carbazol-9-yl)-2-methyl-5-(3-nitrophenyl)-1*H*-pyrrole-3-carboxylate

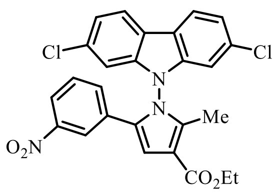

236 mg in 48% yield as a white solid. ^{1}H NMR (500 MHz, Chloroform-*d*) δ 7.98 (d, *J* = 8.4 Hz, 2H), 7.93 – 7.87 (m, 2H), 7.34 – 7.32 (m, 3H), 7.23 (t, *J* = 8.0 Hz, 1H), 7.15 (s, 1H), 7.02 (d, *J* = 1.8 Hz, 2H), 4.39 (q, *J* = 7.1 Hz, 2H), 2.16 (s, 3H), 1.43 (t, *J* = 7.1 Hz, 3H). ^{13}C NMR (126 MHz, Chloroform-*d*) δ 164.2, 148.4, 140.6, 139.4, 133.6, 131.5, 131.2, 130.9, 129.8, 123.1, 122.2, 122.0, 121.1, 119.6, 113.3, 110.4, 108.7, 60.4, 14.5, 10.2. HRMS (m/z, ESI): Calcd. for Chemical Formula: $C_{26}H_{20}Cl_2N_3O_4^+$ $[M+H]^+$: 508.0825, Found: 508.0819.

Ethyl 1-(2,7-dichloro-9*H*-carbazol-9-yl)-5-(3-methoxyphenyl)-2-methyl-1*H*-pyrrole-3-carboxylate

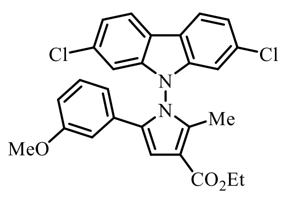

231 mg in 47% yield as a white solid. ^{1}H NMR (500 MHz, Chloroform-*d*) δ 7.94 (d, *J* = 8.3 Hz, 2H), 7.29 (dd, *J* = 8.3, 1.8 Hz, 2H), 7.04 (d, *J* = 1.8 Hz, 2H), 7.01 – 6.95 (m, 2H), 6.74 – 6.72 (m, 1H), 6.64 – 6.60 (m, 1H), 6.53 (t, *J* = 2.1 Hz, 1H), 4.38 (q, *J* = 7.1 Hz, 2H), 3.33 (s, 3H), 2.13 (s, 3H), 1.42 (t, *J* = 7.1 Hz, 3H). ^{13}C NMR (126 MHz, Chloroform-*d*) δ 164.7, 159.4, 140.9, 138.2, 133.6, 133.4, 130.9, 129.7, 122.6, 121.7, 119.4, 119.1, 114.5, 112.8, 110.8, 109.0, 108.8, 60.1, 54.7, 14.5, 10.2. HRMS (m/z, ESI): Calcd. for Chemical Formula: $C_{27}H_{23}Cl_2N_2O_3^+$ [M+H]$^+$: 493.1080, Found: 493.1071.

Ethyl 1-(2,7-dichloro-9*H*-carbazol-9-yl)-2-methyl-5-(naphthalen-2-yl)-1*H*-pyrrole-3-carboxylate

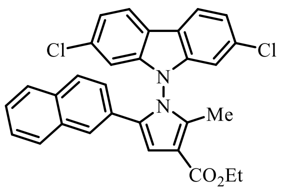

276 mg in 54% yield as a white solid. ^{1}H NMR (500 MHz, Chloroform-*d*) δ 7.94 (d, *J* = 8.3 Hz, 2H), 7.66 – 7.62 (m, 1H), 7.56 (d, *J* = 8.6 Hz, 1H), 7.46 (d, *J* = 1.8 Hz, 1H), 7.42 – 7.38 (m, 1H), 7.36 – 7.31 (m, 2H), 7.31 – 7.26 (m, 3H), 7.12 – 7.07 (m, 3H), 4.40 (q, *J* = 7.1 Hz, 2H), 2.15 (s, 3H), 1.44 (t, *J* = 7.1 Hz, 3H). ^{13}C NMR (126 MHz, Chloroform-*d*) δ 164.7, 140.9, 138.5, 133.7, 133.4, 133.1, 132.5, 128.3, 128.1, 127.4, 127.1, 126.3, 126.2, 125.3, 124.5, 122.6, 121.7, 119.5, 113.0, 109.2, 109.0, 60.2, 14.6, 10.2. HRMS (m/z, ESI): Calcd. for Chemical Formula: $C_{30}H_{23}Cl_2N_2O_2^+$ [M+H]$^+$: 513.1131, Found: 513.1123.

Ethyl 5-(benzofuran-2-yl)-1-(2,7-dichloro-9*H*-carbazol-9-yl)-2-methyl-1*H*-pyrrole-3-

carboxylate

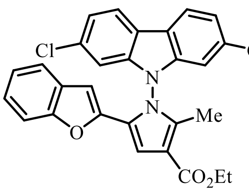

226 mg in 45% yield as a white solid. ^{1}H NMR (500 MHz, Chloroform-*d*) δ 8.05 (d, *J* = 8.3 Hz, 2H), 7.37 – 7.34 (m, 3H), 7.31 (d, *J* = 8.5 Hz, 1H), 7.18 – 7.13 (m, 2H), 7.07 – 7.02 (m, 3H), 5.38 (s, 1H), 4.40 (q, *J* = 7.1 Hz, 2H), 2.19 (s, 3H), 1.44 (t, *J* = 7.1 Hz, 3H). ^{13}C NMR (126 MHz, Chloroform-*d*) δ 164.3, 153.8, 145.7, 140.2, 139.3, 133.6, 128.4, 124.5, 124.3, 123.0, 122.9, 121.8, 120.8, 119.6, 113.3, 110.8, 109.5, 109.0, 100.4, 60.3, 14.5, 10.0. HRMS (m/z, ESI): Calcd. for Chemical Formula: $C_{28}H_{21}Cl_2N_2O_3^+$ [M+H]$^+$: 503.0924, Found: 503.0921.

Ethyl 1-(2,7-dichloro-9*H*-carbazol-9-yl)-2-methyl-5-(thiophen-2-yl)-1*H*-pyrrole-3-carboxylate

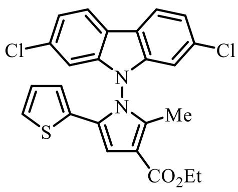

229 mg in 49% yield as a white solid. ^{1}H NMR (500 MHz, Chloroform-*d*) δ 7.99 (d, *J* = 8.3 Hz, 2H), 7.32 (dd, *J* = 8.3, 1.8 Hz, 2H), 7.04 – 7.00 (m, 3H), 6.96 (dd, *J* = 5.0, 1.2 Hz, 1H), 6.73 (dd, *J* = 5.1, 3.7 Hz, 1H), 6.59 (dd, *J* = 3.8, 1.2 Hz, 1H), 4.38 (q, *J* = 7.1 Hz, 2H), 2.11 (s, 3H), 1.42 (t, *J* = 7.1 Hz, 3H). ^{13}C NMR (126 MHz, Chloroform-*d*) δ 164.5, 140.6, 137.8, 133.4, 130.7, 127.5, 127.3, 124.8, 123.8, 122.8, 121.7, 119.7, 112.9, 109.1, 108.0, 60.2, 14.5, 10.1. HRMS (m/z, ESI): Calcd. for Chemical Formula: $C_{24}H_{19}Cl_2N_2O_2S^+$ [M+H]$^+$: 469.0539, Found: 469.0533.

Ethyl 5-cyclopropyl-1-(2,7-dichloro-9*H*-carbazol-9-yl)-2-methyl-1*H*-pyrrole-3-carboxylate

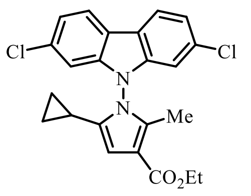

158 mg in 37% yield as a white solid. ^{1}H NMR (500 MHz, Chloroform-*d*) δ 7.99 (d, *J* = 8.3 Hz, 2H), 7.32 (dd, *J* = 8.3, 1.8 Hz, 2H), 7.01 (d, *J* = 1.8 Hz, 2H), 6.34 (d, *J* = 1.0 Hz, 1H), 4.33 (q, *J* = 7.1 Hz, 2H), 2.17 (s, 3H), 1.38 (t, *J* = 7.1 Hz, 3H), 1.11 – 1.04 (m, 1H), 0.53 – 0.41 (m, 4H). ^{13}C NMR (126 MHz, Chloroform-*d*) δ 164.9, 140.9, 136.3, 135.8, 133.2, 122.4, 121.6, 119.6, 111.1, 108.9, 104.4, 59.9, 14.5, 10.2, 6.2, 5.5. HRMS (m/z, ESI): Calcd. for Chemical Formula: $C_{23}H_{21}Cl_2N_2O_2^+$ $[M+H]^+$: 427.0975, Found: 427.0971.

6. The procedure for the synthesis of 1-(3,6-dichloro-9*H*-carbazol-9-yl)-2-methyl-5-phenyl-1*H*-pyrrole-3-carboxylic acid (60).[10]

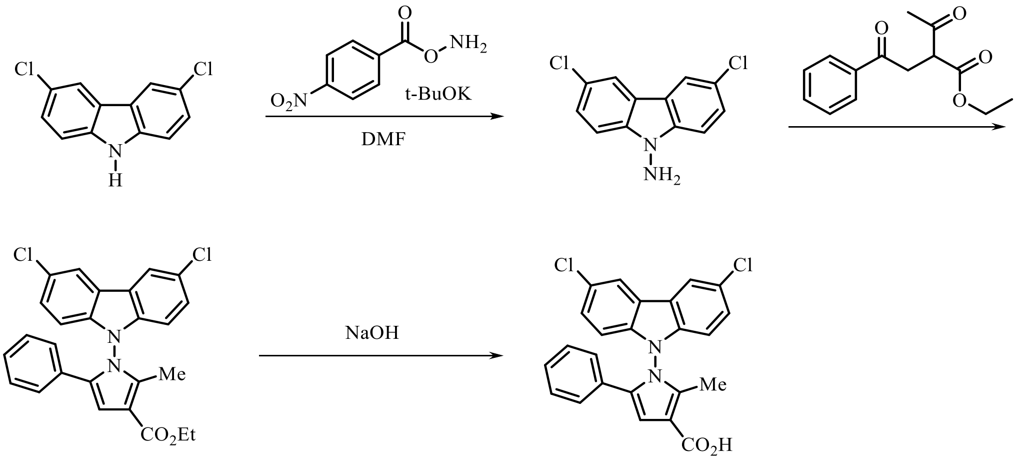

Under the N_2 atmosphere, 3,6-dichlorocarbazole (470 mg, 2 mmol) was added in 10 mL DMF, then *t*-BuOK (272 mg, in 4 mL DMF, 2.4 mmol) was added dropwise at 0 °C. The reaction mixture was stirred for 1 h at room temperature. After that, O-(4-nitrobenzoyl)hydroxylamine (400 mg, in 4 mL DMF, 2.4 mmol) was added dropwise into the reaction mixture at 0 °C, and then warmed to room temperature. After stirring for 24 h, the reaction was quenched with saturated $NH_4Cl_{(aq.)}$ at 0 °C. The mixture was extracted with EA (20 mL X 3) three times. The combined organic phase was

washed with brine, dried over Na_2SO_4, and concentrated in vacuo. The crude materials were purified by flash column chromatography with hexane and EA (50:1 – 10:1) to give the desired product, 2,7-dichloro-9H-carbazol-9-amine, 410 mg in 82% yield as a white solid.

Under the N_2 atmosphere, 2,7-dichloro-9*H*-carbazol-9-amine (250 mg, 1 mmol), ethyl 2,5-dioxoheptanoate (186 mg, 1 mmol), and *p*-Methoxybenzenesulfonic acid (50 mg, 0.2 mmol) were added in 10 mL toluene, then the reaction mixture stirred overnight at 85 °C. After that, quenched the reaction with H_2O, and extracted with EA (15 mL X 3) three times. The combined organic phases were washed with water and brine, dried over Na_2SO_4, and concentrated in vacuo. The crude materials were purified by flash column chromatography with hexane and acetone (250:1 – 50:1) to give the desired product, 280 mg in 70% yield as a white solid.

The above product (280 mg, 0.7 mmol) was dissolved in 2 mL THF, 2 mL EtOH and 2 mL H_2O, followed by the addition of NaOH (126 mg, 3.15 mmol). The resulting reaction mixture was stirred at room temperature for 24 h. After that, the reaction mixture was neutralized with 1 M HCl $_{(aq.)}$ to pH 2 – 3 and extracted with EA (8 mL X 3) three times. The combined organic phases were washed with water and brine, dried over Na_2SO_4, and concentrated in vacuo. The crude materials were purified by flash column chromatography with hexane and acetone (35:1 – 5:1) to give the desired product, 214 mg in 82% yield as a pink solid. ^1H NMR (500 MHz, Chloroform-*d*) δ 8.01 (d, *J* = 2.0 Hz, 2H), 7.41 (dd, *J* = 8.6, 2.0 Hz, 2H), 7.11 – 7.04 (m, 5H), 7.01 (s, 1H), 7.00 (d, *J* = 3.6 Hz, 2H), 2.11 (s, 3H). ^{13}C NMR (126 MHz, Chloroform-*d*) δ 169.8, 139.7, 138.7, 134.3, 129.6, 128.6, 128.0, 128.0, 127.5, 126.6, 121.6, 120.9, 111.6, 109.9, 108.8, 10.3. HRMS (m/z, ESI): Calcd. for Chemical Formula: $C_{24}H_{15}Cl_2N_2O_2^-$ [M-H]$^-$: 433.0516, Found: 433.0515.

Side chain parameters for amino acids[12]

Table S1

Amino acid	π[α]	[-][b]	ν[c]	ν_{reg}[d]	L[e]	B_1[f]	B_5[g]
Ala	0.31	1.28	0.52	0.53	2.87	1.52	2.04
Leu	1.70	2.59	0.98	0.92	4.92	1.52	4.45
Val	1.22	3.67	0.76	0.89	4.11	1.90	3.17
Ile	1.80	4.19	1.02	0.96	4.92	1.90	3.49
Bug	1.51	5.91	1.24	1.24	4.11	2.60	3.17

[a]Hydrophobicity, [b]Graph shape index, [c]Upsilon steric parameter, [d]Smoothed upsilon steric parameter, [e,f,g]STERIMOL length, maximum, and minimum width, respectively.

Table S1-continued

Amino acid	α[h]	ν_V[i]	δH_c[j]	σ_1[k]	pka(RCO_2H)	^{13}C[#] (ppm)	ln er
Ala	0.046	1.0	7.3	-0.01	4.76	49.0	1.48
Leu	0.186	4.0	10.1	-0.01	4.79	51.6	1.86
Val	0.140	3.0	17.2	0.01	4.86	58.2	2.44
Ile	0.186	4.0	16.1	-0.01	4.81	53.8	2.04
Bug	0.186	4.0	21.0	-0.01		60.3	2.67

[h]Polarizability, [i]Normalized, [j] ^1H NMR chemical shift, [k]Localized electrical effect

[#]^{13}C NMR chemical shift of α-carbon (^{13}C) of ligands:

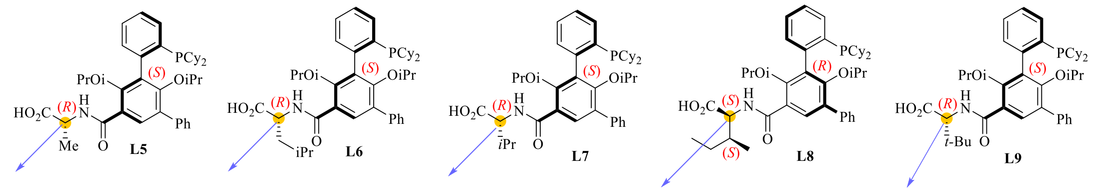

49.0 50%, 81.5:18.5 er 51.6 61%, 86.5:13.5 er 58.2 68%, 92:8 er 53.8 65%, 11.5:88.5 er 60.3 71%, 96:4 er

General procedure for Suzuki cross-coupling desymmetrization reactions of N–C and N–N carbazole acid substrates.

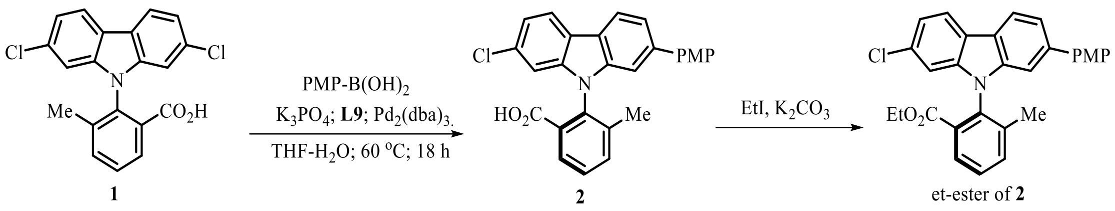

Under N_2 atmosphere, a mixture of $Pd_2(dba)_3$ (2.3 mg, 0.0025 mmol) and **L9** (3.5 mg, 0.005 mmol) was added to 0.5 mL THF, the mixture was then stirred at room temperature for 20 min. The resulting metal-ligand complex solution was added to a reaction flask containing 4.5 mL THF solution of 2-(2,7-dichloro-9*H*-carbazol-9-yl)-3-methylbenzoic acid **1** (92.5 mg, 0.25 mmol) and *p*-methoxyphenylboronic acid (53.2 mg, 0.35 mmol), and K_3PO_4 (265 mg, 1.25 mmol), followed by addition of 0.10 mL H_2O. Then the resulting reaction mixture was stirred at 60 °C for 18 h. The reaction was then quenched with water, neutralized to pH 3 – 5 with 1.0 M HCl $_{(aq.)}$, and extracted with ethyl acetate (4.0 mL X 3) three times. The combined organic phase was washed with brine, dried over Na_2SO_4, and concentrated in vacuo. The organic layer was concentrated in vacuo and purified by flash column chromatography with hexane and acetone as the eluents to give the desired acid product.

Ester Product : After the obtained crude product was dissolved in DMF (1.0 mL), and treated with EtI or MeI (0.5 mmol) and K_2CO_3 (1.0 mmol). The reaction mixture was stirred overnight at room temperature. Then the mixture was diluted with ethyl acetate (20.0 mL) and washed with water, brine, and dried over Na_2SO_4. The organic layer was concentrated in vacuo and purified by flash column chromatography with hexane and acetone as the eluents to give the desired product.

Characterization data of desymmetrizating products from N–C and N–N carbazole acid substrates

Ethyl (*R*)-2-(2-chloro-7-(4-methoxyphenyl)-9*H*-carbazol-9-yl)-3-methylbenzoate (et-ester of 2)

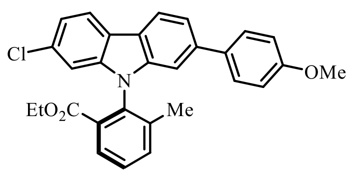

White solid, 83 mg, 69% (isolated yield), $[\alpha]_D^{20}$ = +27.20 (*c* 1.000 $CHCl_3$) for 96:4 er. ^1H NMR (500 MHz, Chloroform-*d*) δ 8.13 (dd, *J* = 8.1, 0.6 Hz, 1H), 8.05 (d, *J* = 8.3 Hz, 1H), 8.02 – 7.97 (m, 1H), 7.68 – 7.66 (m, 1H), 7.61 – 7.53 (m, 3H), 7.50 (dd, *J* = 8.1, 1.6 Hz, 1H), 7.25 (dd, *J* = 8.3, 1.8 Hz, 1H), 7.07 (dd, *J* = 1.6, 0.6 Hz, 1H), 6.99 – 6.93 (m, 3H), 3.84 (s, 3H), 3.72 (q, *J* = 7.1 Hz, 2H), 2.04 (s, 3H), 0.48 (t, *J* = 7.1 Hz, 3H). ^{13}C NMR (126 MHz, Chloroform-*d*) δ 166.0, 159.2, 142.3, 142.3, 139.5, 139.4, 135.1, 134.3, 134.2, 132.0, 131.6, 129.7, 129.2, 128.5, 121.7, 121.4, 121.1, 120.5, 120.2, 119.5, 114.2, 109.6, 107.5, 61.1, 55.4, 17.7, 13.0. HRMS (m/z, ESI): Calcd. for Chemical Formula: $C_{29}H_{25}ClNO_3^+$ $[M+H]^+$: 470.1517, Found: 470.1513. A sample of the ester was hydrolyzed to provide the parent carboxylic acid HPLC analysis of the reaction product: Daicel Chiralpak ADH, hexane/*iso*-propanol = 95:5, 1 mL/min, λ= 315 nm, retention time: 17.76 min (major) and 33.42 min (minor).

Ethyl (*R*)-2-(2-chloro-7-(4-(diphenylamino)phenyl)-9*H*-carbazol-9-yl)-3-methylbenzoate (3)

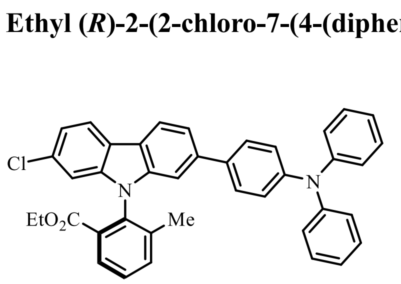

Yellow solid, 100 mg, 66%, $[\alpha]_D^{20}$ = +50.10 (*c* 1.000 $CHCl_3$) for 97.5:2.5 er. ^1H NMR (500 MHz, Chloroform-*d*) δ 8.11 (d, *J* = 8.1 Hz, 1H), 8.03 (d, *J* = 8.3 Hz, 1H), 7.96 (dd, *J* = 7.8, 1.6 Hz, 1H), 7.67 – 7.64 (m, 1H), 7.56 (t, *J* = 7.7 Hz, 1H), 7.51 – 7.43 (m, 3H), 7.23 (dd, *J* = 9.1, 7.1 Hz, 6H), 7.11 (d, *J* = 7.8 Hz, 5H), 7.02 (dd, *J* = 15.3, 7.9 Hz, 3H), 6.90 (d, *J* = 1.9 Hz, 1H), 3.68 (q,

J = 7.1 Hz, 2H), 2.03 (s, 3H), 0.44 (t, J = 7.1 Hz, 3H).^{13}C NMR (126 MHz, Chloroform-*d*) δ 166.0, 147.7, 142.3, 142.2, 139.4, 135.1, 134.1, 132.0, 131.6, 129.7, 129.3, 129.2, 128.1, 124.3, 124.0, 122.9, 121.6, 121.5, 121.1, 120.5, 120.2, 119.4, 109.6, 107.4, 61.1, 17.7, 13.0. HRMS (m/z, ESI): Calcd. for Chemical Formula: $C_{40}H_{32}ClN_2O_2^+$ [M+H]$^+$: 607.2147, Found: 607.2150. HPLC analysis of the reaction product: Daicel Chiralpak ID and ID, hexane/*iso*-propanol = 99:1, 0.8 mL/min, λ= 348 nm, retention time: 17.20 min (major) and 18.77 min (minor).

Ethyl (*S*)-2-(2-chloro-7-(4-(diphenylamino)phenyl)-9*H*-carbazol-9-yl)-3-methylbenzoate (*ent*-3)

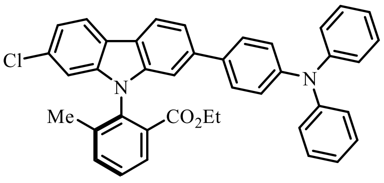

Yellow solid, 103 mg, 68%, $[\alpha]_D^{20}$ = −45.30 (*c* 0.920 $CHCl_3$) for 3:97 er. NMR was same with Ethyl (*R*)-2-(2-chloro-7-(4-(diphenylamino)phenyl)-9*H*-carbazol-9-yl)-3-methylbenzoate. HRMS (m/z, ESI): Calcd. for Chemical Formula: $C_{40}H_{32}ClN_2O_2^+$ [M+H]$^+$: 607.2147, Found: 607.2144. HPLC analysis of the reaction product: Daicel Chiralpak ID and ID, hexane/*iso*-propanol = 99:1, 0.8 mL/min, λ= 348 nm, retention time: 19.73 min (major) and 18.13 min (minor).

Ethyl (*S*)-2-(2-chloro-7-(4-(ethoxycarbonyl)phenyl)-9*H*-carbazol-9-yl)-3-methylbenzoate (4)

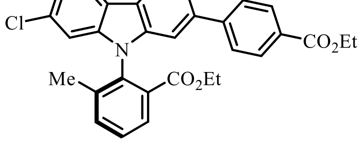

Yellowish oil, 86 mg, 67%, $[\alpha]_D^{20}$ = −54.55 (*c* 1.000 $CHCl_3$) for 5:95 er. ^{1}H NMR (500 MHz, Chloroform-*d*) δ 8.16 (dd, J = 8.1, 0.6 Hz, 1H), 8.10 – 8.04 (m, 3H), 7.99 (dd, J = 7.8, 1.7 Hz, 1H), 7.69 – 7.63 (m, 3H), 7.59 (t, J = 7.7 Hz, 1H), 7.55 (dd, J = 8.1, 1.5 Hz, 1H), 7.27 – 7.23 (m, 1H), 7.13 (dd, J = 1.6, 0.6 Hz, 1H), 6.94 (d, J = 1.8 Hz, 1H), 4.39 (q, J = 7.1 Hz, 2H), 3.71 (q, J = 7.1

Hz, 2H), 2.03 (s, 3H), 1.41 (t, J = 7.1 Hz, 3H), 0.48 (t, J = 7.1 Hz, 3H). ^{13}C NMR (126 MHz, Chloroform-*d*) δ 166.6, 165.9, 146.0, 142.4, 142.2, 139.3, 138.5, 135.2, 134.0, 132.1, 132.0, 130.0, 129.8, 129.3, 129.1, 127.3, 122.5, 121.4, 121.3, 120.7, 120.5, 119.8, 109.7, 108.1, 61.1, 61.0, 17.6, 14.4, 13.0. HRMS (m/z, ESI): Calcd. for Chemical Formula: $C_{31}H_{27}ClNO_4^+$ [M+H]$^+$: 512.1623, Found: 512.1621. HPLC analysis of the reaction product: Daicel Chiralpak IC, hexane/*iso*-propanol = 99:1, 1.0 mL/min, λ= 318 nm, retention time: 19.49 min (major) and 18.08 min (minor).

Ethyl (*S*)-2-(2-chloro-7-(4-cyanophenyl)-9*H*-carbazol-9-yl)-3-methylbenzoate (5)

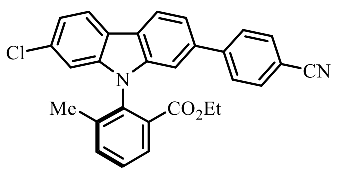

White solid, 64 mg, 55%, $[\alpha]_D^{20}$ = −46.20 (*c* 1.000 $CHCl_3$) for 4:96 er. ^{1}H NMR (500 MHz, Chloroform-*d*) δ 8.18 (dd, J = 8.1, 0.6 Hz, 1H), 8.07 (d, J = 8.2 Hz, 1H), 7.99 (dd, J = 7.8, 1.7 Hz, 1H), 7.70 – 7.66 (m, 5H), 7.60 (t, J = 7.7 Hz, 1H), 7.51 (dd, J = 8.1, 1.6 Hz, 1H), 7.26 (dd, J = 8.3, 1.8 Hz, 1H), 7.09 (dd, J = 1.7, 0.6 Hz, 1H), 6.94 (d, J = 1.9 Hz, 1H), 3.75 – 3.66 (m, 2H), 2.03 (s, 3H), 0.48 (t, J = 7.1 Hz, 3H). ^{13}C NMR (126 MHz, Chloroform-*d*) δ 165.9, 146.2, 142.5, 142.1, 139.3, 137.5, 135.2, 133.8, 132.5, 132.4, 131.9, 129.8, 129.4, 128.1, 122.9, 121.5, 121.2, 120.9, 120.6, 119.6, 119.0, 110.6, 109.8, 108.1, 61.2, 17.6, 13.0. HRMS (m/z, ESI): Calcd. for Chemical Formula: $C_{29}H_{22}ClN_2O_2^+$ [M+H]$^+$: 465.1364, Found: 465.1374. A sample of the Me-ester was provided for the HPLC analysis of the reaction product: Daicel Chiralpak IA, hexane/*iso*-propanol = 99:1, 1.0 mL/min, λ= 315 nm, retention time: 36.14 min (major) and 39.19 min (minor).

Ethyl (*R*)-2-(2-chloro-7-(3-nitrophenyl)-9*H*-carbazol-9-yl)-3-methylbenzoate (6)

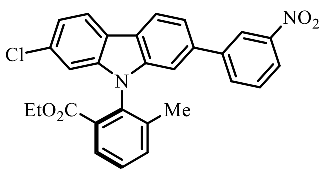

Yellowish solid, 70 mg, 58%, $[\alpha]_D^{20}$ = +30.70 (*c* 1.000 $CHCl_3$) for 99:1 er. ^{1}H NMR (500 MHz, Chloroform-*d*) δ 8.44 (t, *J* = 2.0 Hz, 1H), 8.20 (dd, *J* = 8.1, 0.6 Hz, 1H), 8.17 – 8.15 (m, 1H), 8.07 (d, *J* = 8.3 Hz, 1H), 8.00 (dd, *J* = 7.7, 1.6 Hz, 1H), 7.91 – 7.89 (m, 1H), 7.71 – 7.68 (m, 1H), 7.63 – 7.52 (m, 3H), 7.26 (dd, *J* = 8.2, 1.9 Hz, 1H), 7.11 (dd, *J* = 1.7, 0.6 Hz, 1H), 6.94 (d, *J* = 1.8 Hz, 1H), 3.71 (qd, *J* = 7.1, 2.1 Hz, 2H), 2.04 (s, 3H), 0.50 (t, *J* = 7.1 Hz, 3H). ^{13}C NMR (126 MHz, Chloroform-*d*) δ 165.8, 148.7, 143.4, 142.5, 142.2, 139.3, 137.1, 135.3, 133.8, 133.5, 132.4, 131.9, 129.9, 129.6, 129.5, 122.8, 122.2, 121.9, 121.4, 121.2, 121.0, 120.6, 119.6, 109.8, 108.1, 61.1, 17.6, 13.0. HRMS (m/z, ESI): Calcd. for Chemical Formula: $C_{28}H_{22}ClN_2O_4^+$ $[M+H]^+$: 485.1263, Found: 485.1257. HPLC analysis of the reaction product: Daicel Chiralpak IB, hexane/*iso*-propanol = 99:1, 1.0 mL/min, λ= 311 nm, retention time: 19.66 min (major) and 16.90 min (minor).

Ethyl (*R*)-2-(2-chloro-7-(3-(ethoxycarbonyl)phenyl)-9*H*-carbazol-9-yl)-3-methylbenzoate (7)

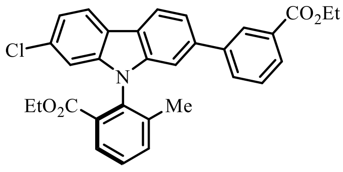

Yellowish oil, 88 mg, 69%, $[\alpha]_D^{20}$ = −81.00 (*c* 1.000 $CHCl_3$) for 97:3 er. ^{1}H NMR (500 MHz, Chloroform-*d*) δ 8.28 (t, *J* = 1.8 Hz, 1H), 8.17 (d, *J* = 8.1 Hz, 1H), 8.06 (d, *J* = 8.3 Hz, 1H), 8.00 – 7.98 (m, 2H), 7.78 – 7.75 (m, 1H), 7.67 (dd, *J* = 8.2, 1.6 Hz, 1H), 7.58 (t, *J* = 7.7 Hz, 1H), 7.55 (dd, *J* = 8.1, 1.6 Hz, 1H), 7.47 (t, *J* = 7.7 Hz, 1H), 7.25 (dd, *J* = 8.3, 1.8 Hz, 1H), 7.12 (d, *J* = 1.6 Hz, 1H), 6.92 (d, *J* = 1.8 Hz, 1H), 4.40 (q, *J* = 7.1 Hz, 2H), 3.71 (q, *J* = 7.1 Hz, 2H), 2.03 (s, 3H), 1.41 (t, *J* = 7.2 Hz, 3H), 0.48 (t, *J* = 7.1 Hz, 3H). ^{13}C NMR (126 MHz, Chloroform-*d*) δ 166.7, 165.9, 142.4, 142.2, 142.0, 139.3, 138.8, 135.2, 134.0, 131.9, 131.0, 129.8, 129.3, 128.7, 128.6, 128.1, 122.2, 121.4, 121.2, 120.7, 120.4, 119.8, 109.7, 108.1, 61.1, 17.6, 14.4, 13.0. HRMS (m/z, ESI): Calcd. for

Chemical Formula: $C_{31}H_{27}ClNO_4^+$ [M+H]$^+$: 512.1623, Found: 512.1625. HPLC analysis of the reaction product: Daicel Chiralpak IC, hexane/*iso*-propanol = 99:1, 1.0 mL/min, λ= 311 nm, retention time: 21.43 min (major) and 19.97 min (minor).

Ethyl (*S*)-2-(2-(3-(benzyloxy)phenyl)-7-chloro-9*H*-carbazol-9-yl)-3-methylbenzoate (8)

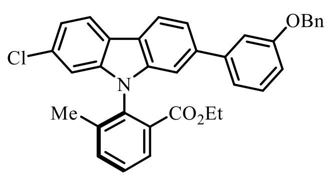

Yellowish oil, 82 mg, 60%, $[\alpha]_D^{20}$ = −63.08 (*c* 1.000 $CHCl_3$) for 3.5:96.5 er. ^{1}H NMR (500 MHz, Chloroform-*d*) δ 8.14 (dd, *J* = 8.1, 0.6 Hz, 1H), 8.06 (d, *J* = 8.3 Hz, 1H), 8.00 (dd, *J* = 7.8, 1.7 Hz, 1H), 7.68 – 7.66 (m, 1H), 7.59 (t, *J* = 7.7 Hz, 1H), 7.52 (dd, *J* = 8.1, 1.5 Hz, 1H), 7.48 – 7.44 (m, 2H), 7.42 – 7.37 (m, 2H), 7.35 – 7.32(m, 2H), 7.25 (dd, *J* = 8.3, 1.9 Hz, 1H), 7.24 – 7.20 (m, 2H), 7.09 (dd, *J* = 1.6, 0.6 Hz, 1H), 6.97 – 6.92 (m, 2H), 5.12 (s, 2H), 3.71 (q, *J* = 7.1 Hz, 2H), 2.03 (s, 3H), 0.47 (t, *J* = 7.1 Hz, 3H). ^{13}C NMR (126 MHz, Chloroform-*d*) δ 165.9, 159.1, 143.3, 142.3, 139.5, 139.4, 136.9, 135.1, 134.1, 131.9, 131.8, 129.8, 129.7, 129.2, 128.6, 128.4, 128.0, 127.6, 121.9, 121.5, 121.2, 120.5, 120.3_1, 120.2_7, 119.7, 114.6, 113.0, 109.6, 108.1, 70.1, 61.1, 17.6, 13.0. HRMS (m/z, ESI): Calcd. for Chemical Formula: $C_{35}H_{29}ClNO_3^+$ [M+H]$^+$: 546.1830, Found: 546.1829. HPLC analysis of the reaction product: Daicel Chiralpak IF, hexane/*iso*-propanol = 99:1, 1.0 mL/min, λ= 312 nm, retention time: 12.31 min (major) and 11.07 min (minor).

Ethyl (*R*)2-(2-chloro-7-(3-methoxyphenyl)-9*H*-carbazol-9-yl)-3-methylbenzoate (9)

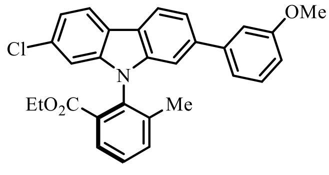

1 mmol scale, White solid, 338 mg, 72%, $[\alpha]_D^{20}$ = +29.10 (*c* 1.000 $CHCl_3$) for 99:1 er. ^{1}H NMR (500 MHz, Chloroform-*d*) δ 8.13 (d, *J* = 8.1 Hz, 1H), 8.04 (d, *J* = 8.3 Hz, 1H), 7.98 (dd, *J* = 7.8, 1.7 Hz,

1H), 7.68 – 7.64 (m, 1H), 7.57 (t, *J* = 7.7 Hz, 1H), 7.51 (dd, *J* = 8.1, 1.5 Hz, 1H), 7.33 (t, *J* = 7.9 Hz, 1H), 7.23 (dd, *J* = 8.2, 1.8 Hz, 1H), 7.19 – 7.17 (m, 1H), 7.12 (t, *J* = 2.2 Hz, 1H), 7.08 (d, *J* = 1.6 Hz, 1H), 6.92 (d, *J* = 1.8 Hz, 1H), 6.88 – 6.85 (m, 1H), 3.85 (s, 3H), 3.70 (q, *J* = 7.1 Hz, 2H), 2.02 (s, 3H), 0.46 (t, *J* = 7.1 Hz, 3H). ^{13}C NMR (126 MHz, Chloroform-*d*) δ 165.9, 159.9, 143.3, 142.3, 142.2, 139.6, 139.4, 135.1, 134.1, 132.0, 131.8, 129.8, 129.7, 129.2, 121.9, 121.5, 121.2, 120.5, 120.3, 120.1, 119.8, 113.6, 112.1, 109.6, 108.1, 61.1, 55.4, 17.6, 13.0. HRMS (m/z, ESI): Calcd. for Chemical Formula: $C_{29}H_{25}ClNO_3^+$ [M+H]$^+$: 470.1517, Found: 470.1515. HPLC analysis of the reaction product: Daicel Chiralpak ID and ID, hexane/*iso*-propanol = 99:1, 0.8 mL/min, λ= 330 nm, retention time: 19.39 min (major) and 21.97 min (minor).

Ethyl (*R*)-2-(2-([1,1'-biphenyl]-2-yl)-7-chloro-9*H*-carbazol-9-yl)-3-methylbenzoate (10)

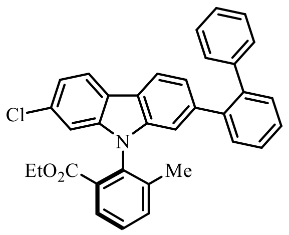

White solid, 94 mg, 73%, $[\alpha]_D^{20}$ = −43.30 (*c* 1.000 $CHCl_3$) for 98.5:1.5 er. ^{1}H NMR (500 MHz, Chloroform-*d*) δ 7.99 – 7.90 (m, 3H), 7.56 (dd, *J* = 7.8, 1.8 Hz, 1H), 7.51 (t, *J* = 7.7 Hz, 1H), 7.48 – 7.44 (m, 1H), 7.42 – 7.37 (m, 3H), 7.21 – 7.19 (m, 1H), 7.16 – 7.06 (m, 6H), 6.87 (t, *J* = 1.7 Hz, 1H), 6.62 (t, 1.5 Hz, 1H), 3.72 – 3.62 (m, 2H), 1.78 (s, 3H), 0.45 – 0.42 (m, 3H). ^{13}C NMR (126 MHz, Chloroform-*d*) δ 165.8, 142.2, 141.8, 141.4, 141.0, 140.8, 139.9, 139.4, 134.9, 134.1, 131.6, 130.9, 130.6, 129.8, 129.7, 129.0, 127.8, 127.41, 127.36, 126.4, 122.2, 121.6, 121.1, 121.0, 120.0, 119.7, 111.3, 109.5, 61.0, 17.5, 13.0. HRMS (m/z, ESI): Calcd. for Chemical Formula: $C_{34}H_{27}ClNO_2^+$ [M+H]$^+$: 516.1725, Found: 516.1727. A sample of the ester was hydrolyzed to provide the parent carboxylic acid for the HPLC analysis of the reaction product: Daicel Chiralpak ID, hexane/*iso*-propanol = 97:3, 1.0 mL/min, λ= 309 nm, retention time: 7.07 min (major) and 11.44

min (minor).

Ethyl (*S*)-2-(2-chloro-7-(6-methoxypyridin-3-yl)-9*H*-carbazol-9-yl)-3-methylbenzoate (11)

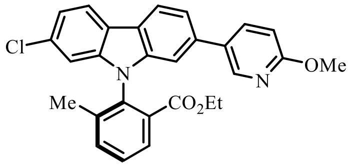

White solid, 73 mg, 62%, $[\alpha]_D^{20}$ = −73.00 (*c* 1.000 $CHCl_3$) for 1.5:98.5 er. ^{1}H NMR (500 MHz, Chloroform-*d*) δ 8.39 (d, *J* = 2.4 Hz, 1H), 8.14 (d, *J* = 8.1 Hz, 1H), 8.04 (d, *J* = 8.3 Hz, 1H), 7.97 (dd, *J* = 7.8, 1.6 Hz, 1H), 7.79 (dd, *J* = 8.6, 2.5 Hz, 1H), 7.67 – 7.65 (m, 1H), 7.57 (t, *J* = 7.7 Hz, 1H), 7.44 (dd, *J* = 8.1, 1.5 Hz, 1H), 7.24 (dd, *J* = 8.3, 1.8 Hz, 1H), 7.02 (d, *J* = 1.5 Hz, 1H), 6.93 (d, *J* = 1.8 Hz, 1H), 6.79 (d, *J* = 8.6 Hz, 1H), 3.96 (s, 3H), 3.70 (q, *J* = 7.1 Hz, 2H), 2.02 (s, 3H), 0.48 (t, *J* = 7.1 Hz, 3H). ^{13}C NMR (126 MHz, Chloroform-*d*) δ 165.9, 163.5, 145.2, 142.3, 139.3, 137.8, 136.3, 135.1, 134.0, 131.9_4, 131.9_0, 130.6, 129.7, 129.3, 121.8, 121.5, 121.2, 120.8, 120.4, 119.2, 110.7, 109.7, 107.5, 61.1, 53.6, 17.6, 13.0. A sample of the Me-ester was provided for the HRMS (m/z, ESI): Calcd. for Chemical Formula: $C_{28}H_{24}ClN_2O_3^+$ $[M+H]^+$: 471.1470, Found: 471.1476. HPLC analysis of the reaction product: Daicel Chiralpak IA, hexane/*iso*-propanol = 99:1, 1.0 mL/min, λ= 307 nm, retention time: 14.93 min (major) and 13.56 min (minor).

Ethyl (*R*)-2-(2-chloro-7-(furan-3-yl)-9*H*-carbazol-9-yl)-3-methylbenzoate (12)

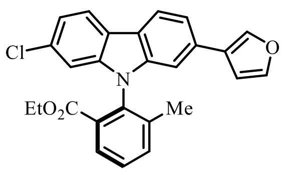

White solid, 70 mg, 65%, $[\alpha]_D^{20}$ = +12.60 (*c* 1.000 $CHCl_3$) for 97.5:2.5 er. ^{1}H NMR (500 MHz, Chloroform-*d*) δ 8.07 (dd, *J* = 8.1, 0.6 Hz, 1H), 8.03 – 7.97 (m, 2H), 7.72 (dd, *J* = 1.5, 0.9 Hz, 1H), 7.68 – 7.66 (m, 1H), 7.58 (t, *J* = 7.7 Hz, 1H), 7.44 (t, *J* = 1.7 Hz, 1H), 7.42 (dd, *J* = 8.1, 1.5 Hz, 1H), 7.22 (dd, *J* = 8.3, 1.8 Hz, 1H), 6.97 (dd, *J* = 1.6, 0.7 Hz, 1H), 6.89 (d, *J* = 1.8 Hz, 1H), 6.68 (dd, *J* = 1.9, 0.9 Hz, 1H),

3.70 (qd, J = 7.2, 1.9 Hz, 2H), 2.01 (s, 3H), 0.46 (t, J = 7.1 Hz, 3H). ^{13}C NMR (126 MHz, Chloroform-d) δ 165.9, 143.6, 142.2, 139.4, 138.7, 135.1, 134.1, 131.9, 131.6, 130.8, 129.8, 129.2, 127.0, 121.6, 121.0, 120.6, 120.3, 118.6, 109.6, 109.2, 106.5, 61.1, 17.6, 13.0. HRMS (m/z, ESI): Calcd. for Chemical Formula: $C_{26}H_{21}ClNO_3^+$ $[M+H]^+$: 430.1204, Found: 430.1200. HPLC analysis of the reaction product: Daicel Chiralpak IF, hexane/*iso*-propanol = 99:1, 1.0 mL/min, λ= 313 nm, retention time: 7.89 min (major) and 7.36 min (minor).

Ethyl (*S*)-2-(2-(5-acetylthiophen-2-yl)-7-chloro-9*H*-carbazol-9-yl)-3-methylbenzoate (13)

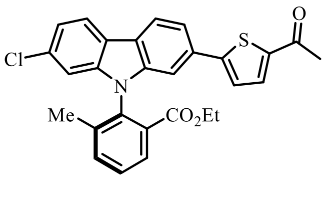

White solid, 40 mg, 33%, $[\alpha]_D^{20}$ = −11.50 (c 1.000 $CHCl_3$) for 7:93 er. ^{1}H NMR (500 MHz, Chloroform-d) δ 8.11 (dd, J = 8.1, 0.6 Hz, 1H), 8.03 (d, J = 8.3 Hz, 1H), 8.00 (dd, J = 7.7, 1.6 Hz, 1H), 7.71 – 7.67 (m, 1H), 7.64 – 7.60 (m, 2H), 7.58 (dd, J = 8.2, 1.5 Hz, 1H), 7.30 (d, J = 4.0 Hz, 1H), 7.24 (dd, J = 8.3, 1.8 Hz, 1H), 7.15 (dd, J = 1.6, 0.6 Hz, 1H), 6.91 (d, J = 1.8 Hz, 1H), 3.72 (qd, J = 7.1, 0.8 Hz, 2H), 2.55 (s, 3H), 2.01 (s, 3H), 0.50 (t, J = 7.1 Hz, 3H). ^{13}C NMR (126 MHz, Chloroform-d) δ 190.6, 165.7, 153.6, 142.8, 142.6, 141.9, 139.3, 135.3, 133.7, 133.5, 132.5, 131.7, 131.5, 129.9, 129.5, 124.0, 123.3, 121.4, 121.3, 120.9, 120.7, 118.8, 109.7, 107.2, 61.1, 26.5, 17.6, 13.1. HRMS (m/z, ESI): Calcd. for Chemical Formula: $C_{28}H_{23}ClNO_3S^+$ $[M+H]^+$: 488.1082, Found: 488.1081. HPLC analysis of the reaction product: Daicel Chiralpak IA, hexane/*iso*-propanol = 99:1, 1.0 mL/min, λ= 355 nm, retention time: 27.21 min (major) and 29.73 min (minor).

Ethyl (*S*)-2-(2-chloro-7-(thiophen-3-yl)-9*H*-carbazol-9-yl)-3-methylbenzoate (14)

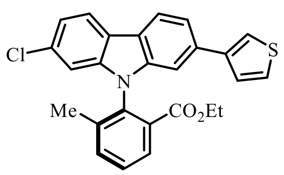

White solid, 78 mg, 70%, $[\alpha]_D^{20} = -71.50$ (*c* 1.000 $CHCl_3$) for 3.5:96.5 er. ^{1}H NMR (500 MHz, Chloroform-*d*) δ 8.10 (dd, *J* = 8.1, 0.7 Hz, 1H), 8.03 (d, *J* = 8.3 Hz, 1H), 7.99 (dd, *J* = 7.8, 1.6 Hz, 1H), 7.68 – 7.66 (m, 1H), 7.58 (t, *J* = 7.7 Hz, 1H), 7.54 (dd, *J* = 8.1, 1.5 Hz, 1H), 7.43 (dd, *J* = 2.9, 1.5 Hz, 1H), 7.38 (dd, *J* = 5.0, 1.4 Hz, 1H), 7.35 (dd, *J* = 5.0, 2.9 Hz, 1H), 7.24 (dd, *J* = 8.3, 1.8 Hz, 1H), 7.10 (dd, *J* = 1.6, 0.6 Hz, 1H), 6.92 (d, *J* = 1.8 Hz, 1H), 3.70 (qd, *J* = 7.1, 1.1 Hz, 2H), 2.02 (s, 3H), 0.47 (t, *J* = 7.1 Hz, 3H). ^{13}C NMR (126 MHz, Chloroform-*d*) δ 166.0, 142.9, 142.3, 142.2, 139.4, 135.1, 134.4, 134.1, 132.0, 131.7, 129.8, 129.2, 126.7, 126.1, 121.7, 121.6, 121.1, 120.6, 120.4, 120.3, 119.2, 109.6, 107.2, 61.1, 17.6, 13.0. A sample of the Me-ester was provided for the HRMS (m/z, ESI): Calcd. for Chemical Formula: $C_{26}H_{21}ClNO_2S^+$ $[M+H]^+$: 446.0976, Found: 446.0971. HPLC analysis of the reaction product: Daicel Chiralpak IA, hexane/iso-propanol = 99: 1, 1.0 mL/min, λ = 314 nm, retention time: 9.85 min (major) and 11.06 min (minor).

Ethyl (*R*)-2-(2-chloro-7-(4,6-dihydropyren-1-yl)-9*H*-carbazol-9-yl)-3-methylbenzoate (15)

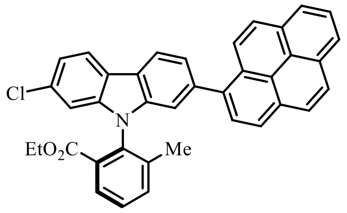

White solid, 100 mg, 71%, $[\alpha]_D^{20} = +24.65$ (*c* 1.000 $CHCl_3$) for 94:6 er. ^{1}H NMR (500 MHz, Chloroform-*d*) δ 8.27 (d, *J* = 7.9 Hz, 1H), 8.23 – 8.18 (m, 3H), 8.17 – 8.13 (m, 2H), 8.08 (s, 2H), 8.01 (dd, *J* = 8.2, 7.2 Hz, 3H), 7.95 (dd, *J* = 7.8, 1.6 Hz, 1H), 7.62 – 7.59 (m, 1H), 7.58 (dd, *J* = 7.9, 1.5 Hz, 1H), 7.49 (t, *J* = 7.7 Hz, 1H), 7.31 (dd, *J* = 8.3, 1.9 Hz, 1H), 7.19 (d, *J* = 1.4 Hz, 1H), 7.00 (d, *J* = 1.8 Hz, 1H), 3.87 – 3.75 (m, 2H), 2.11 (s, 3H), 0.60 (t, *J* = 7.2 Hz, 3H). ^{13}C NMR (126 MHz, Chloroform-*d*) δ 166.0, 142.3, 142.0, 139.6, 139.3, 138.2, 135.1, 134.1, 131.9_4, 131.9_1, 131.5, 131.0, 130.6, 129.8, 129.2, 128.6, 127.9, 127.5, 127.4, 127.4, 126.0, 125.4, 125.1, 125.0, 124.9, 124.8,

124.5, 123.1, 121.7$_3$, 121.6$_9$, 121.3, 120.4, 120.0, 111.6, 109.7, 61.2, 17.7, 13.2.HRMS (m/z, ESI): Calcd. for Chemical Formula: $C_{38}H_{27}ClNO_2^+$ $[M+H]^+$: 564.1725, Found: 564.1728. HPLC analysis of the reaction product: Daicel Chiralpak ODH, hexane/*iso*-propanol = 99:1, 1.0 mL/min, λ= 345 nm, retention time: 12.30 min (major) and 14.02 min (minor).

Ethyl (*R*)-2-(2-chloro-7-(9,9-dimethyl-9*H*-fluoren-1-yl)-9*H*-carbazol-9-yl)-3-methylbenzoate (16)

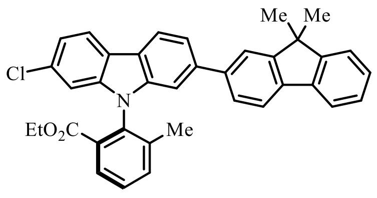

White solid, 95 mg, 68%, $[\alpha]_D^{20}$ = +78.10 (*c* 1.000 $CHCl_3$) for 96.5:3.5 er. ^1H NMR (500 MHz, Chloroform-*d*) δ 8.18 (d, *J* = 8.0 Hz, 1H), 8.08 (d, *J* = 8.2 Hz, 1H), 8.02 (dd, *J* = 7.8, 1.7 Hz, 1H), 7.78 – 7.73 (m, 2H), 7.70 – 7.65 (m, 2H), 7.63 – 7.55 (m, 3H), 7.48 – 7.44 (m, 1H), 7.38 – 7.32 (m, 2H), 7.27 (dd, *J* = 8.1, 2.0 Hz, 1H), 7.16 (d, *J* = 1.5 Hz, 1H), 6.95 (d, *J* = 1.8 Hz, 1H), 3.74 (q, *J* = 7.1 Hz, 2H), 2.06 (s, 3H), 1.55 (d, *J* = 4.8 Hz, 6H), 0.51 (t, *J* = 7.1 Hz, 3H). ^{13}C NMR (126 MHz, Chloroform-*d*) δ 166.0, 154.2, 153.9, 142.3$_4$, 142.2$_9$, 141.0, 140.4, 139.5, 138.9, 138.4, 135.1, 134.2, 132.0, 131.8, 129.8, 129.2, 127.3, 127.0, 126.7, 122.6, 121.7, 121.6, 121.2, 120.5, 120.3, 120.2, 120.1, 120.0, 109.7, 108.0, 61.2, 47.0, 27.3, 17.7, 13.0. HRMS (m/z, ESI): Calcd. for Chemical Formula: $C_{37}H_{31}ClNO_2^+$ $[M+H]^+$: 556.2038, Found: 556.2039. HPLC analysis of the reaction product: Daicel Chiralpak ADH and ADH, hexane/*iso*-propanol = 99:1, 1.0 mL/min, λ= 326 nm, retention time: 19.13 min (major) and 16.70 min (minor).

Ethyl (*R*)-2-(2-chloro-7-(naphthalen-2-yl)-9*H*-carbazol-9-yl)-3-methylbenzoate (17)

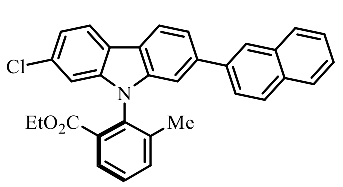

White solid, 90 mg, 74%, $[\alpha]_D^{20}$ = +26.20 (*c* 1.000 $CHCl_3$) for 95.5:4.5 er. ^{1}H NMR (500 MHz, Chloroform-*d*) δ 8.19 (d, *J* = 8.1 Hz, 1H), 8.08 (d, *J* = 8.3 Hz, 1H), 8.05 (d, *J* = 1.9 Hz, 1H), 8.01 (dd, *J* = 7.7, 1.7 Hz, 1H), 7.89 (dd, *J* = 9.2, 2.1 Hz, 2H), 7.85 (dd, *J* = 7.3, 1.8 Hz, 1H), 7.75 (dd, *J* = 8.5, 1.9 Hz, 1H), 7.69 – 7.66 (m, 2H), 7.60 (t, *J* = 7.7 Hz, 1H), 7.52 – 7.45 (m, 2H), 7.26 (dd, *J* = 8.2, 1.9 Hz, 1H), 7.23 (d, *J* = 1.5 Hz, 1H), 6.95 (d, *J* = 1.8 Hz, 1H), 3.73 (q, *J* = 7.1 Hz, 2H), 2.06 (s, 3H), 0.50 (t, *J* = 7.1 Hz, 3H). ^{13}C NMR (126 MHz, Chloroform-*d*) δ 166.0, 142.4, 142.3, 139.7, 139.4, 139.0, 135.1, 134.1, 133.7, 132.6, 132.0, 131.8, 129.8, 129.3, 128.4, 128.2, 127.7, 126.3, 126.0_3, 126.0_0, 125.9, 121.9, 121.6, 121.2, 120.6, 120.3, 120.1, 109.7, 108.3, 61.2, 17.7, 13.0. HRMS (m/z, ESI): Calcd. for Chemical Formula: $C_{32}H_{25}ClNO_2^+$ $[M+H]^+$: 490.1568, Found: 490.1575. A sample of the Me-ester was provided for the HPLC analysis of the reaction product: Daicel Chiralpak ADH, hexane/*iso*-propanol = 99:1, 1.0 mL/min, λ= 318 nm, retention time: 24.83 min (major) and 18.73 min (minor).

Ethyl (*R*)-2-(2-chloro-7-(dibenzo[*b,d*]thiophen-4-yl)-9*H*-carbazol-9-yl)-3-methylbenzoate (18)

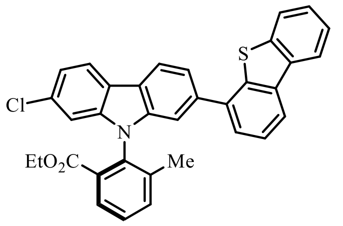

White solid, 95 mg, 70%, $[\alpha]_D^{20}$ = +26.90 (*c* 1.000 $CHCl_3$) for 97.5:2.5 er. ^{1}H NMR (500 MHz, Chloroform-*d*) δ 8.26 (dd, *J* = 8.1, 0.7 Hz, 1H), 8.21 – 8.18 (m, 1H), 8.15 (dd, *J* = 7.5, 1.5 Hz, 1H), 8.13 (d, *J* = 8.3 Hz, 1H), 8.01 (dd, *J* = 7.8, 1.6 Hz, 1H), 7.86 – 7.83 (m, 1H), 7.71 – 7.66 (m, 2H), 7.59 – 7.51 (m, 3H), 7.50 – 7.46 (m, 2H), 7.32 – 7.29 (m, 2H), 6.98 (d, *J* = 1.7 Hz, 1H), 3.85 – 3.73 (m, 2H), 2.11 (s, 3H), 0.56 (t, *J* = 7.1 Hz, 3H). ^{13}C NMR (126 MHz, Chloroform-*d*) δ 165.9, 142.4, 142.1, 139.6, 139.5, 139.0, 137.6, 136.2, 135.8, 135.1, 134.1, 132.0, 131.8, 129.9, 129.3, 127.2,

126.8, 125.1, 124.4, 122.6, 122.3, 121.7, 121.6, 121.3, 120.5_9, 120.5_7, 120.4, 120.3, 109.7, 109.4, 61.2, 17.7, 13.1. HRMS (m/z, ESI): Calcd. for Chemical Formula: $C_{34}H_{25}ClNO_2S^+$ $[M+H]^+$: 546.1289, Found: 546.1291. HPLC analysis of the reaction product: Daicel Chiralpak IB, hexane/*iso*-propanol = 99:1, 1.0 mL/min, λ= 310 nm, retention time: 23.44 min (major) and 17.47 min (minor).

Methyl (*S*)-2-(2-chloro-7-(dibenzo[*b*,*d*]thiophen-3-yl)-9*H*-carbazol-9-yl)-3-methylbenzoate (19)

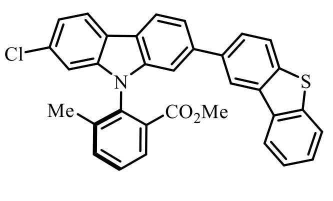

White soild, 84 mg, 62%, $[\alpha]_D^{20}$ = −71.60 (*c* 1.000 $CHCl_3$) for 4.5:95.5 er. ^1H NMR (500 MHz, Chloroform-*d*) δ 8.37 – 8.34 (m, 1H), 8.24 – 8.20 (m, 2H), 8.09 (d, *J* = 8.2 Hz, 1H), 8.01 (dd, *J* = 7.8, 1.6 Hz, 1H), 7.90 – 7.85 (m, 2H), 7.71 – 7.68 (m, 2H), 7.65 (dd, *J* = 8.1, 1.5 Hz, 1H), 7.59 (t, *J* = 7.7 Hz, 1H), 7.49 – 7.45 (m, 2H), 7.28 (dd, *J* = 8.3, 1.8 Hz, 1H), 7.20 (dd, *J* = 1.5, 0.6 Hz, 1H), 6.95 (d, *J* = 1.8 Hz, 1H), 3.32 (s, 3H), 2.06 (s, 3H). ^{13}C NMR (126 MHz, Chloroform-*d*) δ 166.0, 142.3, 142.3, 139.9, 139.8, 139.5, 138.5, 138.4, 136.1, 135.5, 135.4, 134.5, 131.9, 131.3, 129.7, 129.2, 126.9, 126.6, 124.4, 123.0, 122.9, 121.9, 121.7, 121.6, 121.3, 120.8, 120.41, 120.38, 120.1, 109.6, 108.1, 52.2, 17.7. HRMS (m/z, ESI): Calcd. for Chemical Formula: $C_{33}H_{23}ClNO_2S^+$ $[M+H]^+$: 532.1133, Found: 532.1129. HPLC analysis of the reaction product: Daicel Chiralpak IA, hexane/*iso*-propanol = 99:1, 1.0 mL/min, λ= 330nm, retention time: 20.09 min (major) and 25.61 min (minor).

Ethyl (*R*)-2-(7-chloro-9'-phenyl-9*H*,9'*H*-[2,2'-bicarbazol]-9-yl)-3-methylbenzoate (20)

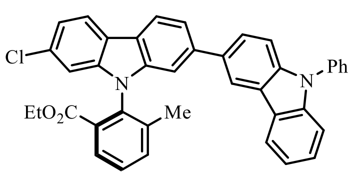

Yellowish solid, 106 mg, 70%, $[\alpha]_D^{20}$ = +45.4 (*c* 1.000 $CHCl_3$) for 94.5:5.5 er. ^{1}H NMR (500 MHz, Chloroform-*d*) δ 8.35 (d, *J* = 1.7 Hz, 1H), 8.23 – 8.16 (m, 2H), 8.07 (d, *J* = 8.3 Hz, 1H), 8.01 (dd, *J* = 7.9, 1.6 Hz, 1H), 7.70 – 7.64 (m, 3H), 7.64 – 7.57 (m, 5H), 7.51 – 7.46 (m, 1H), 7.46 – 7.42 (m, 3H), 7.32 – 7.28 (m, 1H), 7.28 – 7.23 (m, 1H), 7.21 (d, *J* = 1.6 Hz, 1H), 6.95 (d, *J* = 1.8 Hz, 1H), 3.74 (q, *J* = 7.1 Hz, 2H), 2.07 (s, 3H), 0.51 (t, *J* = 7.1 Hz, 3H). ^{13}C NMR (126 MHz, Chloroform-*d*) δ 166.1, 142.4, 142.3, 141.4, 140.7, 140.3, 139.5, 137.7, 135.1, 134.3, 134.0, 132.0, 131.5, 129.9, 129.8, 129.2, 127.5, 127.1, 126.1, 125.9, 123.9, 123.5, 121.7, 121.3, 121.1, 120.5, 120.5, 120.2, 120.1_4, 120.0_5, 119.1, 110.0, 109.9, 109.6, 108.1, 61.2, 17.7, 13.0. HRMS (m/z, ESI): Calcd. for Chemical Formula: $C_{40}H_{30}ClN_2O_2^+$ $[M+H]^+$: 605.1990, Found: 605.1989. HPLC analysis of the reaction product: Daicel Chiralpak ODH, hexane/*iso*-propanol = 99:1, 1.0 mL/min, λ= 364 nm, retention time: 15.85 min (major) and 20.96 min (minor).

Ethyl (*R*)-2-(2-chloro-7-(4-methoxyphenyl)-9*H*-carbazol-9-yl)-3'-methoxy-[1,1'-biphenyl]-3-carboxylate (22)

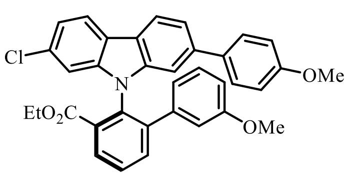

White solid, 95 mg, 68%, $[\alpha]_D^{20}$ = −12.50 (*c* 1.000 $CHCl_3$) for 89.5:10.5 er. ^{1}H NMR (500 MHz, Chloroform-*d*) δ 8.10 (dd, *J* = 7.7, 1.7 Hz, 1H), 7.97 (dd, *J* = 8.1, 0.6 Hz, 1H), 7.88 (d, *J* = 8.3 Hz, 1H), 7.82 (dd, *J* = 7.7, 1.7 Hz, 1H), 7.72 (t, *J* = 7.7 Hz, 1H), 7.52 – 7.46 (m, 2H), 7.38 (dd, *J* = 8.2, 1.6 Hz, 1H), 7.13 – 7.10 (m, 2H), 6.99 – 6.92 (m, 4H), 6.73 – 6.70 (m, 1H), 6.57 – 6.54 (m, 1H), 6.52 (dd, *J* = 2.5, 1.6 Hz, 1H), 3.84 (s, 3H), 3.71 (q, *J* = 7.1 Hz, 2H), 3.21 (s, 3H), 0.47 (t, *J* = 7.1 Hz, 3H). ^{13}C NMR (126 MHz, Chloroform-*d*) δ 166.1, 159.1, 159.0, 143.3, 142.8, 142.5, 139.3,

139.0, 134.9, 134.3, 132.9, 132.6, 131.3, 131.0, 129.3, 129.2, 128.4, 121.5, 121.2, 120.8, 120.4, 120.2, 120.1, 119.4, 114.7, 114.2, 112.2, 109.8, 107.8, 61.3, 55.4, 54.7, 13.0. HRMS (m/z, ESI): Calcd. for Chemical Formula: $C_{35}H_{29}ClNO_4^+$ $[M+H]^+$: 562.1780, Found: 562.1783. HPLC analysis of the reaction product: Daicel Chiralpak IF, hexane/*iso*-propanol = 99:1, 1.0 mL/min, λ= 316 nm, retention time: 14.42 min (major) and 13.34 min (minor).

Ethyl (*R*)-2-(2-chloro-7-(4-methoxyphenyl)-9*H*-carbazol-9-yl)-3'-nitro-[1,1'-biphenyl]-3-carboxylate (23)

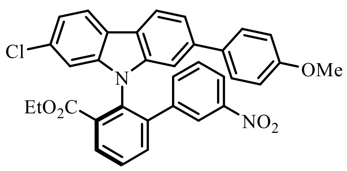

White solid, 100 mg, 69%, $[\alpha]_D^{20}$ = −25.10 (*c* 1.000 $CHCl_3$) for 90.5:9.5 er. ^{1}H NMR (500 MHz, Chloroform-*d*) δ 8.18 (dd, *J* = 7.7, 1.7 Hz, 1H), 8.09 (t, *J* = 2.0 Hz, 1H), 7.96 (d, *J* = 8.1 Hz, 1H), 7.88 (dd, *J* = 9.0, 2.2 Hz, 2H), 7.84 (dd, *J* = 7.8, 1.8 Hz, 1H), 7.78 (t, *J* = 7.7 Hz, 1H), 7.48 – 7.44 (m, 2H), 7.38 (dd, *J* = 8.2, 1.5 Hz, 1H), 7.25 – 7.23 (m, 1H), 7.14 (dd, *J* = 8.2, 1.8 Hz, 1H), 7.11 – 7.05 (m, 2H), 6.98 – 6.92 (m, 3H), 3.84 (s, 3H), 3.72 (q, *J* = 7.1 Hz, 2H), 0.49 (t, *J* = 7.1 Hz, 3H). ^{13}C NMR (126 MHz, Chloroform-*d*) δ 165.7, 159.2, 147.9, 142.4, 142.3, 140.8, 139.7, 139.4, 134.6, 134.1, 133.7, 133.2, 132.8, 132.2, 131.6, 129.7, 129.1, 128.4, 123.2, 122.7, 121.7, 121.2, 121.0, 120.6, 120.5, 119.9, 114.3, 109.5, 107.5, 61.5, 55.4, 13.0. HRMS (m/z, ESI): Calcd. for Chemical Formula: $C_{34}H_{26}ClN_2O_5^+$ $[M+H]^+$: 577.1525, Found: 577.1523. HPLC analysis of the reaction product: Daicel Chiralpak ODH, hexane/*iso*-propanol = 99:1, 1.0 mL/min, λ= 314 nm, retention time: 25.07 min (major) and 29.95 min (minor).

Ethyl (*R*)-2-(2-chloro-7-(4-methoxyphenyl)-9*H*-carbazol-9-yl)-3-(naphthalen-1-yl)benzoate

(24)

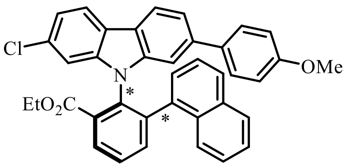

White solid, 82 mg, 56%, $[\alpha]_D^{20}$ = −61.20 (*c* 1.000 $CHCl_3$) for 93:7 er, 1.4:1 dr confirmed by ^{1}H NMR. ^{1}H NMR (500 MHz, Chloroform-*d*) δ 8.19 – 8.16 (m, 1H), 7.98 – 7.82 (m, 3H), 7.80 – 7.56 (m, 4H), 7.54 – 7.27 (m, 4H), 7.19 – 7.14 (m, 1H), 7.12 – 6.97 (m, 3H), 6.95 – 6.76 (m, 4H), 3.86 – 3.82 (m, 3H, 1.77:1.23 = 1.4:1), 3.72 – 3.64 (m, 2H), 7.49 – 7.41 (m, 3H). ^{13}C NMR (126 MHz, Chloroform-*d*) δ 166.3, 166.0, 159.1, 158.8, 143.5_1, 143.4_5, 141.8, 141.8, 141.4, 139.1, 138.9, 136.6, 136.5, 134.5, 134.4, 134.3, 133.6, 133.4, 133.1, 133.0, 131.4, 131.2_7, 131.2_5, 131.2, 130.8, 128.7, 128.6, 128.5_4, 128.5_0, 128.4_0, 128.3_8, 128.3, 126.3, 126.2, 126.1, 125.8, 125.5, 125.2, 125.0, 124.7, 124.4, 121.8, 121.3_3, 121.2_6, 121.0, 120.8, 120.3, 120.2, 120.1, 119.7, 119.3, 119.2, 114.3, 113.7, 110.4, 109.7, 108.5, 107.8, 61.4, 61.3, 55.4_2, 55.3_6, 12.9_9, 12.9_5. HRMS (m/z, ESI): Calcd. for Chemical Formula: $C_{38}H_{29}ClNO_3^+$ $[M+H]^+$: 582.1830, Found: 582.1833. HPLC analysis of the reaction product: Daicel Chiralpak IA, hexane/*iso*-propanol = 99:1, 1.0 mL/min, λ= 314 nm, retention time: 15.48 min (major) and 13.95 min (minor).

Ethyl (*R*)-2-(2-chloro-7-(4-methoxyphenyl)-9*H*-carbazol-9-yl)-2',3',4',5'-tetrahydro-[1,1'-biphenyl]-3-carboxylate (25)

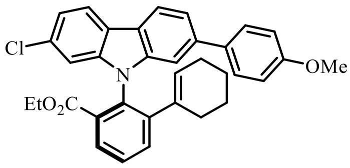

White solid, 95 mg, 71%, $[\alpha]_D^{20}$ = −2.20 (*c* 1.000 $CHCl_3$) for 90.5:9.5 er. ^{1}H NMR (500 MHz, Chloroform-*d*) δ 8.06 (d, *J* = 8.1 Hz, 1H), 7.98 (d, *J* = 8.2 Hz, 1H), 7.94 (dd, *J* = 7.3, 2.0 Hz, 1H), 7.63 – 7.56 (m, 2H), 7.55 – 7.51 (m, 2H), 7.47 – 7.44 (m, 1H), 7.20 (dd, *J* = 8.2, 1.8 Hz, 1H), 7.13 (d, *J* = 1.5 Hz, 1H), 7.00 – 6.94 (m, 3H), 5.59 – 5.57 (m, 1H), 3.84 (s, 3H), 3.69 (d, *J* = 7.1 Hz, 2H),

1.80 – 1.72 (m, 2H), 1.61 – 1.58 (m, 2H), 1.19 – 1.11 (m, 4H), 0.47 (t, *J* = 7.1 Hz, 3H). ^{13}C NMR (126 MHz, Chloroform-*d*) δ 166.5, 159.0, 145.8, 142.6, 142.4, 139.0, 135.9, 134.4, 133.7, 132.5, 132.1, 131.2, 129.9, 128.9, 128.5, 128.4, 121.6, 121.3, 120.7, 120.2, 120.1, 119.3, 114.2, 110.4, 108.2, 61.2, 55.4, 28.4, 25.5, 22.5, 21.5, 13.0. HRMS (m/z, ESI): Calcd. for Chemical Formula: $C_{34}H_{31}ClNO_3^+$ [M+H]$^+$: 536.1987, Found: 536.1985. HPLC analysis of the reaction product: Daicel Chiralpak IA, hexane/*iso*-propanol = 99: 1, 1.0 mL/min, λ= 316 nm, retention time: 8.21 min (major) and 7.33 min (minor).

Ethyl (*R*)-2-(2-chloro-7-(4-methoxyphenyl)-9*H*-carbazol-9-yl)-3-(3,6-dihydro-2*H*-pyran-4-yl)benzoate (26)

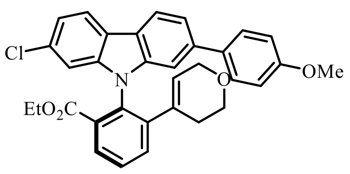

White solid, 90 mg, 67%, $[\alpha]_D^{20}$ = +8.10 (*c* 1.000 $CHCl_3$) for 90.5:9.5 er. ^{1}H NMR (500 MHz, Chloroform-*d*) δ 8.07 (d, *J* = 8.0 Hz, 1H), 8.02 – 7.97 (m, 2H), 7.66 – 7.61 (m, 2H), 7.54 – 7.49 (m, 2H), 7.46 (dd, *J* = 8.1, 1.5 Hz, 1H), 7.21 (dd, *J* = 8.2, 1.8 Hz, 1H), 7.10 (d, *J* = 1.5 Hz, 1H), 6.95 (dd, *J* = 8.7, 2.0 Hz, 3H), 5.60 – 5.58 (m, 1H), 3.90 – 3.81 (m, 5H), 3.69 (q, *J* = 7.1 Hz, 2H), 3.28 – 3.18 (m, 2H), 1.77 – 1.69 (m, 2H), 0.46 (t, *J* = 7.1 Hz, 3H).^{13}C NMR (126 MHz, Chloroform-*d*) δ 166.2, 159.1, 143.5, 142.6, 142.3, 139.3, 134.2, 133.6, 133.5, 132.6, 132.4, 131.4, 130.6, 129.2, 128.4, 126.5, 121.6, 121.3, 121.0, 120.4, 119.6, 114.2, 110.2, 107.9, 65.3, 63.8, 61.3, 55.4, 28.3, 13.0. HRMS (m/z, ESI): Calcd. for Chemical Formula: $C_{33}H_{29}ClNO_4^+$ [M+H]$^+$: 538.1780, Found: 538.1775. HPLC analysis of the reaction product: Daicel Chiralpak IA, hexane/*iso*-propanol = 97:3, 1.0 mL/min, λ= 316 nm, retention time: 12.72 min (major) and 10.75 min (minor).

Ethyl (*S*)-3-chloro-2-(2-chloro-7-(4-methoxyphenyl)-9*H*-carbazol-9-yl)benzoate (27)

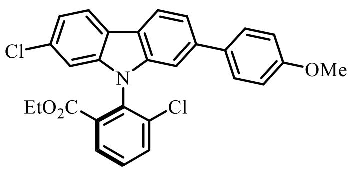

White solid, 79 mg, 65%, $[\alpha]_D^{20}$ = +21.70 (*c* 1.000 $CHCl_3$) for 97:3 er. ^{1}H NMR (500 MHz, Chloroform-*d*) δ 8.10 (dd, *J* = 8.1, 0.6 Hz, 1H), 8.07 – 7.99 (m, 2H), 7.87 (dd, *J* = 8.1, 1.5 Hz, 1H), 7.62 (t, *J* = 8.0 Hz, 1H), 7.56 – 7.47 (m, 3H), 7.25 (dd, *J* = 8.1, 2.0 Hz, 1H), 7.07 (d, *J* = 1.4 Hz, 1H), 6.99 – 6.92 (m, 3H), 3.84 (s, 3H), 3.71 (q, *J* = 7.1 Hz, 2H), 0.47 (t, *J* = 7.1 Hz, 3H). ^{13}C NMR (126 MHz, Chloroform-*d*) δ 164.9, 159.1, 142.1, 142.0, 139.6, 136.0, 134.3, 134.2, 133.9, 133.4, 131.6, 130.4, 130.1, 128.5, 121.9, 121.6, 121.0, 120.7, 120.5, 119.9, 114.2, 109.9, 107.6, 61.6, 55.4, 12.9. HRMS (m/z, ESI): Calcd. for Chemical Formula: $C_{28}H_{22}Cl_2NO_3^+$ $[M+H]^+$: 490.0971, Found: 490.0979. HPLC analysis of the reaction product: Daicel Chiralpak IA, hexane/*iso*-propanol = 99:1, 1.0 mL/min, λ= 314 nm, retention time: 13.21 min (major) and 16.65 min (minor).

(*S*)-2-(2-(3-(((*tert*-Butyldimethylsilyl)oxy)methyl)phenyl)-7-chloro-9*H*-carbazol-9-yl)-3-chlorobenzoate (28)

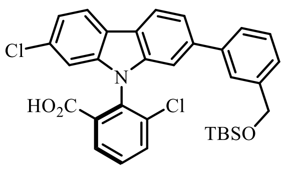

White solid, 70 mg, 47%, one step. ^{1}H NMR (500 MHz, Chloroform-*d*) δ 8.07 – 8.04 (m, 2H), 7.98 (dd, *J* = 8.3, 0.5 Hz, 1H), 7.87 (dd, *J* = 8.1, 1.5 Hz, 1H), 7.60 (t, *J* = 8.0 Hz, 1H), 7.52 – 7.47 (m, 3H), 7.34 – 7.30 (m, 2H), 7.24 – 7.21 (m, 1H), 7.02 (dd, *J* = 1.5, 0.6 Hz, 1H), 6.89 (d, *J* = 1.8 Hz, 1H), 4.75 (s, 2H), 0.94 (s, 9H), 0.10 (s, 6H). ^{13}C NMR (126 MHz, Chloroform-*d*) δ 167.2, 142.0, 141.9, 140.3, 140.2, 139.8, 136.5, 135.2, 134.1, 132.1, 131.7, 130.9, 130.2, 127.3, 126.4, 122.1, 122.0, 121.2, 120.7, 120.5, 120.2, 109.7, 107.8, 64.8, 26.0, 18.4, -5.2. HRMS (m/z, ESI): Calcd. for Chemical Formula: $C_{32}H_{32}Cl_2NO_3Si^+$ $[M+H]^+$: 576.1523, Found: 576.1525.

(*S*)-3-Chloro-2-(2-chloro-7-(3-(hydroxymethyl)phenyl)-9*H*-carbazol-9-yl)benzoate (SI-**28)**

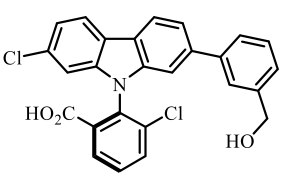

Under the N_2 atmosphere, **28** (70 mg, 0.12 mmol) was dissolved in 1.0 mL THF, then TBAF (1.0 M in THF, 0.18 mL, 0.18 mmol) was added dropwise into the reaction mixture at 0 °C. After that, the mixture was stirred overnight at room temperature. The reaction was then quenched with water, and extracted with ethyl acetate (4 mL X 3) three times. The combined organic phases were washed with brine, dried over Na_2SO_4, and concentrated in vacuo and purified by flash column chromatography with hexane and acetone as the eluents to give the desired product. White solid, 54 mg, 98%. $[\alpha]_D^{20}$ = +16.35 (*c* 1.000 $CHCl_3$) for 93:7 er. ^{1}H NMR (500 MHz, Acetone-*d*$_6$) δ 8.26 (d, *J* = 8.1 Hz, 1H), 8.20 (d, *J* = 8.3 Hz, 1H), 8.14 (d, *J* = 7.9 Hz, 1H), 8.02 (d, *J* = 8.2 Hz, 1H), 7.80 (s, 1H), 7.59 (dd, *J* = 8.0, 2.1 Hz, 3H), 7.41 (d, *J* = 7.9 Hz, 2H), 7.30 – 7.22 (m, 2H), 7.04 (s, 1H), 4.65 (s, 2H). ^{13}C NMR (126 MHz, Acetone-*d*$_6$) δ 142.3, 141.7, 139.9, 139.7, 134.1, 132.8, 131.2, 130.9, 130.5, 127.1, 127.0, 122.1, 122.0, 121.5, 120.8, 120.3, 119.8, 109.7, 107.8, 63.5. HRMS (m/z, ESI): Calcd. for Chemical Formula: $C_{26}H_{18}Cl_2NO_3^+$ $[M+H]^+$: 462.0658, Found: 462.0658. HPLC analysis of the reaction product: Daicel Chiralpak ADH, hexane/*iso*-propanol = 80:20, 1.0 mL/min, λ= 312 nm, retention time: 8.18 min (major) and 19.90 min (minor).

Ethyl (*S*)-2-(2-(3-(((*tert*-butoxycarbonyl)amino)methyl)phenyl)-7-chloro-9*H*-carbazol-9-yl)-3-chlorobenzoate (29)

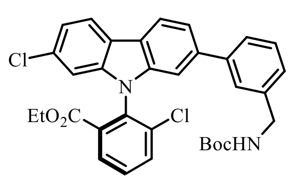

White solid, 89 mg, 64%, $[\alpha]_D^{20}$ = +25.85 (*c* 1.000 $CHCl_3$) for 99.5:0.5 er. ^{1}H NMR (500 MHz, Chloroform-*d*) δ 8.13 (d, *J* = 8.1 Hz, 1H), 8.07 – 8.02 (m, 2H), 7.88 (dd, *J* = 8.2, 1.5 Hz, 1H), 7.63 (t, *J* = 8.0 Hz, 1H), 7.53 – 7.47 (m, 3H), 7.37 (t, *J* = 7.9 Hz, 1H), 7.28 – 7.23 (m, 2H), 7.09 (d, *J* = 1.5 Hz, 1H), 6.95 (d, *J* = 1.8 Hz, 1H), 4.88 (s, 1H), 4.37 (d, *J* = 6.0 Hz, 2H), 3.71 (q, *J* = 7.1 Hz, 2H), 1.46 (s, 9H), 0.47 (t, *J* = 7.1 Hz, 3H). ^{13}C NMR (126 MHz, Chloroform-*d*) δ 164.8, 142.1, 142.1, 139.7, 139.4, 136.1, 134.3, 133.9, 133.3, 131.9, 130.5, 130.2, 129.0, 126.7, 126.3, 122.1, 121.8, 121.2, 120.8, 120.5, 120.3, 109.9, 108.2, 79.5, 61.6, 44.8, 28.4, 12.9. HRMS (m/z, ESI): Calcd. for Chemical Formula: $C_{33}H_{30}Cl_2N_2NaO_4^+$ $[M+Na]^+$: 611.1475, Found: 611.1474. HPLC analysis of the reaction product: Daicel Chiralpak ID, hexane/*iso*-propanol = 95:5, 1.0 mL/min, λ= 310 nm, retention time: 17.05 min (major) and 19.34 min (minor).

Ethyl (*R*)-2-(2-chloro-7-(4-methoxyphenyl)-9*H*-carbazol-9-yl)benzoate (30)

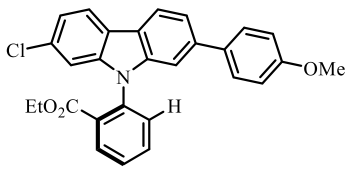

White solid, 83 mg, 73%, racemic. At 40 °C for 20 h, 57 mg, 50%, 66.5:33.5 er ^{1}H NMR (500 MHz, Chloroform-*d*) δ 8.18 (dd, *J* = 7.9, 1.6 Hz, 1H), 8.10 (dd, *J* = 8.1, 0.7 Hz, 1H), 8.03 (d, *J* = 8.2 Hz, 1H), 7.78 (td, *J* = 7.6, 1.7 Hz, 1H), 7.67 – 7.63 (m, 1H), 7.58 (dd, *J* = 7.9, 1.2 Hz, 1H), 7.56 – 7.52 (m, 2H), 7.49 (dd, *J* = 8.1, 1.5 Hz, 1H), 7.24 (dd, *J* = 8.3, 1.8 Hz, 1H), 7.22 (dd, *J* = 1.5, 0.6 Hz, 1H), 7.12 (d, *J* = 1.8 Hz, 1H), 6.99 – 6.94 (m, 2H), 3.84 (s, 3H), 3.75 (q, *J* = 7.1 Hz, 2H), 0.49 (t, *J* = 7.2 Hz, 3H). ^{13}C NMR (126 MHz, Chloroform-*d*) δ 166.0, 159.2, 142.8, 142.7, 139.6, 136.1, 134.3, 133.7, 132.5, 131.6, 130.7, 130.3, 129.0, 128.6, 121.8, 121.5, 121.1, 120.5_3, 120.5_0, 119.7, 114.3, 109.7, 107.6, 61.4, 55.5, 13.1. HRMS (m/z, ESI): Calcd. for Chemical Formula:

$C_{28}H_{23}ClNO_3^+$ [M+H]$^+$: 456.1361, Found: 456.1359.

The reaction was performed with the substrate 2-(2,7-dichloro-9*H*-carbazol-9-yl)benzoic acid (0.1 mmol), *p*-methoxyphenylboronic acid (0.14 mmol), $Pd_2(dba)_3$ (2.0 mol%), (S_a, *R*)-**L9** (4.0 mol%), K_3PO_4 (0.5 mmol) in 0.25 mL THF and 0.04 mL H_2O at 40 °C for 1 h. White solid, 8 mg, 17%, $[\alpha]_D^{20}$ = +17.10 (*c* 0.600 $CHCl_3$) for 91:9 er. HPLC analysis of the reaction product: Daicel Chiralpak IA, hexane/*iso*-propanol = 99:1, 1.0 mL/min, λ= 316 nm, retention time: 13.36 min (major) and 15.89 min (minor).

Ethyl (*R*)-2-(2-chloro-3,6-dimethoxy-7-(4-methoxyphenyl)-9*H*-carbazol-9-yl)-3-methylbenzoate (31)

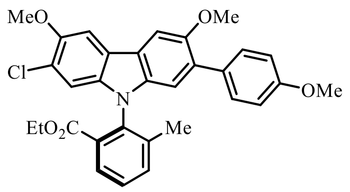

Yellow solid, 66 mg, 50%, $[\alpha]_D^{20}$ = +25.90 (*c* 0.500 $CHCl_3$) for 93:7 er. ^{1}H NMR (500 MHz, Chloroform-*d*) δ 7.90 (dd, *J* = 7.8, 1.7 Hz, 1H), 7.61 (d, *J* = 14.1 Hz, 3H), 7.51 (t, *J* = 7.7 Hz, 1H), 7.48 – 7.43 (m, 2H), 6.96 (s, 1H), 6.94 – 6.90 (m, 2H), 6.81 (s, 1H), 4.06 (s, 3H), 3.94 (s, 3H), 3.82 (s, 3H), 3.74 (qd, *J* = 7.2, 3.7 Hz, 2H), 2.00 (s, 3H), 0.57 (t, *J* = 7.1 Hz, 3H). ^{13}C NMR (126 MHz, Chloroform-*d*) δ 166.1, 158.7, 151.3, 149.2, 139.4, 136.9, 136.5, 134.9, 134.5, 132.2, 131.5, 130.8, 130.4, 129.4, 128.9, 121.8, 121.6, 121.6, 113.5, 111.5, 111.2, 103.2, 102.1, 61.1, 57.0, 56.4, 55.3, 17.6, 13.2. HRMS (m/z, ESI): Calcd. for Chemical Formula: $C_{31}H_{29}ClNO_5^+$ [M+H]$^+$: 530.1729, Found: 530.1731. HPLC analysis of the reaction product: Daicel Chiralpak IF, hexane/*iso*-propanol = 95:5, 1.0 mL/min, λ= 272 nm, retention time: 7.76 min (major) and 8.57 min (minor).

Ethyl (*S*)-3-chloro-2-(2-chloro-8-(4-methoxyphenyl)-10*H*-phenoxazin-10-yl)benzoate (32)

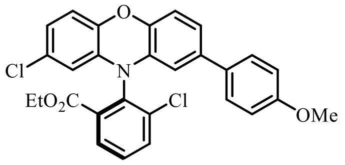

White solid, 79 mg, 63%, $[\alpha]_D^{20}$ = +6.30 (*c* 0.500 $CHCl_3$) for 63.5:36.5 er. After two days, it was racemic. ^1H NMR (500 MHz, Chloroform-*d*) δ 8.06 (dd, *J* = 7.9, 1.5 Hz, 1H), 7.85 (dd, *J* = 8.1, 1.6 Hz, 1H), 7.56 (t, *J* = 8.0 Hz, 1H), 7.23 – 7.18 (m, 2H), 6.88 – 6.82 (m, 3H), 6.74 (d, *J* = 8.2 Hz, 1H), 6.66 – 6.61 (m, 2H), 5.86 (d, *J* = 2.1 Hz, 1H), 5.71 (d, *J* = 2.1 Hz, 1H), 4.22 (qd, *J* = 7.1, 1.9 Hz, 2H), 3.78 (s, 3H), 1.11 (t, *J* = 7.2 Hz, 3H). ^{13}C NMR (101 MHz, Chloroform-*d*) δ 164.7, 158.9, 142.8, 142.5, 137.8, 136.5, 135.1, 134.6, 134.1, 133.4, 133.1, 131.8, 131.5, 130.3, 128.2, 127.6, 121.1, 120.4, 116.4, 115.8, 114.0, 112.6, 111.1, 62.1, 55.3, 13.7. HRMS (m/z, ESI): Calcd. for Chemical Formula: $C_{28}H_{22}Cl_2NO_4^+$ $[M+H]^+$: 506.0920, Found: 506.0915. HPLC analysis of the reaction product: Daicel Chiralpak IC, hexane/*iso*-propanol = 99:1, 1.0 mL/min, λ= 328 nm, retention time: 8.88 min (major) and 9.58 min (minor).

The reaction was performed with the substrate 3-chloro-2-(2,8-dichloro-10*H*-phenoxazin-10-yl)benzoic acid (0.1 mmol), *p*-methoxyphenylboronic acid (0.14 mmol), $Pd_2(dba)_3$ (2.0 mol%), ($S_{a,}$ *R*)-**L9** (4.0 mol%), K_3PO_4 (0.5 mmol) in 0.25 mL THF and 0.04 mL H_2O at 50 °C for 1 h. White solid, 3 mg, 6%, 85.5:14.5 er. HPLC analysis of the reaction product: Daicel Chiralpak IC and IC, hexane/*iso*-propanol = 99.5:0.5, 1.0 mL/min, λ= 328 nm, retention time: 19.59 min (major) and 21.44 min (minor).

Ethyl (*S*)-3-chloro-2-(3-chloro-6-(4-methoxyphenyl)-9,9-dimethylacridin-10(9*H*)-yl)benzoate (33)

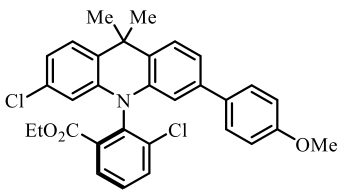

Colorless oil, 68 mg, 51%. $[\alpha]_D^{20}$ = −32.50 (*c* 1.000 $CHCl_3$) for 91.5:8.5 er. ^{1}H NMR (500 MHz, Chloroform-*d*) δ 8.07 (dd, *J* = 7.9, 1.6 Hz, 1H), 7.87 (dd, *J* = 8.1, 1.5 Hz, 1H), 7.59 (t, *J* = 8.0 Hz, 1H), 7.49 (d, *J* = 8.1 Hz, 1H), 7.40 (d, *J* = 8.4 Hz, 1H), 7.30 – 7.27 (m, 2H), 7.12 (dd, *J* = 8.0, 1.8 Hz, 1H), 6.92 – 6.85 (m, 3H), 6.15 (d, *J* = 1.8 Hz, 1H), 5.99 (d, *J* = 2.1 Hz, 1H), 3.98 (qd, *J* = 7.1, 1.5 Hz, 2H), 3.80 (s, 3H), 1.79 (s, 3H), 1.67 (s, 3H), 0.79 (t, *J* = 7.1 Hz, 3H). ^{13}C NMR (126 MHz, Chloroform-*d*) δ 164.8, 159.0, 140.3, 139.4, 138.7, 137.4, 136.3, 135.3, 135.2, 133.7, 132.2, 130.9, 129.9, 128.3, 128.1, 128.0, 127.2, 126.3, 120.7, 119.9, 114.1, 112.9, 111.3, 61.6, 55.3, 35.6, 34.5, 31.4, 13.4. HRMS (m/z, ESI): Calcd. for Chemical Formula: $C_{31}H_{28}Cl_2NO_3^+$ $[M+H]^+$: 532.1441, Found: 532.1442. HPLC analysis of the reaction product: Daicel Chiralpak IA, hexane/*iso*-propanol = 99:1, 1.0 mL/min, λ= 281 nm, retention time: 12.10 min (major) and 9.85 min (minor).

Ethyl (*R*)-1-(2-chloro-7-(4-(ethoxycarbonyl)phenyl)-9*H*-carbazol-9-yl)-5-ethyl-1*H*-pyrrole-2-carboxylate (35)

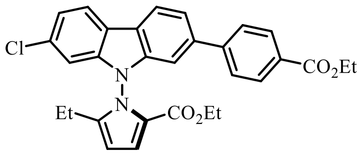

White solid, 85 mg, 66%, $[\alpha]_D^{20}$ = +4.00 (*c* 1.000 $CHCl_3$) for 99:1 er. ^{1}H NMR (500 MHz, Chloroform-*d*) δ 8.15 – 8.08 (m, 3H), 8.03 (d, *J* = 8.2 Hz, 1H), 7.68 – 7.64 (m, 2H), 7.59 (dd, *J* = 8.1, 1.6 Hz, 1H), 7.29 (dd, *J* = 8.3, 1.8 Hz, 1H), 7.23 (d, *J* = 4.3 Hz, 1H), 7.12 (d, *J* = 1.5 Hz, 1H), 6.94 (d, *J* = 1.8 Hz, 1H), 6.27 (d, *J* = 4.2 Hz, 1H), 4.40 (q, *J* = 7.1 Hz, 2H), 3.83 (q, *J* = 7.1 Hz, 2H), 2.36 (q, *J* = 7.5 Hz, 2H), 1.41 (t, *J* = 7.1 Hz, 3H), 1.16 (t, *J* = 7.6 Hz, 3H), 0.74 (t, *J* = 7.1 Hz, 3H).^{13}C NMR (126 MHz, Chloroform-*d*) δ 166.5, 158.8, 145.5, 143.6, 141.9, 141.6, 139.2, 132.9, 130.1, 129.3, 127.4, 122.0, 121.8, 121.6, 121.0, 121.0, 120.9, 119.8, 118.3, 108.7, 106.9, 105.7, 61.0, 59.9,

18.6, 14.4, 13.6, 12.5. HRMS (m/z, ESI): Calcd. for Chemical Formula: $C_{30}H_{28}ClN_2O_4^+$ $[M+H]^+$: 515.1732, Found: 515.1729. HPLC analysis of the reaction product: Daicel Chiralpak IC, hexane/*iso*-propanol = 99:1, 1.0 mL/min, λ= 333 nm, retention time: 35.86 min (major) and 32.25 min (minor).

Ethyl (*R*)-1-(2-chloro-7-(4-cyanophenyl)-9*H*-carbazol-9-yl)-5-ethyl-1*H*-pyrrole-2-carboxylate (36)

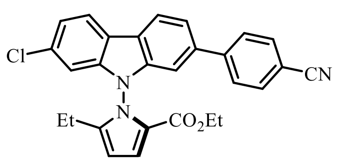

White solid, 75 mg, 64%, $[\alpha]_D^{20}$ = +5.40 (*c* 1.000 $CHCl_3$) for 99.5:0.5 er. ^{1}H NMR (500 MHz, Chloroform-*d*) δ 8.15 (d, *J* = 8.1 Hz, 1H), 8.03 (d, *J* = 8.4 Hz, 1H), 7.73 – 7.68 (m, 4H), 7.55 (dd, *J* = 8.2, 1.6 Hz, 1H), 7.30 (dd, *J* = 8.3, 1.9 Hz, 1H), 7.23 (d, *J* = 4.3 Hz, 1H), 7.08 (d, *J* = 1.5 Hz, 1H), 6.94 (d, *J* = 1.8 Hz, 1H), 6.28 (d, *J* = 4.3 Hz, 1H), 3.84 (q, *J* = 7.2 Hz, 2H), 2.35 (q, *J* = 7.6 Hz, 2H), 1.16 (t, *J* = 7.5 Hz, 3H), 0.76 (t, *J* = 7.1 Hz, 3H). ^{13}C NMR (126 MHz, Chloroform-*d*) δ 158.7, 145.7, 143.5, 142.0, 141.6, 138.2, 133.2, 132.6, 128.1, 122.0, 121.7, 121.2, 120.9, 119.7, 118.9, 118.3, 110.9, 108.7, 106.9, 105.7, 59.9, 18.6, 13.6, 12.5. HRMS (m/z, ESI): Calcd. for Chemical Formula: $C_{28}H_{23}ClN_3O_2^+$ $[M+H]^+$: 468.1473, Found: 468.1473. HPLC analysis of the reaction product: Daicel Chiralpak IC, hexane/*iso*-propanol = 95:5, 1.0 mL/min, λ= 315 nm, retention time: 42.07 min (major) and 36.22 min (minor).

Ethyl (*R*)-1-(2-chloro-7-(4-nitrophenyl)-9*H*-carbazol-9-yl)-5-ethyl-1*H*-pyrrole-2-carboxylate (37)

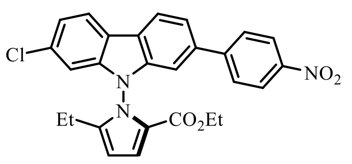

Yellow solid, 75 mg, 62%, $[\alpha]_D^{20}$ = +11.40 (*c* 1.000 $CHCl_3$) for 98.5:1.5 er. ^{1}H NMR (500 MHz, Chloroform-*d*) δ 8.31 – 8.26 (m, 2H), 8.17 (d, *J* = 8.1 Hz, 1H), 8.04 (d, *J* = 8.3 Hz, 1H), 7.78 – 7.71 (m, 2H), 7.58 (dd, *J* = 8.1, 1.6 Hz, 1H), 7.31 (dd, *J* = 8.3, 1.8 Hz, 1H), 7.23 (d, *J* = 4.4 Hz, 1H), 7.12 (d, *J* = 1.6 Hz, 1H), 6.94 (d, *J* = 1.8 Hz, 1H), 6.28 (d, *J* = 4.4 Hz, 1H), 3.85 (q, *J* = 7.1 Hz, 2H), 2.36 (q, *J* = 7.6 Hz, 2H), 1.16 (t, *J* = 7.6 Hz, 3H), 0.77 (t, *J* = 7.1 Hz, 3H). ^{13}C NMR (126 MHz, Chloroform-*d*) δ 158.7, 147.6, 147.1, 143.5, 142.0, 141.6, 137.7, 133.3, 128.1, 124.1, 122.0, 121.8, 121.6, 121.3, 121.0, 119.6, 118.3, 108.8, 107.1, 105.8, 59.9, 18.6, 13.6, 12.5. HRMS (m/z, ESI): Calcd. for Chemical Formula: $C_{27}H_{23}ClN_3O_4^+$ $[M+H]^+$:488.1372, Found: 488.1370. HPLC analysis of the reaction product: Daicel Chiralpak IC, hexane/*iso*-propanol = 95:5, 1.0 mL/min, λ= 334 nm, retention time: 50.51 min (major) and 27.52 min (minor).

Ethyl (*R*)-1-(2-chloro-7-(4-methoxyphenyl)-9*H*-carbazol-9-yl)-5-ethyl-1*H*-pyrrole-2-carboxylate (38)

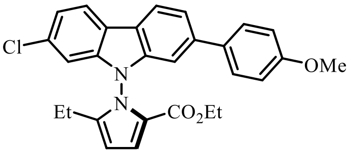

White solid, 80 mg, 68%, $[\alpha]_D^{20}$ = +9.10 (*c* 1.000 $CHCl_3$) for 99.5:0.5 er. ^{1}H NMR (500 MHz, Chloroform-*d*) δ 8.08 (d, *J* = 8.1 Hz, 1H), 8.00 (d, *J* = 8.2 Hz, 1H), 7.56 – 7.50 (m, 3H), 7.28 (dd, *J* = 8.3, 1.8 Hz, 1H), 7.23 (d, *J* = 4.4 Hz, 1H), 7.04 (d, *J* = 1.6 Hz, 1H), 6.99 – 6.95 (m, 2H), 6.94 (d, *J* = 1.8 Hz, 1H), 6.26 (dd, *J* = 4.3, 0.9 Hz, 1H), 3.86 – 3.80 (m, 5H), 2.37 (q, *J* = 7.5 Hz, 2H), 1.16 (t, *J* = 7.5 Hz, 3H), 0.72 (t, *J* = 7.1 Hz, 3H). ^{13}C NMR (101 MHz, Chloroform-*d*) δ 159.3, 158.8, 143.6, 141.8, 141.7, 140.2, 133.8, 132.3, 128.5, 122.1, 121.6, 121.3, 120.7, 120.6, 120.1, 119.7, 118.2, 114.3, 108.6, 106.2, 105.5, 59.8, 55.4, 18.6, 13.5, 12.5. HRMS (m/z, ESI): Calcd. for

Chemical Formula: $C_{28}H_{26}ClN_2O_3^+$ $[M+H]^+$: 473.1626, Found: 473.1623. HPLC analysis of the reaction product: Daicel Chiralpak IC, hexane/*iso*-propanol = 99:1, 1.0 mL/min, λ= 327 nm, retention time: 11.89 min (major) and 13.00 min (minor).

Ethyl (*R*)-1-(2-chloro-7-(3-nitrophenyl)-9*H*-carbazol-9-yl)-5-ethyl-1*H*-pyrrole-2-carboxylate (39)

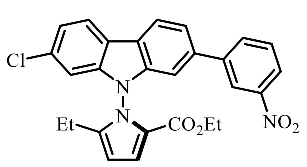

White solid, 75 mg, 62%, $[\alpha]_D^{20}$ = +3.90 (*c* 1.000 $CHCl_3$) for 96.5:3.5 er. ^{1}H NMR (500 MHz, Chloroform-*d*) δ 8.46 (t, *J* = 2.0 Hz, 1H), 8.22 – 8.15 (m, 2H), 8.04 (d, *J* = 8.3 Hz, 1H), 7.93 – 7.91 (m, 1H), 7.62 – 7.57 (m, 2H), 7.31 (dd, *J* = 8.3, 1.8 Hz, 1H), 7.24 (d, *J* = 4.3 Hz, 1H), 7.12 (d, *J* = 1.6 Hz, 1H), 6.93 (d, *J* = 1.8 Hz, 1H), 6.29 (d, *J* = 4.3 Hz, 1H), 3.85 (q, *J* = 7.1 Hz, 2H), 2.37 (q, *J* = 7.6 Hz, 2H), 1.17 (t, *J* = 7.5 Hz, 3H), 0.78 (t, *J* = 7.1 Hz, 3H). ^{13}C NMR (126 MHz, Chloroform-*d*) δ 158.8, 148.7, 143.5, 143.0, 142.0, 141.7, 137.7, 133.5, 133.1, 129.7, 122.3, 122.1, 122.0, 121.7, 121.3, 121.2, 120.9, 119.7, 118.3, 108.7, 106.8, 105.8, 59.9, 18.6, 13.6, 12.5. HRMS (m/z, ESI): Calcd. for Chemical Formula: $C_{27}H_{23}ClN_3O_4^+$ $[M+H]^+$: 488.1372, Found: 488.1381. HPLC analysis of the reaction product: Daicel Chiralpak IF, hexane/*iso*-propanol = 99:1, 1.0 mL/min, λ= 333 nm, retention time: 14.99 min (major) and 16.06 min (minor).

Ethyl (*R*)-1-(2-chloro-7-(3-methoxyphenyl)-9*H*-carbazol-9-yl)-5-ethyl-1*H*-pyrrole-2-carboxylate (40)

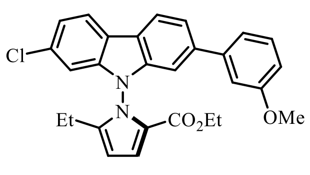

White solid, 83 mg, 70%. $[\alpha]_D^{20}$ = +7.20 (*c* 1.000 $CHCl_3$) for 98:2 er. ^{1}H NMR (500 MHz, Chloroform-*d*) δ 8.10 (d, *J* = 8.1 Hz, 1H), 8.01 (d, *J* = 8.3 Hz, 1H), 7.56 (dd, *J* = 8.1, 1.6 Hz, 1H), 7.35 (t, *J* = 7.9 Hz, 1H), 7.28 (dd, *J* = 8.3, 1.8 Hz, 1H), 7.23 – 7.17 (m, 2H), 7.13 (t, *J* = 2.1 Hz, 1H), 7.08 (d, *J* = 1.5 Hz, 1H), 6.96 – 6.86 (m, 2H), 6.25 (dd, *J* = 4.3, 0.9 Hz, 1H), 3.90 – 3.79 (m, 5H), 2.36 (q, *J* = 7.6 Hz, 2H), 1.15 (t, *J* = 7.5 Hz, 3H), 0.73 (t, *J* = 7.1 Hz, 3H). ^{13}C NMR (126 MHz, Chloroform-*d*) δ 159.9, 158.8, 143.5, 142.8, 141.8, 141.6, 140.3, 132.5, 129.8, 122.0, 121.6, 121.4, 121.0, 120.8, 120.3, 120.1, 118.2, 113.7, 112.3, 108.6, 106.8, 105.6, 59.8, 55.4, 18.6, 13.5, 12.5. HRMS (m/z, ESI): Calcd. for Chemical Formula: $C_{28}H_{26}ClN_2O_3^+$ $[M+H]^+$: 473.1626, Found: 473.1629. HPLC analysis of the reaction product: Daicel Chiralpak IC, hexane/*iso*-propanol = 99:1, 1.0 mL/min, λ= 323 nm, retention time: 9.78 min (major) and 11.04 min (minor).

Ethyl (*R*)-1-(2-(3-(benzyloxy)phenyl)-7-chloro-9*H*-carbazol-9-yl)-5-ethyl-1*H*-pyrrole-2-carboxylate (41)

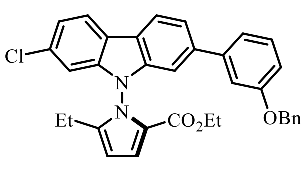

White solid, 80 mg, 58%, $[\alpha]_D^{20}$ = +5.35 (*c* 1.000 $CHCl_3$) for 99.5:0.5 er. ^{1}H NMR (500 MHz, Chloroform-*d*) δ 8.10 (d, *J* = 8.1 Hz, 1H), 8.01 (d, *J* = 8.3 Hz, 1H), 7.55 (dd, *J* = 8.1, 1.5 Hz, 1H), 7.46 (d, *J* = 7.1 Hz, 2H), 7.40 (t, *J* = 7.5 Hz, 2H), 7.37 – 7.32 (m, 2H), 7.28 (dd, *J* = 8.3, 1.8 Hz, 1H), 7.24 – 7.18 (m, 3H), 7.07 (d, *J* = 1.6 Hz, 1H), 6.99 – 6.90 (m, 2H), 6.26 (d, *J* = 4.3 Hz, 1H), 5.12 (s, 2H), 3.82 (qd, *J* = 7.1, 1.5 Hz, 2H), 2.35 (q, *J* = 7.5 Hz, 2H), 1.15 (t, *J* = 7.5 Hz, 3H), 0.72 (t, *J* = 7.1 Hz, 3H).^{13}C NMR (126 MHz, Chloroform-*d*) δ 159.2, 158.8, 143.5, 142.8, 141.8, 141.6, 140.2, 136.9, 132.5, 129.8, 128.7, 128.1, 127.6, 122.0, 121.6, 121.4, 121.0, 120.8, 120.3, 120.0, 118.2, 114.6,

113.3, 108.6, 106.8, 105.6, 70.2, 59.8, 18.6, 13.5, 12.5. HRMS (m/z, ESI): Calcd. for Chemical Formula: $C_{34}H_{30}ClN_2O_3^+$ $[M+H]^+$: 549.1939, Found: 549.1930. HPLC analysis of the reaction product: Daicel Chiralpak IC, hexane/*iso*-propanol = 99:1, 1.0 mL/min, λ= 336 nm, retention time: 10.53 min (major) and 11.34 min (minor).

Ethyl (*R*)-1-(2-chloro-7-(6-methoxypyridin-3-yl)-9*H*-carbazol-9-yl)-5-ethyl-1*H*-pyrrole-2-carboxylate (42)

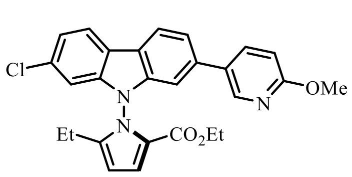

White solid, 70 mg, 59%, $[\alpha]_D^{20}$ = +2.10 (*c* 1.000 $CHCl_3$) for 98.5:1.5 er. ^{1}H NMR (500 MHz, Chloroform-*d*) δ 8.39 (d, *J* = 2.5 Hz, 1H), 8.11 (d, *J* = 8.1 Hz, 1H), 8.01 (d, *J* = 8.3 Hz, 1H), 7.80 (dd, *J* = 8.6, 2.6 Hz, 1H), 7.48 (dd, *J* = 8.1, 1.6 Hz, 1H), 7.28 (dd, *J* = 8.3, 1.8 Hz, 1H), 7.21 (d, *J* = 4.3 Hz, 1H), 7.00 (d, *J* = 1.6 Hz, 1H), 6.95 (d, *J* = 1.8 Hz, 1H), 6.81 (d, *J* = 8.6 Hz, 1H), 6.26 (d, *J* = 4.3 Hz, 1H), 3.97 (s, 3H), 3.83 (q, *J* = 7.1 Hz, 2H), 2.36 (qd, *J* = 7.6, 2.7 Hz, 2H), 1.15 (t, *J* = 7.6 Hz, 3H), 0.75 (t, *J* = 7.1 Hz, 3H). ^{13}C NMR (126 MHz, Chloroform-*d*) δ 163.7, 158.8, 145.3, 143.5, 141.8, 137.7, 137.1, 132.6, 130.2, 122.0, 121.7, 121.4, 121.0, 120.3, 120.2, 119.9, 118.2, 110.8, 108.7, 106.2, 105.6, 59.8, 53.6, 18.6, 13.6, 12.5. HRMS (m/z, ESI): Calcd. for Chemical Formula: $C_{27}H_{25}ClN_3O_3^+$ $[M+H]^+$: 474.1579, Found: 474.1577. HPLC analysis of the reaction product: Daicel Chiralpak IF, hexane/*iso*-propanol = 97: 3, 1.0 mL/min, λ= 334 nm, retention time: 7.38 min (major) and 10.24 min (minor).

Ethyl (*R*)-1-(2-chloro-7-(furan-3-yl)-9*H*-carbazol-9-yl)-5-ethyl-1*H*-pyrrole-2-carboxylate (43)

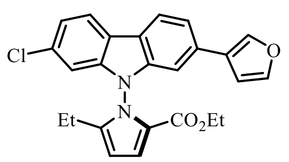

White solid, 81 mg, 75%, $[\alpha]_D^{20}$ = +3.25 (*c* 1.000 $CHCl_3$) for 99:1 er. ^1H NMR (500 MHz, Chloroform-*d*) δ 8.04 (d, *J* = 8.1 Hz, 1H), 7.98 (d, *J* = 8.3 Hz, 1H), 7.75 (t, *J* = 1.2 Hz, 1H), 7.50 – 7.43 (m, 2H), 7.29 – 7.25 (m, 1H), 7.23 (d, *J* = 4.3 Hz, 1H), 6.96 (d, *J* = 1.5 Hz, 1H), 6.91 (d, *J* = 1.9 Hz, 1H), 6.71 (d, *J* = 1.9 Hz, 1H), 6.27 (d, *J* = 4.3 Hz, 1H), 3.82 (q, *J* = 7.1 Hz, 2H), 2.35 (q, *J* = 7.5 Hz, 2H), 1.15 (t, *J* = 7.5 Hz, 3H), 0.71 (t, *J* = 7.1 Hz, 3H). ^{13}C NMR (126 MHz, Chloroform-*d*) δ 158.8, 143.7, 143.6, 141.7, 138.9, 132.4, 131.6, 126.7, 122.1, 121.6, 121.2, 120.9, 120.1, 120.0, 119.7, 118.2, 109.1, 108.6, 105.6, 105.3, 59.8, 18.6, 13.5, 12.4. HRMS (m/z, ESI): Calcd. for Chemical Formula: $C_{25}H_{22}ClN_2O_3^+$ $[M+H]^+$: 433.1313, Found: 433.1314. HPLC analysis of the reaction product: Daicel Chiralpak IC, hexane/*iso*-propanol = 99:1, 0.6 mL/min, λ= 315 nm, retention time: 14.72 min (major) and 13.69 min (minor).

Ethyl (*R*)-1-(2-chloro-7-(2-methoxypyrimidin-5-yl)-9*H*-carbazol-9-yl)-5-ethyl-1*H*-pyrrole-2-carboxylate (44)

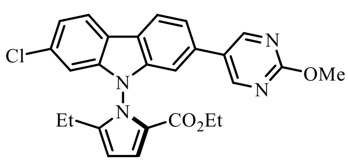

White solid, 71 mg, 60%, $[\alpha]_D^{20}$ = +13.70 (*c* 1.000 $CHCl_3$) for 96.5:3.5 er. ^1H NMR (500 MHz, Chloroform-*d*) δ 8.73 (s, 2H), 8.15 (d, *J* = 8.0 Hz, 1H), 8.03 (d, *J* = 8.3 Hz, 1H), 7.48 – 7.43 (m, 1H), 7.30 (d, *J* = 8.3 Hz, 1H), 7.22 (d, *J* = 4.3 Hz, 1H), 6.99 (d, *J* = 1.6 Hz, 1H), 6.95 (d, *J* = 1.8 Hz, 1H), 6.27 (d, *J* = 4.3 Hz, 1H), 4.06 (s, 3H), 3.85 (q, *J* = 7.1 Hz, 2H), 2.35 (qd, *J* = 7.8, 3.0 Hz, 2H), 1.16 (t, *J* = 7.4 Hz, 3H), 0.79 (t, *J* = 7.1 Hz, 3H). ^{13}C NMR (101 MHz, Chloroform-*d*) δ 165.1, 158.7, 157.6, 143.5, 141.8, 141.7, 133.4, 133.0, 128.4, 121.9, 121.6, 121.4, 120.9, 120.1, 119.7, 118.3, 108.7, 106.1, 105.8, 59.9, 55.1, 18.6, 13.6, 12.5. HRMS (m/z, ESI): Calcd. for Chemical Formula:

$C_{26}H_{24}ClN_4O_3^+$ [M+H]+: 475.1531, Found: 475.1730. HPLC analysis of the reaction product: Daicel Chiralpak IC, hexane/*iso*-propanol = 95:5, 1.0 mL/min, λ= 307 nm, retention time: 56.41 min (major) and 47.89 min (minor).

Ethyl (*R*)-1-(2-chloro-7-(9,9-dimethyl-9*H*-fluoren-2-yl)-9*H*-carbazol-9-yl)-5-ethyl-1*H*-pyrrole-2-carboxylate (45)

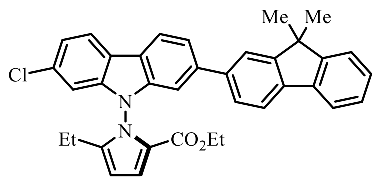

White solid, 75 mg, 57%, $[\alpha]_D^{20}$ = +14.40 (*c* 1.000 $CHCl_3$) for 99:1 er. ^{1}H NMR (500 MHz, Chloroform-*d*) δ 8.13 (d, *J* = 8.1 Hz, 1H), 8.03 (d, *J* = 8.3 Hz, 1H), 7.79 – 7.72 (m, 2H), 7.66 – 7.60 (m, 2H), 7.56 (dd, *J* = 7.9, 1.7 Hz, 1H), 7.47 – 7.44 (m, 1H), 7.37 – 7.31 (m, 2H), 7.29 (dd, *J* = 8.2, 1.8 Hz, 1H), 7.24 (d, *J* = 4.4 Hz, 1H), 7.14 (d, *J* = 1.5 Hz, 1H), 6.93 (d, *J* = 1.8 Hz, 1H), 6.27 (d, *J* = 4.3 Hz, 1H), 3.84 (q, *J* = 7.1 Hz, 2H), 2.38 (qd, *J* = 7.8, 2.7 Hz, 2H), 1.54 (s, 6H), 1.17 (t, *J* = 7.5 Hz, 3H), 0.73 (t, *J* = 7.2 Hz, 3H). ^{13}C NMR (126 MHz, Chloroform-*d*) δ 158.9, 154.3, 153.9, 143.6, 141.8, 141.7, 141.0, 140.4, 138.7, 138.7, 132.5, 127.4, 127.1, 126.7, 122.6, 122.1, 121.7, 121.6, 121.4, 121.2, 120.8, 120.3, 120.1, 120.1, 118.2, 108.6, 106.7, 105.6, 59.9, 47.0, 27.3, 18.7, 13.5, 12.5. HRMS (m/z, ESI): Calcd. for Chemical Formula: $C_{36}H_{32}ClN_2O_2^+$ [M+H]+: 559.2147, Found: 559.2151. HPLC analysis of the reaction product: Daicel Chiralpak IC, hexane/*iso*-propanol = 99:1, 1.0 mL/min, λ= 346 nm, retention time: 7.57 min (major) and 8.17 min (minor).

Ethyl (*R*)-1-(2-chloro-7-(naphthalen-2-yl)-9*H*-carbazol-9-yl)-5-ethyl-1*H*-pyrrole-2-carboxylate (46)

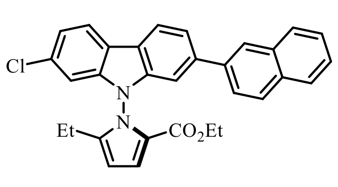

White solid, 80 mg, 65%. $[\alpha]_D^{20}$ = +11.90 (*c* 1.000 $CHCl_3$) for 99.5:0.5 er. ^{1}H NMR (500 MHz, Chloroform-*d*) δ 8.16 (d, *J* = 8.1 Hz, 1H), 8.07 – 8.01 (m, 2H), 7.91 (d, *J* = 8.1 Hz, 2H), 7.88 – 7.84 (m, 1H), 7.75 (dd, *J* = 8.5, 1.9 Hz, 1H), 7.70 (dd, *J* = 8.1, 1.6 Hz, 1H), 7.53 – 7.47 (m, 2H), 7.30 (dd, *J* = 8.3, 1.8 Hz, 1H), 7.25 (s, 1H), 7.22 (d, *J* = 1.6 Hz, 1H), 6.95 (d, *J* = 1.8 Hz, 1H), 6.29 (d, *J* = 4.3 Hz, 1H), 3.88 – 3.80 (m, 2H), 2.39 (q, *J* = 7.5 Hz, 2H), 1.17 (t, *J* = 7.5 Hz, 3H), 0.74 (t, *J* = 7.1 Hz, 3H). ^{13}C NMR (126 MHz, Chloroform-*d*) δ 158.8, 143.6, 141.9, 141.8, 140.4, 138.5, 133.7, 132.7, 132.6, 128.5, 128.2, 127.7, 126.4, 126.1_4, 126.0_7, 125.8, 122.1, 121.7, 121.5, 121.3, 120.9, 120.3, 120.0, 118.2, 108.6, 107.0, 105.6, 59.9, 18.7, 13.5, 12.5. HRMS (m/z, ESI): Calcd. for Chemical Formula: $C_{31}H_{26}ClN_2O_2^+$ $[M+H]^+$: 493.1677, Found: 493.1674. HPLC analysis of the reaction product: Daicel Chiralpak IC, hexane/*iso*-propanol = 99:1, 0.6 mL/min, λ= 323 nm, retention time: 16.49 min (major) and 17.38 min (minor).

Ethyl (*R*)-1-(2-chloro-7-(dibenzo[b,d]thiophen-4-yl)-9*H*-carbazol-9-yl)-5-ethyl-1*H*-pyrrole-2-carboxylate (47)

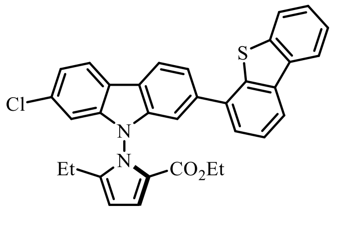

White solid, 80 mg, 62%. $[\alpha]_D^{20}$ = +26.80 (*c* 1.000 $CHCl_3$) for 99:1 er. ^{1}H NMR (500 MHz, Chloroform-*d*) δ 8.21 – 8.13 (m, 3H), 8.06 (d, *J* = 8.3 Hz, 1H), 7.83 – 7.78 (m, 1H), 7.69 (dd, *J* = 8.1, 1.5 Hz, 1H), 7.55 (t, *J* = 7.5 Hz, 1H), 7.51 (dd, *J* = 7.3, 1.3 Hz, 1H), 7.49 – 7.44 (m, 2H), 7.33 – 7.28 (m, 2H), 7.20 (d, *J* = 4.3 Hz, 1H), 6.98 (d, *J* = 1.8 Hz, 1H), 6.23 (d, *J* = 4.3 Hz, 1H), 3.90 – 3.83 (m, 2H), 2.43 (q, *J* = 7.6 Hz, 2H), 1.19 (t, *J* = 7.5 Hz, 3H), 0.75 (t, *J* = 7.1 Hz, 3H). ^{13}C NMR (126 MHz, Chloroform-*d*) δ 158.8, 143.6, 141.9, 141.5, 139.6, 139.5, 138.9, 137.1, 136.3, 135.8, 132.7, 127.1,

126.9, 125.1, 124.4, 122.6, 122.1, 121.9, 121.8, 121.7, 121.6, 120.9, 120.7, 120.6, 120.0, 118.3, 108.7, 108.2, 105.6, 59.9, 18.7, 13.6, 12.5. HRMS (m/z, ESI): Calcd. for Chemical Formula: $C_{33}H_{26}ClN_2O_2S^+$ $[M+H]^+$: 549.1398, Found: 549.1394. HPLC analysis of the reaction product: Daicel Chiralpak IC, hexane/*iso*-propanol = 99:1, 1.0 mL/min, λ= 308 nm, retention time: 10.28 min (major) and 10.86 min (minor).

Ethyl (*R*)-1-(7-chloro-9'-phenyl-9*H*,9'*H*-[2,3'-bicarbazol]-9-yl)-5-ethyl-1*H*-pyrrole-2-carboxylate (48)

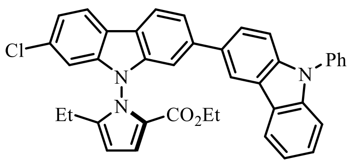

White solid, 86 mg, 57%. $[\alpha]_D^{20}$ = +11.70 (*c* 1.000 $CHCl_3$) for 92:8 er. ^1H NMR (500 MHz, Chloroform-*d*) δ 8.35 (d, *J* = 1.9 Hz, 1H), 8.21 (d, *J* = 7.8 Hz, 1H), 8.14 (d, *J* = 8.1 Hz, 1H), 8.03 (d, *J* = 8.3 Hz, 1H), 7.69 (dd, *J* = 8.2, 1.6 Hz, 1H), 7.67 – 7.60 (m, 3H), 7.60 – 7.56 (m, 2H), 7.51 – 7.44 (m, 2H), 7.44 – 7.41 (m, 2H), 7.33 – 7.27 (m, 2H), 7.25 (d, *J* = 4.5 Hz, 1H), 7.19 (d, *J* = 1.5 Hz, 1H), 6.94 (d, *J* = 1.8 Hz, 1H), 6.28 (d, *J* = 4.4 Hz, 1H), 3.85 (q, *J* = 7.1 Hz, 2H), 2.40 (q, *J* = 7.5 Hz, 2H), 1.17 (t, *J* = 7.6 Hz, 3H), 0.73 (t, *J* = 7.1 Hz, 3H). ^{13}C NMR (126 MHz, Chloroform-*d*) δ 158.9, 143.6, 141.8, 141.8, 141.4, 140.5, 137.6, 133.5, 132.3, 130.0, 127.6, 127.1, 126.2, 125.9, 123.9, 123.4, 122.1, 121.6, 121.3, 121.3, 120.8, 120.5, 120.1, 119.6, 119.2, 118.2, 110.1, 110.0, 108.6, 106.8, 105.6, 59.9, 18.7, 13.5, 12.5. HRMS (m/z, ESI): Calcd. for Chemical Formula: $C_{39}H_{31}ClN_3O_2^+$ $[M+H]^+$: 608.2099, Found: 608.2101. HPLC analysis of the reaction product: Daicel Chiralpak IC, hexane/*iso*-propanol = 99:1, 1.0 mL/min, λ= 327 nm, retention time: 12.55 min (major) and 14.56 min (minor).

General procedure for Suzuki cross-coupling desymmetrization reactions of N–N carbazole ester substrates.

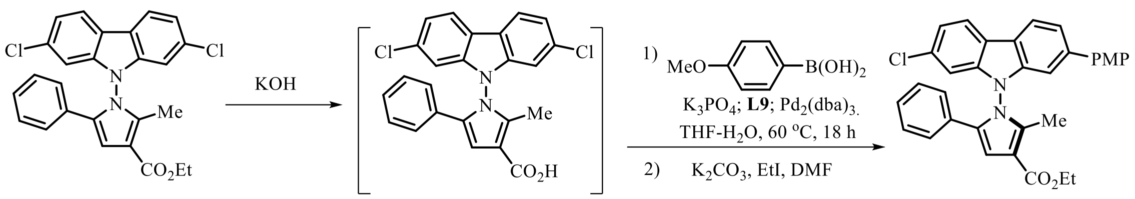

Under the N_2 atmosphere, ethyl 1-(2,7-dichloro-9*H*-carbazol-9-yl)-2-methyl-5-phenyl-1*H*-pyrrole-3-carboxylate (115.5 mg, 0.25 mmol) was dissolved in 1.0 mL THF, and 2.0 M KOH $_{(aq.)}$ (0.65 mL). The resulting reaction mixture was heated to 75 °C and stirred overnight. After cooling to room temperature, the reaction mixture was concentrated and dried in a vacuum. The crude materials were gone to the next step without further purification. Under N_2 atmosphere, a mixture of $Pd_2(dba)_3$ (2.3 mg, 0.0025 mmol) and **L9** (3.5 mg, 0.005 mmol) was added to 0.5 mL THF, and the mixture was stirred at room temperature for 20 min. The resulting metal-ligand complex solution was added to a reaction flask containing 4.5 mL THF solution of the above crude product mixture and *p*-methoxyphenylboronic acid (53.2 mg, 0.35 mmol), and K_3PO_4 (265 mg, 1.25 mmol), followed by addition of 0.10 mL H_2O. Then the resulting reaction mixture was stirred at 60 °C for 18 h. The reaction was then quenched with water, neutralized to pH 3 – 5 with 1.0 M HCl $_{(aq.)}$, and extracted with ethyl acetate (4 mL X 3) three times. The combined organic phases were washed with brine, dried over Na_2SO_4, and concentrated in vacuo. After that, the obtained crude product and K_2CO_3 (1.0 mmol) were added to DMF (1.0 mL), then treated with EtI or MeI (0.5 mmol). The reaction mixture was stirred overnight at room temperature. Then the mixture was diluted with ethyl acetate (20 mL) and washed with water. The organic layer was concentrated in vacuo and purified by flash column chromatography with hexane and acetone as the eluents to give the desired product.

Characterization data of desymmetrizating products from N–N carbazole ester substrates

Ethyl (*S*)-1-(2-chloro-7-(4-methoxyphenyl)-9*H*-carbazol-9-yl)-2-methyl-5-phenyl-1*H*-pyrrole-3-carboxylate (49)

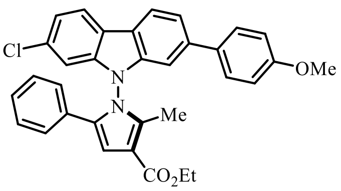

White solid, 80 mg, 60%. $[\alpha]_D^{20}$ = +5.10 (*c* 1.000 $CHCl_3$) for 96:4 er. ^{1}H NMR (500 MHz, Chloroform-*d*) δ 8.05 (d, *J* = 8.1 Hz, 1H), 7.97 (d, *J* = 8.3 Hz, 1H), 7.55 – 7.50 (m, 3H), 7.27 (dd, *J* = 8.5, 2.0 Hz, 1H), 7.19 – 7.13 (m, 3H), 7.09 – 7.05 (m, 3H), 7.03 (d, *J* = 1.8 Hz, 1H), 7.01 – 6.96 (m, 3H), 4.38 (q, *J* = 7.1 Hz, 2H), 3.85 (s, 3H), 2.12 (s, 3H), 1.42 (t, *J* = 7.1 Hz, 3H).^{13}C NMR (126 MHz, Chloroform-*d*) δ 164.8, 159.5, 141.1, 141.0, 140.7, 138.3, 133.9, 133.3, 132.7, 130.0, 128.6, 128.5, 127.6, 126.6, 122.2, 121.5, 121.1, 121.0, 120.0, 119.5, 114.3, 112.6, 108.8, 108.5, 106.5, 60.1, 55.4, 14.5, 10.3. HRMS (m/z, ESI): Calcd. for Chemical Formula: $C_{33}H_{28}ClN_2O_3^+$ $[M+H]^+$: 535.1783, Found: 535.1783. HPLC analysis of the reaction product: Daicel Chiralpak IC, hexane/*iso*-propanol = 99:1, 1.0 mL/min, λ= 315 nm, retention time: 10.93 min (major) and 13.36 min (minor).

Ethyl (*S*)-1-(2-chloro-7-(4-methoxyphenyl)-9*H*-carbazol-9-yl)-2-methyl-5-(naphthalen-2-yl)-1*H*-pyrrole-3-carboxylate (50)

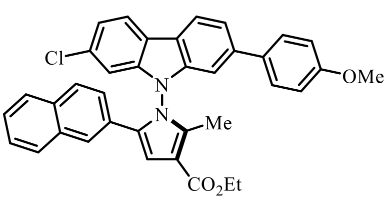

White solid, 76 mg, 52% yield, $[\alpha]_D^{20}$ = +66.80 (*c* 1.000 $CHCl_3$) for 94.5:5.5 er. ^{1}H NMR (500 MHz, Chloroform-*d*) δ 8.06 (d, *J* = 8.1 Hz, 1H), 7.97 (d, *J* = 8.3 Hz, 1H), 7.64 – 7.61 (m, 1H), 7.57 – 7.50 (m, 5H), 7.41 – 7.37 (m, 1H), 7.35 – 7.29 (m, 3H), 7.29 – 7.26 (m, 1H), 7.23 (d, *J* = 1.5 Hz, 1H), 7.13 (s, 1H), 7.07 (d, *J* = 1.8 Hz, 1H), 6.99 – 6.95 (m, 2H), 4.40 (q, *J* = 7.1 Hz,

2H), 3.84 (s, 3H), 2.18 (s, 3H), 1.44 (t, J = 7.1 Hz, 3H). ^{13}C NMR (126 MHz, Chloroform-d) δ 164.8, 159.5, 141.1, 141.0, 140.7, 138.7, 133.8, 133.4, 133.1, 132.8, 132.4, 128.5, 128.3, 128.1, 127.4, 127.3, 126.2, 126.1, 125.3, 124.6, 122.2, 121.6, 121.2, 121.0, 120.0, 119.6, 114.3, 112.7, 109.0, 108.8, 106.5, 60.1, 55.4, 14.6, 10.3. HRMS (m/z, ESI): Calcd. for Chemical Formula: $C_{37}H_{30}ClN_2O_3^+$ [M+H]$^+$: 585.1939, Found: 585.1932. HPLC analysis of the reaction product: Daicel Chiralpak IC, hexane/*iso*-propanol = 99:1, 0.8 mL/min, λ= 325 nm, retention time: 18.48 min (major) and 26.22 min (minor).

Ethyl (*S*)-1-(2-chloro-7-(4-methoxyphenyl)-9*H*-carbazol-9-yl)-5-(3-methoxyphenyl)-2-methyl-1*H*-pyrrole-3-carboxylate (51)

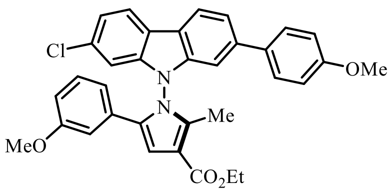

White solid, 63 mg, 45% yield, $[\alpha]_D^{20}$ = +1.25 (c 1.000 $CHCl_3$) for 97:3 er. ^{1}H NMR (500 MHz, Chloroform-d) δ 8.05 (d, J = 8.1 Hz, 1H), 7.97 (d, J = 8.3 Hz, 1H), 7.55 – 7.50 (m, 3H), 7.29 – 7.26 (m, 1H), 7.19 (d, J = 1.5 Hz, 1H), 7.04 – 6.95 (m, 5H), 6.82 – 6.80 (m, 1H), 6.62 – 6.56 (m, 2H), 4.38 (q, J = 7.1 Hz, 2H), 3.85 (s, 3H), 3.26 (s, 3H), 2.17 (s, 3H), 1.42 (t, J = 7.1 Hz, 3H). ^{13}C NMR (126 MHz, Chloroform-d) δ 164.8, 159.5, 159.4, 141.1, 140.9, 140.7, 138.5, 133.7, 133.3, 132.8, 131.1, 129.6, 128.5, 122.2, 121.5, 121.1, 121.0, 119.9, 119.5, 119.1, 114.5, 114.4, 112.5, 110.6, 108.8, 108.7, 106.4, 60.1, 55.4, 54.6, 14.5, 10.3. HRMS (m/z, ESI): Calcd. for Chemical Formula: $C_{34}H_{30}ClN_2O_4^+$ [M+H]$^+$: 565.1889, Found: 565.1894. HPLC analysis of the reaction product: Daicel Chiralpak IC, hexane/*iso*-propanol = 99:1, 1.0 mL/min, λ= 312nm, retention time: 16.03 min (major) and 24.65 min (minor).

Ethyl (*S*)-1-(2-chloro-7-(4-methoxyphenyl)-9*H*-carbazol-9-yl)-2-methyl-5-(3-nitrophenyl)-1*H*-pyrrole-3-carboxylate (52)

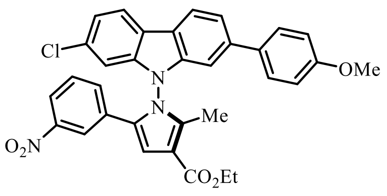

White solid, 81 mg, 56%, $[\alpha]_D^{20}$ = +20.00 (*c* 1.000 $CHCl_3$) for 89.5:10.5 er. ^{1}H NMR (500 MHz, Chloroform-*d*) δ 8.08 (d, *J* = 8.2 Hz, 1H), 8.02 – 7.98 (m, 2H), 7.90 – 7.88 (m, 1H), 7.56 (dd, *J* = 8.2, 1.5 Hz, 1H), 7.53 – 7.48 (m, 2H), 7.36 – 7.34 (m, 1H), 7.31 (dd, *J* = 8.3, 1.8 Hz, 1H), 7.20 (t, *J* = 8.1 Hz, 1H), 7.14 (d, *J* = 1.5 Hz, 2H), 7.01 (d, *J* = 1.8 Hz, 1H), 6.99 – 6.94 (m, 2H), 4.40 (q, *J* = 7.1 Hz, 2H), 3.84 (s, 3H), 2.18 (s, 3H), 1.43 (t, *J* = 7.1 Hz, 3H). ^{13}C NMR (126 MHz, Chloroform-*d*) δ 164.4, 159.6, 148.4, 141.1, 140.8, 140.7, 139.7, 133.2, 133.0, 131.5, 131.4, 131.1, 129.7, 128.5, 122.6, 122.1, 121.8, 121.6, 121.2_9, 121.2_7, 120.2, 119.7, 114.4, 113.1, 110.2, 108.6, 106.2, 60.3, 55.4, 14.5, 10.3. HRMS (m/z, ESI): Calcd. for Chemical Formula: $C_{33}H_{27}ClN_3O_5^+$ $[M+H]^+$: 580.1634, Found: 580.1626. HPLC analysis of the reaction product: Daicel Chiralpak IF, hexane/*iso*-propanol = 99:1, 1.0 mL/min, λ= 325 nm, retention time: 27.53 min (major) and 24.57 min (minor).

Ethyl (*S*)-5-(benzofuran-2-yl)-1-(2-chloro-7-(4-methoxyphenyl)-9*H*-carbazol-9-yl)-2-methyl-1*H*-pyrrole-3-carboxylate (53)

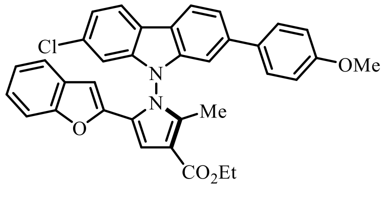

White solid, 73 mg, 51%, $[\alpha]_D^{20}$ = +89.00 (*c* 1.000 $CHCl_3$) for 92:8 er. ^{1}H NMR (400 MHz, Chloroform-*d*) δ 8.17 (d, *J* = 8.2 Hz, 1H), 8.09 (d, *J* = 8.3 Hz, 1H), 7.61 (dd, *J* = 8.1, 1.6 Hz, 1H), 7.54 – 7.50 (m, 2H), 7.39 (s, 1H), 7.37 – 7.32 (m, 2H), 7.19 – 7.12 (m, 3H), 7.07 – 7.00 (m, 2H), 6.97 – 6.91 (m, 2H), 5.43 (s, 1H), 4.41 (q, *J* = 7.1 Hz, 2H), 3.83 (s, 3H), 2.21 (s, 3H), 1.44 (t, *J* = 7.1 Hz, 3H). ^{13}C NMR (101 MHz, Chloroform-*d*) δ 164.4, 159.5, 153.8, 145.9, 140.9, 140.4,

140.2, 139.6, 133.2, 133.0, 128.6, 124.5, 124.4, 122.9, 122.6, 121.7, 121.5, 121.1, 120.9, 120.1, 119.6, 114.3, 113.1, 110.8, 109.3, 108.9, 106.4, 100.4, 60.2, 55.4, 14.5, 10.1. HRMS (m/z, ESI): Calcd. for Chemical Formula: $C_{35}H_{28}ClN_2O_4^+$ $[M+H]^+$: 575.1732, Found: 575.1730. HPLC analysis of the reaction product: Daicel Chiralpak ID, hexane/*iso*-propanol = 99:1, 0.6 mL/min, λ= 305 nm, retention time: 17.83 min (major) and 16.48 min (minor).

Ethyl (*S*)-1-(2-chloro-7-(4-methoxyphenyl)-9*H*-carbazol-9-yl)-2-methyl-5-(thiophen-2-yl)-1*H*-pyrrole-3-carboxylate (54)

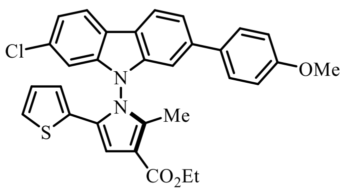

White solid, 58 mg, 44% yield, $[\alpha]_D^{20} = -14.5$ (*c* 1.000 $CHCl_3$) for 96.5:3.5 er. ^{1}H NMR (500 MHz, Chloroform-*d*) δ 8.10 (d, *J* = 8.1 Hz, 1H), 8.02 (d, *J* = 8.3 Hz, 1H), 7.57 (dd, *J* = 8.2, 1.5 Hz, 1H), 7.54 – 7.50 (m, 2H), 7.31 (dd, *J* = 8.3, 1.8 Hz, 1H), 7.15 (d, *J* = 1.5 Hz, 1H), 7.05 (s, 1H), 7.02 (d, *J* = 1.8 Hz, 1H), 6.98 – 6.94 (m, 3H), 6.71 (dd, *J* = 5.1, 3.7 Hz, 1H), 6.62 (dd, *J* = 3.7, 1.2 Hz, 1H), 4.38 (q, *J* = 7.2 Hz, 2H), 3.84 (s, 3H), 2.14 (s, 3H), 1.42 (t, *J* = 7.1 Hz, 3H). ^{13}C NMR (126 MHz, Chloroform-*d*) δ 164.6, 159.5, 140.8, 140.7, 138.1, 133.3, 132.8, 131.0, 128.5, 127.7, 127.3, 124.7, 123.8, 122.4, 121.6, 121.3, 121.0, 120.2, 119.7, 114.3, 112.6, 108.9, 107.8, 106.5, 60.1, 55.4, 14.5, 10.2. HRMS (m/z, ESI): Calcd. for Chemical Formula: $C_{31}H_{26}ClN_2O_3S^+$ $[M+H]^+$: 541.1347, Found: 541.1346. HPLC analysis of the reaction product: Daicel Chiralpak IB, hexane/*iso*-propanol = 99:1, 1.0 mL/min, λ= 312 nm, retention time: 7.68 min (major) and 10.33 min (minor).

Ethyl (*R*)-1-(2-chloro-7-(4-methoxyphenyl)-9*H*-carbazol-9-yl)-5-cyclopropyl-2-methyl-1*H*-pyrrole-3-carboxylate (55)

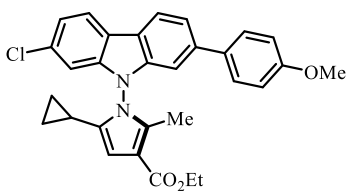

White solid, 75 mg, 56%, $[\alpha]_D^{20}$ = −12.25 (*c* 1.000 $CHCl_3$) for 82:18 er. ^{1}H NMR (500 MHz, Chloroform-*d*) δ 8.10 (d, *J* = 8.1 Hz, 1H), 8.02 (d, *J* = 8.3 Hz, 1H), 7.59 – 7.51 (m, 3H), 7.32 (dd, *J* = 8.3, 1.9 Hz, 1H), 7.14 (d, *J* = 1.6 Hz, 1H), 7.04 – 6.95 (m, 3H), 6.36 (s, 1H), 4.34 (q, *J* = 7.1 Hz, 2H), 3.86 (s, 3H), 2.20 (s, 3H), 1.39 (t, *J* = 7.1 Hz, 3H), 1.17 – 1.11 (m, 1H), 0.55 – 0.41 (m, 4H). ^{13}C NMR (126 MHz, Chloroform-*d*) δ 165.1, 159.5, 141.1, 141.0, 140.5, 136.6, 136.0, 133.4, 132.6, 128.5, 122.0, 121.5, 121.0, 120.9, 120.1, 119.6, 114.3, 110.9, 108.8, 106.4, 104.1, 59.8, 55.4, 14.6, 10.3, 6.2, 6.2, 5.6. HRMS (m/z, ESI): Calcd. for Chemical Formula: $C_{30}H_{28}ClN_2O_3^+$ $[M+H]^+$: 499.1783, Found: 499.1785. The ester product was provided for the HPLC analysis of the reaction product: Daicel Chiralpak IC and IC, hexane/*iso*-propanol = 99: 1, 1.0 mL/min, λ= 314 nm, retention time: 25.57 min (major) and 28.61 min (minor).

Ethyl (*S*)-1-(2-chloro-7-(4-methoxyphenyl)-9*H*-carbazol-9-yl)-2-ethyl-5-phenyl-1*H*-pyrrole-3-carboxylate (56)

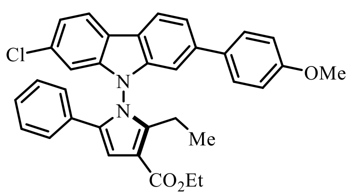

White solid, 65 mg, 47%, $[\alpha]_D^{20}$ = +9.80 (*c* 1.000 $CHCl_3$) for 96.5:3.5 er. ^{1}H NMR (500 MHz, Chloroform-*d*) δ 8.04 (d, *J* = 8.2 Hz, 1H), 7.96 (d, *J* = 8.3 Hz, 1H), 7.54 – 7.49 (m, 3H), 7.28 – 7.26 (m, 1H), 7.18 (d, *J* = 1.6 Hz, 1H), 7.10 – 6.95 (m, 9H), 4.39 (q, *J* = 7.1 Hz, 2H), 3.85 (s, 3H), 2.62 – 2.49 (m, 2H), 1.42 (t, *J* = 7.1 Hz, 3H), 0.92 (t, *J* = 7.4 Hz, 3H). ^{13}C NMR (126 MHz, Chloroform-*d*) δ 164.5, 159.5, 144.4, 141.4, 141.3, 140.5, 133.8, 133.4, 132.6, 130.0, 128.5, 127.6, 126.7, 122.1, 121.5, 121.0, 120.9, 119.8, 119.4, 114.3, 112.0, 108.9_2, 108.8_7, 106.6, 60.0, 55.4, 18.4, 14.5, 14.2. HRMS (m/z, ESI): Calcd. for Chemical Formula: $C_{34}H_{30}ClN_2O_3^+$ $[M+H]^+$: 549.1939, Found:

549.1938. HPLC analysis of the reaction product: Daicel Chiralpak IC, hexane/*iso*-propanol = 99: 1, 1.0 mL/min, λ= 332 nm, retention time: 8.90 min (major) and 11.37 min (minor).

Methyl (*S*)-2-butyl-1-(2-chloro-7-(4-methoxyphenyl)-9*H*-carbazol-9-yl)-5-phenyl-1*H*-pyrrole-3-carboxylate (57)

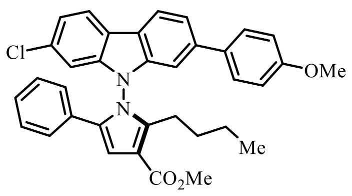

White solid, 62 mg, 43%, $[\alpha]_D^{20}$ = +17.50 (*c* 1.000 $CHCl_3$) for 96:4 er. ^{1}H NMR (500 MHz, Chloroform-*d*) δ 8.04 (d, *J* = 8.2 Hz, 1H), 7.96 (d, *J* = 8.3 Hz, 1H), 7.54 – 7.48 (m, 3H), 7.27 (d, *J* = 7.5 Hz, 1H), 7.16 (d, *J* = 1.6 Hz, 1H), 7.08 (dd, *J* = 7.5, 2.2 Hz, 2H), 7.05 – 7.01 (m, 4H), 6.98 (d, *J* = 9.0 Hz, 3H), 3.91 (s, 3H), 3.85 (s, 3H), 2.60 – 2.52 (m, 1H), 2.51 – 2.42 (m, 1H), 1.31 – 1.26 (m, 2H), 1.06 – 0.97 (m, 2H), 0.51 (t, *J* = 7.3 Hz, 3H). ^{13}C NMR (126 MHz, Chloroform-*d*) δ 165.0, 159.4, 143.5, 141.4, 141.3, 140.4, 133.9, 133.4, 132.6, 130.0, 128.5, 128.5, 127.6, 126.7, 122.1, 121.5, 121.1, 120.9, 119.9, 119.4, 114.3, 112.0, 109.0, 108.7, 106.7, 55.4, 51.2, 31.5, 24.5, 22.3, 13.2. HRMS (m/z, ESI): Calcd. for Chemical Formula: $C_{35}H_{32}ClN_2O_3^+$ $[M+H]^+$: 563.2096, Found: 563.2089. HPLC analysis of the reaction product: Daicel Chiralpak IB, hexane/*iso*-propanol = 99:1, 0.8 mL/min, λ= 333 nm, retention time: 9.10 min (major) and 10.08 min (minor).

Buchwald-hartwig amination and Sonogashira cross-coupling desymmetrization reaction

Ethyl (*R*)-2-(2-chloro-7-((4-methoxyphenyl)amino)-9*H*-carbazol-9-yl)-3-methylbenzoate (21)

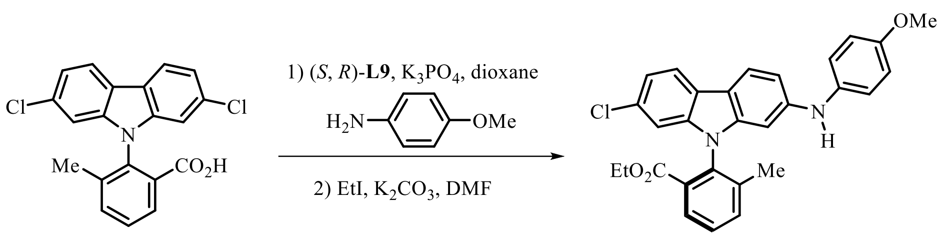

Under N_2 atmosphere, a mixture of $Pd_2(dba)_3$ (2.3 mg, 0.0025 mmol) and **L9** (3.5 mg, 0.005 mmol) was added to 0.5 mL dioxane, and the mixture was stirred at room temperature for 20 min. The resulting metal-ligand complex solution was added to a reaction flask containing 4.5 mL dioxane solution of 2-(2,7-dichloro-9*H*-carbazol-9-yl)-3-methylbenzoic acid **1** (92.5 mg, 0.25 mmol) and *p*-Anisidine (40 mg, 0.33 mmol), and K_3PO_4 (265 mg, 1.25 mmol). Then the resulting reaction mixture was stirred at 100 °C for 18 h. The reaction was then quenched with water, neutralized to pH 5 – 6 with 1.0 M HCl $_{(aq.)}$, and extracted with ethyl acetate (4 mL X 3) three times. The combined organic phases were washed with brine, dried over Na_2SO_4, and concentrated in vacuo. After that, the obtained crude product and K_2CO_3 (138 mg, 1.0 mmol) were added to DMF (1.0 mL), then treated with EtI (78 mg, 0.5 mmol). The reaction mixture was stirred overnight at room temperature. Then the mixture was diluted with ethyl acetate (20 mL) and washed with water. The organic layer was concentrated in vacuo and purified by flash column chromatography with hexane and acetone (50:1 – 10:1) to give the desired product, reddish solid, 50 mg, 41%, $[\alpha]_D^{20}$ = +46.00 (*c* 1.000 $CHCl_3$) for 99.5:0.5 er ^1H NMR (500 MHz, Chloroform-*d*) δ 7.99 – 7.81 (m, 3H), 7.60 – 7.60 (m, 1H), 7.52 (t, *J* = 7.7 Hz, 1H), 7.21 – 6.76 (m, 7H), 6.44 (s, 1H), 3.84 – 3.69 (m, 5H), 2.02 (s, 3H), 0.51 (t, *J* = 7.1 Hz, 3H). ^{13}C NMR (126 MHz, Chloroform-*d*) δ 166.1, 139.3, 135.0, 134.2, 131.9, 129.6, 129.0, 121.2, 119.9, 114.7, 109.2, 61.1, 55.6, 17.6, 13.0. HRMS (m/z, ESI): Calcd. for Chemical Formula: $C_{29}H_{26}ClN_2O_3^+$ $[M+H]^+$: 485.1626, Found: 485.1625. HPLC analysis of the reaction product: Daicel Chiralpak ID, hexane/iso-propanol = 97:3, 1.0 mL/min, λ= 344 nm, retention time: 15.33 min (major)

and 13.20 min (minor).

Ethyl (*S*)1-(2-chloro-7-((4-methoxyphenyl)ethynyl)-9*H*-carbazol-9-yl)-2-methyl-5-phenyl-1*H*-pyrrole-3-carboxylate (58)

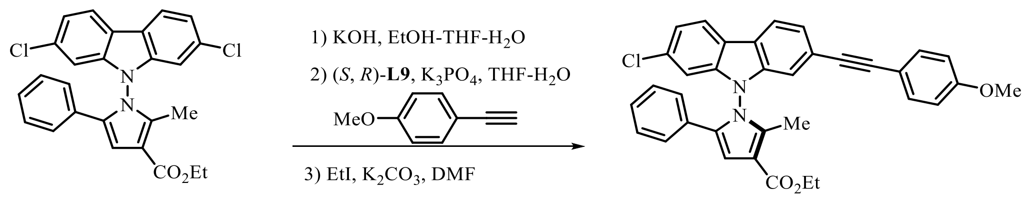

Under the N_2 atmosphere, ethyl 1-(2,7-dichloro-9*H*-carbazol-9-yl)-2-methyl-5-phenyl-1*H*-pyrrole-3-carboxylate (115.5 mg, 0.25 mmol) was dissolved in 1.0 mL THF, and 2.0 M KOH $_{(aq.)}$ (0.65 mL). The resulting reaction mixture was heated to 75 °C and stirred overnight. After cooling to room temperature, the reaction mixture was concentrated and dried in a vacuum. The crude acid **68** was gone to the next step without further purification. Under N_2 atmosphere, a mixture of $Pd_2(dba)_3$ (2.3 mg, 0.0025 mmol) and **L9** (3.5 mg, 0.005 mmol) was added to 0.5 mL THF, and the mixture was stirred at room temperature for 20 min. The resulting metal-ligand complex solution was added to a reaction flask containing 4.5 mL THF solution of the above crude product mixture and 4-Ethynylanisole (46.2 mg, 0.35 mmol), and K_3PO_4 (265 mg, 1.25 mmol), followed by addition of 0.10 mL H_2O. Then the resulting reaction mixture was stirred at 60 °C for 24 h. The reaction was then quenched with water, neutralized to pH 3 – 5 with 1.0 M HCl $_{(aq.)}$, and extracted with ethyl acetate (4 mL X 3) three times. The combined organic phases were washed with brine, dried over Na_2SO_4, and concentrated in vacuo. After that, the obtained crude product and K_2CO_3 (138 mg, 1.0 mmol) were added to DMF (1.0 mL), then treated with EtI (78 mg, 0.5 mmol). The reaction mixture was stirred overnight at room temperature. Then the mixture was diluted with ethyl acetate (20 mL) and washed with water. The organic layer was concentrated in vacuo and purified by flash column

chromatography with hexane and acetone (50:1 – 10:1) to give the desired product, white solid, 85 mg, 61%, $[\alpha]_D^{20}$ = +63.10 (*c* 1.000 $CHCl_3$) for 95:5 er ^1H NMR (500 MHz, Chloroform-*d*) δ 7.99 (d, *J* = 8.1 Hz, 1H), 7.95 (d, *J* = 8.3 Hz, 1H), 7.50 – 7.45 (m, 3H), 7.29 – 7.26 (m, 1H), 7.21 (d, *J* = 1.3 Hz, 1H), 7.13 (dd, *J* = 7.4, 2.4 Hz, 2H), 7.07 (dd, *J* = 5.3, 2.1 Hz, 3H), 7.02 – 6.96 (m, 2H), 6.90 – 6.85 (m, 2H), 4.38 (q, *J* = 7.1 Hz, 2H), 3.83 (s, 3H), 2.09 (s, 3H), 1.41 (t, *J* = 7.1 Hz, 3H). ^{13}C NMR (126 MHz, Chloroform-*d*) δ 164.8, 159.8, 141.1, 140.2, 138.3, 133.8, 133.3, 133.1, 129.8, 128.6, 127.7, 126.6, 125.5, 122.5, 122.4, 121.8, 120.7, 120.5, 119.8, 115.0, 114.1, 112.7, 111.5, 108.9, 108.5, 90.5, 88.4, 60.1, 55.3, 14.5, 10.2. HRMS (m/z, ESI): Calcd. for Chemical Formula: $C_{35}H_{28}ClN_2O_3^+$ $[M+H]^+$: 559.1783, Found: 559.1783. HPLC analysis of the reaction product: Daicel Chiralpak IB, hexane/iso-propanol = 99:1, 0.8 mL/min, λ= 346 nm, retention time: 11.02 min (major) and 12.13 min (minor).

Ethyl (*R*)-5-cyclopropyl-1-(2-(4-methoxyphenyl)-7-((4-methoxyphenyl)ethynyl)-9*H*-carbazol-9-yl)-2-methyl-1*H*-pyrrole-3-carboxylate (59)

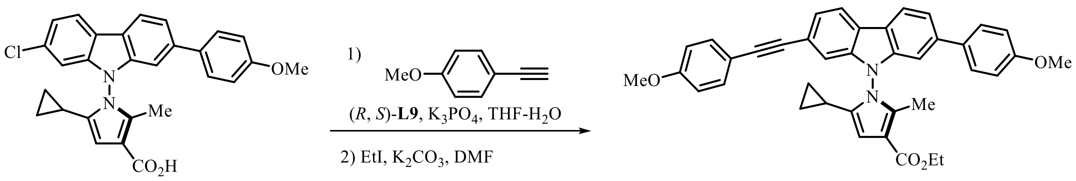

(**55**) 82:18 er (**59**) 68% 88.5:11.5 er

Under N_2 atmosphere, a mixture of $Pd_2(dba)_3$ (0.92 mg, 0.001 mmol) and *ent*-**L9** (1.4 mg, 0.002 mmol) was added to 0.3 mL THF, and the mixture was stirred at room temperature for 20 min. The resulting metal-ligand complex solution was added to a reaction flask containing 1.7 mL THF solution of (*R*)-1-(2-chloro-7-(4-methoxyphenyl)-9*H*-carbazol-9-yl)-5-cyclopropyl-2-methyl-1*H*-pyrrole-3-carbo-xylic acid (47 mg, 0.1 mmol) and 4-Ethynylanisole (10.6 mg, 0.08 mmol), and

K_3PO_4 (106 mg, 0.5 mmol), followed by addition of 0.04 mL H_2O. Then the resulting reaction mixture was stirred at 60 °C for 24 h. The reaction was then quenched with water, neutralized to pH 3 – 5 with 1.0 M HCl $_{(aq.)}$, and extracted with ethyl acetate (4 mL X 3) three times. The combined organic phases were washed with brine, dried over Na_2SO_4, and concentrated in vacuo. After that, the obtained crude product and K_2CO_3 (138 mg, 1.0 mmol) were added to DMF (1.0 mL), then treated with EtI (78 mg, 0.5 mmol). The reaction mixture was stirred overnight at room temperature. Then the mixture was diluted with ethyl acetate (20 mL) and washed with water. The organic layer was concentrated in vacuo and purified by flash column chromatography with hexane and acetone (50:1 – 10:1) to give the desired product, Colorless oil, 40.5 mg, 68%, $[\alpha]_D^{20}$ = +9.90 (*c* 0.500 $CHCl_3$) for 88.5:11.5 er. ^{1}H NMR (500 MHz, Chloroform-*d*) δ 8.12 (d, *J* = 8.1 Hz, 1H), 8.07 (d, *J* = 8.1 Hz, 1H), 7.58 – 7.54 (m, 3H), 7.52 – 7.46 (m, 3H), 7.16 (dd, *J* = 7.8, 1.4 Hz, 2H), 7.01 – 6.96 (m, 2H), 6.92 – 6.86 (m, 2H), 6.36 (d, *J* = 1.0 Hz, 1H), 4.33 (d, *J* = 7.1 Hz, 2H), 3.86 (s, 3H), 3.83 (s, 3H), 2.22 (s, 3H), 1.39 (t, *J* = 7.1 Hz, 3H), 1.19 – 1.14 (m, 1H), 0.59 – 0.39 (m, 4H). ^{13}C NMR (126 MHz, Chloroform-*d*) δ 165.1, 159.7, 159.4, 141.3, 140.5, 140.2, 136.8, 136.2, 133.5, 133.1, 128.5, 125.0, 121.7, 121.1, 121.1, 120.8, 120.5, 120.0, 115.2, 114.3, 114.1, 111.3, 110.7, 106.3, 104.0, 90.0, 88.7, 59.8, 55.4, 55.3, 14.6, 10.3, 6.3, 6.1, 5.6. HRMS (m/z, ESI): Calcd. for Chemical Formula: $C_{39}H_{35}N_2O_4^+$ $[M+H]^+$: 595.2591, Found: 595.2595. HPLC analysis of the reaction product: Daicel Chiralpak ADH, hexane/*iso*-propanol = 99:1, 1.0 mL/min, λ= 342 nm, retention time: 33.48 min (major) and 26.75 min (minor).

Suzuki cross-coupling desymmetrization reactions of ethyl ester substrates.

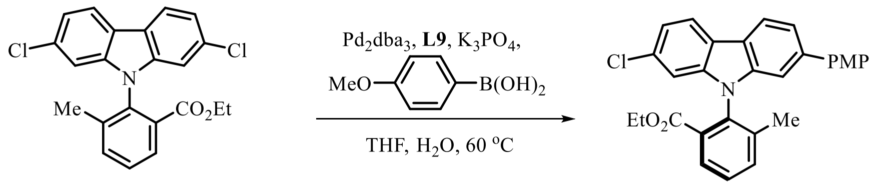

(SI-**1**) (**2**) 56% Conv., 55:45 er

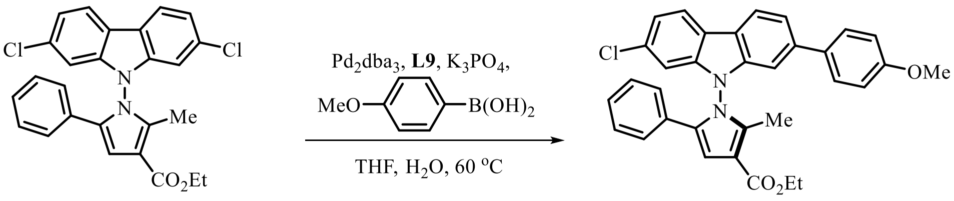

(SI-**4**) (**49**) 52% conv., 51:49 er

The procedure was the same as the procedure of the Suzuki cross-coupling desymmetrization reaction. The conversion was determined by the crude ^1H NMR.

Racemization of N–C and N–N carbazole products.

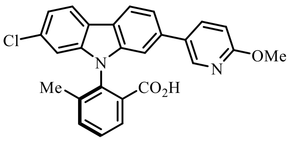

11, 1.5:98.5 er with methyl ester (60 °C)

Temperature (°C, 24 h)	er value
80	1.5:98.5
100	1.5:98.5
120	1.5:98.5

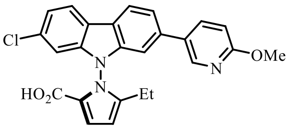

42, 98.5:1.5 er with ethyl ester (60 °C)

Temperature (°C, 24 h)	er value
80	98.5:1.5
100	98.5:1.5
120	98.5:1.5

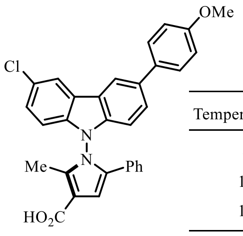

61, 83.5:16.5 er with ethyl ester (60 °C)

Temperature (°C, 24 h)	er value
80	83.5:16.5
100	83.5:16.5
120	83.5:16.5

Each enantioenriched sample was dissolved in toluene and then heated up at the specified temperature (80, 100, and 120 °C) for 24 hours. No racemization was observed in all cases. Therefore, the erosion of enantipurity is unlikely under the typical reaction conditions (60 °C).

Suzuki cross-coupling desymmetrization reactions of 2-(3,6-dichloro-9*H*-carbazol-9-yl)-3-methylbenzoic acid

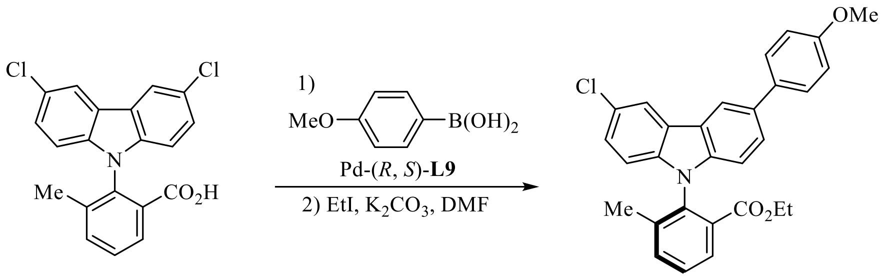

(SI-**3**) (SI-**10**), 51%, 69.5:30.5 er

The procedure was the same as the procedure of the Suzuki cross-coupling desymmetrization reaction. (SI-**3**) was 0.1 mmol, *p*-methoxyphenylboronic acid was 0.14 mmol. Isolated yield as ethyl ester reported.

Ethyl (*S*)-2-(3-chloro-6-(4-methoxyphenyl)-9*H*-carbazol-9-yl)-3-methylbenzoate (SI-10)

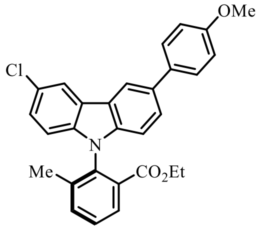

White solid, 24 mg, 51%. $[\alpha]_D^{20}$ = -5.60 (*c* 1.000 $CHCl_3$) for 69.5:30.5 er. ^1H NMR (500 MHz, Chloroform-*d*) δ 8.25 (d, *J* = 1.8 Hz, 1H), 8.14 (d, *J* = 2.0 Hz, 1H), 7.95 (dd, *J* = 7.8, 1.6 Hz, 1H), 7.66 – 7.62 (m, 3H), 7.60 – 7.54 (m, 2H), 7.32 (dd, *J* = 8.6, 2.1 Hz, 1H), 7.04 – 7.01 (m, 2H), 6.96 (d, *J* = 8.5 Hz, 1H), 6.87 (d, *J* = 8.6 Hz, 1H), 3.88 (s, 3H), 3.68 (q, *J* = 7.1 Hz, 2H), 2.01 (s, 3H), 0.47 (t, *J* = 7.1 Hz, 3H). ^{13}C NMR (126 MHz, Chloroform-*d*) δ 166.1, 158.7, 141.0, 140.0, 139.4, 135.0, 134.5, 134.4, 133.2, 132.1, 129.6, 129.1, 128.3, 126.1, 126.0, 125.1, 124.4, 122.7, 120.1, 118.5, 114.3, 114.2, 110.6, 109.9, 61.1, 55.4, 17.6, 13.0. HRMS (m/z, ESI): Calcd. for Chemical Formula: $C_{29}H_{25}ClNO_3^+$ $[M+H]^+$: 470.1517, Found: 470.1517. HPLC analysis of the reaction product: Daicel Chiralpak IA, hexane/*iso*-propanol = 97:3, 1.0 mL/min, λ= 281 nm, retention time: 8.87 min (major) and 8.26 min (minor).

Synthetic utilities

A) (3,6-Dichlorocarbazole)

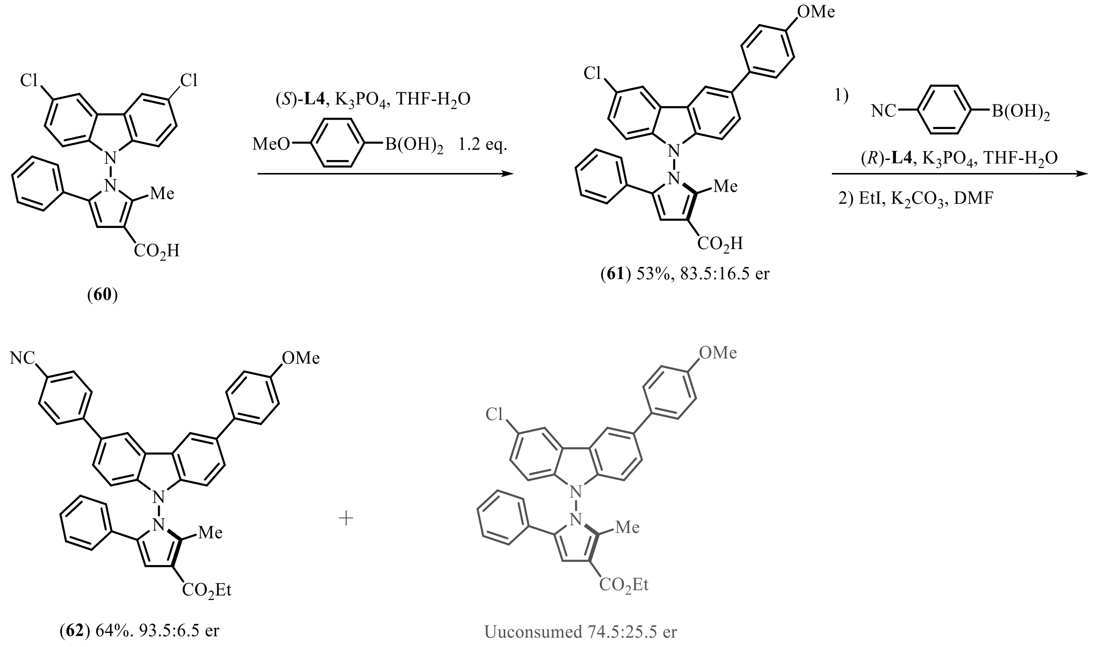

(*S*)-1-(3-Chloro-6-(4-methoxyphenyl)-9*H*-carbazol-9-yl)-2-methyl-5-phenyl-1*H*-pyrrole-3-carboxylic acid (61)

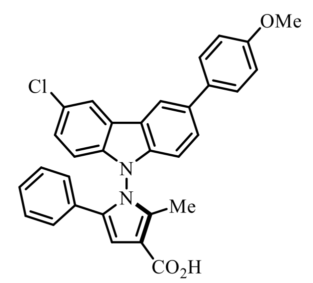

Under N_2 atmosphere, a mixture of $Pd_2(dba)_3$ (2.3 mg, 0.0025 mmol) and (*S*)-**L4** (2.9 mg, 0.005 mmol) was added to 0.5 mL THF, the mixture was then stirred at room temperature for 20 min. The resulting metal-ligand complex solution was added to a reaction flask containing 4.5 mL THF solution of 1-(3,6-dichloro-9*H*-carbazol-9-yl)-2-methyl-5-phenyl-1*H*-pyrrole-3-carboxylic acid **60** (108.5 mg, 0.25 mmol) and *p*-methoxyphenylboronic acid (45.6 mg, 0.30 mmol), and K_3PO_4 (265 mg, 1.25 mmol), followed by addition of 0.10 mL H_2O. Then the resulting reaction mixture was stirred at 60 °C for 18 h. The reaction was then quenched with water, neutralized to pH 3 – 5 with 1.0 M HCl $_{(aq.)}$, and extracted with ethyl acetate (4.0 mL X 3) three times. The combined

organic phase was washed with brine, dried over Na_2SO_4, and concentrated in vacuo. The organic layer was concentrated in vacuo and purified by flash column chromatography with hexane and acetone (50:1 – 5:1) to give the desired acid product. According to ester procedure gave Et-ester product by EtI, K_2CO_3, and DMF. White solid, 71 mg, 53%. $[\alpha]_D^{20}$ = +12.70 (*c* 1.000 $CHCl_3$) for 83.5:16.5 er. ^{1}H NMR (500 MHz, Chloroform-*d*) δ 8.18 (dd, *J* = 1.7, 0.7 Hz, 1H), 8.09 (dd, *J* = 2.1, 0.5 Hz, 1H), 7.63 (dd, *J* = 8.5, 1.7 Hz, 1H), 7.61 – 7.57 (m, 2H), 7.37 (dd, *J* = 8.6, 2.0 Hz, 1H), 7.14 – 7.04 (m, 6H), 7.03 – 7.00 (m, 2H), 6.99 – 6.96 (m, 2H), 4.38 (q, *J* = 7.1 Hz, 2H), 3.87 (s, 3H), 2.09 (s, 3H), 1.41 (t, *J* = 7.1 Hz, 3H). ^{13}C NMR (126 MHz, Chloroform-*d*) δ 164.8, 159.0, 139.6, 138.8, 138.5, 135.1, 133.9, 133.7, 130.0, 128.6, 128.3, 127.6, 127.2, 127.1, 127.0, 126.5, 122.8, 121.0, 120.7, 118.9, 114.4, 112.5, 109.8, 109.1, 108.4, 60.0, 55.4, 14.5, 10.2. HRMS (m/z, ESI): Calcd. for Chemical Formula: $C_{33}H_{28}ClN_2O_3^+$ $[M+H]^+$: 535.1783, Found: 535.1782. HPLC analysis of the reaction product: Daicel Chiralpak IC, hexane/*iso*-propanol = 99:1, 1.0 mL/min, λ= 294 nm, retention time: 20.90 min (major) and 17.02 min (minor).

Ethyl (*R*)-1-(3-(4-cyanophenyl)-6-(4-methoxyphenyl)-9*H*-carbazol-9-yl)-2-methyl-5-phenyl-1*H*-pyrrole-3-carboxylate (62)

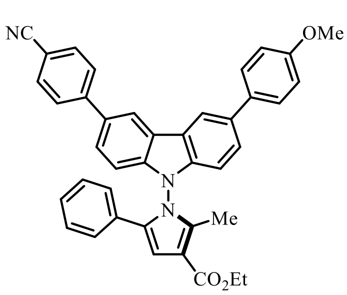

Under N_2 atmosphere, a mixture of $Pd_2(dba)_3$ (1.6 mg, 0.0018 mmol) and (*R*)-**L4** (2.1 mg, 0.0036 mmol) was added to 0.42 mL THF, and the mixture was stirred at room temperature for 20 min. The resulting metal-ligand complex solution was added to a reaction flask containing 1.0 mL THF solution of (*S*)-1-(3-chloro-6-(4-methoxyphenyl)-9*H*-carbazol-9-yl)-2-methyl-5-phenyl-1*H*-pyrrole-3-carboxylic acid **61** (36 mg, 0.071 mmol) and *p*-

cyanophenylboronic acid (8.3 mg, 0.57 mmol), and K_3PO_4 (75 mg, 0.355 mmol), followed by addition of 0.05 mL H_2O. Then the resulting reaction mixture was stirred at 60 °C for 18 h. The reaction was then quenched with water, neutralized to pH 3 – 5 with 1.0 M HCl $_{(aq.)}$, and extracted with ethyl acetate (2 mL X 3) three times. The combined organic phases were washed with brine, dried over Na_2SO_4, and concentrated in vacuo. After that, the obtained crude product and K_2CO_3 (29 mg, 0.213 mmol) were added to DMF (0.5 mL), then treated with EtI (55 mg, 0.355 mmol). The reaction mixture was stirred overnight at room temperature. Then the mixture was diluted with ethyl acetate (20 mL) and washed with water. The organic layer was concentrated in vacuo and purified by flash column chromatography with hexane and acetone (150:1 – 50:1) to give the desired product. White solid, 27 mg, 64%, $[\alpha]_D^{20}$ = −1.30 (*c* 1.000 $CHCl_3$) for 93.5:6.5 er. ^1H NMR (500 MHz, Chloroform-*d*) δ 8.34 (d, *J* = 1.7 Hz, 1H), 8.29 (d, *J* = 1.6 Hz, 1H), 7.80 – 7.73 (m, 4H), 7.66 – 7.60 (m, 4H), 7.19 – 7.12 (m, 4H), 7.09 – 6.98 (m, 6H), 4.39 (q, *J* = 7.1 Hz, 2H), 3.87 (s, 3H), 2.12 (s, 3H), 1.42 (t, *J* = 7.1 Hz, 3H). ^{13}C NMR (126 MHz, Chloroform-*d*) δ 164.9, 159.0, 145.9, 140.5, 139.6, 138.6, 135.2, 133.9, 133.8, 132.8, 132.7, 130.1, 128.6, 128.3, 127.8, 127.6, 126.9, 126.6, 126.5, 122.3, 121.8, 119.7, 119.1, 118.9, 114.4, 112.5, 110.4, 109.4, 109.2, 108.4, 60.1, 55.4, 14.6, 10.2. HRMS (m/z, ESI): Calcd. for Chemical Formula: $C_{40}H_{32}N_3O_3^+$ $[M+H]^+$: 602.2438, Found: 602.2441. HPLC analysis of the reaction product: Daicel Chiralpak IF, hexane/*iso*-propanol = 97:3, 1.0 mL/min, λ= 333 nm, retention time: 43.59 min (major) and 39.60 min (minor).

B) (Sequential coupling of trichloro *N*-aryl-carbazole)

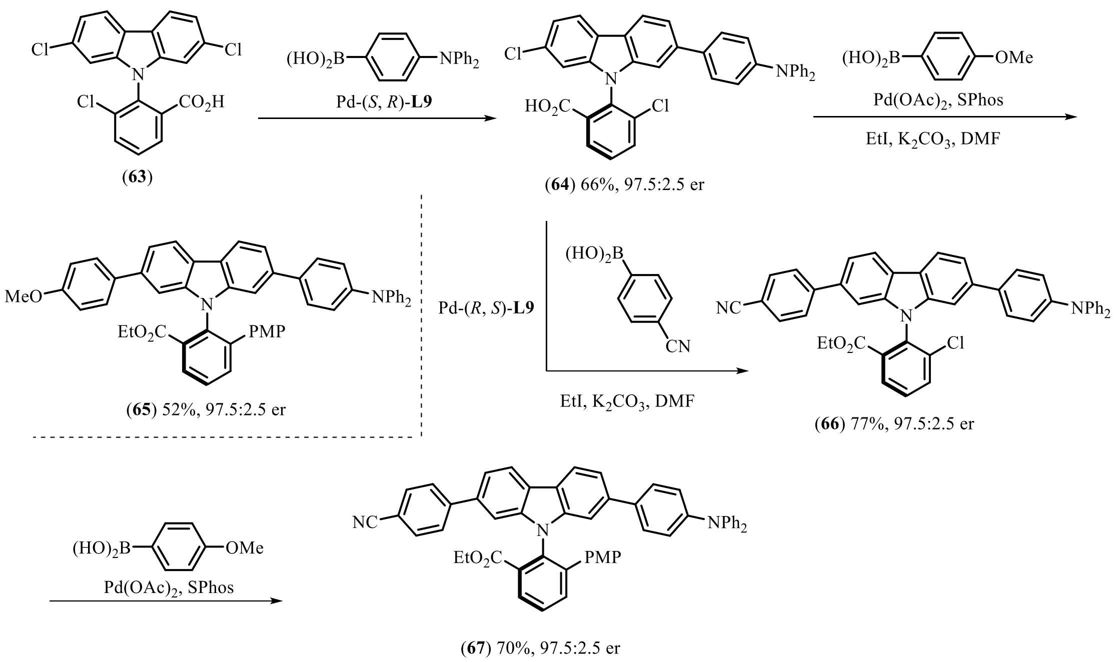

S-3-Chloro-2-(2-chloro-7-(4-(diphenylamino)phenyl)-9*H*-carbazol-9-yl)benzoic acid (64)

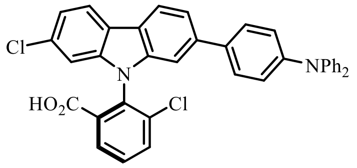

Under N_2 atmosphere, a mixture of $Pd_2(dba)_3$ (2.3 mg, 0.0025 mmol) and (*S*, *R*)-**L9** (3.5 mg, 0.005 mmol) was added to 0.5 mL THF, the mixture was then stirred at room temperature for 20 min. The resulting metal-ligand complex solution was added to a reaction flask containing 4.5 mL THF solution of 3-chloro-2-(2,7-dichloro-9*H*-carbazol-9-yl)benzoic acid **63** (97.3 mg, 0.25 mmol) and (4-(diphenylamino)phenyl)boronic acid (101.2 mg, 0.35 mmol), and K_3PO_4 (265 mg, 1.25 mmol), followed by addition of 0.10 mL H_2O. Then the resulting reaction mixture was stirred at 60 °C for 18 h. The reaction was then quenched with water, neutralized to pH 3 – 5 with 1.0 M HCl $_{(aq.)}$, and extracted with ethyl acetate (4.0 mL X 3) three times. The combined organic phase was washed with brine, dried over Na_2SO_4, and concentrated in vacuo. The organic layer was concentrated in vacuo and purified by flash column chromatography with hexane and acetone (50:1 – 7:1) to give the desired acid product. According to ester procedure gave Et-ester product by EtI, K_2CO_3, and DMF. Yellow solid, 103 mg, 66%. $[\alpha]_D^{20}$ = +52.10 (*c* 1.000 $CHCl_3$) for 97.5:2.5 er. ^1H NMR (500 MHz,

Chloroform-*d*) δ 8.11 (d, *J* = 8.1 Hz, 1H), 8.06 – 8.00 (m, 2H), 7.87 (dd, *J* = 8.2, 1.5 Hz, 1H), 7.62 (t, *J* = 8.0 Hz, 1H), 7.53 – 7.46 (m, 3H), 7.27 – 7.24 (m, 5H), 7.14 – 7.07 (m, 7H), 7.04 – 7.01 (m, 2H), 6.96 (d, *J* = 1.8 Hz, 1H), 3.70 (q, *J* = 7.0 Hz, 2H), 0.46 (t, *J* = 7.1 Hz, 3H). ^{13}C NMR (126 MHz, Chloroform-*d*) δ 147.7, 147.2, 142.2, 142.1, 139.4, 136.0, 135.6, 134.3, 133.9, 133.4, 131.7, 130.4, 130.2, 129.3, 128.2, 124.4, 124.0, 122.9, 121.9, 121.7, 121.1, 120.7, 120.5, 119.8, 109.9, 107.6, 61.6, 12.9.HRMS (m/z, ESI): Calcd. for Chemical Formula: $C_{39}H_{29}Cl_2N_2O_2^+$ [M+H]$^+$: 627.1601, Found: 627.1605. HPLC analysis of the reaction product: Daicel Chiralpak IA, hexane/*iso*-propanol = 99:1, 0.8 mL/min, λ= 347 nm, retention time: 11.26 min (major) and 14.35min (minor).

Ethyl (*R*)-2-(2-(4-(diphenylamino)phenyl)-7-(4-methoxyphenyl)-9*H*-carbazol-9-yl)-4'-methoxy-[1,1'-biphenyl]-3-carboxylate (65)

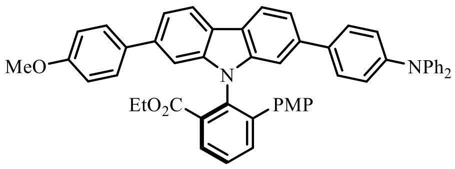

Under N_2 atmosphere, a mixture of $Pd(OAc)_2$ (0.87 mg, 0.0039 mmol) and SPhos (3.16 mg, 0.0077 mmol) was added to 0.3 mL THF, the mixture was then stirred at room temperature for 20 min. The resulting metal-ligand complex solution was added to a reaction flask containing 1.2 mL THF solution of *R*-3-chloro-2-(2-chloro-7-(4-(diphenylamino)phenyl)-9*H*-carbazol-9-yl)benzoic acid (**64**) (46 mg, 0.077 mmol) and *p*-methoxyphenylboronic acid (35 mg, 0.23 mmol), and K_3PO_4 (82 mg, 0.39 mmol), followed by addition of 0.50 mL H_2O. Then the resulting reaction mixture was stirred at 85 °C for 12 h. The reaction was then quenched with water, neutralized to pH 3 – 5 with 1.0 M HCl $_{(aq.)}$, and extracted with ethyl acetate (3.0 mL X 3) three times. The combined organic phase was washed with brine, dried over Na_2SO_4, and concentrated in vacuo. After that, the obtained crude product and K_2CO_3 (69 mg, 0.5 mmol) were added to DMF

(0.8 mL), then treated with EtI (55 mg, 0.355 mmol). The reaction mixture was stirred overnight at room temperature. Then the mixture was diluted with ethyl acetate (20 mL) and washed with water. The organic layer was concentrated in vacuo and purified by flash column chromatography with hexane and acetone (50:1 – 5:1) to to give the desired product, yellow soild, 31 mg, 52%, $[\alpha]_D^{20}$ = +7.58 (*c* 1.200 $CHCl_3$) for 97.5:2.5 er. ^{1}H NMR (500 MHz, Chloroform-*d*) δ 8.07 – 7.99 (m, 3H), 7.81 (dd, *J* = 7.8, 1.7 Hz, 1H), 7.69 (t, *J* = 7.8 Hz, 1H), 7.54 – 7.50 (m, 2H), 7.49 – 7.45 (m, 2H), 7.41 (td, *J* = 7.8, 1.6 Hz, 2H), 7.28 (d, *J* = 7.9 Hz, 4H), 7.17 – 7.12 (m, 8H), 7.09 – 7.03 (m, 4H), 7.00 – 6.96 (m, 2H), 6.60 – 6.55 (m, 2H), 3.87 (s, 3H), 3.65 – 3.58 (m, 5H), 0.41 (t, *J* = 7.1 Hz, 3H). ^{13}C NMR (126 MHz, Chloroform-*d*) δ 166.8, 159.0, 147.8, 146.9, 142.6, 142.5, 138.7, 138.5, 136.2, 134.8, 132.8, 130.5, 130.3, 129.3, 129.1, 128.8, 128.4, 128.1, 124.3, 124.2, 122.8, 122.0, 121.9, 120.1, 119.1, 119.0, 114.1, 113.7, 107.7, 61.3, 55.4, 55.0, 12.9. HRMS (m/z, ESI): Calcd. for Chemical Formula: $C_{53}H_{43}N_2O_4^+$ $[M+H]^+$: 771.3217, Found: 771.3211. HPLC analysis of the reaction product: Daicel Chiralpak IA, hexane/*iso*-propanol = 97:3, 1.0 mL/min, λ= 351 nm, retention time: 9.19 min (major) and 12.23 min (minor).

Ethyl (*R*)-3-chloro-2-(2-(4-cyanophenyl)-7-(4-(diphenylamino)phenyl)-9*H*-carbazol-9-yl)benzoate (66)

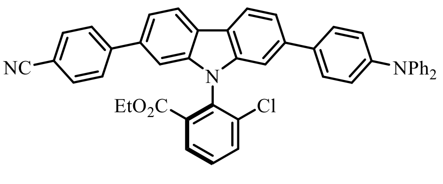

Under N_2 atmosphere, a mixture of $Pd_2(dba)_3$ (0.91 mg, 0.001 mmol) and (*R*, *S*)-**L9** (1.4 mg, 0.002 mmol) was added to 0.5 mL THF, and the mixture was stirred at room temperature for 20 min. The resulting metal-ligand complex solution was added to a reaction flask containing 1.5 mL THF solution of *R*-3-chloro-2-(2-chloro-7-(4-(diphenylamino)phenyl)-9*H*-

carbazol-9-yl)benzoic acid (**64**) (59.8 mg, 0.1 mmol) and *p*-cyanophenylboronic acid (17.6 mg, 0.12 mmol), and K_3PO_4 (106 mg, 0.5 mmol), followed by addition of 0.04 mL H_2O. Then the resulting reaction mixture was stirred at 60 °C for 18 h. The reaction was then quenched with water, neutralized to pH 3 – 5 with 1.0 M HCl $_{(aq.)}$, and extracted with ethyl acetate (2 mL X 3) three times. The combined organic phases were washed with brine, dried over Na_2SO_4, and concentrated in vacuo. After that, the obtained crude product and K_2CO_3 (69 mg, 0.5 mmol) were added to DMF (0.8 mL), then treated with EtI (55 mg, 0.355 mmol). The reaction mixture was stirred overnight at room temperature. Then the mixture was diluted with ethyl acetate (20 mL) and washed with water. The organic layer was concentrated in vacuo and purified by flash column chromatography with hexane and acetone (150:1 – 30:1) to give the desired product. Yellow solid, 53 mg, 77%, $[\alpha]_D^{20}$ = +7.58 (*c* 0.910 $CHCl_3$) for 97.5:2.5 er. ^1H NMR (500 MHz, Chloroform-*d*) δ 8.21 (d, *J* = 8.1 Hz, 1H), 8.18 (d, *J* = 8.1 Hz, 1H), 8.03 (dd, *J* = 7.8, 1.5 Hz, 1H), 7.89 (dd, *J* = 8.2, 1.5 Hz, 1H), 7.78 (d, *J* = 8.4 Hz, 1H), 7.70 (dd, *J* = 8.6, 4.2 Hz, 4H), 7.64 (t, *J* = 8.0 Hz, 1H), 7.56 – 7.49 (m, 4H), 7.28 – 7.24 (m, 4H), 7.16 – 7.12 (m, 7H), 7.05 – 7.01 (m, 2H), 3.71 – 3.64 (m, 2H), 0.43 (t, *J* = 7.1 Hz, 3H). ^{13}C NMR (126 MHz, Chloroform-*d*) δ 147.7, 147.2, 146.3, 142.5, 142.0, 139.7, 137.1, 135.9, 135.6, 134.3, 134.1, 133.6, 132.9, 132.5, 130.3, 130.1, 129.3, 128.2, 128.1, 128.0, 124.4, 124.0, 123.7, 123.0, 121.8, 120.9, 120.8, 119.8, 119.7, 119.1, 110.5, 108.3, 107.7, 61.6, 12.9. HRMS (m/z, ESI): Calcd. for Chemical Formula: $C_{46}H_{33}ClN_3O_2^+$ $[M+H]^+$: 694.2256, Found: 694.2251. HPLC analysis of the reaction product: Daicel Chiralpak IA, hexane/*iso*-propanol = 97:3, 1.0 mL/min, λ= 375 nm, retention time: 12.89 min (major) and 15.37 min (minor).

Ethyl (*S*)-2-(2-(4-cyanophenyl)-7-(4-(diphenylamino)phenyl)-9*H*-carbazol-9-yl)-4'-methoxy-

[1,1'-biphenyl]-3-carboxylate (67)

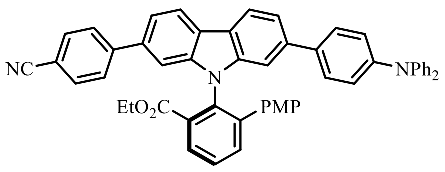

Under N_2 atmosphere, a mixture of $Pd(OAc)_2$ (0.73 mg, 0.0033 mmol) and SPhos (2.67 mg, 0.0065 mmol) was added to 0.3 mL THF, the mixture was then stirred at room temperature for 20 min. The resulting metal-ligand complex solution was added to a reaction flask containing 1.2 mL THF solution of ethyl (*R*)-3-chloro-2-(2-(4-cyanophenyl)-7-(4-(diphenylamino)phenyl)-9*H*-carbazol-9-yl)benzoate (**66**) (45 mg, 0.065 mmol) and *p*-methoxyphenylboronic acid (19.8 mg, 0.13 mmol), and K_3PO_4 (41 mg, 0.19 mmol), followed by addition of 0.50 mL H_2O. Then the resulting reaction mixture was stirred at 85 °C for 12 h. The reaction was then quenched with water, neutralized to pH 3 – 5 with 1.0 M HCl $_{(aq.)}$, and extracted with ethyl acetate (3.0 mL X 3) three times. The combined organic phase was washed with brine, dried over Na_2SO_4, and concentrated in vacuo. The organic layer was concentrated in vacuo and purified by flash column chromatography with hexane and acetone (50:1 – 7:1) to give the desired product, yellow soild, 35 mg, 70%, $[\alpha]_D^{20}$ = −18.20 (*c*1.500 $CHCl_3$) for 97.5:2.5 er. ^1H NMR (500 MHz, Chloroform-*d*) δ 8.07 (t, *J* = 8.4 Hz, 2H), 8.00 (dd, *J* = 7.7, 1.6 Hz, 1H), 7.81 (dd, *J* = 7.8, 1.7 Hz, 1H), 7.72 – 7.67 (m, 3H), 7.65 (d, *J* = 8.4 Hz, 2H), 7.48 – 7.39 (m, 4H), 7.29 – 7.25 (m, 4H), 7.17 (d, *J* = 1.4 Hz, 1H), 7.15 – 7.11 (m, 7H), 7.03 (dd, *J* = 8.1, 6.1 Hz, 4H), 6.57 – 6.53 (m, 2H), 3.64 – 3.56 (m, 5H), 0.40 (t, *J* = 7.1 Hz, 3H). ^{13}C NMR (126 MHz, Chloroform-*d*) δ 166.7, 159.1, 147.7, 147.1, 146.5, 142.9, 142.5, 142.2, 139.4, 136.7, 135.8, 134.9, 132.9, 132.8, 132.5, 130.4, 129.3, 129.1, 129.0, 128.1, 128.0, 124.4, 124.0, 123.5, 122.9, 121.6, 120.6, 119.4, 119.2_2, 119.1_8, 113.8, 110.3, 108.4, 107.8, 61.3, 55.1, 13.0. HRMS (m/z, ESI): Calcd. for Chemical Formula: $C_{53}H_{40}N_3O_3^+$ $[M+H]^+$: 766.3064, Found: 766.3061. HPLC analysis of the reaction product: Daicel

Chiralpak IA, hexane/*iso*-propanol = 97:3, 1.0 mL/min, λ= 363 nm, retention time: 12.84 min (major) and 15.95 min (minor).

C) (Tactic conjugated oligomer)

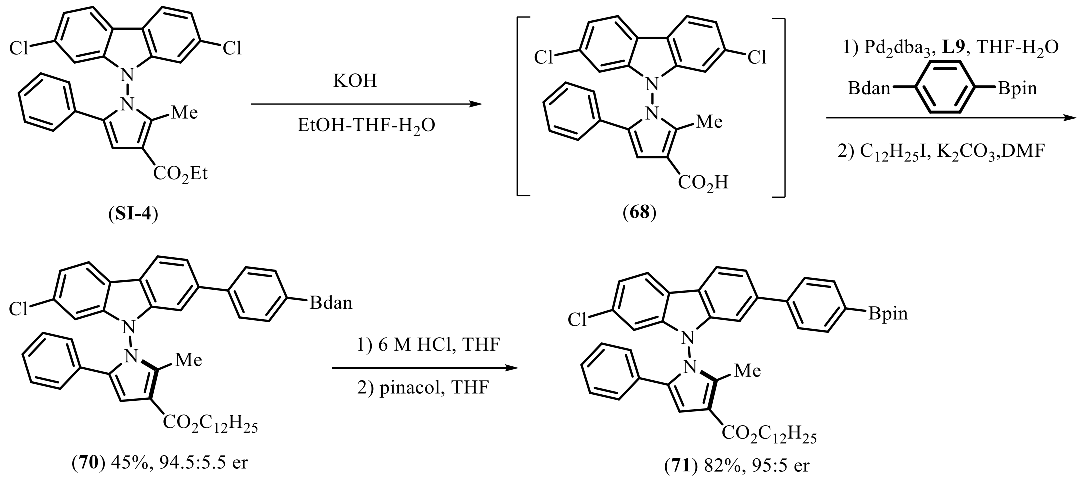

Dodecyl (*S*)-1-(2-(4-(1H-naphtho[1, 8-de][1, 3, 2]diazaborinin-2(3*H*)-yl)phenyl)-7-chloro-9*H*-carbazol-9-yl)-2-methyl-5-phenyl-1*H*-pyrrole-3-carboxylate (70)

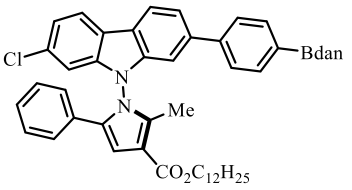

Under the N_2 atmosphere, ethyl 1-(2,7-dichloro-9*H*-carbazol-9-yl)-2-methyl-5-phenyl-1*H*-pyrrole-3-carboxylate (115.5 mg, 0.25 mmol) was dissolved in 1.0 mL THF, and 2.0 M KOH $_{(aq.)}$ (0.65 mL). The resulting reaction mixture was heated to 75 °C and stirred overnight. After cooling to room temperature, the reaction mixture was concentrated and dried in a vacuum. The crude acid **68** was gone to the next step without further purification.

Under N_2 atmosphere, a mixture of $Pd_2(dba)_3$ (2.29 mg, 0.0025 mmol) and **L9** (3.5 mg, 0.005 mmol) was added to 0.5 mL THF, and the mixture was stirred at room temperature for 20 min. The resulting metal-ligand complex solution was added to a reaction flask containing 4.5 mL THF solution of the above crude acid (**68**) and 2-(4-(4,4,5,5-tetramethyl-1,3,2-dioxaborolan-2-yl)phenyl)-2,3-dihydro-

1H naphtho[1,8de][1,3,2]diazaborinine (**69**) (129.5 mg, 0.35 mmol), and K_3PO_4 (265 mg, 1.25 mmol), followed by addition of 0.1 mL H_2O. Then the resulting reaction mixture was stirred at 60 °C for 18 h. The reaction was then quenched with water, neutralized to pH 4 – 6 with saturated $KH_2PO_{4\ (aq.)}$, and extracted with ethyl acetate (4 mL X 3) three times. The combined organic phase was washed with brine, dried over Na_2SO_4, and concentrated in vacuo. After that, the obtained crude product was dissolved in DMF (1.0 mL) and treated with 1-Iodododecane (296 mg, 1.0 mmol) and K_2CO_3 (276 mg, 2.0 mmol). The reaction mixture was stirred for 1h at room temperature. Then the mixture was diluted with ethyl acetate (10 mL) and washed with water. The organic layer was concentrated in vacuo and purified by flash column chromatography with hexane and acetone (100:1 – 20:1) to give the desired product, brownish red oil, 92 mg, 45%, $[\alpha]_D^{20}$ = +39.00 (*c* 1.000 $CHCl_3$) for 94.5:5.5 er. ^{1}H NMR (500 MHz, Chloroform-*d*) δ 8.22 (dd, *J* = 8.1, 2.2 Hz, 1H), 8.11 (dd, *J* = 8.4, 1.4 Hz, 1H), 7.84 – 7.79 (m, 2H), 7.78 – 7.75 (m, 2H), 7.72 (dd, *J* = 8.1, 1.2 Hz, 1H), 7.42 (dd, *J* = 8.3, 1.8 Hz, 1H), 7.39 (dd, *J* = 4.2, 2.1 Hz, 1H), 7.29 (dd, *J* = 7.5, 4.0 Hz, 3H), 7.20 (dd, *J* = 6.7, 2.6 Hz, 5H), 7.15 (d, *J* = 3.0 Hz, 1H), 6.56 (d, *J* = 7.2 Hz, 2H), 6.19 (s, 2H), 4.45 (td, *J* = 6.8, 2.4 Hz, 2H), 2.26 (d, *J* = 2.2 Hz, 3H), 1.94 – 1.88 (m, 2H), 1.62 – 1.56 (m, 2H), 1.53 – 1.47 (m, 2H), 1.38 (dd, *J* = 8.1, 3.3 Hz, 14H), 1.02 – 0.99 (m, 3H). ^{13}C NMR (126 MHz, Chloroform-*d*) δ 165.0, 142.6, 141.1, 141.0, 140.5, 138.3, 136.4, 133.9, 133.1, 132.1, 130.0, 128.6, 127.7_1, 127.6_6, 127.3, 126.6, 122.3, 121.8, 121.4, 121.2, 120.4, 119.9_1, 119.8_5, 117.9, 112.7, 108.9, 108.6, 107.0, 106.1, 64.4, 31.9, 29.7_1, 29.6_7, 29.6, 29.4, 28.9, 26.2, 24.9, 22.7, 14.2, 10.3. HRMS (m/z, ESI): Calcd. for Chemical Formula: $C_{52}H_{53}[11B]ClN_4O_2^+$ $[M+H]^+$: 811.3954, Found: 811.3945. HPLC analysis of the reaction product: Daicel Chiralpak IB, hexane/iso-propanol = 90:10, 1.0 mL/min, λ= 319 nm, retention time: 32.98 min (major) and 35.92 min (minor).

Dodecyl (*S*)-1-(2-chloro-7-(4-(4, 4, 5, 5-tetramethyl-1, 3, 2-dioxaborolan-2-yl)phenyl)-9*H*-carbazol-9-yl)-2-methyl-5-phenyl-1*H*-pyrrole-3-carboxylate (71)

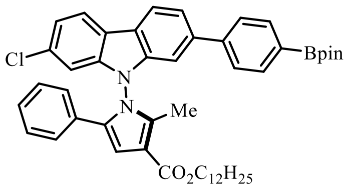

Under air atmosphere, dodecyl (*S*)-1-(2-(4-(1*H*-naphtho[1,8-de][1,3,2]diazaborinin-2(3*H*)-yl)phenyl)-7-chloro-9*H*-carbazol-9-yl)-2-methyl-5-phenyl-1*H*-pyrrole-3-carboxylate (**70**) (92 mg, 0.12 mmol) was dissolved into 1.0 mL THF, and 6.0 M HCl $_{(aq.)}$ (1.0 mL) was added into the solution. After that, the reaction mixture was stirred overnight at room temperature. The reaction was then quenched with water and extracted with ethyl acetate (4 mL X 3) three times. The combined organic phase was washed with brine, dried over Na_2SO_4, and concentrated in vacuo. The crude product was dissolved into 1.0 mL THF, and pinacol (118 mg, 1 mmol) was added to the solution. After that, the reaction mixture was stirred overnight at room temperature. Then the mixture was diluted with ethyl acetate (10 mL) and washed with water. The organic layer was concentrated in vacuo and purified by flash column chromatography with hexane and acetone (100:1 – 20:1) to give the desired product, brownish red oil, 71 mg, 82%. $[\alpha]_D^{20}$ = +17.45 (*c* 1.000 $CHCl_3$) for 95:5 er. ^{1}H NMR (500 MHz, Chloroform-*d*) δ 8.11 (d, *J* = 8.1 Hz, 1H), 8.01 (d, *J* = 8.3 Hz, 1H), 7.92 – 7.87 (m, 2H), 7.64 – 7.59 (m, 3H), 7.30 (dd, *J* = 8.4, 1.8 Hz, 1H), 7.26 (d, *J* = 1.5 Hz, 1H), 7.17 – 7.13 (m, 2H), 7.08 (dd, *J* = 5.2, 2.0 Hz, 3H), 7.04 (d, *J* = 1.8 Hz, 1H), 7.00 (s, 1H), 4.33 (t, *J* = 6.7 Hz, 2H), 2.13 (s, 3H), 1.79 (q, *J* = 7.1 Hz, 2H), 1.51 – 1.46 (m, 2H), 1.39 (s, 14H), 1.32 – 1.27 (m, 14H), 0.89 (s, 3H). ^{13}C NMR (126 MHz, Chloroform-*d*) δ 164.9, 143.5, 141.1, 141.0, 140.8, 138.3, 135.3, 133.9, 133.0, 130.0, 128.6, 127.6, 126.8, 126.6, 122.2, 121.7, 121.6, 121.0, 120.3, 119.8, 112.7, 108.9, 108.5, 107.1, 83.9, 64.3, 31.9, 29.7, 29.7, 29.6, 29.6, 29.4, 28.9, 26.2, 24.9, 22.7, 14.1, 10.3. HRMS (m/z, ESI): Calcd.

for Chemical Formula: $C_{48}H_{57}[11B]ClN_2O_4^+$ $[M+H]^+$: 711.4103, Found: 711.4106. HPLC analysis of the reaction product: Daicel Chiralpak IF, hexane/iso-propanol = 99:1, 1.0 mL/min, λ= 327 nm, retention time: 7.48 min (major) and 6.57 min (minor).

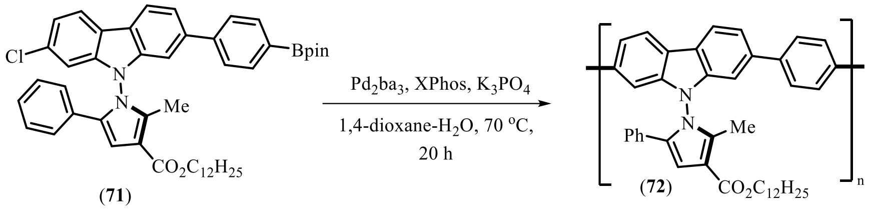

Under N_2 atmosphere, a mixture of $Pd_2(dba)_3$ (1.14 mg, 0.00125 mmol) and XPhos (2.38 mg, 0.005 mmol) was added to 0.05 mL 1,4-dioxane, the mixture was then stirred at room temperature for 20 min. The resulting metal-ligand complex solution was added to a reaction flask containing dodecyl (*S*)-1-(2-chloro-7-(4-(4,4,5,5-tetramethyl-1,3,2-dioxaborolan-2-yl)phenyl)-9*H*-carbazol-9-yl)-2-methyl-5-phenyl-1*H*-pyrrole-3-carboxylate (**71**) (38.5 mg, 0.05 mmol) and K_3PO_4 (53 mg, 0.25 mmol), followed by addition of 1,4-dioxane (0.05 mL) and H_2O (0.10 mL). Then the resulting reaction mixture was stirred at 70 °C for 20 h. The reaction was then quenched with water, neutralized to pH 4 – 6 with saturated KH_2PO_4 $_{(aq.)}$, and extracted with ethyl acetate (1.5 mL X 3) three times. The combined organic phase was washed with brine, dried over Na_2SO_4, and concentrated in vacuo. The organic layer was concentrated in vacuo and purified by PLC with hexane and acetone (3:1) to give the desired product, (**72**) 10 mg, 31%, yellowish solid. ^{1}H NMR (500 MHz, Chloroform-*d*) δ 8.13 (dd, *J* = 29.4, 7.7 Hz, 2H), 7.74 – 7.58 (m, 5H), 7.30 (d, *J* = 7.7 Hz, 1H), 7.25 – 7.13 (m, 3H), 7.11 – 6.96 (m, 5H), 4.30 (s, 2H), 2.18 – 2.09 (m, 3H), 2.01 (d, *J* = 6.4 Hz, 2H), 1.78 (t, *J* = 7.5 Hz, 3H), 1.64 (s, 3H), 1.31 (s, 14H), 0.89 (d, *J* = 6.5 Hz, 3H).

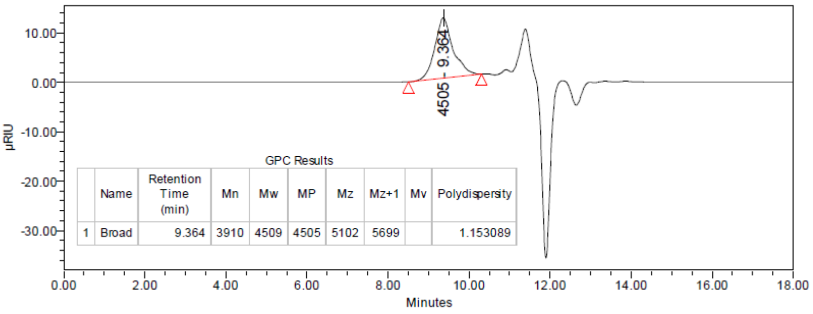

Gel permeation chromatography (GPC) for **72**

D) (24-Membered chiral macrocycle)

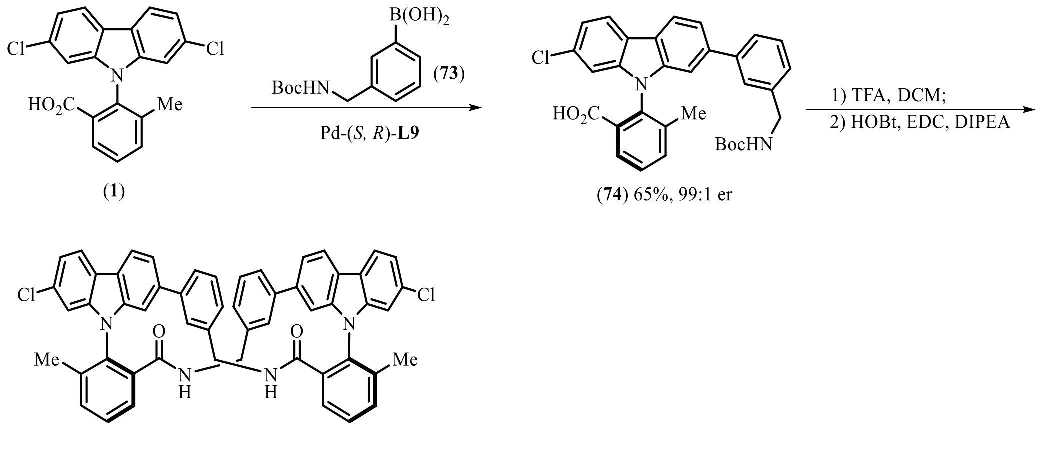

(**75**) 20% over two steps, >99.5:0.5 er

Ethyl (*R*)-2-(2-(3-(((*tert*-butoxycarbonyl)amino)methyl)phenyl)-7-chloro-9*H*-carbazol-9-yl)-3-methylbenzoic acid (74)

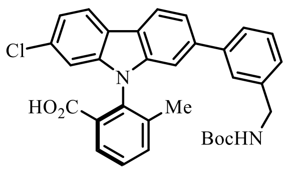

Under N_2 atmosphere, a mixture of $Pd_2(dba)_3$ (2.3 mg, 0.0025 mmol) and **L9** (3.5 mg, 0.005 mmol) was added to 0.5 mL THF, the mixture was then stirred at room temperature for 20 min. The resulting metal-ligand complex solution was added to a reaction flask containing 4.5 mL THF solution of 2-(2,7-dichloro-9*H*-carbazol-9-yl)-3-methylbenzoic acid **1** (92.5 mg, 0.25 mmol) and 3-((N-Boc-amino)methyl)phenylboronic acid (88 mg, 0.35 mmol), and K_3PO_4 (265 mg, 1.25 mmol), followed by addition of 0.10 mL H_2O. Then the resulting reaction mixture was stirred at 60 °C for 18 h. The reaction was then quenched with water, neutralized to pH 3 – 5 with 1.0 M HCl $_{(aq.)}$, and extracted

with ethyl acetate (4.0 mL X 3) three times. The combined organic phase was washed with brine, dried over Na_2SO_4, and concentrated in vacuo. The organic layer was concentrated in vacuo and purified by flash column chromatography with hexane and acetone as the eluents to give the desired acid product, white solid, 88 mg, 65%. ^1H NMR (500 MHz, Chloroform-*d*) δ 8.11 – 7.95 (m, 3H), 7.68 (d, *J* = 7.7 Hz, 1H), 7.56 (t, *J* = 7.8 Hz, 1H), 7.48 – 7.28 (m, 4H), 7.21 (dd, *J* = 8.2, 1.8 Hz, 2H), 7.01 (d, *J* = 1.5 Hz, 1H), 6.86 (d, *J* = 1.8 Hz, 1H), 4.85 (s, 1H), 4.29 (d, *J* = 5.0 Hz, 2H), 1.94 (s, 3H), 1.43 – 1.36 (m, 9H). ^{13}C NMR (126 MHz, Chloroform-*d*) δ 142.2, 142.0, 139.7, 139.5, 139.2, 136.0, 134.8, 131.8, 130.4, 129.3, 129.0, 126.7, 126.3, 122.0, 121.6, 121.2, 120.6, 120.3, 119.8, 109.5, 107.8, 44.7, 28.4, 17.5, 14.2. According to ester procedure gave Et-ester product (SI-**74**) by EtI, K_2CO_3, and DMF. $[\alpha]_D^{20}$ = +30.30 (*c* 1.000 $CHCl_3$) for 99:1 er. ^1H NMR (500 MHz, Chloroform-*d*) δ 8.14 (d, *J* = 8.1 Hz, 1H), 8.05 (d, *J* = 8.3 Hz, 1H), 7.99 (dd, *J* = 7.9, 1.6 Hz, 1H), 7.68 – 7.66 (m, 1H), 7.58 (t, *J* = 7.7 Hz, 1H), 7.53 – 7.47 (m, 3H), 7.37 (t, *J* = 7.9 Hz, 1H), 7.24 (dd, *J* = 8.3, 1.9 Hz, 2H), 7.09 – 7.07 (m, 1H), 6.92 (d, *J* = 1.8 Hz, 1H), 4.91 (s, 1H), 4.37 (d, *J* = 5.8 Hz, 2H), 3.70 (q, *J* = 7.1 Hz, 2H), 2.03 (s, 3H), 1.46 (s, 9H), 0.47 (t, *J* = 7.1 Hz, 3H). ^{13}C NMR (126 MHz, Chloroform-*d*) δ 165.9, 155.9, 142.3, 142.2, 142.1, 139.6, 139.4, 139.4, 135.2, 134.1, 132.0, 131.8, 129.8, 129.3, 129.0, 126.6, 126.3, 121.9, 121.5, 121.2, 120.5, 120.3, 119.8, 109.6, 108.0, 79.5, 61.1, 44.8, 28.4, 17.6, 13.0. HRMS (m/z, ESI): Calcd. for Chemical Formula: $C_{34}H_{33}ClN_2NaO_4^+$ $[M+Na]^+$: 591.2021, Found: 591.2025. HPLC analysis of the reaction product: Daicel Chiralpak ID, hexane/*iso*-propanol = 95:5, 1.0 mL/min, λ= 311 nm, retention time: 14.76 min (major) and 18.15 min (minor).

(*R*, *R*) 2^7, 8^7-Dichloro-3^6, 9^6-dimethyl-2^9*H*, 8^9*H*-5, 11-diaza-2, 8 (9, 2)-dicarbazola-1, 7 (1, 2), 3, 9 (1, 3) -tetrabenzenacyclododecaphane-4, 10-dione (75)

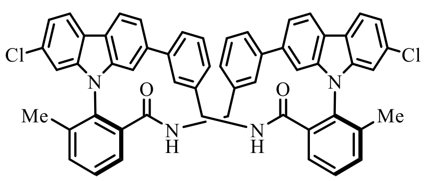

Under N_2 atmosphere, the above acid product (**74**) (50 mg, 0.093 mmol) was dissolved in 1.0 mL DCM, then TFA (0.2 mL) was added. The resulting reaction mixture was stirred overnight at room temperature. After that, the reaction solvents were removded in vacuo, the crude product was used directly without further purification. Under N_2 atmosphere, the above crude product, Et_3N (37.6 mg, 0.37 mmol), and HOBt·H_2O (36.5 mg, 0.19 mmol) were added DCM (1 mL). The mixture was cooled to 0 °C and EDC·HCl (24.3 mg, 0.19 mmol) was then added to the solution. After that, the resulting reaction mixture was stirred at room temperature for 24 h, then the reaction mixture was heated to reflux and was stirred for 24 h at this temperature. After that, the reaction was cooled to room temperature and was quenched with H_2O, then the mixture was extracted with EA (3 mL X 3) three times. The combined organic phases were washed with brine, dried over Na_2SO_4, and concentrated in vacuo. The crude materials were purified by flash column chromatography with hexane and acetone (30:1 – 6:1) to give the desired product, white solid, 15.5 mg, 20% yield over two steps. $[\alpha]_D^{20}$ = +30.30 (*c* 1.000 $CHCl_3$) for >99.5:0.5 er. ^{1}H NMR (500 MHz, Chloroform-*d*) δ 7.98 – 7.91 (m, 4H), 7.84 (d, *J* = 8.0 Hz, 2H), 7.57 (t, *J* = 7.7 Hz, 2H), 7.54 – 7.49 (m, 2H), 7.37 – 7.33 (m, 2H), 7.29 (dd, *J* = 8.3, 1.7 Hz, 4H), 7.12 (t, *J* = 7.6 Hz, 2H), 6.96 (d, *J* = 1.8 Hz, 2H), 6.79 (d, *J* = 1.4 Hz, 2H), 6.55 – 6.45 (m, 4H), 5.39 (s, 2H), 4.21 (dd, *J* = 13.9, 5.7 Hz, 2H), 3.65 (dd, *J* = 13.9, 4.7 Hz, 2H), 1.84 (s, 6H). ^{13}C NMR (126 MHz, Chloroform-*d*) δ 165.1, 141.6, 141.4, 140.8, 139.7, 138.7, 137.2, 135.7, 133.9, 132.5, 131.4, 130.0, 129.4, 128.8, 127.3, 126.6, 126.1, 121.7, 121.3, 121.2, 121.0, 120.7, 109.8, 106.8, 44.0, 17.4. HRMS (m/z, ESI): Calcd.

for Chemical Formula: $C_{54}H_{39}Cl_2N_4O_2^+$ $[M+H]^+$: 845.2445, Found: 845.2441. HPLC analysis of the reaction product: Daicel Chiralpak IH, hexane/*iso*-propanol = 80:20, 1.0 mL/min, λ= 330 nm, retention time: 8.05 min (major) and 14.95 min (minor).

E) (Curtius rearrangement)

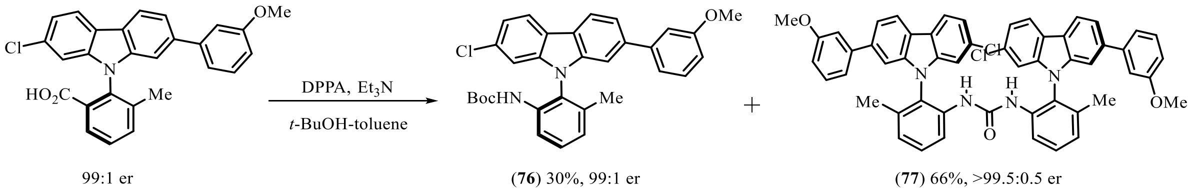

Under N_2 atmosphere, a mixture of DPPA (33 mg, 0.12 mmol) and (*R*)-2-(2-chloro-7-(3-methoxyphenyl)-9*H*-carbazol-9-yl)-3-methylbenzoic acid (44 mg, 0.1 mmol) was added to toluene (2 mL) and *tert*-butanol (1 mL), then the mixture was heated to 110 °C and stirred at this temperature for 18 h. After that the reaction was cooled to room temperature, quenched with water, and extracted with ethyl acetate (3.5 mL X 3) three times. The combined organic phase was washed with brine, dried over Na_2SO_4, and concentrated in vacuo. The organic layer was concentrated in vacuo and purified by flash column chromatography with hexane and acetone (150:1 – 10:1) to give product, (*R*)-*tert*-butyl (2-(2-chloro-7-(3-methoxyphenyl)-9*H*-carbazol-9-yl)-3-methylphenyl)carbamate (**76**), white solid, 16 mg, 30%. $[\alpha]_D^{20}$ =+43.00 (*c* 1.000 $CHCl_3$) for 99:1 er. ^1H NMR (500 MHz, Chloroform-*d*) δ 8.20 (dd, *J* = 23.2, 8.2 Hz, 2H), 8.08 (d, *J* = 8.3 Hz, 1H), 7.59 (dd, *J* = 8.1, 1.5 Hz, 1H), 7.45 (t, *J* = 8.0 Hz, 1H), 7.37 – 7.28 (m, 2H), 7.21 – 7.13 (m, 3H), 7.08 (d, *J* = 7.6 Hz, 1H), 6.99 (d, *J* = 1.8 Hz, 1H), 6.91 – 6.86 (m, 1H), 6.18 (s, 1H), 3.86 (s, 3H), 1.70 (s, 3H), 1.35 (s, 9H). ^{13}C NMR (126 MHz, Chloroform-*d*) δ 159.9, 152.6, 142.8, 141.5, 141.4, 140.2, 138.2, 137.3, 132.4, 130.0, 129.8, 125.3, 123.2, 122.3, 122.0, 121.5, 121.2, 120.8, 120.5, 120.1, 117.8, 113.6, 112.4, 110.0, 108.4, 81.1, 55.4, 28.1, 17.5. HRMS (m/z, ESI): Calcd. for Chemical Formula:

$C_{31}H_{30}ClN_2O_3^+$ [M+H]$^+$: 513.1939, Found: 513.1928. HPLC analysis of the reaction product: Daicel Chiralpak IA, hexane/iso-propanol = 99:1, 1.0 mL/min, λ= 311 nm, retention time: 12.48 min (major) and 21.59 min (minor).

1,3-Bis(2-((*R*)-2-chloro-7-(3-methoxyphenyl)-9*H*-carbazol-9-yl)-3-methylphenyl)urea (77), 28 mg, 66%, $[\alpha]_D^{20}$ =+21.07 (*c* 1.000 $CHCl_3$) for >99.5:0.5 er. ^1H NMR (400 MHz, Benzene-d_6) δ 8.00 (dd, *J* = 8.1, 0.7 Hz, 2H), 7.78 (dd, *J* = 8.2, 0.6 Hz, 2H), 7.58 (dd, *J* = 8.1, 1.6 Hz, 2H), 7.45 (dd, *J* = 1.6, 0.7 Hz, 2H), 7.26 – 7.22 (m, 6H), 7.13 – 7.10 (m, 2H), 7.07 (t, *J* = 7.8 Hz, 2H), 7.01 (t, *J* = 7.8 Hz, 2H), 6.77 – 6.74 (m, 2H), 6.55 – 6.52 (m, 2H), 6.31 – 6.25 (m, 2H), 3.26 (s, 6H), 2.70 (s, 2H), 1.60 (s, 6H).^{13}C NMR (126 MHz, Chloroform-*d*) δ 159.9, 144.3, 143.1, 141.2, 140.9, 139.9, 138.7, 137.1, 132.1, 130.0, 129.7, 122.1, 121.6, 121.3, 120.8, 120.7, 120.2, 120.1, 114.2, 113.7, 112.2, 109.9, 108.4, 55.4, 17.5. HRMS (m/z, ESI): Calcd. for Chemical Formula: $C_{53}H_{41}Cl_2N_4O_3^+$ [M+H]$^+$: 851.2550, Found: 851.2556. HPLC analysis of the reaction product: Daicel Chiralpak IA, hexane/iso-propanol = 99:1, 1.0 mL/min, λ= 311 nm, retention time: 29.92 min (major) and 22.00 min (minor).

F. (CP-TADF materials)

***R*-4-(7-Chloro-9-(2-(4, 6-diphenyl-1, 3, 5-triazin-2-yl)-6-methylphenyl)-9*H*-carbazol-2-yl)-*N*, *N*-diphenylaniline (78)**

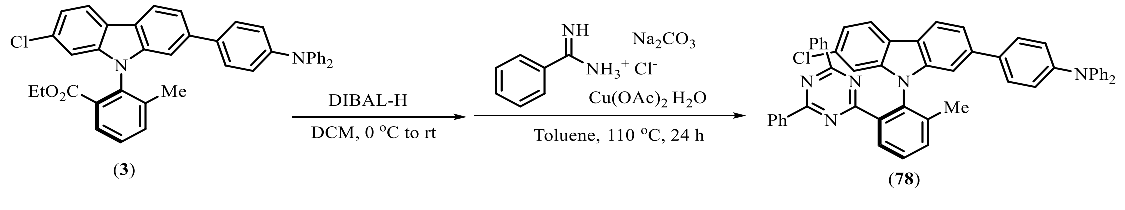

Under the N_2 atmosphere, ethyl (*R*)-2-(2-chloro-7-(4-(diphenylamino)phenyl)-9*H*-carbazol-9-yl)-3-methylbenzoate (**3**) (46 mg, 0.076 mmol) was dissolved in DCM (1 mL), then the mixture was cooded to 0 ºC. After that, DIBAL-H (1 mmol in THF, 0.3 mL, 0.3 mmol) was added dropwise the above reaction mixture. The mixture was stirred 0.5 h at 0 ºC then it was warmed to room temperature, and stirred 1 h. The reaction was then quenched with water at 0 ºC and extracted with ethyl acetate (10 mL X 3) three times. The combined organic phase was washed with brine, dried over Na_2SO_4, and concentrated in vacuo. The crude product was directly used for next step without further purification.

Under the air atmosphere, the above crude product, benzimidamide hydrochloride (28 mg, 0.18 mmol), $Cu(OAc)_2$ H_2O (4 mg, 0.02 mmol), and Na_2CO_3 (23 mg, 0.22 mmol) were added to the toluene (1 mL), then the mixture was heated to 110 ºC, and stirred for 24 h at this temperature. After that, the reaction was cooled to room temperature, then the mixture was diluted with ethyl acetate (10 mL) and washed with water. The organic layer was concentrated in vacuo and purified by flash column chromatography with hexane and acetone (200:1 – 30:1) to give the desired product (**78**), yellow solid, 31 mg, 57% over two steps. $[\alpha]_D^{20}$ = +172.93 (*c* 1.500 $CHCl_3$) for 98:2 er. ^1H NMR (500 MHz, Chloroform-*d*) δ 8.31 – 8.25 (m, 1H), 8.04 (d, *J* = 7.6 Hz, 4H), 7.93 (dd, *J* = 27.0, 8.1 Hz, 2H), 7.70 (q, *J* = 2.9 Hz, 2H), 7.48 – 7.36 (m, 5H), 7.31 (t, *J* = 7.7 Hz, 4H), 7.26 – 7.20 (m, 6H), 7.15 – 7.06 (m, 5H), 7.05 – 6.98 (m, 4H), 2.12 (s, 3H). ^{13}C NMR (126 MHz, Chloroform-*d*) δ 172.6, 171.3, 147.7, 146.9, 142.5, 142.3, 139.4, 139.1, 137.6, 135.8, 135.5, 134.2, 134.1, 132.3, 131.7, 130.3, 129.4, 129.3, 129.3, 128.8, 128.4, 128.2, 124.4, 124.3, 123.9, 122.9, 121.8, 121.5, 121.2, 120.5, 120.0, 119.3, 110.0, 107.9, 17.8. HRMS (m/z, ESI): Calcd. for Chemical Formula: $C_{52}H_{37}ClN_5^+$ $[M+H]^+$: 766.2732, Found: 766.2736. HPLC analysis of the reaction product: Daicel

Chiralpak ADH and ADH, hexane/iso-propanol = 99:1, 0.8 mL/min, λ= 346 nm, retention time: 13.16 min (major) and 14.63 min (minor).

***S*-4-(7-Chloro-9-(2-(4, 6-diphenyl-1, 3, 5-triazin-2-yl)-6-methylphenyl)-9*H*-carbazol-2-yl)-*N*, *N*-diphenylaniline (*ent*-78)**

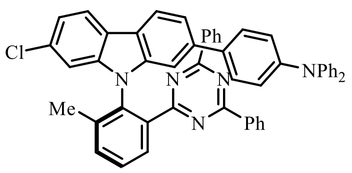

Prepared form *ent*-**3**. Yellow solid, 70 mg, 59% over two steps. $[\alpha]_D^{20} = -189.5$ (*c* 0.970 $CHCl_3$) for 3:97 er. NMR was same with *R*-4-(7-chloro-9-(2-(4, 6-diphenyl-1, 3, 5-triazin-2-yl)-6-methyl-phenyl)-9*H*-carbazol-2-yl)-*N*, *N*-diphenyl- aniline. HRMS (m/z, ESI): Calcd. for Chemical Formula: $C_{52}H_{37}ClN_5^+$ $[M+H]^+$: 766.2732, Found: 766.2729. HPLC analysis of the reaction product: Daicel Chiralpak ADH and ADH, hexane/iso-propanol = 99:1, 0.8 mL/min, λ= 346 nm, retention time: 15.19 min (major) and 13.50 min (minor).

***S*-4-(9-(2-(4, 6-Diphenyl-1, 3, 5-triazin-2-yl)-6-methylphenyl)-9*H*-carbazol-2-yl)-*N*,*N*-diphenyl -aniline (79)**

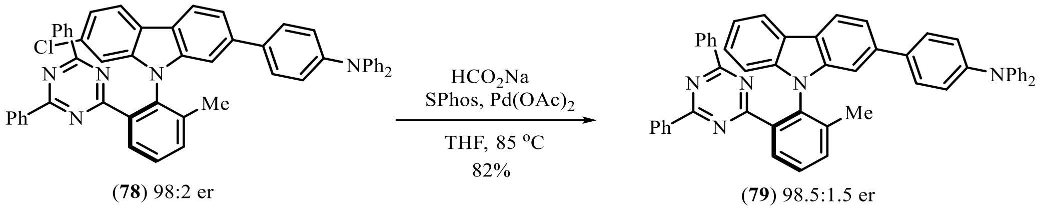

Under the N_2 atmosphere, *R*-4-(7-chloro-9-(2-(4, 6-diphenyl-1, 3, 5-triazin-2-yl)-6-methylphenyl)-9*H*-carbazol-2-yl)-*N*, *N*-diphenylaniline (**78**) (15 mg, 0.02 mmol), $Pd(OAc)_2$ (0.22 mg, 0.001 mmol), SPhos (0.82 mg, 0.002 mmol) and HCO_2Na (13.6 mg, 0.2 mmol) were added in THF (0.8 mL). The reaction mixture was heated to reflux and stirred for 48 h at this temperature. Then, the reaction was

cooled to room temperature and quenched with H_2O. The mixture was extracted with EA (3 mL X 3) three times. The combined organic phase was washed with brine, dried over Na_2SO_4, and concentrated in vacuo. The crude materials were purified by flash column chromatography with hexane and acetone (150:1 – 30:1) to give the desired product (**79**), 12 mg in 82% yield as a yellow solid. $[\alpha]_D^{20}$ = +198.50 (*c* 1.000 $CHCl_3$) for 98.5:1.5 er. ^{1}H NMR (500 MHz, Chloroform-*d*) δ 8.25 (dd, *J* = 7.2, 2.0 Hz, 1H), 8.03 – 7.96 (m, 6H), 7.72 – 7.65 (m, 2H), 7.44 – 7.35 (m, 6H), 7.31 – 7.27 (m, 4H), 7.26 – 7.22 (m, 6H), 7.14 – 7.05 (m, 5H), 7.03 – 6.98 (m, 4H), 2.13 (s, 3H). ^{13}C NMR (126 MHz, Chloroform-*d*) δ 172.8, 171.2, 147.7, 146.8, 141.9, 139.2, 139.0, 137.6, 136.1, 135.5, 134.7, 134.1, 132.2, 130.2, 129.2, 129.0, 128.8, 128.3, 128.2, 125.9, 124.2, 123.9, 123.2, 122.8, 122.2, 120.5, 120.4, 119.5, 118.7, 109.9, 107.7, 17.9. HRMS (m/z, ESI): Calcd. for Chemical Formula: $C_{52}H_{38}N_5^+$ $[M+H]^+$: 732.3122, Found: 732.3126. HPLC analysis of the reaction product: Daicel Chiralpak ADH, hexane/iso-propanol = 99:1, 0.8 mL/min, λ= 342 nm, retention time: 7.41 min (major) and 8.49 min (minor).

G) (2,7- Dibromocarbazole)

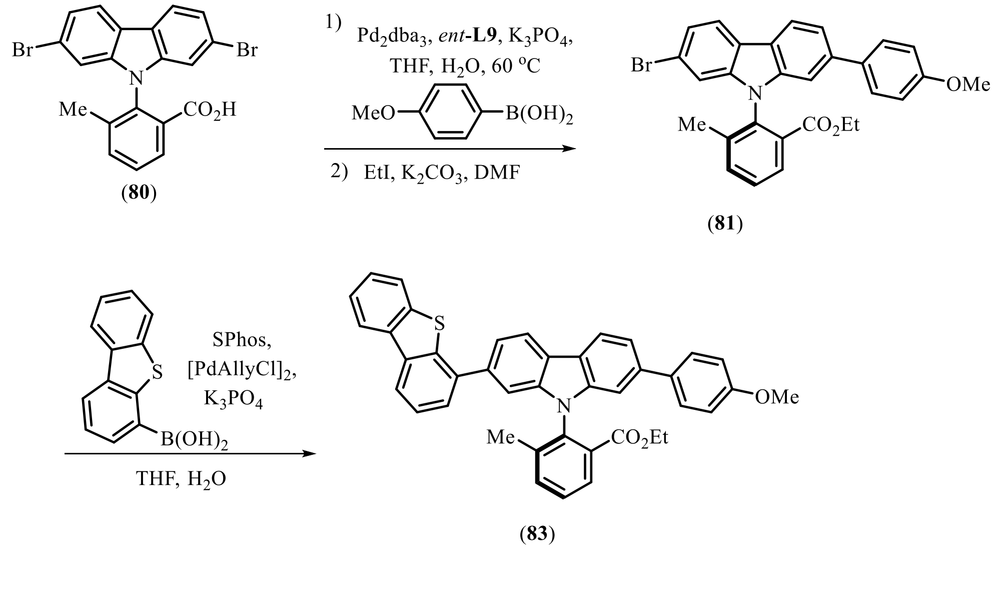

Ethyl (*S*)-2-(2-bromo-7-(4-methoxyphenyl)-9*H*-carbazol-9-yl)-3-methylbenzoate (81)

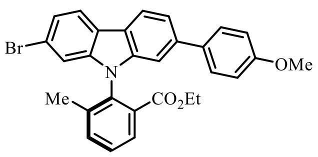

Under N_2 atmosphere, a mixture of $Pd_2(dba)_3$ (0.92 mg, 0.001 mmol) and *ent*-**L9** (1.4 mg, 0.002 mmol) was added to 0.2 mL THF, the mixture was then stirred at room temperature for 20 min. The resulting metal-ligand complex solution was added to a reaction flask containing 1.8 mL THF solution of 2-(2,7-dibromo-9*H*-carbazol-9-yl)-3-methylbenzoic acid (**80**) (46 mg, 0.1 mmol) and *p*-methoxyphenylboronic acid (7.6 mg, 0.05 mmol), and K_3PO_4 (106 mg, 0.5 mmol), followed by addition of 0.04 mL H_2O. Then the resulting reaction mixture was stirred at 60 °C for 18 h. The reaction was then quenched with water, neutralized to pH 3 – 5 with 1.0 M HCl $_{(aq.)}$, and extracted with ethyl acetate (4.0 mL X 3) three times. The combined organic phase was washed with brine, dried over Na_2SO_4, and concentrated in vacuo. The organic layer was concentrated in vacuo and purified by flash column chromatography with hexane and acetone (50:1 – 5:1) to give the desired acid product. According to ester procedure gave Et-ester product by EtI, K_2CO_3, and DMF. White solid, 13 mg, 50% (based on *p*-methoxyphenylboronic acid), $[\alpha]_D^{20}$ = -23.75 (*c* 0.320 $CHCl_3$) for 92.5:7.5 er.^{1}H NMR (600 MHz, Chloroform-*d*) δ 8.12 (dd, *J* = 8.1, 0.7 Hz, 1H), 8.00 – 7.96 (m, 2H), 7.67 – 7.65 (m, 1H), 7.57 (t, *J* = 7.7 Hz, 1H), 7.54 – 7.50 (m, 2H), 7.48 (dt, *J* = 8.1, 1.1 Hz, 1H), 7.39 – 7.35 (m, 1H), 7.07 (dd, *J* = 1.7, 0.6 Hz, 1H), 7.03 (dd, *J* = 1.5, 0.7 Hz, 1H), 6.97 – 6.92 (m, 2H), 3.83 (d, *J* = 0.7 Hz, 3H), 3.70 (q, *J* = 7.0 Hz, 2H), 2.02 (s, 3H), 0.46 (t, *J* = 7.1, 3H). ^{13}C NMR (151 MHz, Chloroform-*d*) δ 166.0, 159.1, 142.5, 142.1, 139.7, 139.4, 135.1, 134.3, 134.1, 132.0, 129.7, 129.2, 128.5, 122.9, 122.0, 121.4, 121.3, 120.5, 119.5, 119.4, 114.2, 112.5, 107.5, 61.1, 55.4, 17.7, 13.0. HRMS (m/z, ESI): Calcd. for Chemical Formula: $C_{29}H_{25}BrNO_3^+$ $[M+H]^+$: 514.1012, Found: 514.1011. HPLC analysis of the reaction product: Daicel Chiralpak ADH, hexane/*iso*-propanol =

99:1, 1.0 mL/min, λ= 316 nm, retention time: 14.68 min (major) and 13.67 min (minor).

Ethyl (*S*)-2-(2-(dibenzo[b,d]thiophen-4-yl)-7-(4-methoxyphenyl)-9*H*-carbazol-9-yl)-3-methylbenzoate (83)

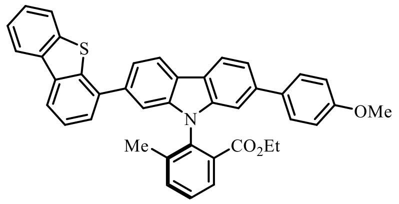

Under N_2 atmosphere, a mixture of $[PdAllyCl]_2$ (0.35 mg, 0.00095 mmol) and SPhos (0.39 mg, 0.0019 mmol) was added to 0.2 mL THF, and the mixture was stirred at room temperature for 20 min. The resulting metal-ligand complex solution was added to a reaction flask containing 0.3 mL THF solution of ethyl (*S*)-2-(2-bromo-7-(4-methoxyphenyl)-9*H*-carbazol-9-yl)-3-methylbenzoate (**81**) (10 mg, 0.019 mmol) and 4-dibenzothiopheneboronic acid **82** (8.7 mg, 0.038 mmol), and K_3PO_4 (20 mg, 0.095 mmol), followed by addition of 0.1 mL H_2O. Then the resulting reaction mixture was stirred at 70 °C for 10 h. The reaction was then quenched with water, and extracted with ethyl acetate (2 mL X 3) three times. The combined organic phases were washed with brine, dried over Na_2SO_4. Then the organic layer was concentrated in vacuo and purified by flash column chromatography with hexane and acetone (150:1 – 30:1) to give the desired product. White solid, 11 mg, 91%, $[\alpha]_D^{20}$ = -1.40 (*c* 1.000 $CHCl_3$) for 92:8 er. ^1H NMR (600 MHz, Chloroform-*d*) δ 8.27 (dd, *J* = 8.0, 0.7 Hz, 1H), 8.22 (dd, *J* = 8.1, 0.7 Hz, 1H), 8.19 – 8.16 (m, 1H), 8.13 (dd, *J* = 6.5, 2.5 Hz, 1H), 7.98 – 7.96 (m, 1H), 7.85 – 7.82 (m, 1H), 7.69 – 7.63 (m, 2H), 7.60 – 7.50 (m, 6H), 7.48 – 7.43 (m, 2H), 7.30 (dd, *J* = 1.5, 0.7 Hz, 1H), 7.11 (dd, *J* = 1.6, 0.6 Hz, 1H), 7.00 – 6.95 (m, 2H), 3.85 (s, 3H), 3.77 – 3.67 (m, 2H), 2.14 (s, 3H), 0.48 (t, *J* = 7.1 Hz, 3H). ^{13}C NMR (126 MHz, Chloroform-*d*) δ 166.3, 159.1, 142.6, 142.1, 139.7, 139.5, 139.4, 139.0, 138.5, 137.9, 136.2, 135.9, 135.0, 134.6, 134.5, 132.2, 129.7, 129.0, 128.5, 127.2, 126.7, 125.0, 124.3, 122.8, 122.6, 121.8, 121.7, 120.6, 120.5, 120.2, 120.2, 119.2, 114.2, 109.3, 107.4, 61.2, 55.4, 17.8, 13.0. HRMS

(m/z, ESI): Calcd. for Chemical Formula: $C_{41}H_{32}NO_3S^+$ $[M+H]^+$: 618.2097, Found: 618.2100. HPLC analysis of the reaction product: Daicel Chiralpak IF, hexane/*iso*-propanol = 97:3, 1.0 mL/min, λ= 325 nm, retention time: 12.73 min (major) and 14.22 min (minor).

Chiroptical properties

Absolute configuration of building units was extracted directly from X-ray crystal structures. The optimized initial structure was modified on the crystal structure. Then, the B3LYP-D3/def2SVP basis set was utilized to calculate absorption/ECD spectra based on time-dependent density functional theory (TDDFT). Excited singlet and triplet state geometries were generated with TDDFT methods starting from ground-state 171 optimized geometries at B3LYP-D3 level of theory (6-31+G(d) basis set). Charge transfer complex structures were optimized by the B3LYP-D3/def2SVP basis set of the Gaussian 16 program to obtain a low-energy conformation. All computed spectra are unshifted and multiplied by a 0.3 factor.

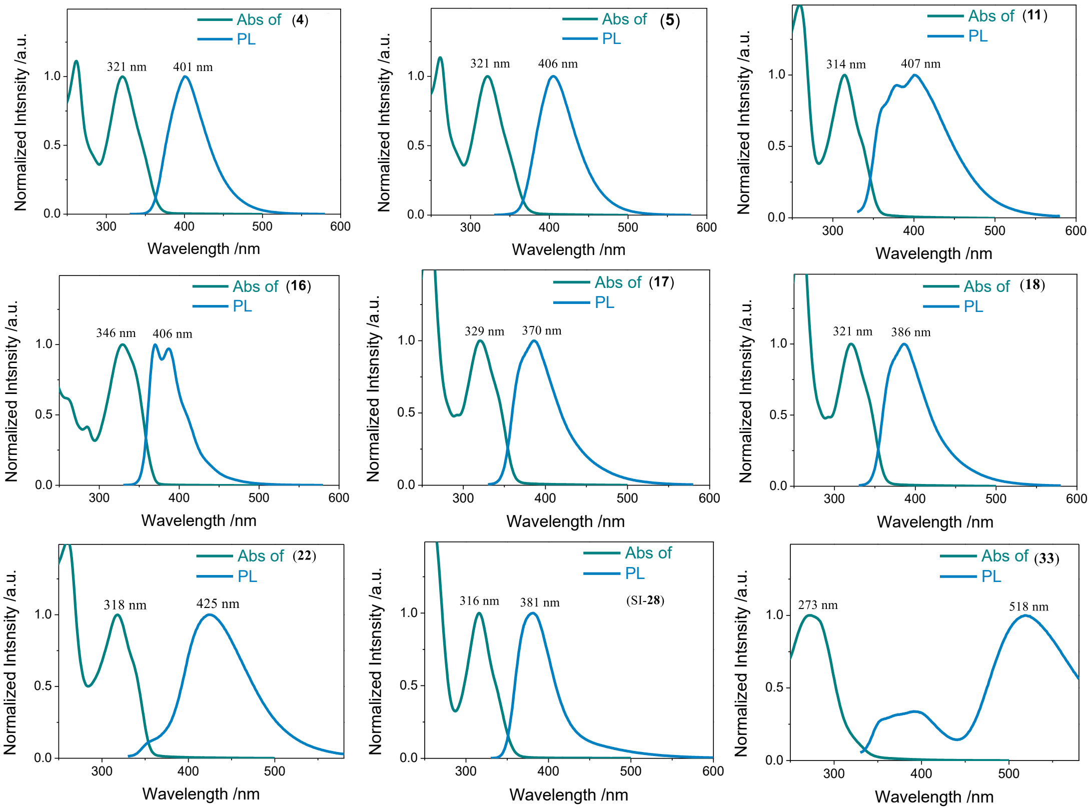

Fig. S1. Normalized absorption and emission spectra of carbazole-based axial chiral compounds (0.2 mM) in solution (DCE).

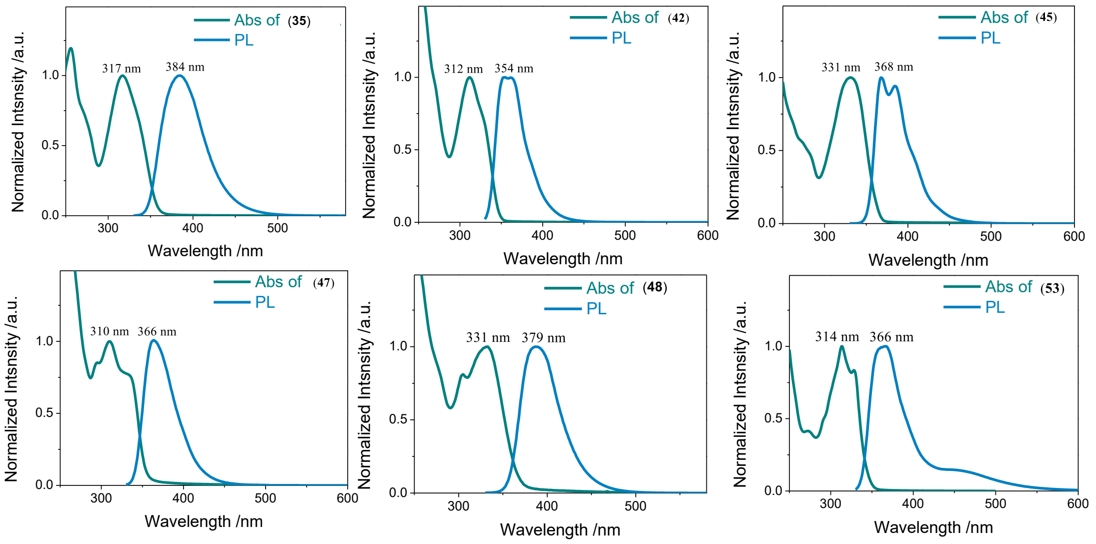

Fig. S1-continued. Normalized absorption and emission spectra of carbazole-based axial chiral compounds (0.2 mM) in solution (DCE).

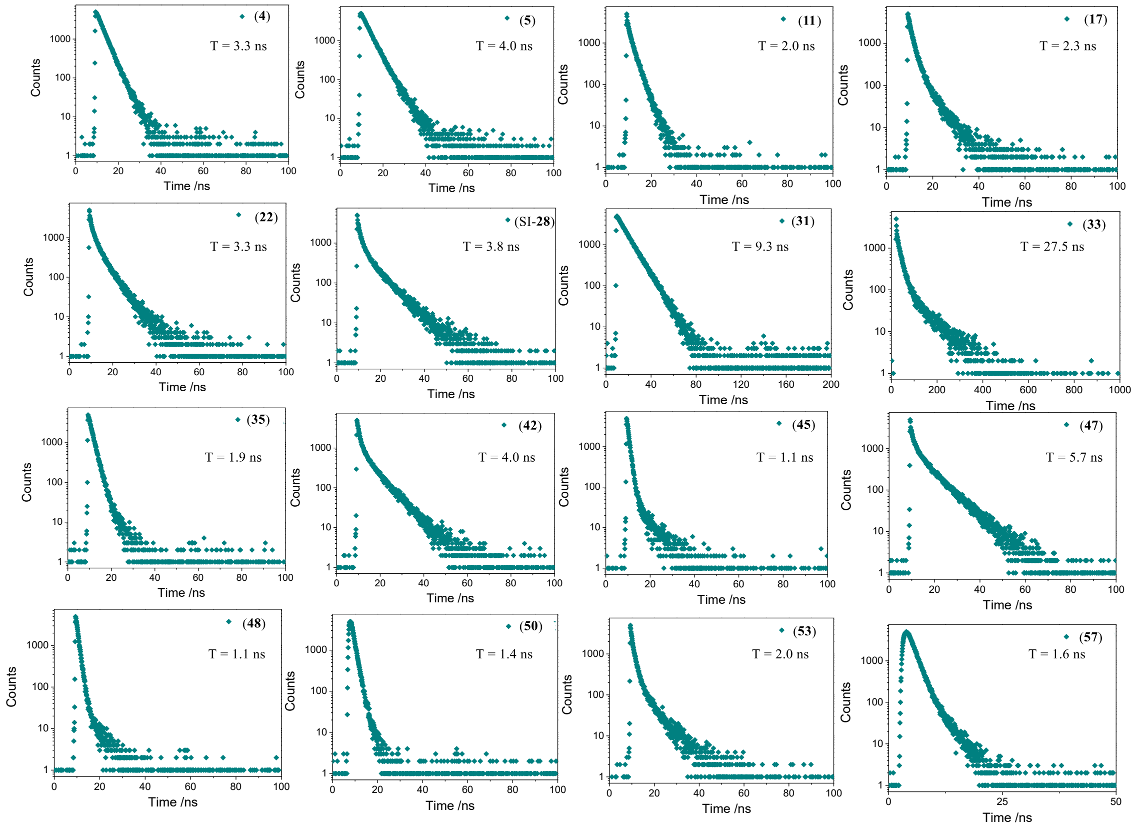

Fig. S2. Life time (τ) of carbazole-based axial chiral compounds.

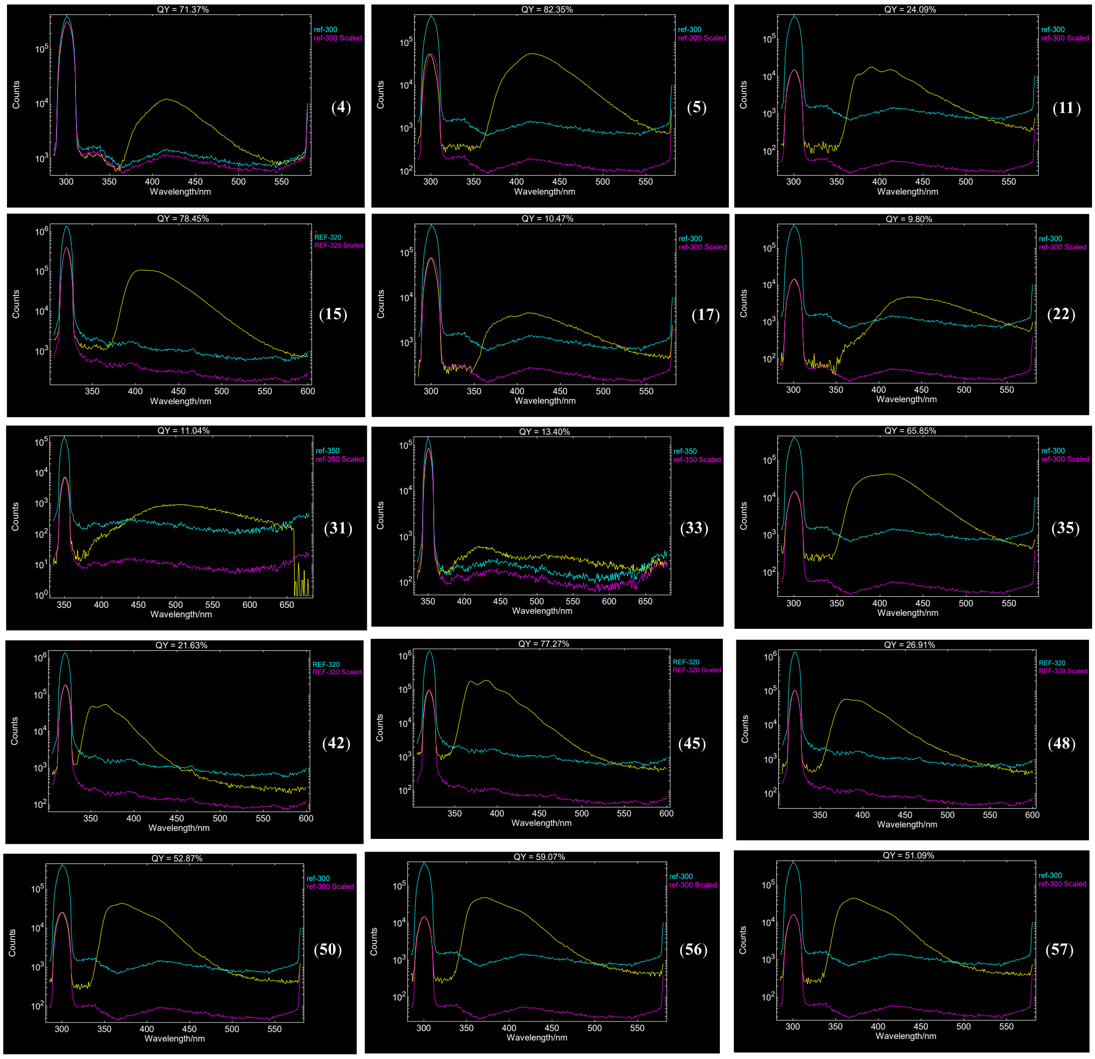

(**4**) QY = 71.4%; (**5**) QY = 82.4%; (**11**) QY = 24.1%; (**15**) QY = 78.5%; (**17**) QY = 10.5%; (**22**) QY = 9.8%; (**31**) QY = 11%; (**33**) QY = 13.4%; (**35**) QY = 65.9%; (**42**) QY = 21.6%;

(**45**) QY = 77.3%; (**48**) QY = 26.9%; (**50**) QY = 52.9%; (**56**) QY = 59.1%; (**57**) QY = 51.1%

Fig. S3. Quantum yields (Φ) of carbazole-based axial chiral compounds

Table S2 Configure and Cotton effects of carbazole-based axial chiral compounds.

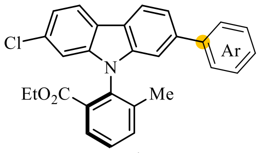

C-N atropisomers

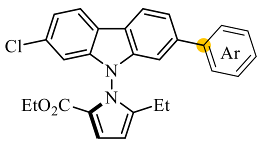

N-N atropisomers

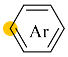	Config.	CD sign[a]	Config.	CD sign[a]
	with *ent*-**L9**		with **L9**	
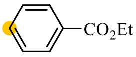	*S*-(**4**)	−	*R*-(**35**)	+
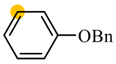	*S*-(**8**)	−	*R*-(**41**)	+
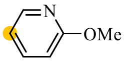	*S*-(**11**)	−	*R*-(**42**)	+
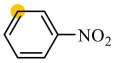	*R*-(**6**)	+	*R*-(**37**)	+
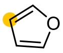	*R*-(**12**)	+	*R*-(**43**)	+
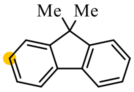	*R*-(**16**)	+	*R*-(**45**)	+
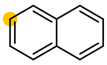	*R*-(**17**)	+	*R*-(**46**)	+
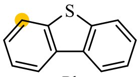	*R*-(**18**)	+	*R*-(**47**)	+
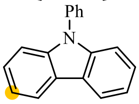	*R*-(**20**)	+	*R*-(**48**)	+

[a]CD spectra of carbazole-based axial chiral compounds (0.2 mM) in solution (DCE). The +/- sign at maximum absorbance wavelength of each compound is shown.

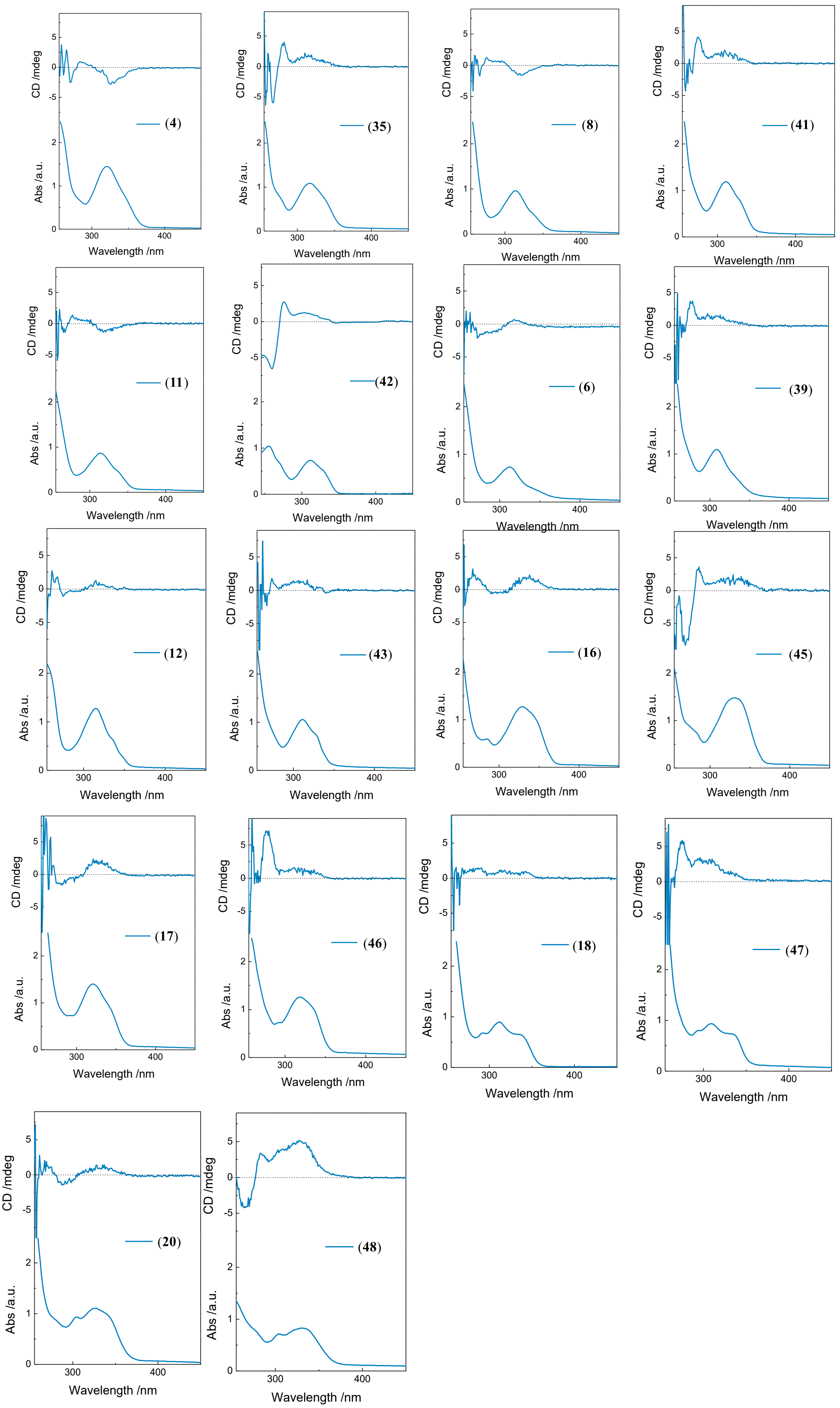

Fig. S4. CD spectra of carbazole-based axial chiral compounds (0.2 mM) in solution (DCE).

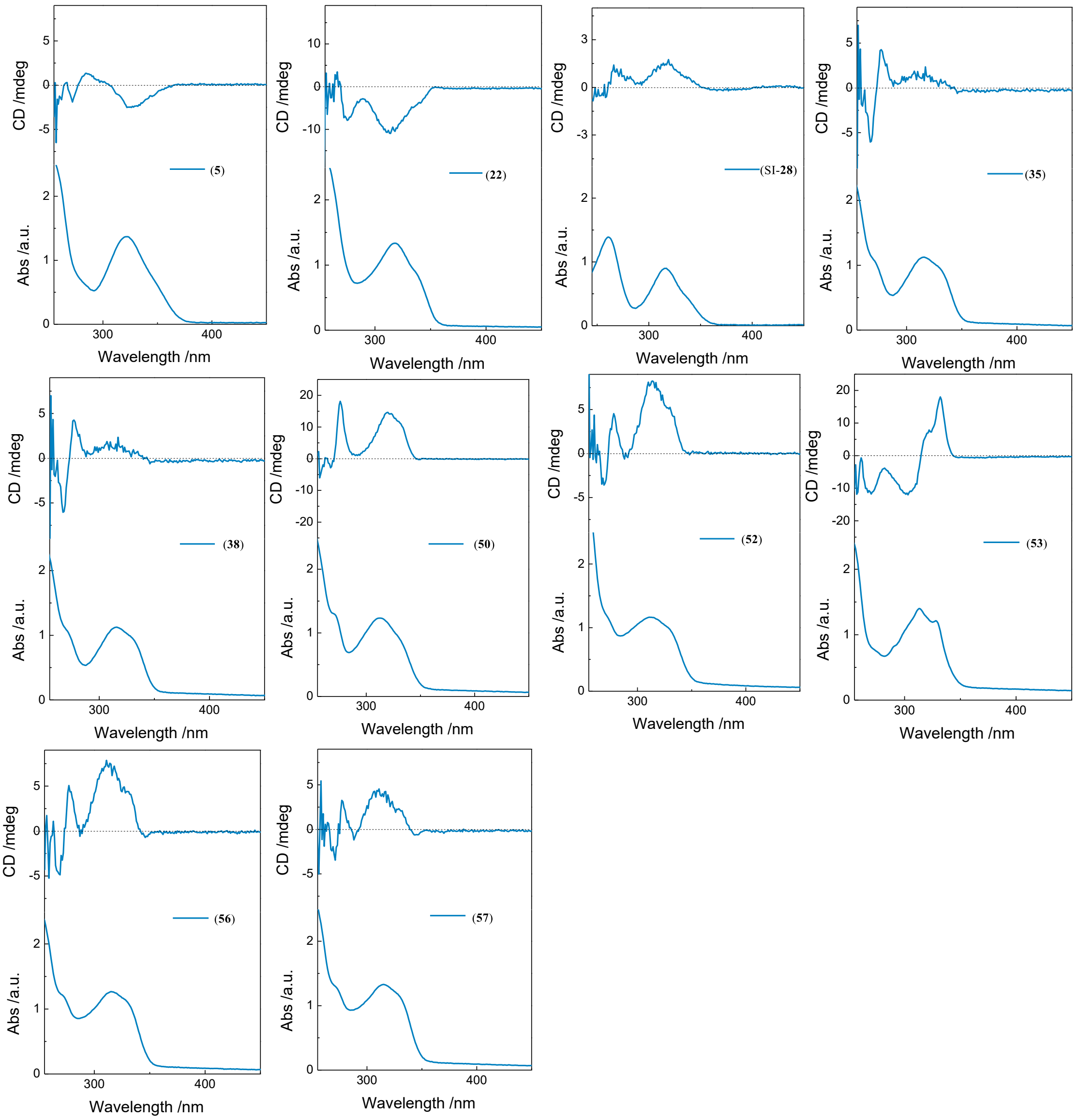

Fig. S4-continued. CD spectra of carbazole-based axial chiral compounds (0.2 mM) in solution (DCE).

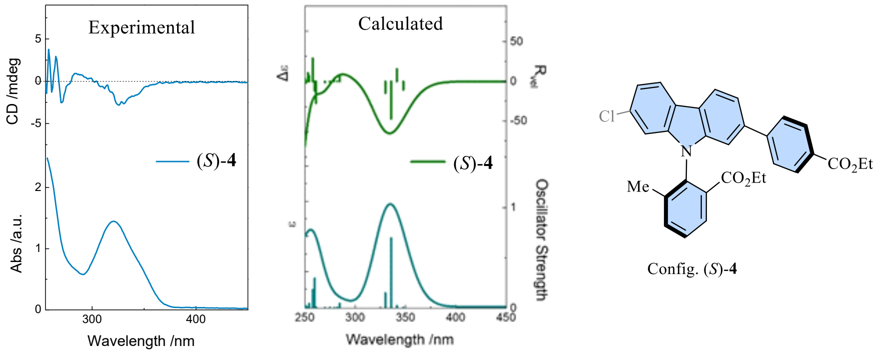

Fig. S5. CD spectrum of (*S*)-**4** in DCE with a concentration of 0.2 mM. and Calculated electronic CD spectrum of (*S*)-**4** based on the DFT-optimized geometry.

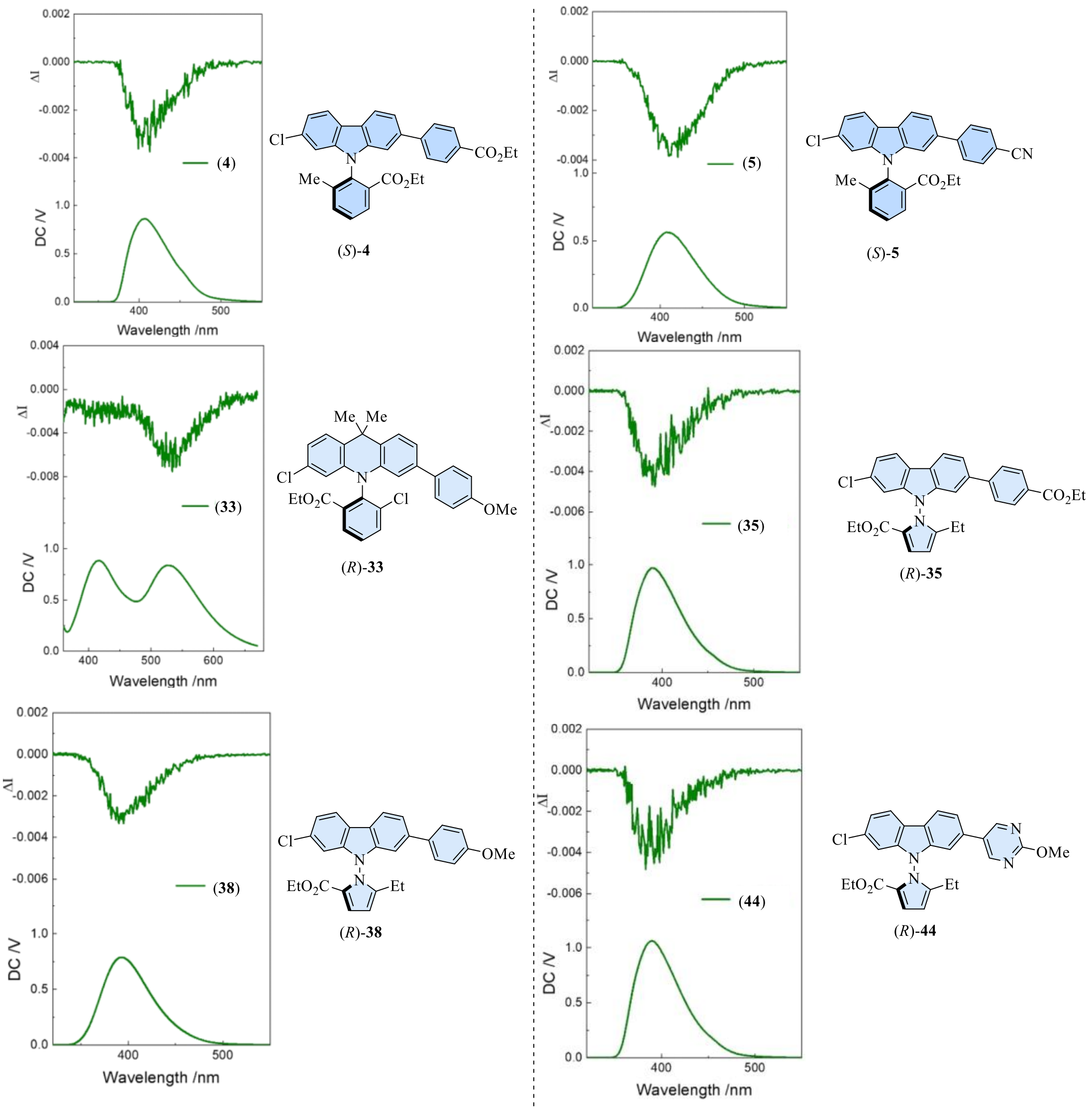

Fig.S6. CPL spectra (λ_{ex} = 300 nm) of carbazole-based axial chiral compounds in PMMA.

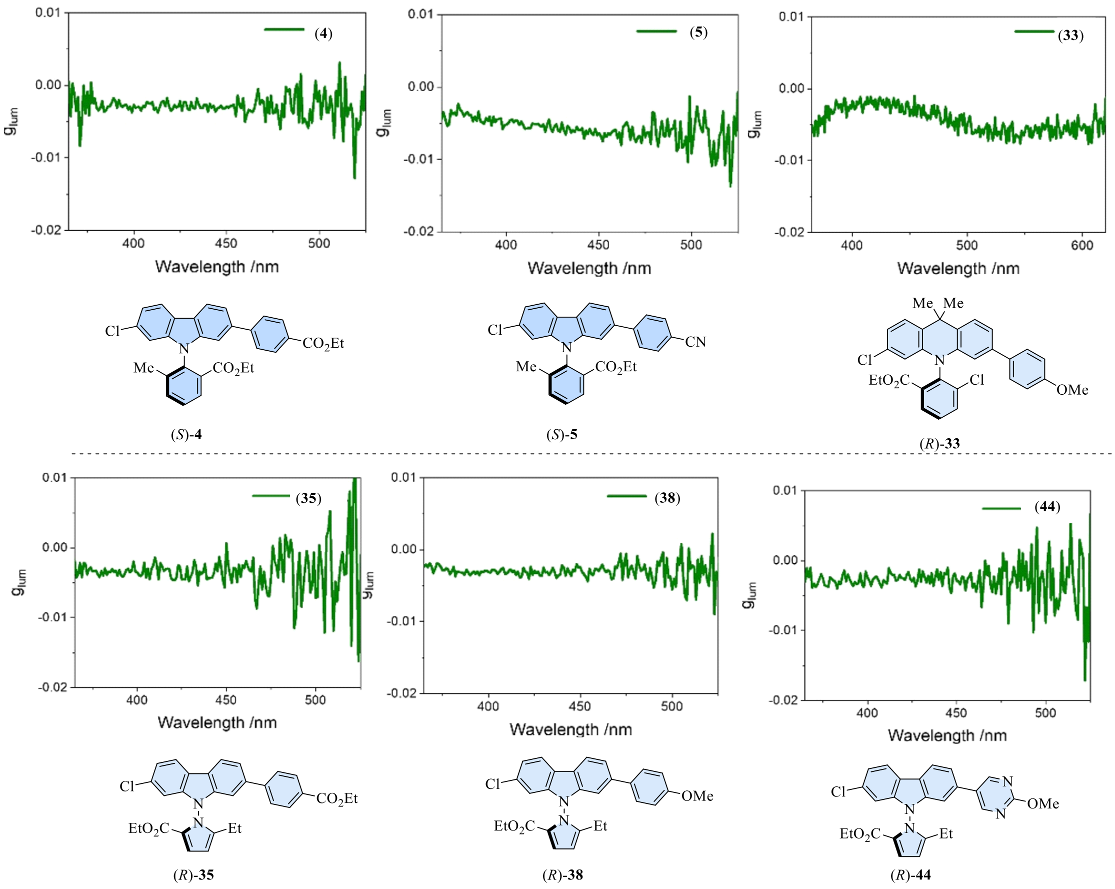

Fig. S7. G$_{lum}$ spectra (λ_{ex} = 300 nm) of carbazole-based axial chiral compounds in PMMA.

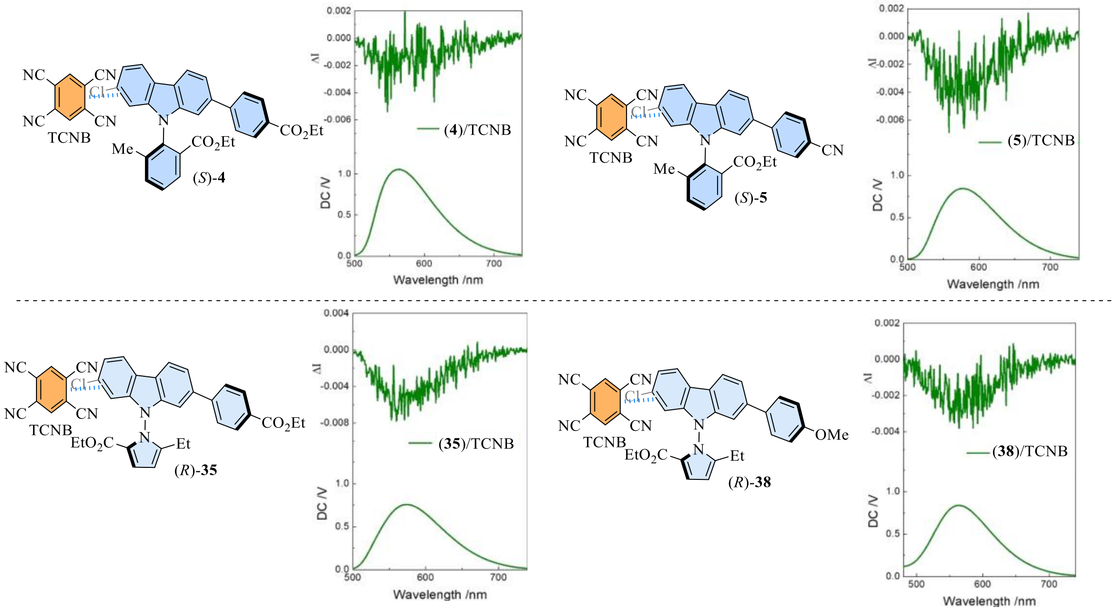

Fig. S8. CPL spectra (λ_{ex} = 300 nm) of carbazole-based axial chiral compounds with TCNB (1 molar equiv.) in PMMA.

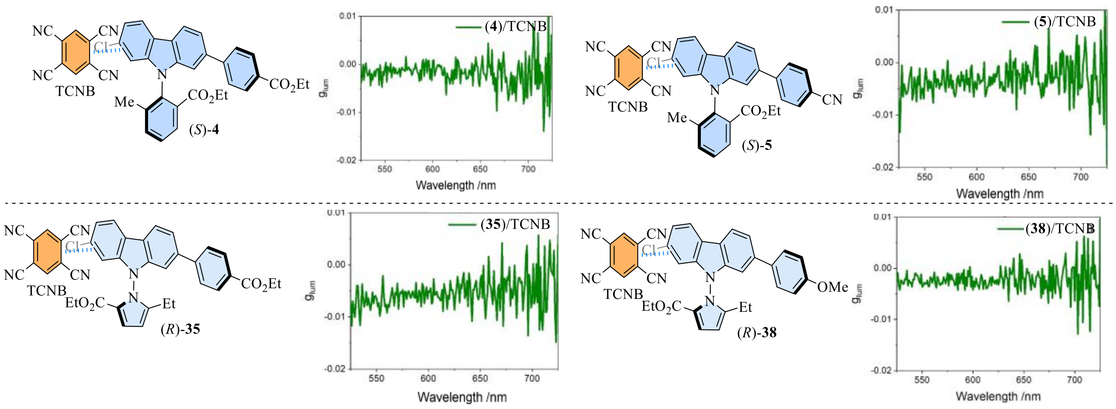

Fig. S9. G_{lum} spectra (λ_{ex} = 300 nm) of carbazole-based axial chiral compounds with TCNB (1 molar equiv.) in PMMA**.**

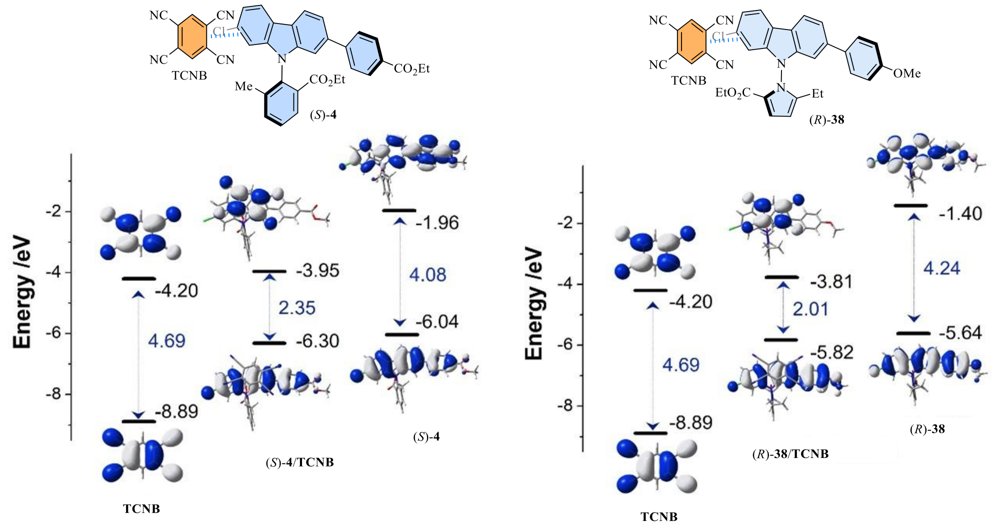

Fig. S10. HOMO-LUMO energy levels and distributions of TCNB, (*S*)-**4**, (*R*)-**38**, (*S*)-**4**/TCNB and (*R*)-**38**/TCNB.

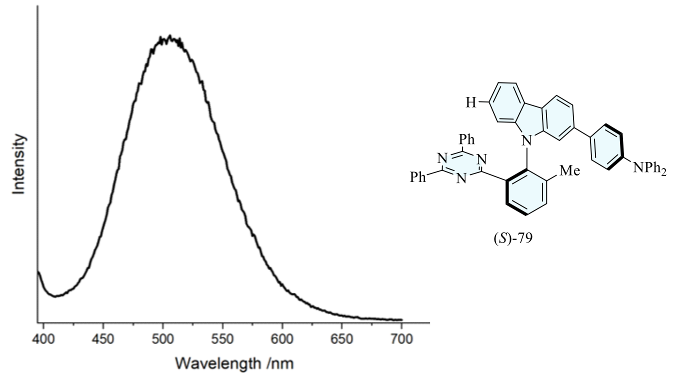

Fig. S11. Delayed emission spectrum of (*S*)-**79** in the DPEPO matrix. (λ_{ex} = 400 nm)

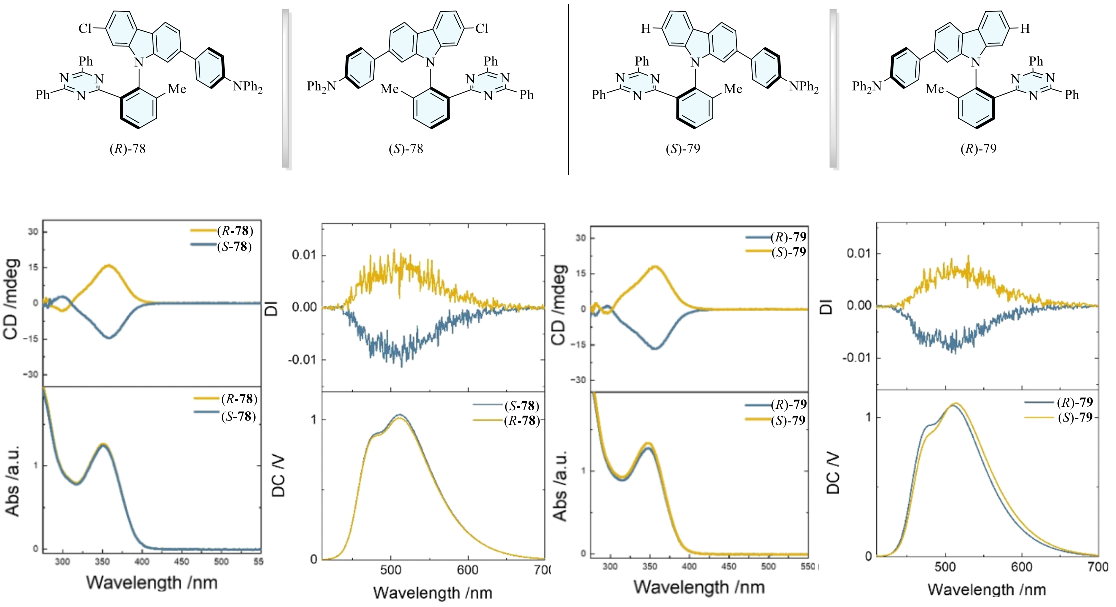

Fig. S12. CD, CPL spectra (CPL was measured in DPEPO matrix, CD was measured in DCE, c = 0.2 mM). (λ_{ex} = 400 nm)

NMR Spectra

L5-((*S*)-2-(dicyclohexylphosphaneyl)-2',6'-diisopropoxy-[1,1':3',1''-terphenyl]-5'-carbonyl)-

***D*-alanine** ^{1}H NMR (500MHz, Acetone-d_6)

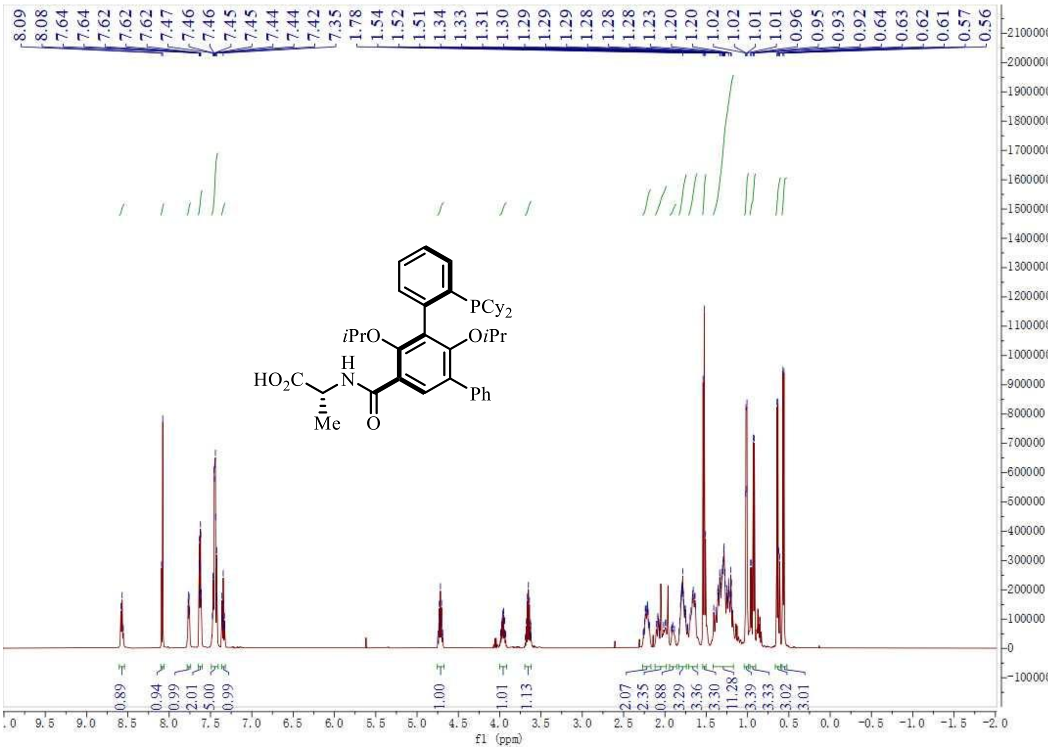

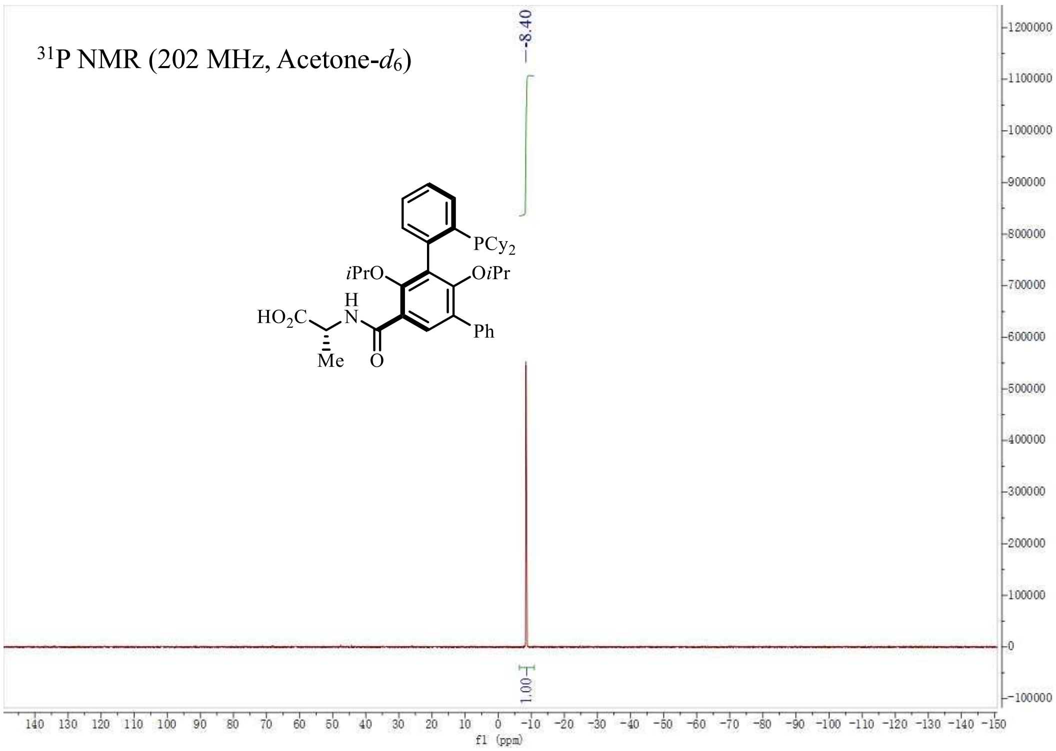

^{13}C NMR (126 MHz, Acetone-d_6)

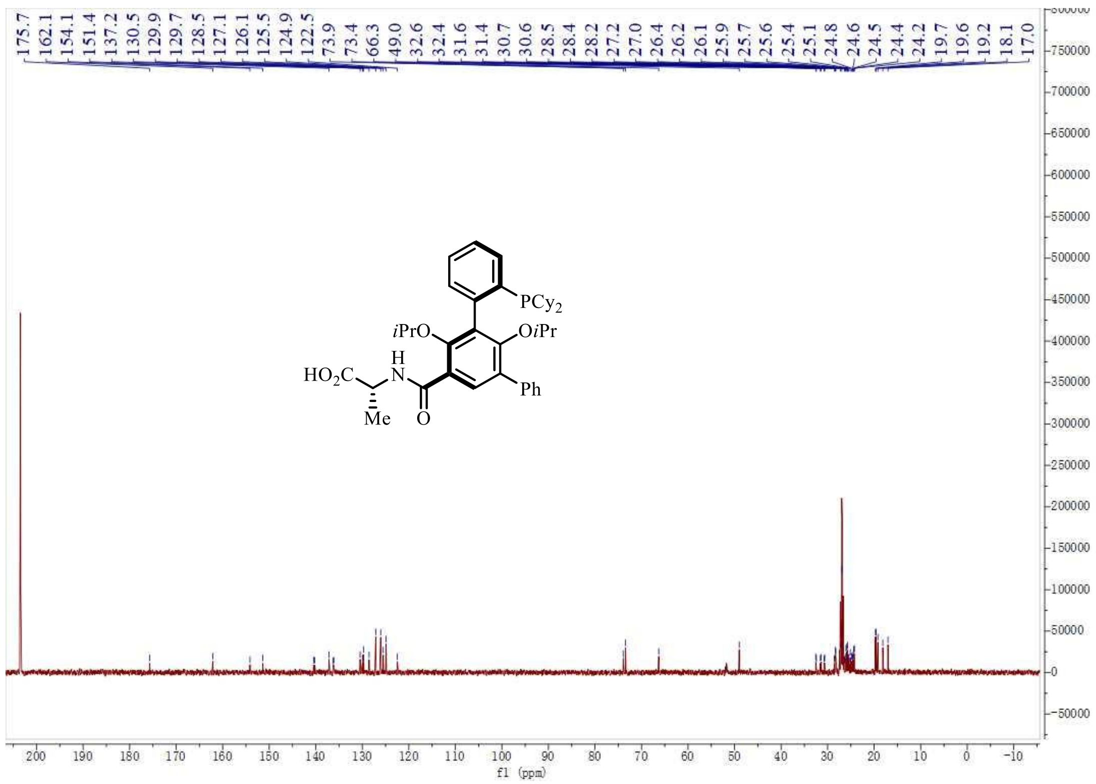

L6-((*S*)-2-(dicyclohexylphosphaneyl)-2',6'-diisopropoxy-[1,1':3',1''-terphenyl]-5'-carbonyl)-

***D*-leucine** ^{1}H NMR (500 MHz, Acetone-d_6)

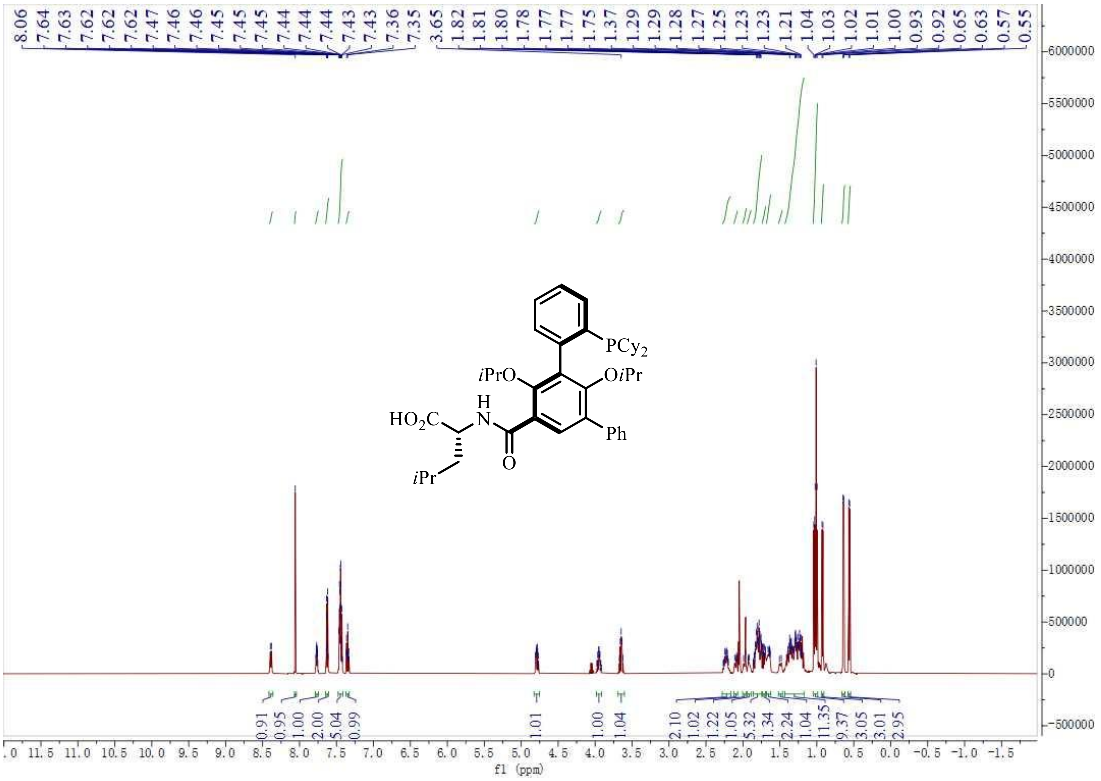

^{13}C NMR (126 MHz, Acetone-d_6)

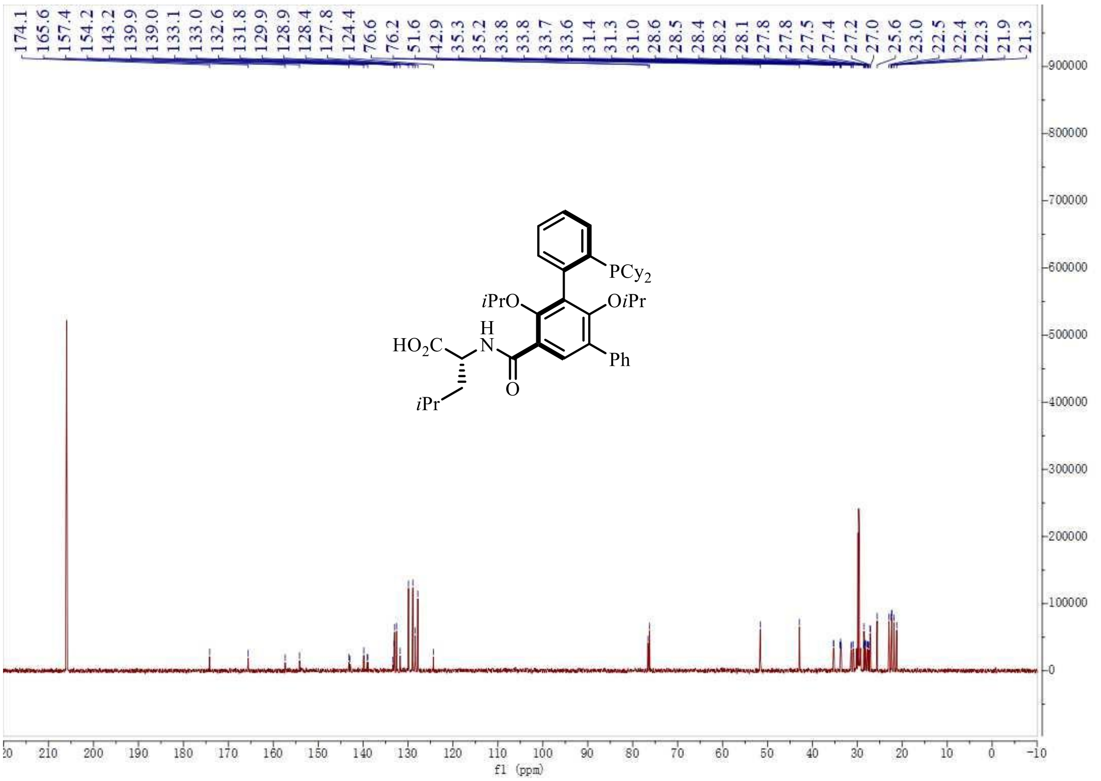

^{31}P NMR (202 MHz, Acetone-d_6)

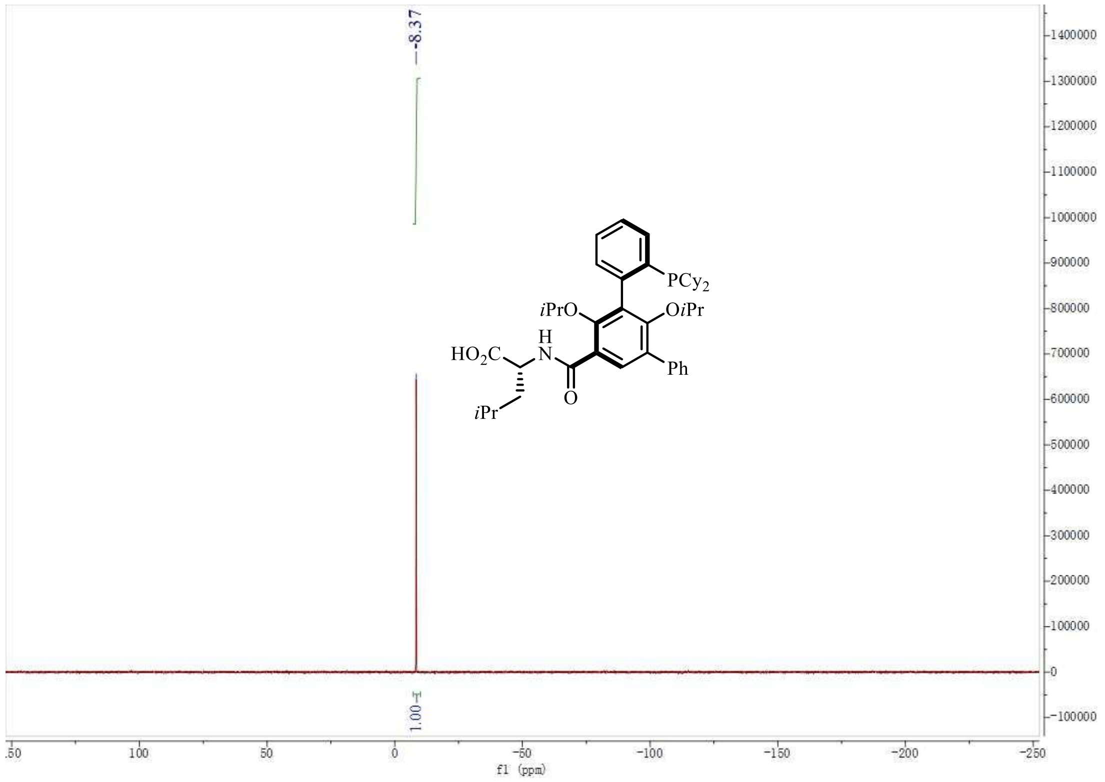

L7-((S)-2-(dicyclohexylphosphaneyl)-2',6'-diisopropoxy-[1,1':3',1''-terphenyl]-5'-carbonyl)-

***D*-valine** ^{1}H NMR (500 NHz, Acetone-d_6)

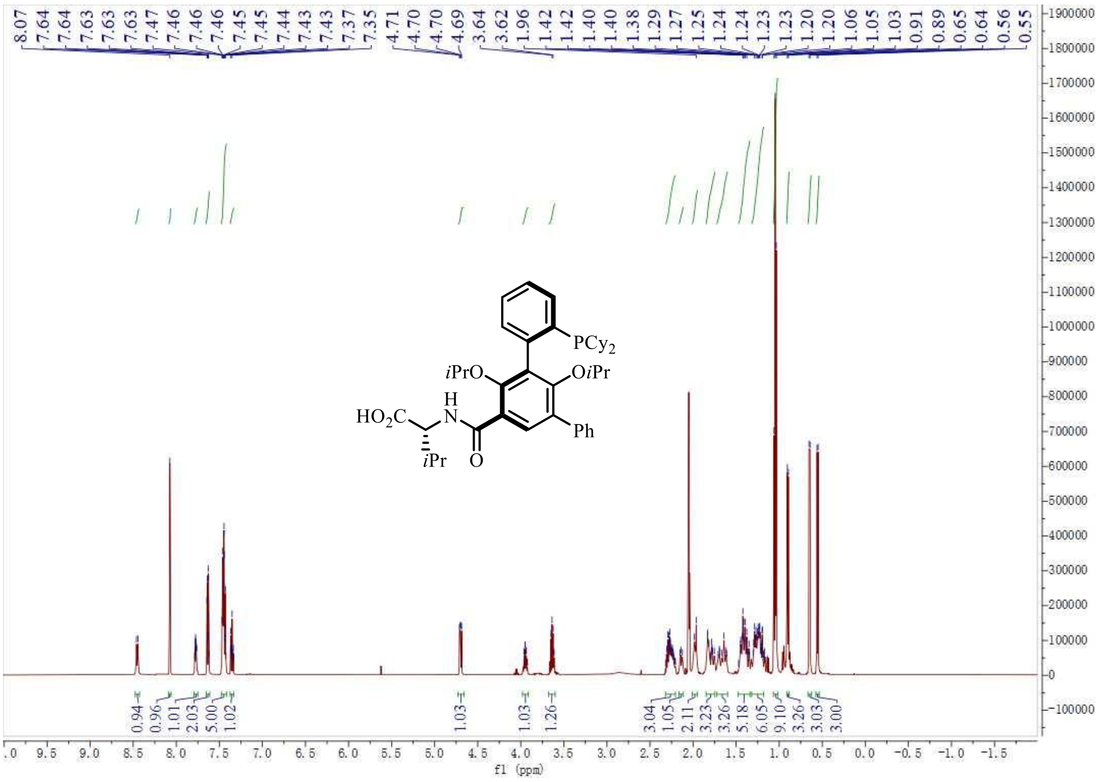

^{13}C NMR (126 NHz, Acetone-d_6)

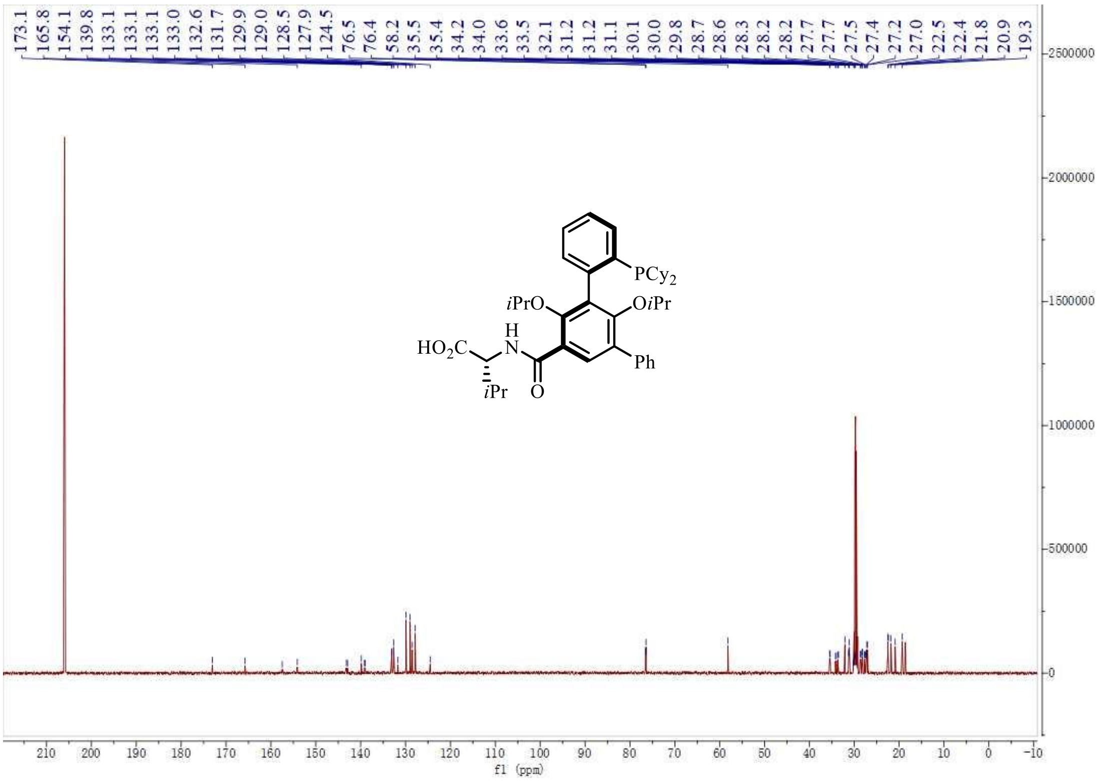

^{31}P NMR (202 NHz, Acetone-d_6)

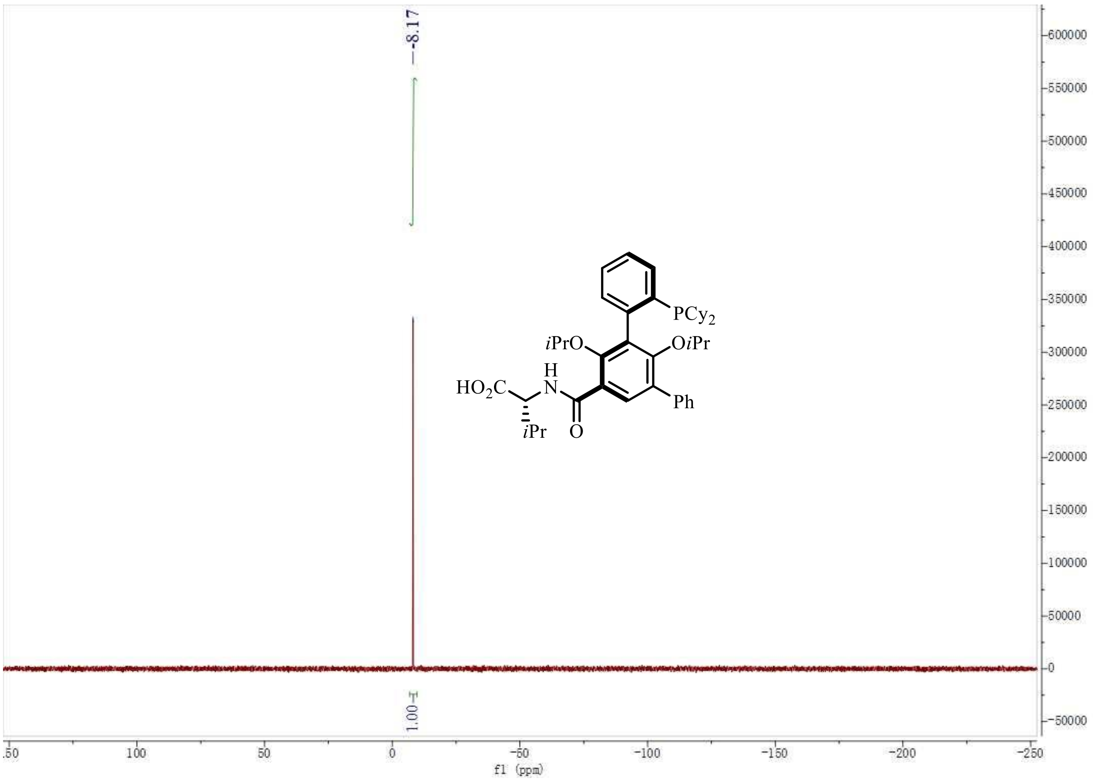

L8-(*R*)-2'-(dicyclohexylphosphaneyl)-2,6-diisopropoxy-5-methyl-[1,1'-biphenyl]-3-carbonyl-*L*-isoleucine ^{1}H NMR (500 MHz, Acetone-d_6)

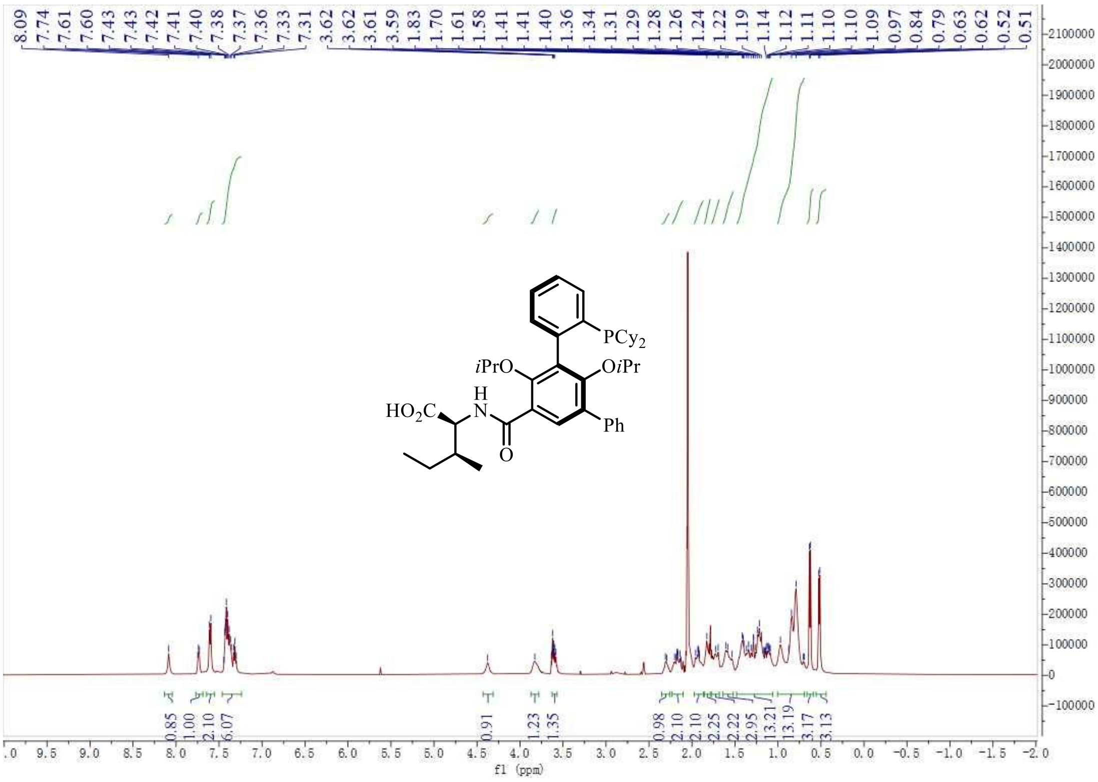

^{13}C NMR (126 MHz, Acetone-d_6)

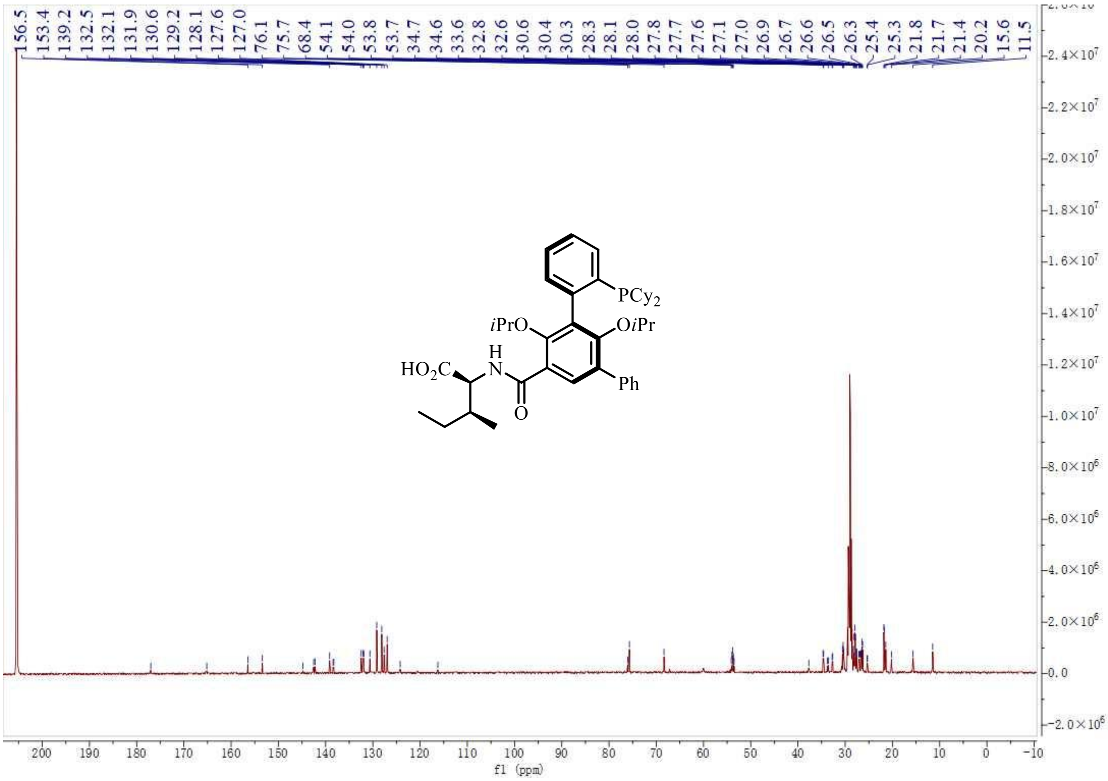

^{31}P NMR (202 MHz, Acetone-d_6)

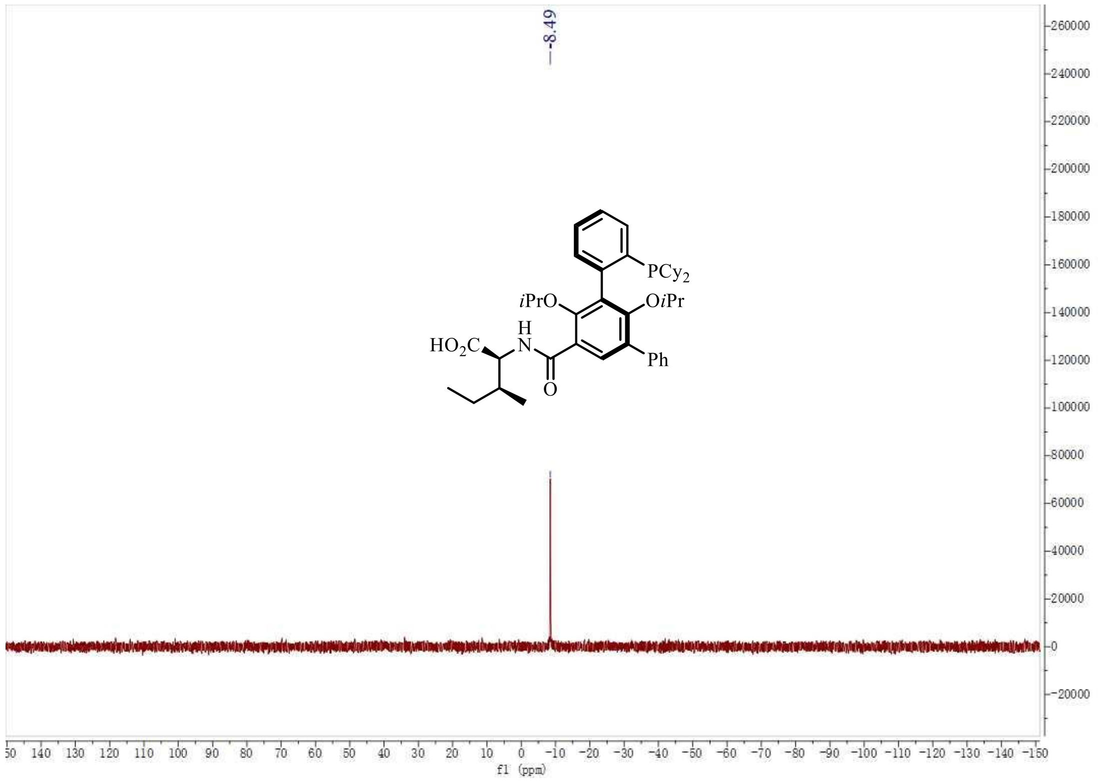

L9-((*S*)-2-(dicyclohexylphosphaneyl)-2',6'-diisopropoxy-[1,1':3',1''-terphenyl]-5'-carbonyl)-*D*-tert-leucine ^{1}H NMR (500 MHz, Acetone-d_6)

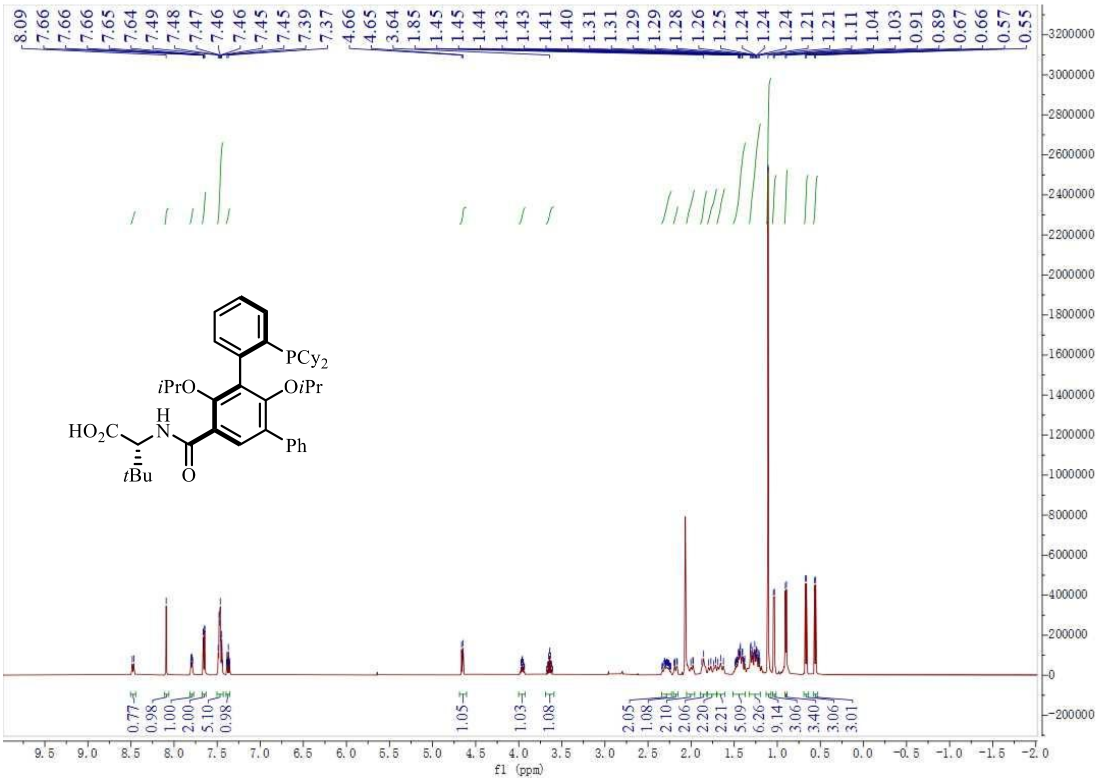

^{13}C NMR (101 MHz, Acetone-d_6)

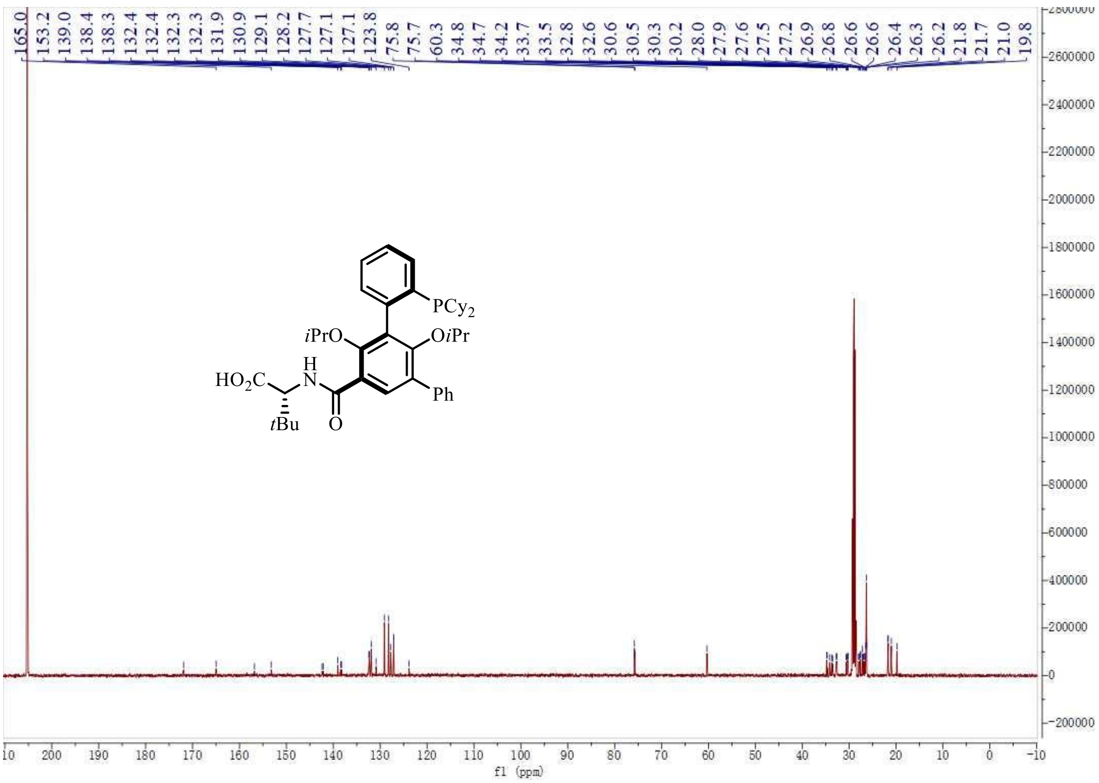

^{31}P NMR (202 MHz, Acetone-d_6)

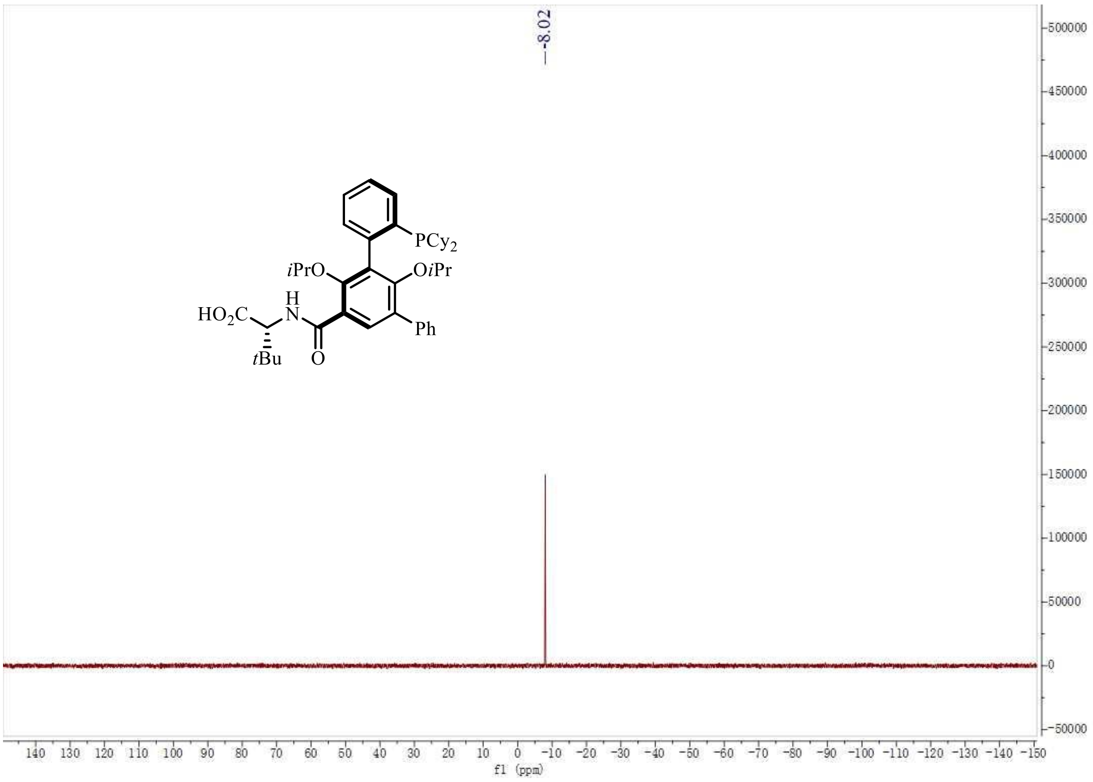

2,7-dichloro-9*H*-carbazole

^{1}H NMR (500 MHz, Chloroform-*d*)

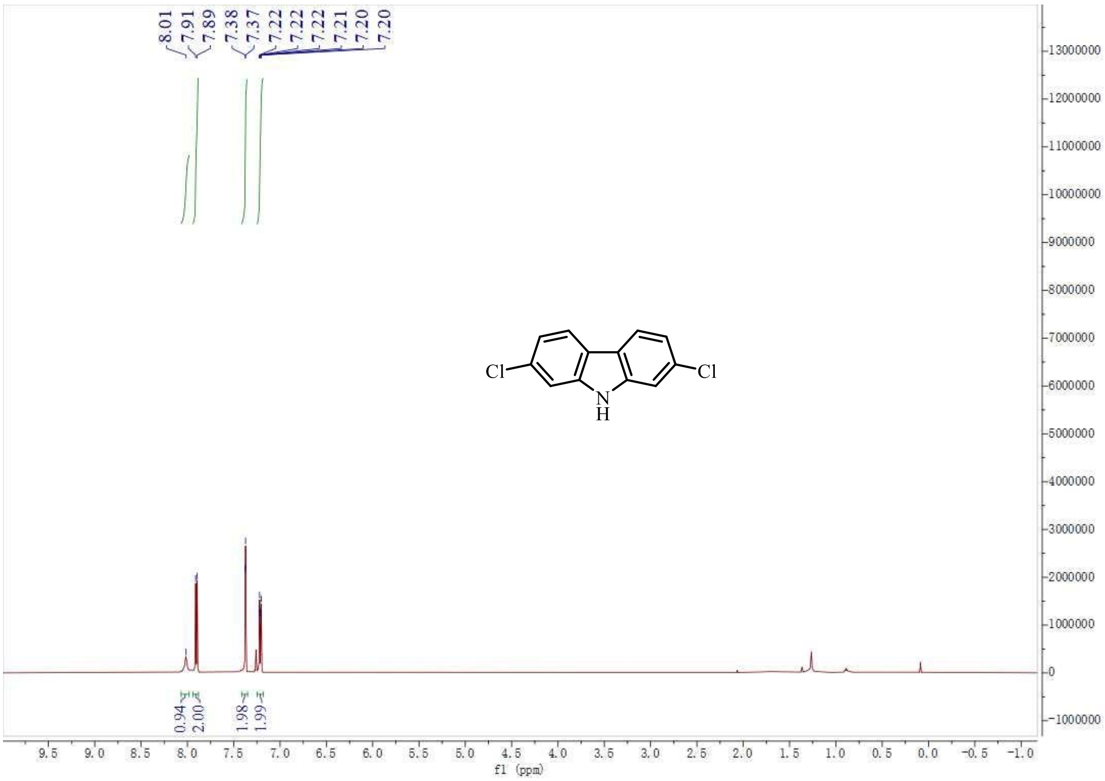

2-(2,7-dichloro-9*H*-carbazol-9-yl)-3-methylbenzoic acid (1)

^{1}H NMR (500 MHz, Chloroform-*d*)

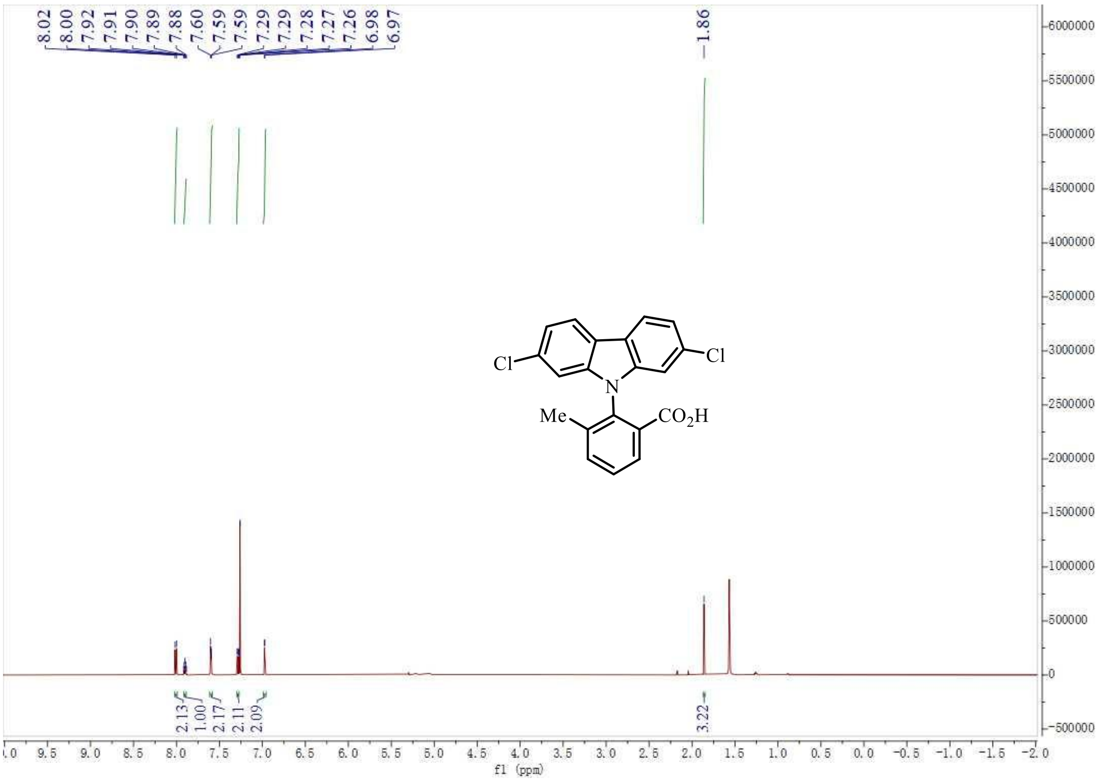

^{13}C NMR (126 MHz, Chloroform-*d*)

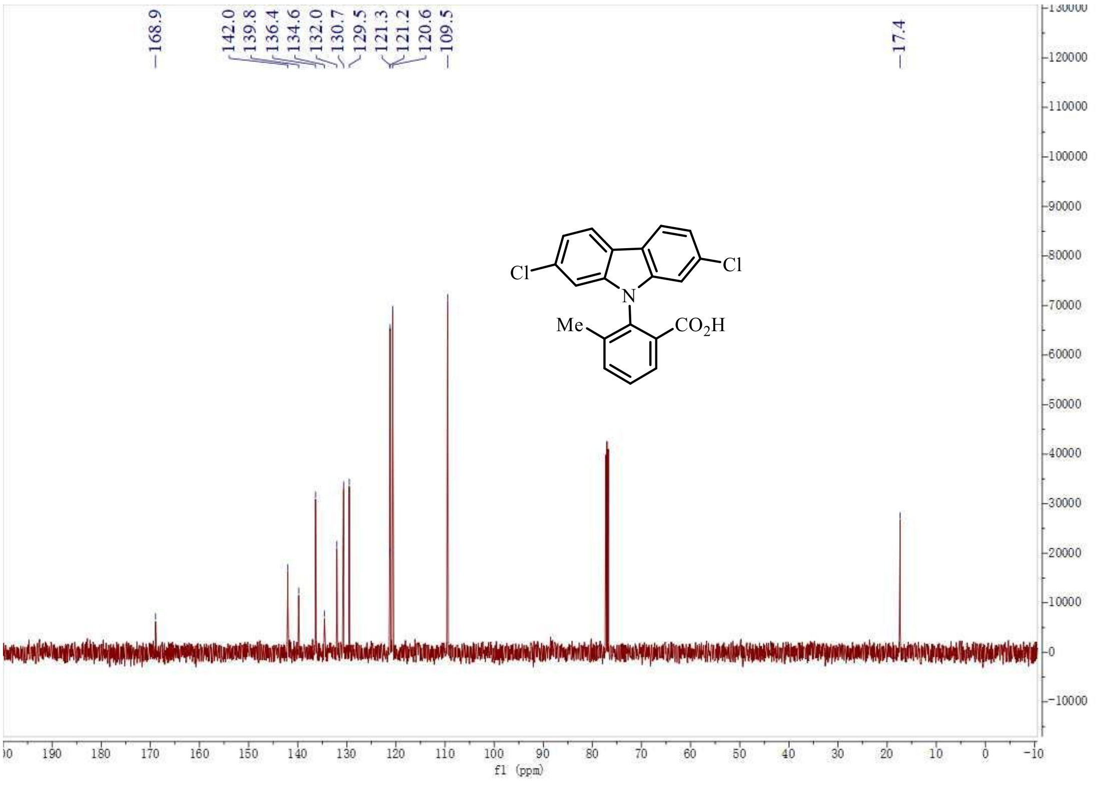

Ethyl 2-(2,7-dichloro-9*H*-carbazol-9-yl)-3-methylbenzoate (SI-1)

^{1}H NMR (500 MHz, Chloroform-*d*)

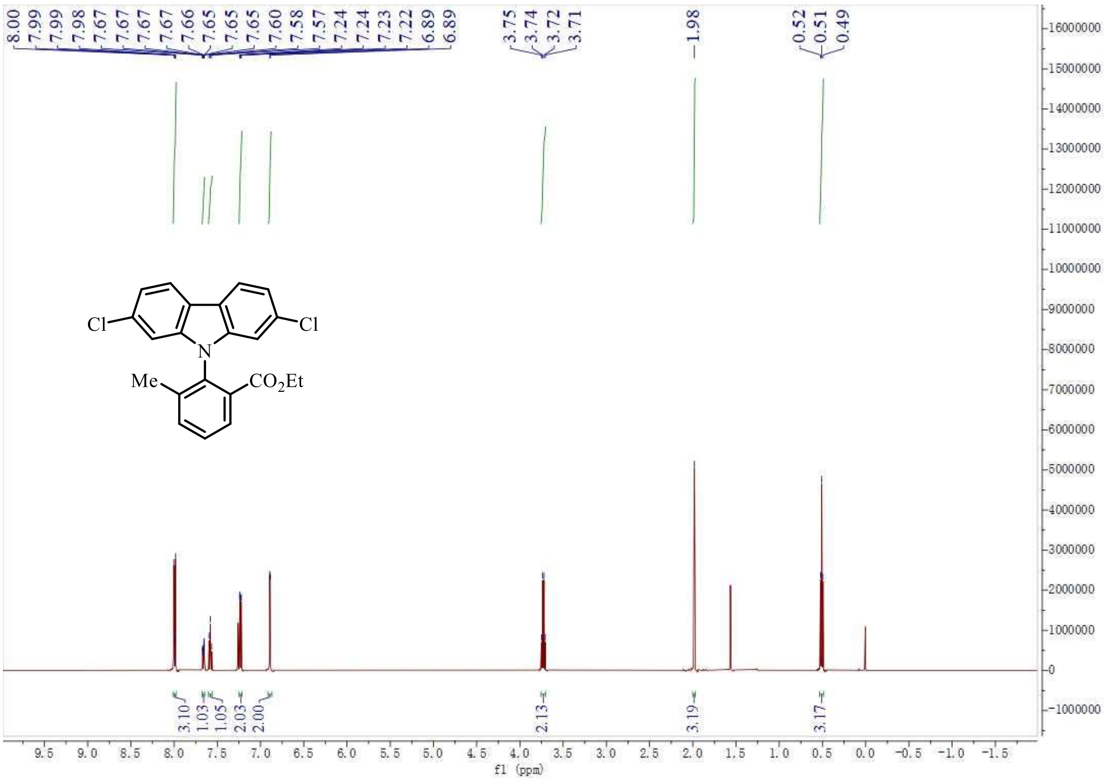

^{13}C NMR (101 MHz, Chloroform-*d*)

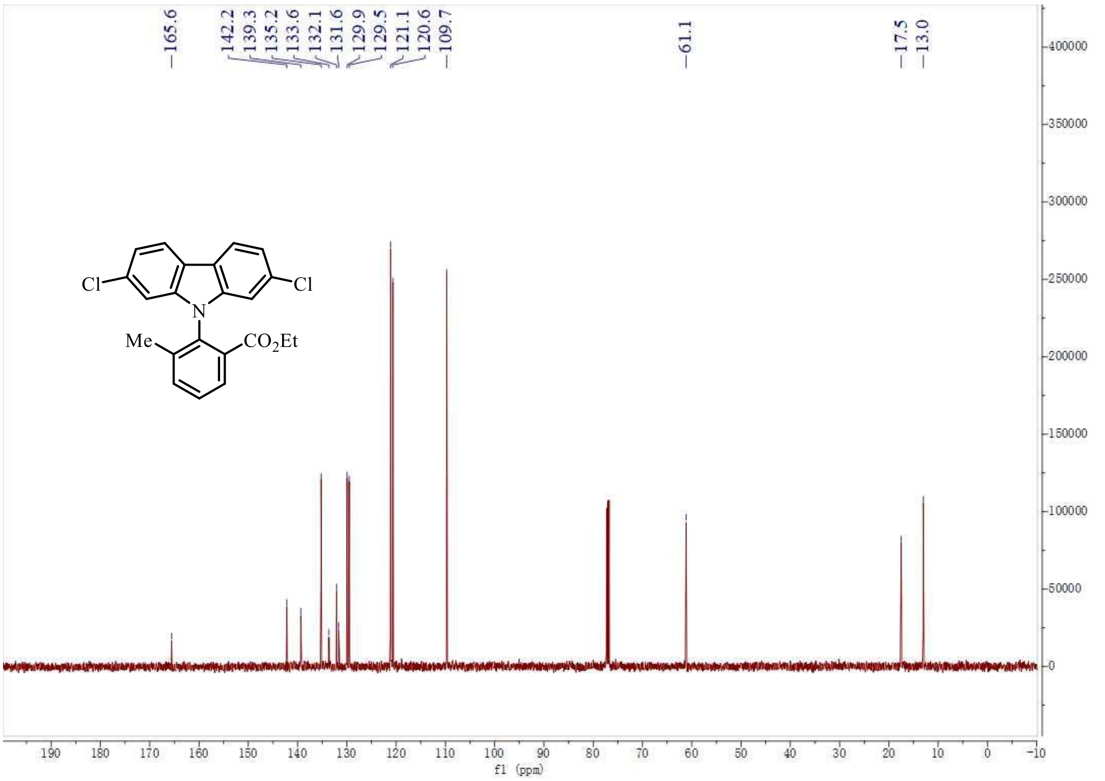

2-(2,7-dichloro-3,6-dimethoxy-9*H*-carbazol-9-yl)-3-methylbenzoic acid

^{1}H NMR (500 MHz, Chloroform-*d*)

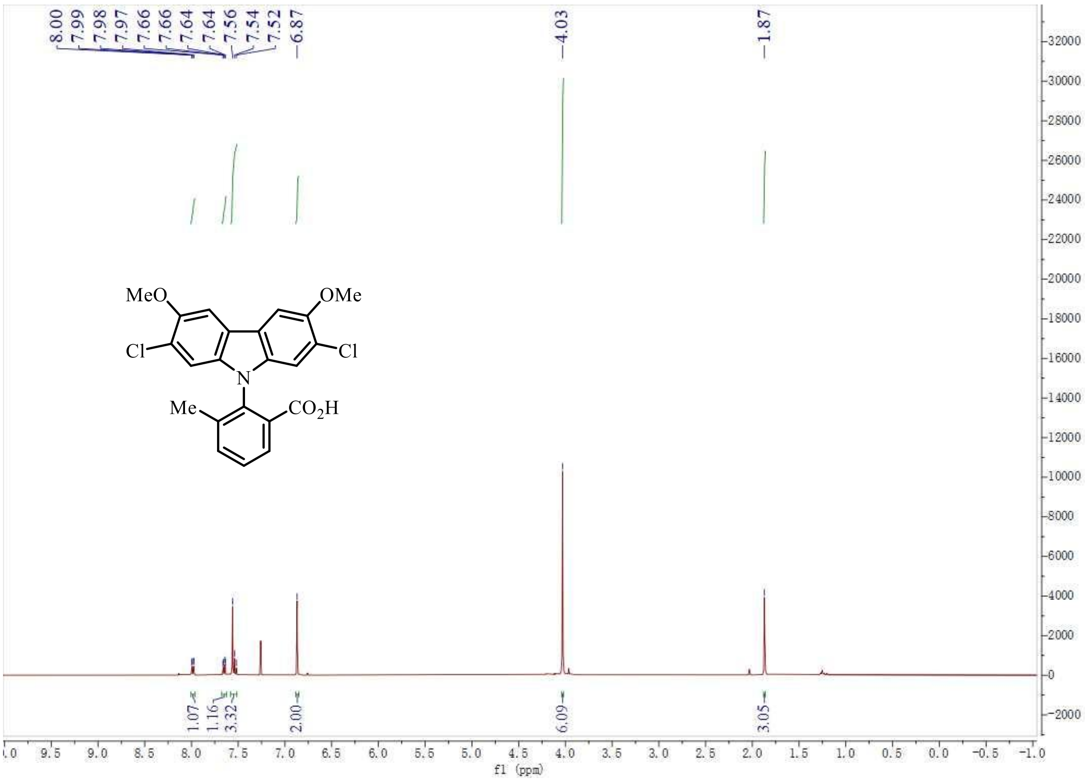

^{13}C NMR (101 MHz, Chloroform-*d*)

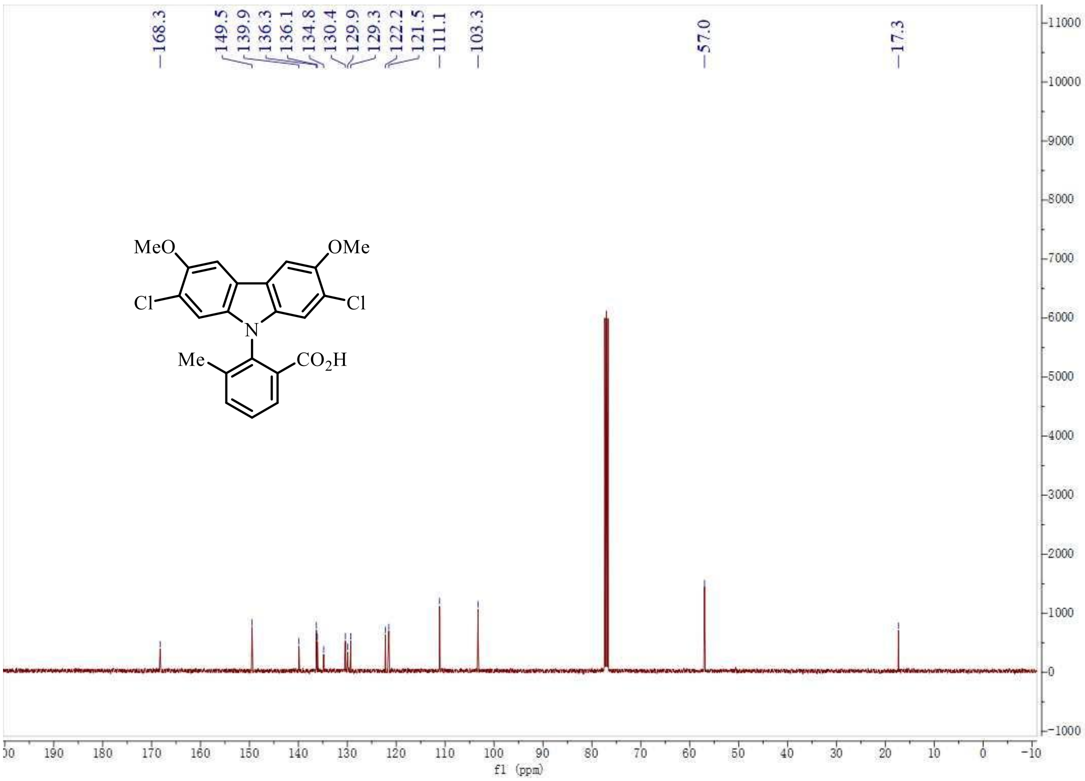

2-(2,7-dichloro-9H-carbazol-9-yl)-3'-methoxy-[1,1'-biphenyl]-3-carboxylic acid

^{1}H NMR (500 MHz, Chloroform-d)

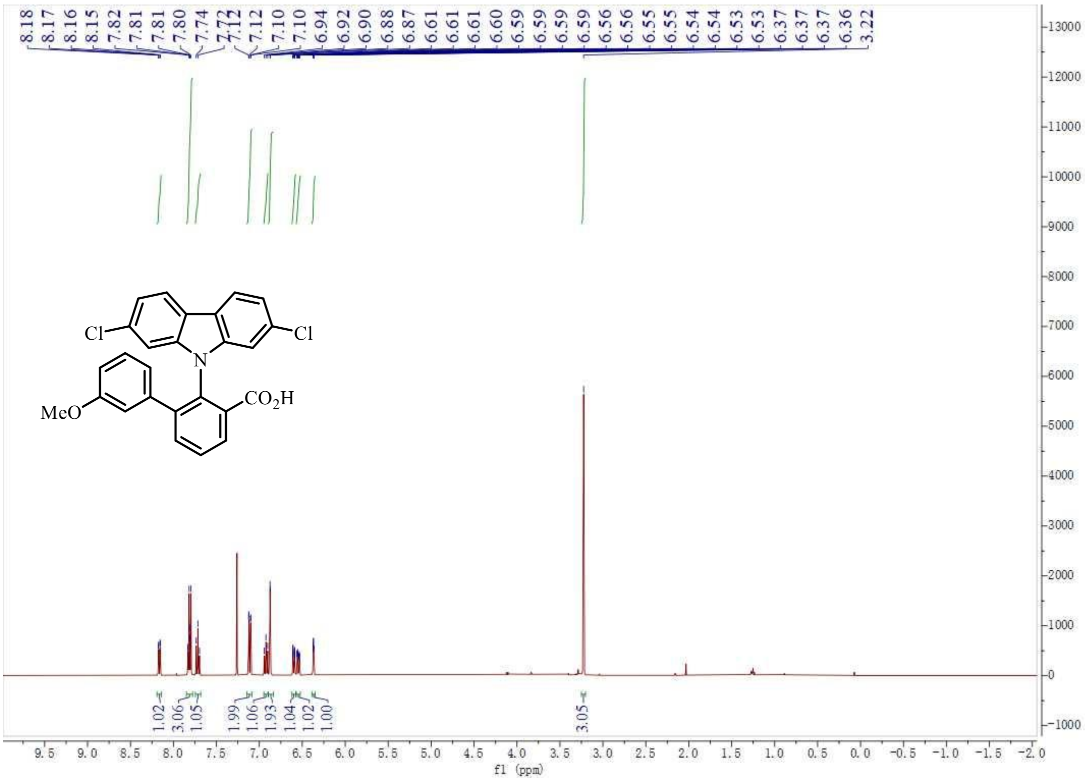

^{13}C NMR (126 MHz, Chloroform-d)

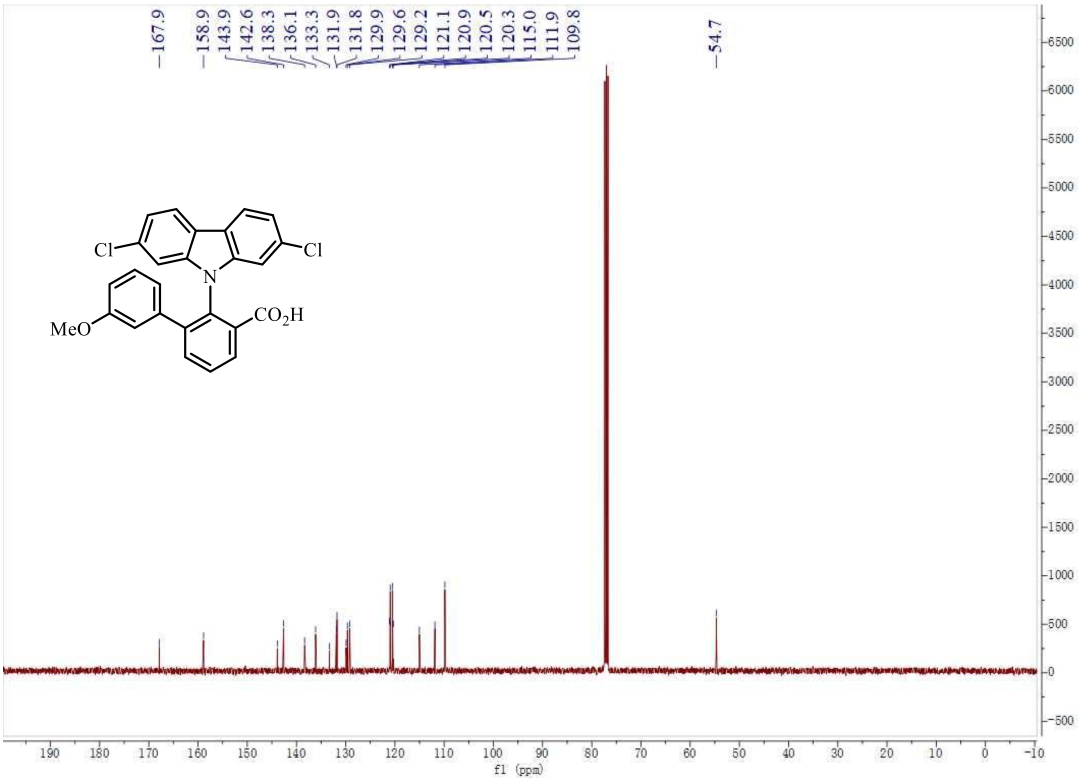

2-(2,7-dichloro-9*H*-carbazol-9-yl)-3'-nitro-[1,1'-biphenyl]-3-carboxylic acid

^{1}H NMR (500 MHz, Chloroform-*d*)

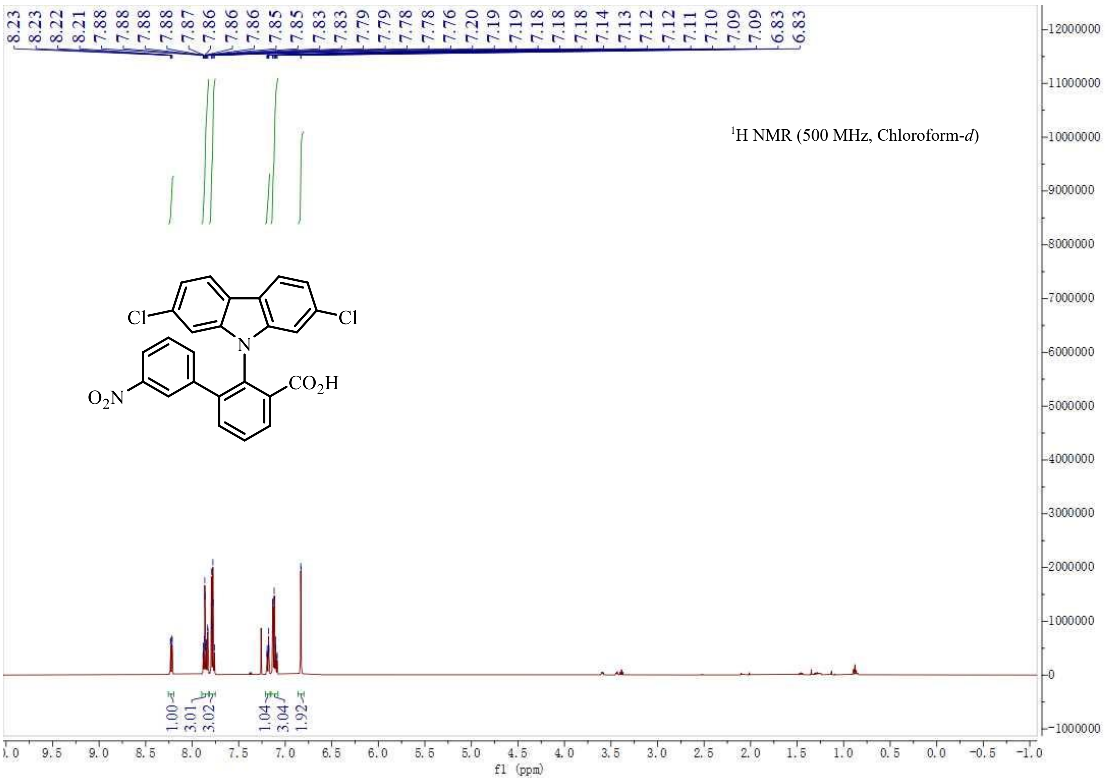

^{13}C NMR (126 MHz, Chloroform-*d*)

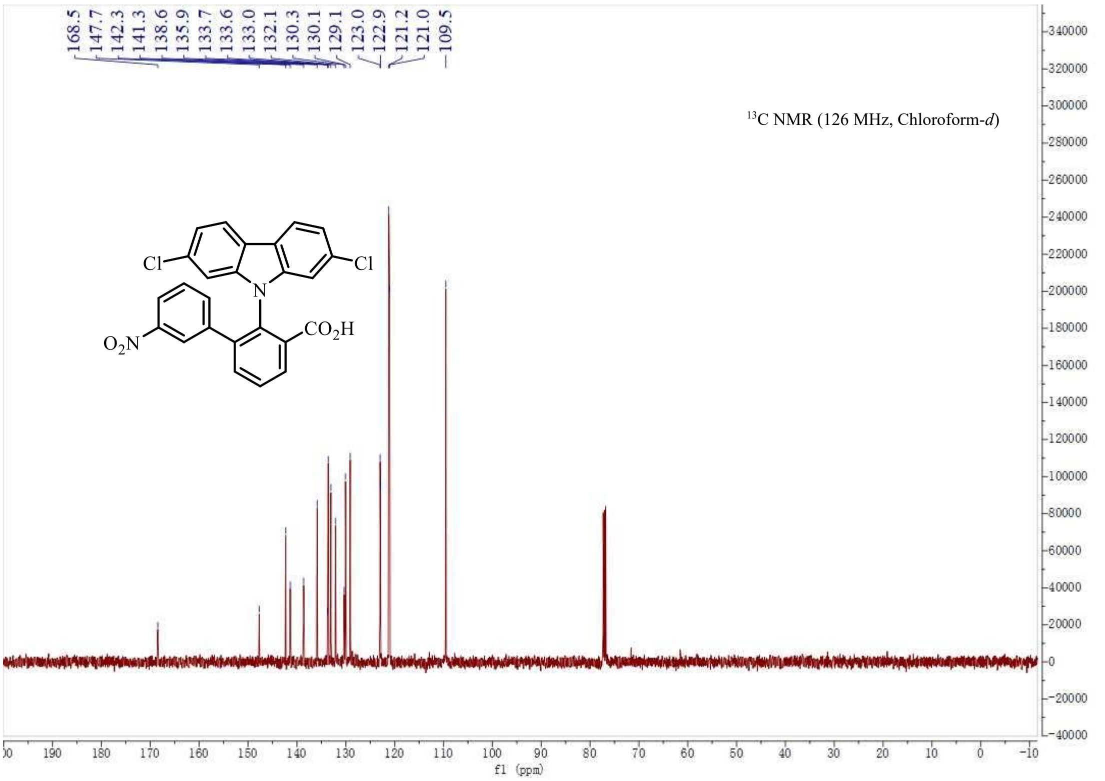

2-(2,7-dichloro-9*H*-carbazol-9-yl)-2',3',4',5'-tetrahydro-[1,1'-biphenyl]-3-carboxylic acid

^{1}H NMR (500 MHz, Chloroform-*d*)

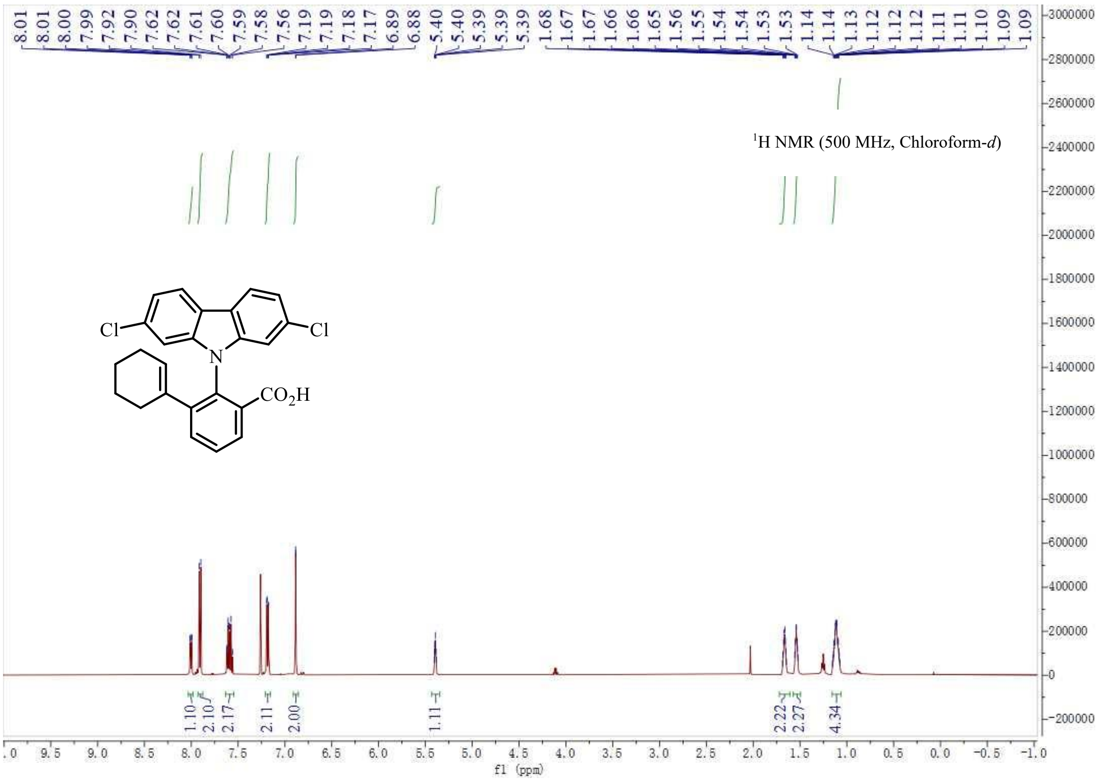

^{13}C NMR (126 MHz, Chloroform-*d*)

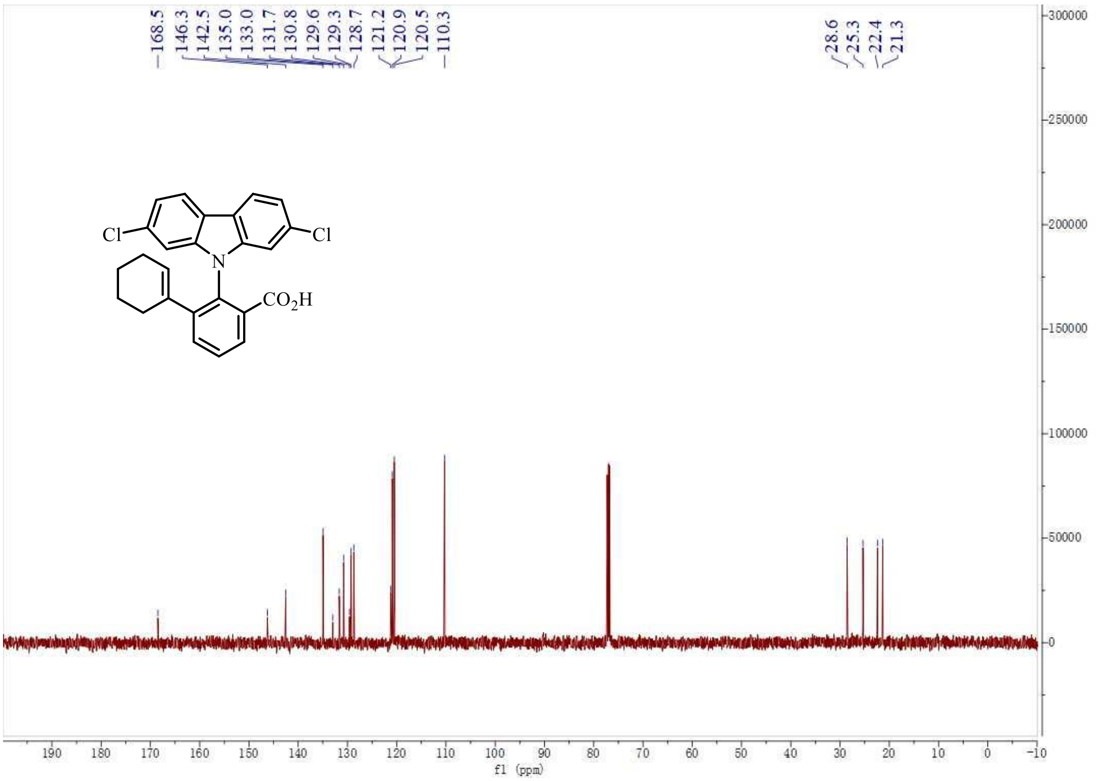

2-(2,7-dichloro-9*H*-carbazol-9-yl)-3-(3,6-dihydro-2*H*-pyran-4-yl)benzoic acid

^{1}H NMR (500 MHz, Chloroform-*d*)

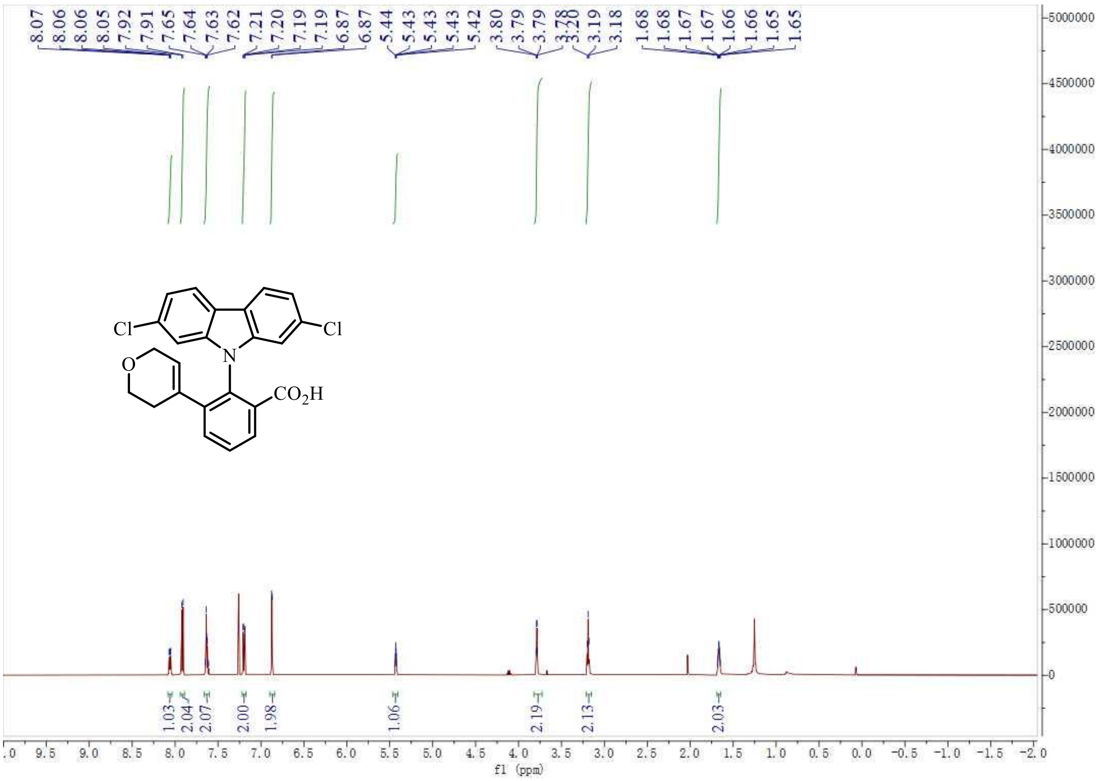

^{13}C NMR (126 MHz, Chloroform-*d*)

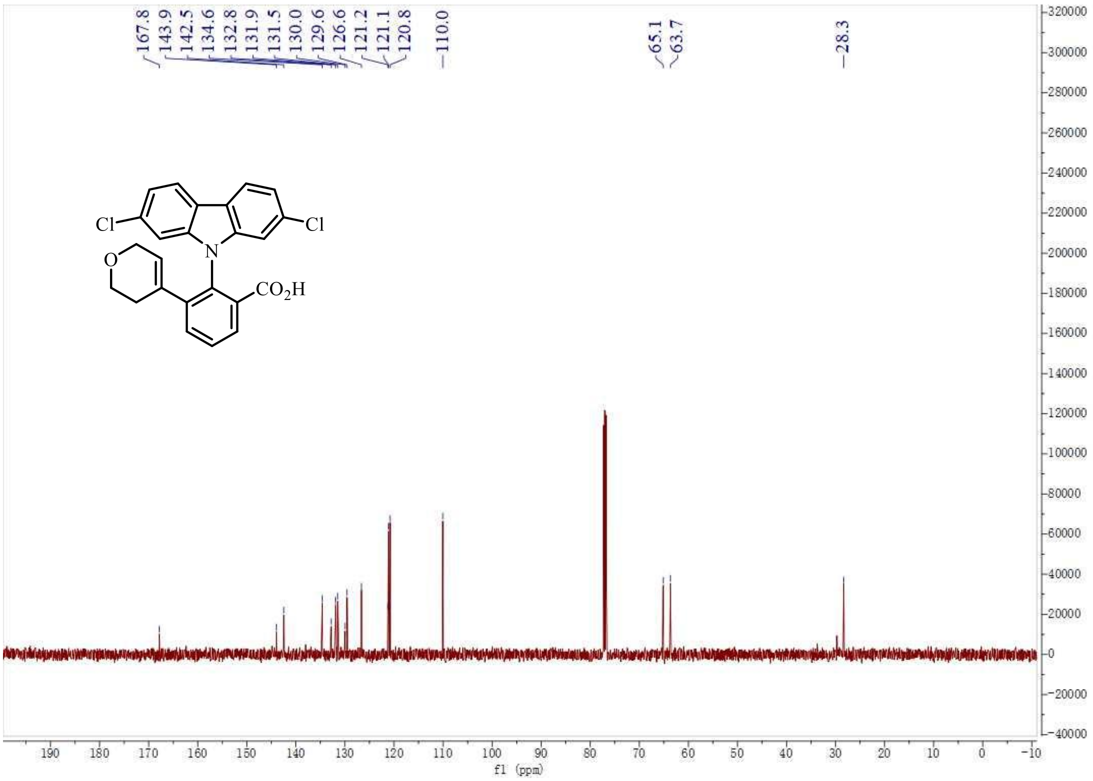

2-(2,7-dichloro-9*H*-carbazol-9-yl)-3-(naphthalen-1-yl)benzoic acid

^{1}H NMR (500 MHz, MeOD)

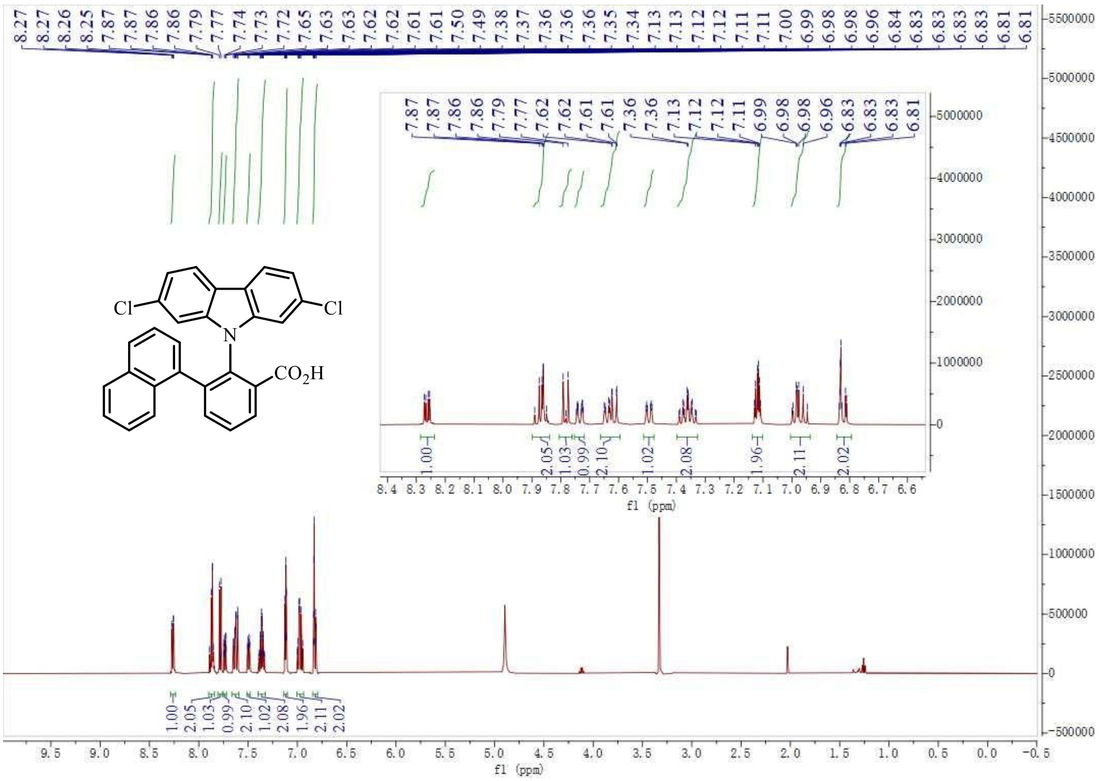

^{13}C NMR (126 MHz, MeOD)

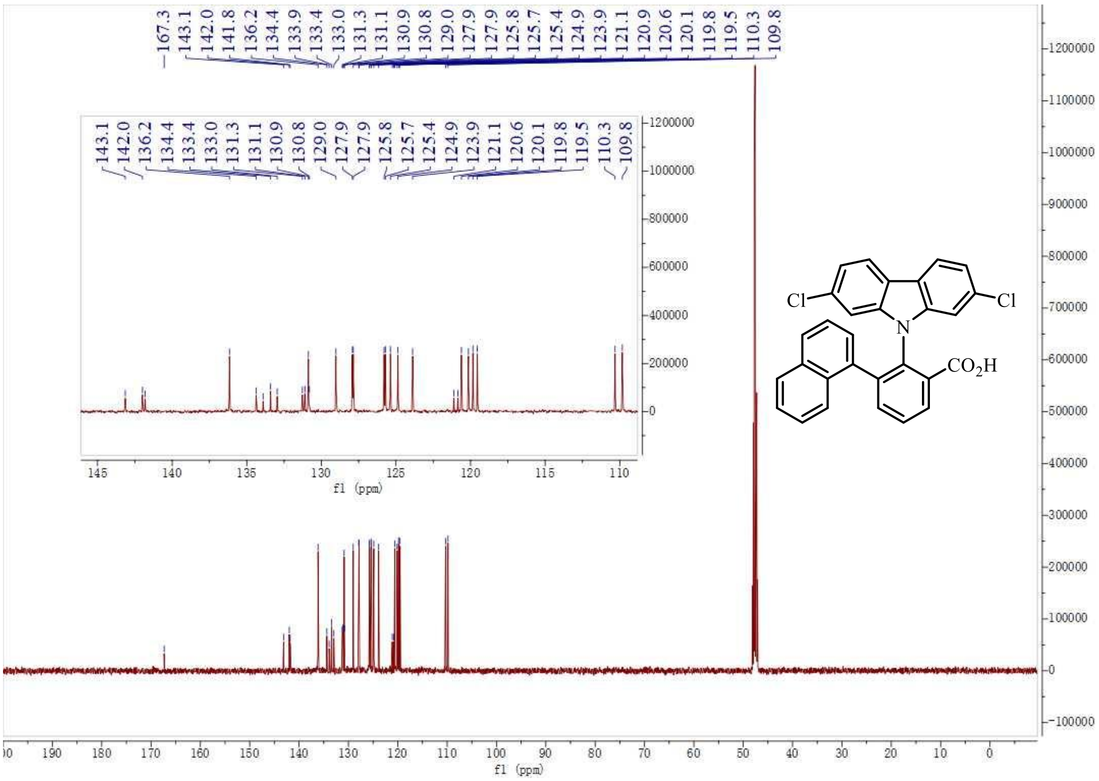

3-chloro-2-(2,7-dichloro-9*H*-carbazol-9-yl)benzoic acid (63)

^{1}H NMR (500 MHz, Acetone-d_6)

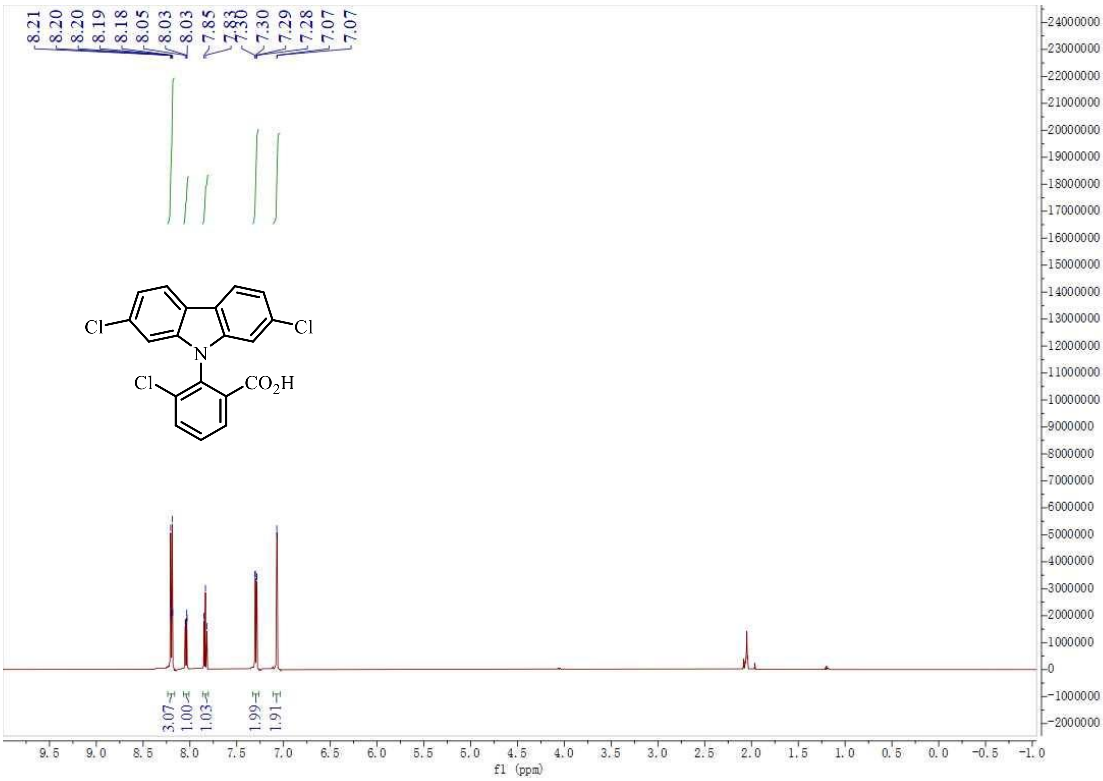

^{13}C NMR (126 MHz, Acetone-d_6)

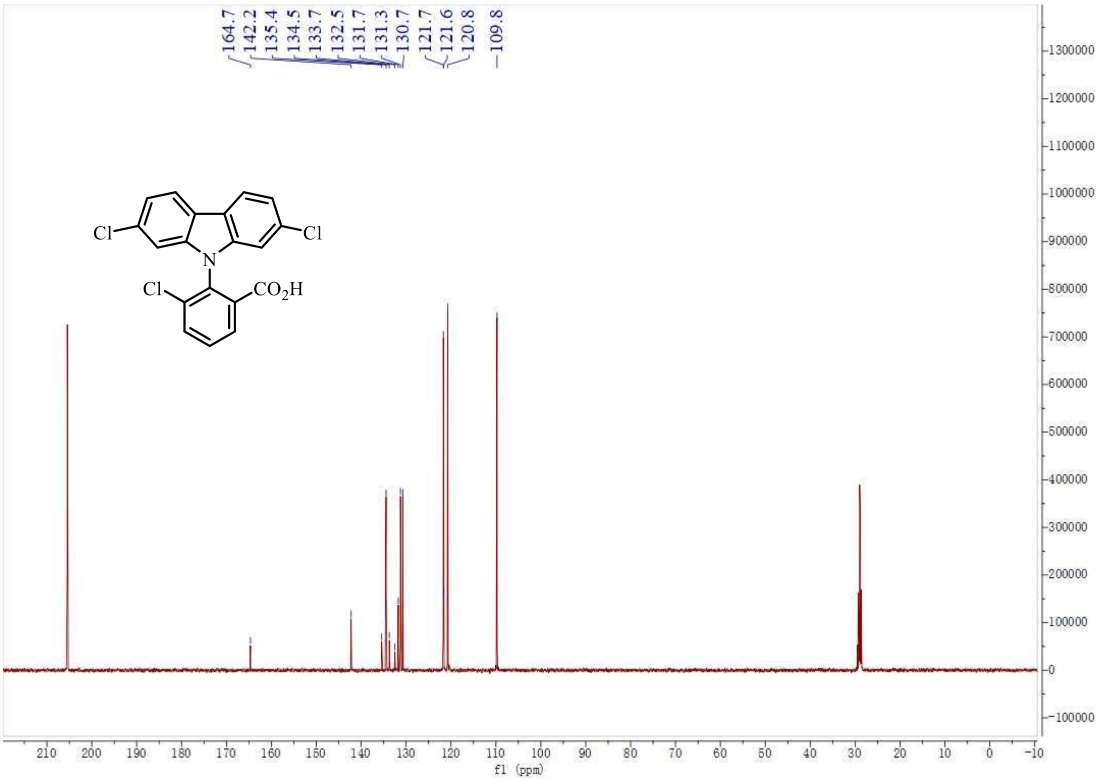

2-(2,7-dichloro-9*H*-carbazol-9-yl)benzoic acid (SI-**2**)

^{1}H NMR (500 MHz, Acetone-d_6)

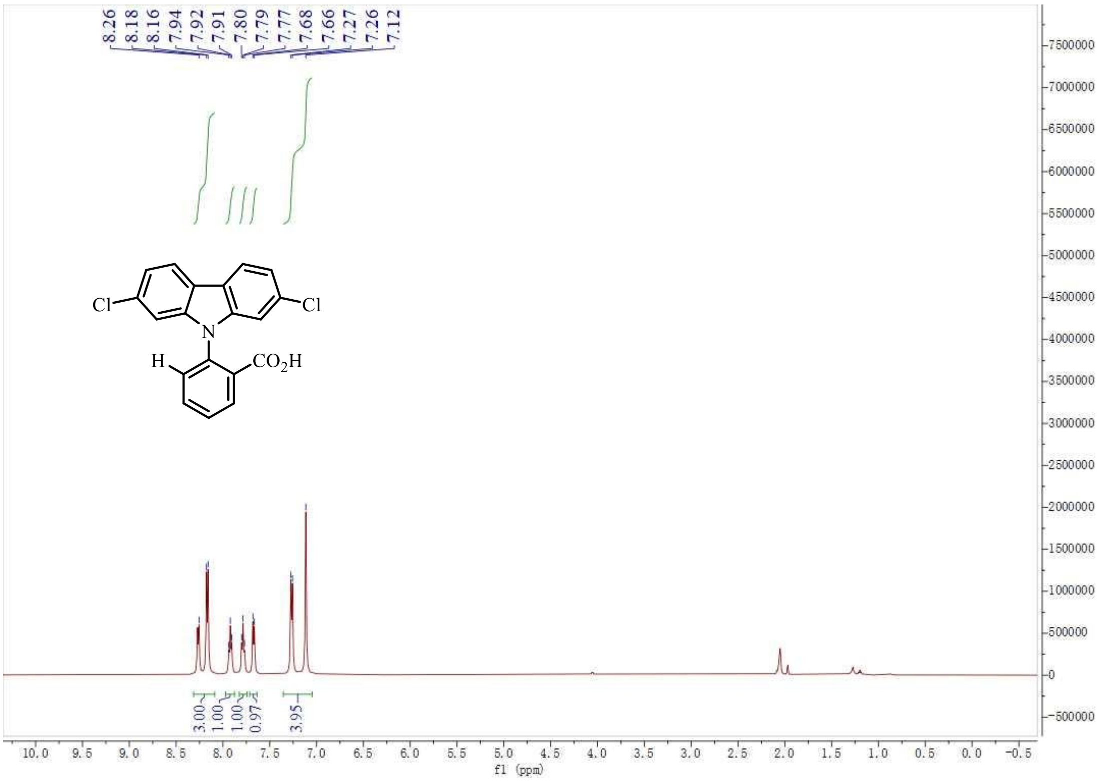

^{13}C NMR (126 MHz, Acetone-d_6)

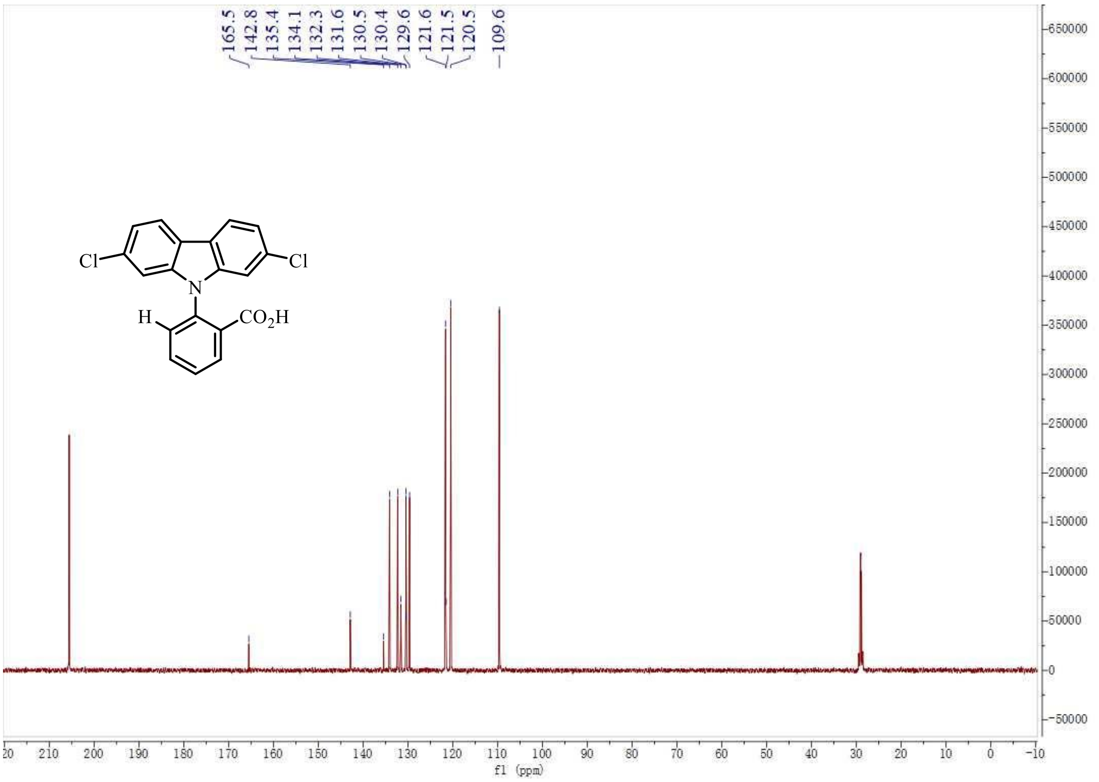

2-(2,7-dibromo-9H-carbazol-9-yl)-3-methylbenzoic acid (80)

^{1}H NMR (500 MHz, Chloroform-d)

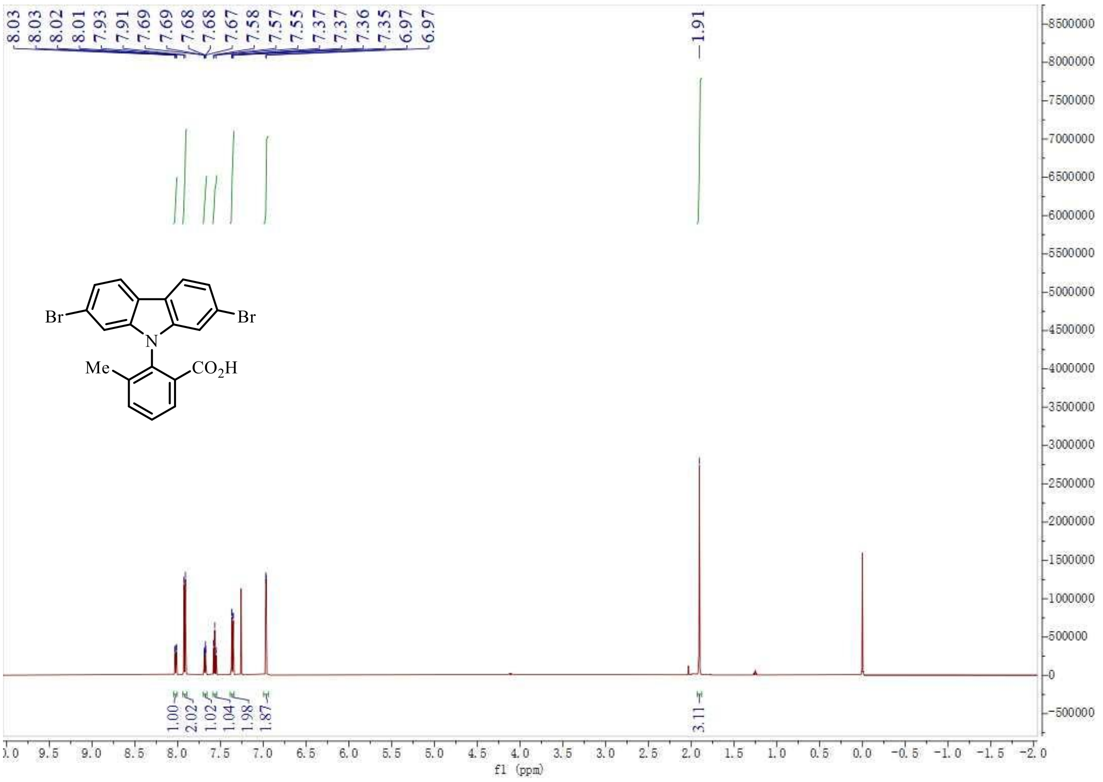

^{13}C NMR (126 MHz, Chloroform-d)

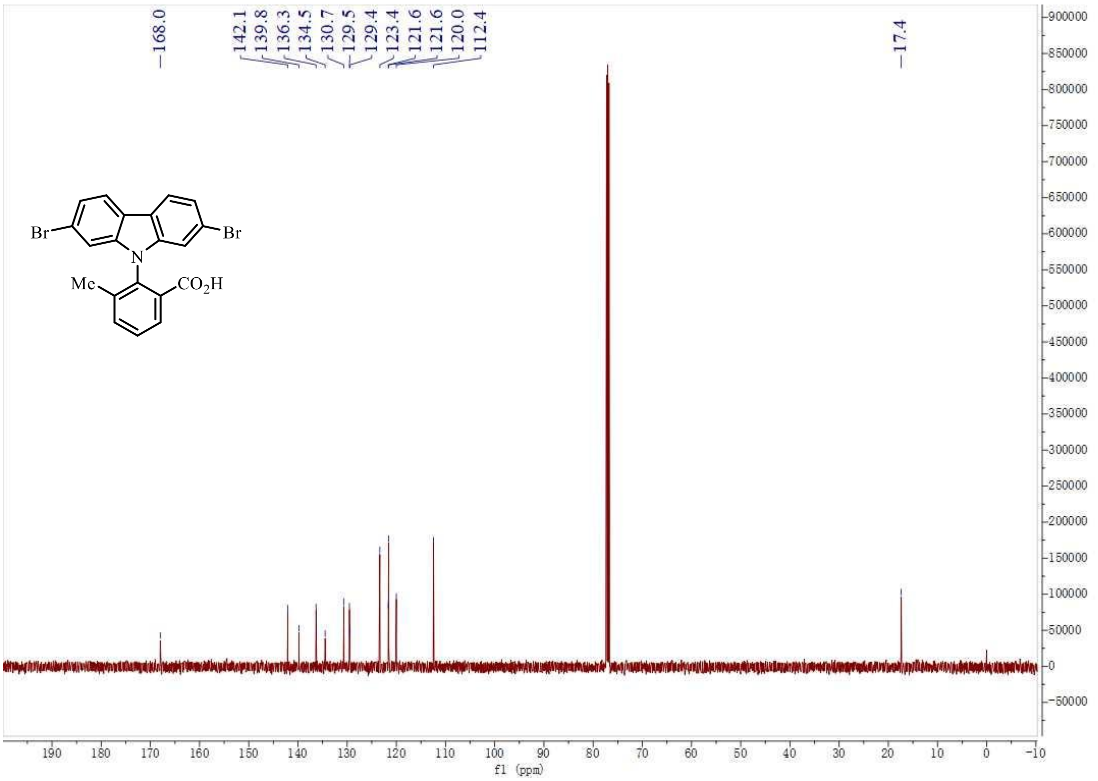

2-(3,6-dichloro-9*H*-carbazol-9-yl)-3-methylbenzoic acid

^{1}H NMR (500 MHz, Chloroform-*d*)

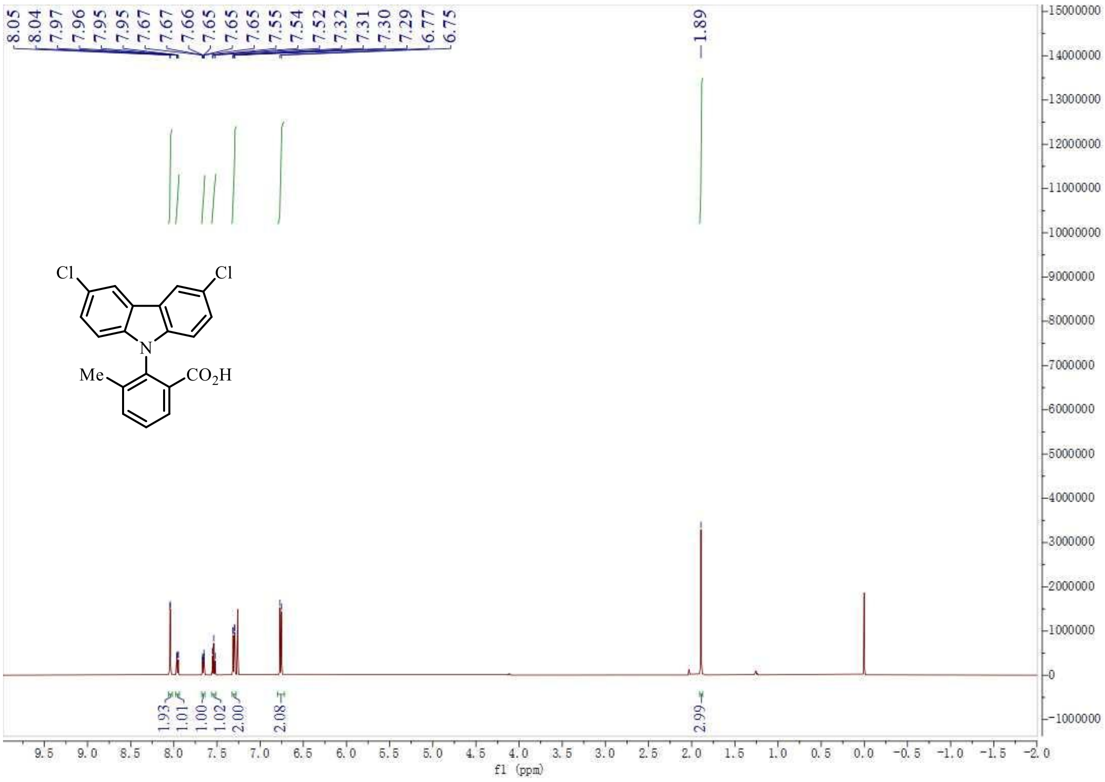

^{13}C NMR (126 MHz, Chloroform-*d*)

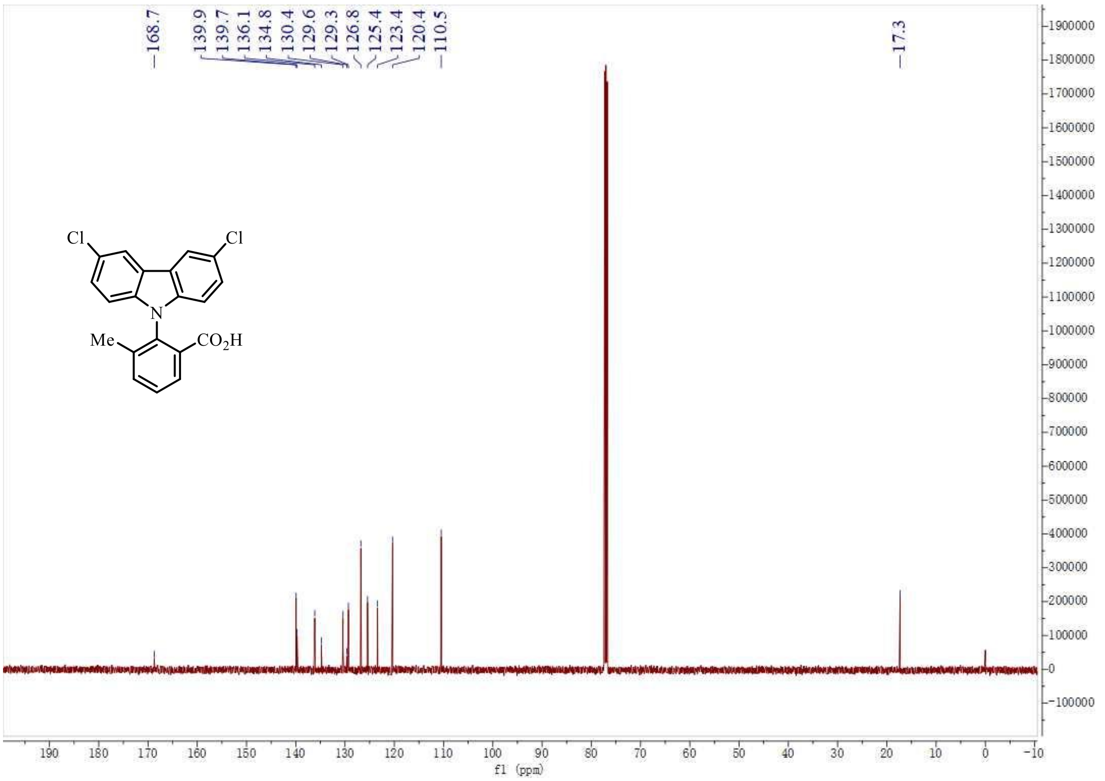

3,6-dichloro-9,9-dimethyl-9,10-dihydroacridine

^{1}H NMR (400 MHz, Chloroform-*d*)

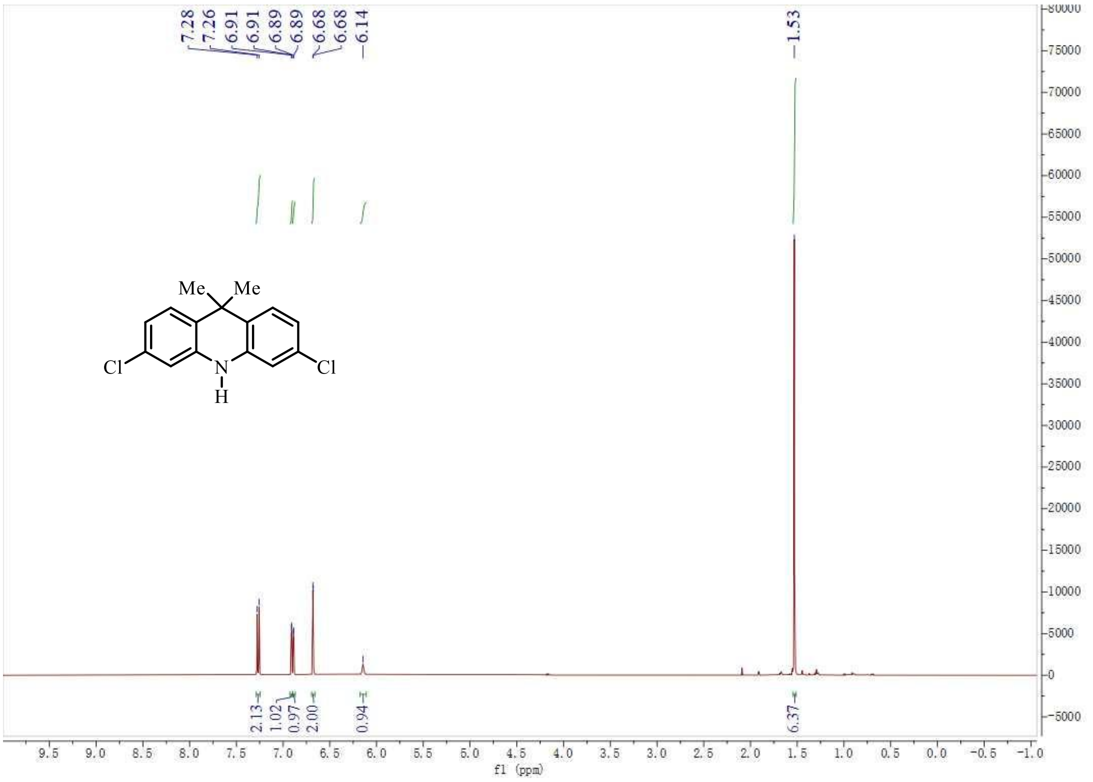

^{13}C NMR (101 MHz, Chloroform-*d*)

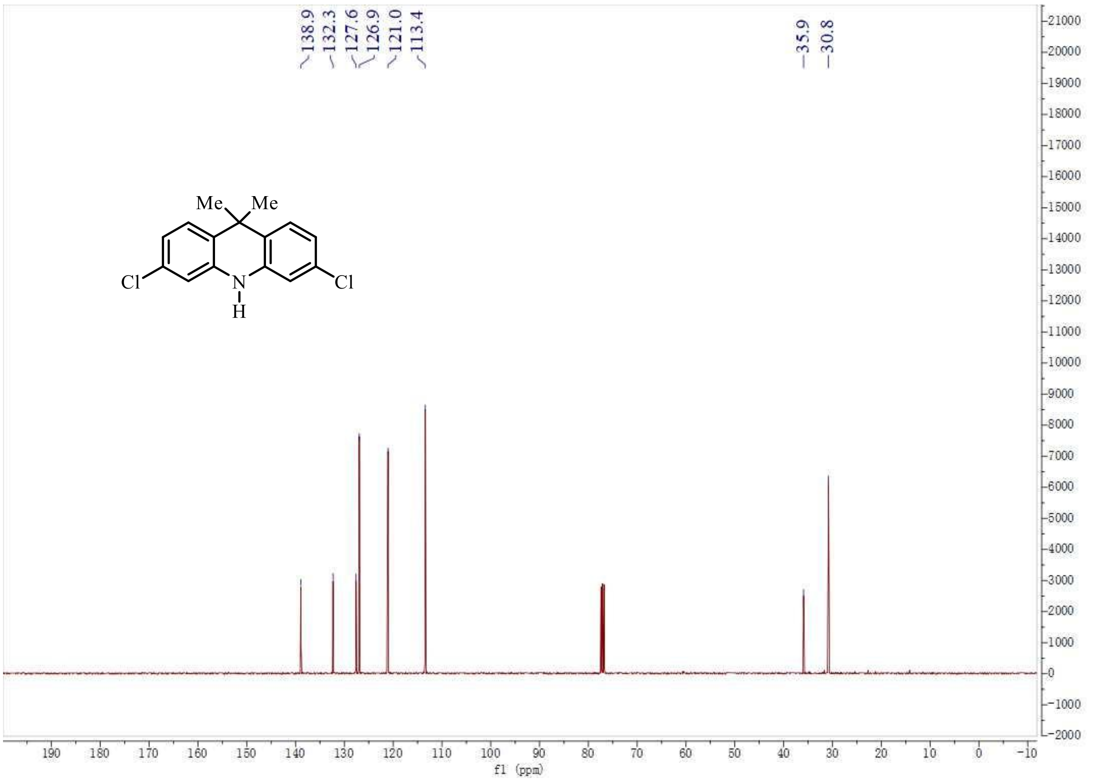

3-chloro-2-(3,6-dichloro-9,9-dimethylacridin-10(9*H*)-yl)benzoic acid

^{1}H NMR (500 MHz, Chloroform-*d*)

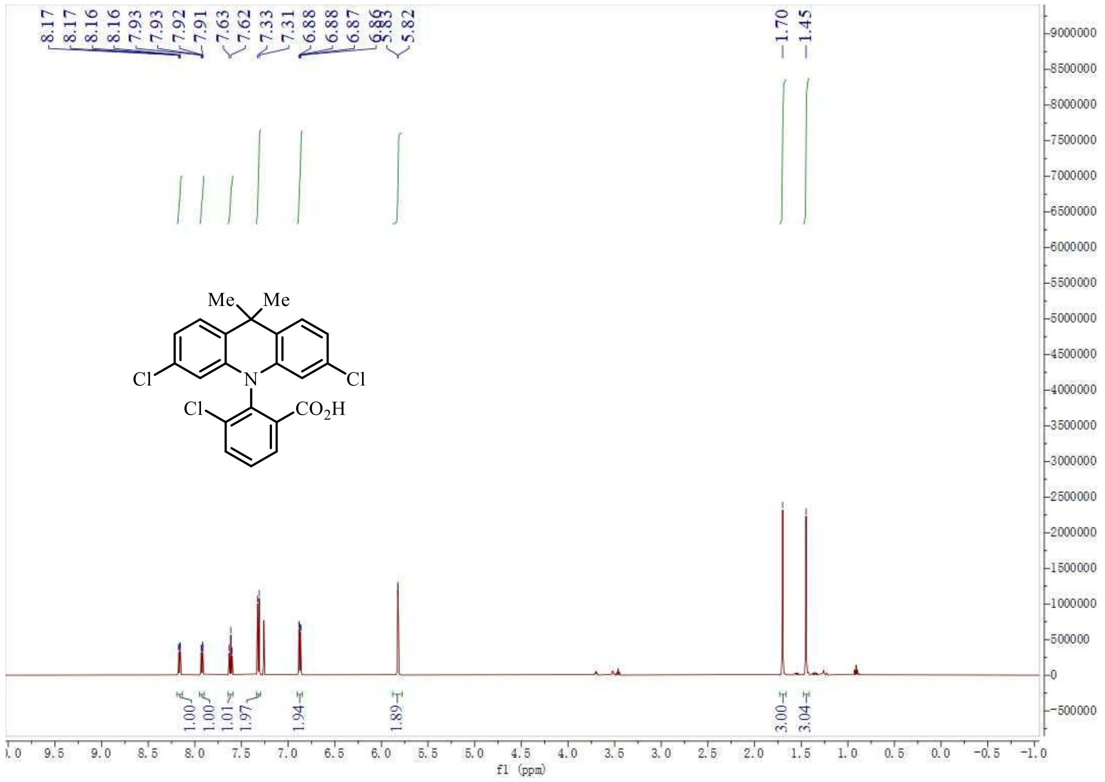

^{13}C NMR (126 MHz, Chloroform-*d*)

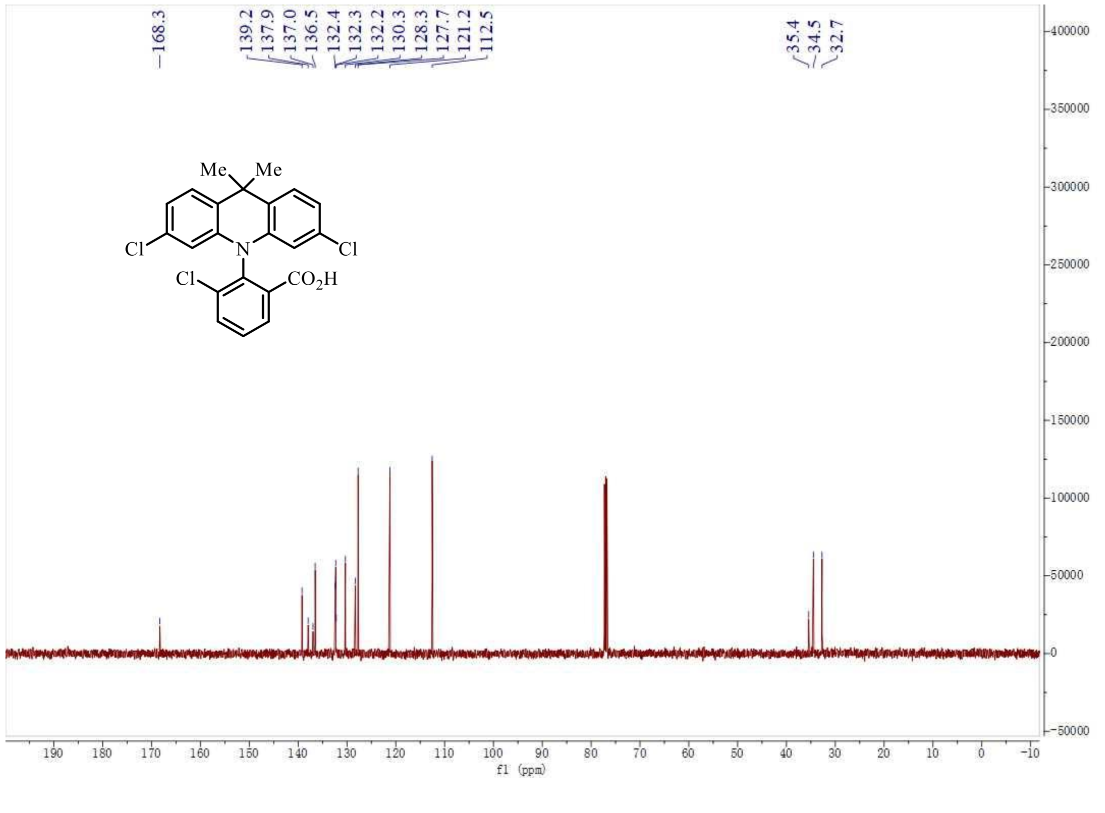

2,8-dichloro-10*H*-phenoxazine

^{1}H NMR (400 MHz, Acetonitrile-d_3)

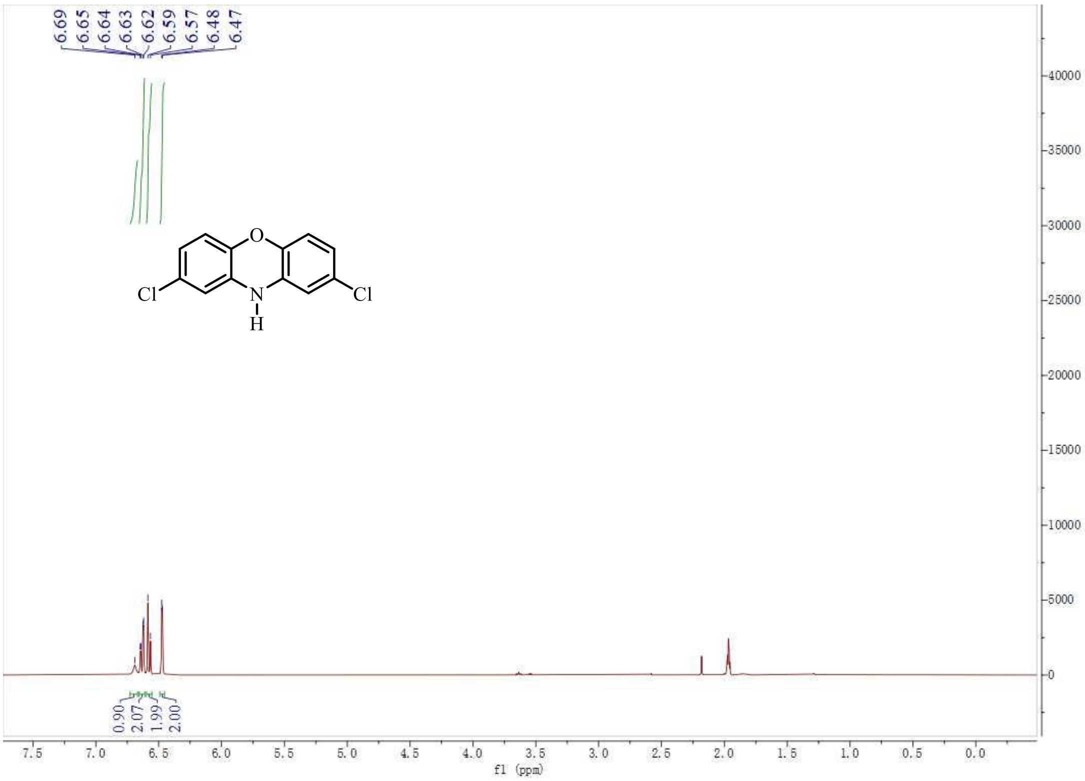

^{13}C NMR (101 MHz, Acetonitrile-d_3)

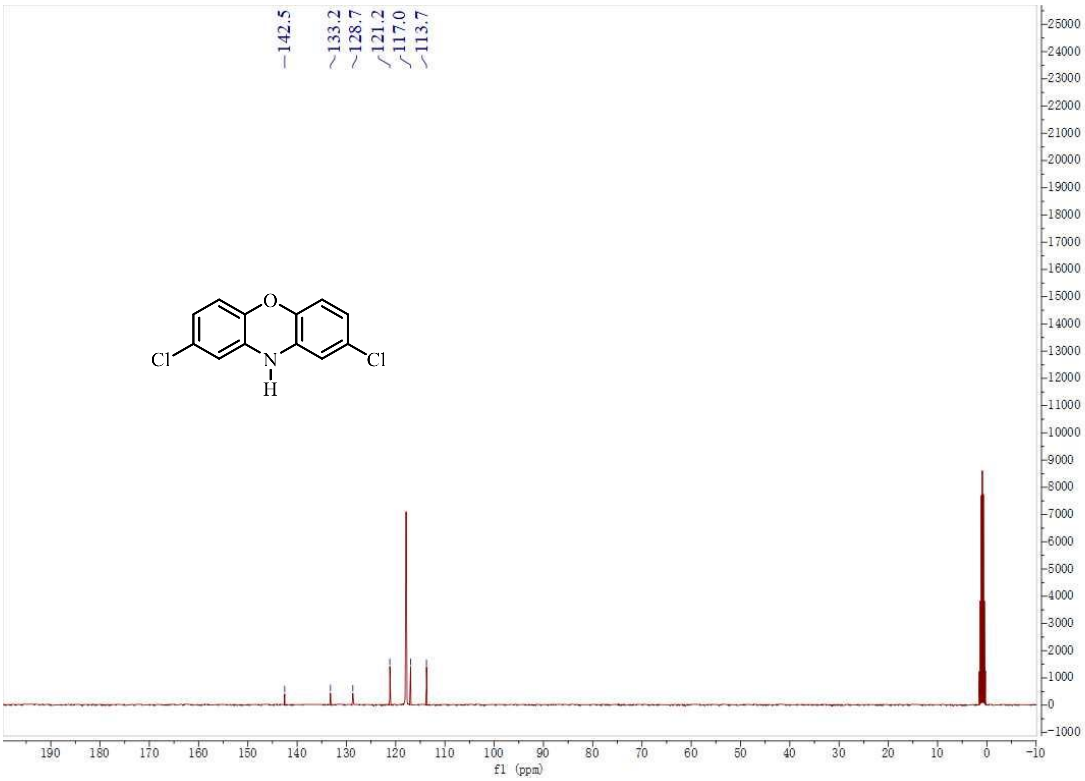

3-chloro-2-(2,8-dichloro-10*H*-phenoxazin-10-yl)benzoic acid

^{1}H NMR (500 MHz, Methanol-*d*)

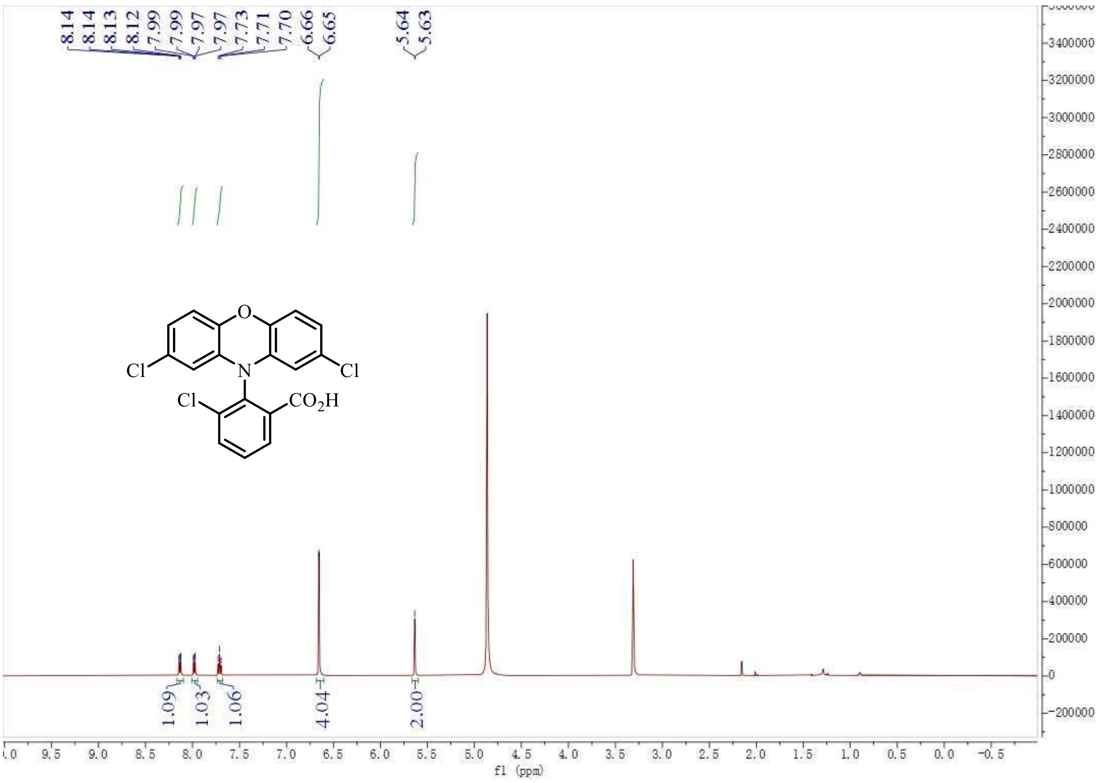

^{13}C NMR (126 MHz, Tetrahydrofuran-d_8)

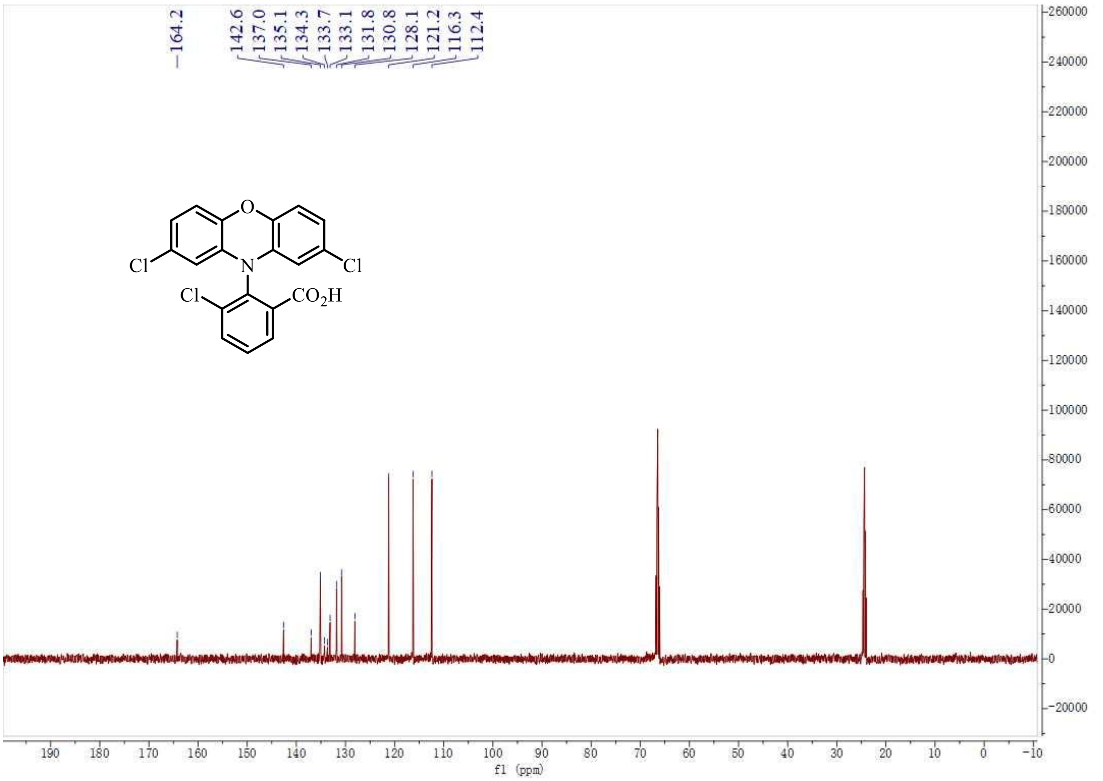

2,7-dichloro-9*H*-carbazol-9-amine

^{1}H NMR (500 MHz, Chloroform-*d*)

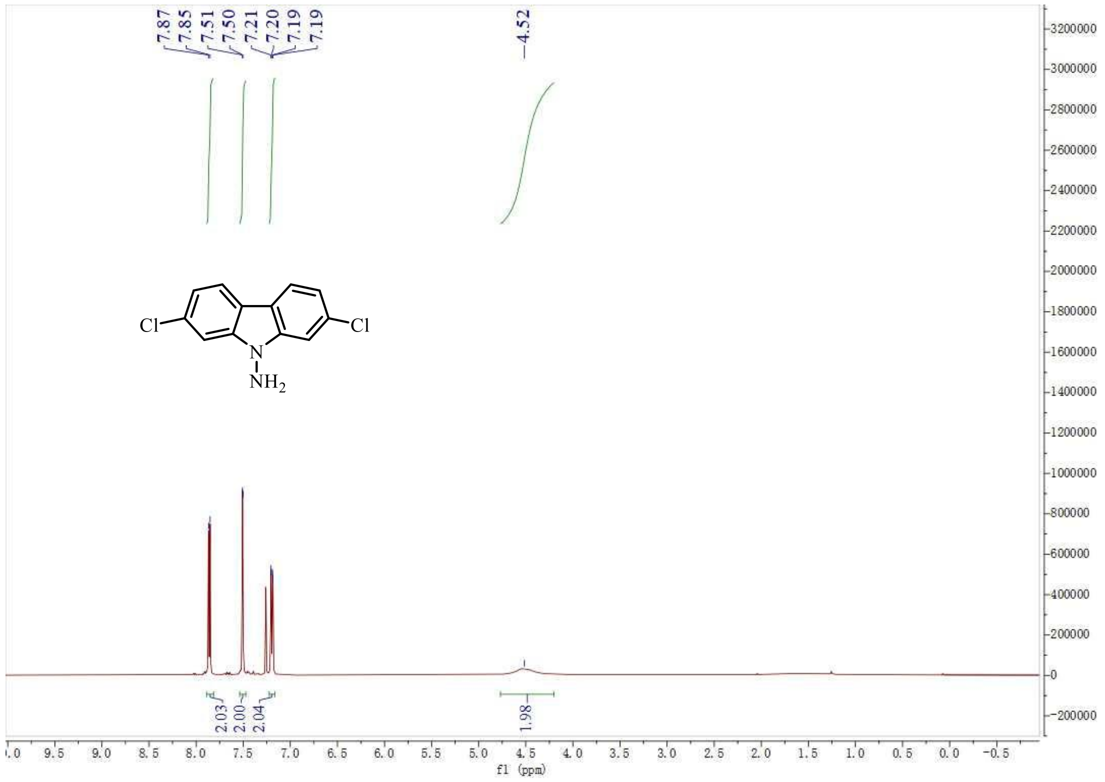

^{13}C NMR (126 MHz, Chloroform-*d*)

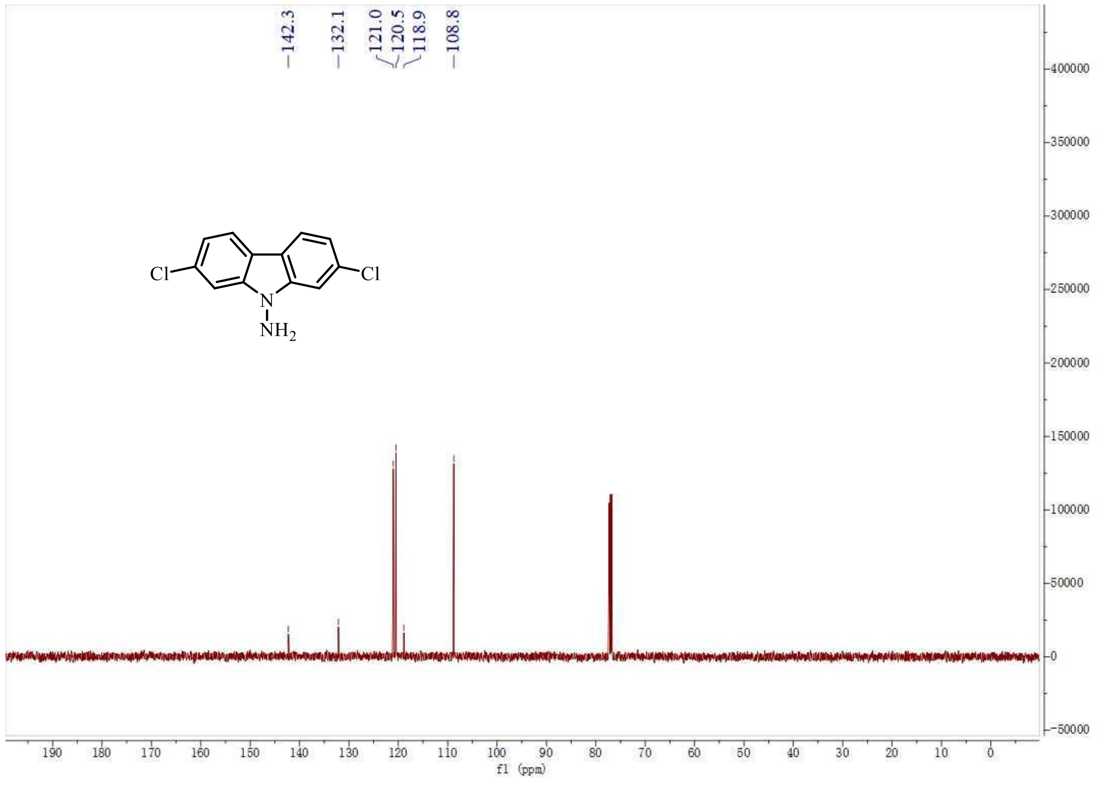

1-(2,7-dichloro-9*H*-carbazol-9-yl)-5-ethyl-1*H*-pyrrole-2-carboxylic acid (34)

^{1}H NMR (500 MHz, Chloroform-*d*)

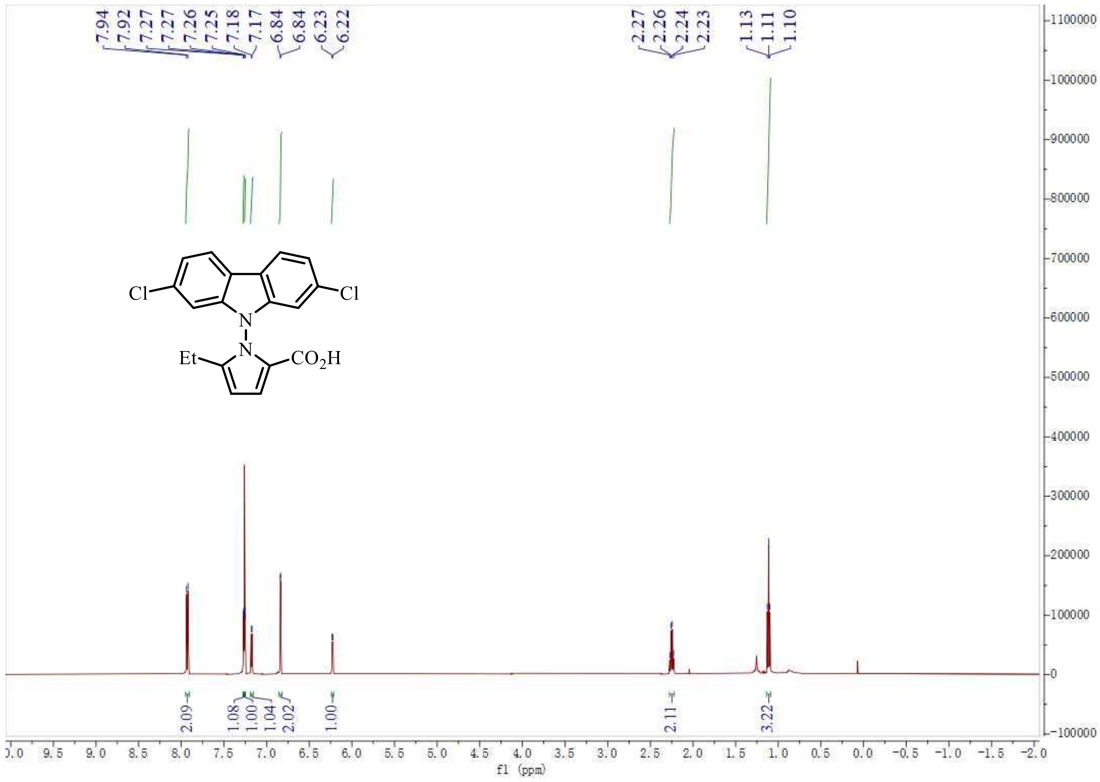

^{13}C NMR (126 MHz, Chloroform-*d*)

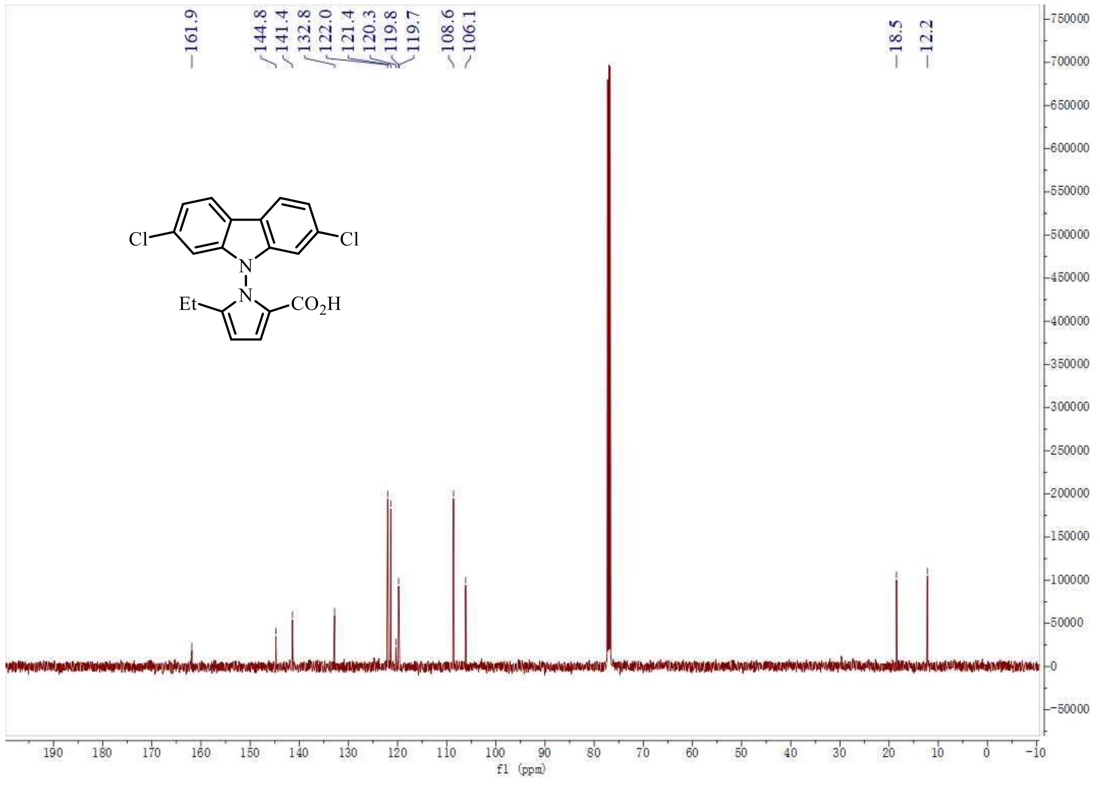

Ethyl 1-(2,7-dichloro-9*H*-carbazol-9-yl)-2-methyl-5-phenyl-1*H*-pyrrole-3-carboxylate (SI-4)

^{1}H NMR (500 MHz, Chloroform-*d*)

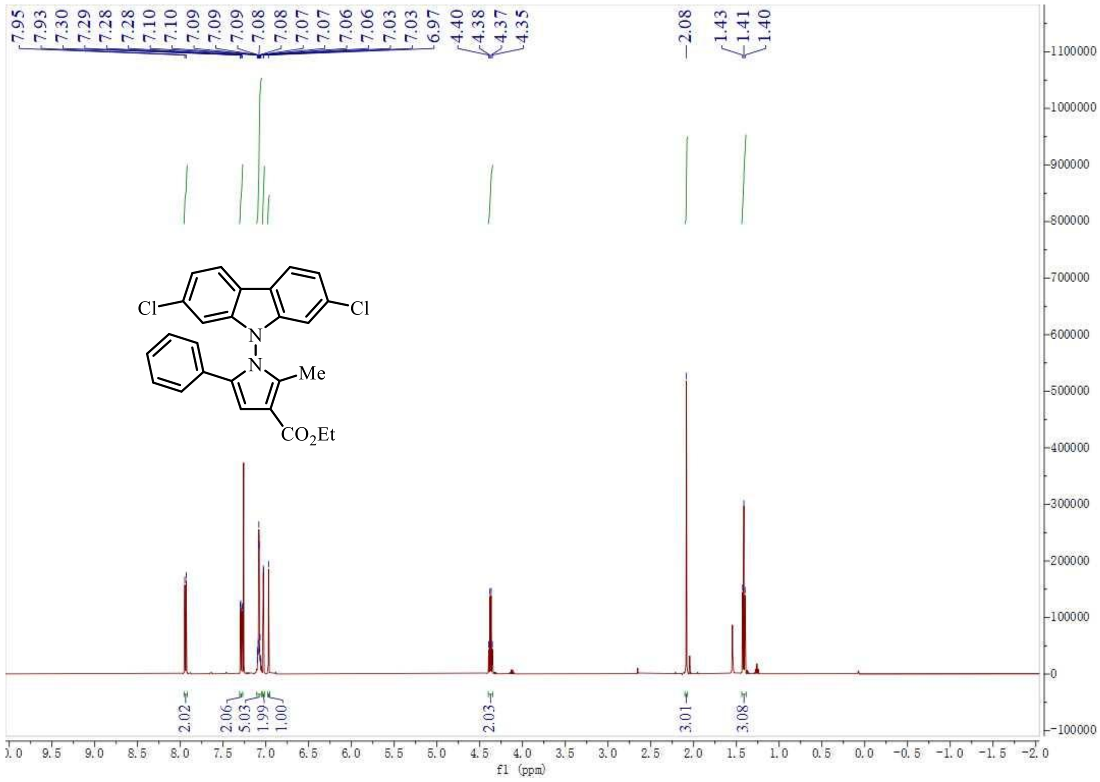

^{13}C NMR (126 MHz, Chloroform-*d*)

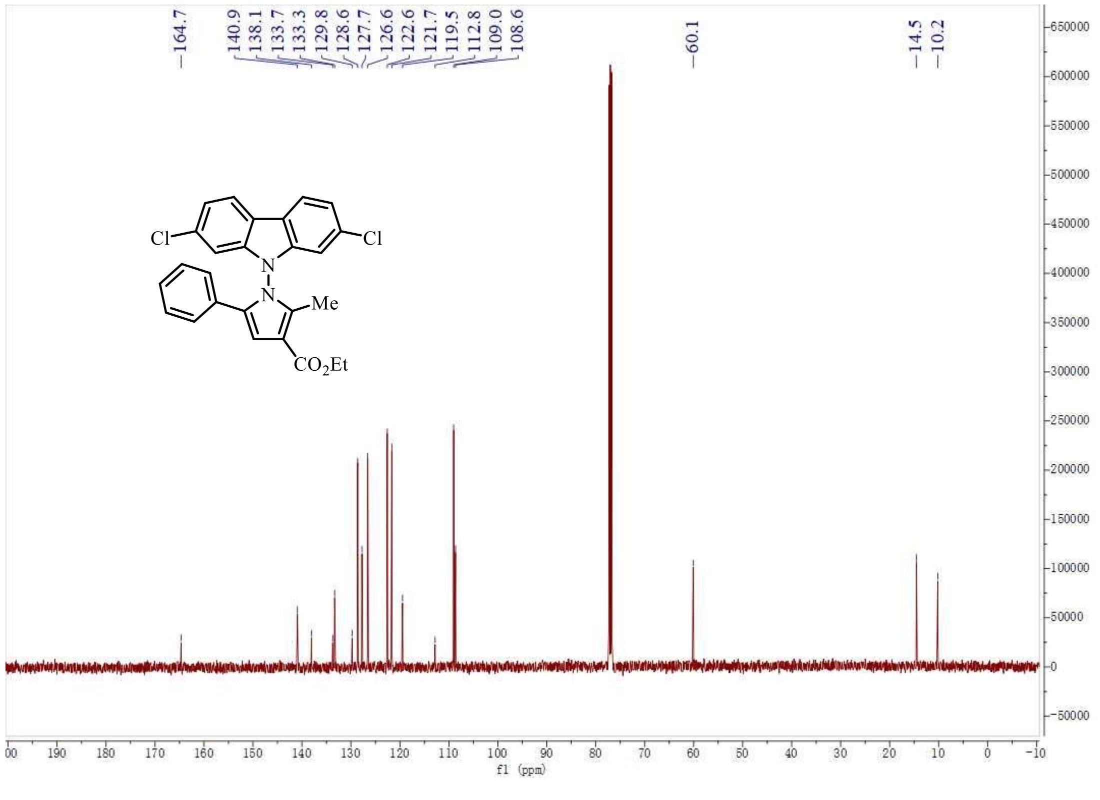

Ethyl 1-(2,7-dichloro-9*H*-carbazol-9-yl)-2-ethyl-5-phenyl-1*H*-pyrrole-3-carboxylate

^{1}H NMR (500 MHz, Chloroform-*d*)

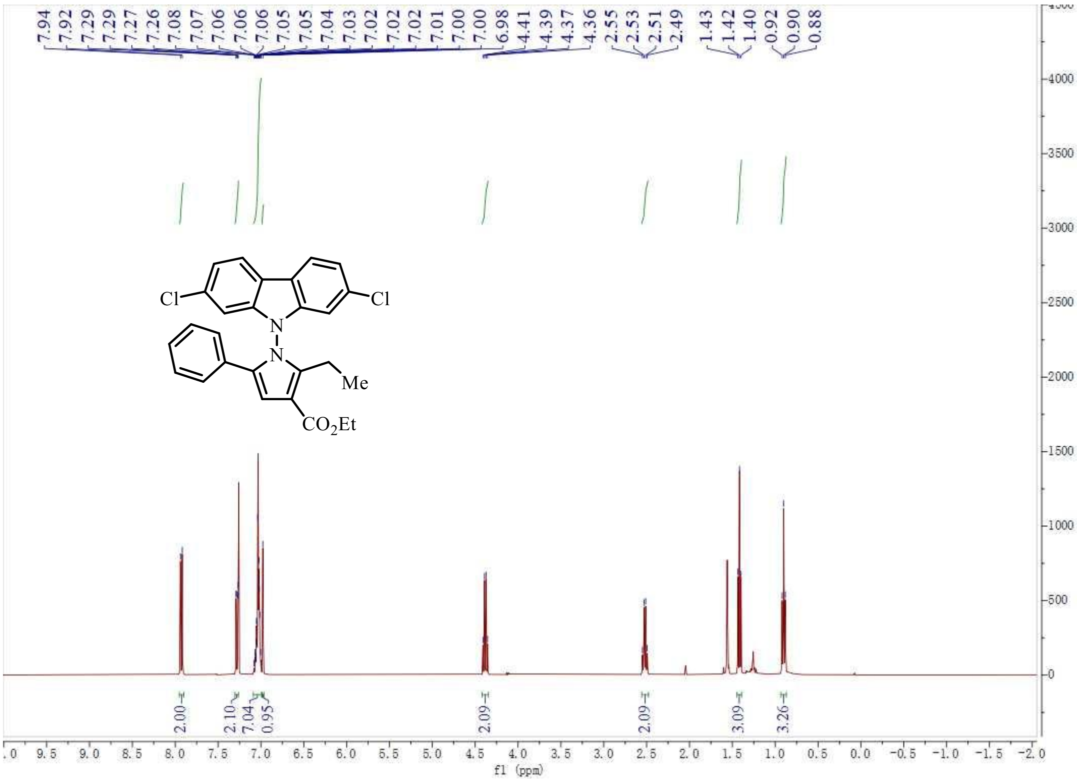

^{13}C NMR (126 MHz, Chloroform-*d*)

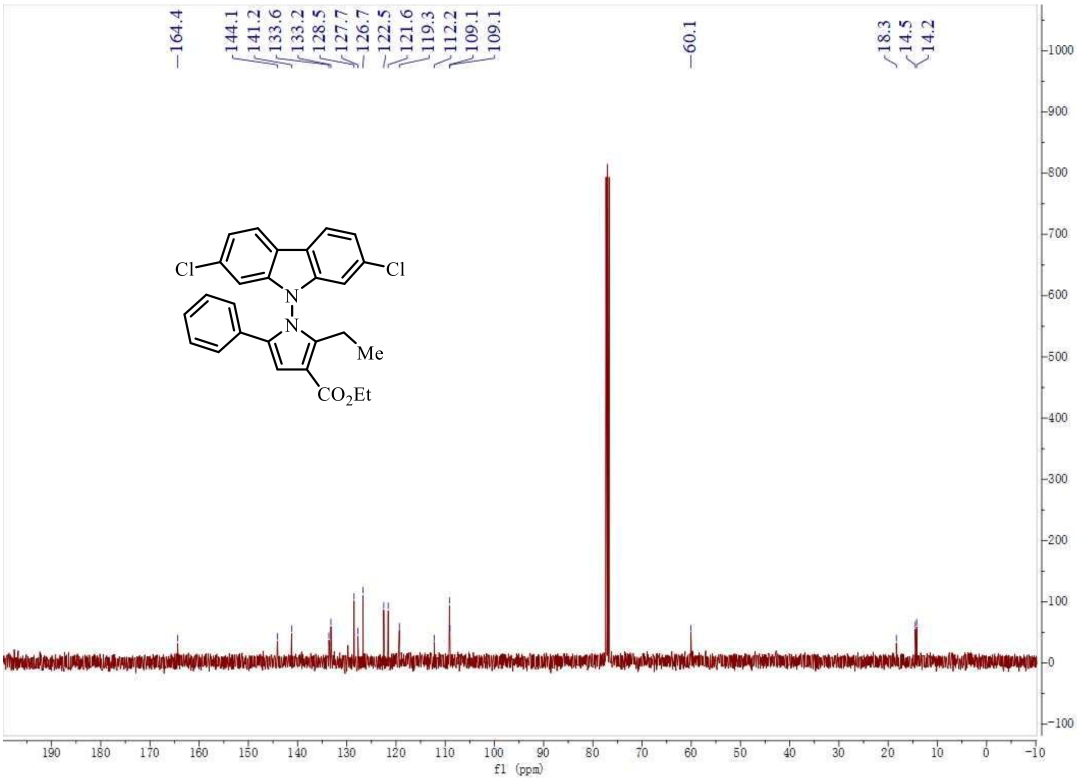

Ethyl 2-butyl-1-(2,7-dichloro-9*H*-carbazol-9-yl)-5-phenyl-1*H*-pyrrole-3-carboxylate

^{1}H NMR (500 MHz, Chloroform-*d*)

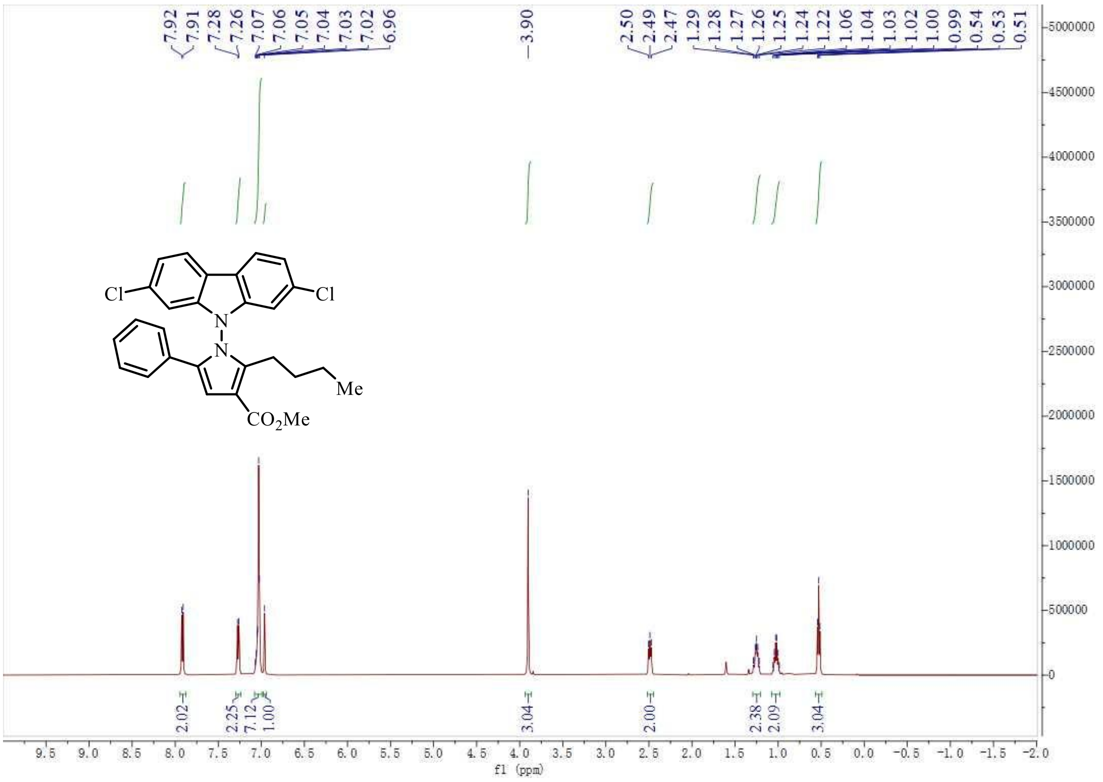

^{13}C NMR (126 MHz, Chloroform-*d*)

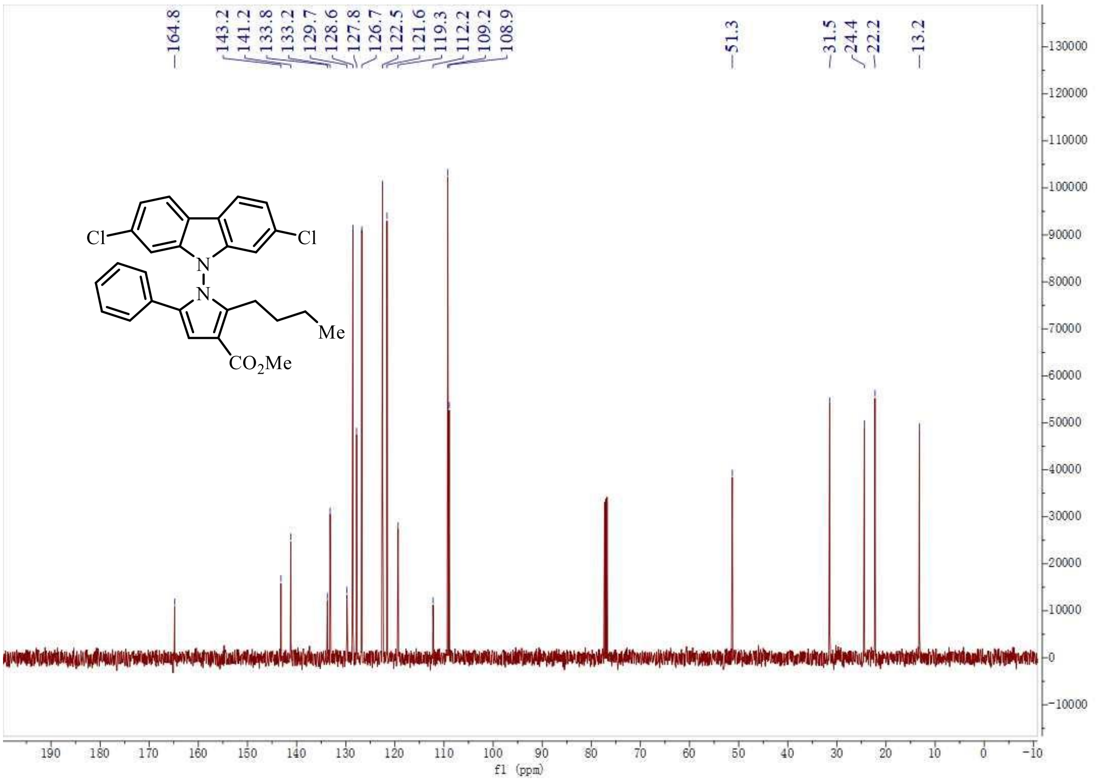

Ethyl 1-(2,7-dichloro-9*H*-carbazol-9-yl)-2-methyl-5-(3-nitrophenyl)-1*H*-pyrrole-3-carboxylate ^{1}H NMR (500 MHz, Chloroform-*d*)

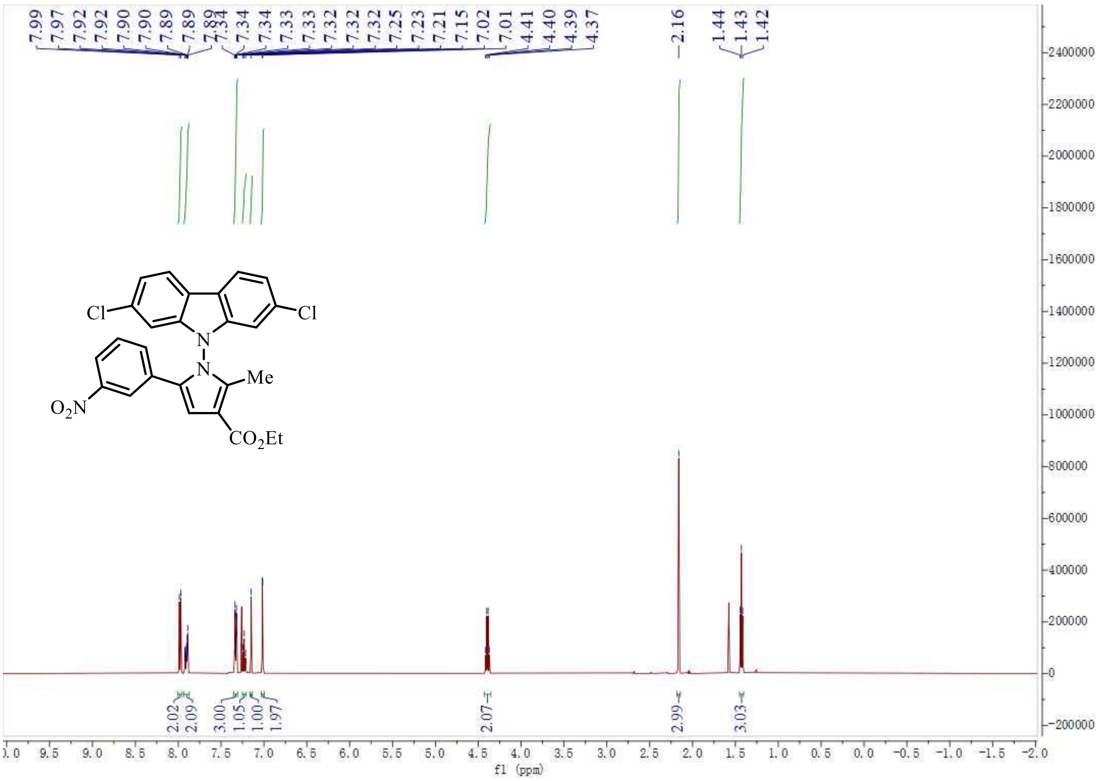

^{13}C NMR (126 MHz, Chloroform-*d*)

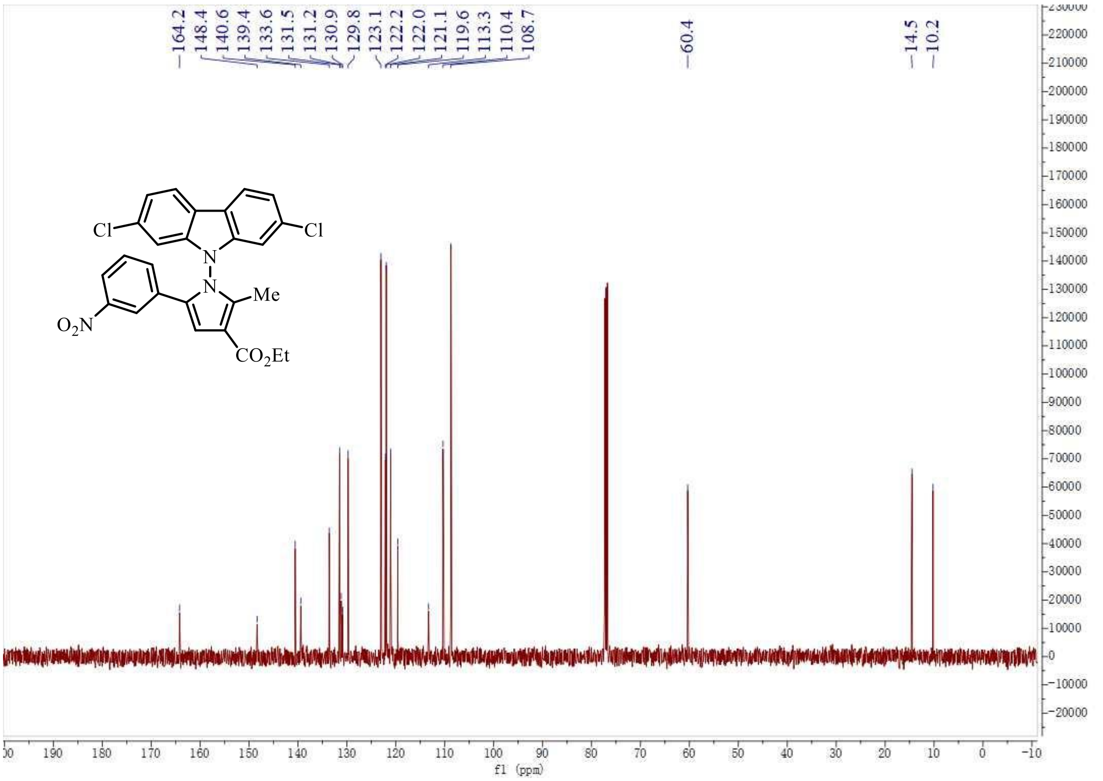

Ethyl 1-(2,7-dichloro-9*H*-carbazol-9-yl)-5-(3-methoxyphenyl)-2-methyl-1*H*-pyrrole-3-carboxylate ^{1}H NMR (500 MHz, Chloroform-*d*)

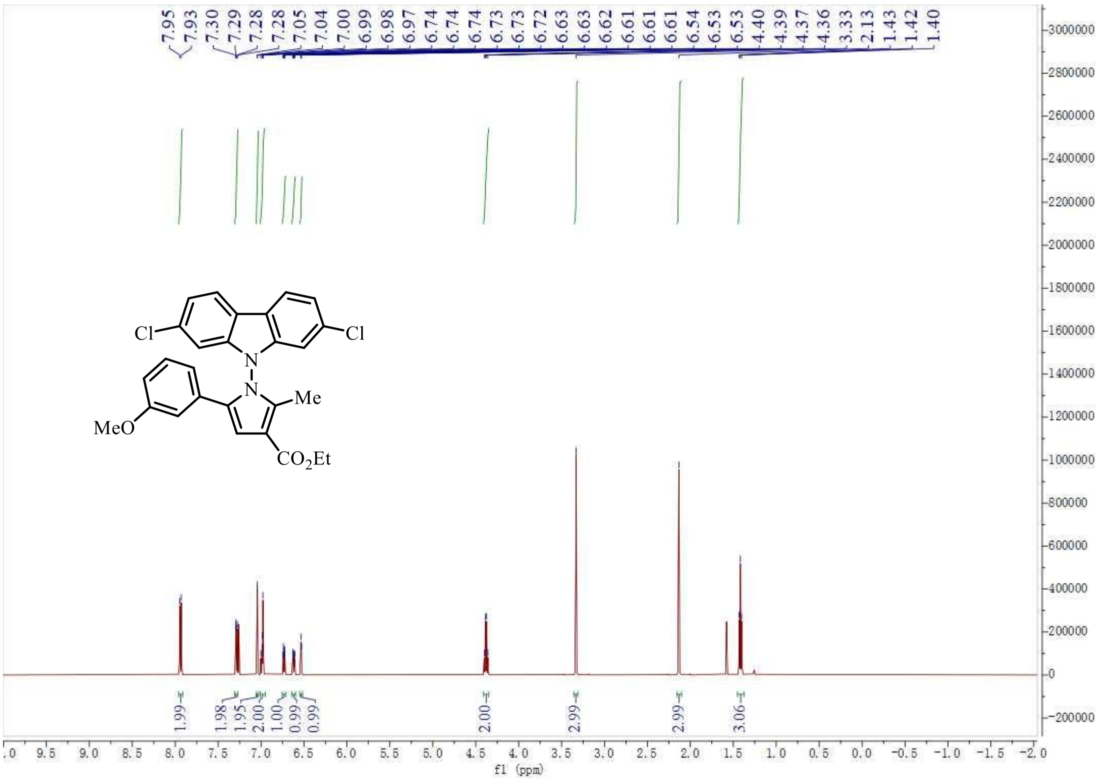

^{13}C NMR (126 MHz, Chloroform-*d*)

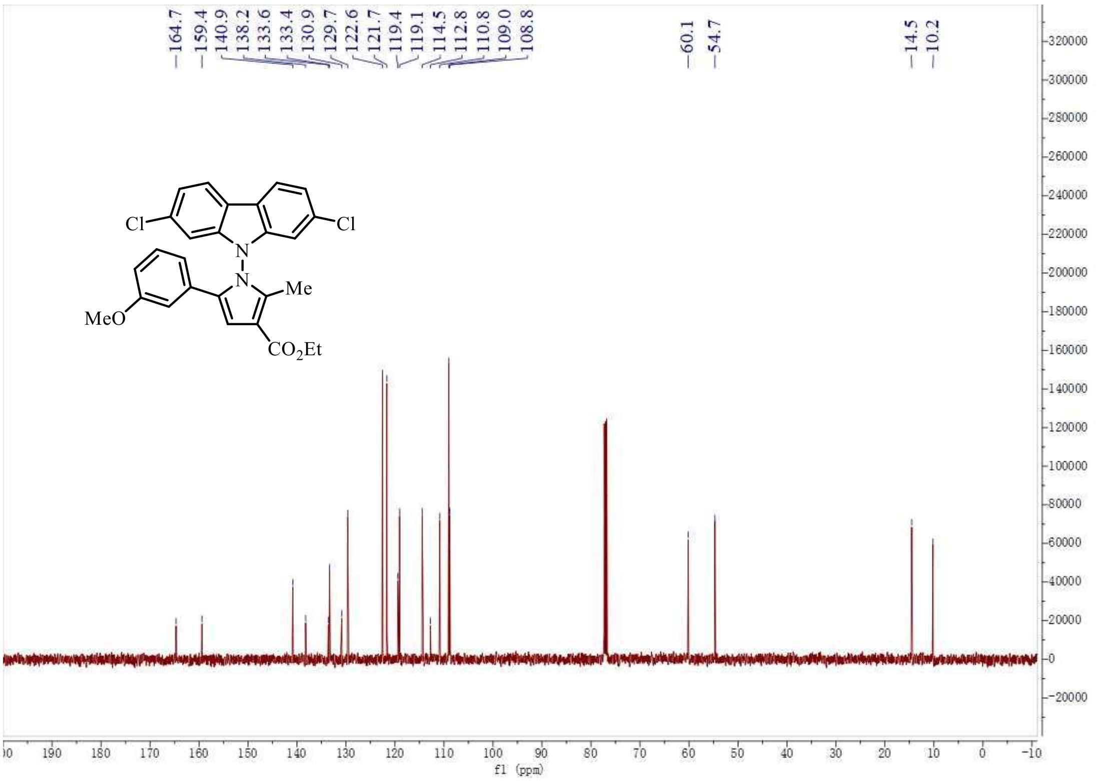

Ethyl 1-(2,7-dichloro-9*H*-carbazol-9-yl)-2-methyl-5-(naphthalen-2-yl)-1*H*-pyrrole-3-carboxylate ^{1}H NMR (500 MHz, Chloroform-*d*)

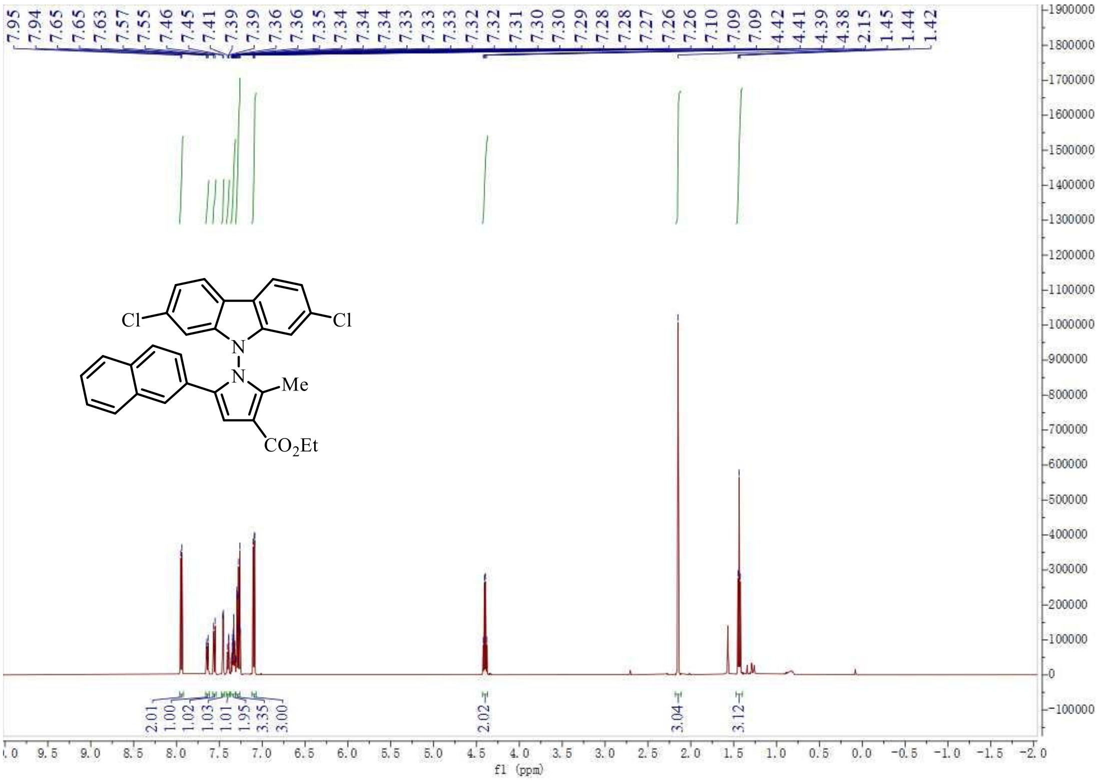

^{13}C NMR (126 MHz, Chloroform-*d*)

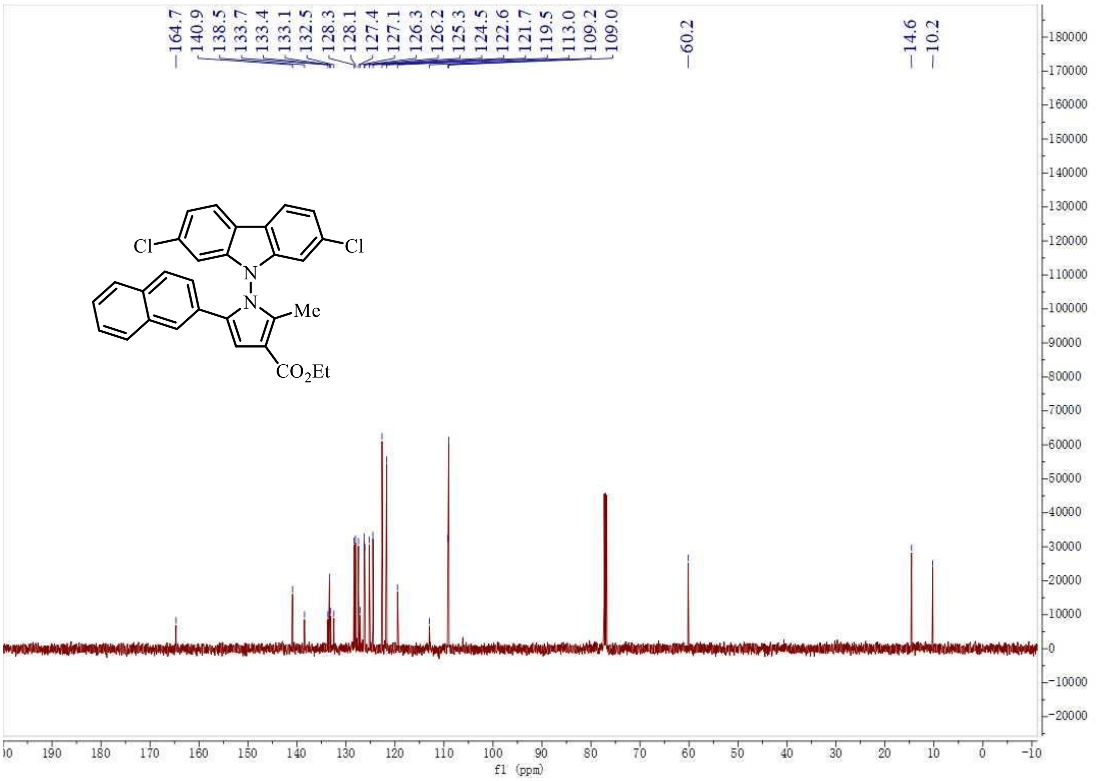

Ethyl 5-(benzofuran-2-yl)-1-(2,7-dichloro-9*H*-carbazol-9-yl)-2-methyl-1*H*-pyrrole-3-carboxylate ^{1}H NMR (500 MHz, Chloroform-*d*)

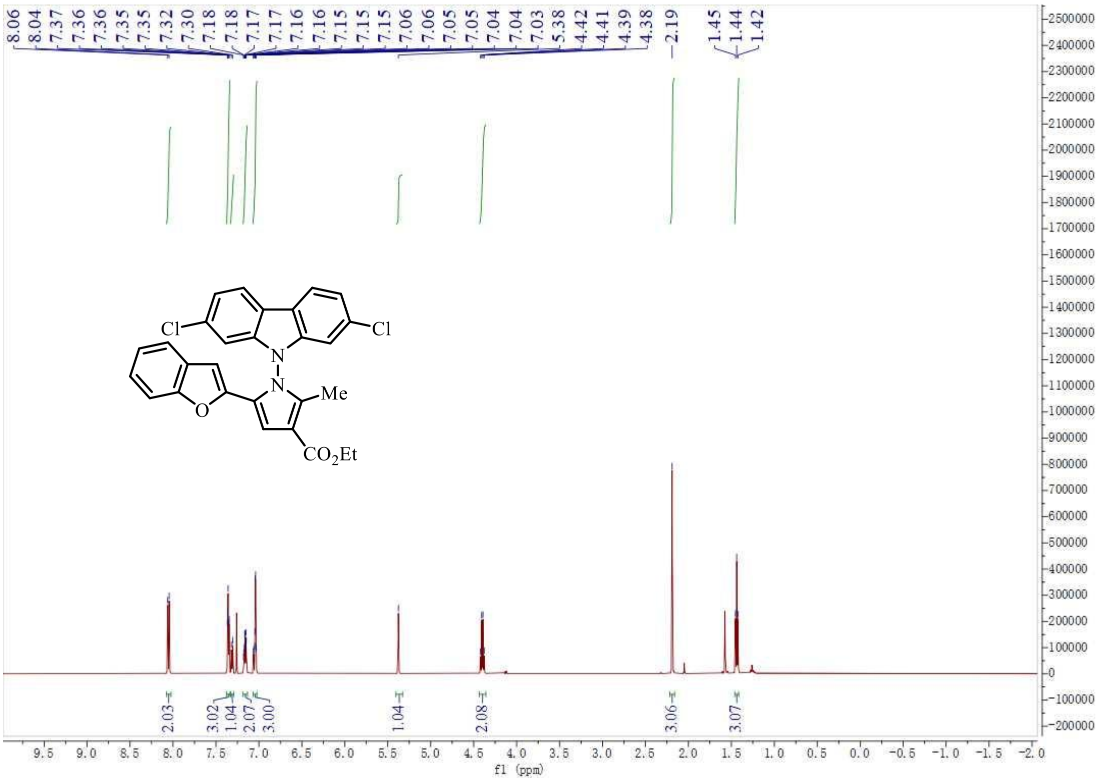

^{13}C NMR (126 MHz, Chloroform-*d*)

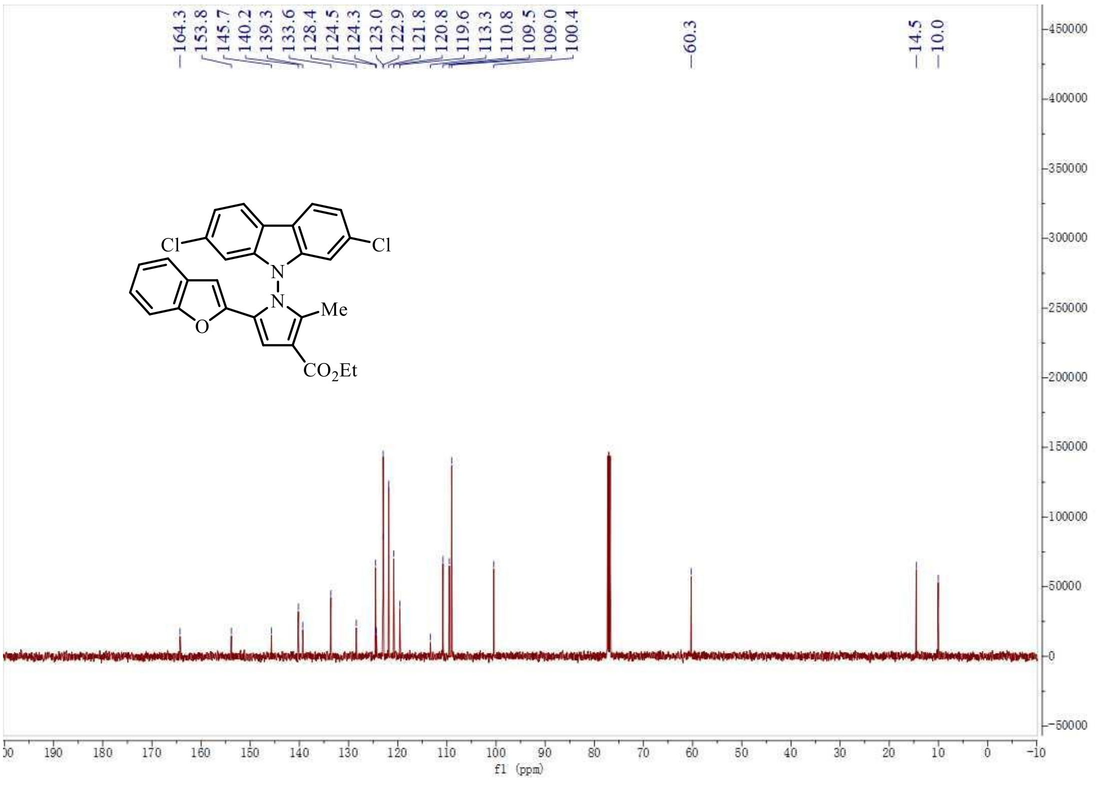

Ethyl 1-(2,7-dichloro-9*H*-carbazol-9-yl)-2-methyl-5-(thiophen-2-yl)-1*H*-pyrrole-3-carboxylate ^{1}H NMR (500 MHz, Chloroform-*d*)

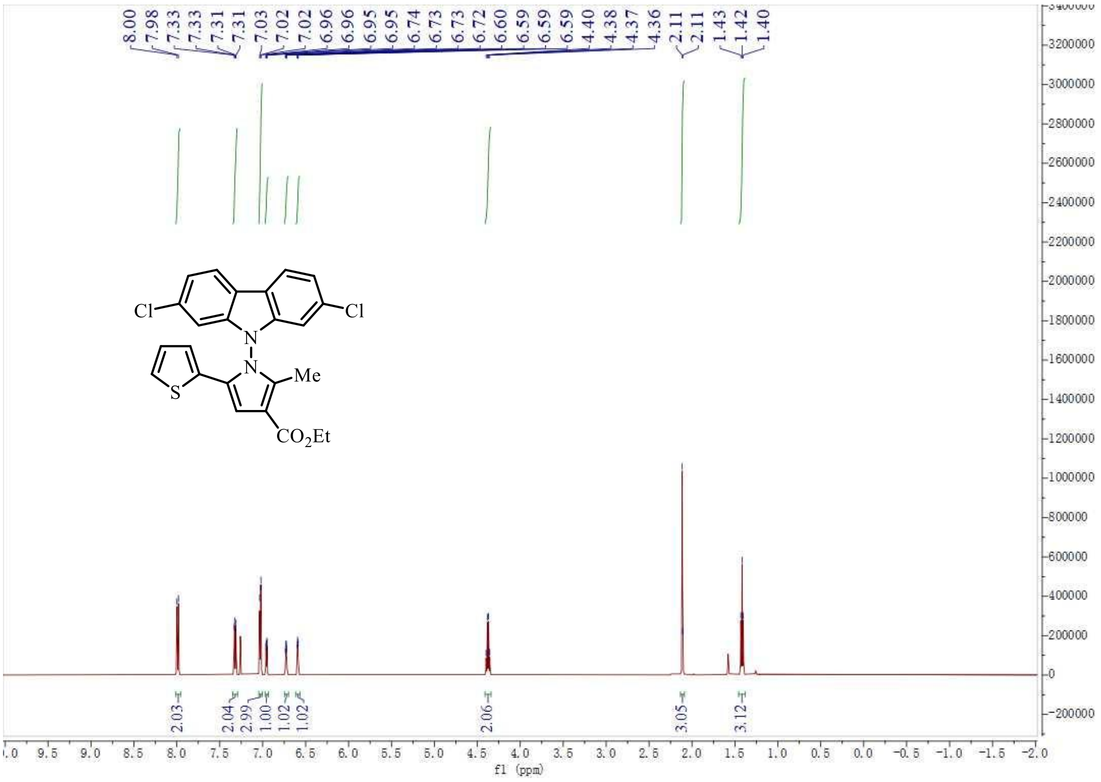

^{13}C NMR (126 MHz, Chloroform-*d*)

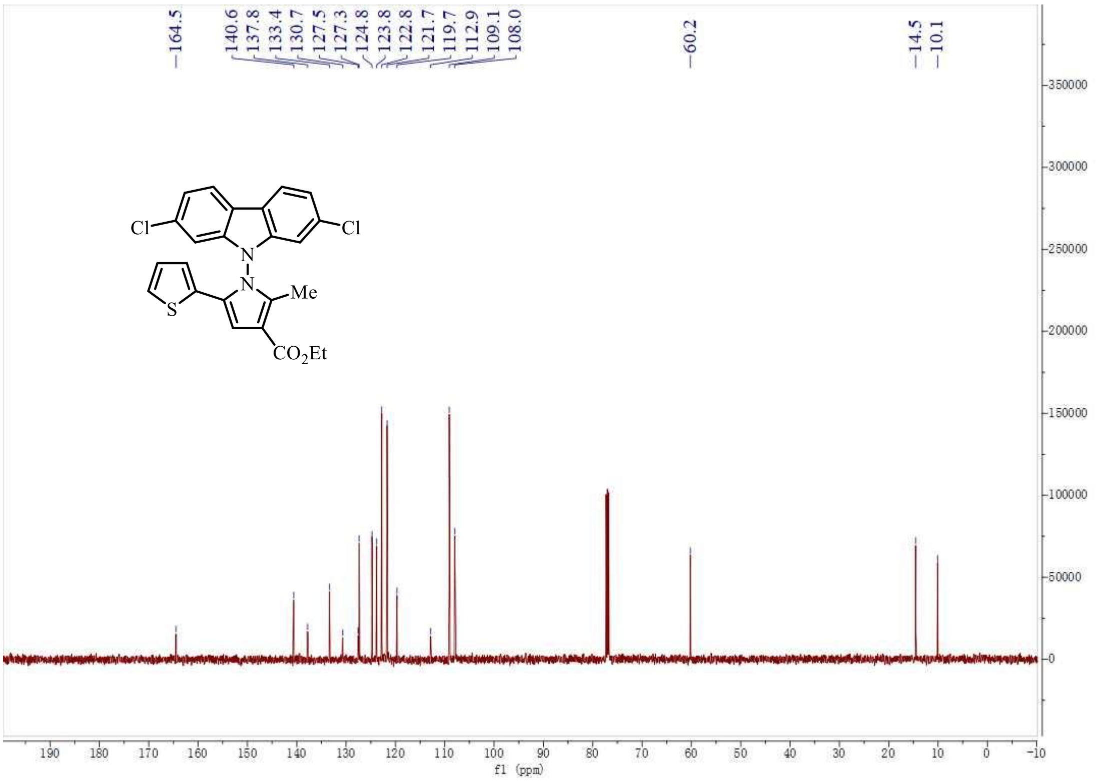

Ethyl 5-cyclopropyl-1-(2,7-dichloro-9H-carbazol-9-yl)-2-methyl-1H-pyrrole-3-carboxylate

^{1}H NMR (500 MHz, Chloroform-d)

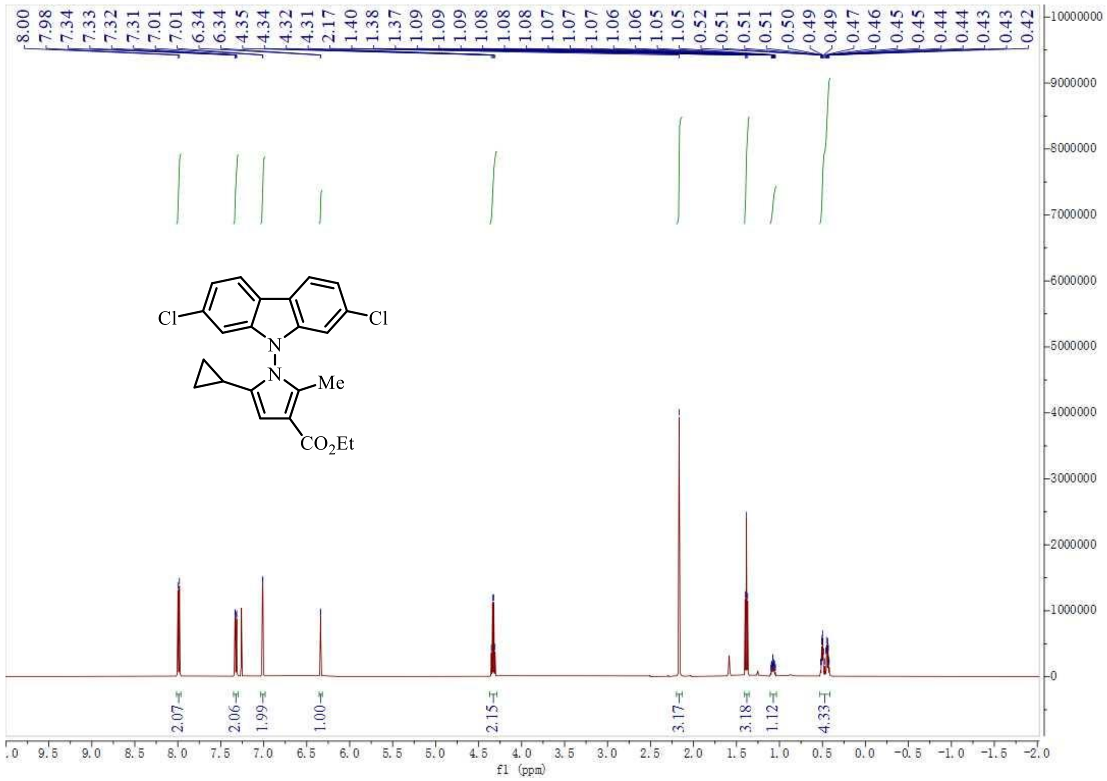

^{13}C NMR (126 MHz, Chloroform-d)

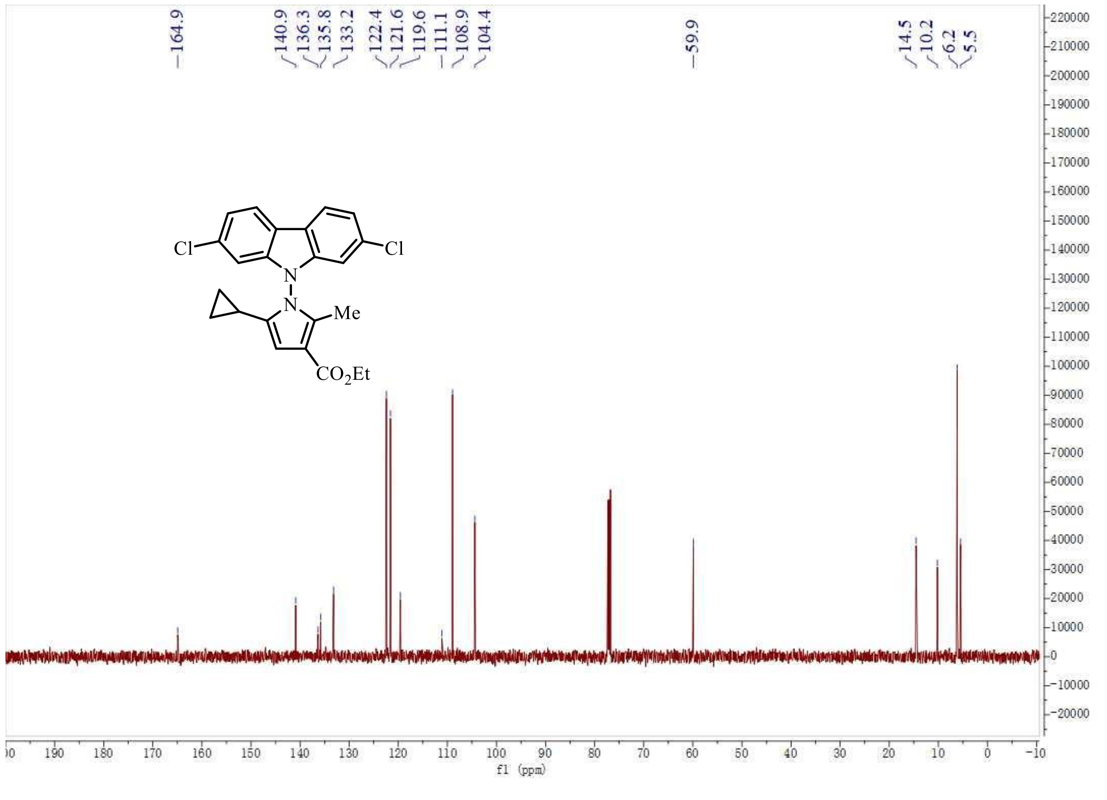

1-(3,6-Dichloro-9*H*-carbazol-9-yl)-2-methyl-5-phenyl-1*H*-pyrrole-3-carboxylic acid (60)

^{1}H NMR (500 MHz, Chloroform-*d*)

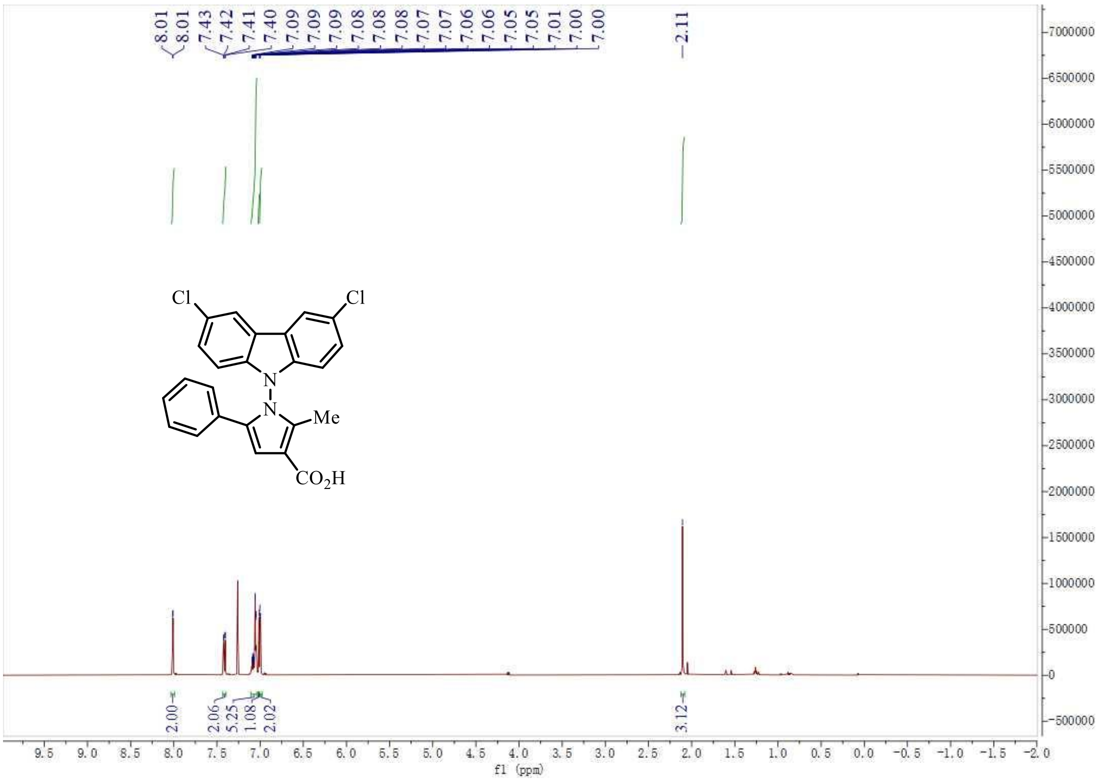

^{13}C NMR (126 MHz, Chloroform-*d*)

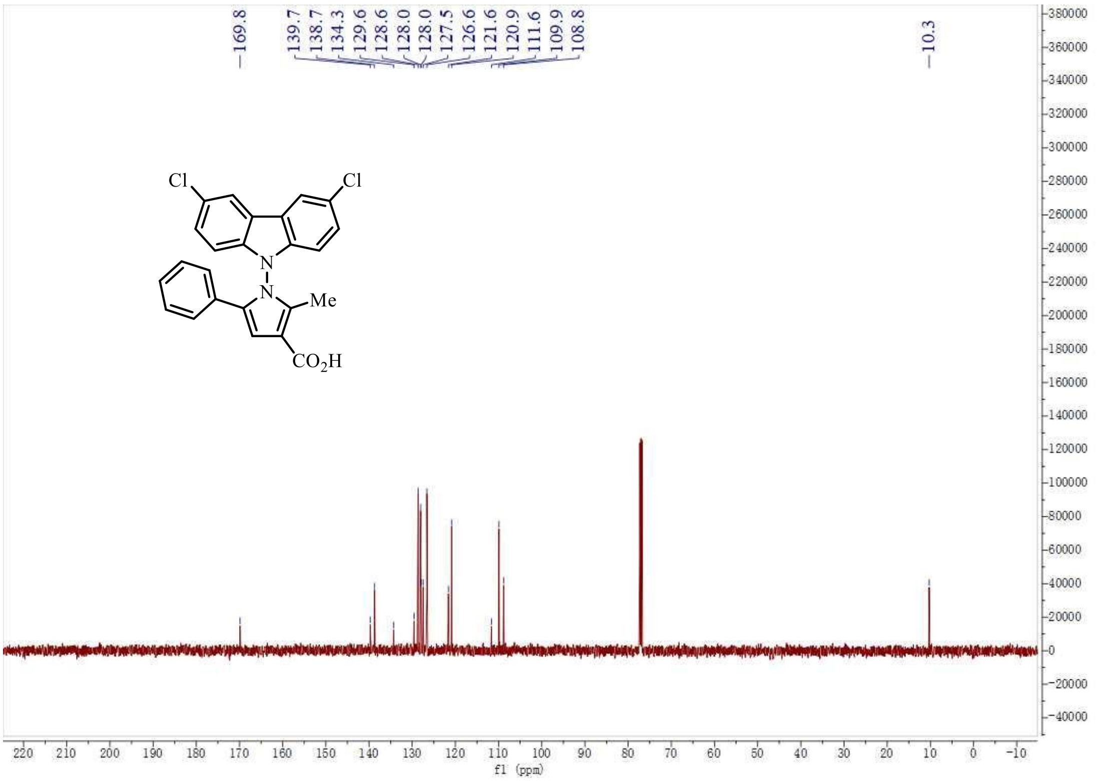

Ethyl (*R*)-2-(2-chloro-7-(4-methoxyphenyl)-9*H*-carbazol-9-yl)-3-methylbenzoate (2)

^{1}H NMR (500 MHz, Chloroform-*d*)

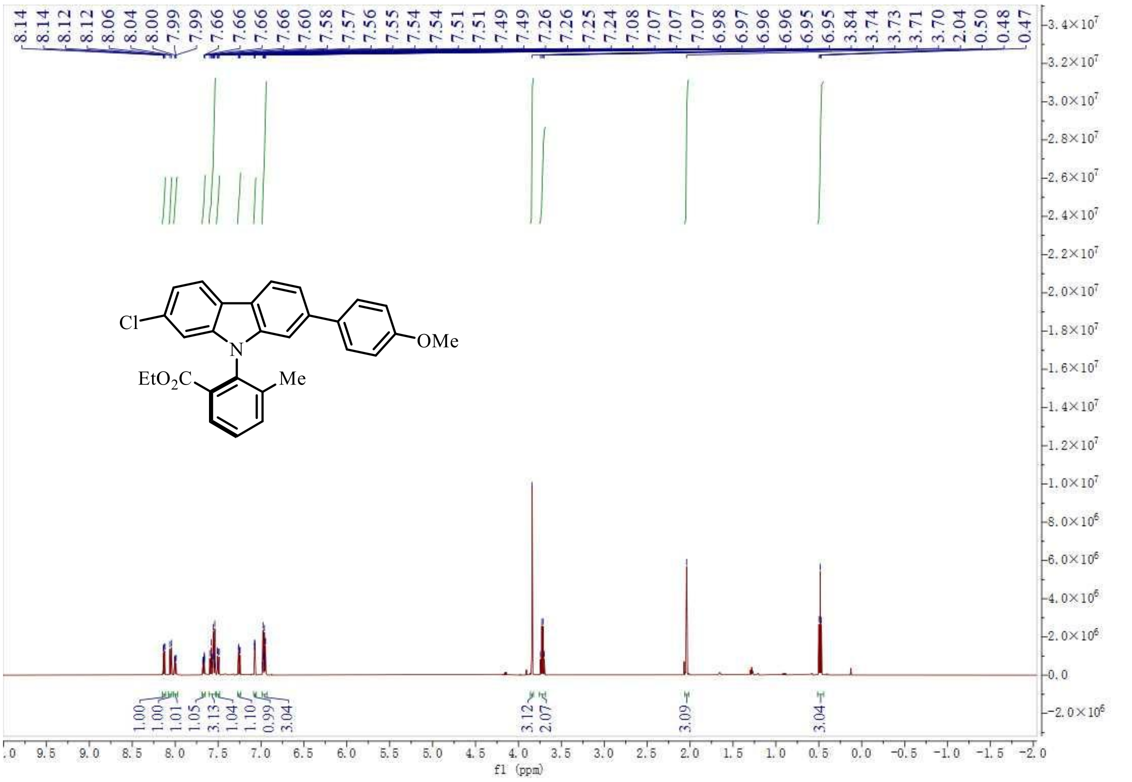

^{13}C NMR (126 MHz, Chloroform-*d*)

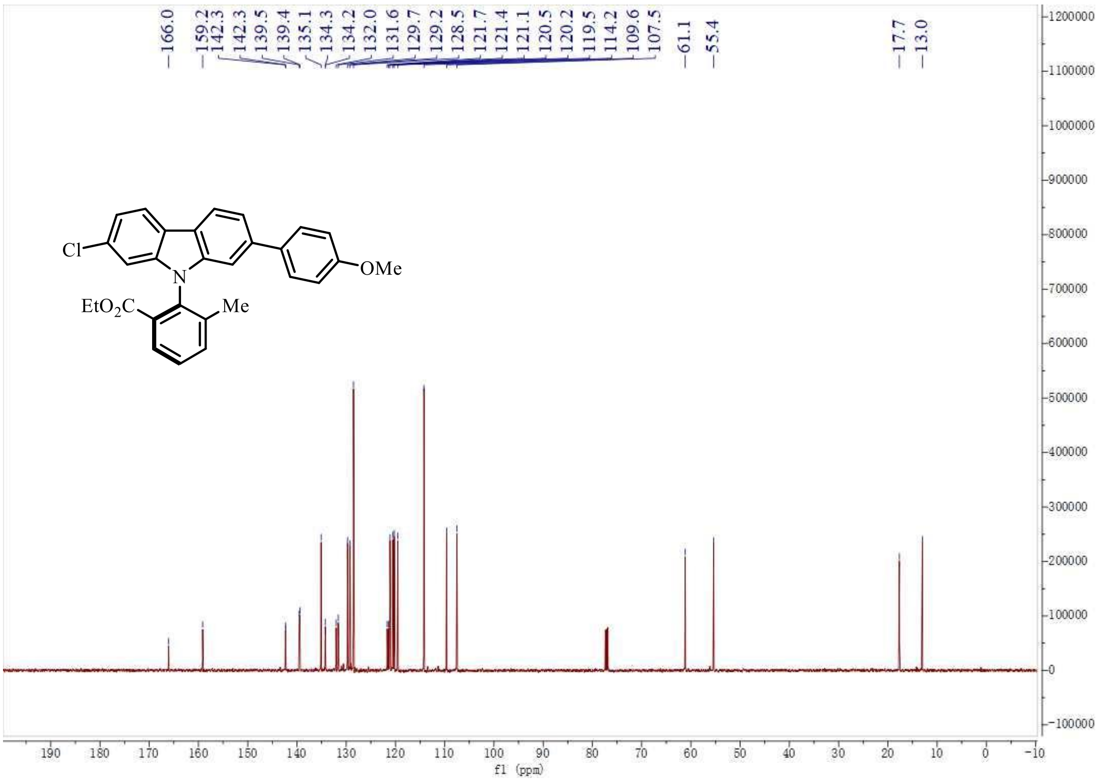

Ethyl (*R*)-2-(2-chloro-7-(4-(diphenylamino)phenyl)-9*H*-carbazol-9-yl)-3-methylbenzoate (3)

^{1}H NMR (500 MHz, Chloroform-*d*)

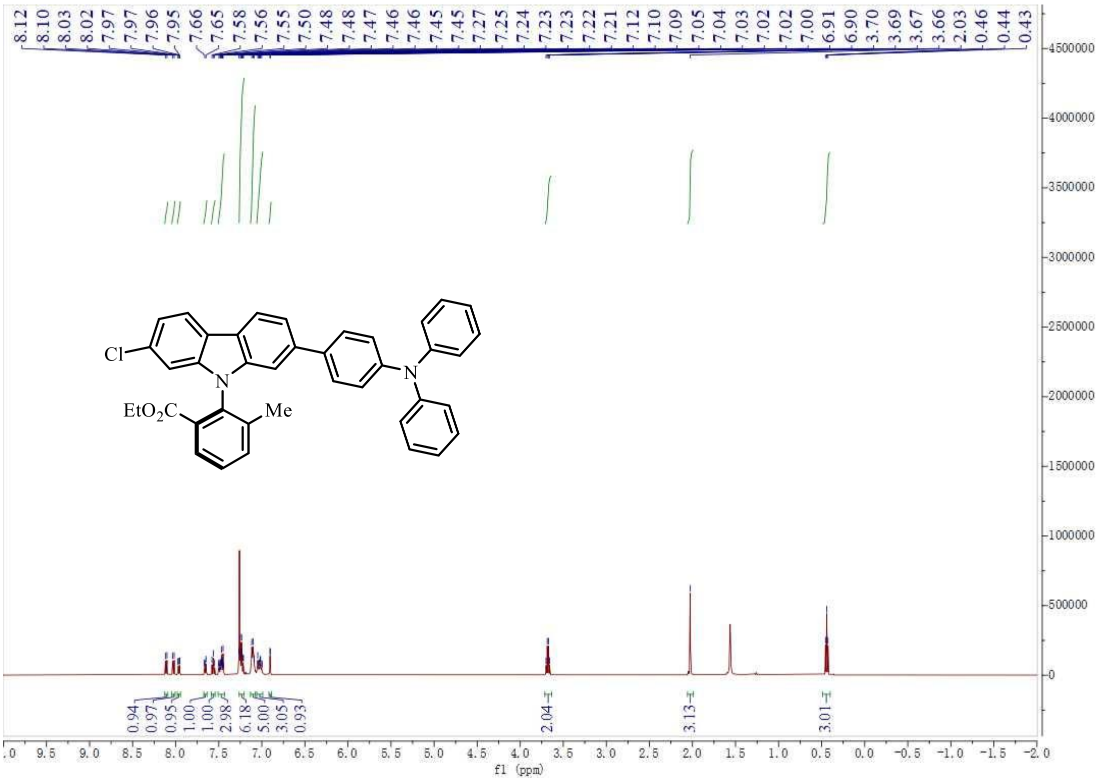

^{13}C NMR (126 MHz, Chloroform-*d*)

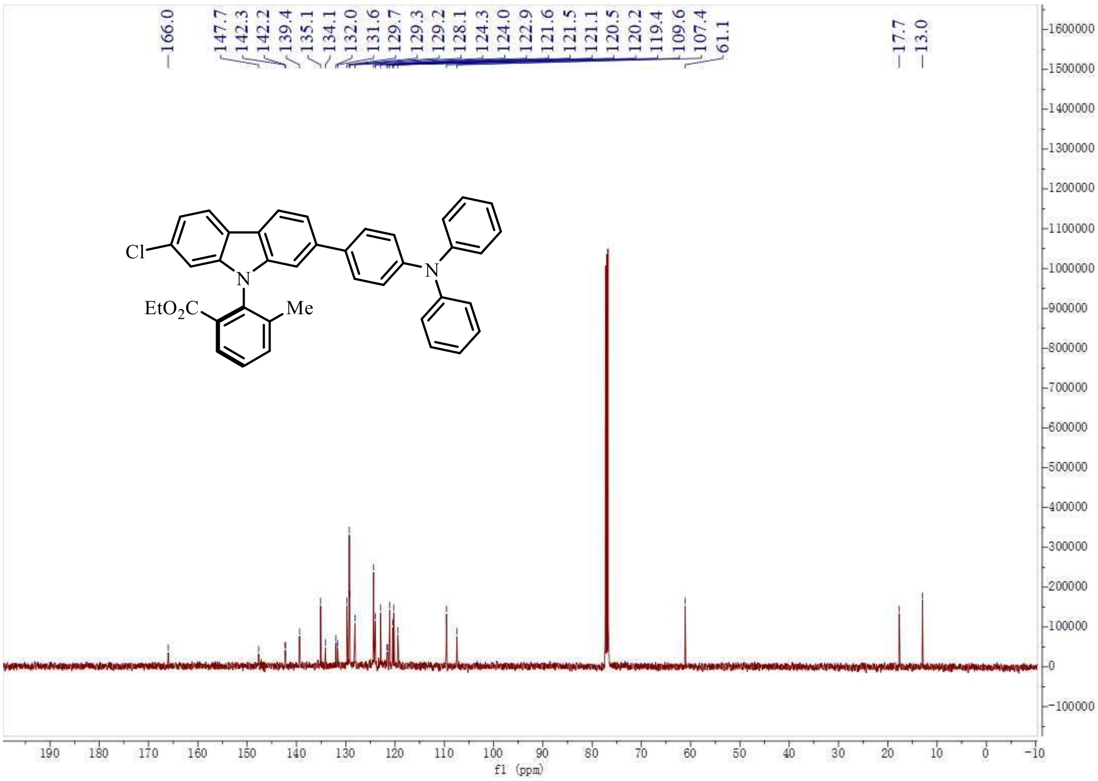

Ethyl (*S*)-2-(2-chloro-7-(4-(ethoxycarbonyl)phenyl)-9*H*-carbazol-9-yl)-3-methylbenzoate (4)

^{1}H NMR (500 MHz, Chloroform-*d*)

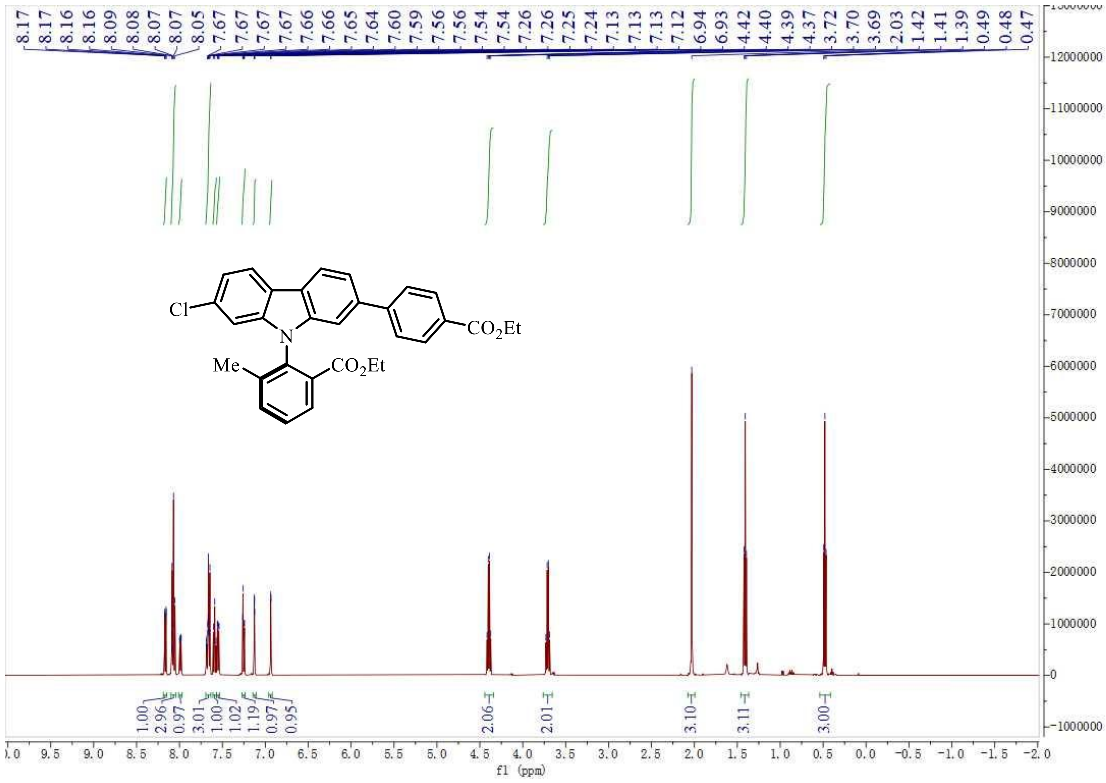

^{13}C NMR (126 MHz, Chloroform-*d*)

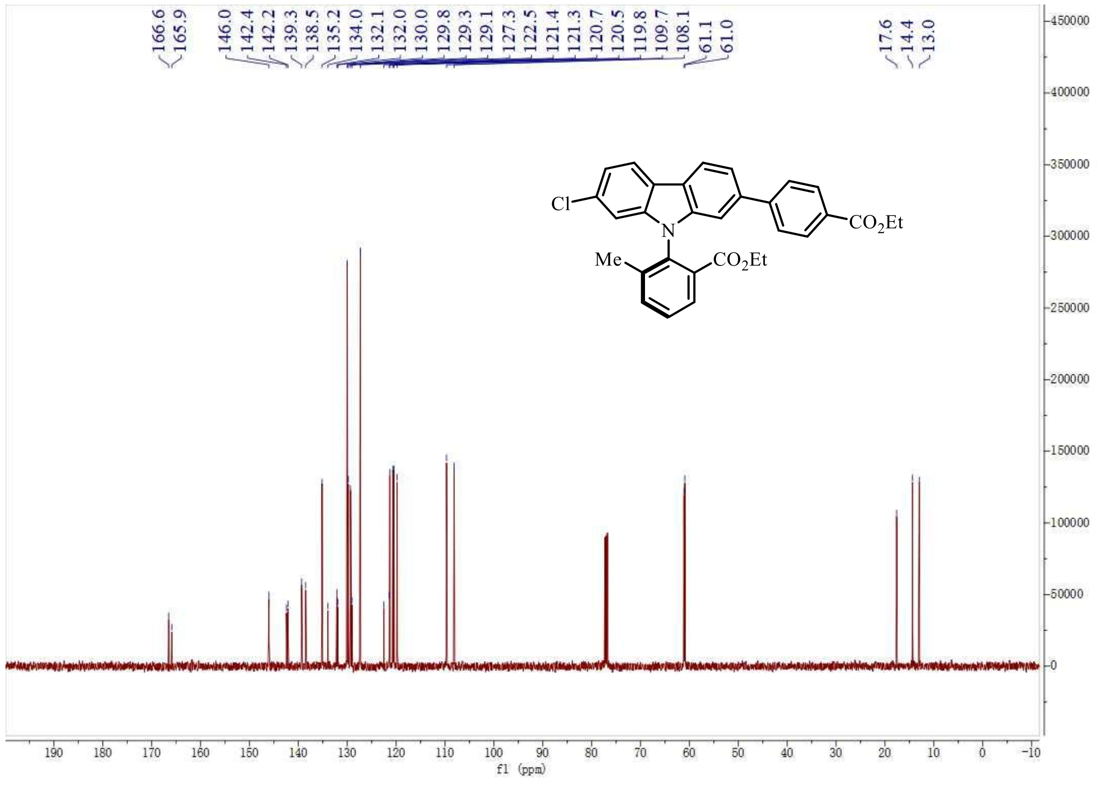

Ethyl (*S*)-2-(2-chloro-7-(4-cyanophenyl)-9*H*-carbazol-9-yl)-3-methylbenzoate (5)

^{1}H NMR (500 MHz, Chloroform-*d*)

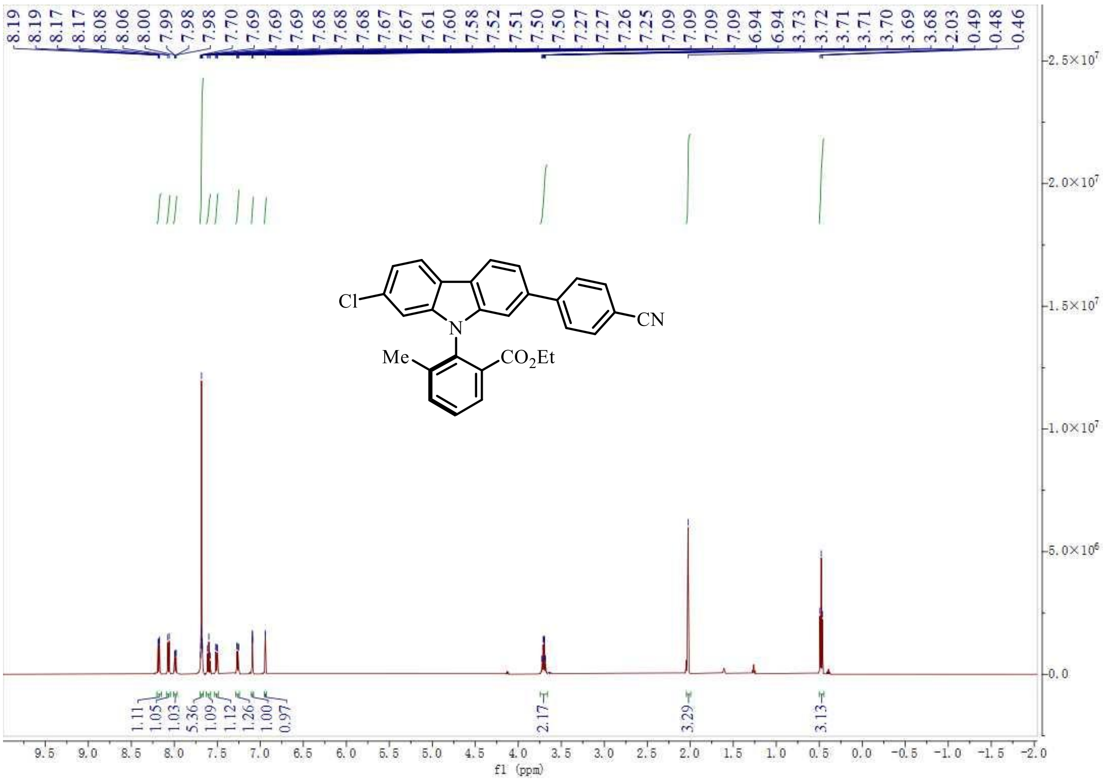

^{13}C NMR (126 MHz, Chloroform-*d*)

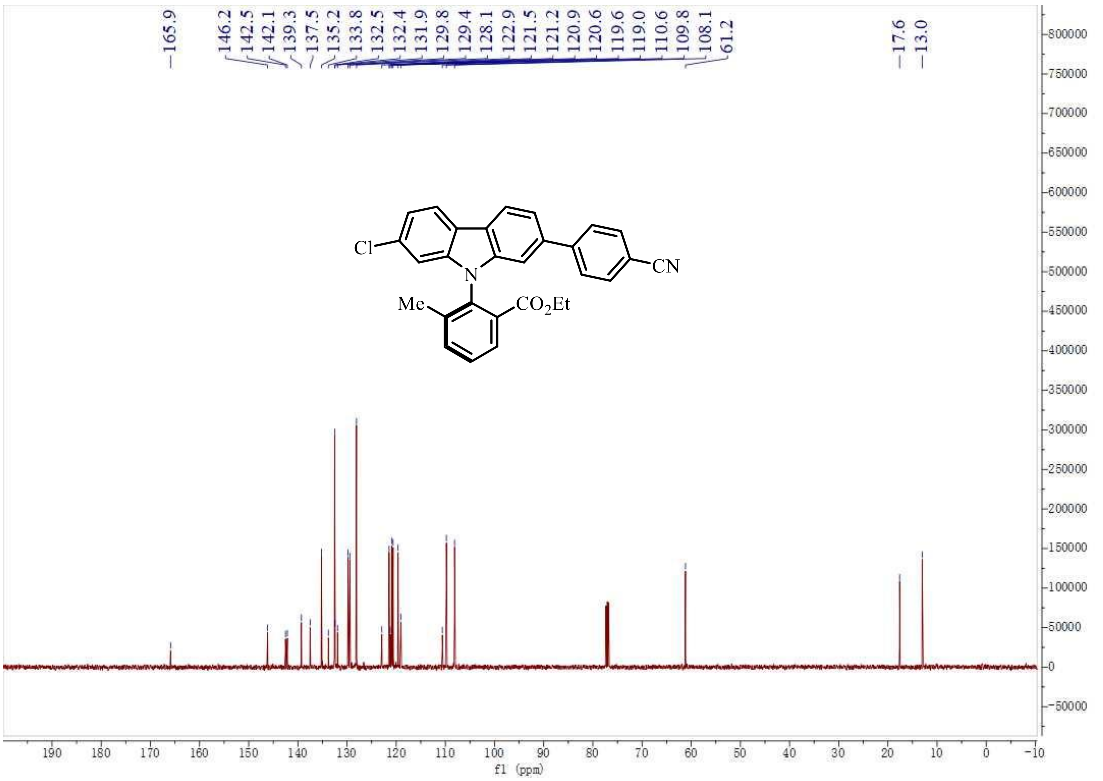

Ethyl (*R*)-2-(2-chloro-7-(3-nitrophenyl)-9*H*-carbazol-9-yl)-3-methylbenzoate (6)

^{1}H NMR (500 MHz, Chloroform-*d*)

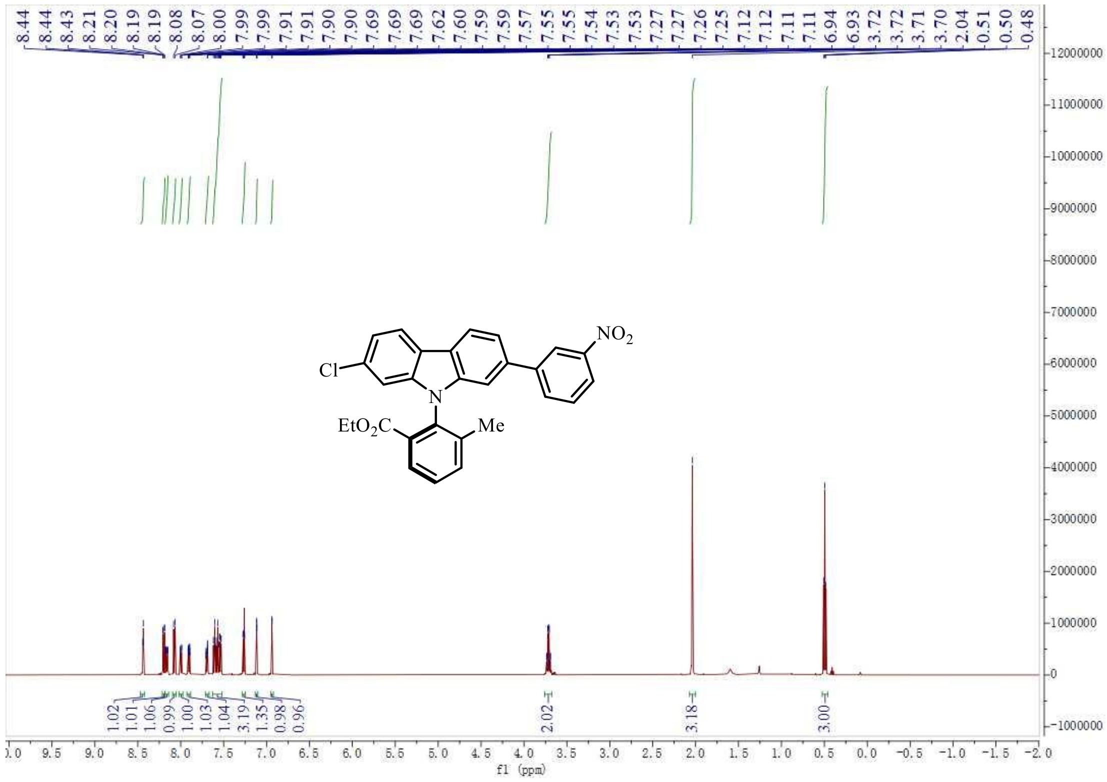

^{13}C NMR (126 MHz, Chloroform-*d*)

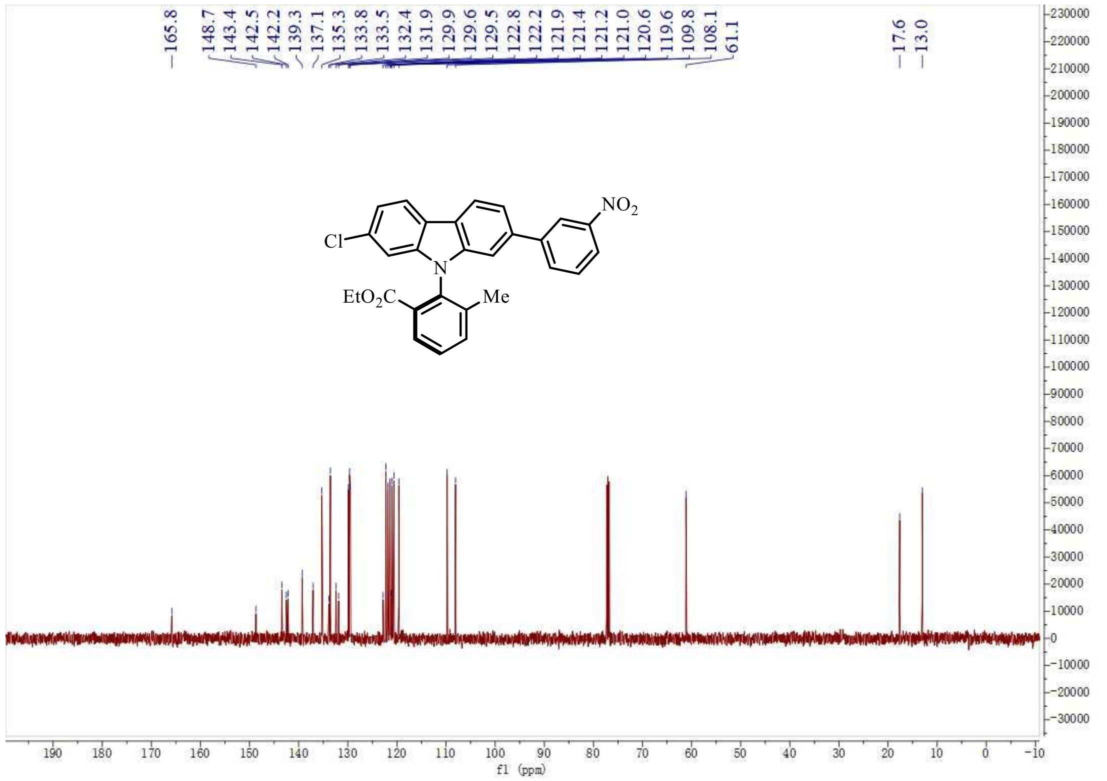

Ethyl (*R*)-2-(2-chloro-7-(3-(ethoxycarbonyl)phenyl)-9*H*-carbazol-9-yl)-3-methylbenzoate (7)

^{1}H NMR (500 MHz, Chloroform-*d*)

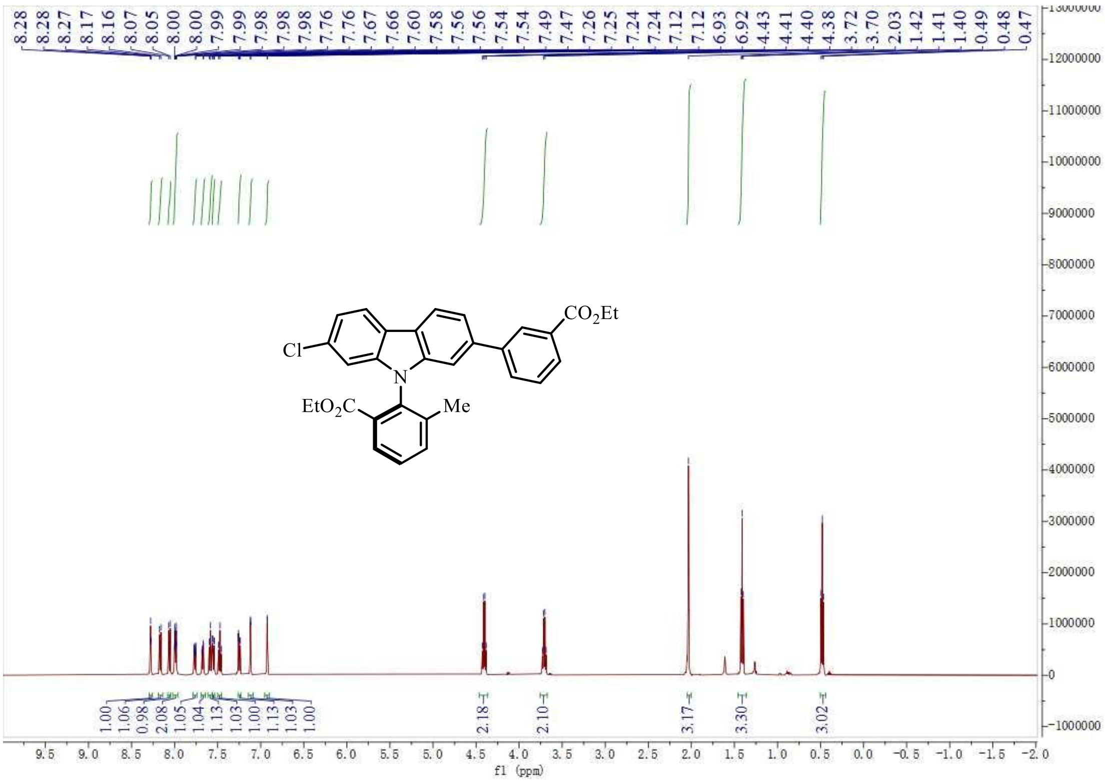

^{13}C NMR (126 MHz, Chloroform-*d*)

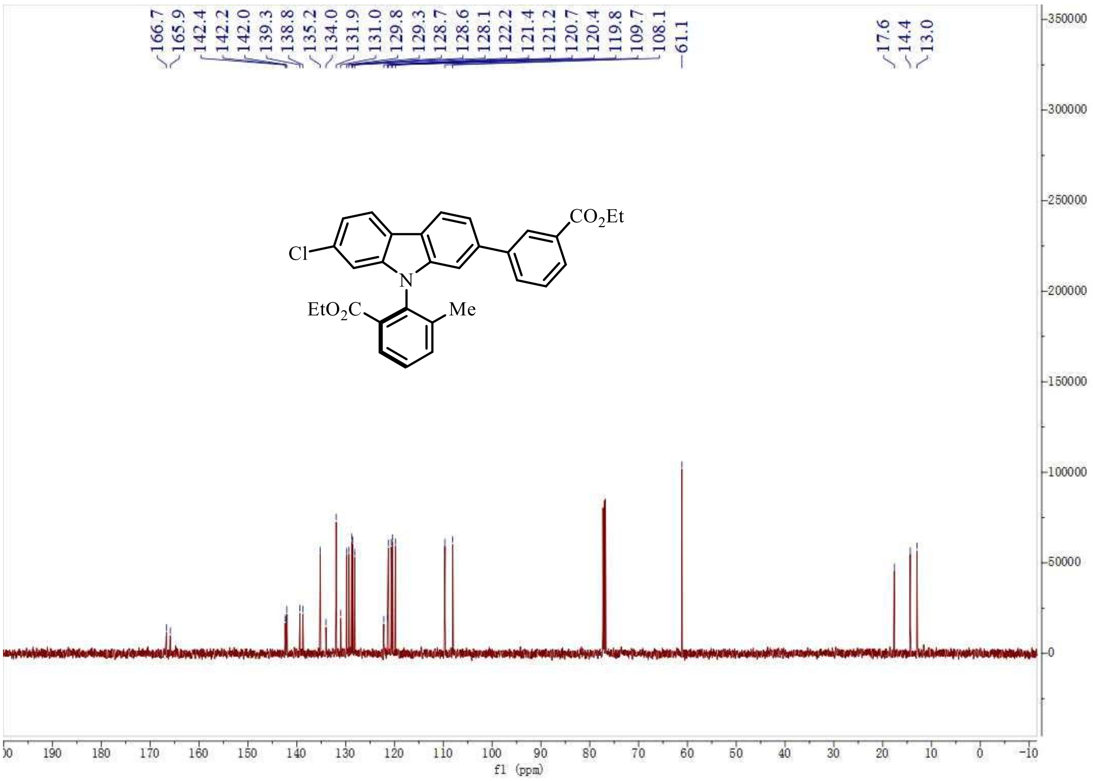

Ethyl (*S*)-2-(2-(3-(benzyloxy)phenyl)-7-chloro-9*H*-carbazol-9-yl)-3-methylbenzoate (8)

^{1}H NMR (500 MHz, Chloroform-*d*)

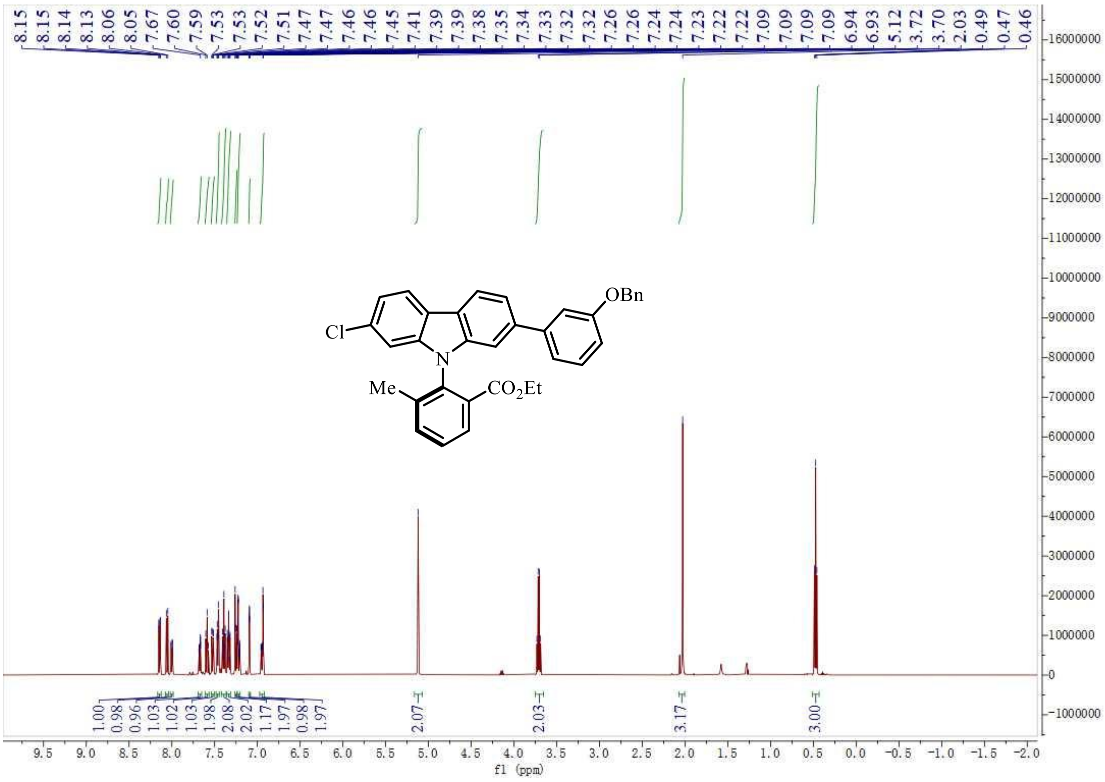

^{13}C NMR (126 MHz, Chloroform-*d*)

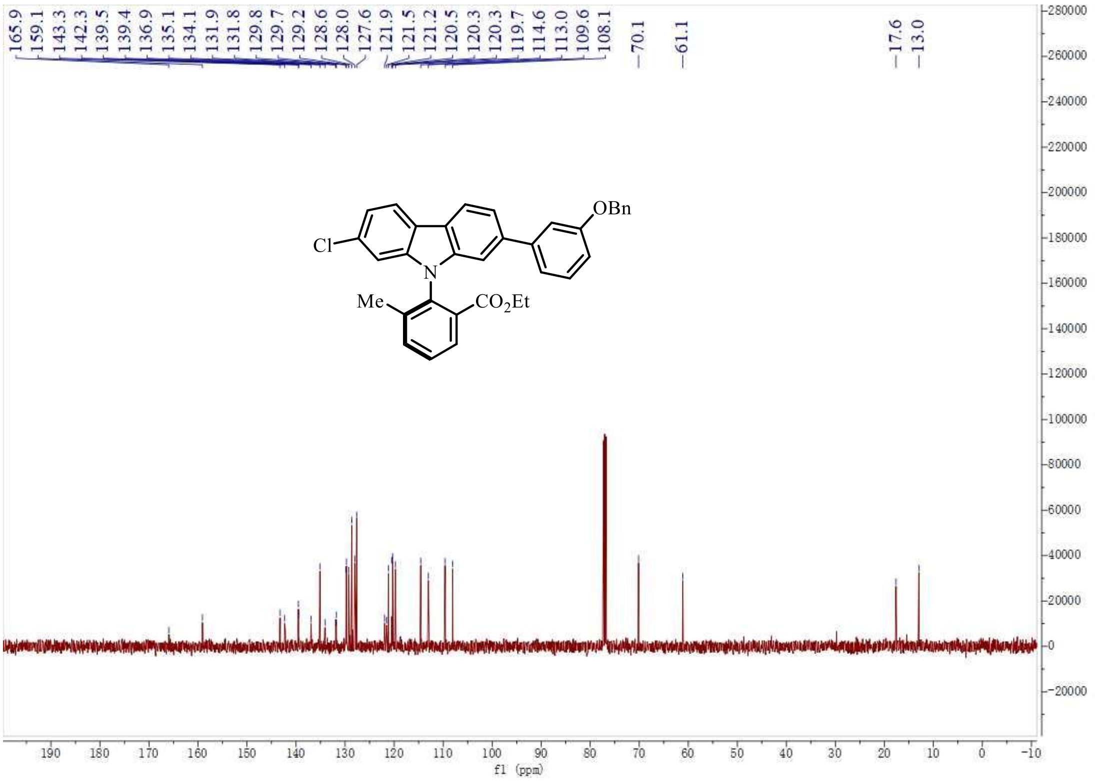

Ethyl (*R*)2-(2-chloro-7-(3-methoxyphenyl)-9*H*-carbazol-9-yl)-3-methylbenzoate (9)

^{1}H NMR (500 MHz, Chloroform-*d*)

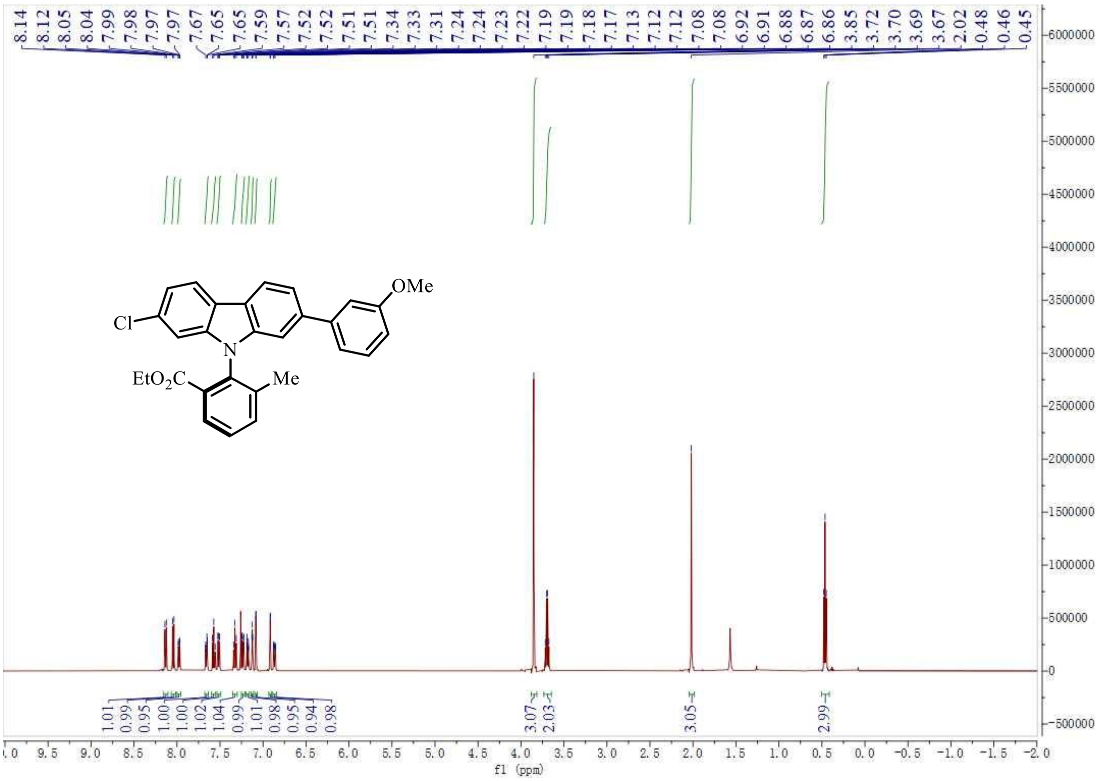

^{13}C NMR (126 MHz, Chloroform-*d*)

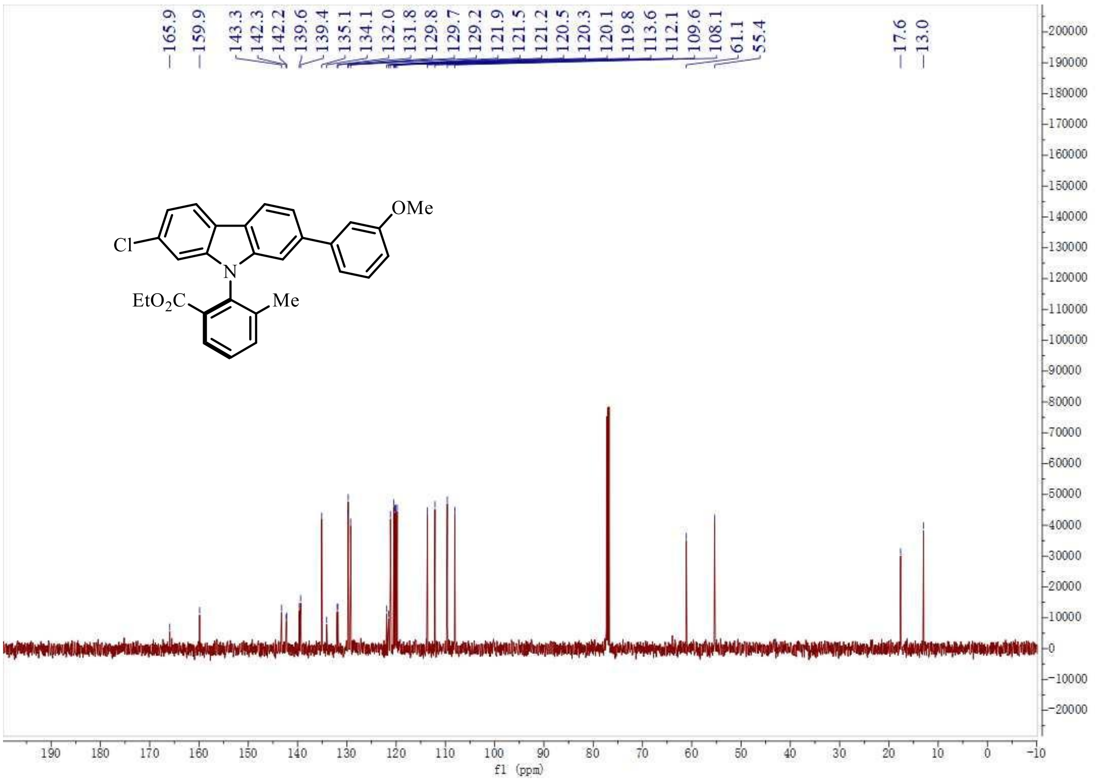

Ethyl (*R*)-2-(2-([1,1'-biphenyl]-2-yl)-7-chloro-9*H*-carbazol-9-yl)-3-methylbenzoate (10)

^{1}H NMR (500 MHz, Chloroform-*d*)

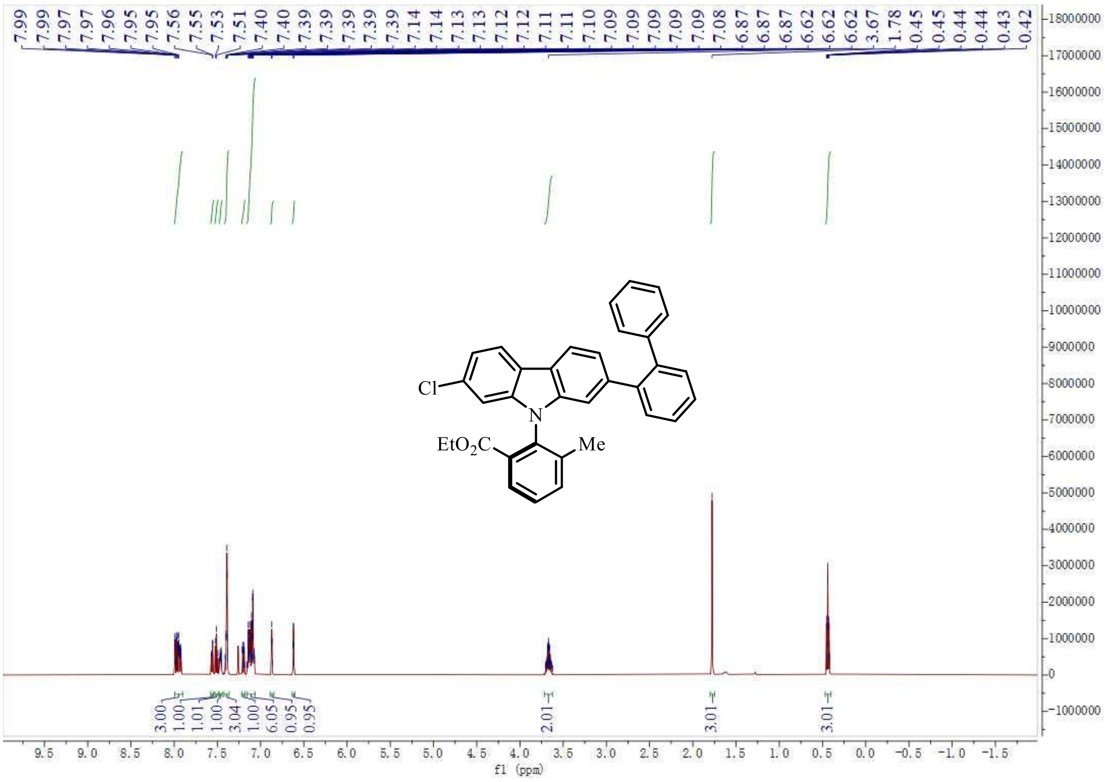

^{13}C NMR (126 MHz, Chloroform-*d*)

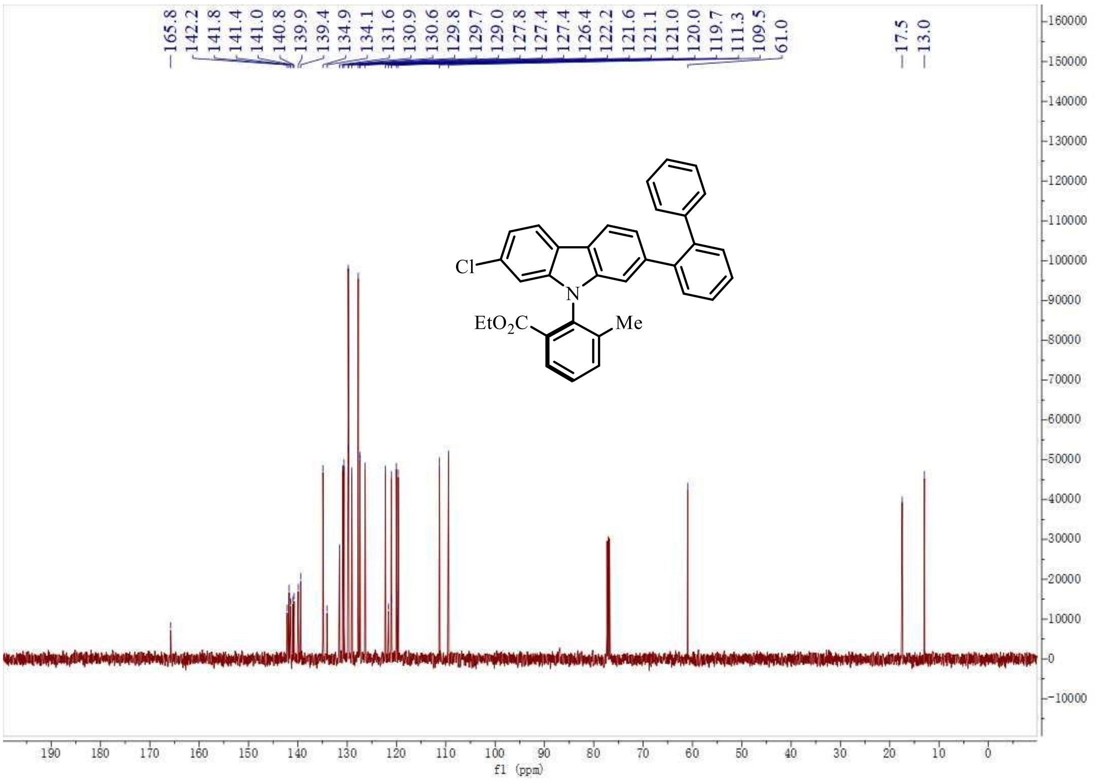

Ethyl (*S*)-2-(2-chloro-7-(6-methoxypyridin-3-yl)-9*H*-carbazol-9-yl)-3-methylbenzoate (11)

^{1}H NMR (500 MHz, Chloroform-*d*)

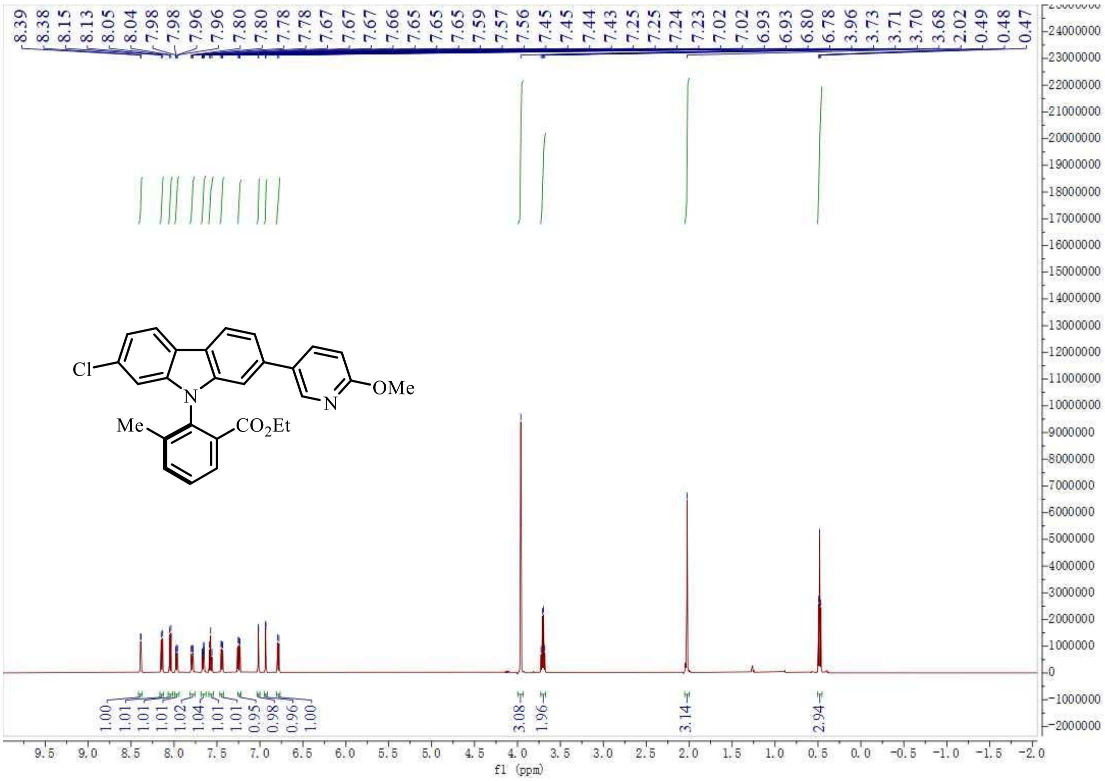

^{13}C NMR (126 MHz, Chloroform-*d*)

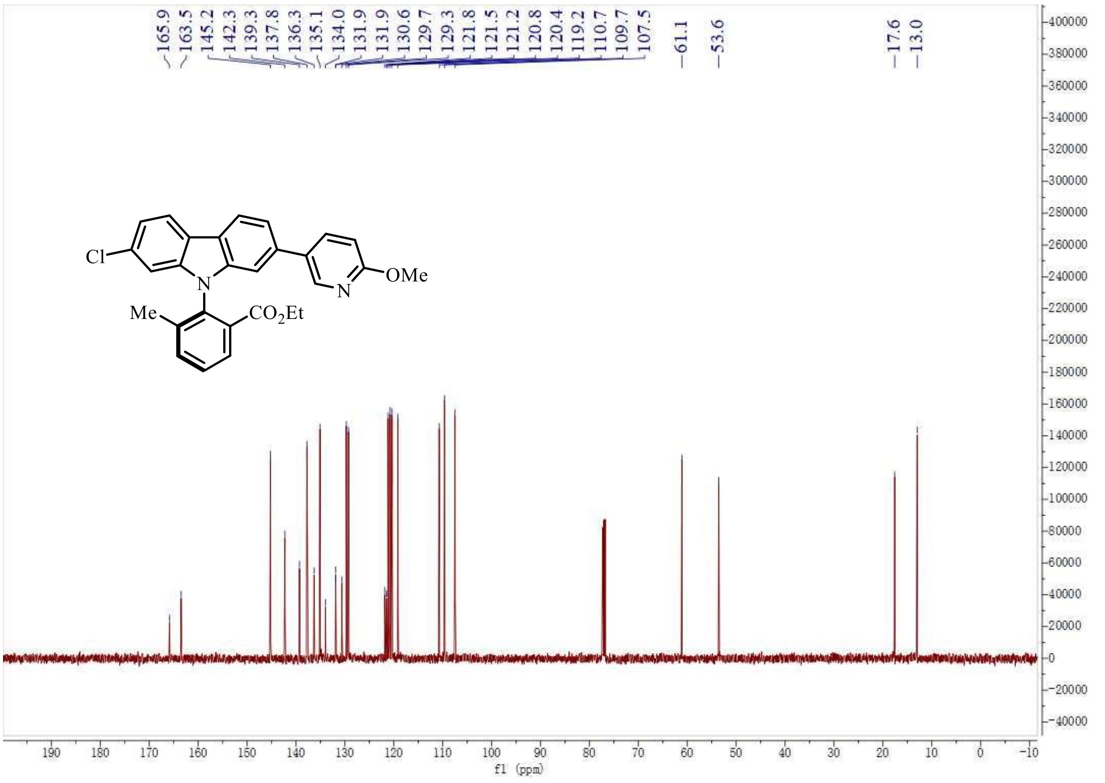

Ethyl (*R*)-2-(2-chloro-7-(furan-3-yl)-9*H*-carbazol-9-yl)-3-methylbenzoate (12)

^{1}H NMR (500 MHz, Chloroform-*d*)

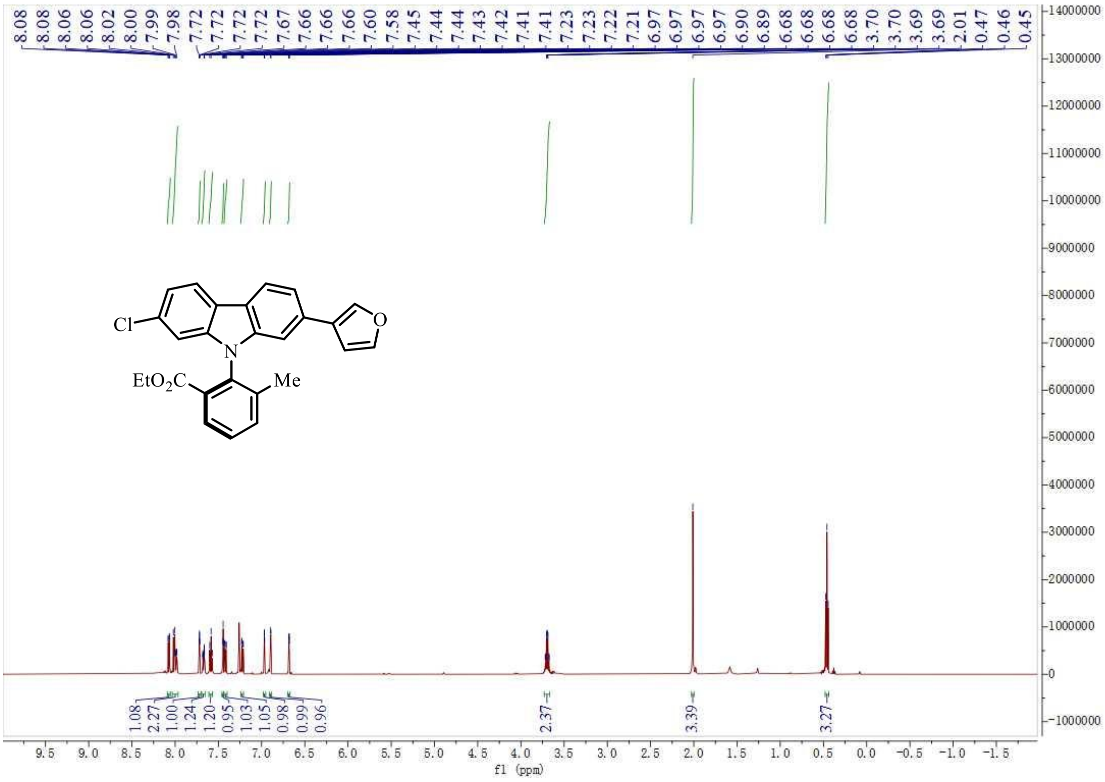

^{13}C NMR (126 MHz, Chloroform-*d*)

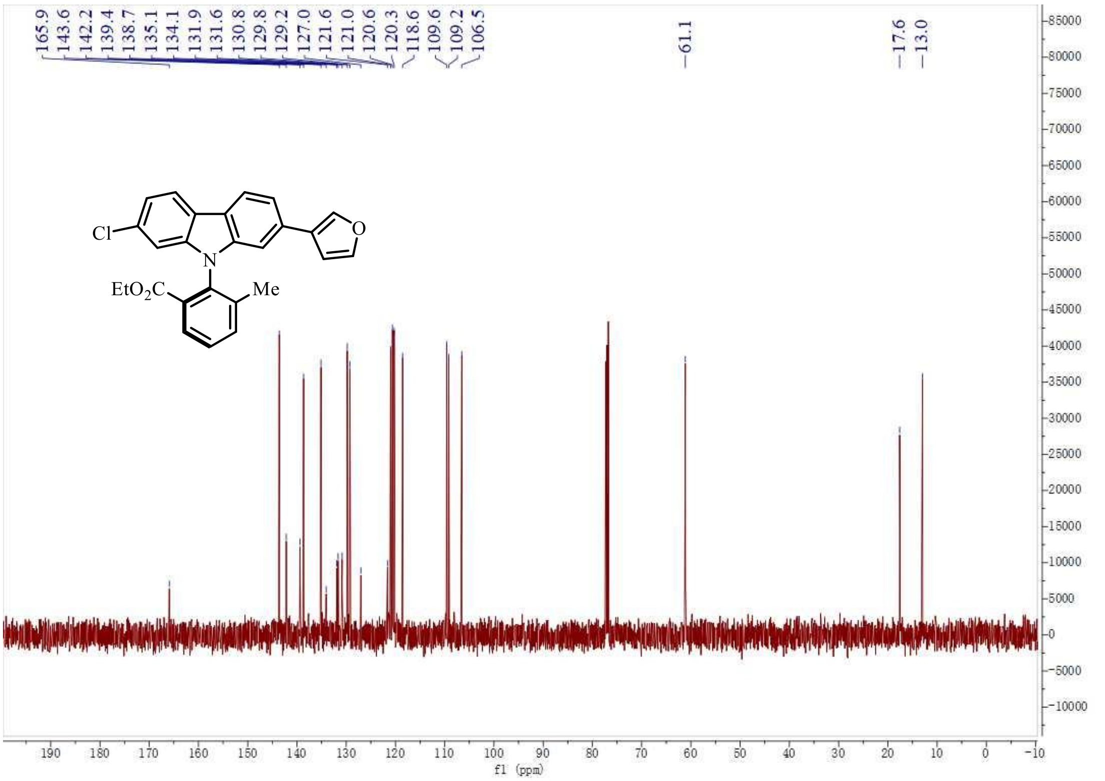

Ethyl (*S*)-2-(2-(5-acetylthiophen-2-yl)-7-chloro-9*H*-carbazol-9-yl)-3-methylbenzoate (13)

^{1}H NMR (500 MHz, Chloroform-*d*)

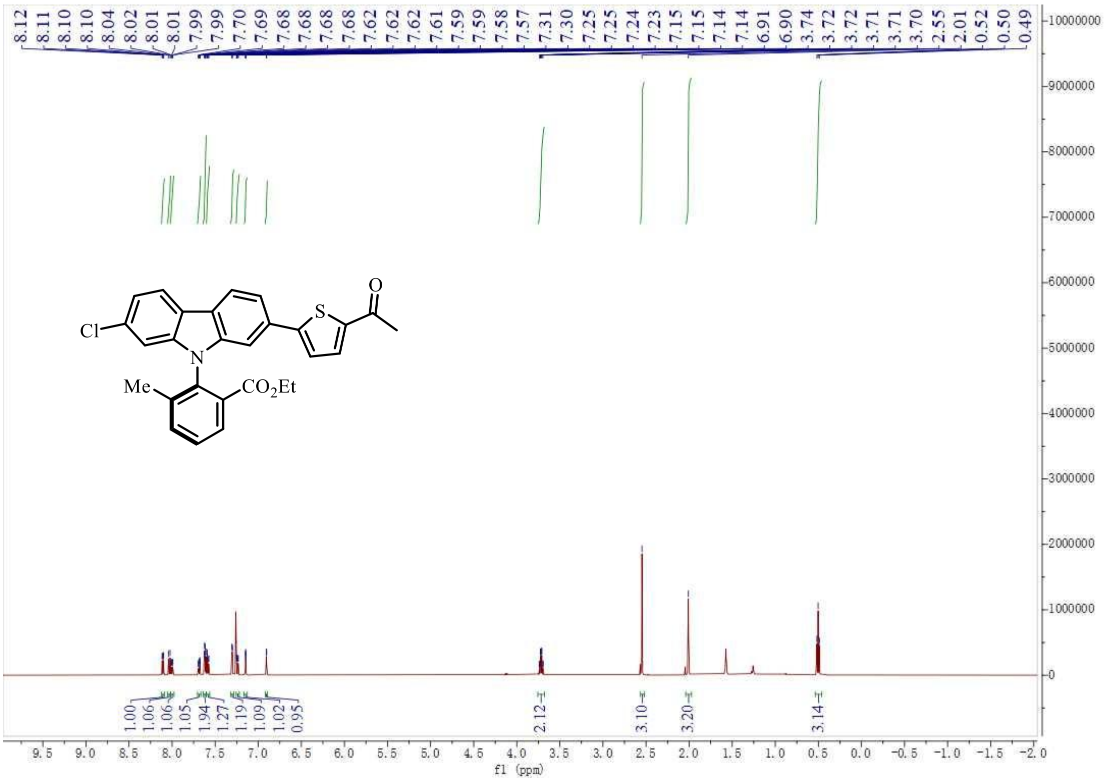

^{13}C NMR (126 MHz, Chloroform-*d*)

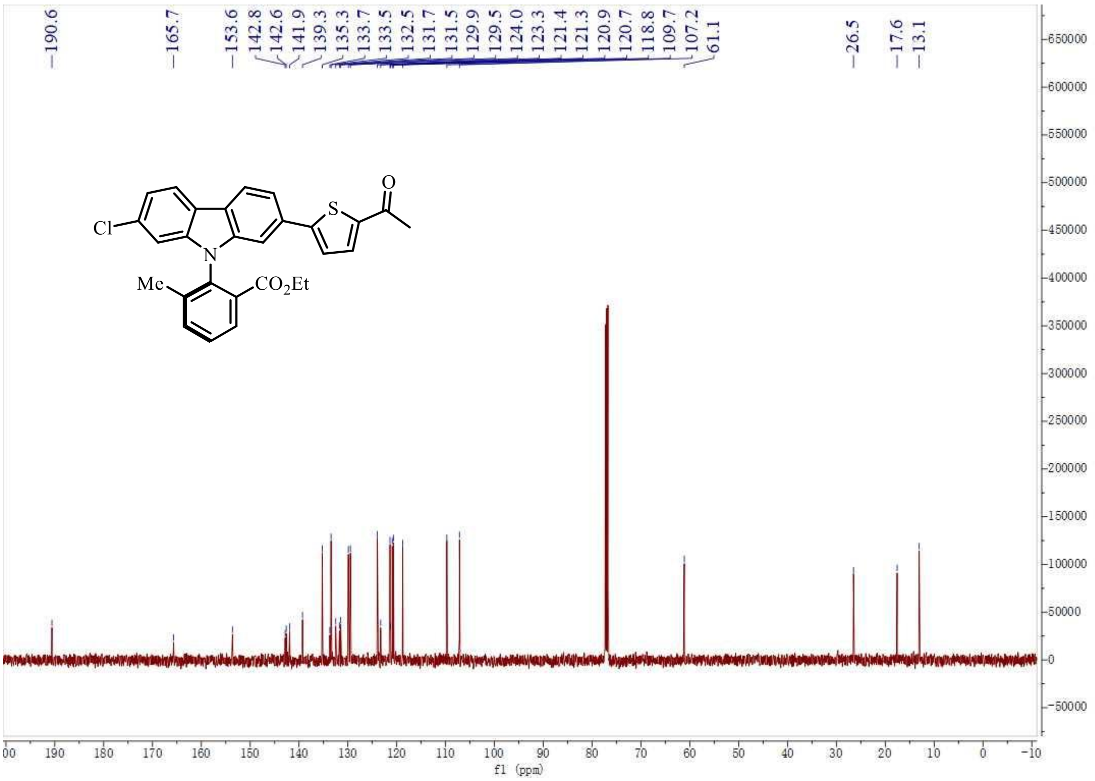

Ethyl (*S*)-2-(2-chloro-7-(thiophen-3-yl)-9*H*-carbazol-9-yl)-3-methylbenzoate (14)

^{1}H NMR (500 MHz, Chloroform-*d*)

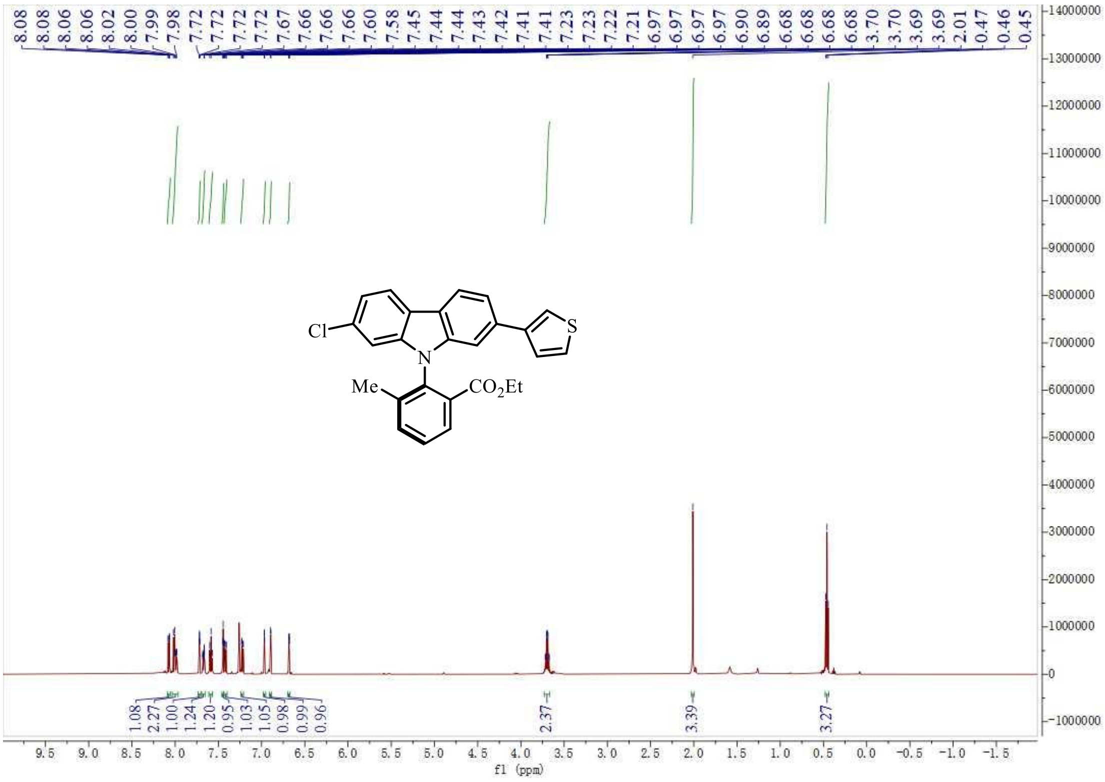

^{13}C NMR (126 MHz, Chloroform-*d*)

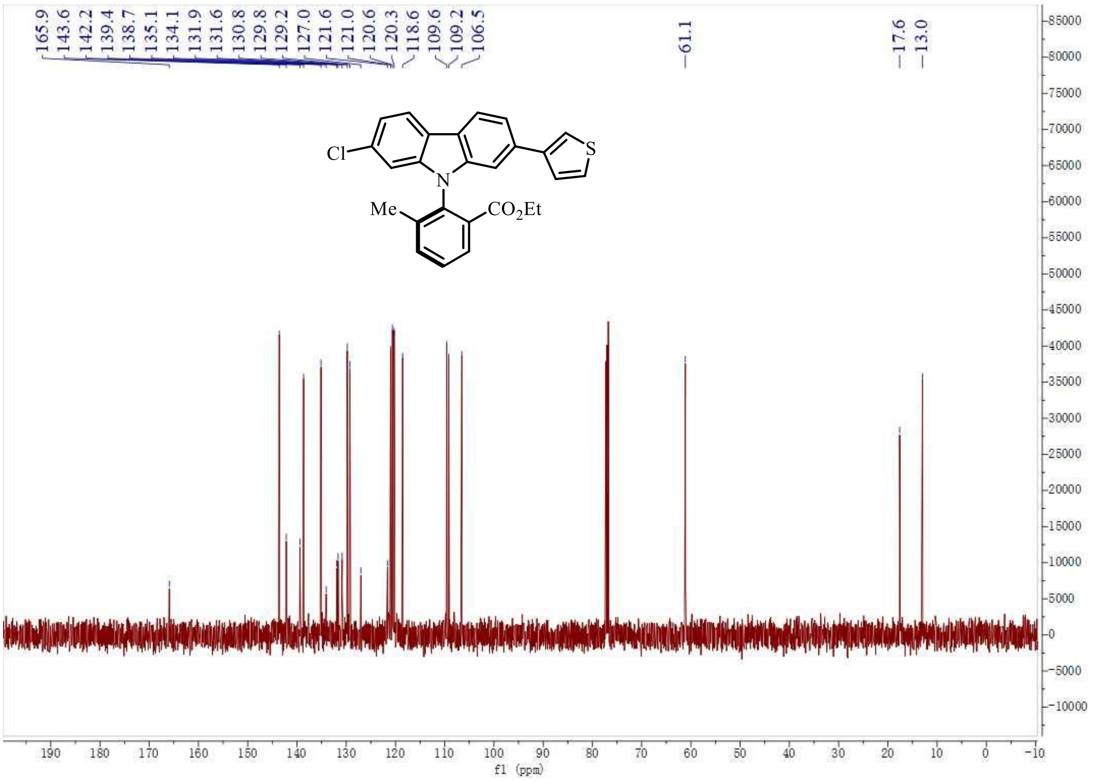

Ethyl (*R*)-2-(2-chloro-7-(4,6-dihydropyren-1-yl)-9*H*-carbazol-9-yl)-3-methylbenzoate (15)

^{1}H NMR (500 MHz, Chloroform-*d*)

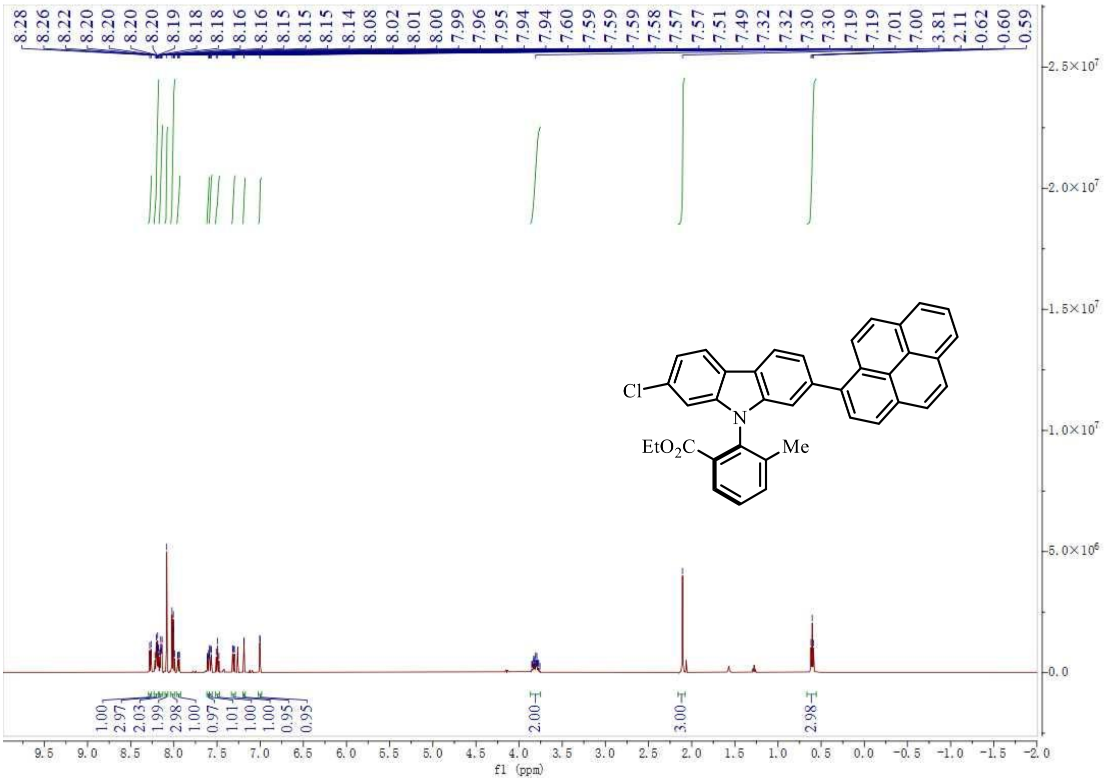

^{13}C NMR (126 MHz, Chloroform-*d*)

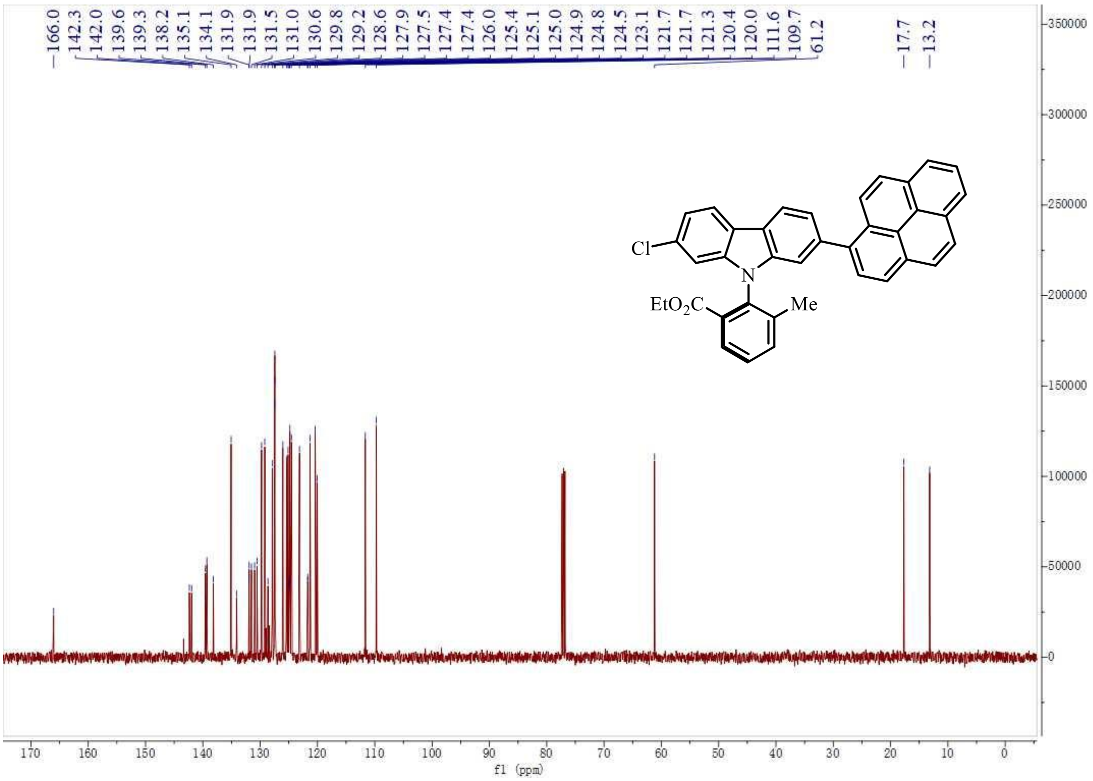

Ethyl (*R*)-2-(2-chloro-7-(9,9-dimethyl-9*H*-fluoren-1-yl)-9*H*-carbazol-9-yl)-3-methylbenzoate

(16) ^{1}H NMR (500 MHz, Chloroform-*d*)

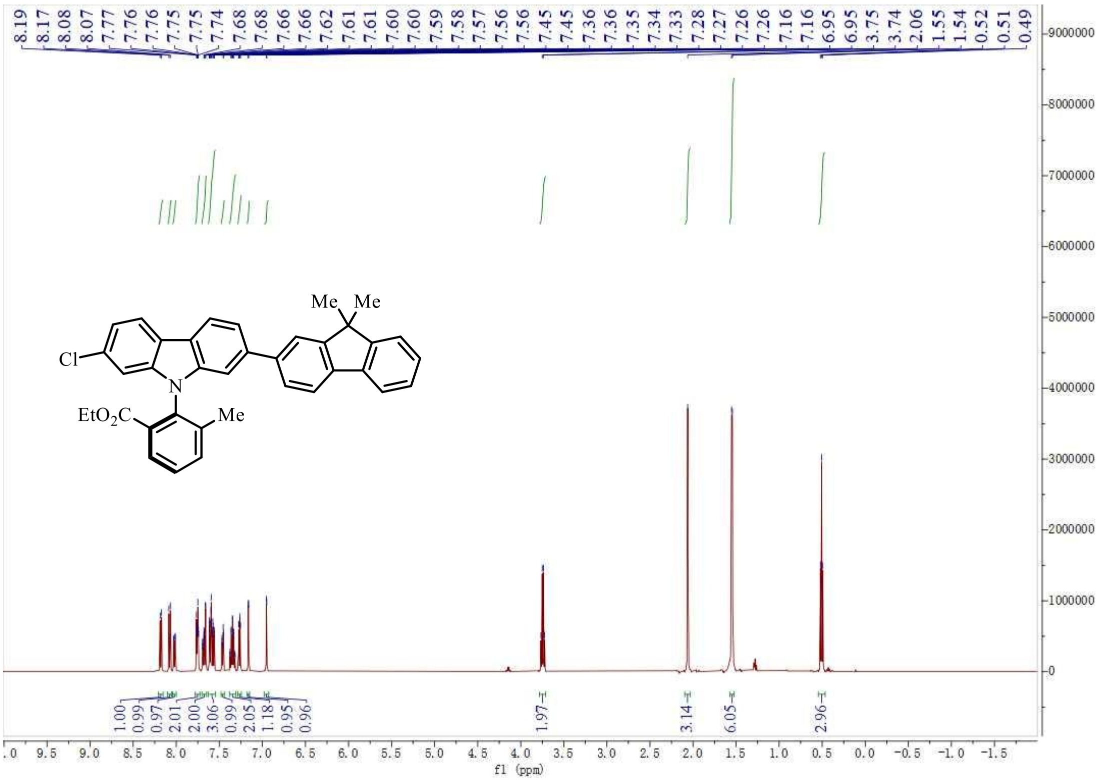

^{13}C NMR (126 MHz, Chloroform-*d*)

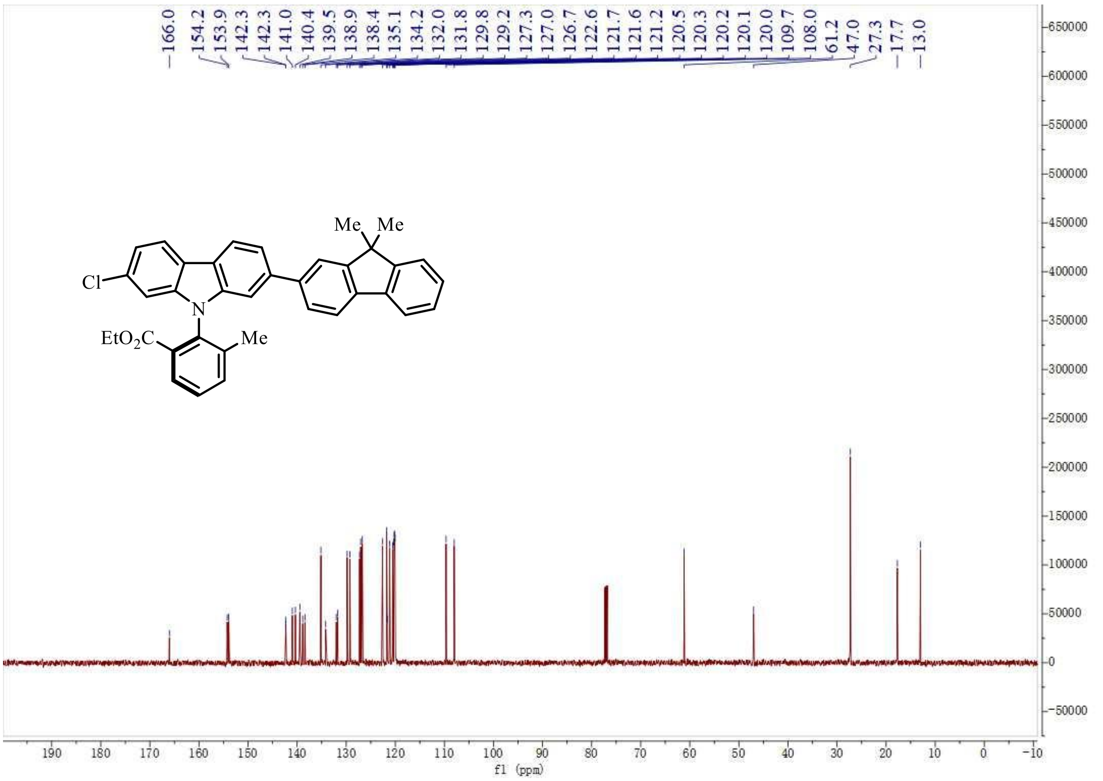

Ethyl (*R*)-2-(2-chloro-7-(naphthalen-2-yl)-9*H*-carbazol-9-yl)-3-methylbenzoate (17)

^{1}H NMR (500 MHz, Chloroform-*d*)

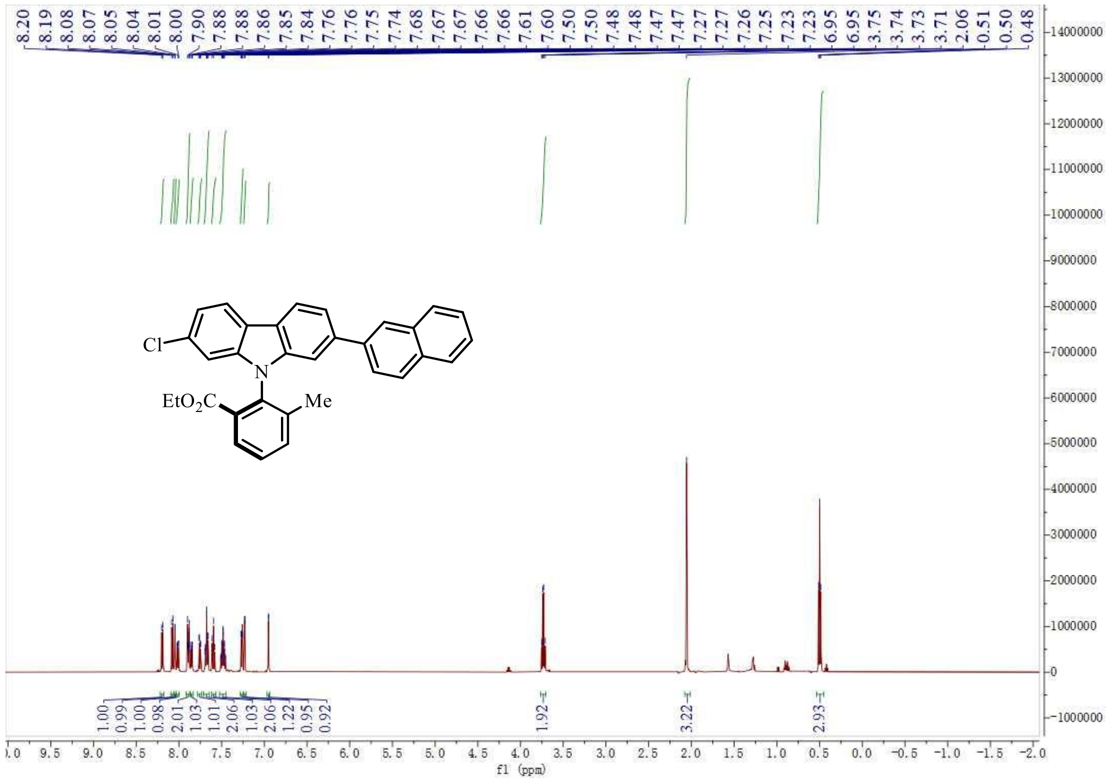

^{13}C NMR (126 MHz, Chloroform-*d*)

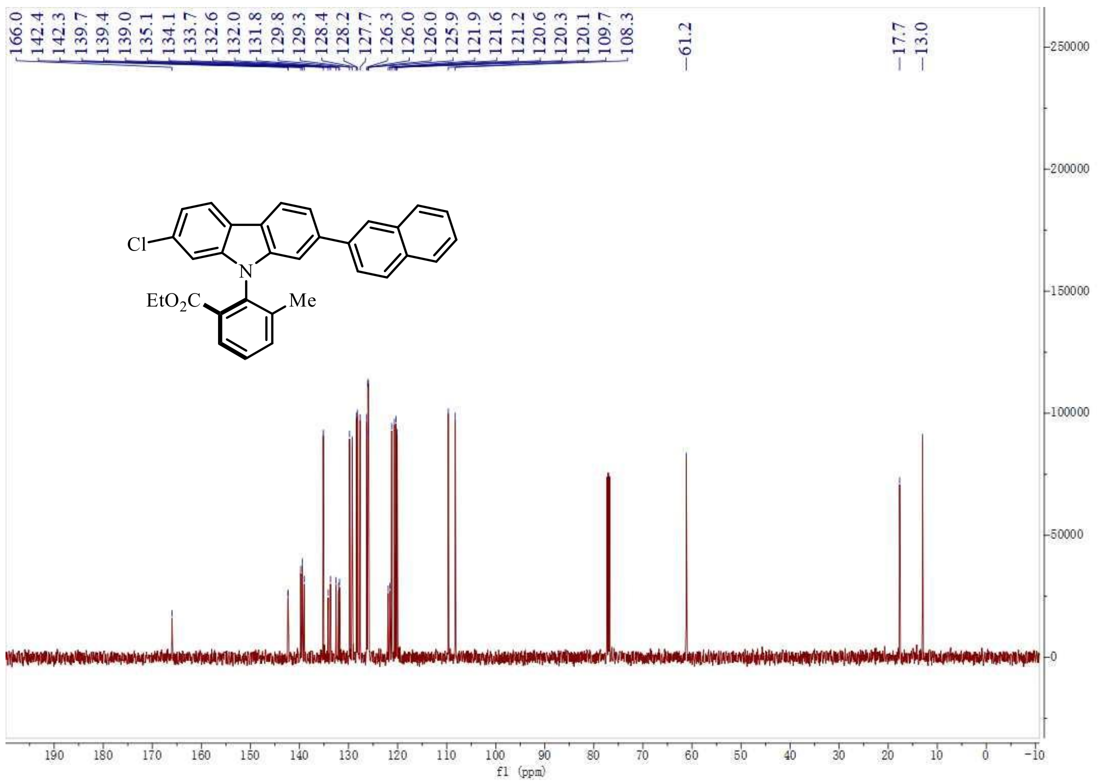

Ethyl (*R*)-2-(2-chloro-7-(dibenzo[*b*,*d*]thiophen-4-yl)-9*H*-carbazol-9-yl)-3-methylbenzoate (18)

^{1}H NMR (500 MHz, Chloroform-*d*)

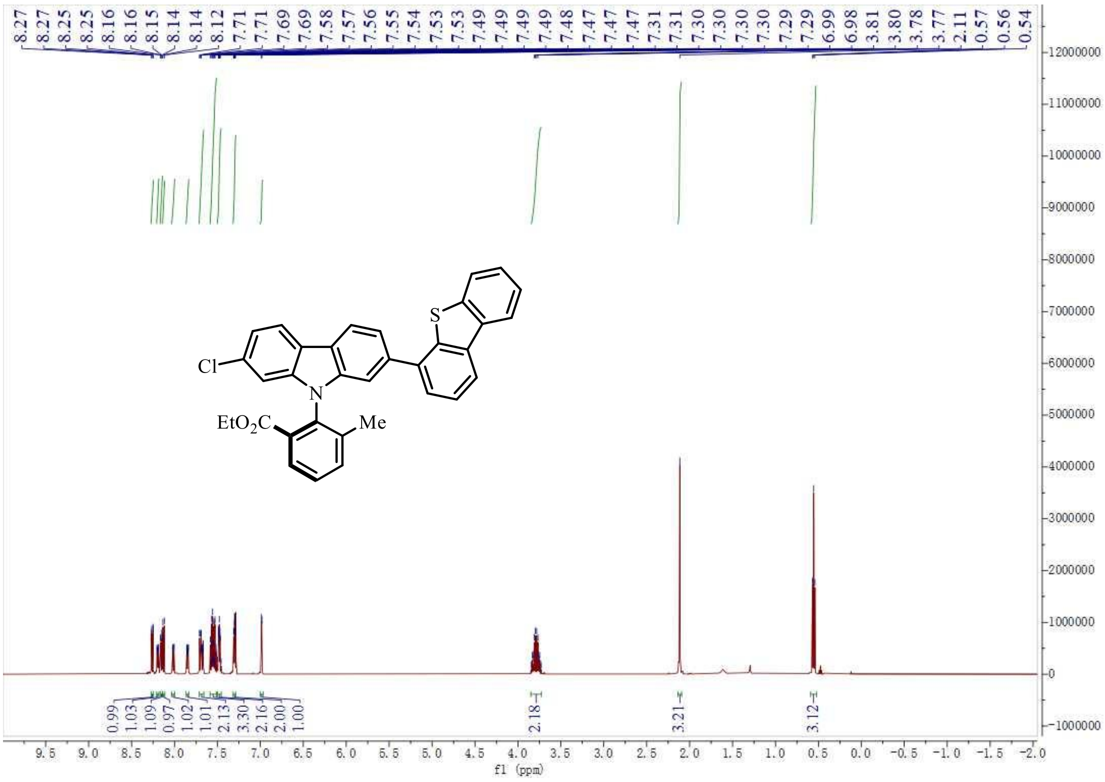

^{13}C NMR (126 MHz, Chloroform-*d*)

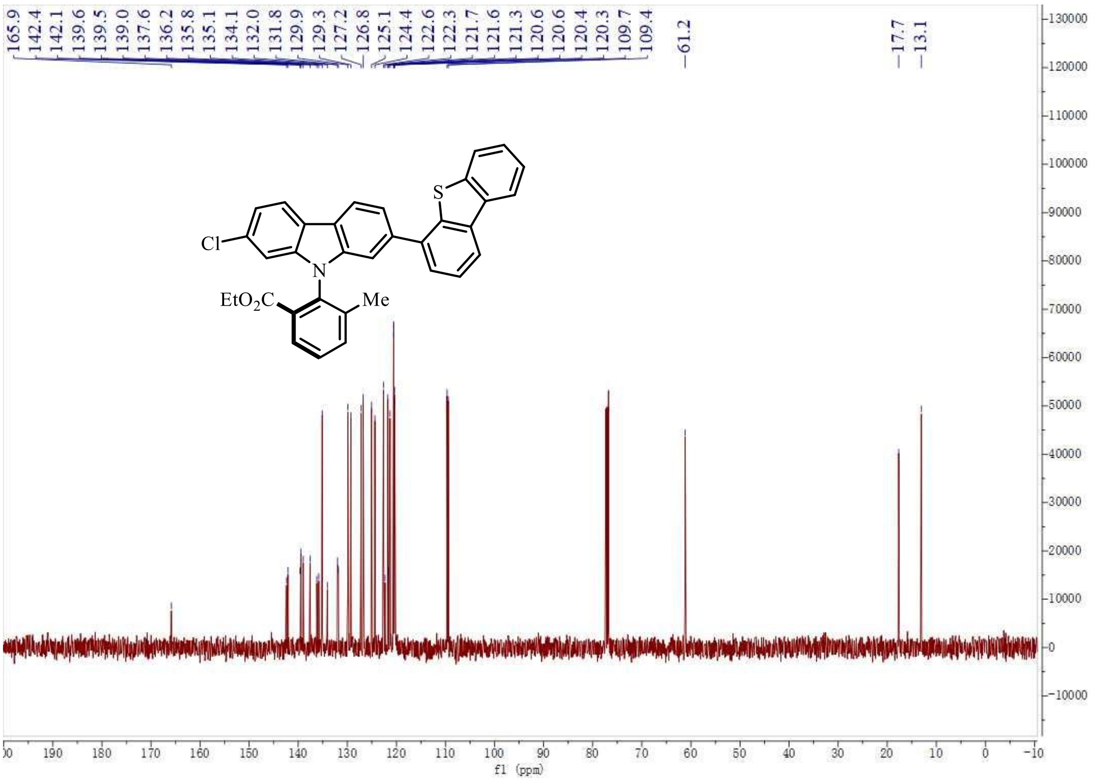

Methyl (*S*)-2-(2-chloro-7-(dibenzo[*b*,*d*]thiophen-3-yl)-9*H*-carbazol-9-yl)-3-methylbenzoate

(19) ^{1}H NMR (500 MHz, Chloroform-*d*)

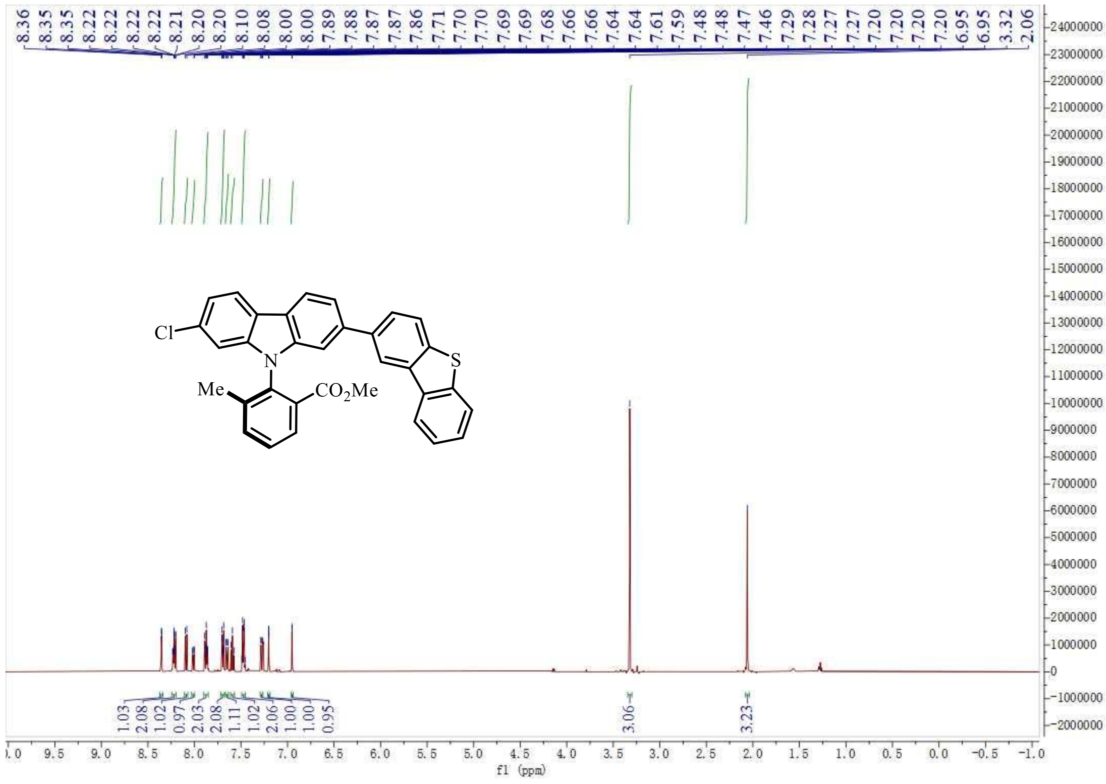

^{13}C NMR (126 MHz, Chloroform-*d*)

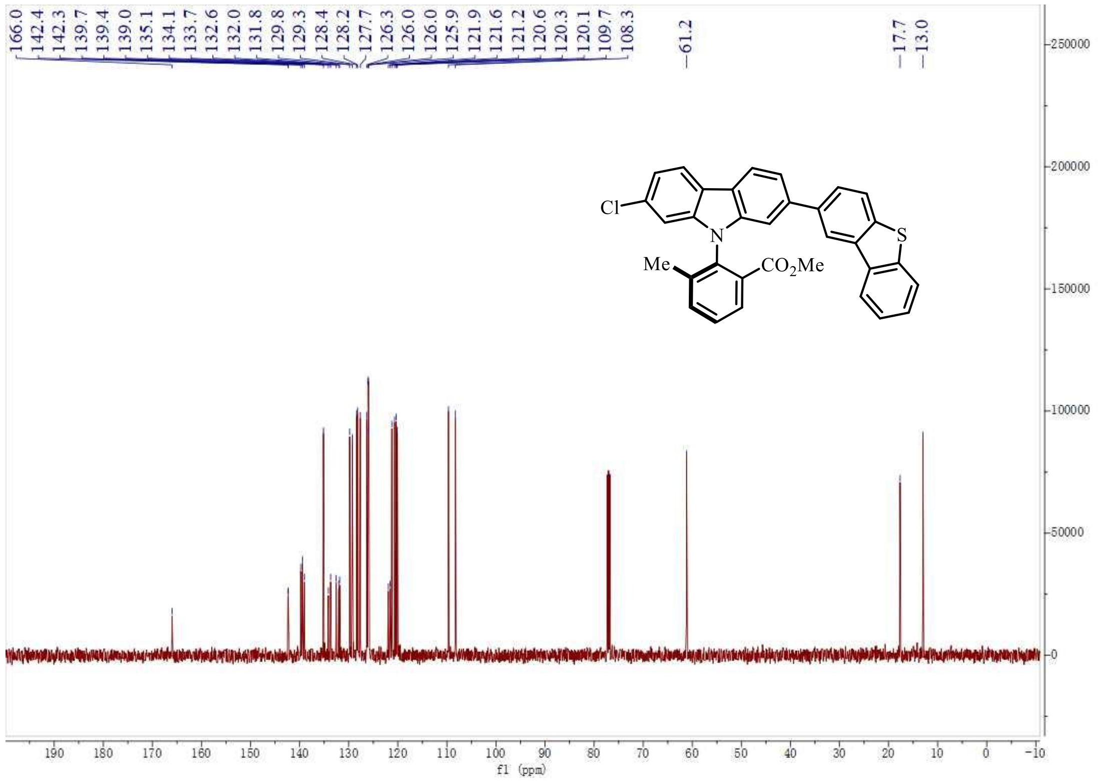

Ethyl (*R*)-2-(7-chloro-9'-phenyl-9*H*,9'*H*-[2,2'-bicarbazol]-9-yl)-3-methylbenzoate (20)

^{1}H NMR (500 MHz, Chloroform-*d*)

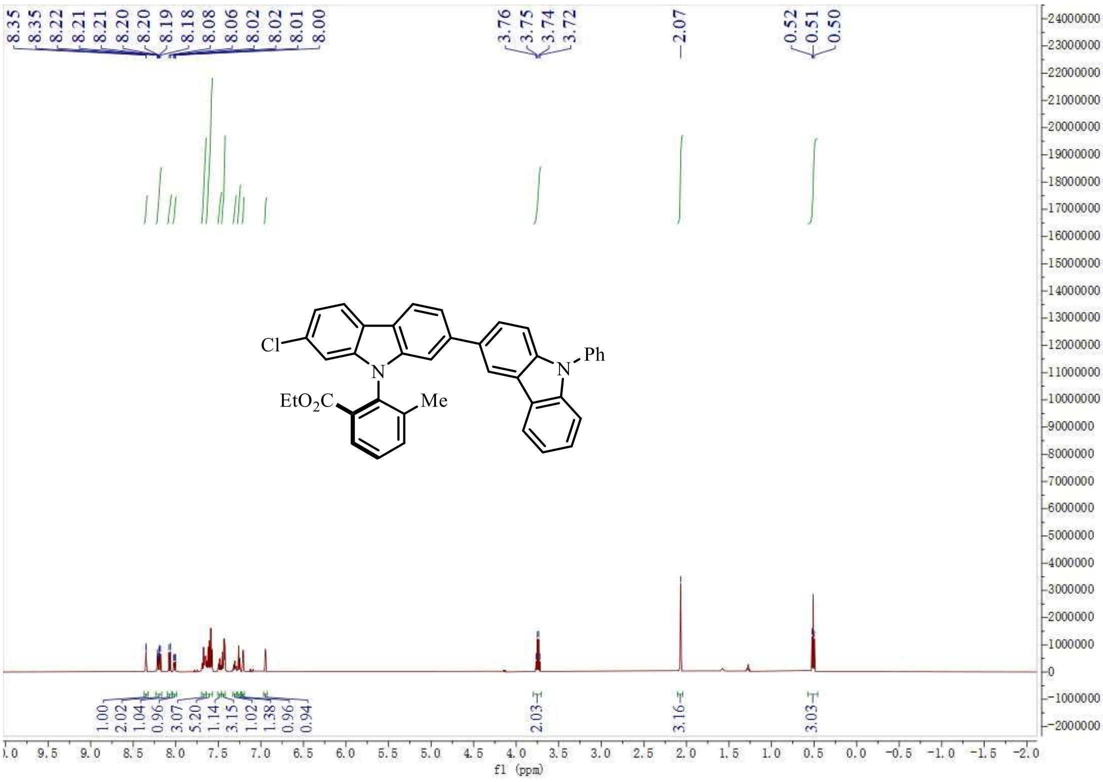

^{13}C NMR (126 MHz, Chloroform-*d*)

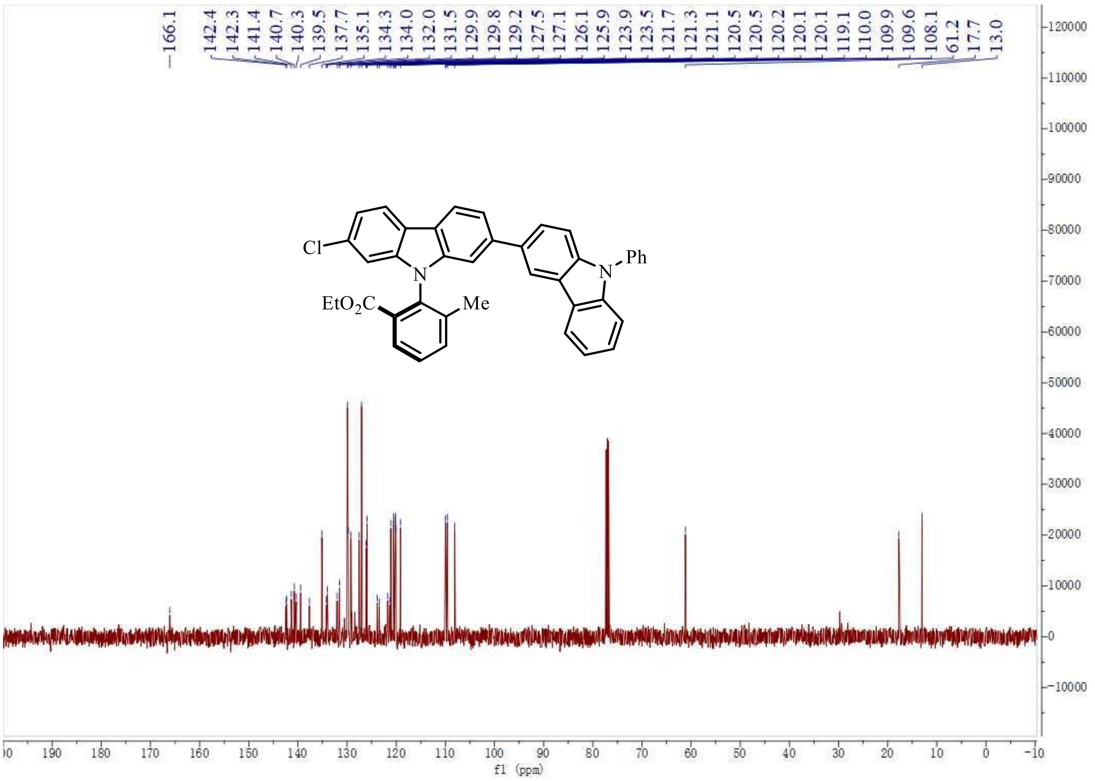

Ethyl (*R*)-2-(2-chloro-7-((4-methoxyphenyl)amino)-9*H*-carbazol-9-yl)-3-methylbenzoate (21)

^{1}H NMR (500 MHz, Chloroform-*d*)

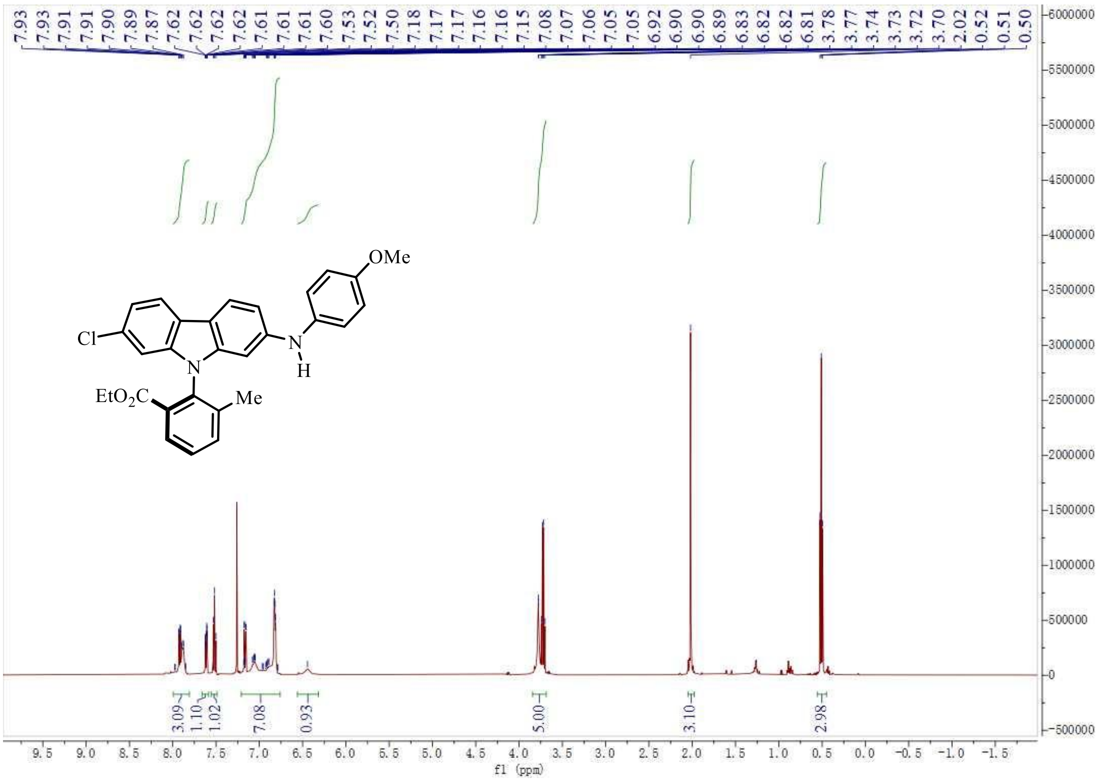

^{13}C NMR (126 MHz, Chloroform-*d*)

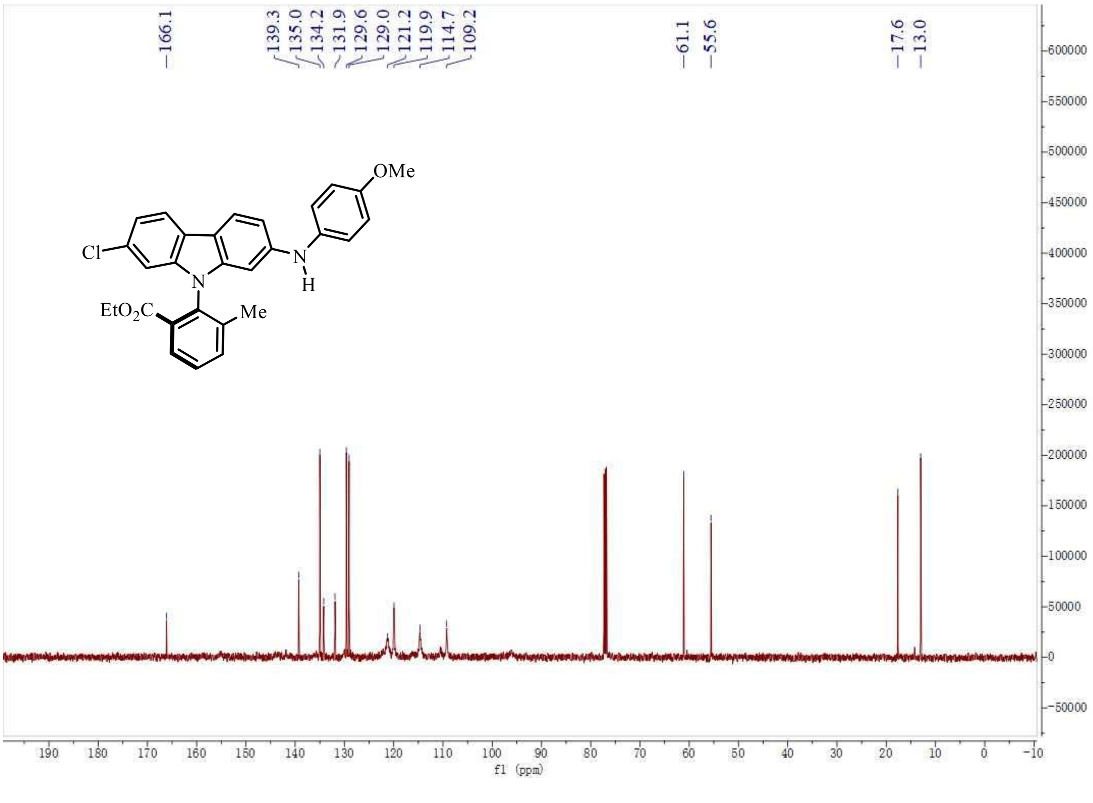

Ethyl (*R*)-2-(2-chloro-7-(4-methoxyphenyl)-9*H*-carbazol-9-yl)-3'-methoxy-[1,1'-biphenyl]-3-carboxylate (22) ^{1}H NMR (500 MHz, Chloroform-*d*)

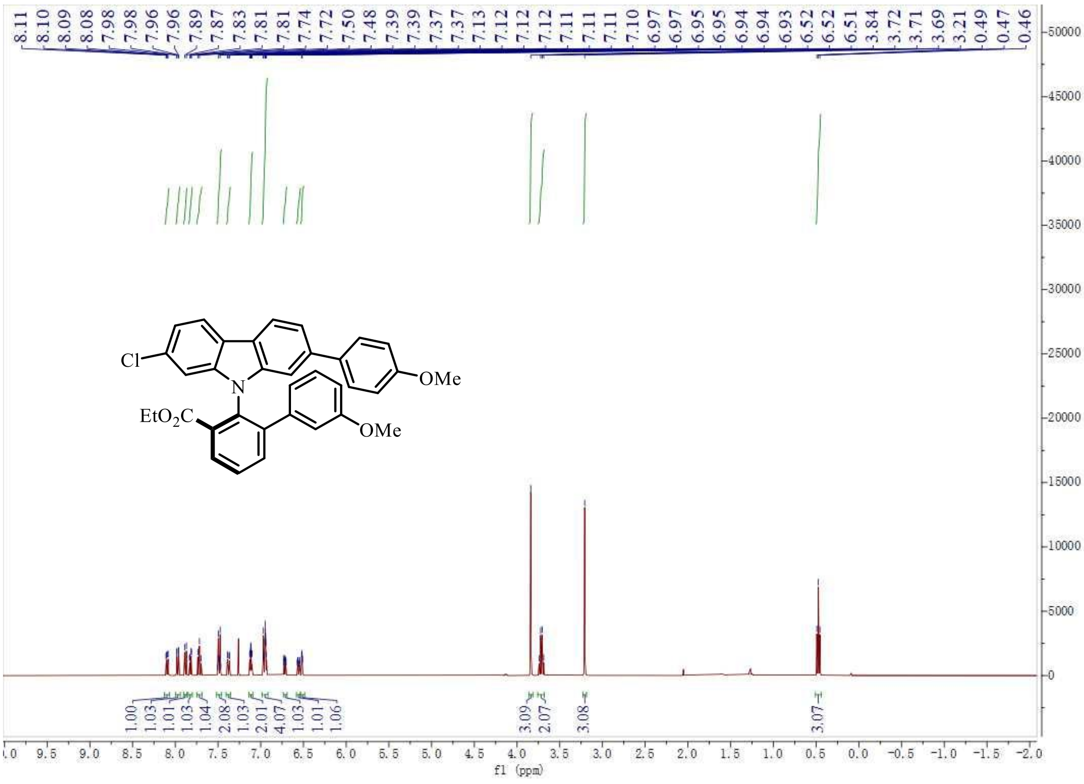

^{13}C NMR (126 MHz, Chloroform-*d*)

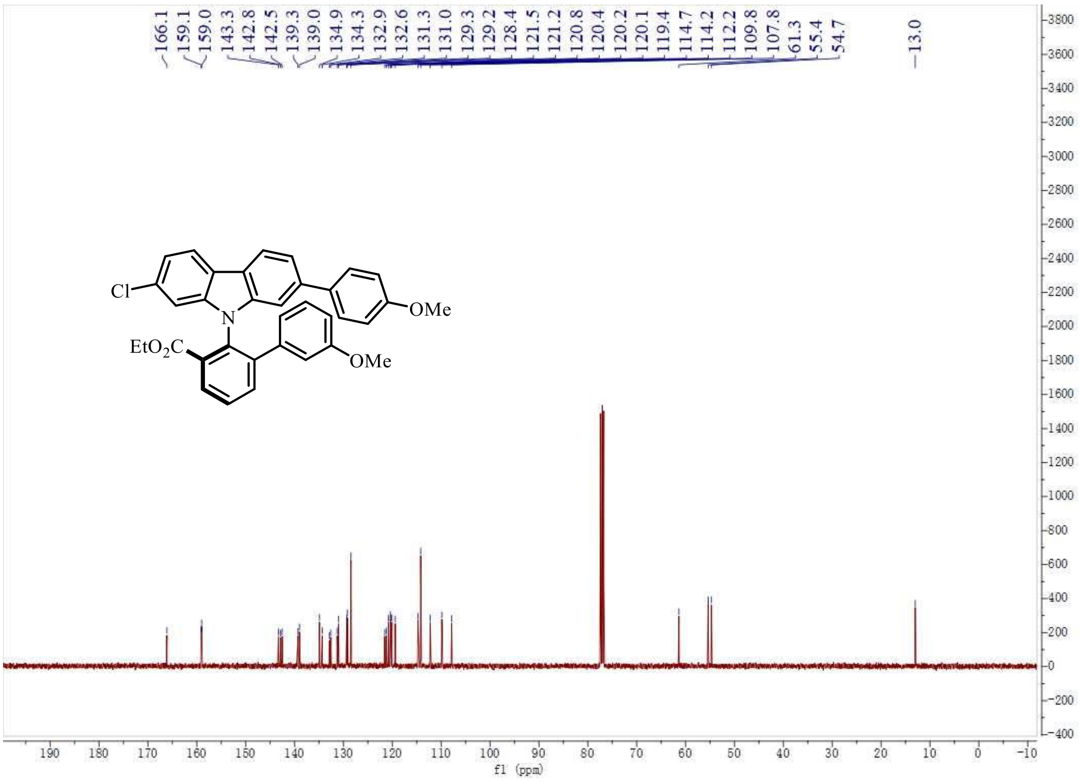

Ethyl (*R*)-2-(2-chloro-7-(4-methoxyphenyl)-9*H*-carbazol-9-yl)-3'-nitro-[1,1'-biphenyl]-3-carboxylate (23) ^{1}H NMR (500 MHz, Chloroform-*d*)

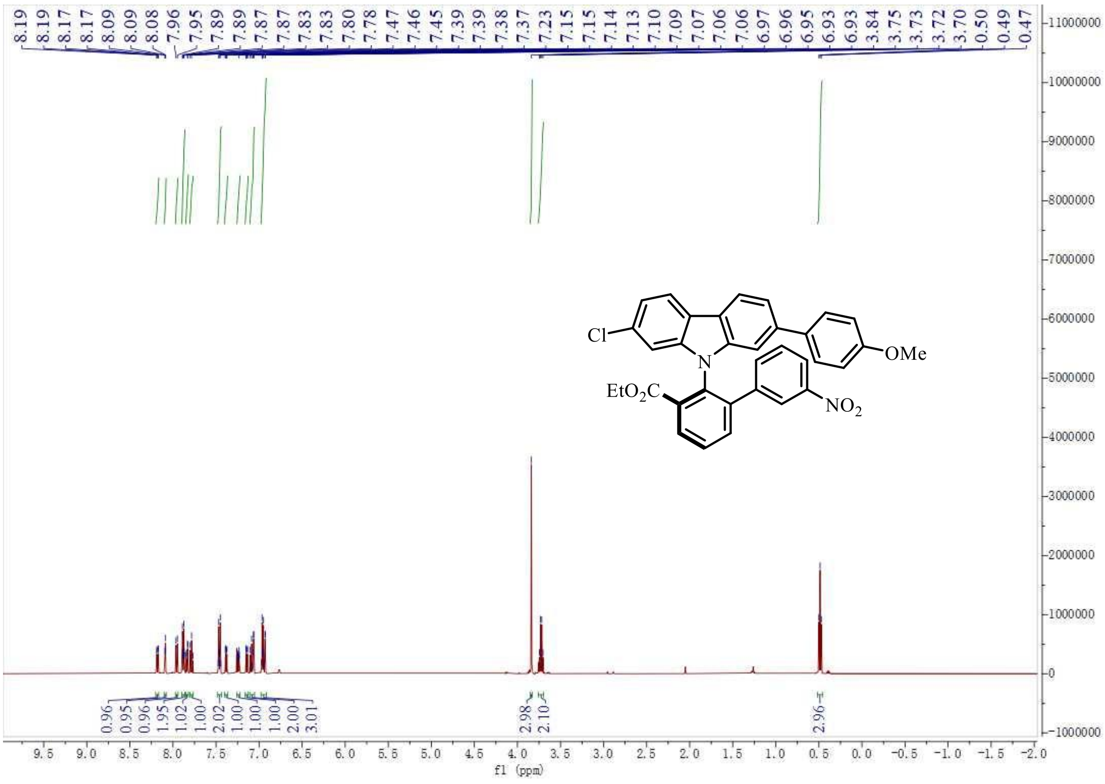

^{13}C NMR (126 MHz, Chloroform-*d*)

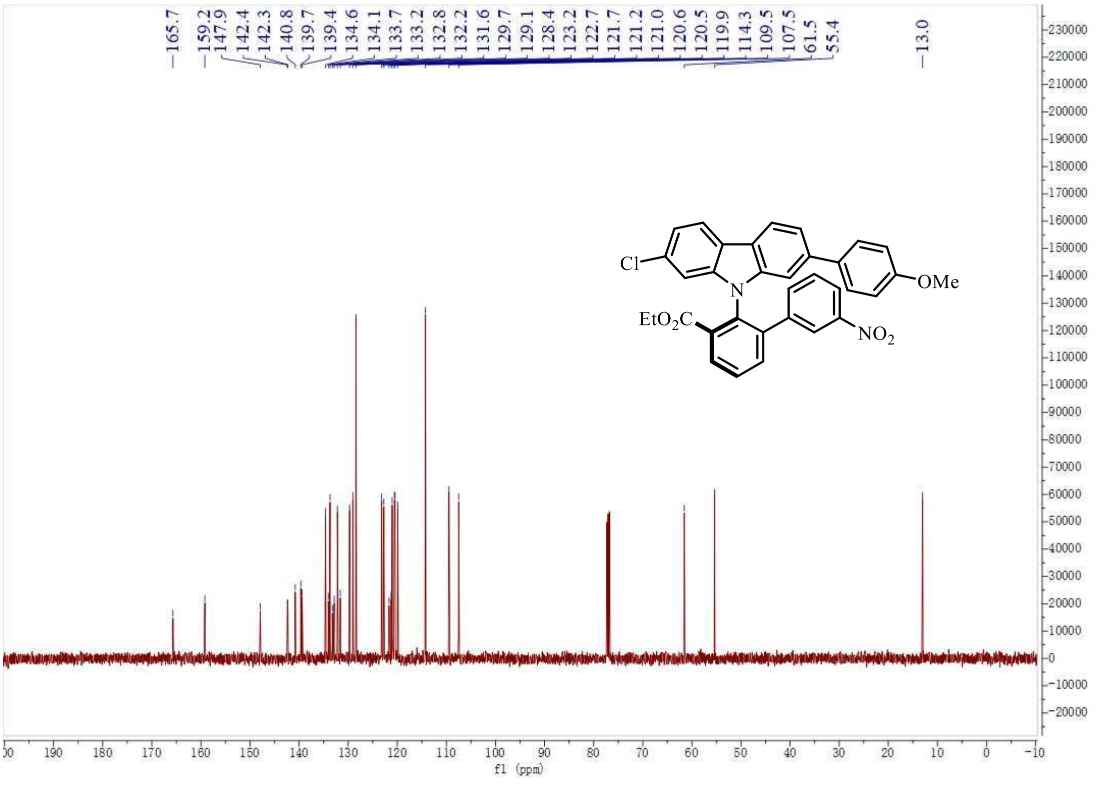

Ethyl (*R*)2-(2-chloro-7-(4-methoxyphenyl)-9*H*-carbazol-9-yl)-3-(naphthalen-1-yl)benzoate

(24) ^{1}H NMR (500 MHz, Chloroform-*d*)

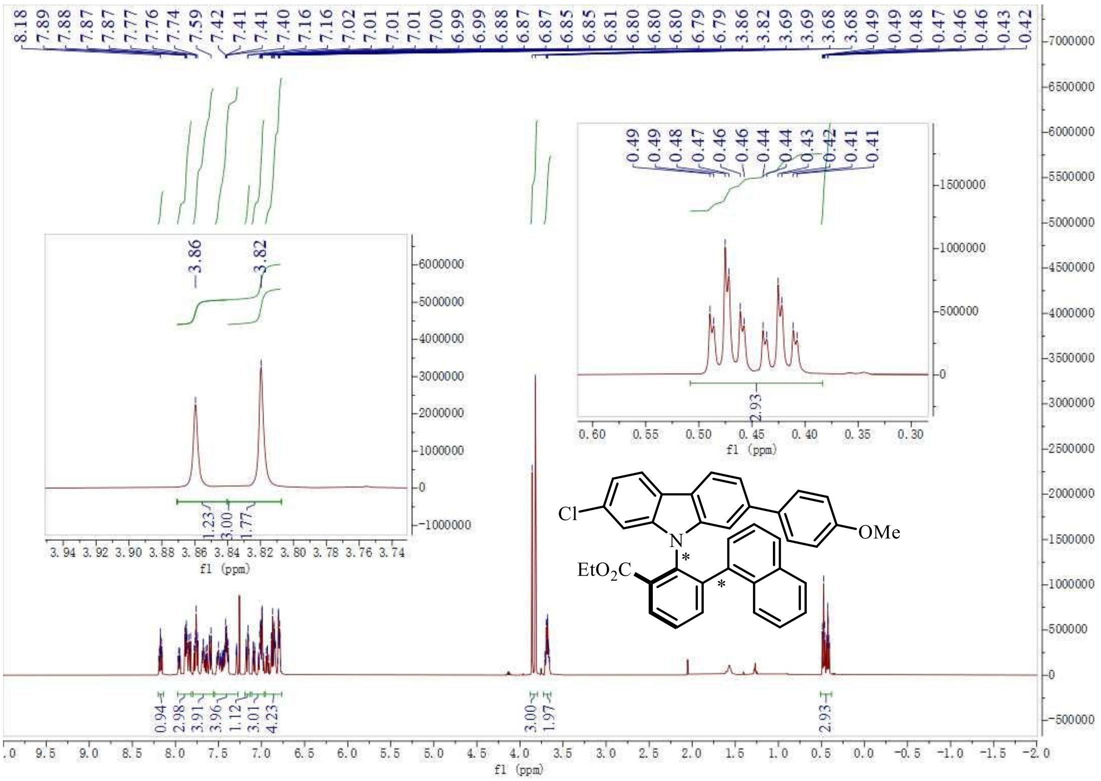

^{13}C NMR (126 MHz, Chloroform-*d*)

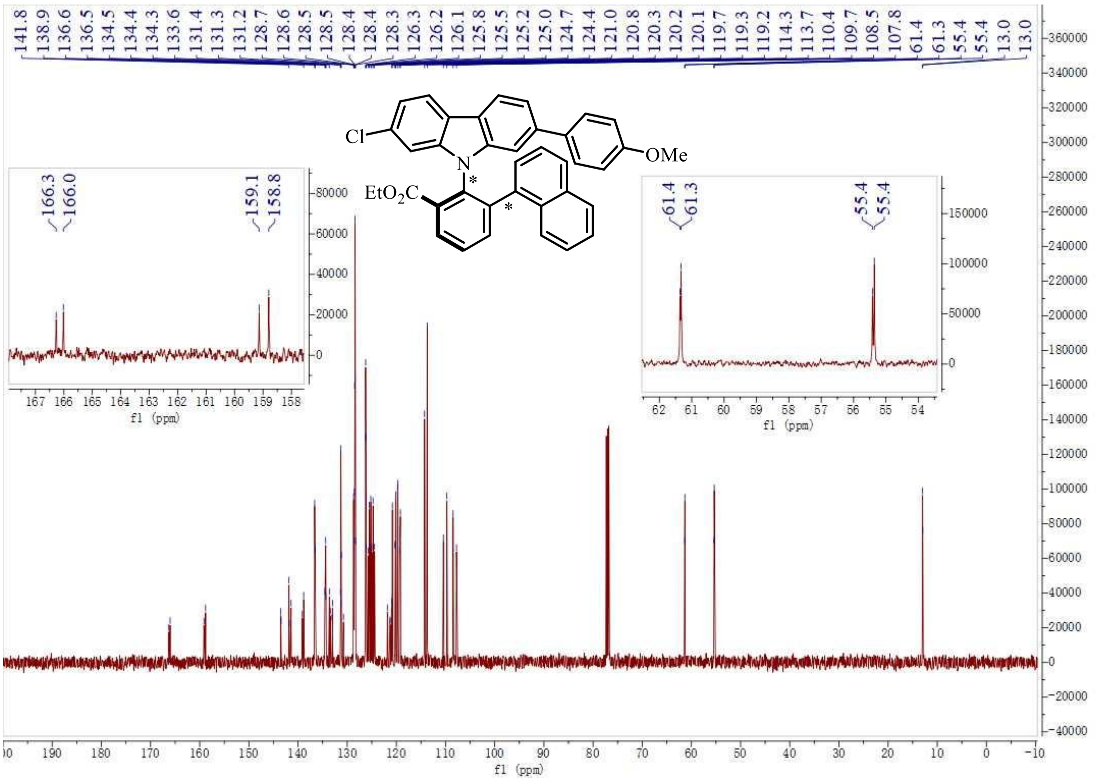

Ethyl (*R*)-2-(2-chloro-7-(4-methoxyphenyl)-9*H*-carbazol-9-yl)-2',3',4',5'-tetrahydro-[1,1'-biphenyl]-3-carboxylate (25) ^{1}H NMR (500 MHz, Chloroform-*d*)

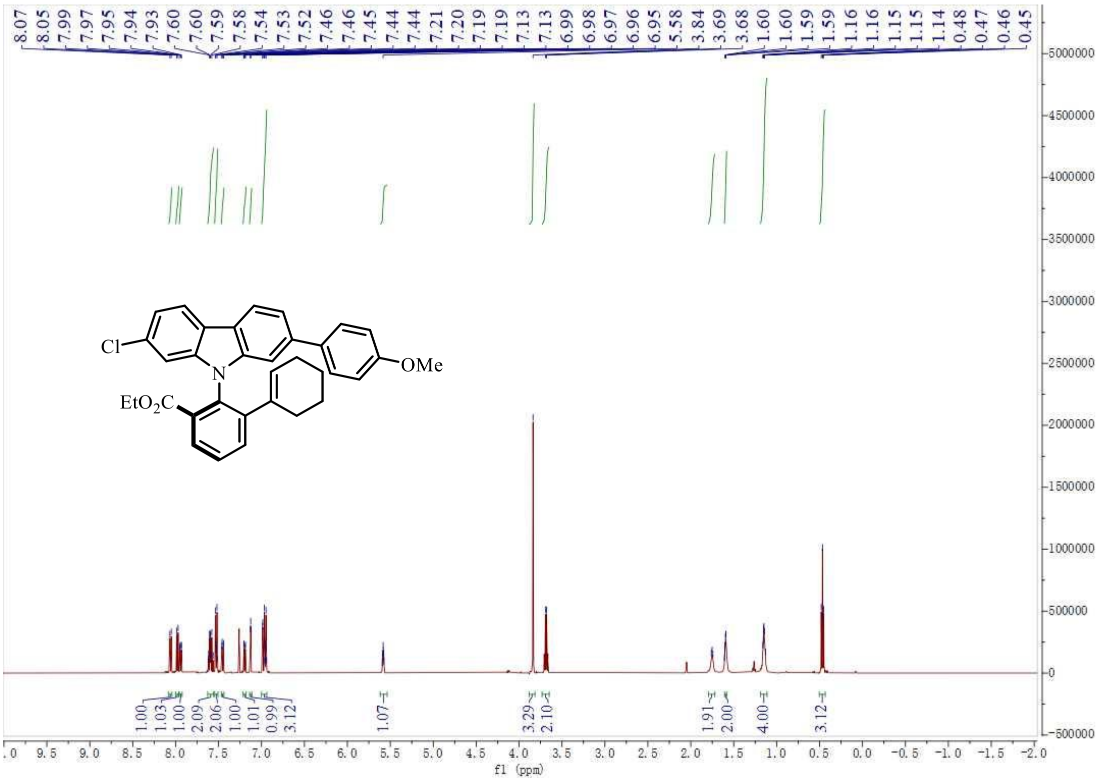

^{13}C NMR (126 MHz, Chloroform-*d*)

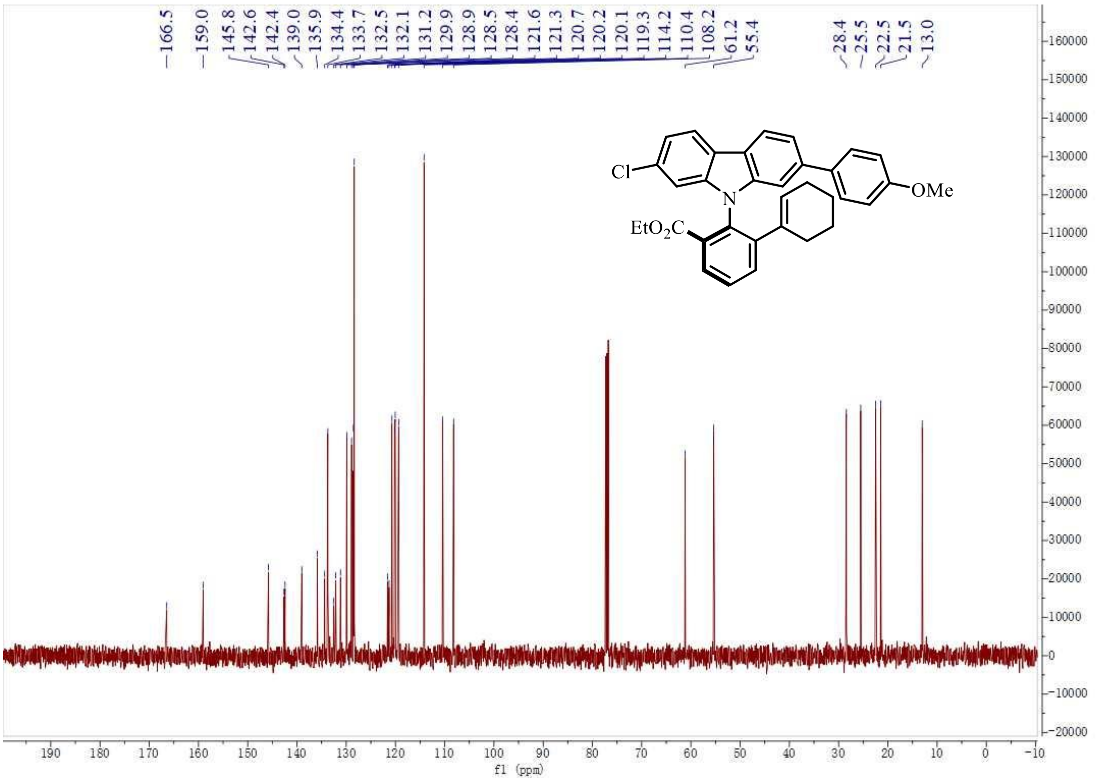

Ethyl (*R*)-2-(2-chloro-7-(4-methoxyphenyl)-9*H*-carbazol-9-yl)-3-(3,6-dihydro-2*H*-pyran-4-yl)benzoate (26) ^{1}H NMR (500 MHz, Chloroform-*d*)

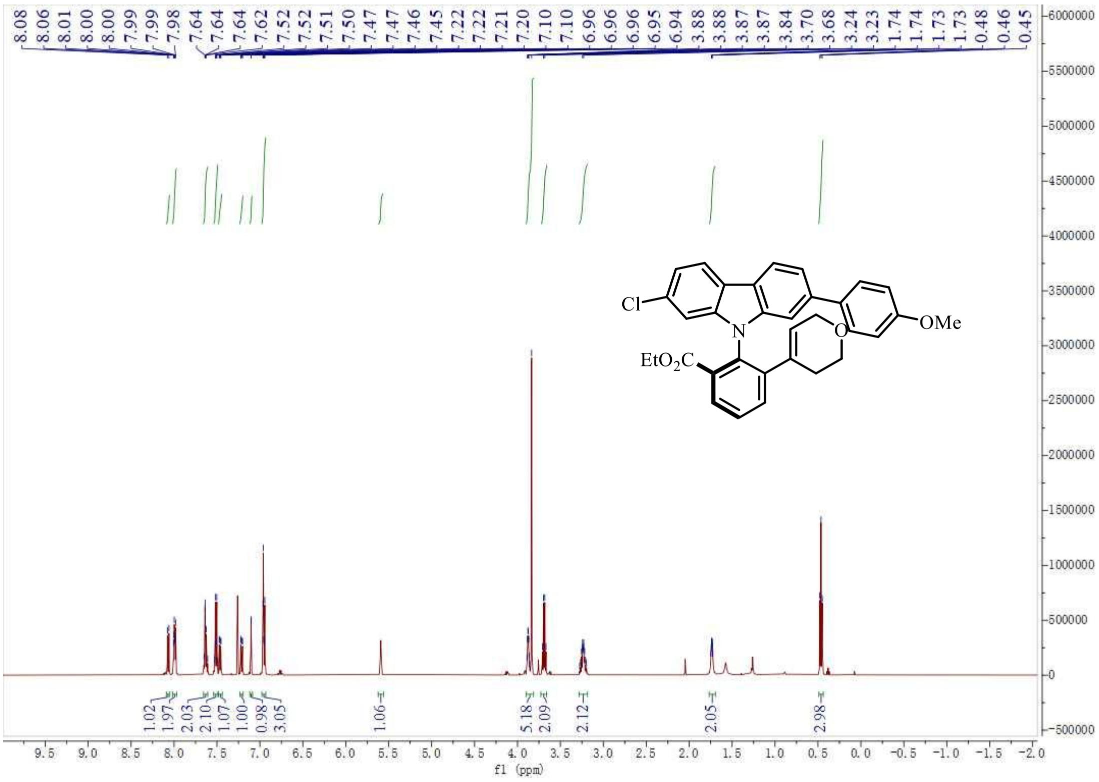

^{13}C NMR (126 MHz, Chloroform-*d*)

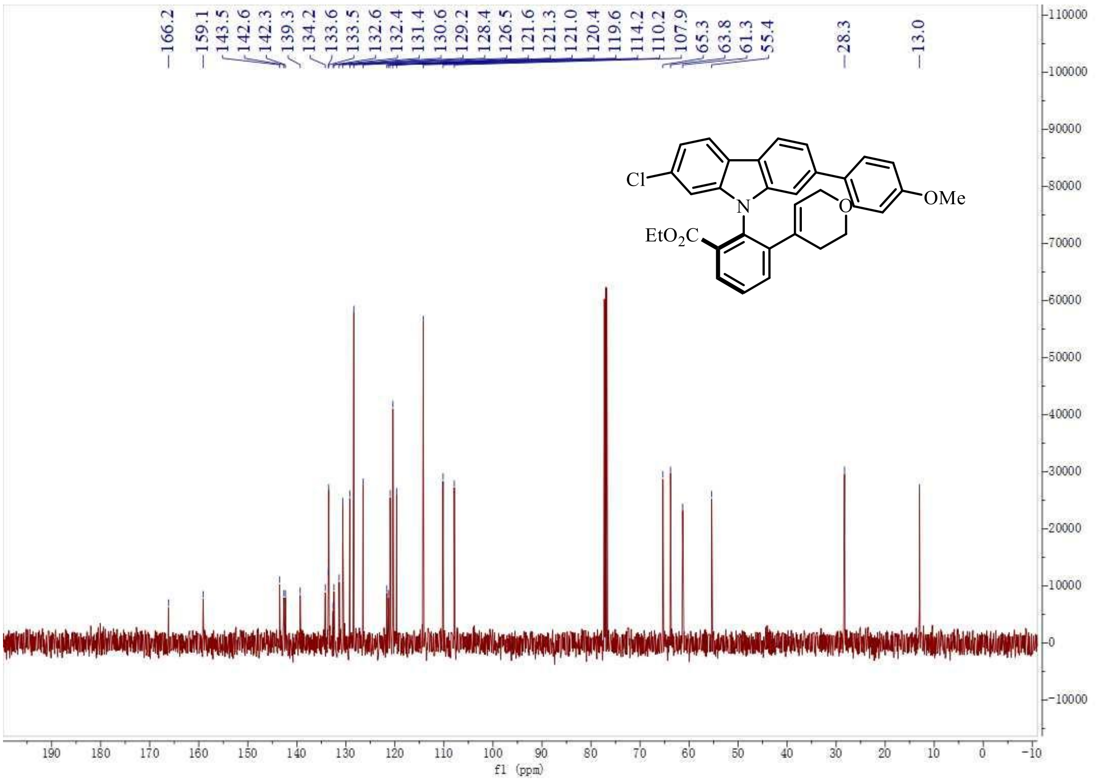

Ethyl (*S*)-3-chloro-2-(2-chloro-7-(4-methoxyphenyl)-9*H*-carbazol-9-yl)benzoate (27)

^{1}H NMR (500 MHz, Chloroform-*d*)

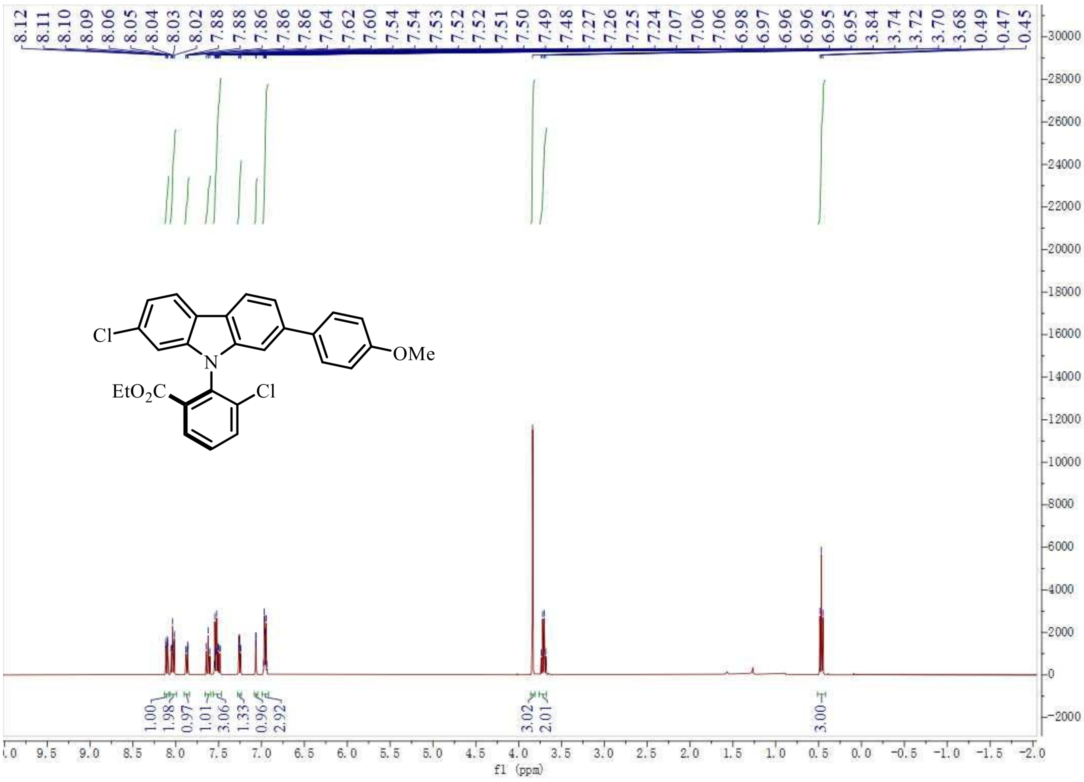

^{13}C NMR (126 MHz, Chloroform-*d*)

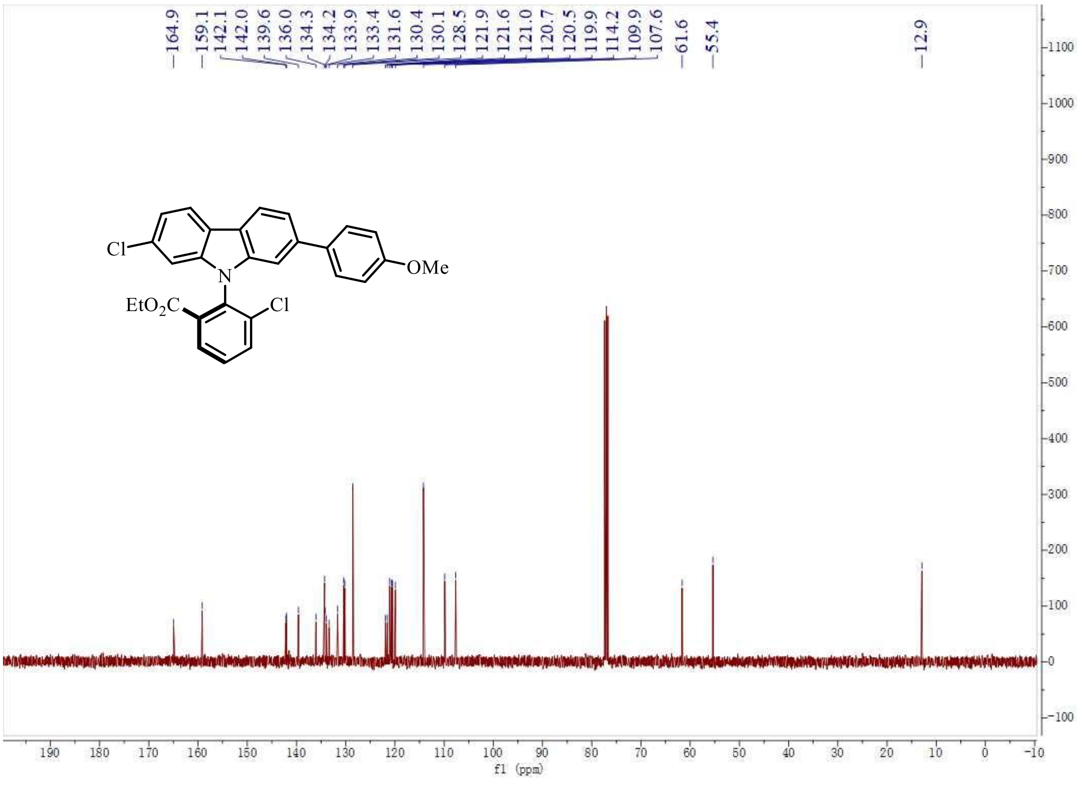

(*S*)-2-(2-(3-(((*tert*-Butyldimethylsilyl)oxy)methyl)phenyl)-7-chloro-9*H*-carbazol-9-yl)-3-chlorobenzoate (28) ^{1}H NMR (500 MHz, Chloroform-*d*)

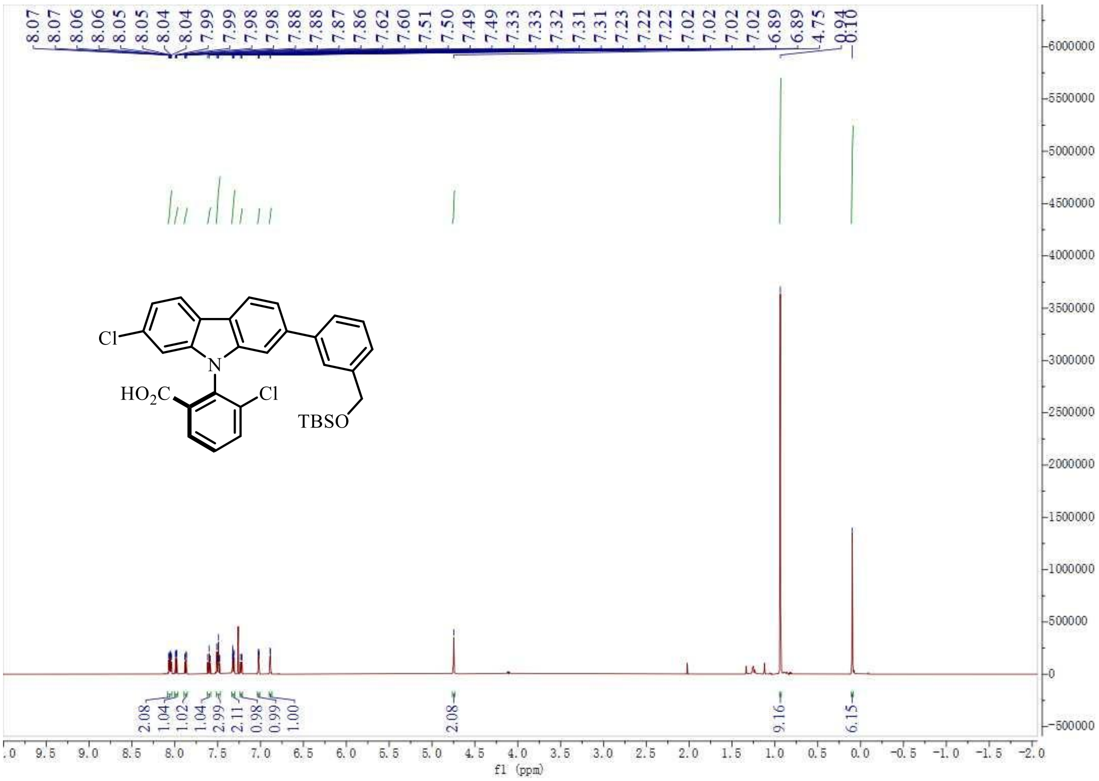

^{13}C NMR (126 MHz, Chloroform-*d*)

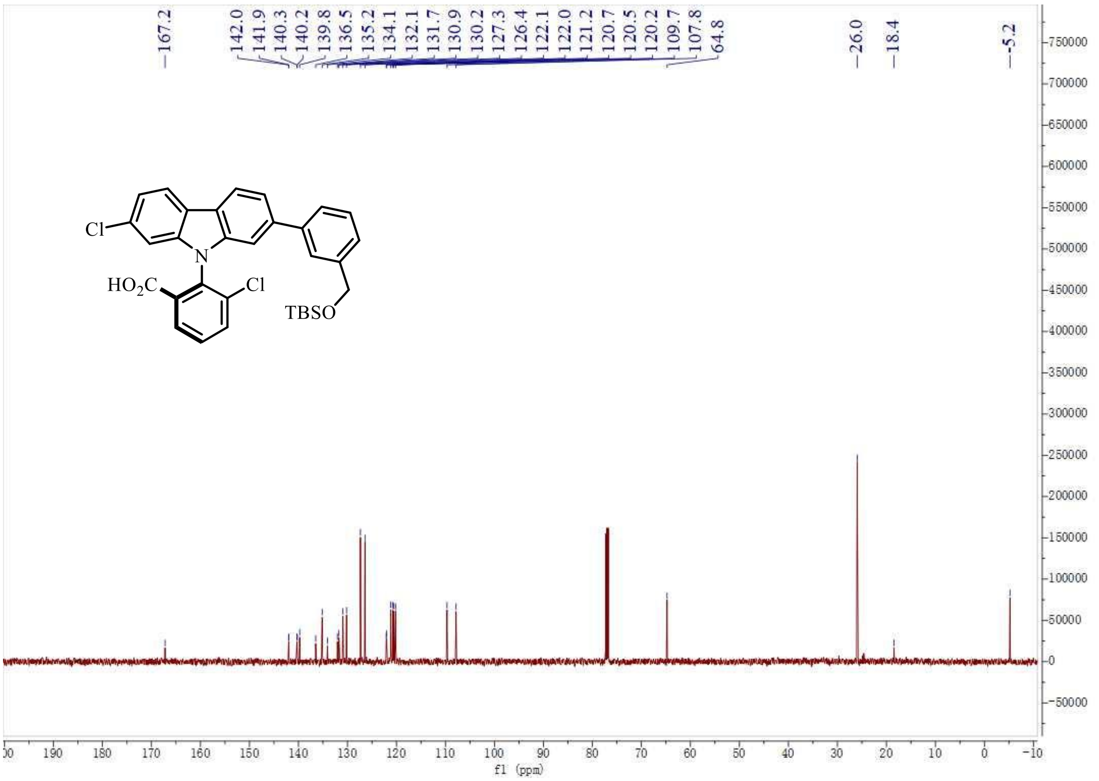

(*S*)-3-Chloro-2-(2-chloro-7-(3-(hydroxymethyl)phenyl)-9*H*-carbazol-9-yl)benzoate (SI-**28**)

^{1}H NMR (500 MHz, Acetone-d_6)

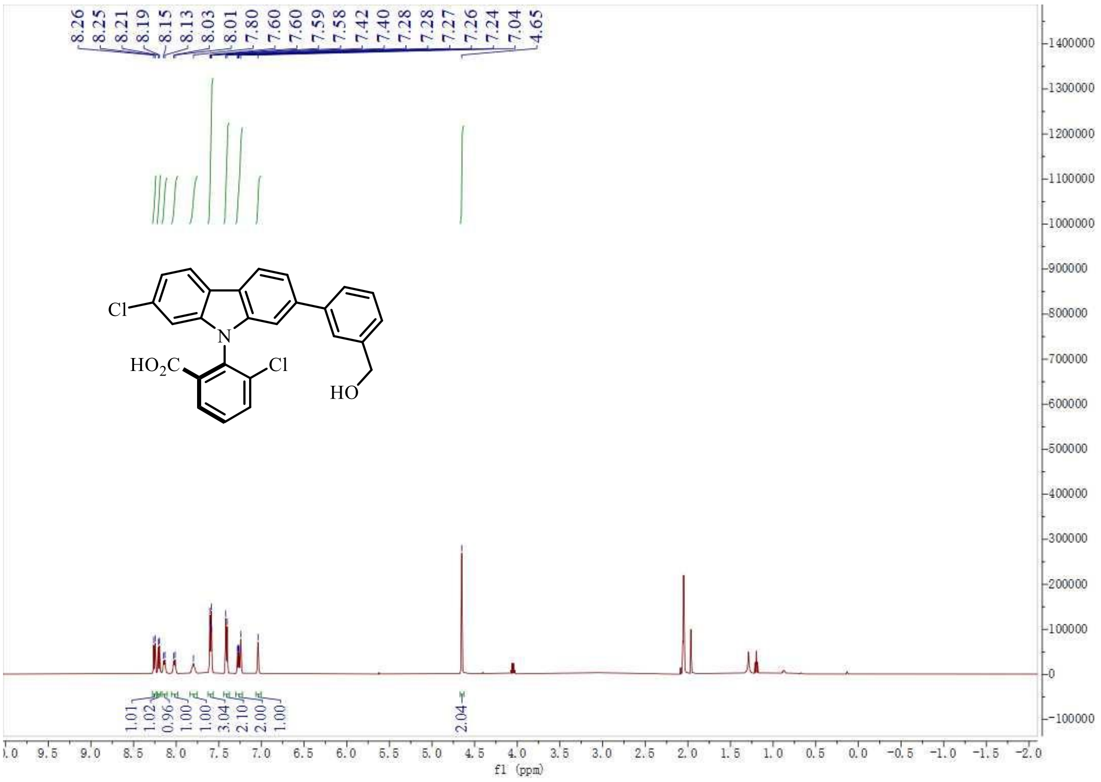

^{13}C NMR (126 MHz, Acetone-d_6)

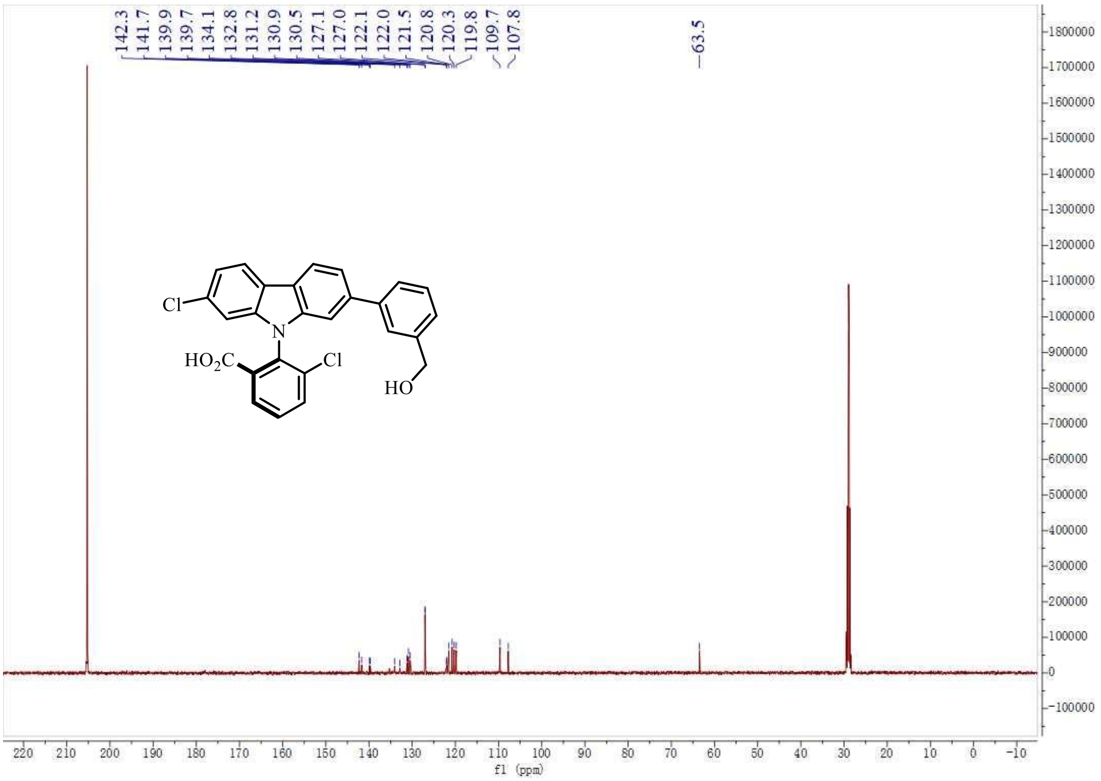

Ethyl (*S*)-2-(2-(3-(((*tert*-butoxycarbonyl)amino)methyl)phenyl)-7-chloro-9*H*-carbazol-9-yl)-3-chlorobenzoate (29) ^{1}H NMR (500 MHz, Chloroform-*d*)

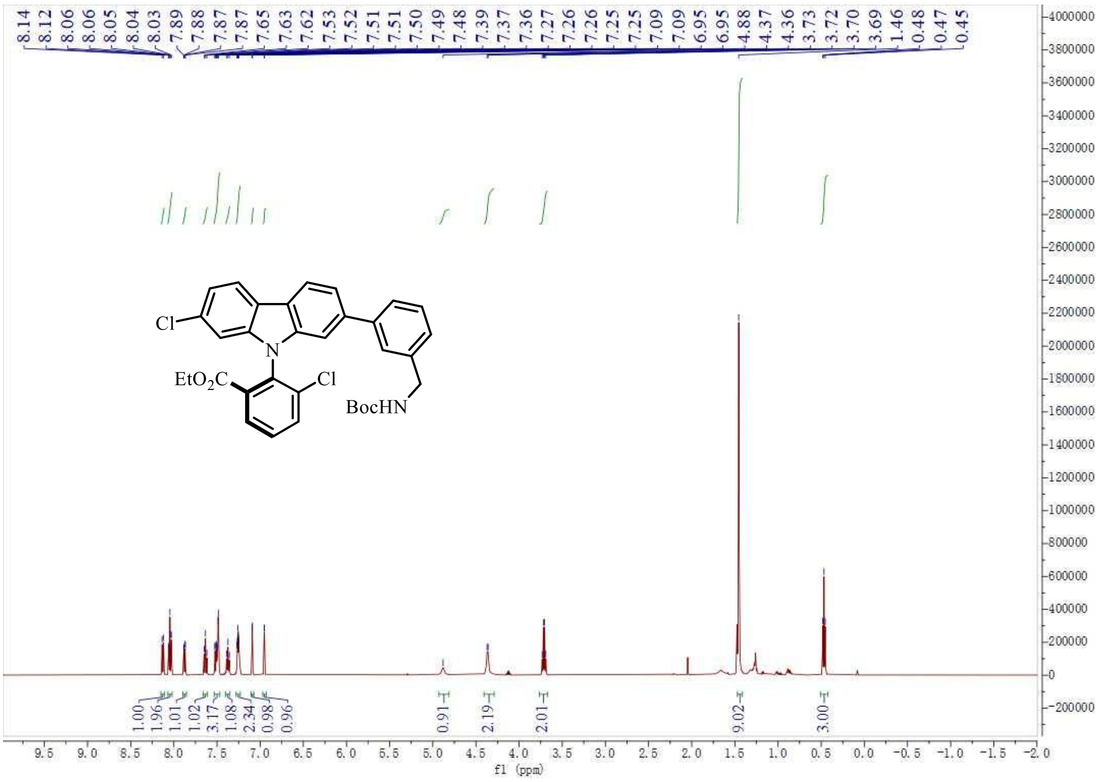

^{13}C NMR (126 MHz, Chloroform-*d*)

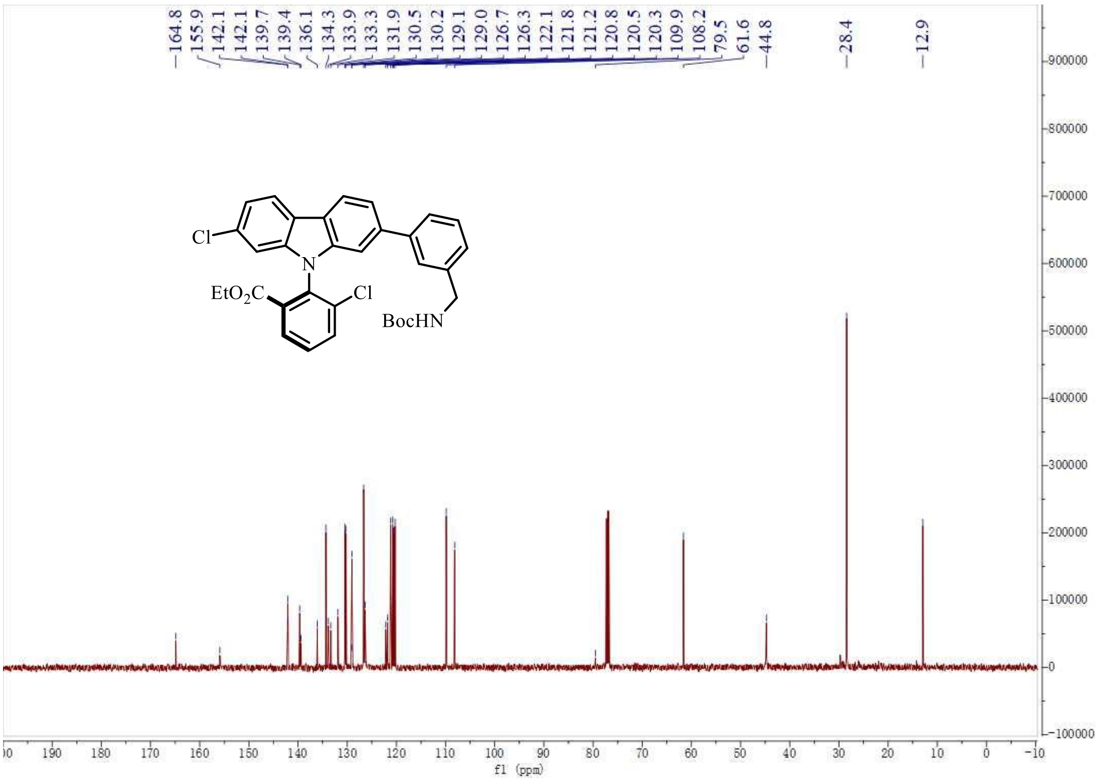

Ethyl (*R*)-2-(2-chloro-7-(4-methoxyphenyl)-9*H*-carbazol-9-yl)benzoate (30)

^{1}H NMR (500 MHz, Chloroform-*d*)

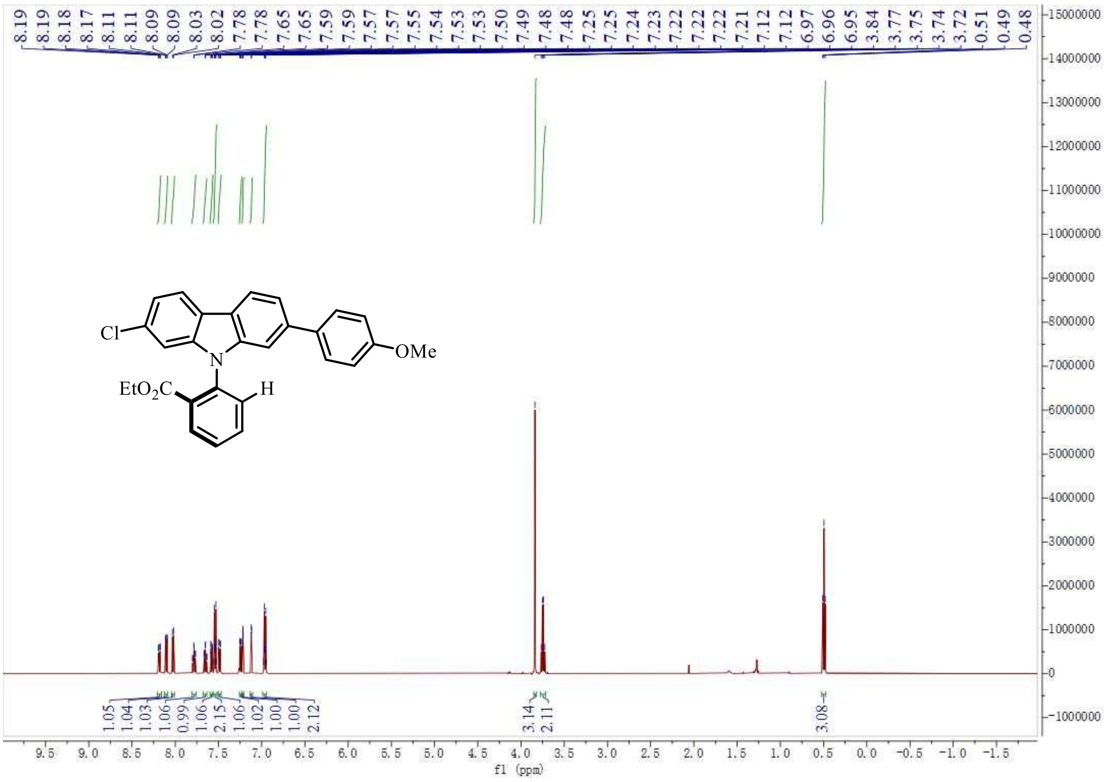

^{13}C NMR (126 MHz, Chloroform-*d*)

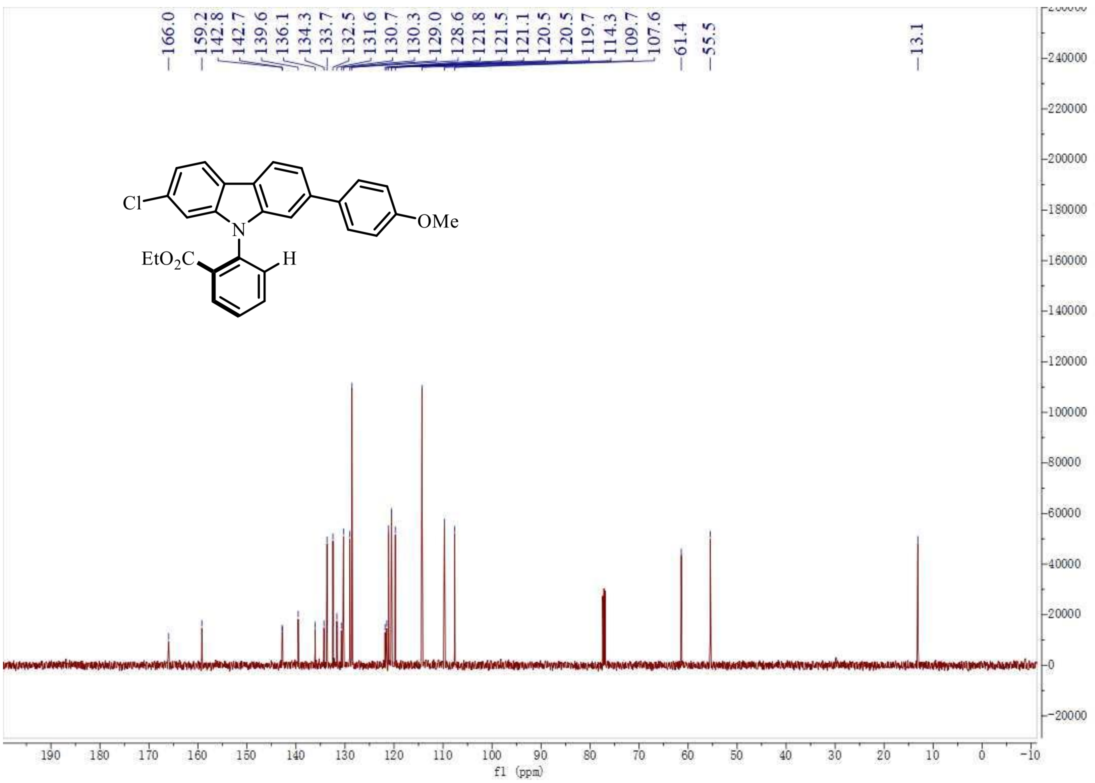

Ethyl (*R*)-2-(2-chloro-3,6-dimethoxy-7-(4-methoxyphenyl)-9*H*-carbazol-9-yl)-3-methylbenzoate (31) ^{1}H NMR (500 MHz, Chloroform-*d*)

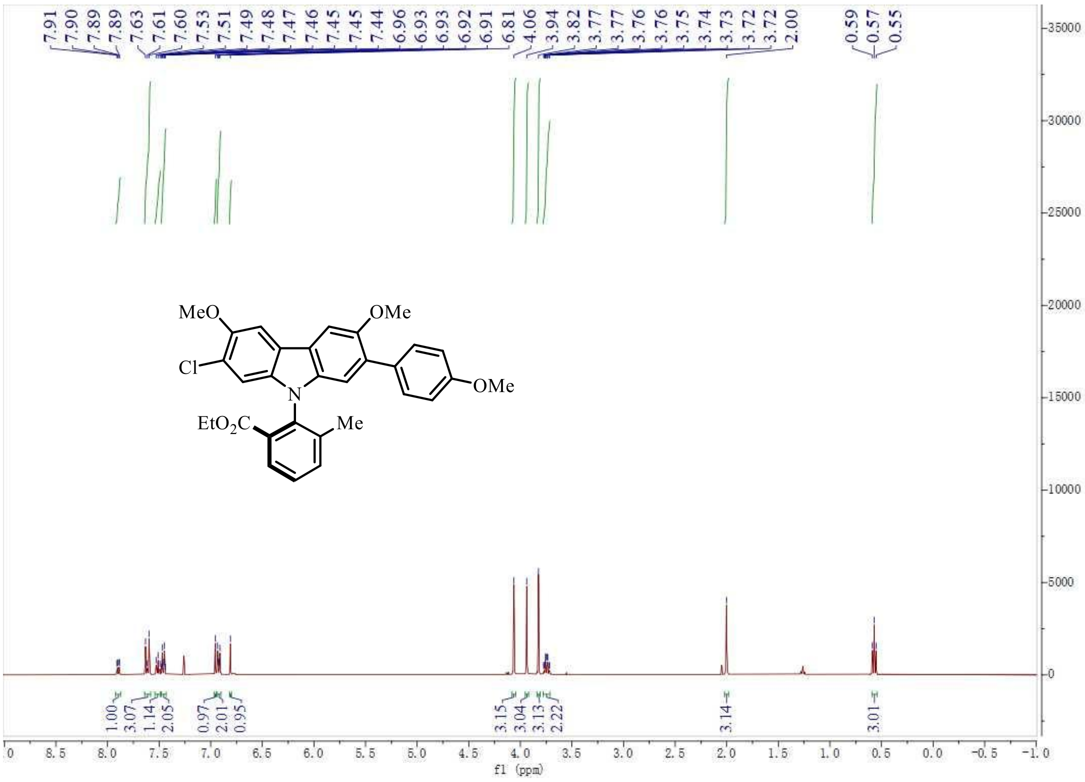

^{13}C NMR (126 MHz, Chloroform-*d*)

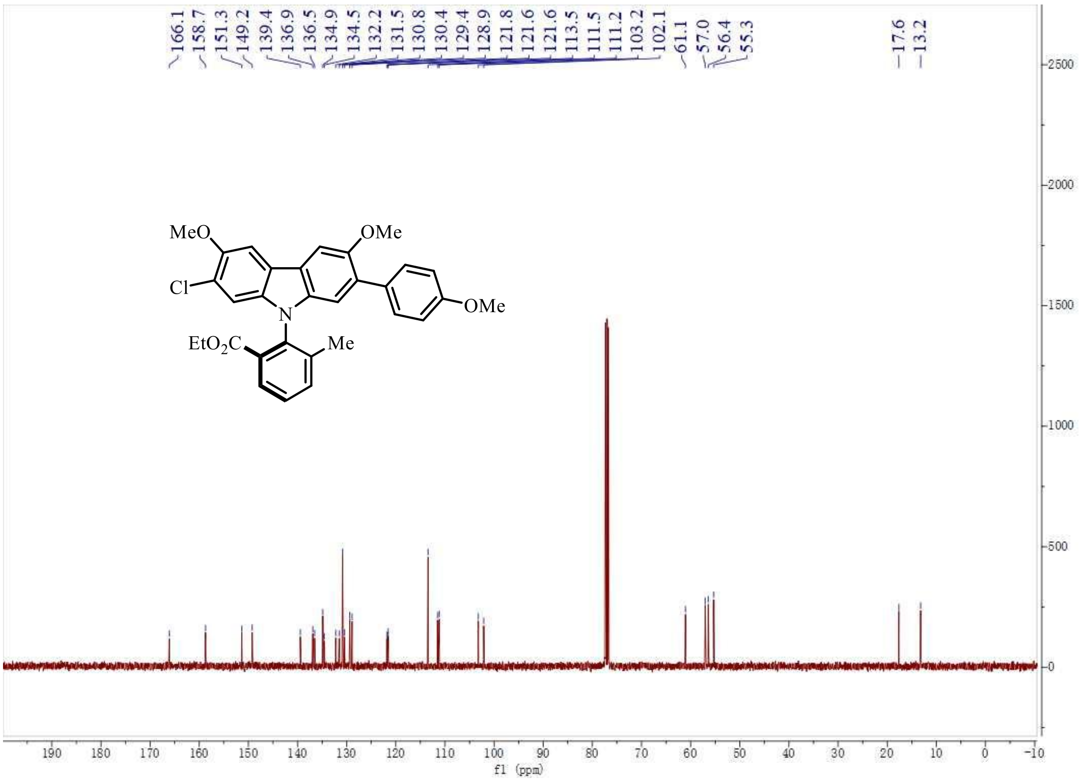

Ethyl (*S*)-3-chloro-2-(2-chloro-8-(4-methoxyphenyl)-10*H*-phenoxazin-10-yl)benzoate (32)

^{1}H NMR (500 MHz, Chloroform-*d*)

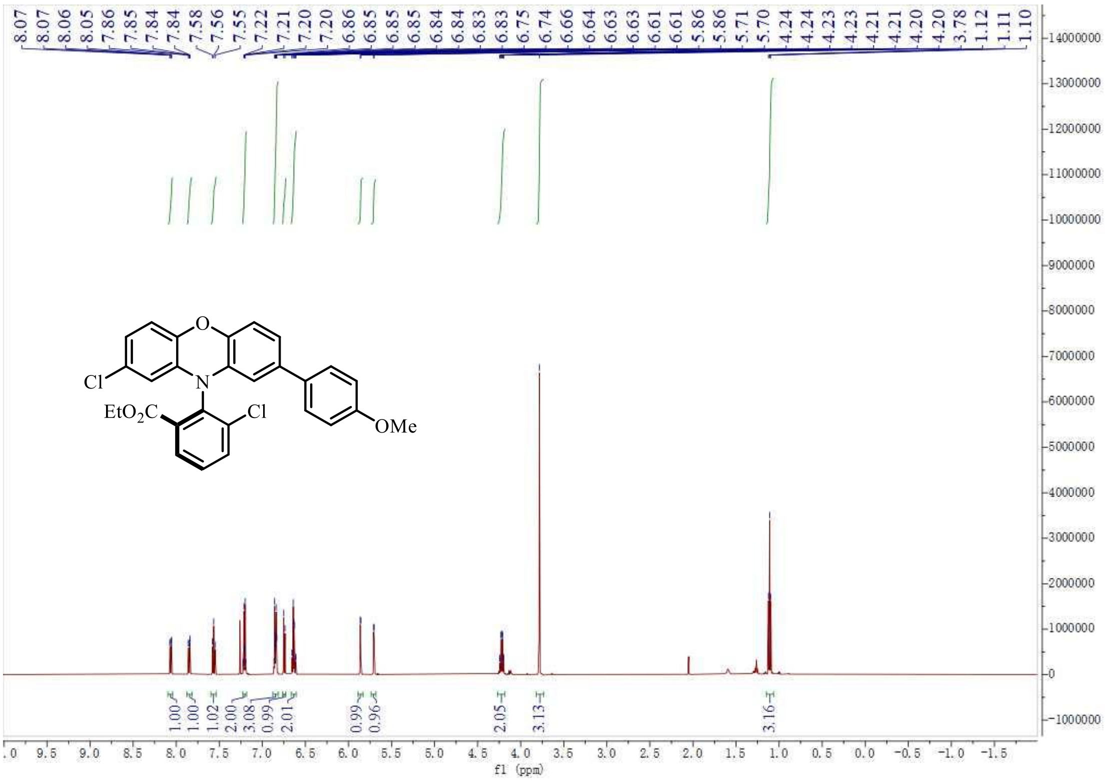

^{13}C NMR (101 MHz, Chloroform-*d*)

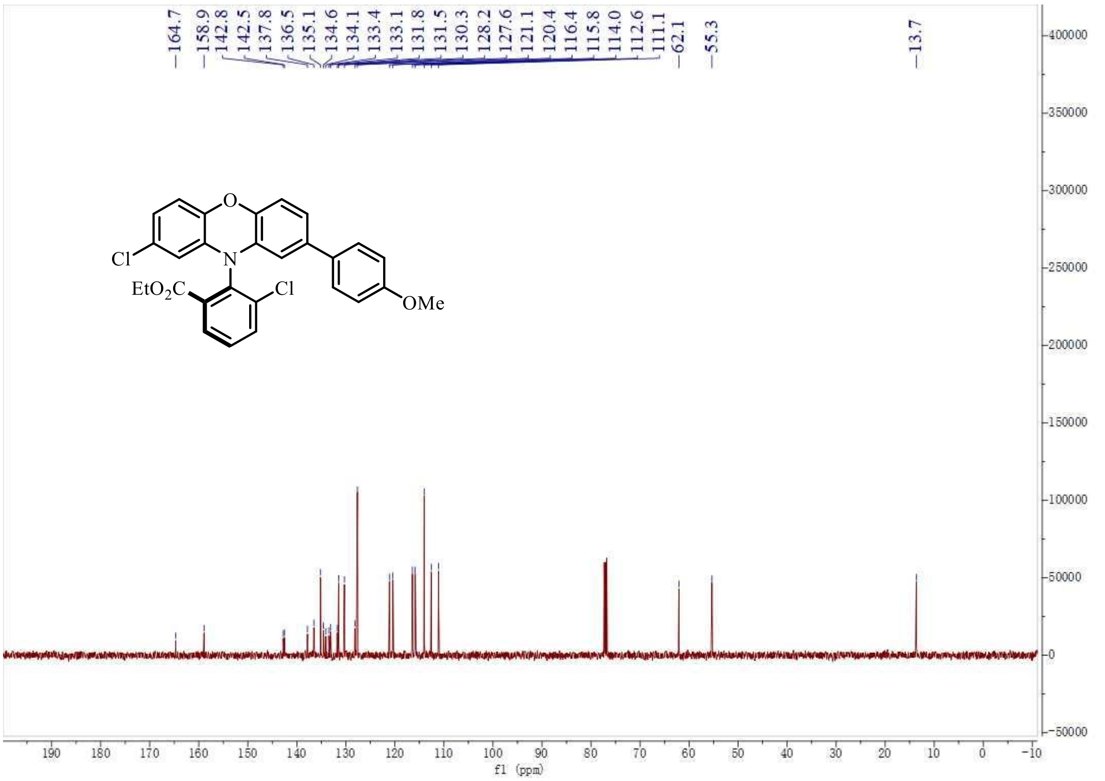

Ethyl (*S*)-3-chloro-2-(3-chloro-6-(4-methoxyphenyl)-9,9-dimethylacridin-10(9*H*)-yl)benzoate

(33) ^{1}H NMR (500 MHz, Chloroform-*d*)

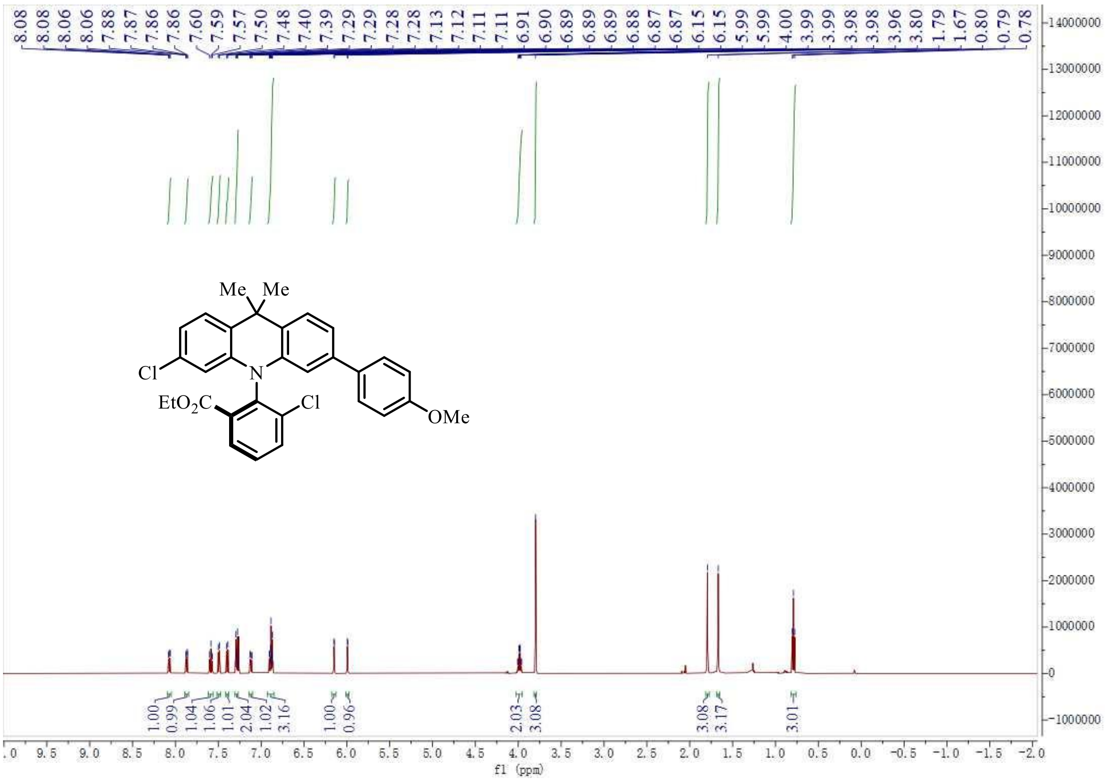

^{13}C NMR (126 MHz, Chloroform-*d*)

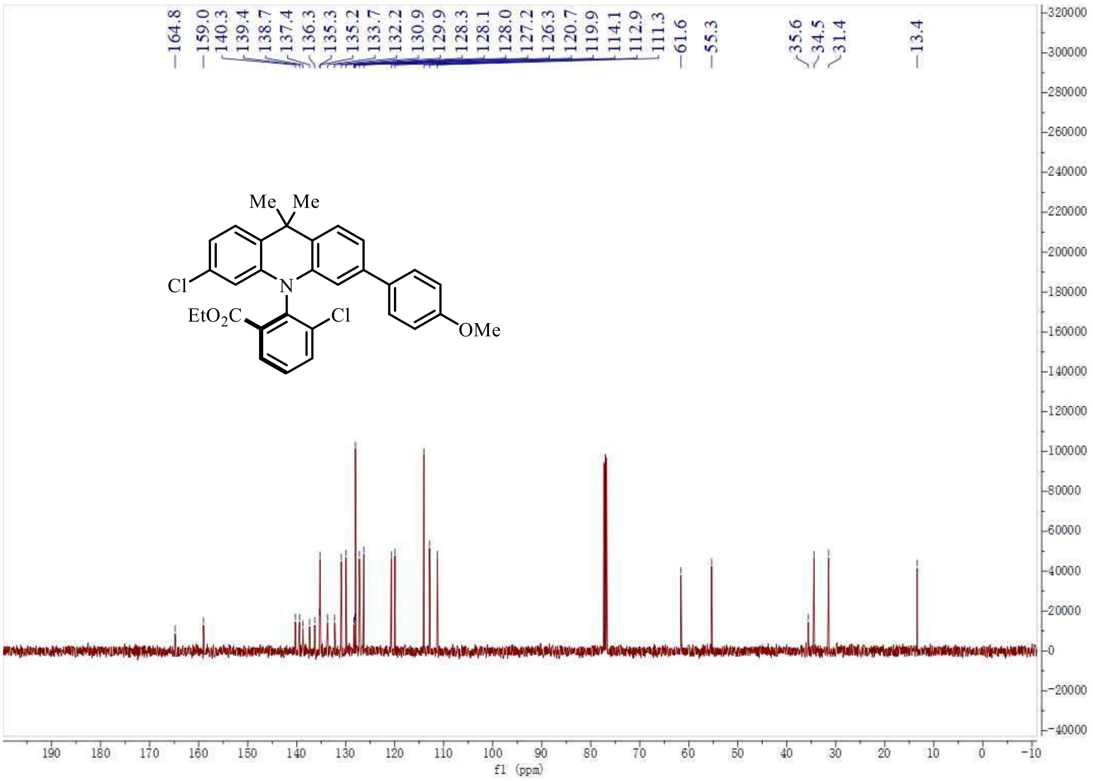

Ethyl (*R*)-1-(2-chloro-7-(4-(ethoxycarbonyl)phenyl)-9*H*-carbazol-9-yl)-5-ethyl-1*H*-pyrrole-2-carboxylate (35) ^{1}H NMR (500 MHz, Chloroform-*d*)

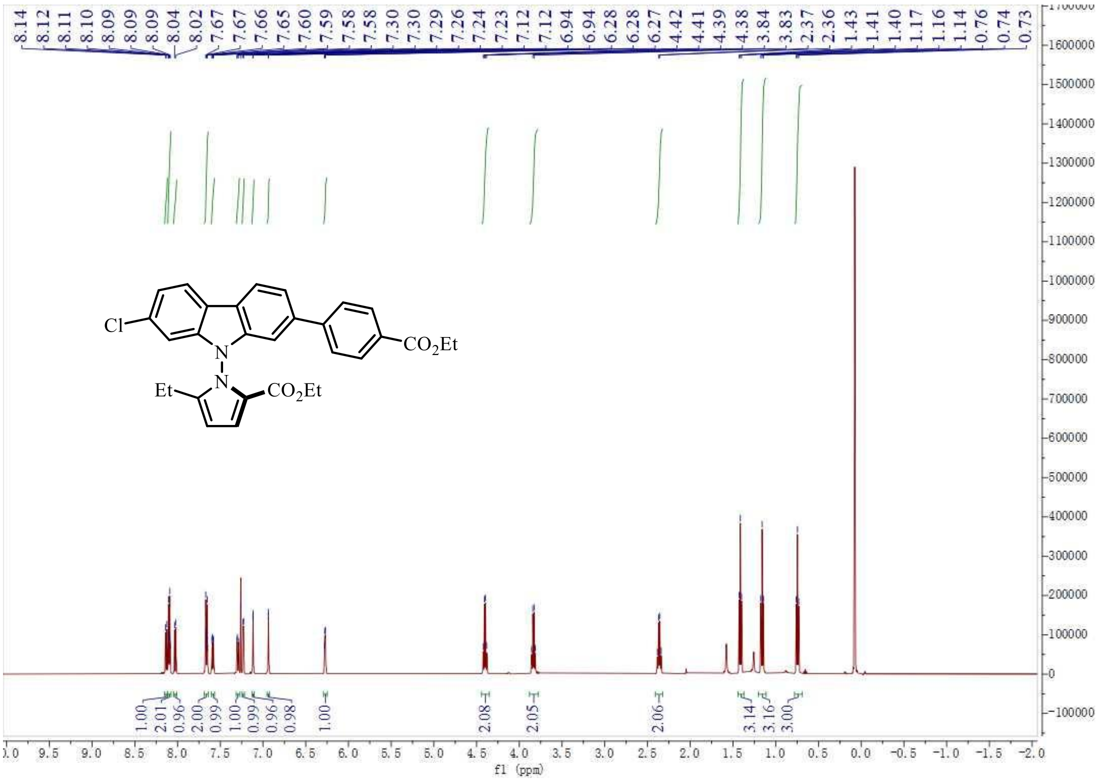

^{13}C NMR (126 MHz, Chloroform-*d*)

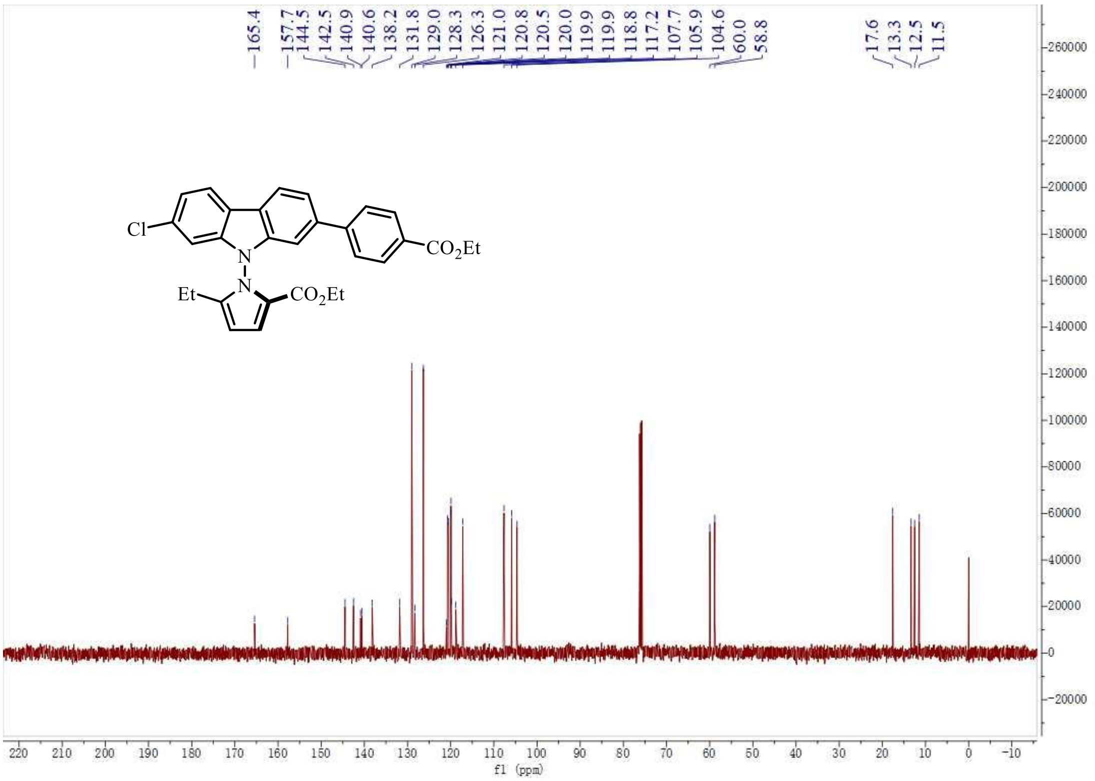

Ethyl (*R*)-1-(2-chloro-7-(4-cyanophenyl)-9*H*-carbazol-9-yl)-5-ethyl-1*H*-pyrrole-2-carboxylate

(36) ^{1}H NMR (500 MHz, Chloroform-*d*)

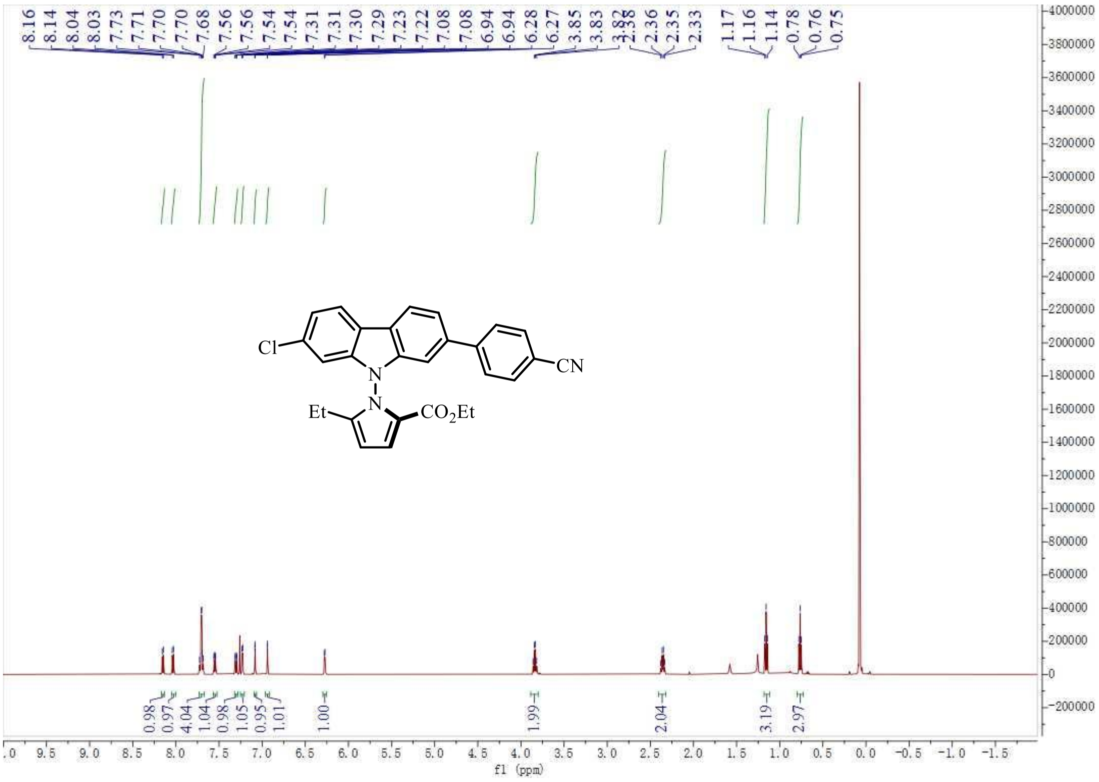

^{13}C NMR (126 MHz, Chloroform-*d*)

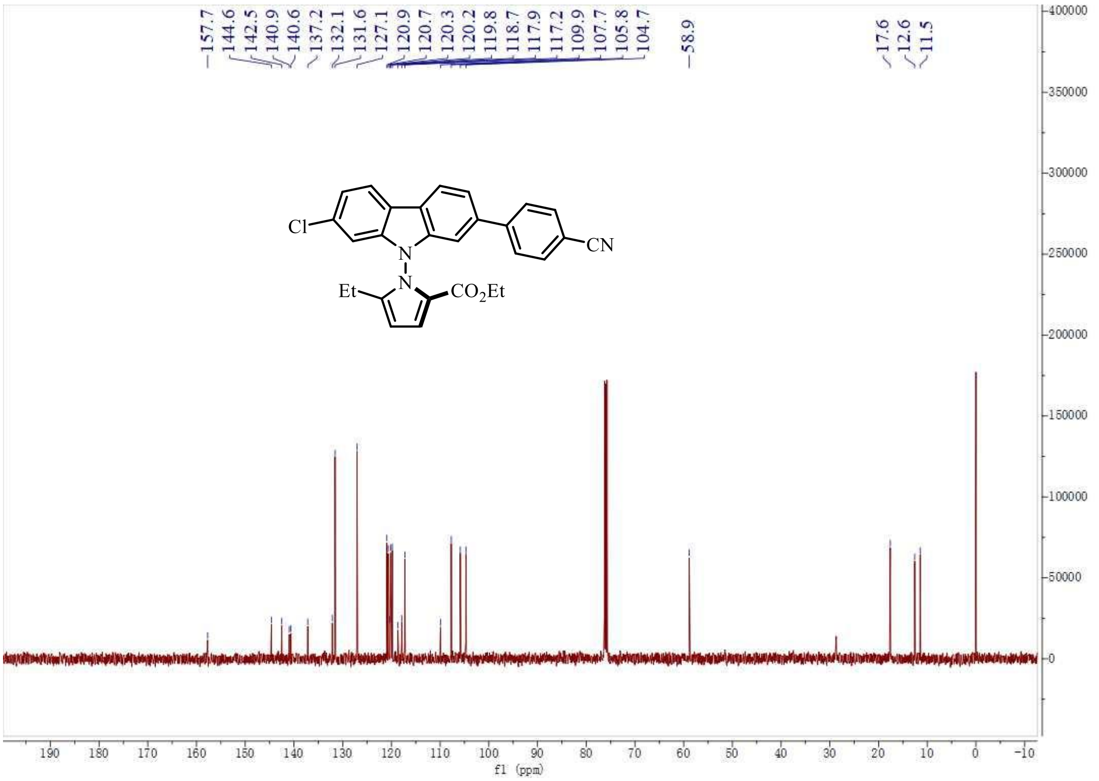

Ethyl (*R*)-1-(2-chloro-7-(4-nitrophenyl)-9*H*-carbazol-9-yl)-5-ethyl-1*H*-pyrrole-2-carboxylate

(37) ^{1}H NMR (500 MHz, Chloroform-*d*)

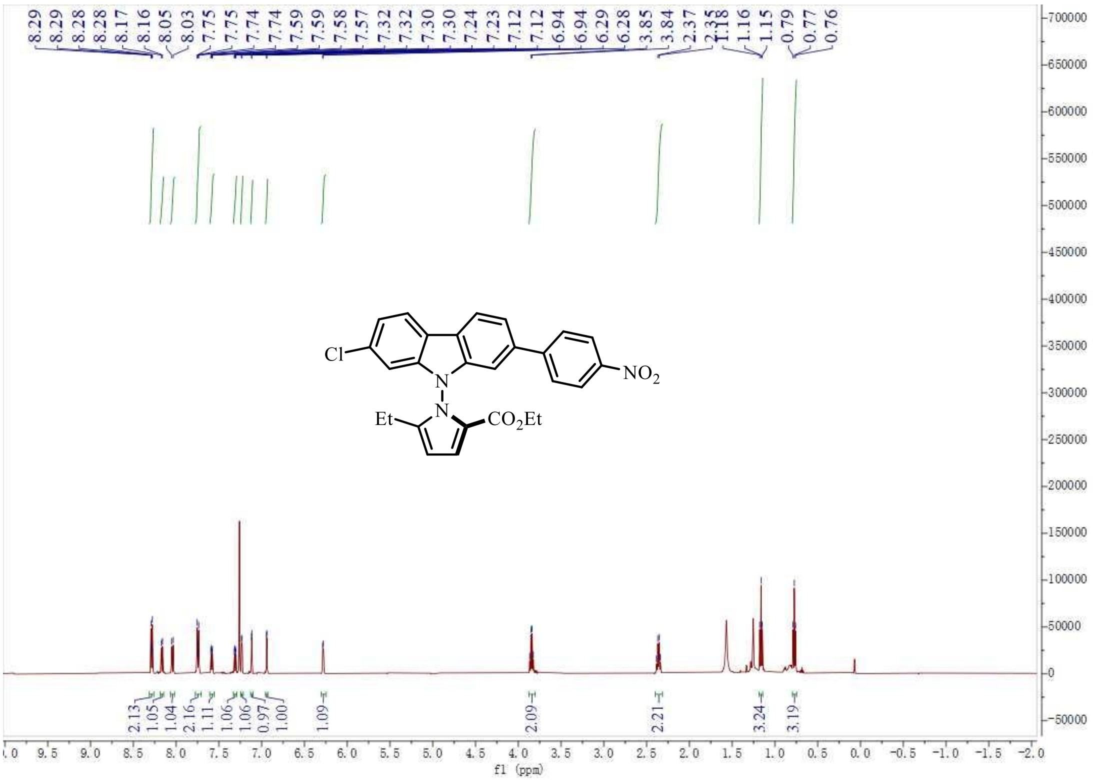

^{13}C NMR (126 MHz, Chloroform-*d*)

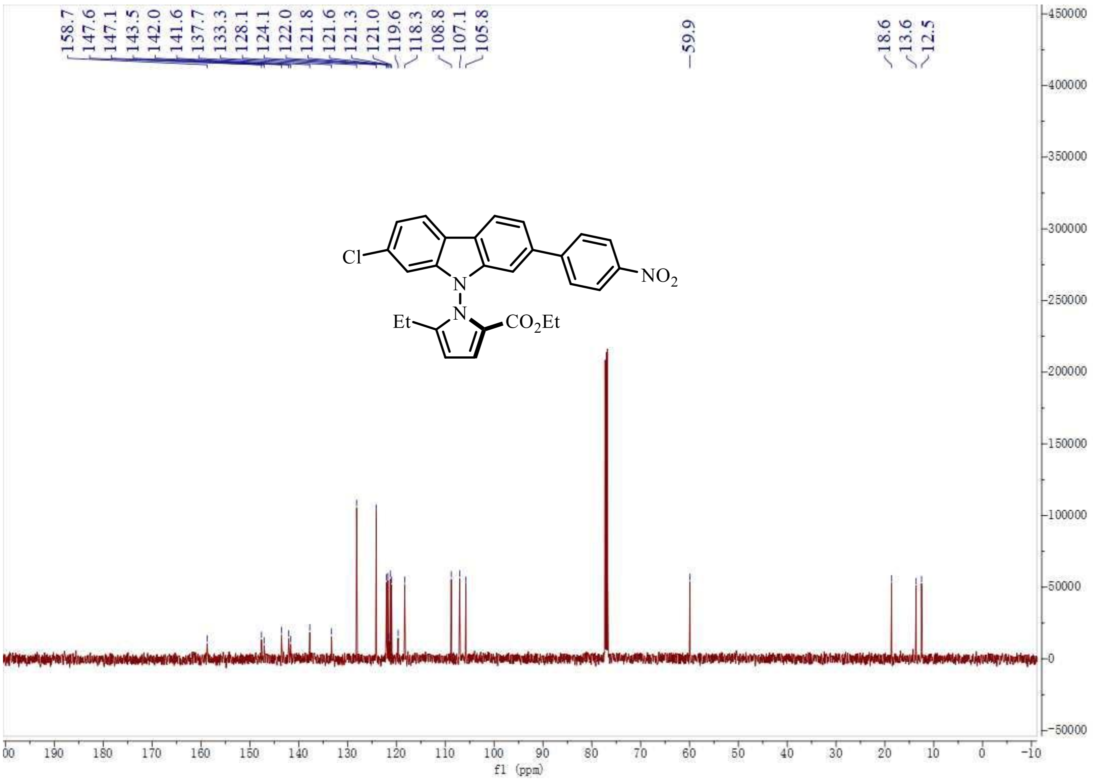

Ethyl (*R*)-1-(2-chloro-7-(4-methoxyphenyl)-9*H*-carbazol-9-yl)-5-ethyl-1*H*-pyrrole-2-carboxylate (38) ^{1}H NMR (500 MHz, Chloroform-*d*)

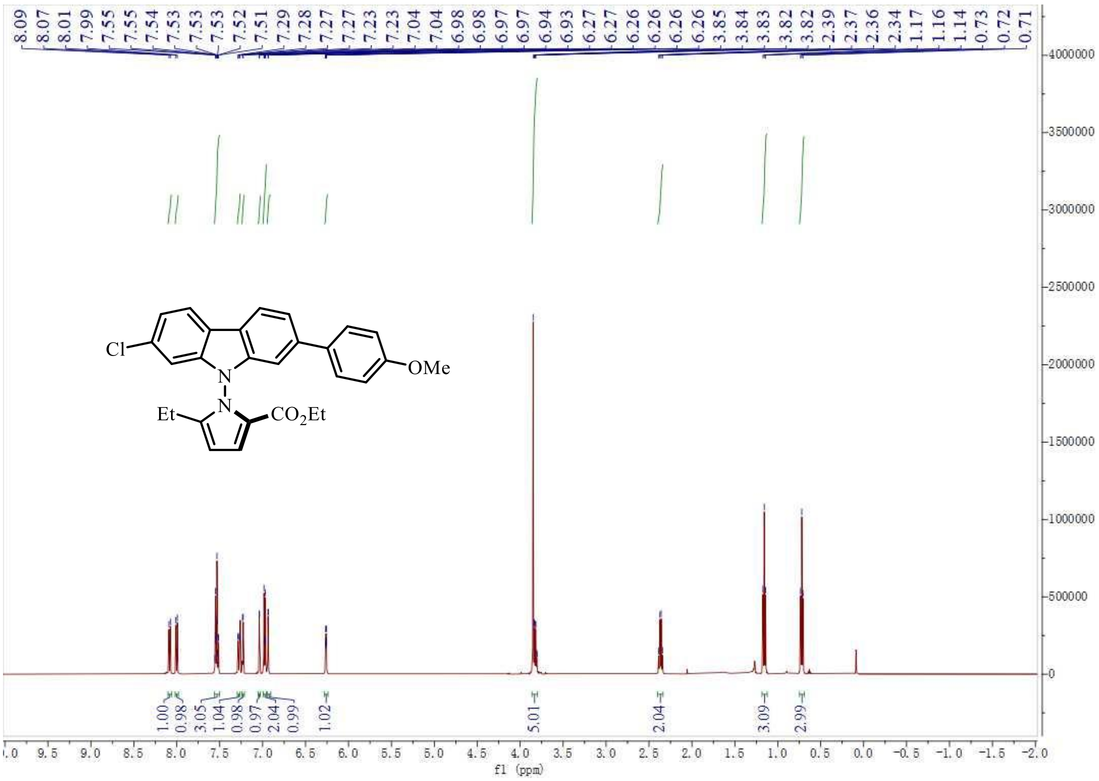

^{13}C NMR (126 MHz, Chloroform-*d*)

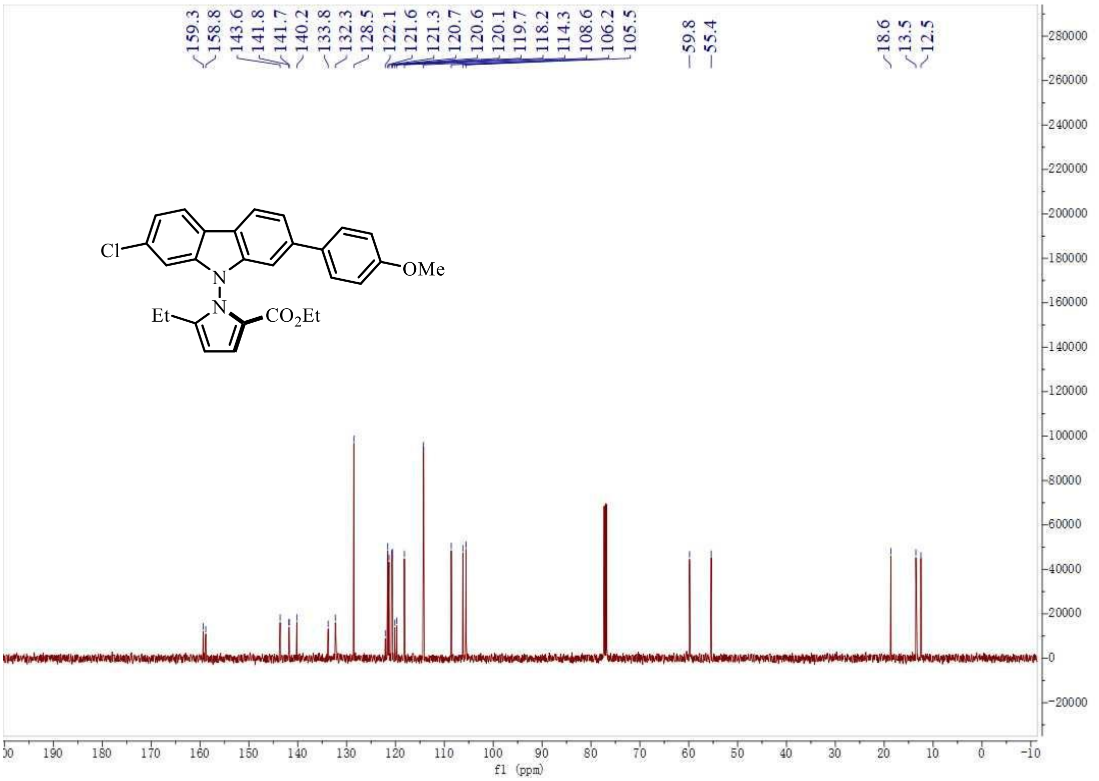

Ethyl (*R*)-1-(2-chloro-7-(3-nitrophenyl)-9*H*-carbazol-9-yl)-5-ethyl-1*H*-pyrrole-2-carboxylate

(39) ^{1}H NMR (500 MHz, Chloroform-*d*)

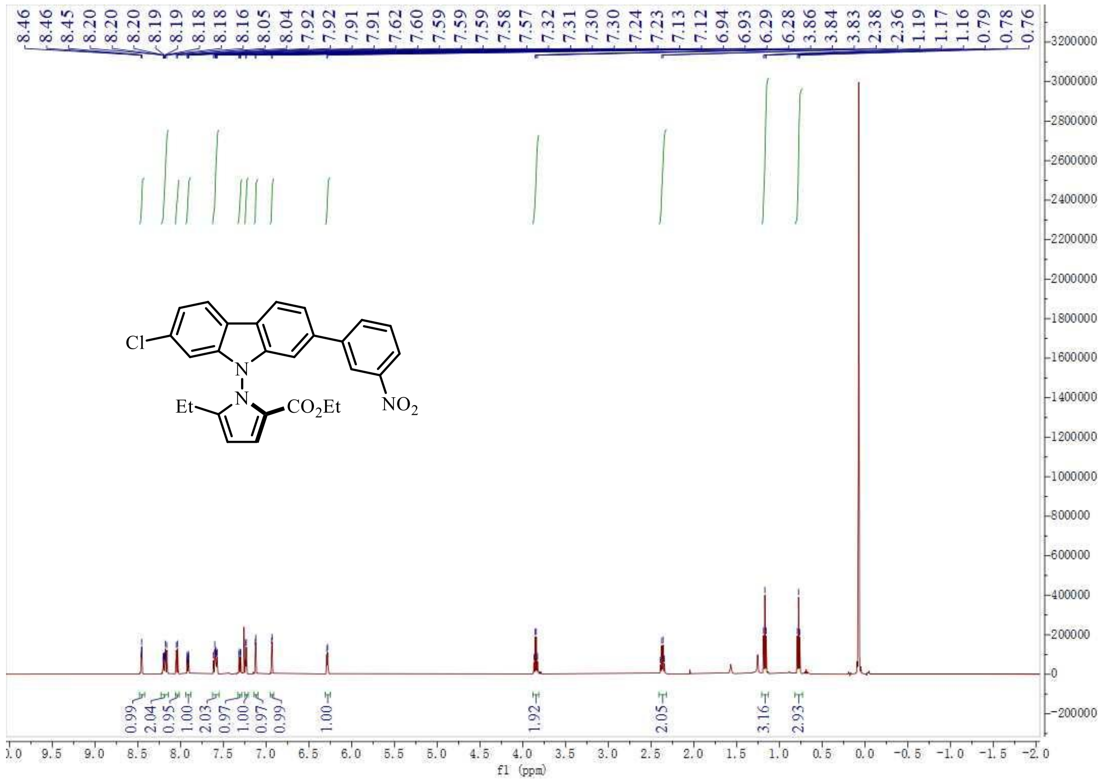

^{13}C NMR (126 MHz, Chloroform-*d*)

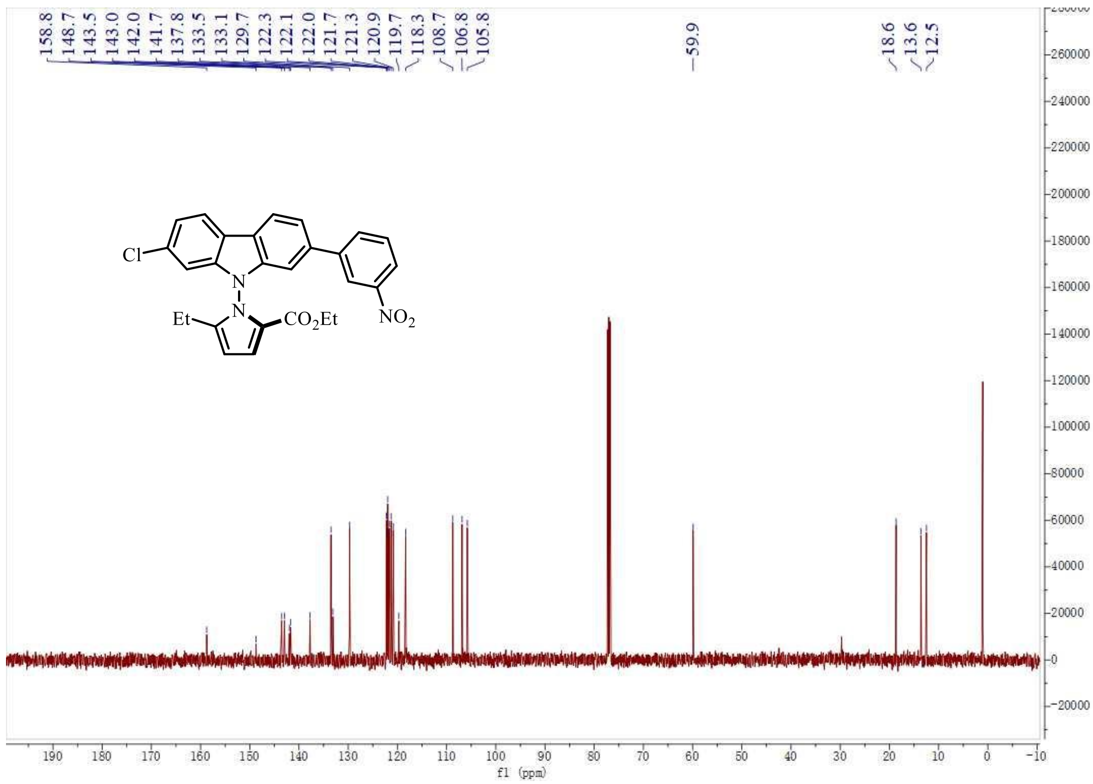

Ethyl (*R*)-1-(2-chloro-7-(3-methoxyphenyl)-9*H*-carbazol-9-yl)-5-ethyl-1*H*-pyrrole-2-carboxylate (40) ^{1}H NMR (500 MHz, Chloroform-*d*)

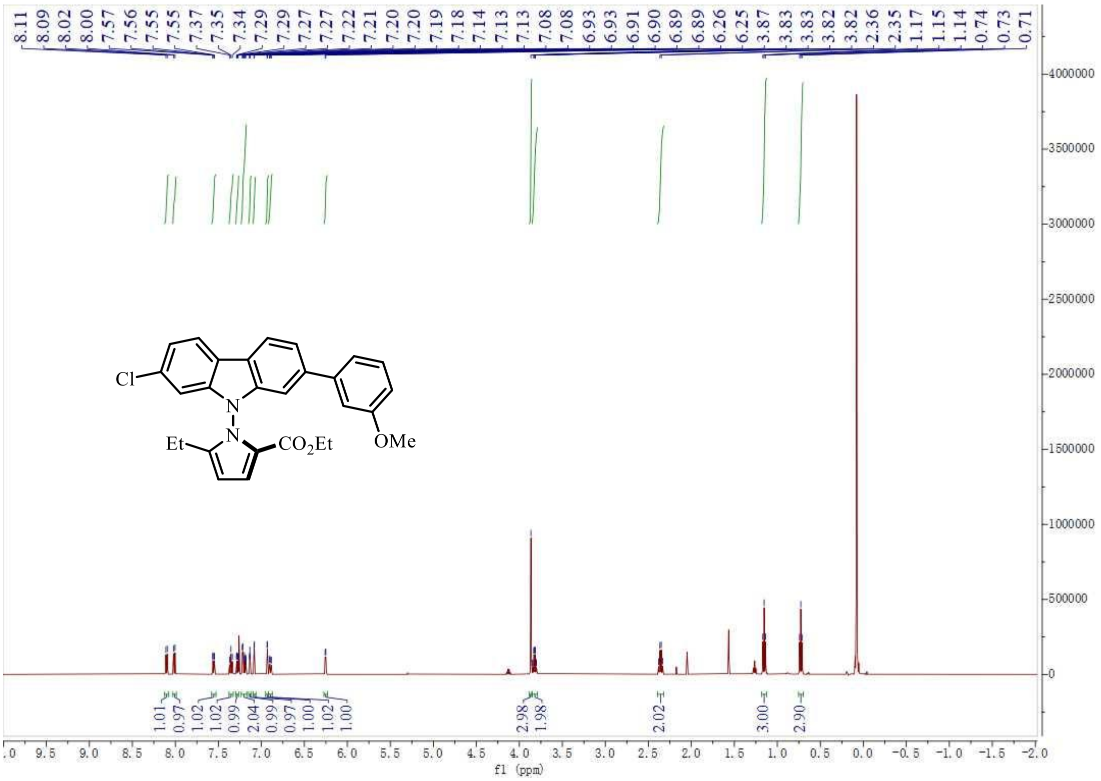

^{13}C NMR (126 MHz, Chloroform-*d*)

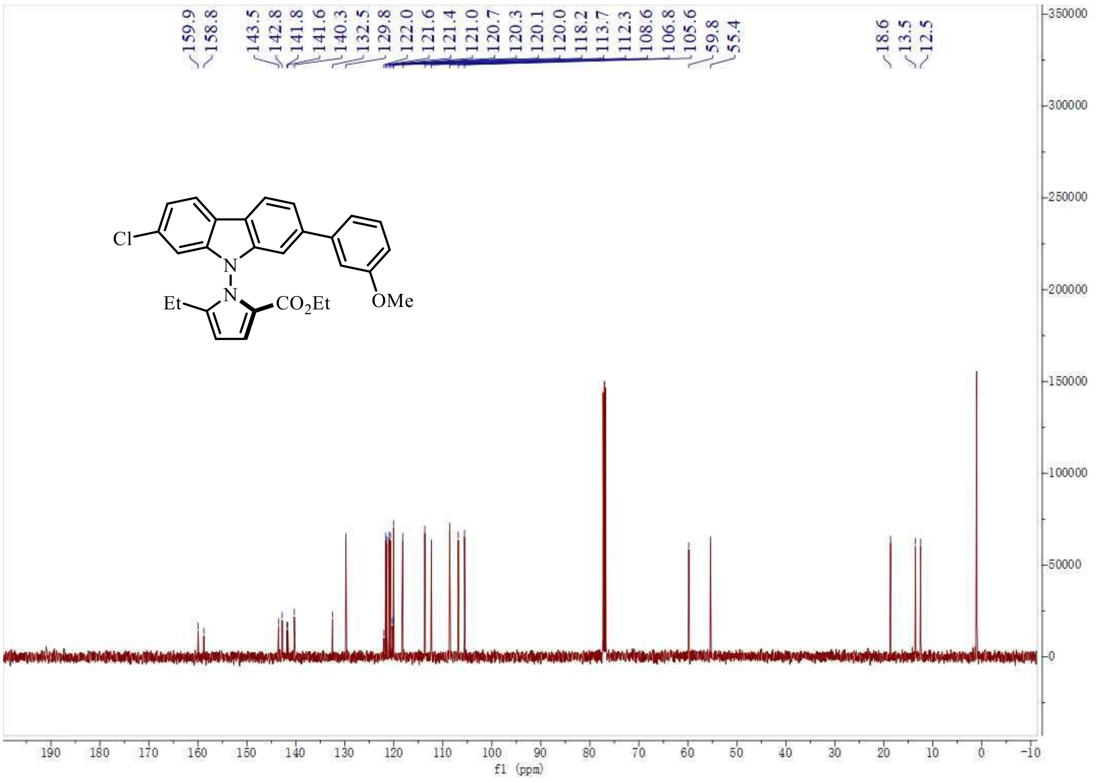

Ethyl (*R*)-1-(2-(3-(benzyloxy)phenyl)-7-chloro-9*H*-carbazol-9-yl)-5-ethyl-1*H*-pyrrole-2-carboxylate (41) ^{1}H NMR (500 MHz, Chloroform-*d*)

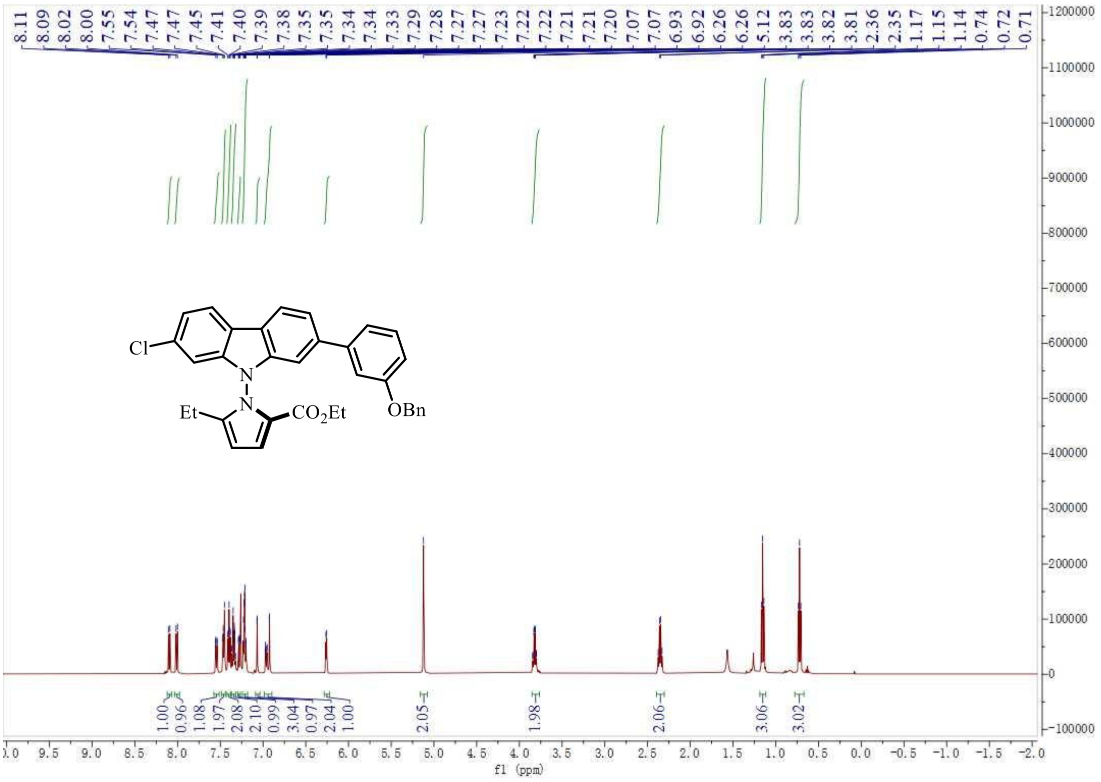

^{13}C NMR (126 MHz, Chloroform-*d*)

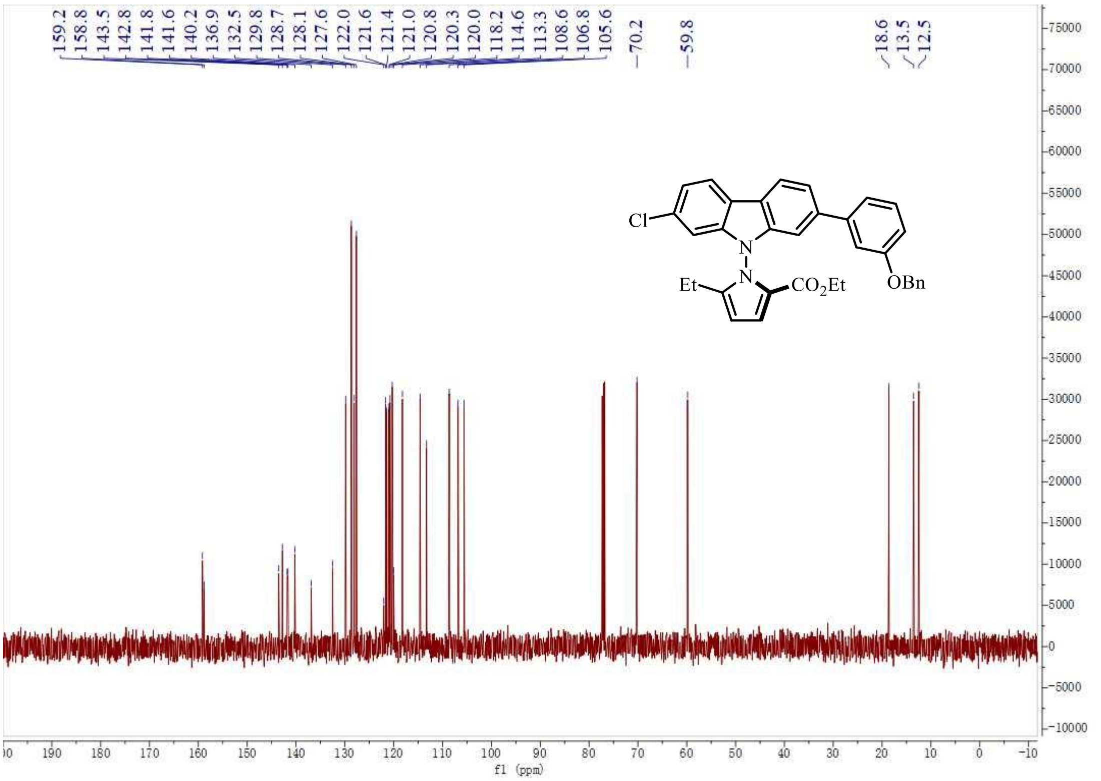

Ethyl (*R*)-1-(2-chloro-7-(6-methoxypyridin-3-yl)-9*H*-carbazol-9-yl)-5-ethyl-1*H*-pyrrole-2-carboxylate (42) ^{1}H NMR (500 MHz, Chloroform-*d*)

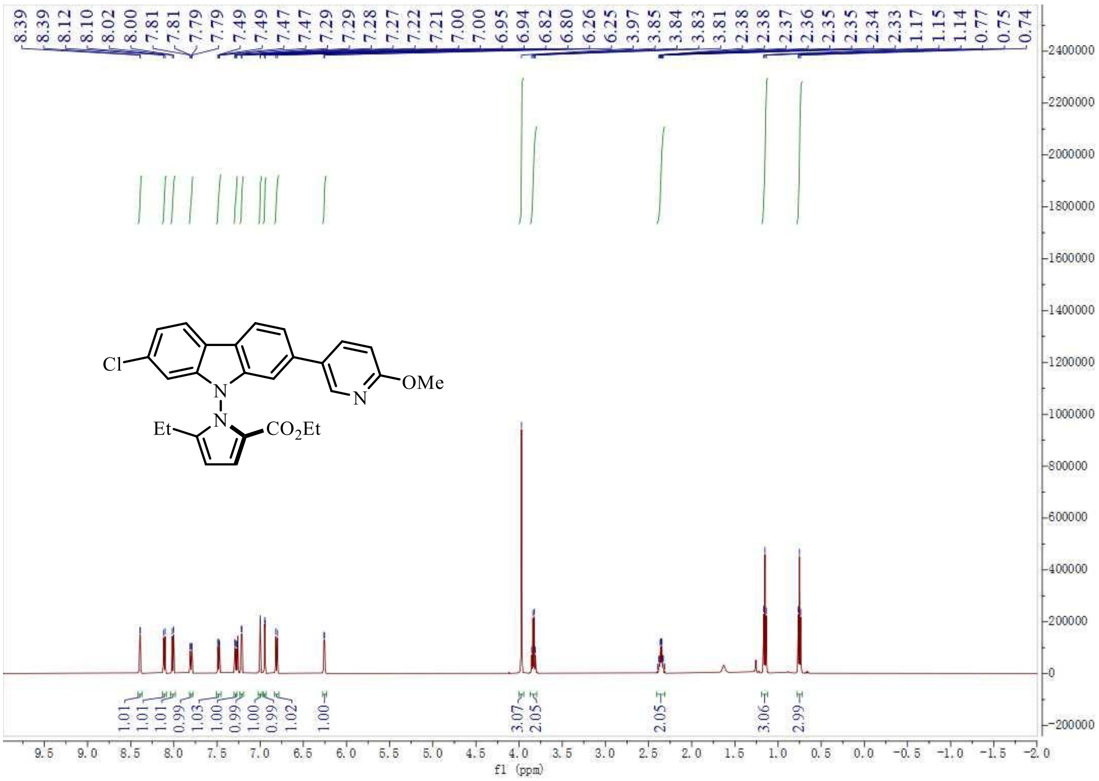

^{13}C NMR (126 MHz, Chloroform-*d*)

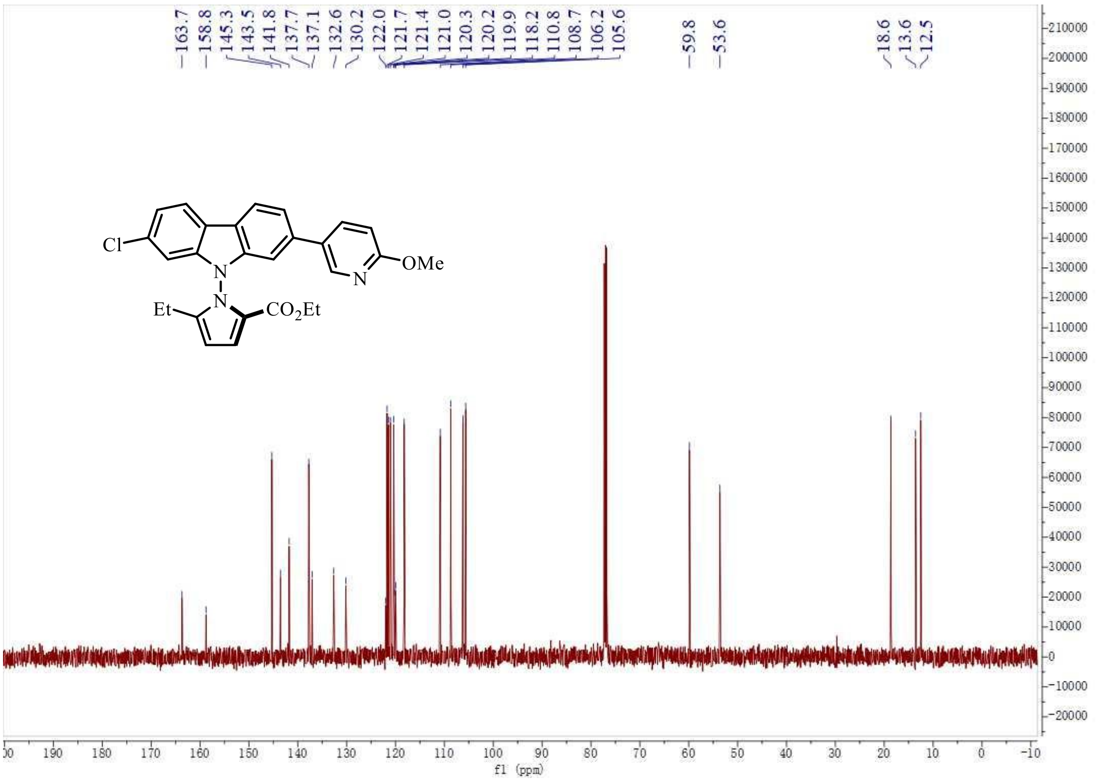

Ethyl (*R*)-1-(2-chloro-7-(furan-3-yl)-9*H*-carbazol-9-yl)-5-ethyl-1*H*-pyrrole-2-carboxylate (43)

^{1}H NMR (500 MHz, Chloroform-*d*)

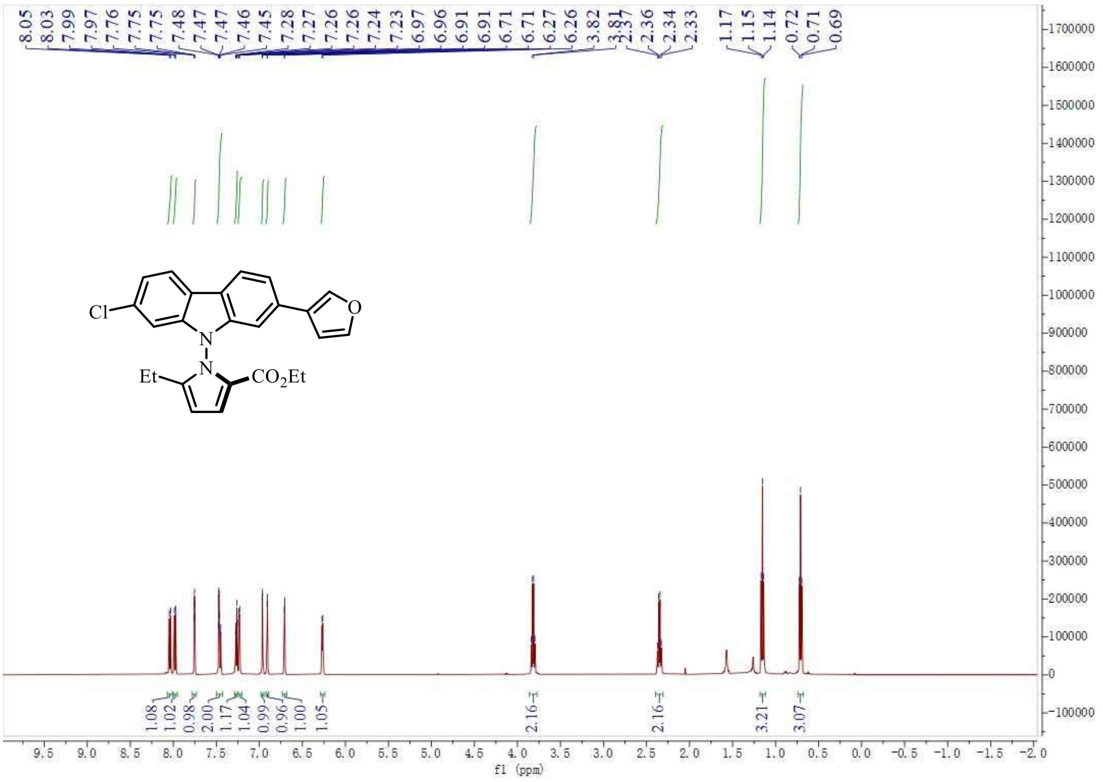

^{13}C NMR (126 MHz, Chloroform-*d*)

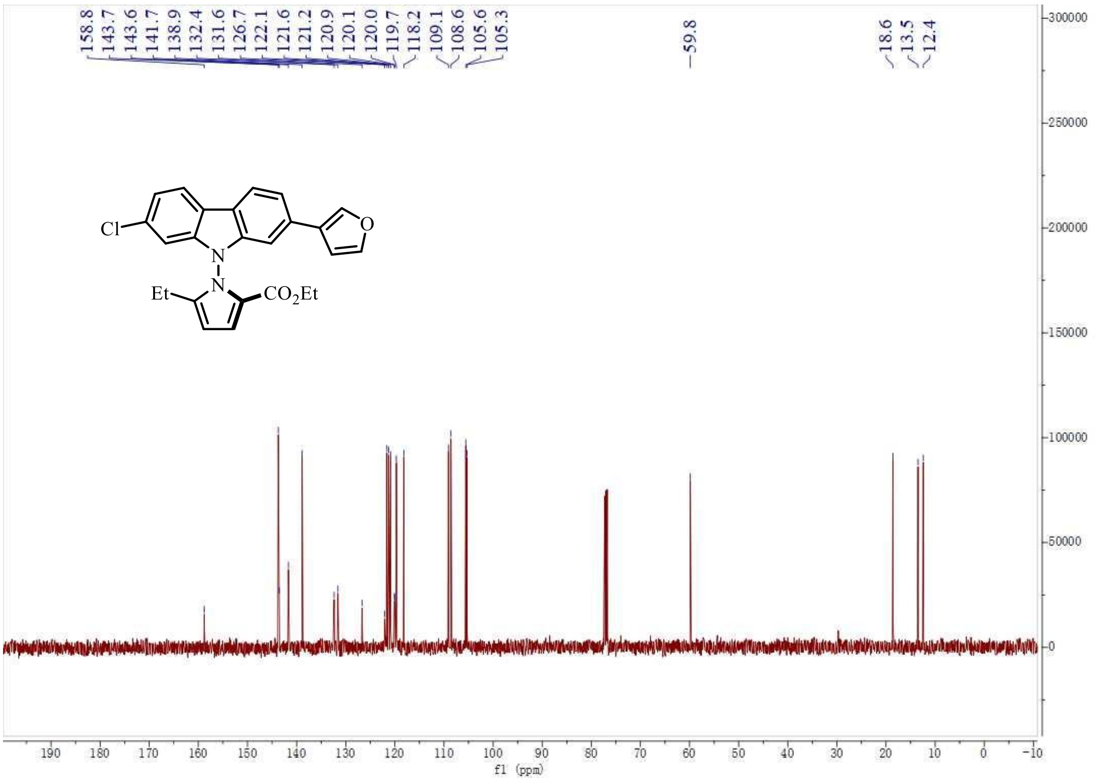

Ethyl (*R*)-1-(2-chloro-7-(2-methoxypyrimidin-5-yl)-9*H*-carbazol-9-yl)-5-ethyl-1*H*-pyrrole-2-carboxylate (44) ^{1}H NMR (500 MHz, Chloroform-*d*)

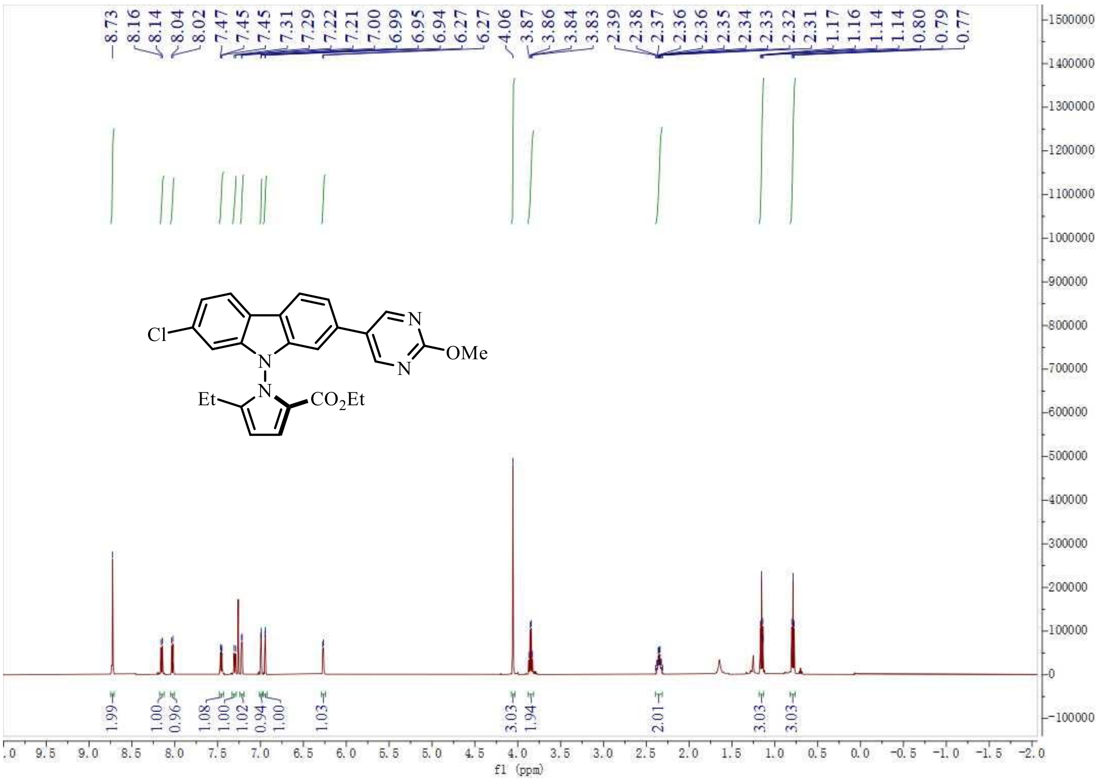

^{13}C NMR (101 MHz, Chloroform-*d*)

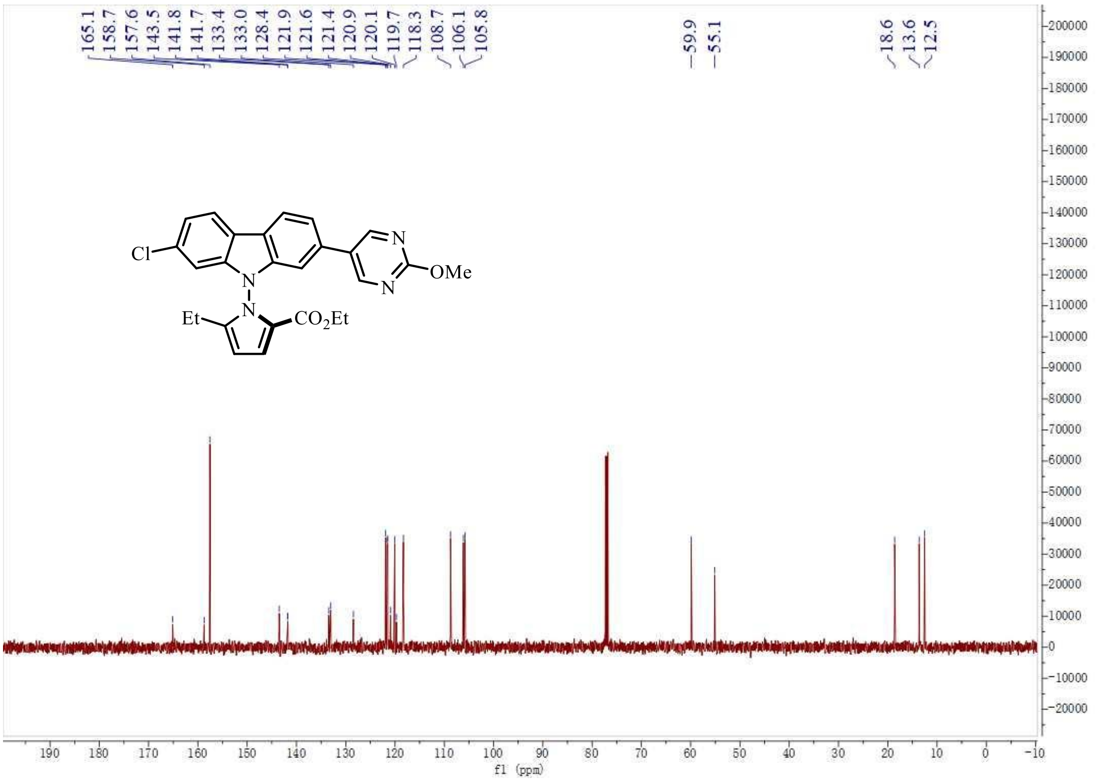

Ethyl (*R*)-1-(2-chloro-7-(9,9-dimethyl-9*H*-fluoren-2-yl)-9*H*-carbazol-9-yl)-5-ethyl-1*H*-pyrrole-2-carboxylate (45) ^{1}H NMR (500 MHz, Chloroform-*d*)

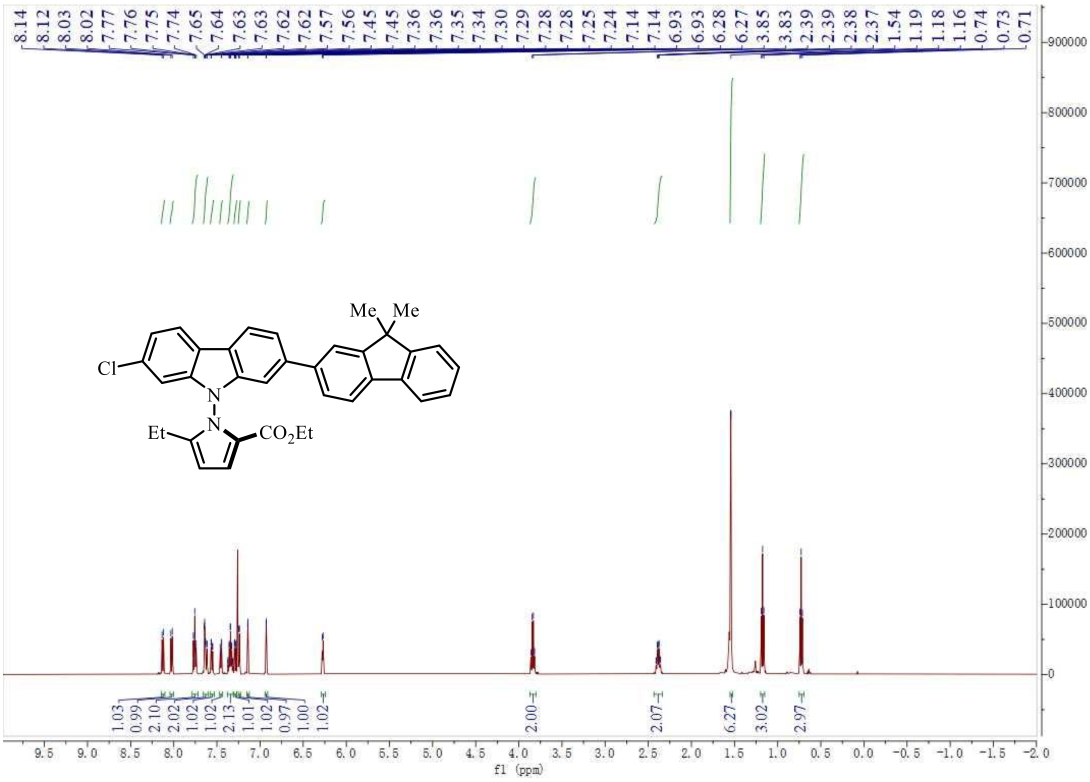

^{13}C NMR (126 MHz, Chloroform-*d*)

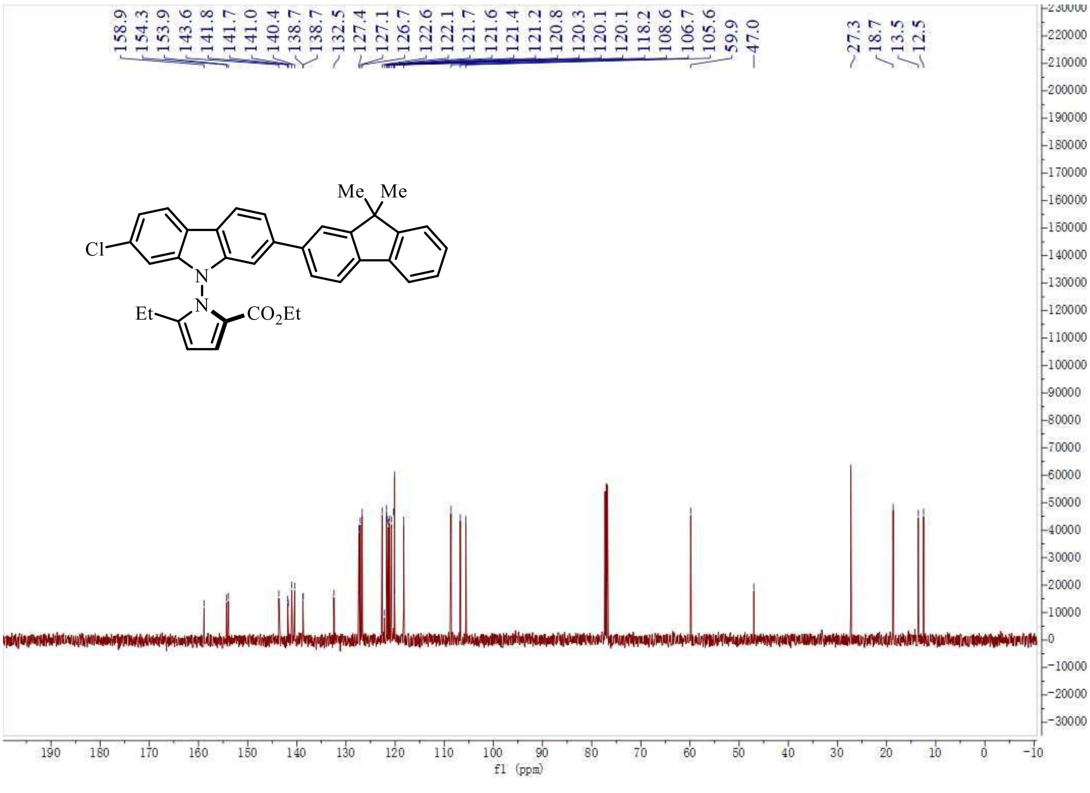

Ethyl (*R*)-1-(2-chloro-7-(naphthalen-2-yl)-9*H*-carbazol-9-yl)-5-ethyl-1*H*-pyrrole-2-carboxylate (46) ^{1}H NMR (500 MHz, Chloroform-*d*)

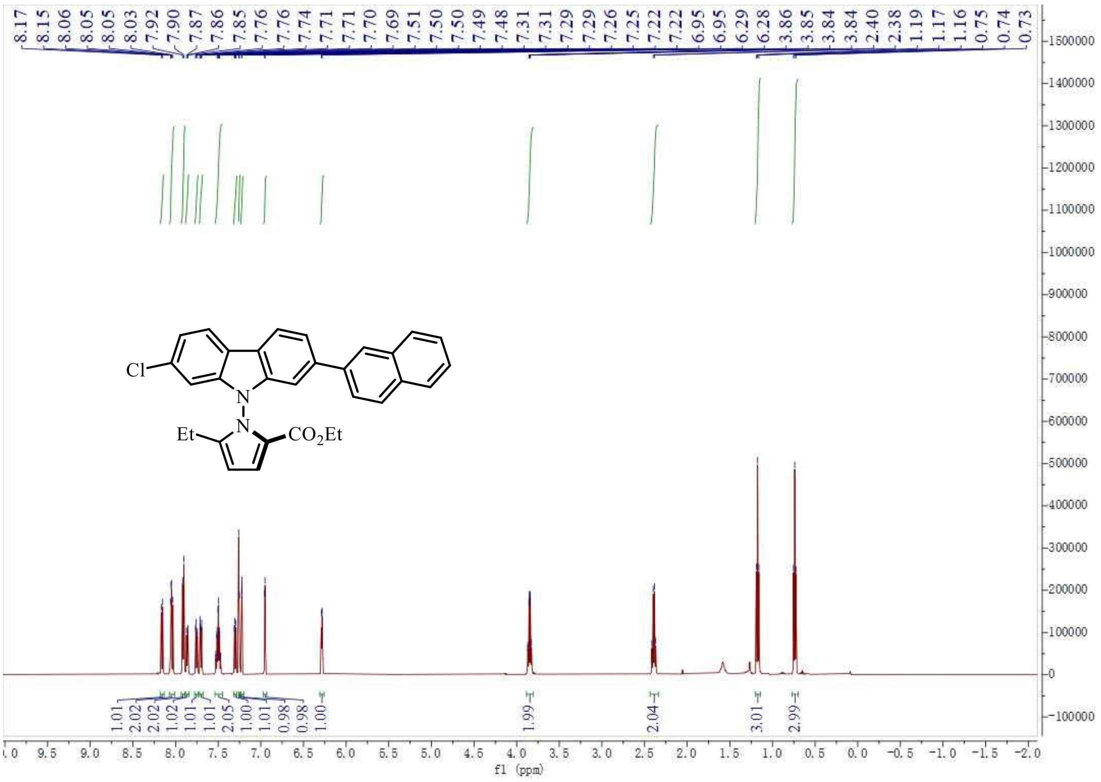

^{13}C NMR (126 MHz, Chloroform-*d*)

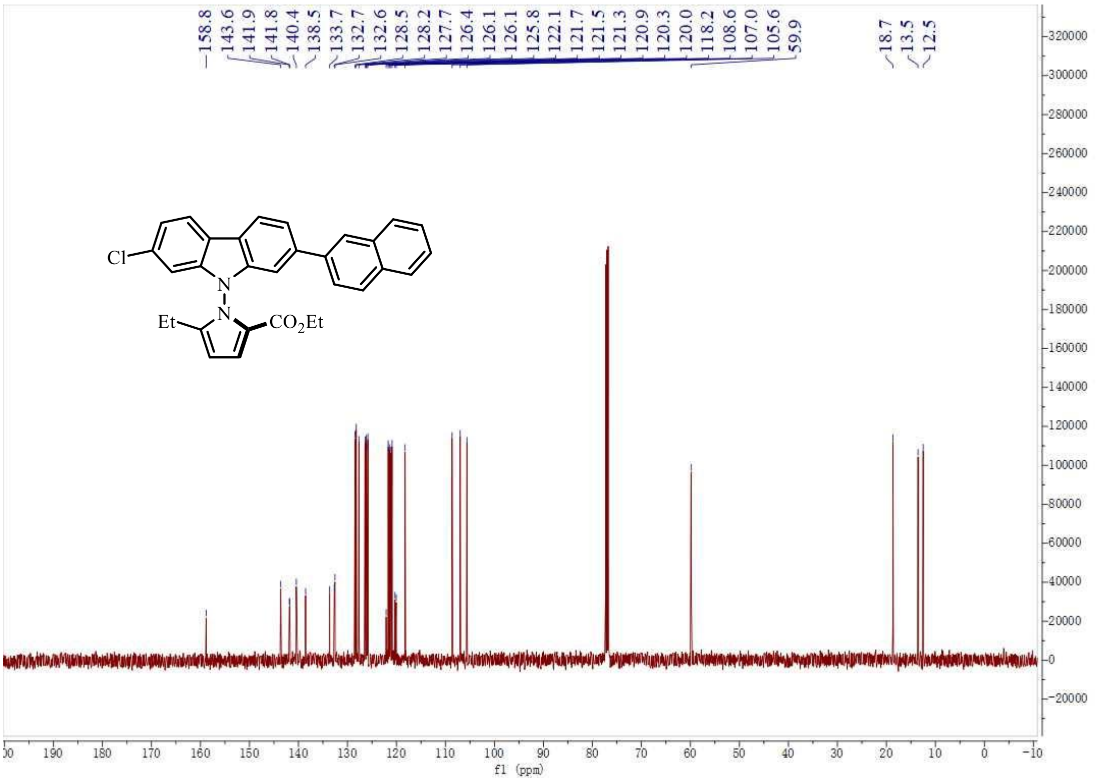

Ethyl (*R*)-1-(2-chloro-7-(dibenzo[b,d]thiophen-4-yl)-9*H*-carbazol-9-yl)-5-ethyl-1*H*-pyrrole-2-carboxylate (47) ^{1}H NMR (500 MHz, Chloroform-*d*)

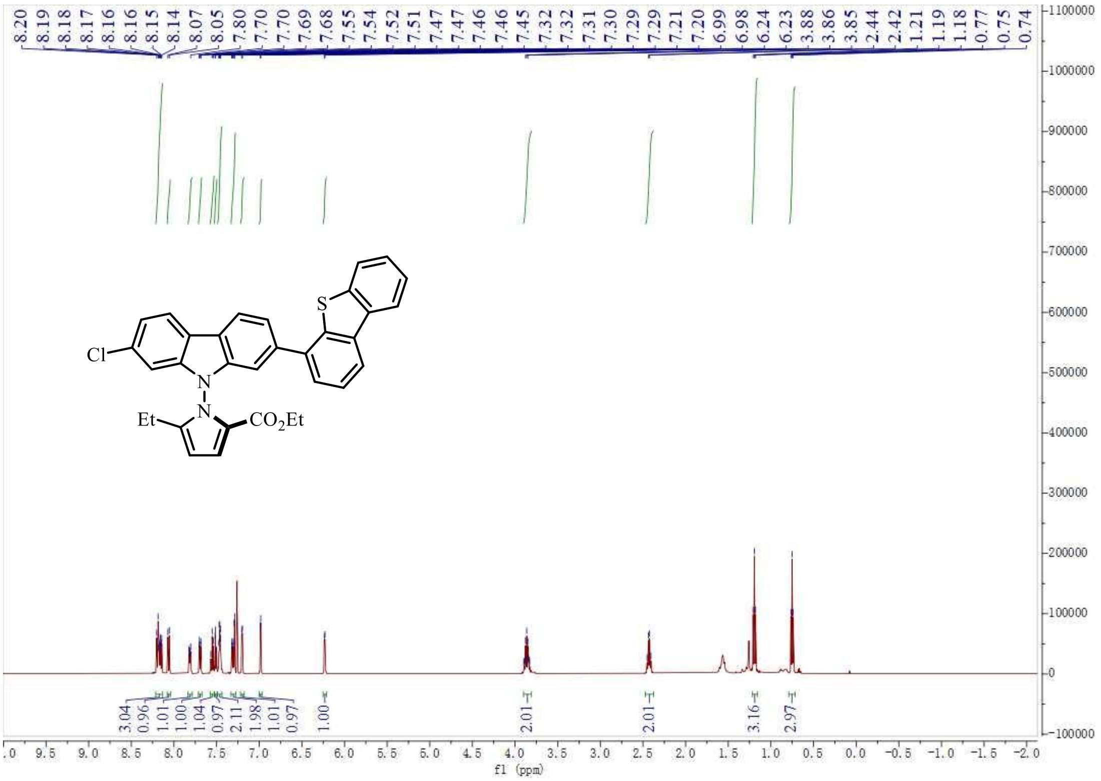

^{13}C NMR (126 MHz, Chloroform-*d*)

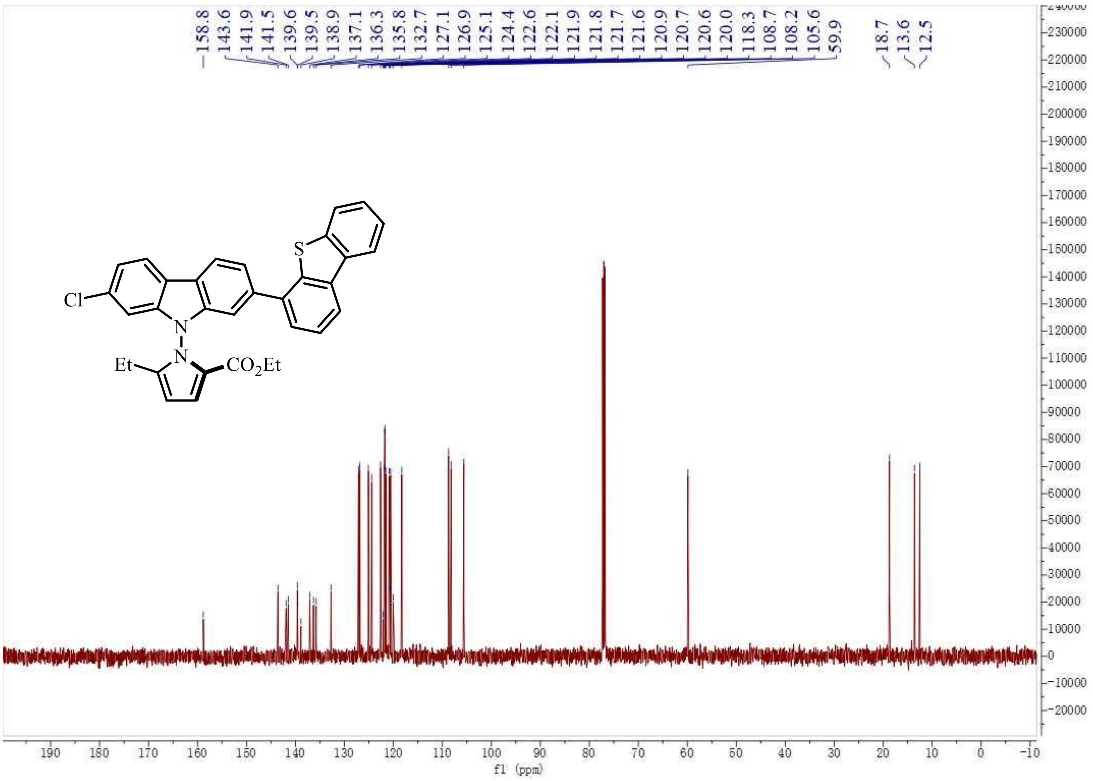

Ethyl (*R*)-1-(7-chloro-9'-phenyl-9*H*,9'*H*-[2,3'-bicarbazol]-9-yl)-5-ethyl-1*H*-pyrrole-2-carboxylate (48) ^{1}H NMR (500 MHz, Chloroform-*d*)

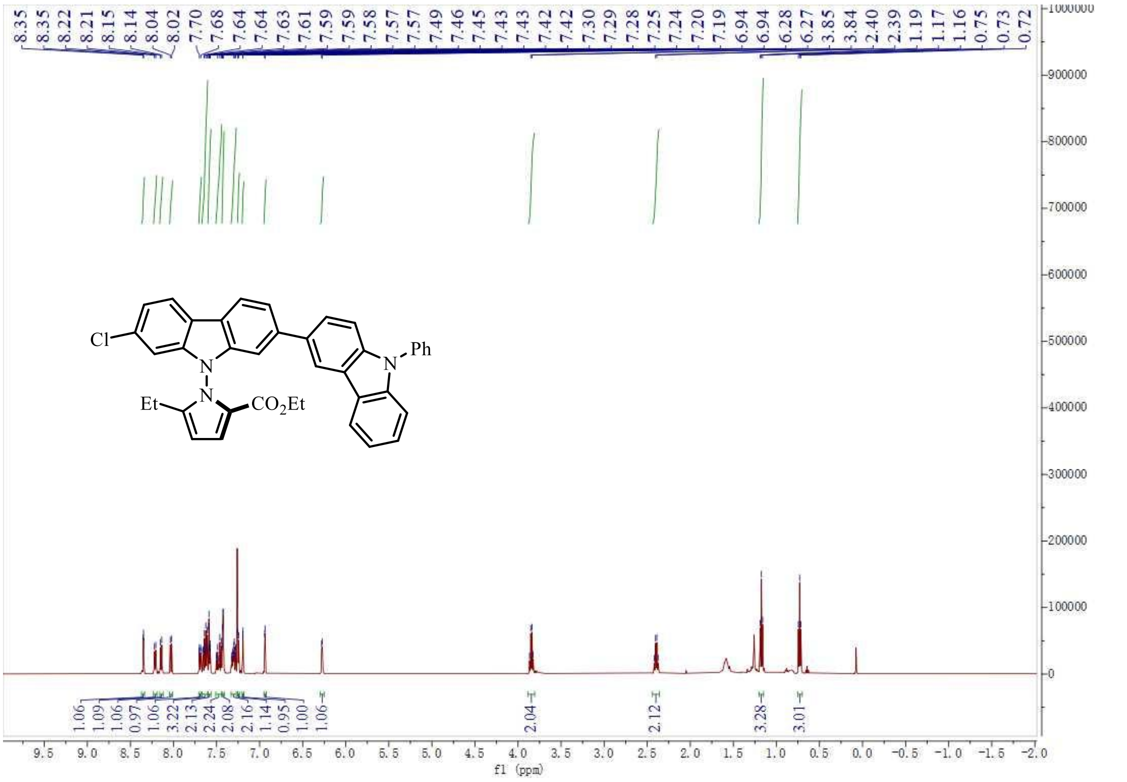

^{13}C NMR (126 MHz, Chloroform-*d*)

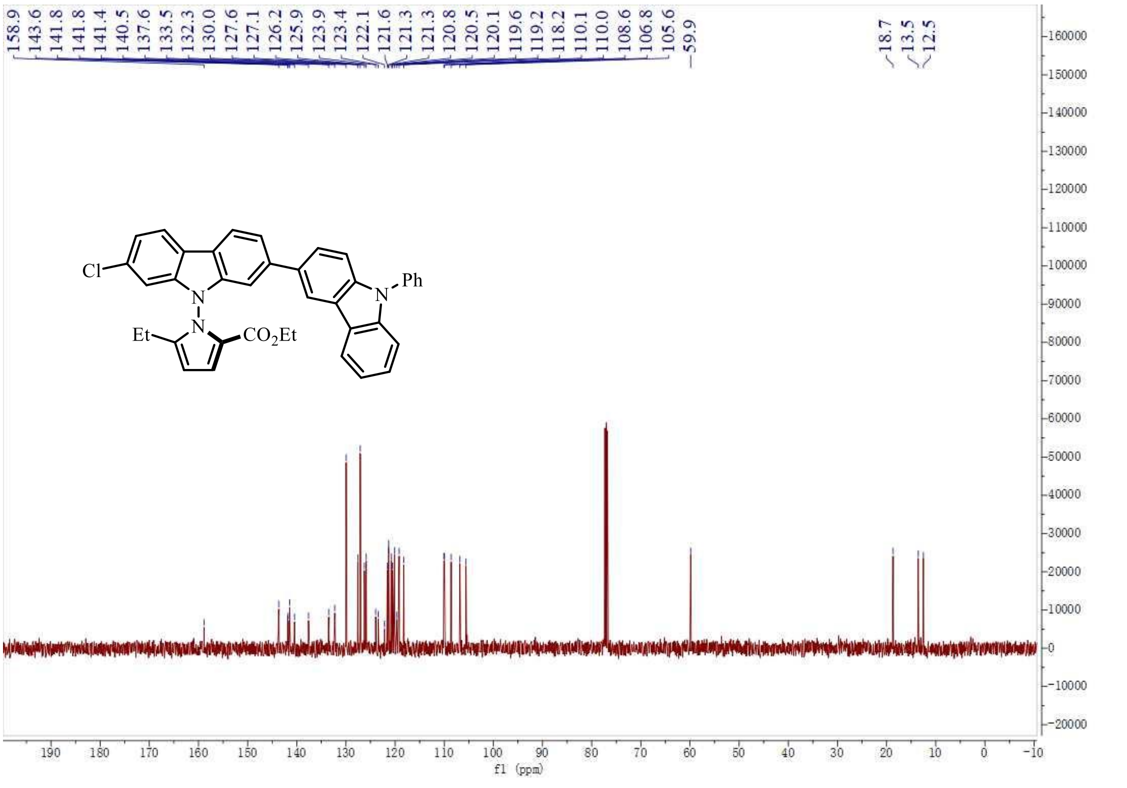

Ethyl (*S*)-1-(2-chloro-7-(4-methoxyphenyl)-9*H*-carbazol-9-yl)-2-methyl-5-phenyl-1*H*-pyrrole-3-carboxylate (49) ^{1}H NMR (500 MHz, Chloroform-*d*)

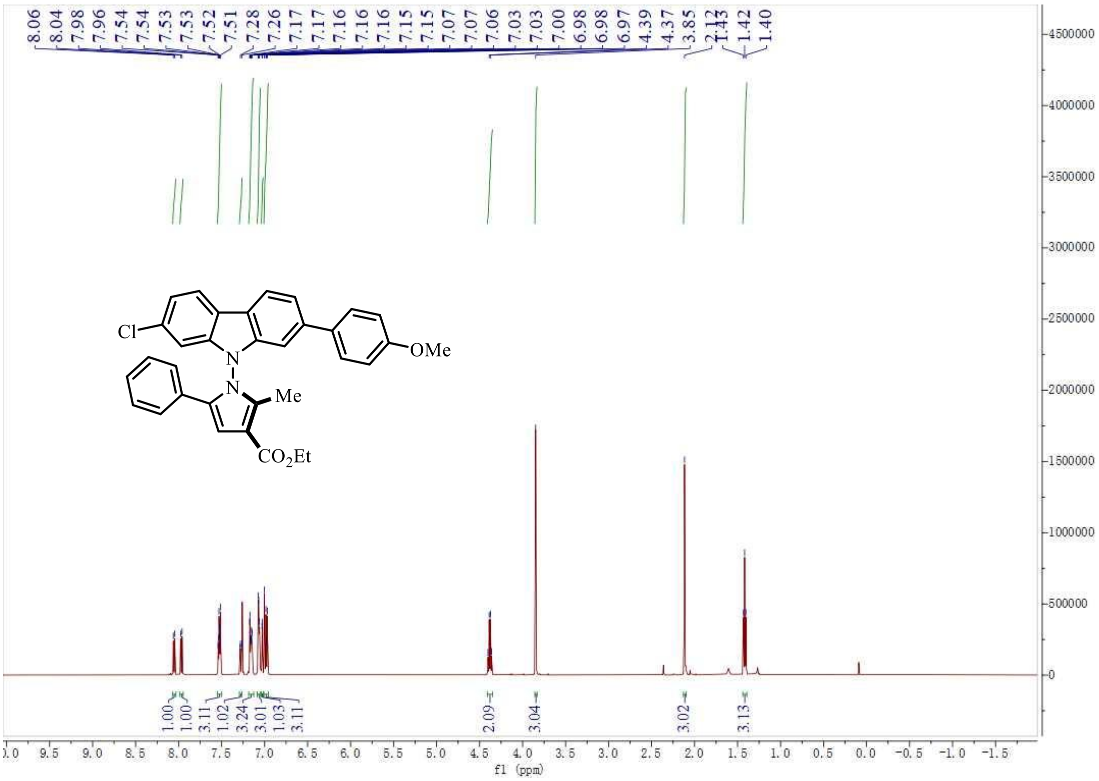

^{13}C NMR (126 MHz, Chloroform-*d*)

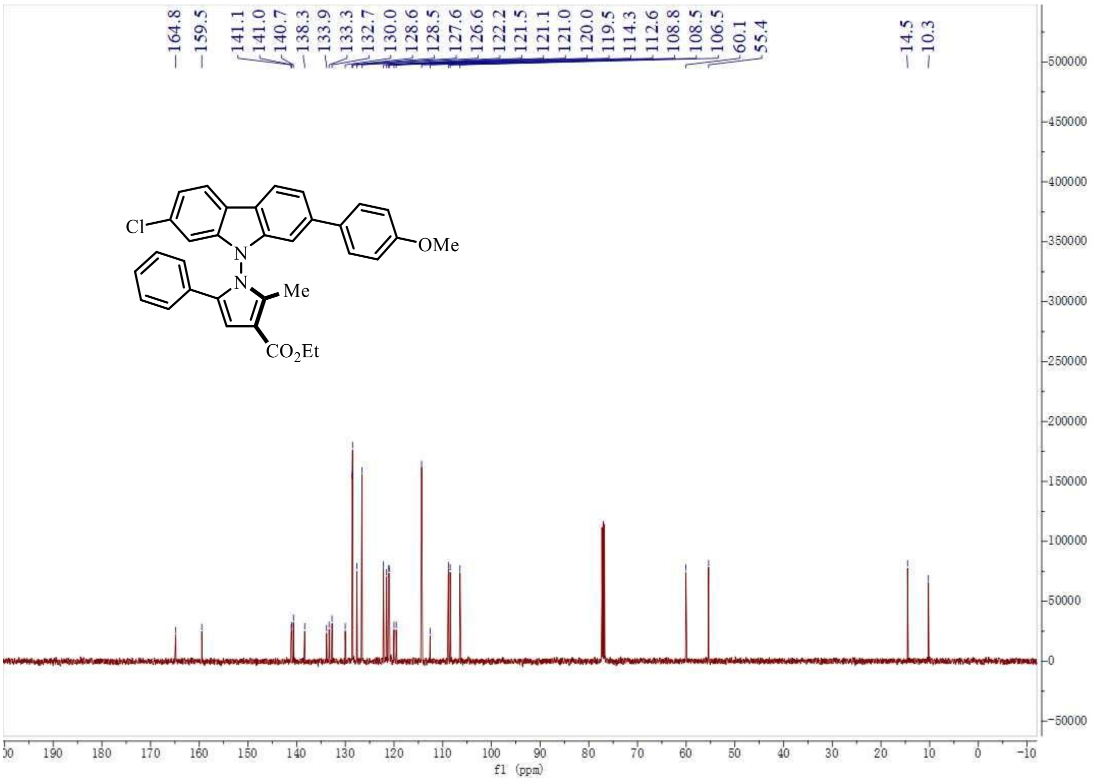

Ethyl (*S*)-1-(2-chloro-7-(4-methoxyphenyl)-9*H*-carbazol-9-yl)-2-methyl-5-(naphthalen-2-yl)-1*H*-pyrrole-3-carboxylate (50) ^{1}H NMR (500 MHz, Chloroform-*d*)

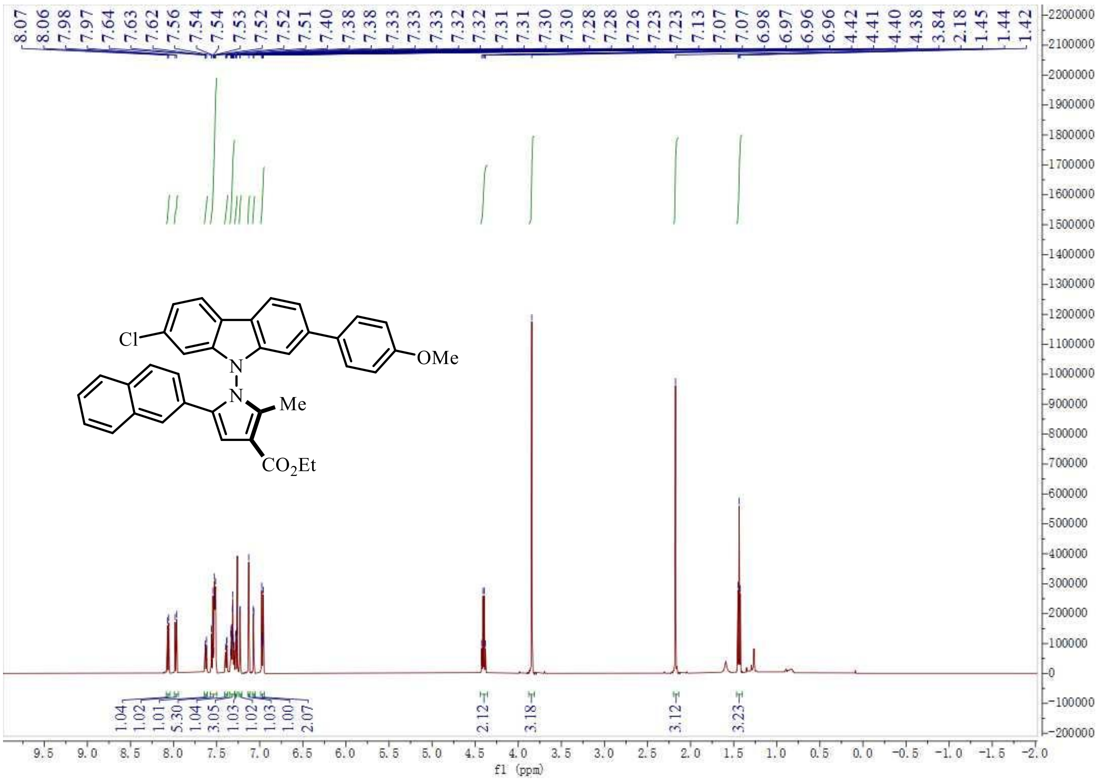

^{13}C NMR (126 MHz, Chloroform-*d*)

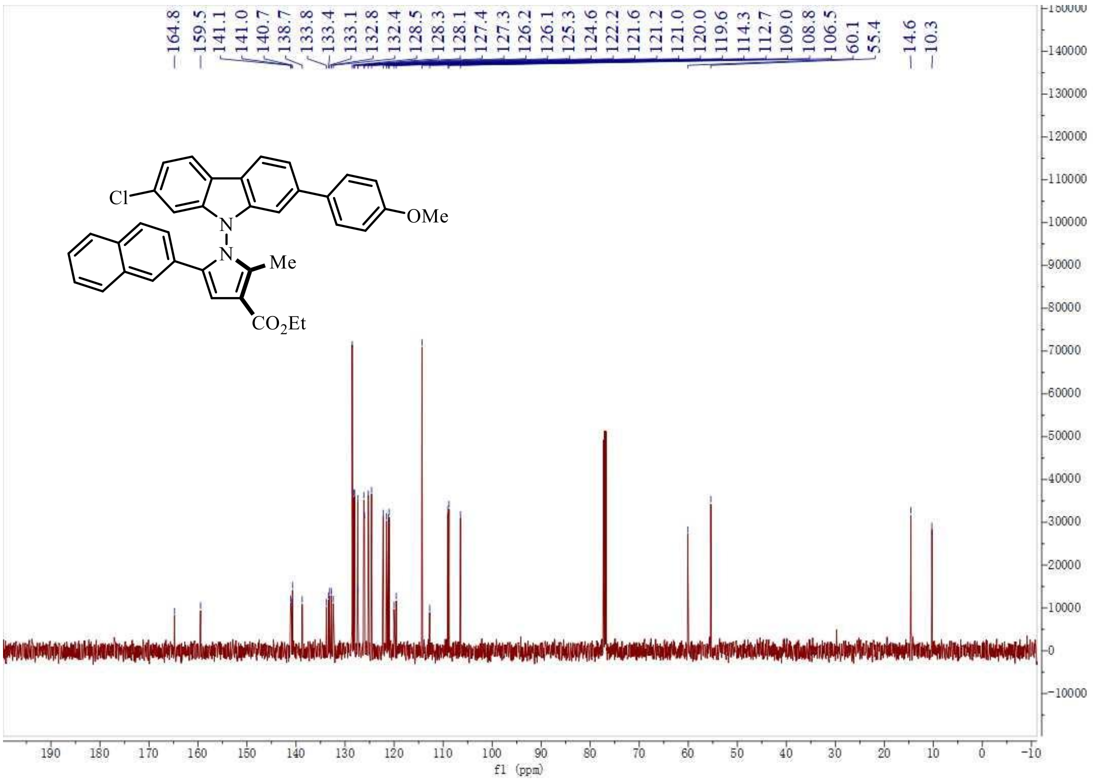

Ethyl (*S*)-1-(2-chloro-7-(4-methoxyphenyl)-9*H*-carbazol-9-yl)-5-(3-methoxyphenyl)-2-methyl-1*H*-pyrrole-3-carboxylate (51) ^{1}H NMR (500 MHz, Chloroform-*d*)

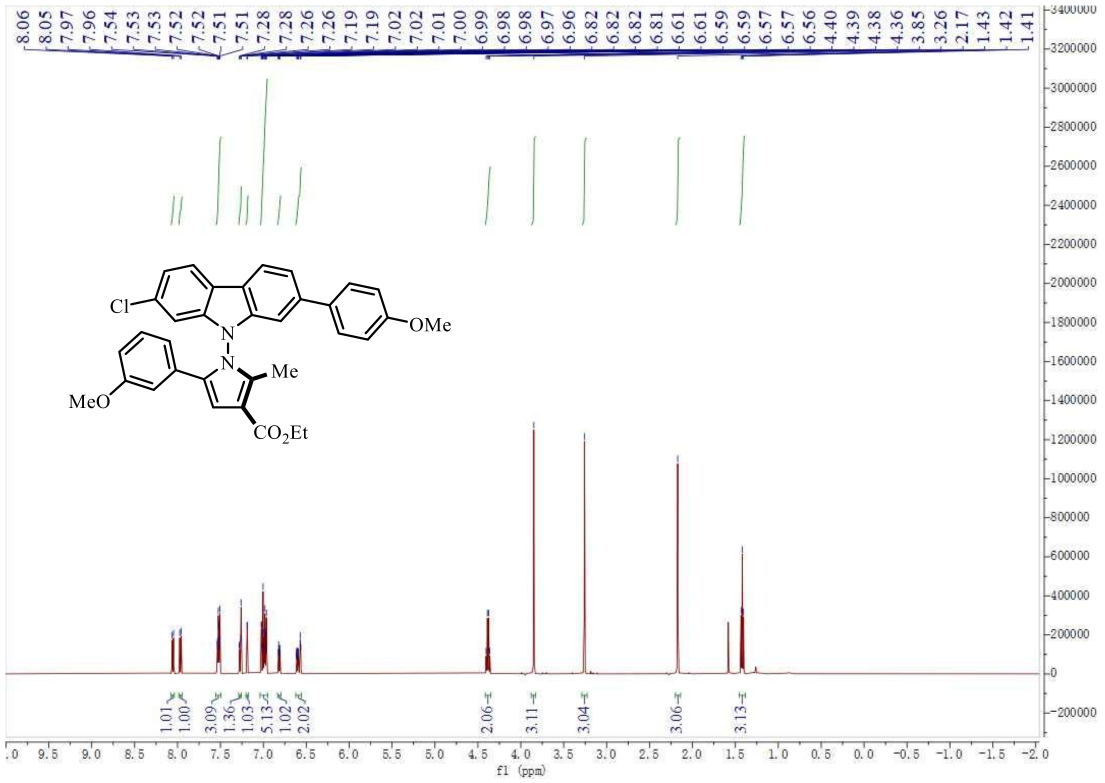

^{13}C NMR (126 MHz, Chloroform-*d*)

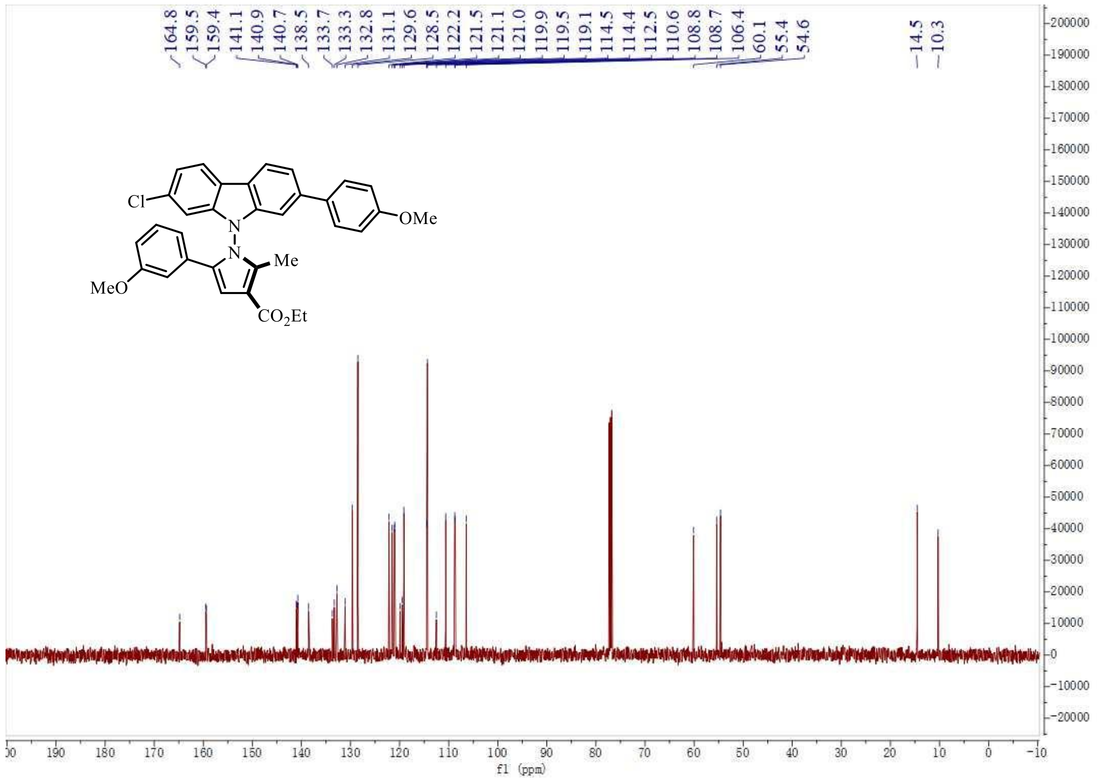

Ethyl (*S*)-1-(2-chloro-7-(4-methoxyphenyl)-9*H*-carbazol-9-yl)-2-methyl-5-(3-nitrophenyl)-1*H*-pyrrole-3-carboxylate (52) ^{1}H NMR (500 MHz, Chloroform-*d*)

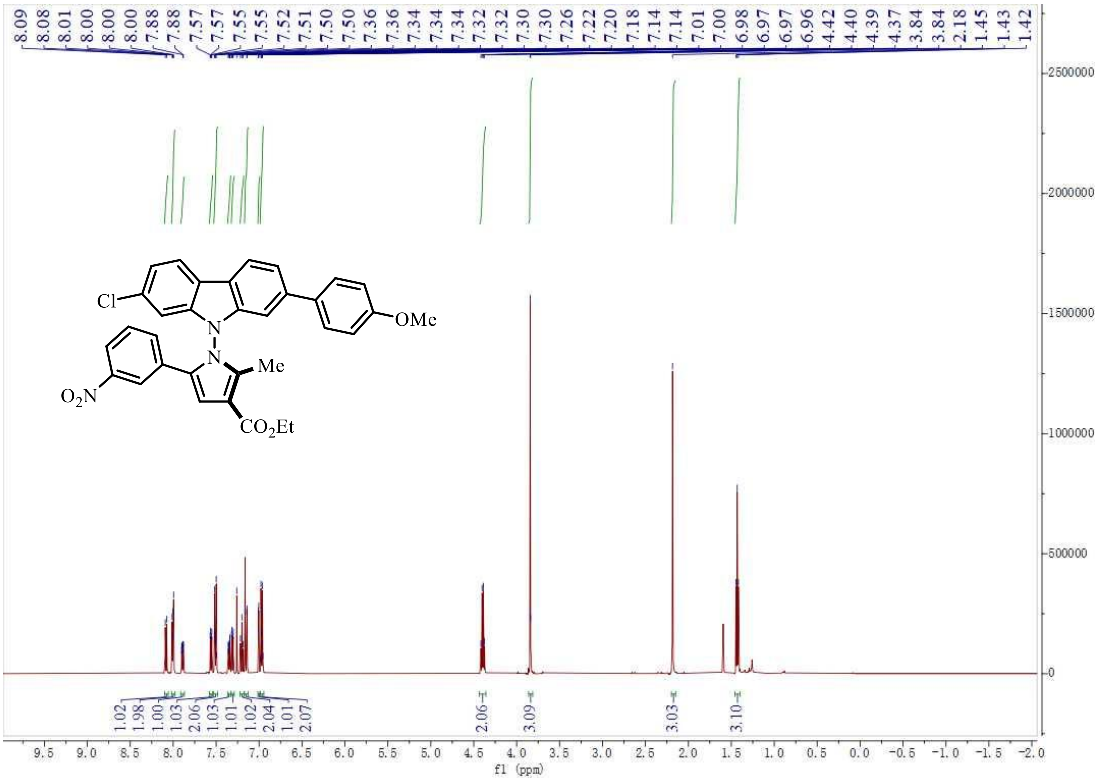

^{13}C NMR (126 MHz, Chloroform-*d*)

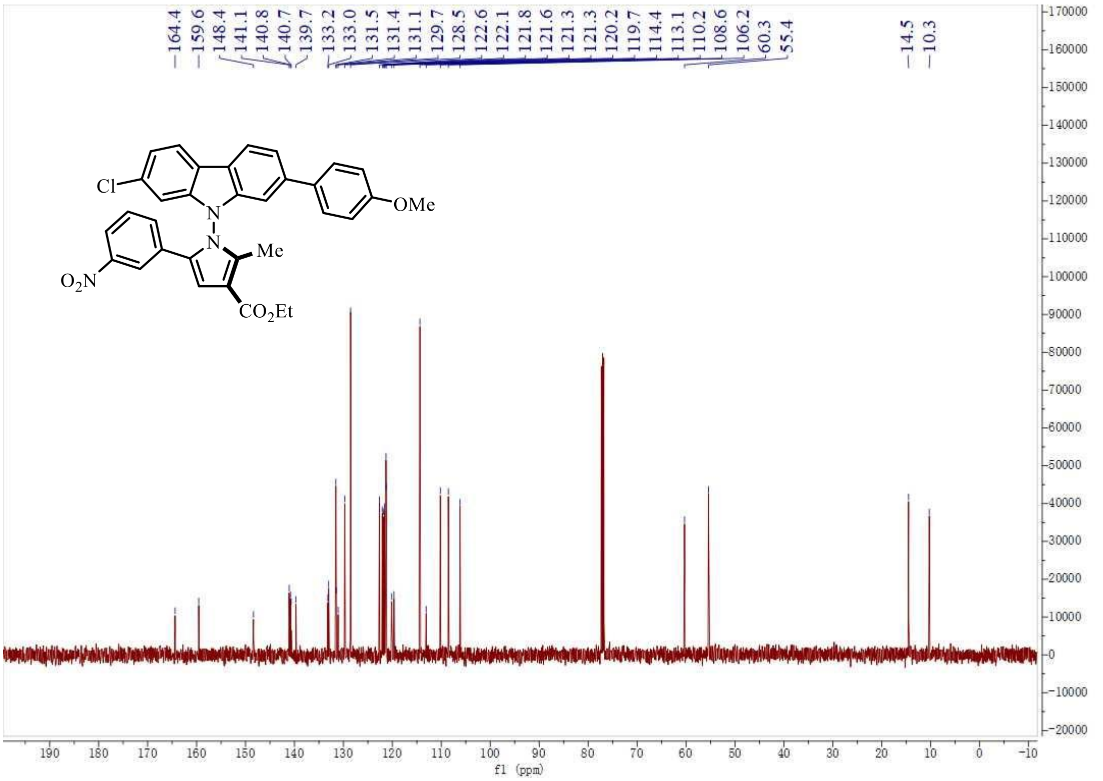

Ethyl (*S*)-5-(benzofuran-2-yl)-1-(2-chloro-7-(4-methoxyphenyl)-9*H*-carbazol-9-yl)-2-methyl-1*H*-pyrrole-3-carboxylate (53) ^{1}H NMR (400 MHz, Chloroform-*d*)

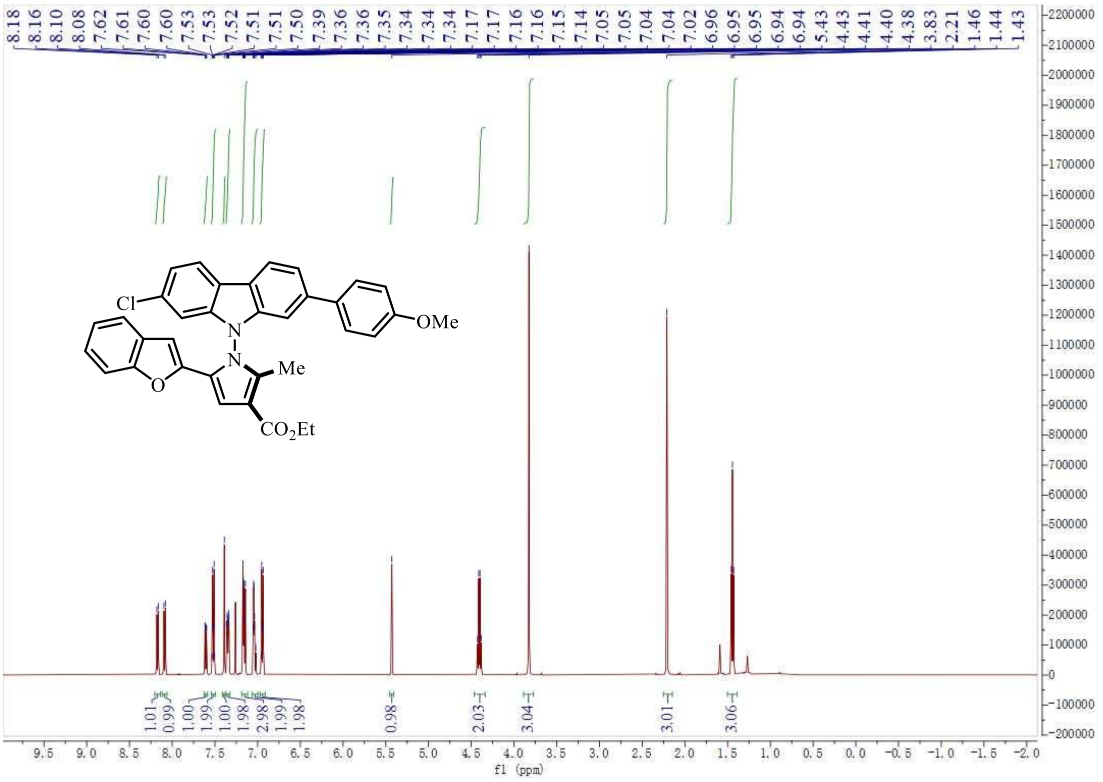

^{13}C NMR (101 MHz, Chloroform-*d*)

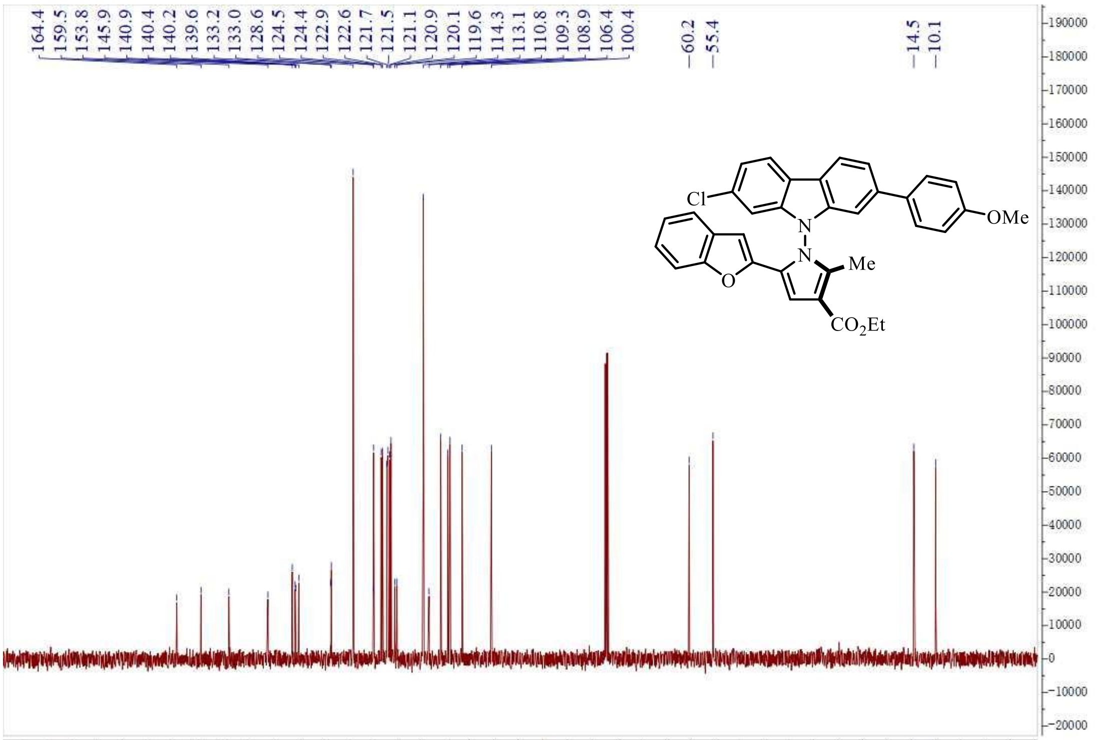

Ethyl (*S*)-1-(2-chloro-7-(4-methoxyphenyl)-9*H*-carbazol-9-yl)-2-methyl-5-(thiophen-2-yl)-1*H*-pyrrole-3-carboxylate (54) ^{1}H NMR (500 MHz, Chloroform-*d*)

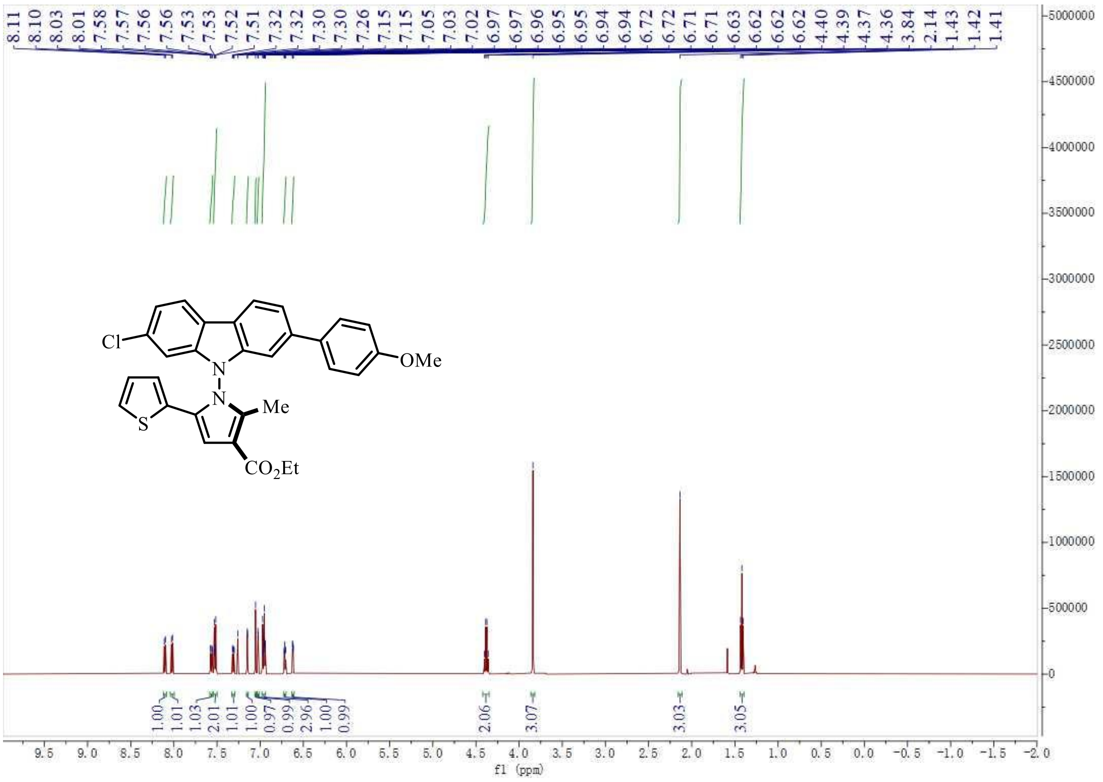

^{13}C NMR (126 MHz, Chloroform-*d*)

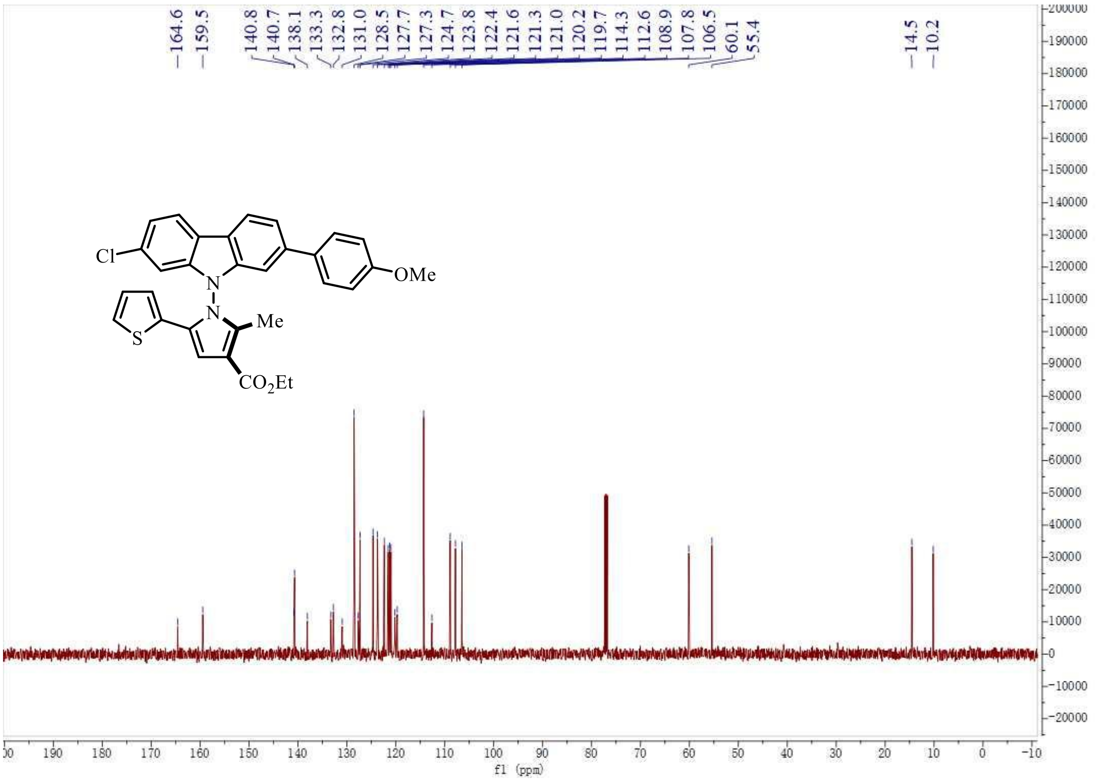

Ethyl (*R*)-1-(2-chloro-7-(4-methoxyphenyl)-9*H*-carbazol-9-yl)-5-cyclopropyl-2-methyl-1*H*-pyrrole-3-carboxylate (55) ^{1}H NMR (500 MHz, Chloroform-*d*)

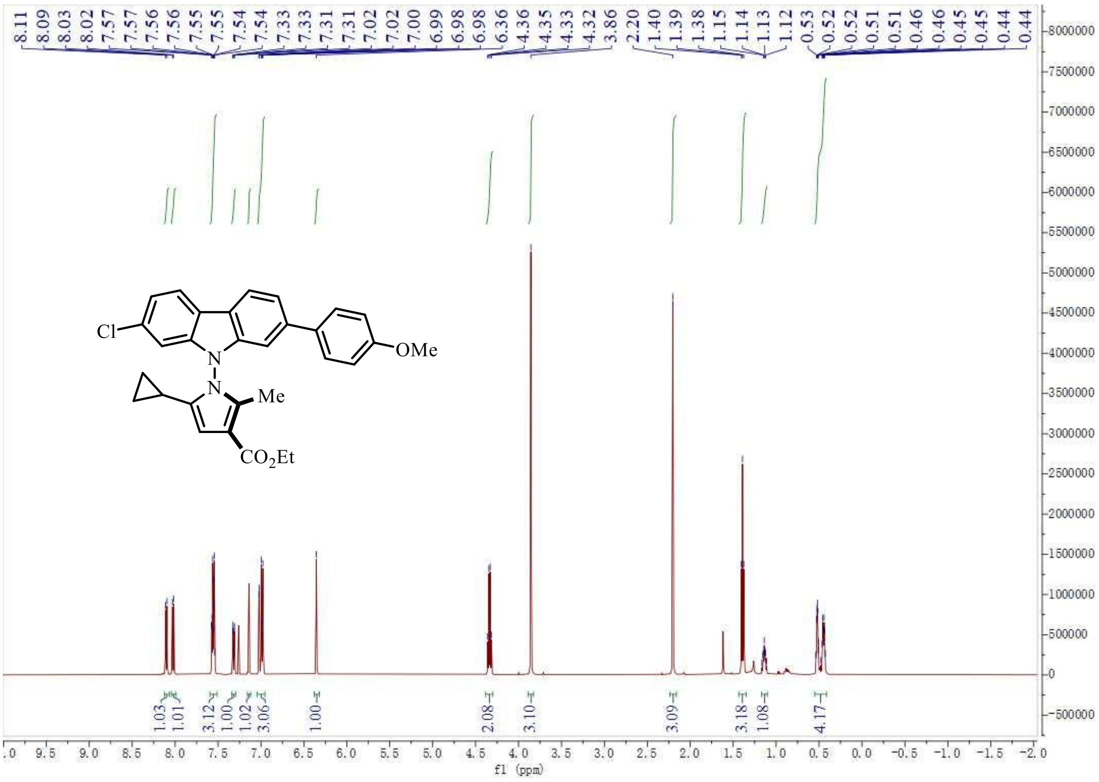

^{13}C NMR (126 MHz, Chloroform-*d*)

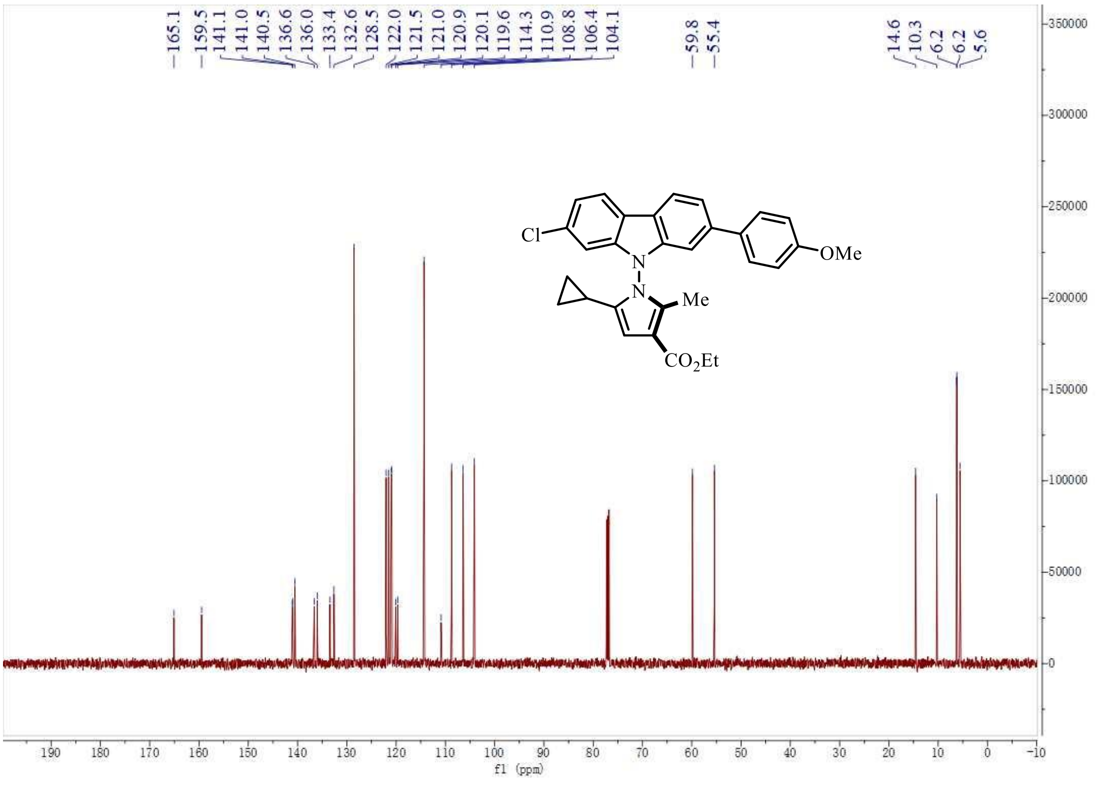

Ethyl (*S*)-1-(2-chloro-7-(4-methoxyphenyl)-9*H*-carbazol-9-yl)-2-ethyl-5-phenyl-1*H*-pyrrole-3-carboxylate (56) ^{1}H NMR (500 MHz, Chloroform-*d*)

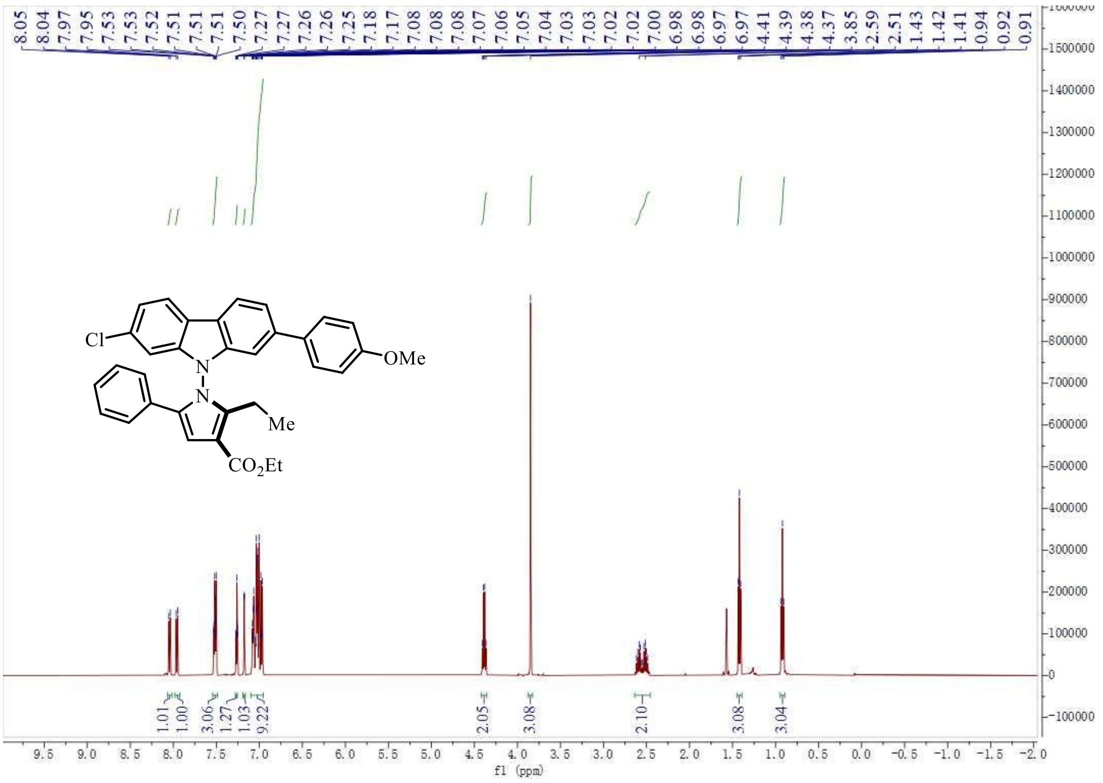

^{13}C NMR (126 MHz, Chloroform-*d*)

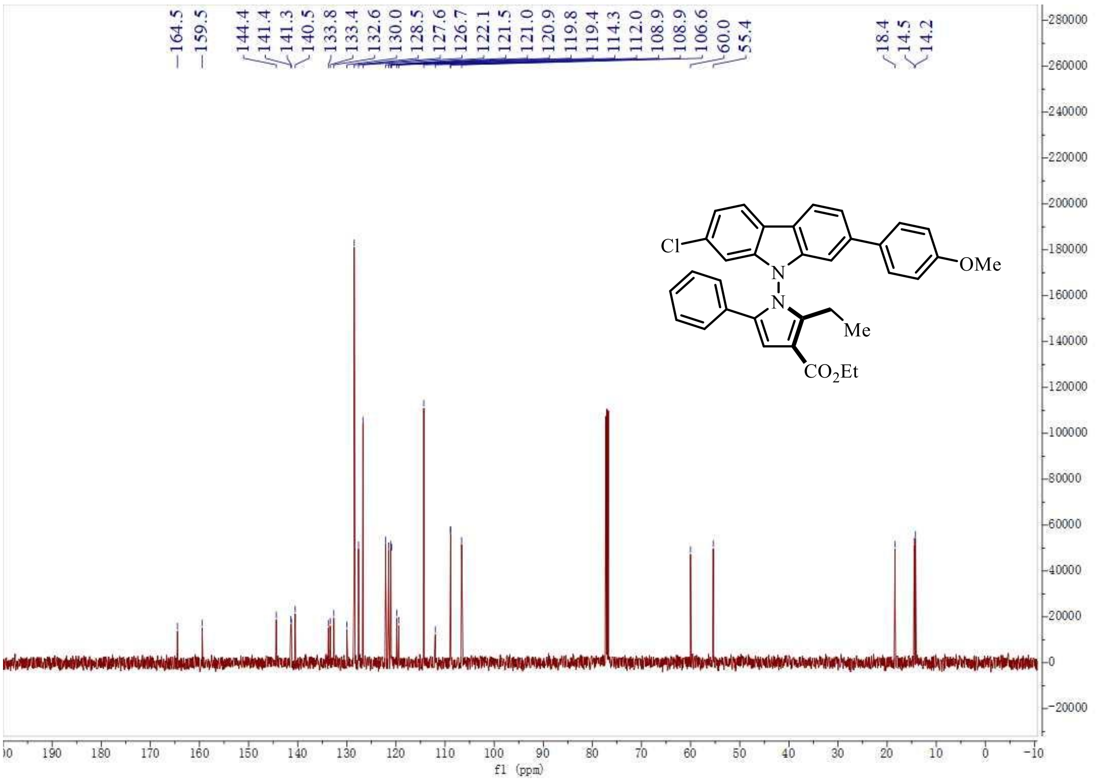

Methyl (*S*)-2-butyl-1-(2-chloro-7-(4-methoxyphenyl)-9*H*-carbazol-9-yl)-5-phenyl-1*H*-pyrrole-3-carboxylate (57) ^{1}H NMR (500 MHz, Chloroform-*d*)

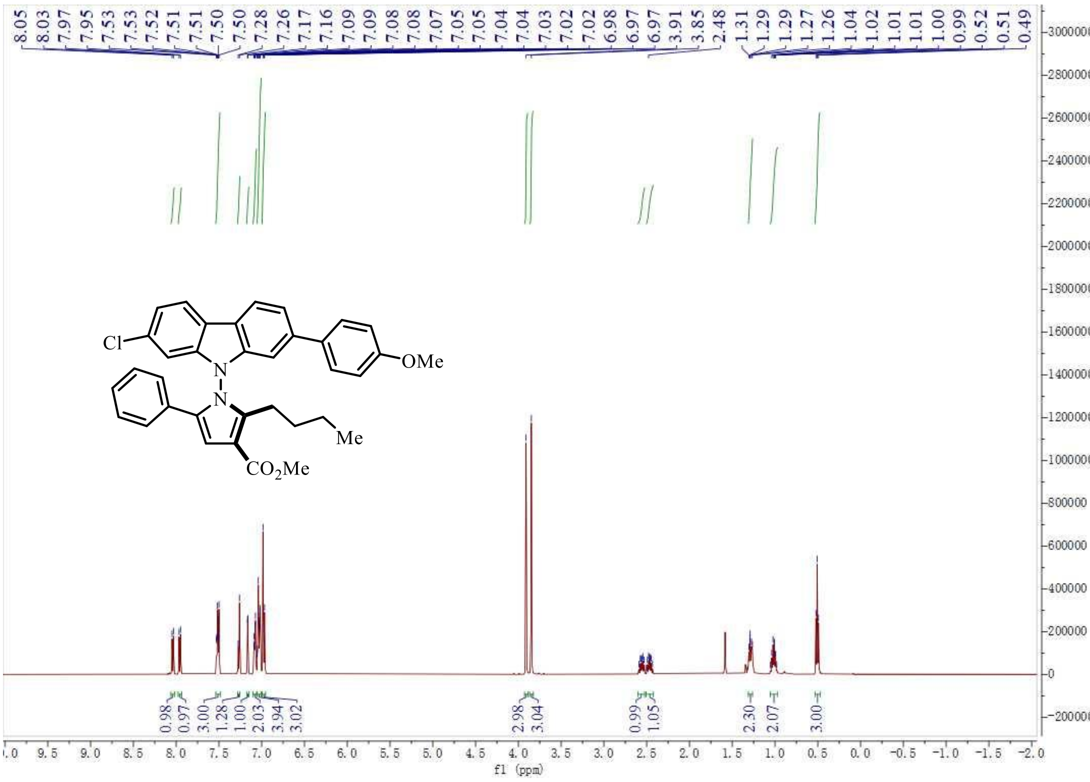

^{13}C NMR (126 MHz, Chloroform-*d*)

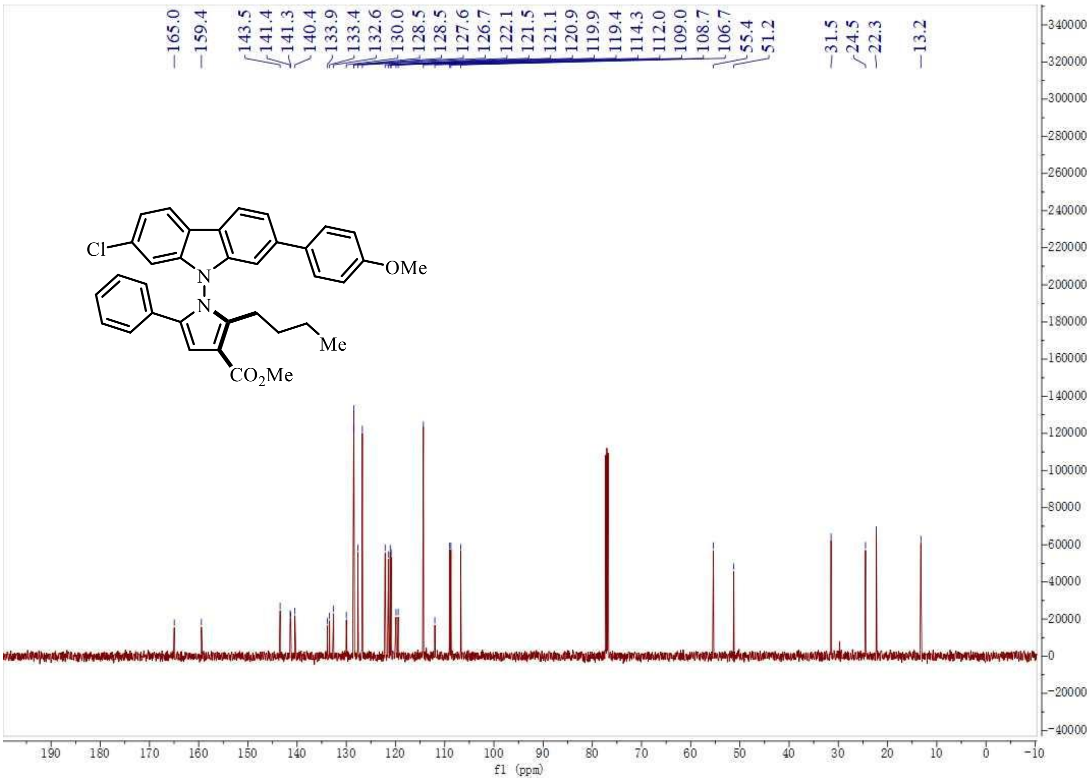

Ethyl (*S*)1-(2-chloro-7-((4-methoxyphenyl)ethynyl)-9*H*-carbazol-9-yl)-2-methyl-5-phenyl-1*H*-pyrrole-3-carboxylate (58) ^{1}H NMR (500 MHz, Chloroform-*d*)

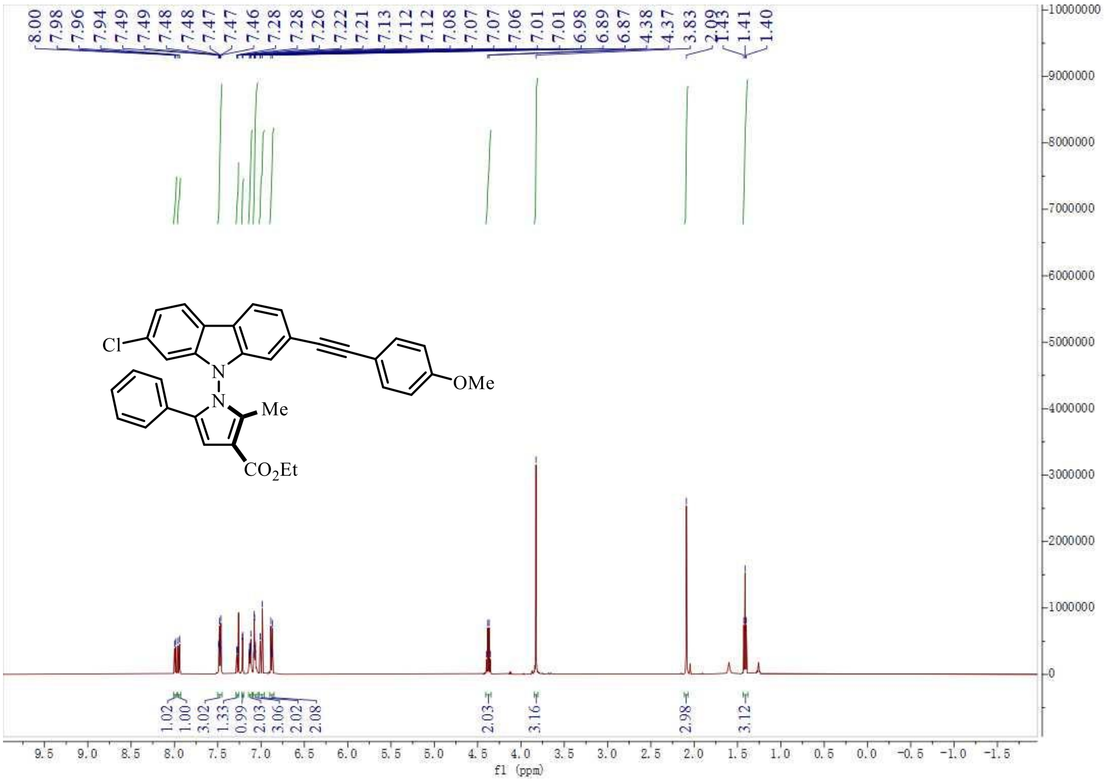

^{13}C NMR (126 MHz, Chloroform-*d*)

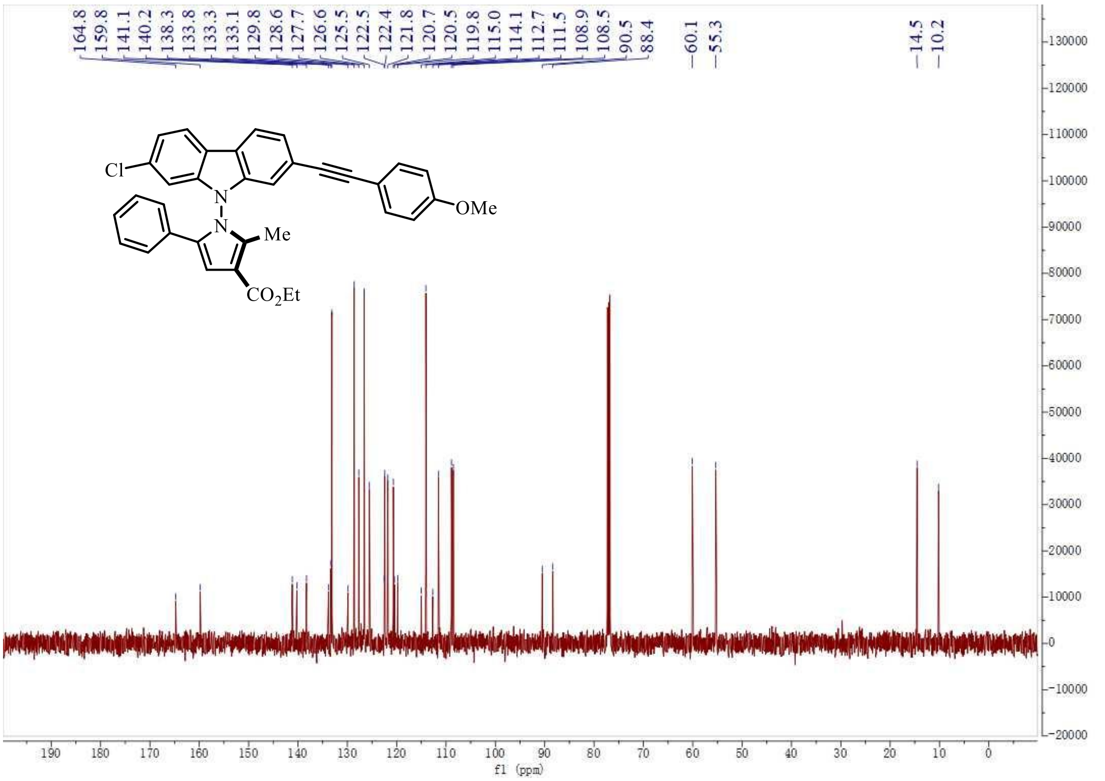

Ethyl (*R*)-5-cyclopropyl-1-(2-(4-methoxyphenyl)-7-((4-methoxyphenyl)ethynyl)-9*H*-carbazol-9-yl)-2-methyl-1*H*-pyrrole-3-carboxylate (59) ^{1}H NMR (500 MHz, Chloroform-*d*)

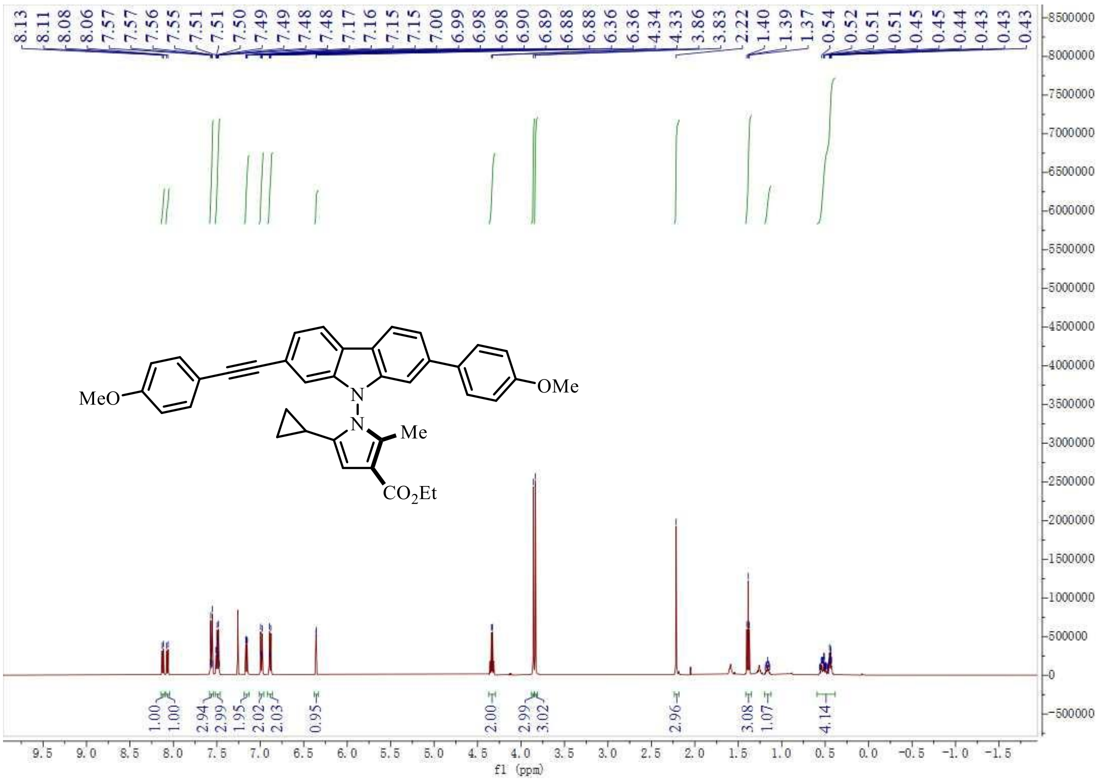

^{13}C NMR (126 MHz, Chloroform-*d*)

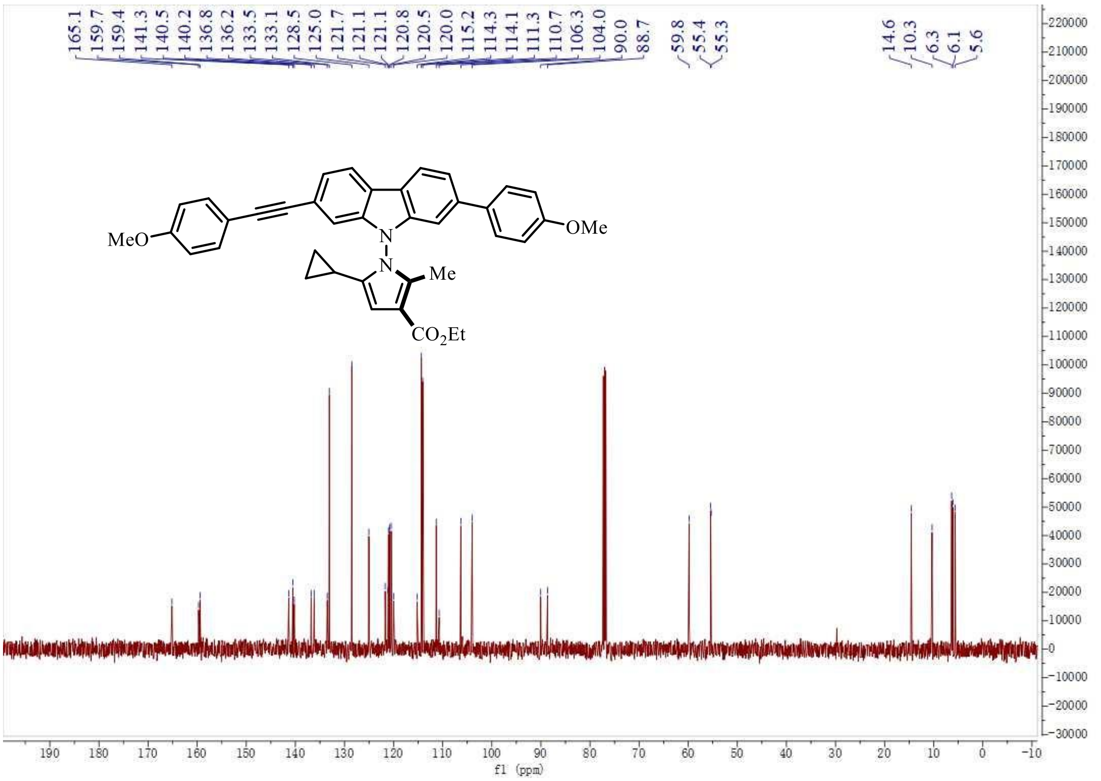

Ethyl (*S*)-1-(3-chloro-6-(4-methoxyphenyl)-9*H*-carbazol-9-yl)-2-methyl-5-phenyl-1*H*-pyrrole-3-carboxylate (61) ^{1}H NMR (500 MHz, Chloroform-*d*)

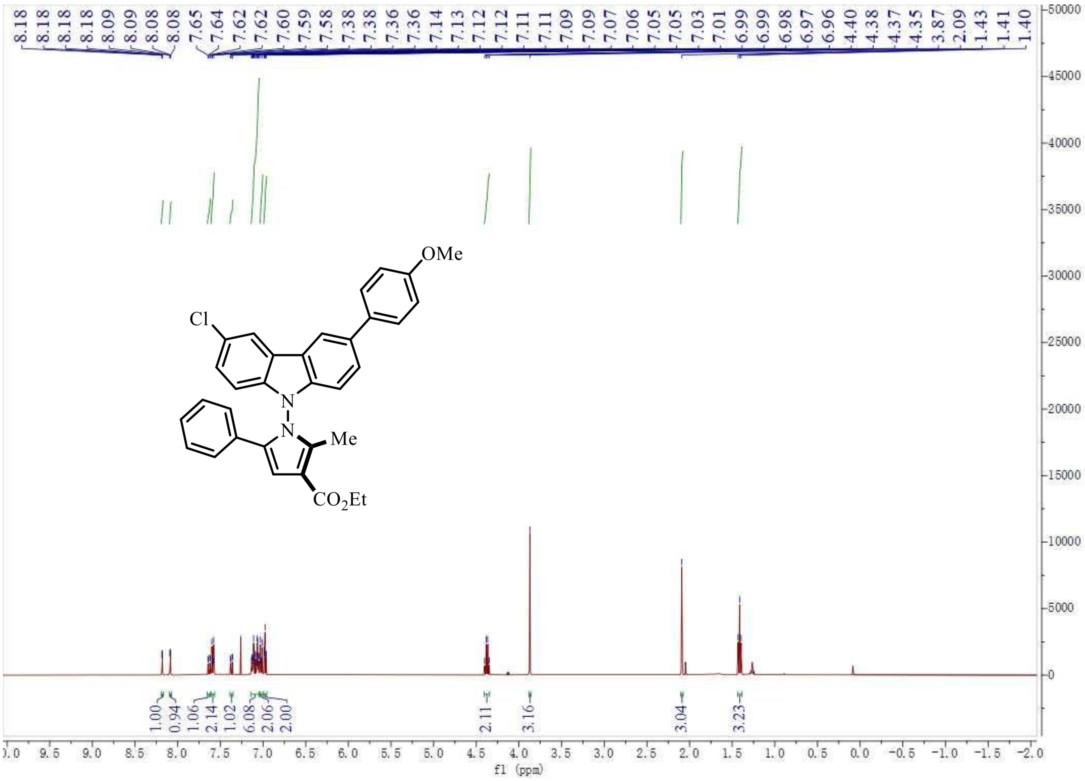

^{13}C NMR (126 MHz, Chloroform-*d*)

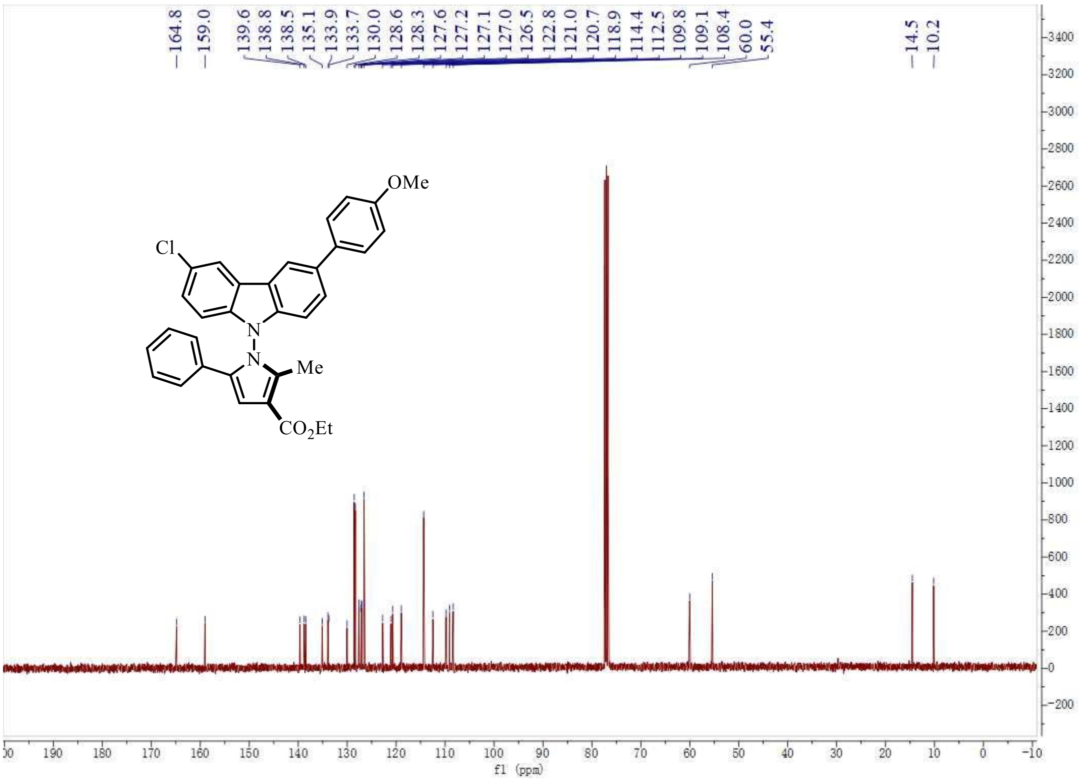

Ethyl (*R*)-1-(3-(4-cyanophenyl)-6-(4-methoxyphenyl)-9*H*-carbazol-9-yl)-2-methyl-5-phenyl-1*H*-pyrrole-3-carboxylate (62) ^{1}H NMR (500 MHz, Chloroform-*d*)

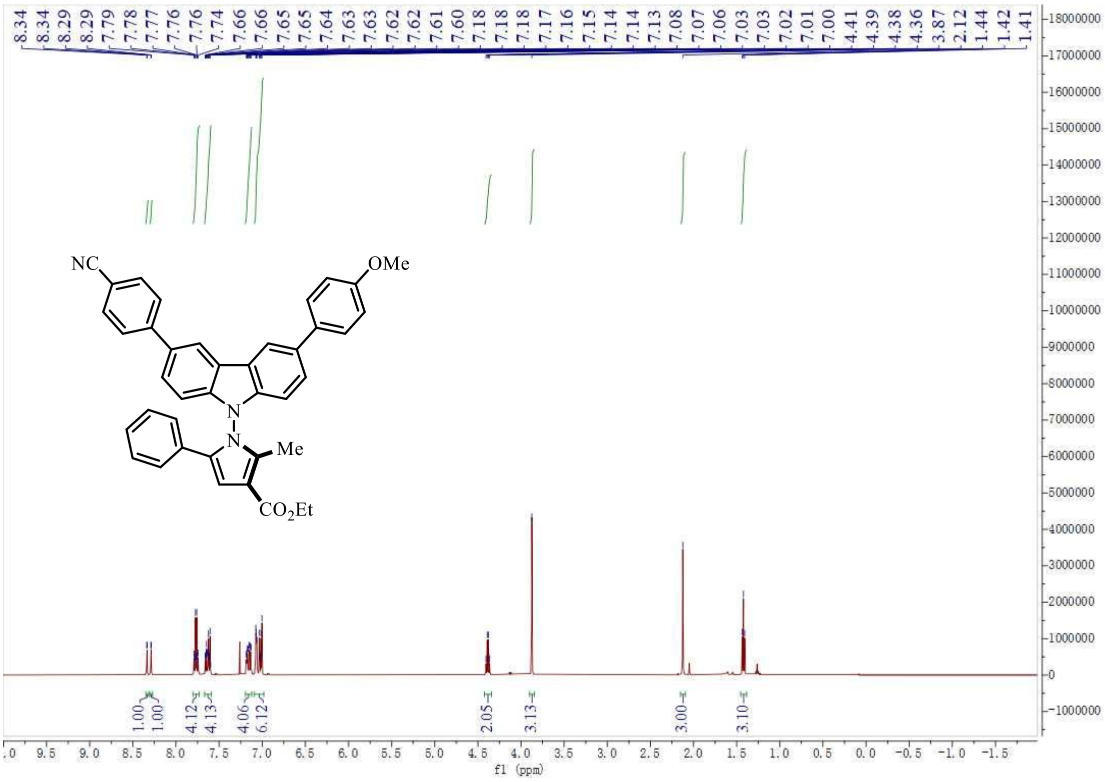

^{13}C NMR (126 MHz, Chloroform-*d*)

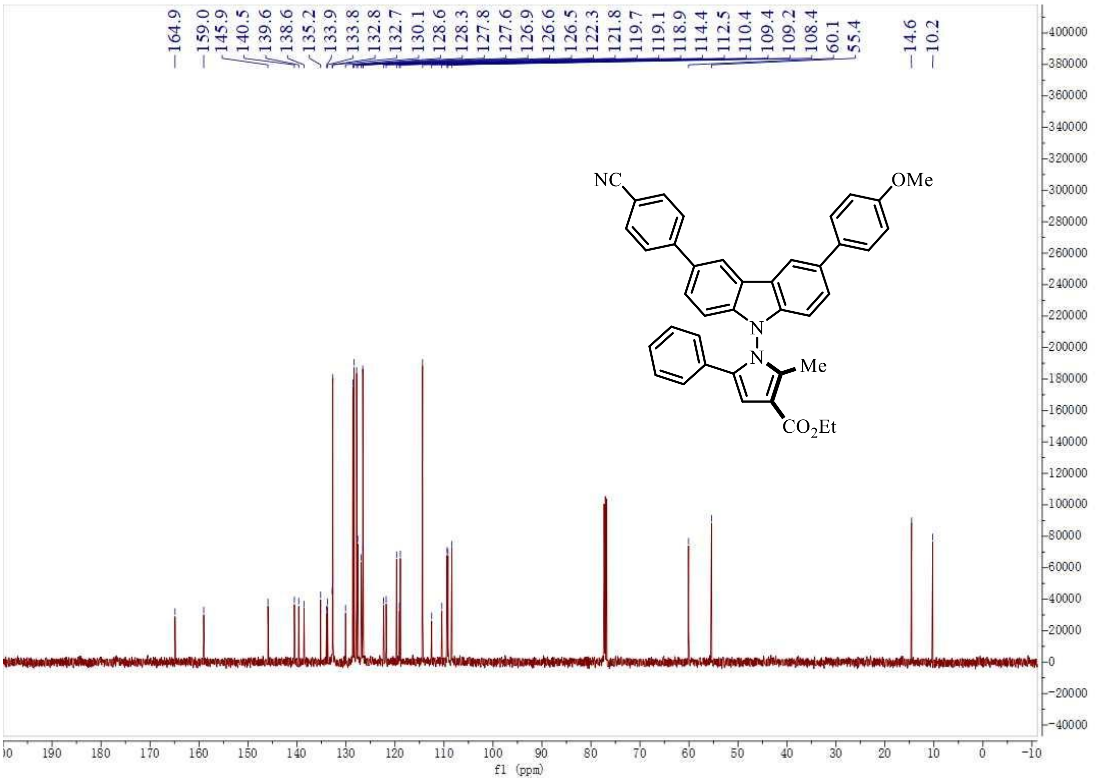

Ethyl (*S*)-3-chloro-2-(2-chloro-7-(4-(diphenylamino)phenyl)-9*H*-carbazol-9-yl)benzoate (64)

^{1}H NMR (500 MHz, Chloroform-*d*)

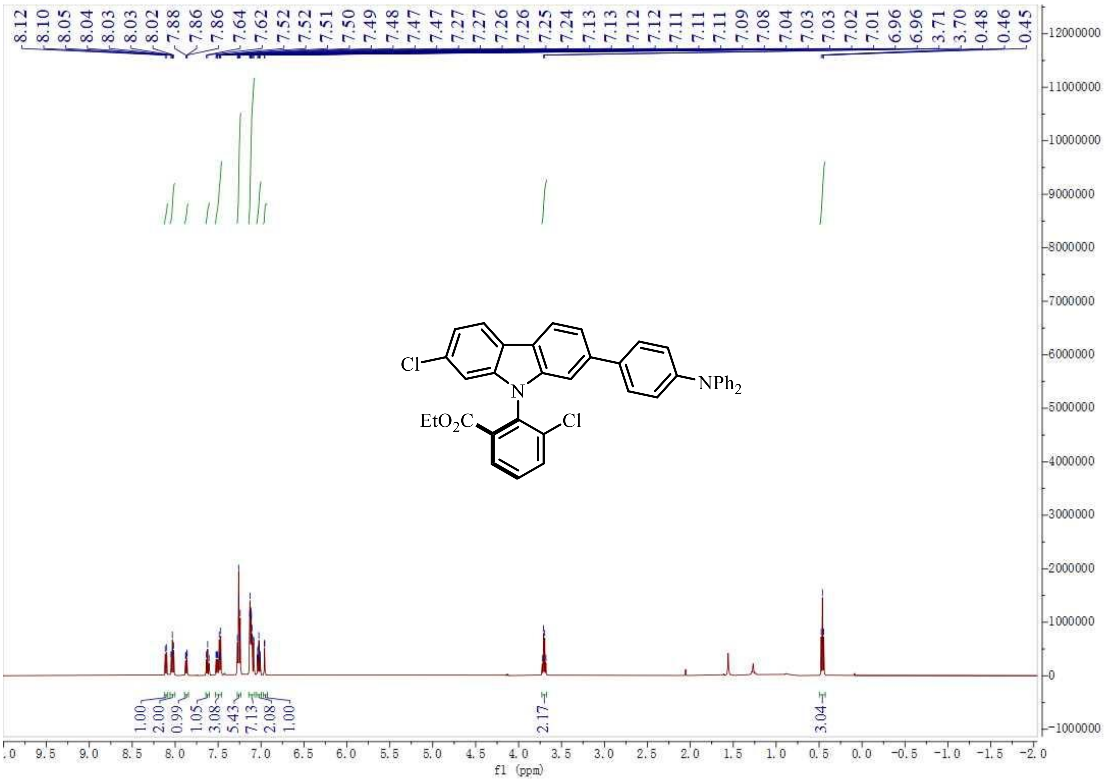

^{13}C NMR (126 MHz, Chloroform-*d*)

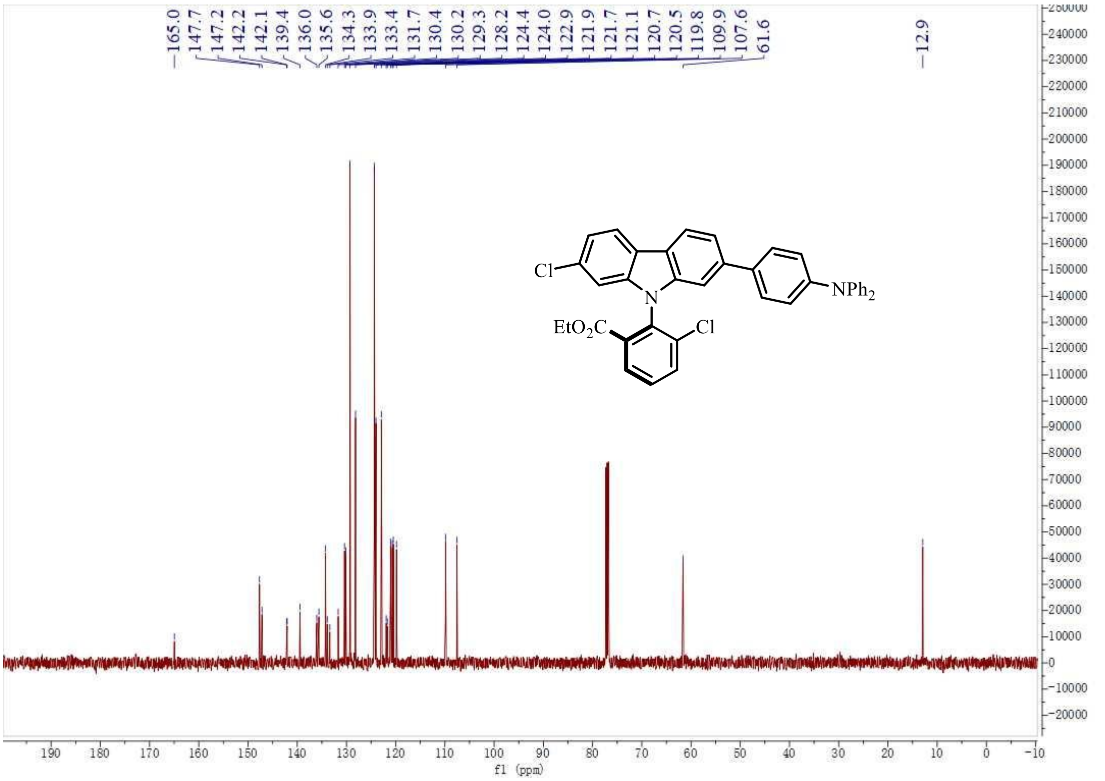

Ethyl (*R*)-2-(2-(4-(diphenylamino)phenyl)-7-(4-methoxyphenyl)-9*H*-carbazol-9-yl)-4'-methoxy-[1,1'-biphenyl]-3-carboxylate (65) ^{1}H NMR (500 MHz, Chloroform-*d*)

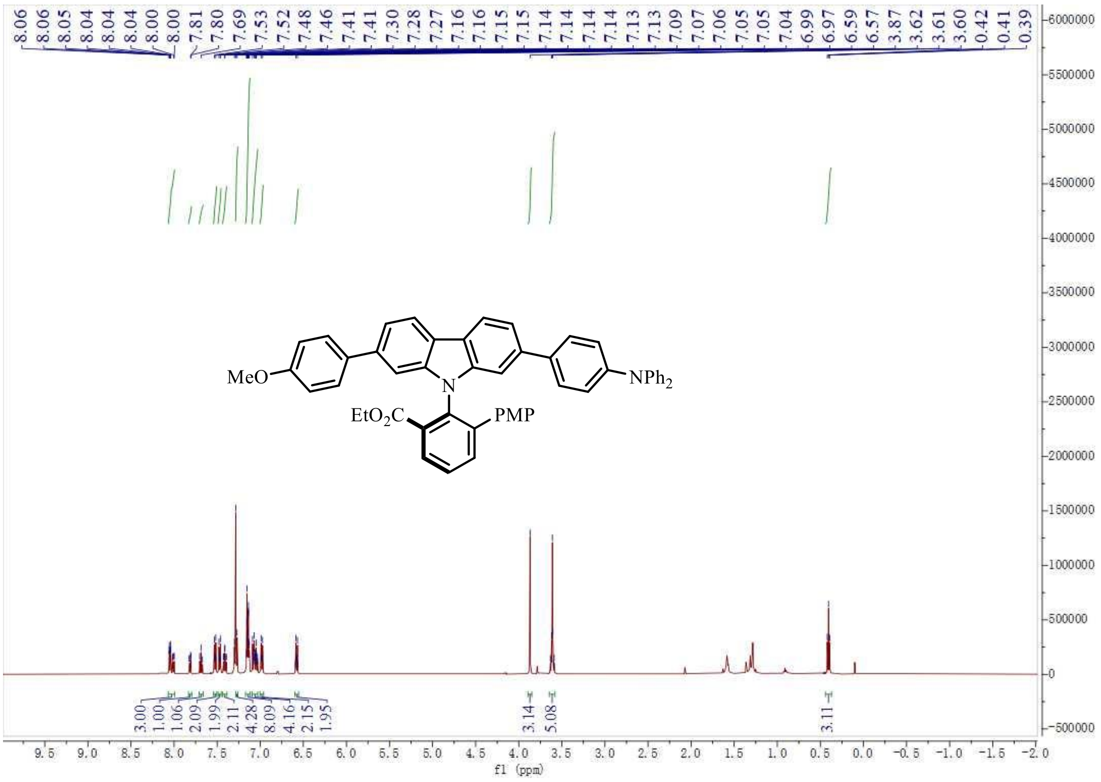

^{13}C NMR (126 MHz, Chloroform-*d*)

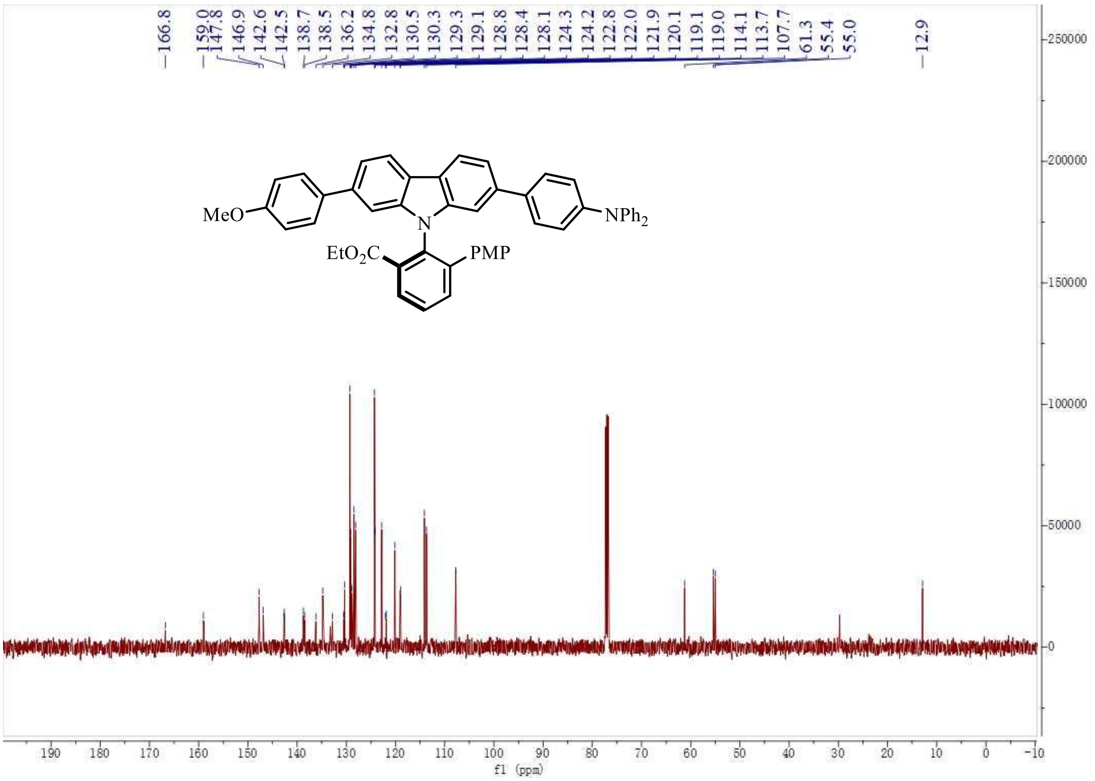

Ethyl (*R*)-3-chloro-2-(2-(4-cyanophenyl)-7-(4-(diphenylamino)phenyl)-9*H*-carbazol-9-yl)benzoate (66) ^{1}H NMR (500 MHz, Chloroform-*d*)

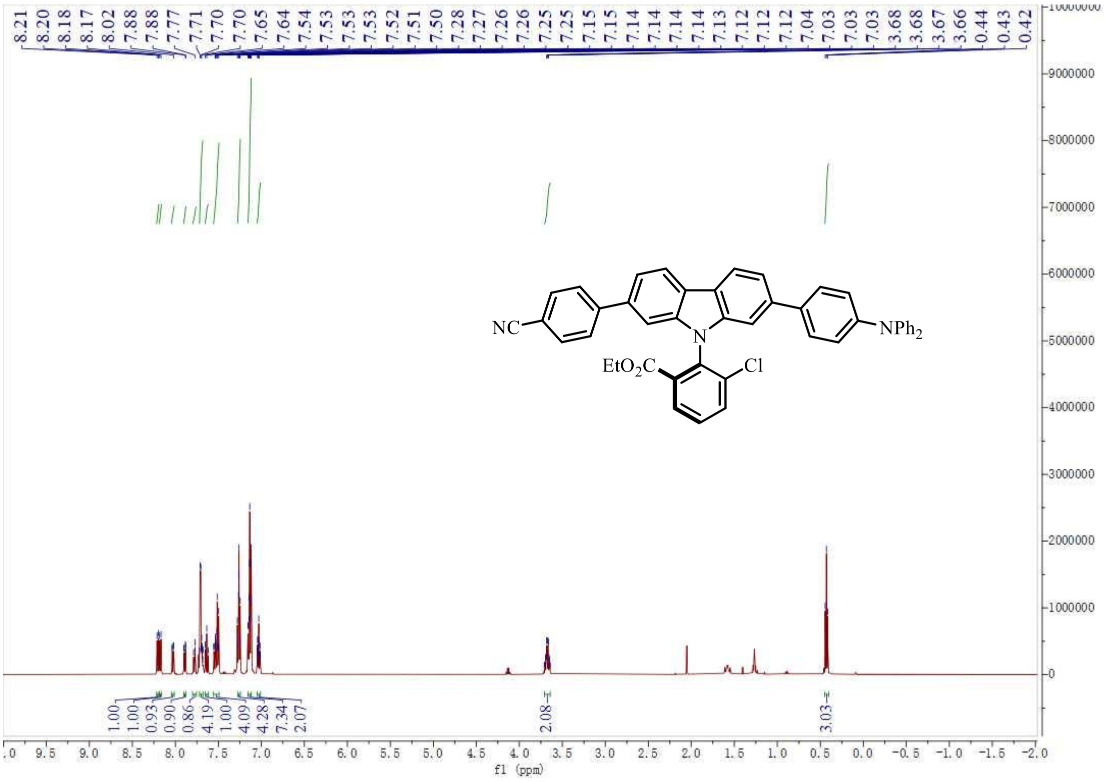

^{13}C NMR (126 MHz, Chloroform-*d*)

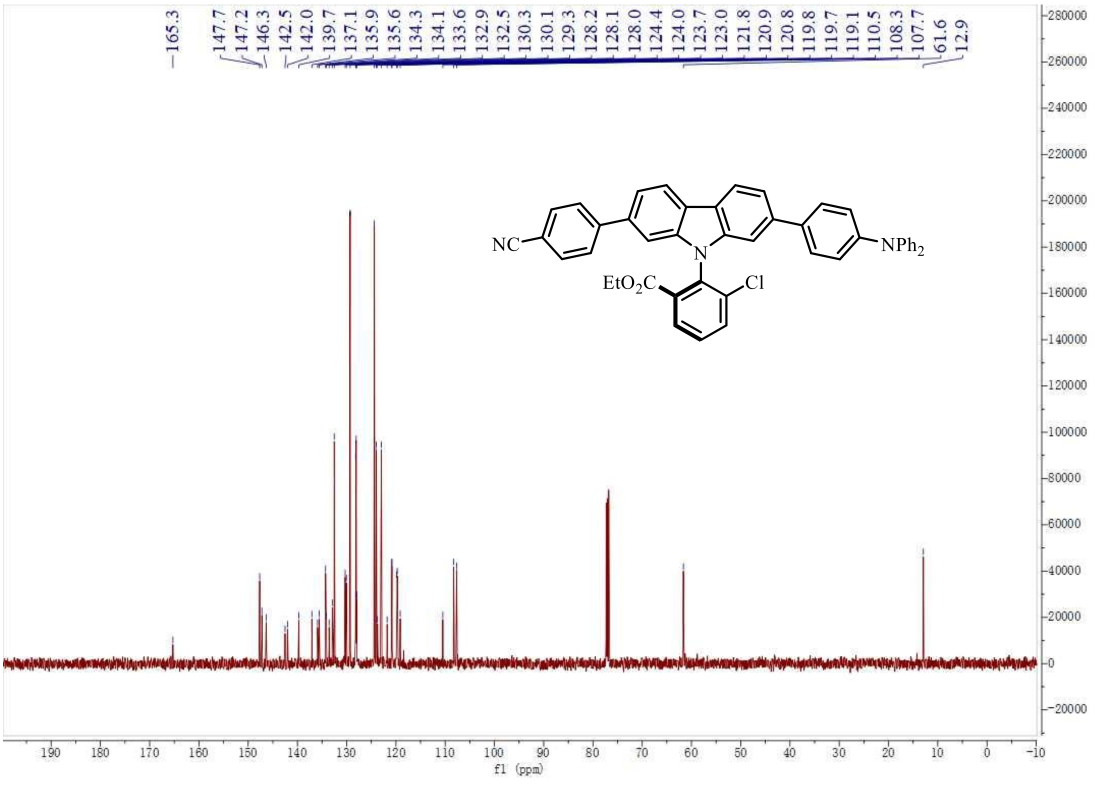

Ethyl (*S*)-2-(2-(4-cyanophenyl)-7-(4-(diphenylamino)phenyl)-9*H*-carbazol-9-yl)-4'-methoxy-[1,1'-biphenyl]-3-carboxylate (67) ^{1}H NMR (500 MHz, Chloroform-*d*)

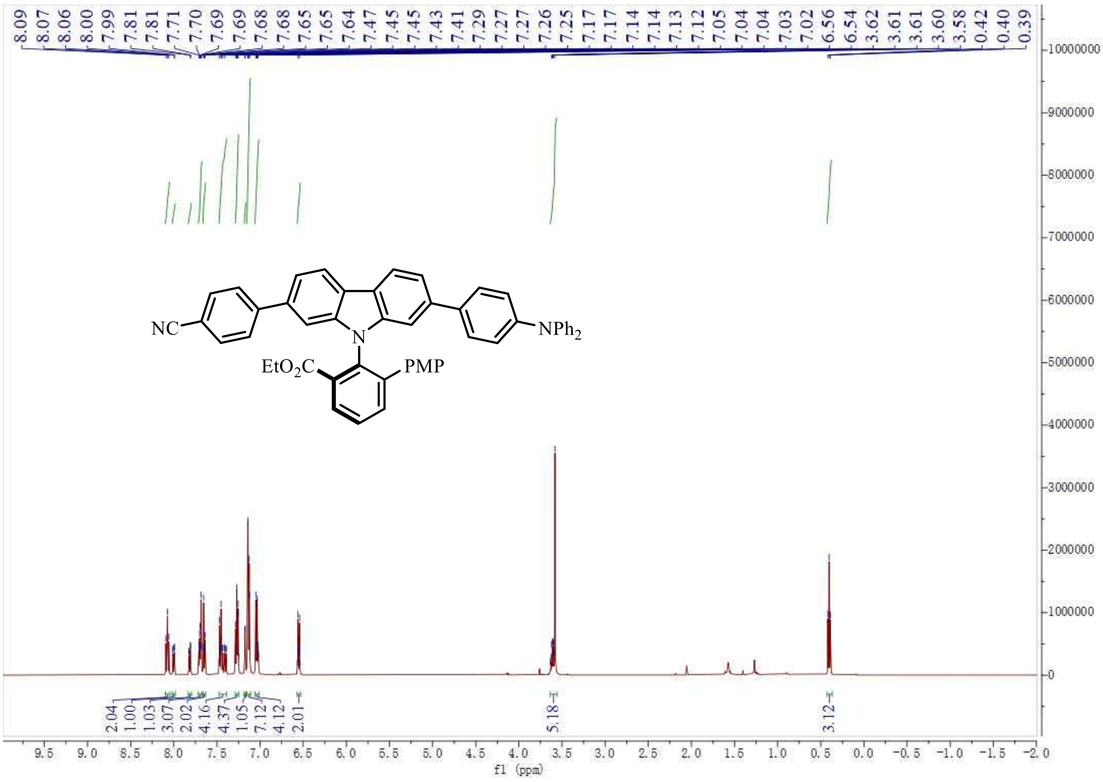

^{13}C NMR (126 MHz, Chloroform-*d*)

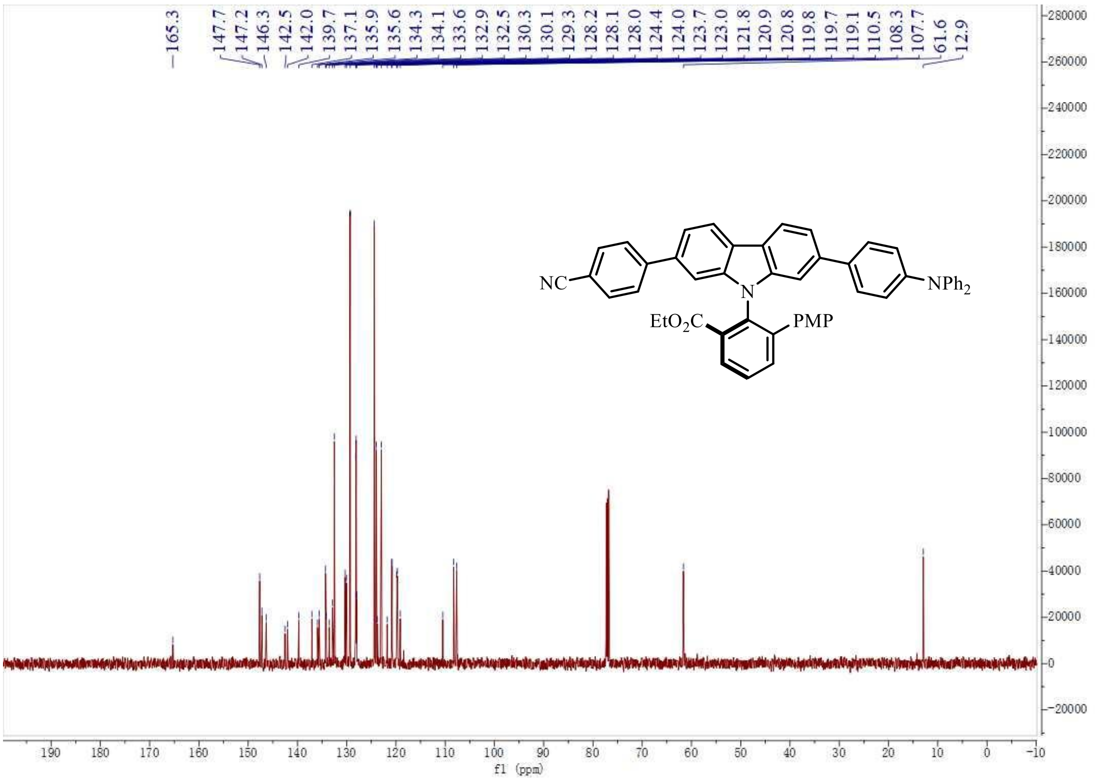

Dodecyl (*S*)-1-(2-(4-(1*H*-naphtho[1, 8-de][1, 3, 2]diazaborinin-2(3*H*)-yl)phenyl)-7-chloro-9*H*-carbazol-9-yl)-2-methyl-5-phenyl-1*H*-pyrrole-3-carboxylate (70)

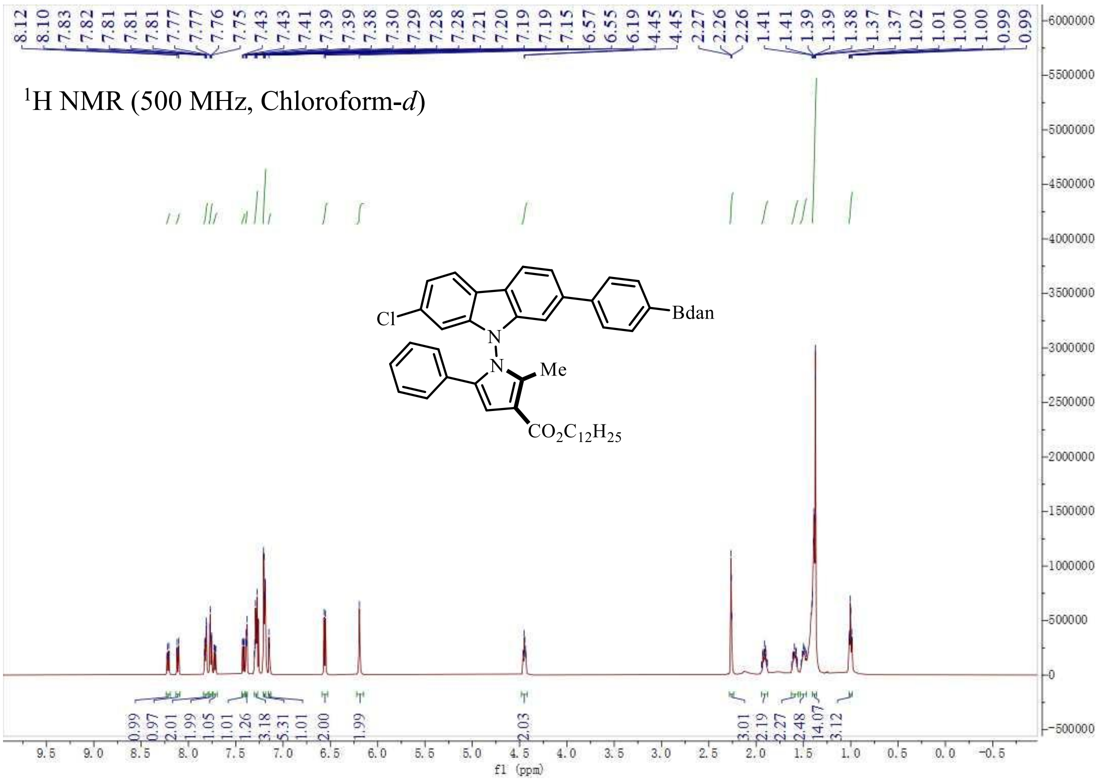

^{13}C NMR (126 MHz, Chloroform-*d*)

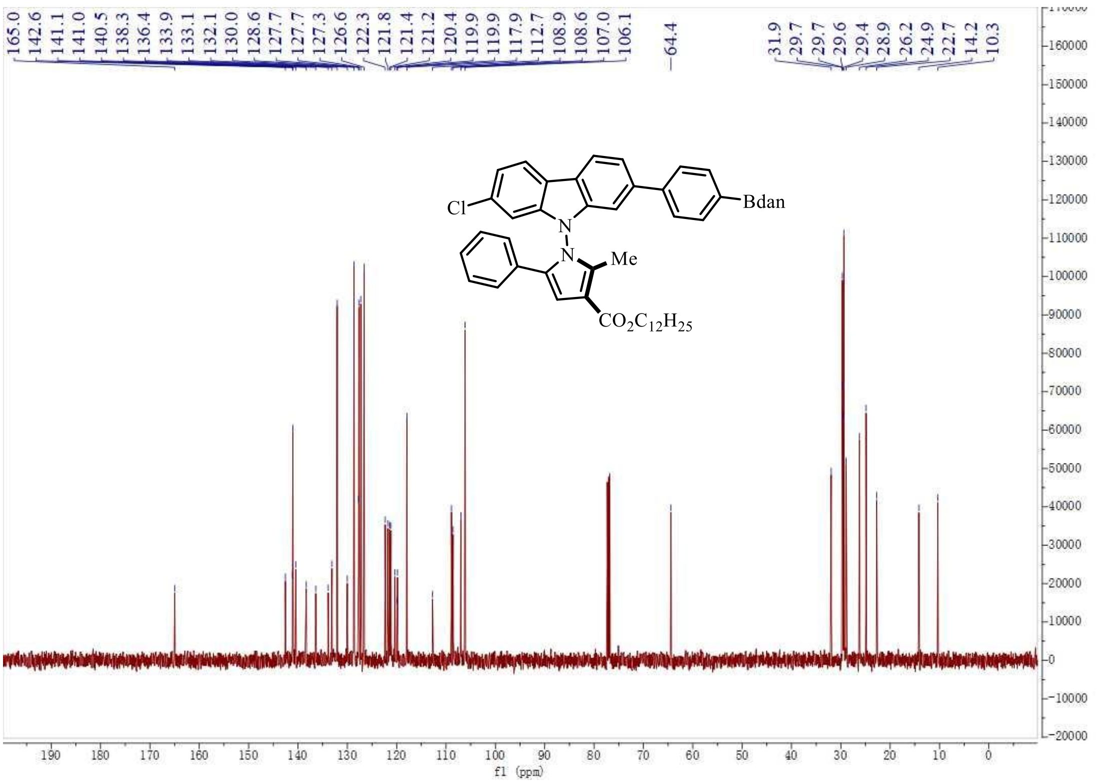

Dodecyl (*S*)-1-(2-chloro-7-(4-(4, 4, 5, 5-tetramethyl-1, 3, 2-dioxaborolan-2-yl)phenyl)-9*H*-carbazol-9-yl)-2-methyl-5-phenyl-1*H*-pyrrole-3-carboxylate (71)

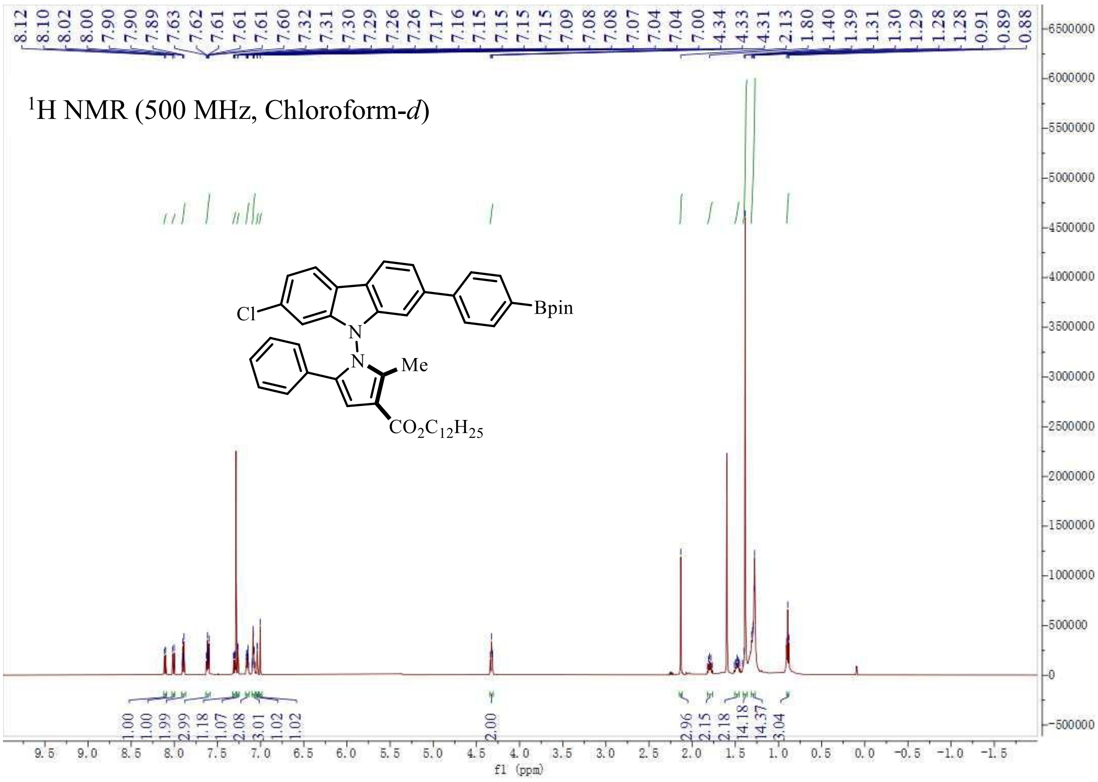

^{13}C NMR (126 MHz, Chloroform-*d*)

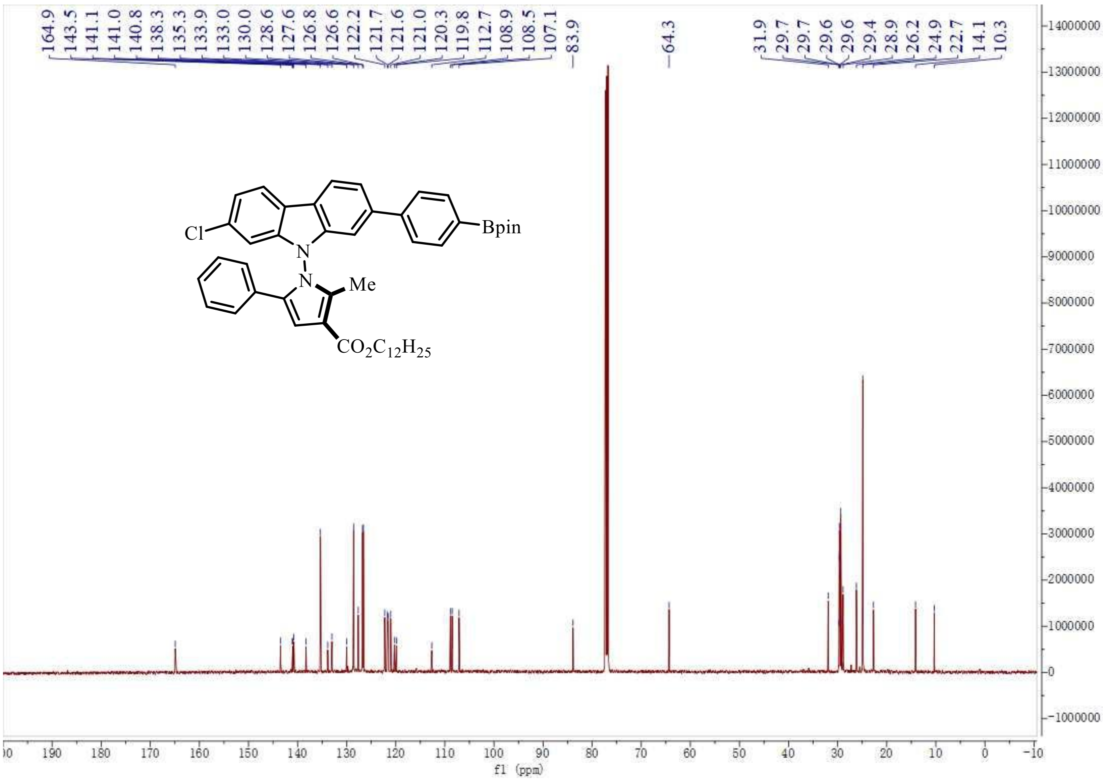

72 ^{1}H NMR (500 MHz, Chloroform-*d*)

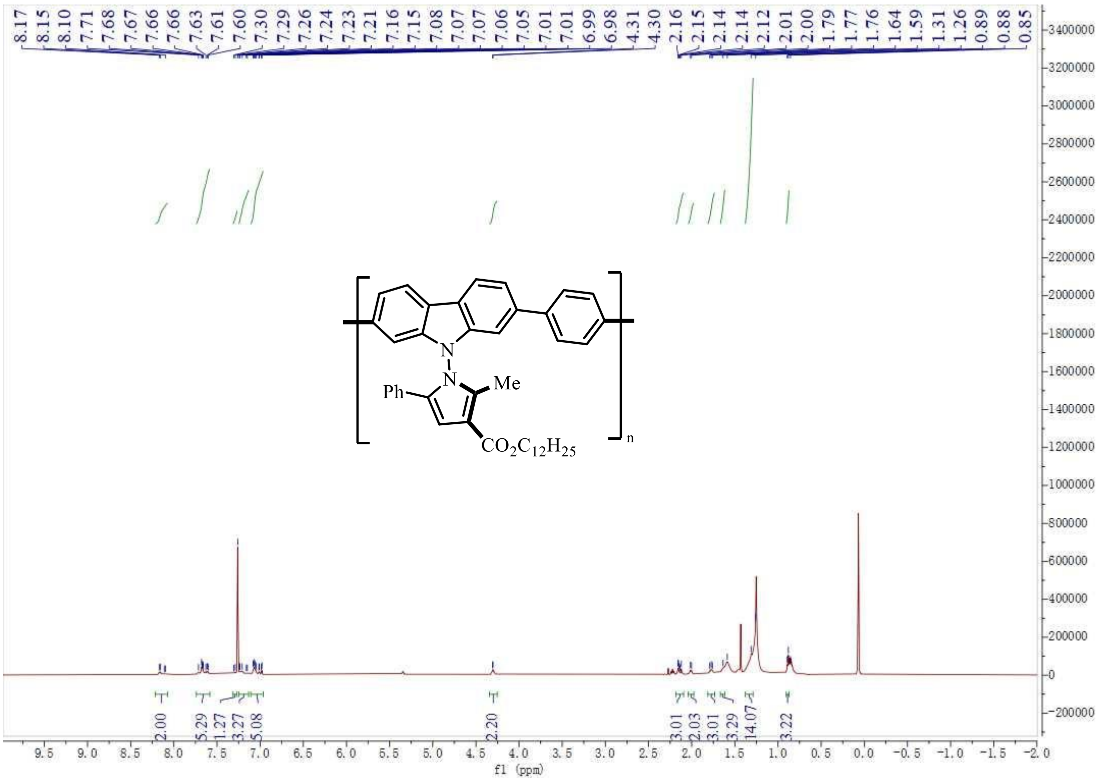

_R_-2-(2-(3-(((tert-Butoxycarbonyl)amino)-methyl)phenyl)-7-chloro-9_H_-carbazol-9-yl)-3-methylbenzoic acid (74) ^{1}H NMR (500 MHz, Chloroform-*d*)

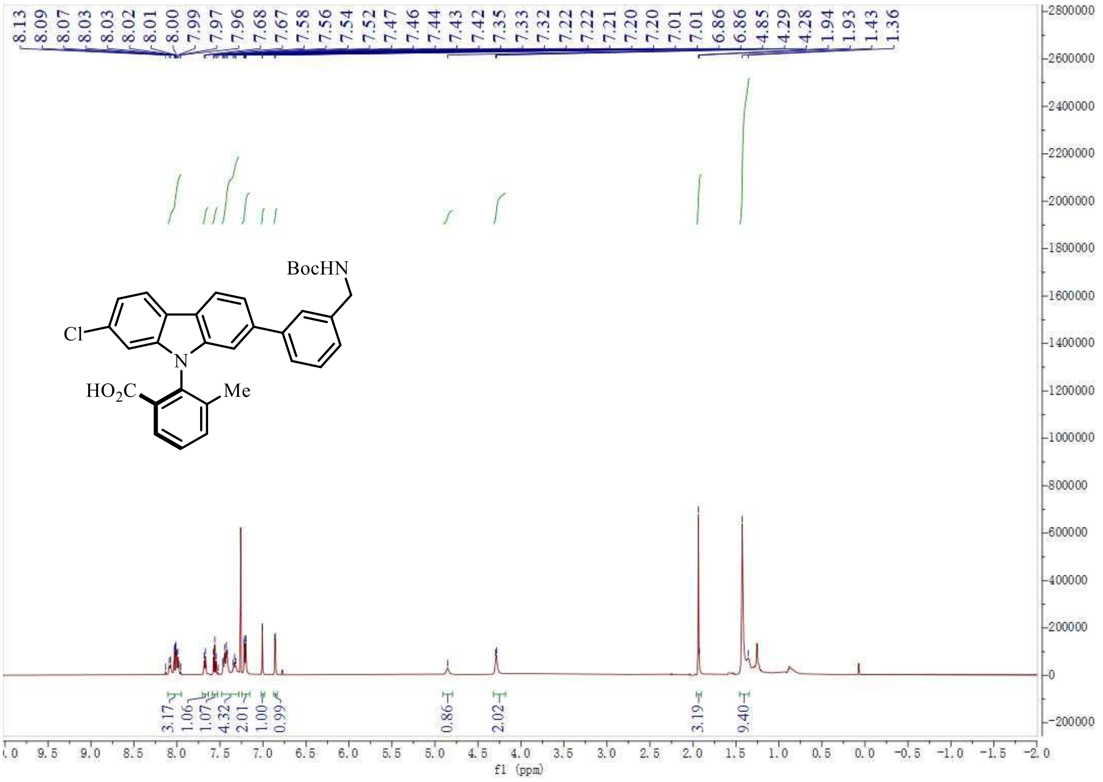

^{1}H NMR (500 MHz, Chloroform-*d*)

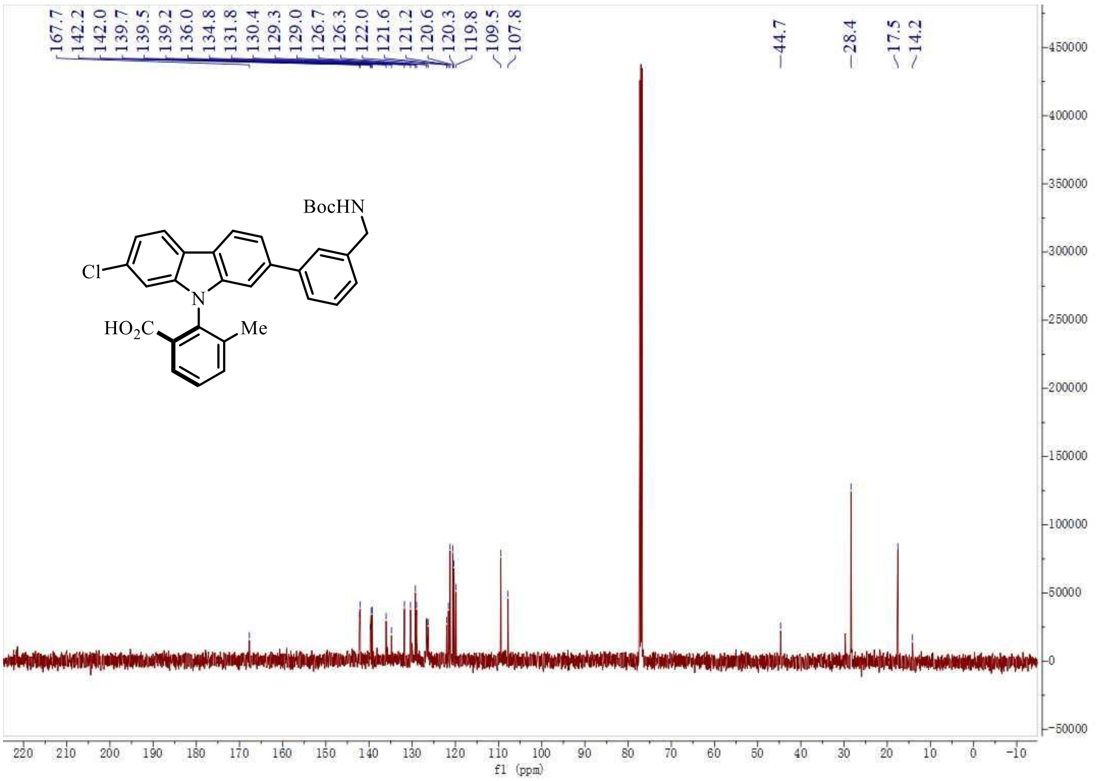

Ethyl (*R*)-2-(2-(3-(((*tert*-butoxycarbonyl)amino)methyl)phenyl)-7-chloro-9*H*-carbazol-9-yl)-3-methylbenzoate (SI-74) ^{1}H NMR (500 MHz, Chloroform-*d*)

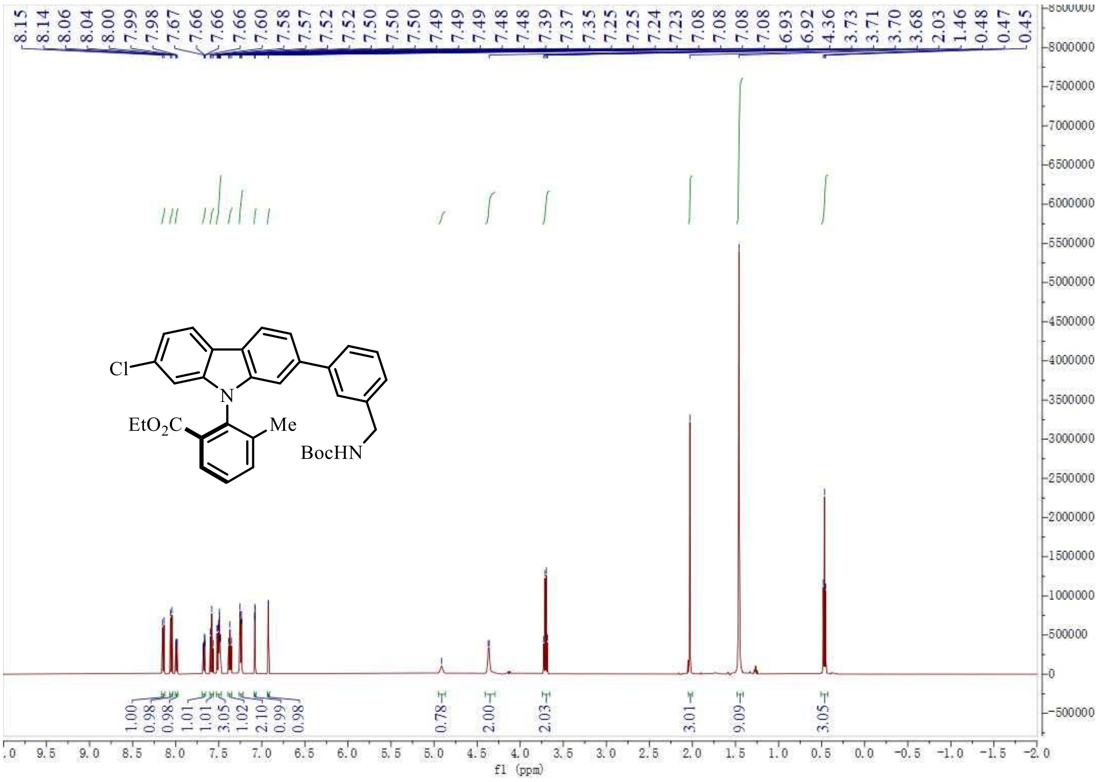

^{13}C NMR (126 MHz, Chloroform-*d*)

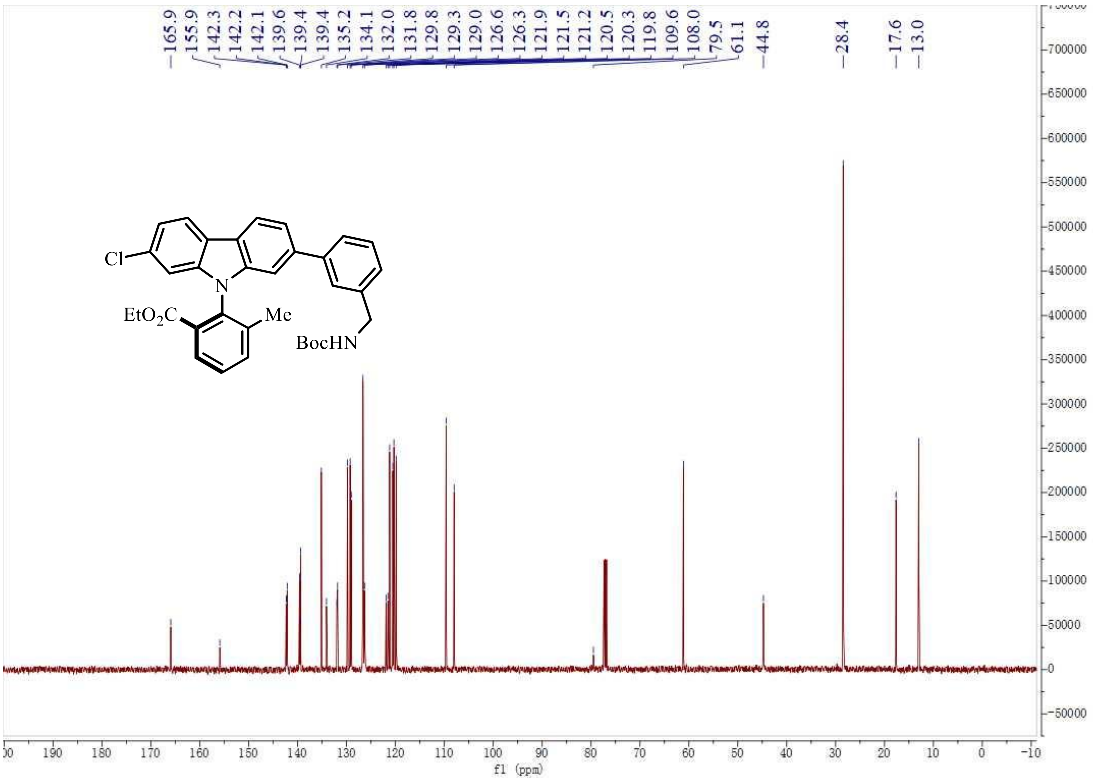

(*R*, *R*) 2^7, 8^7-Dichloro-3^6, 9^6-dimethyl-2^9H, 8^9H-5, 11-diaza-2, 8 (9, 2)-dicarbazola-1, 7 (1, 2), 3, 9 (1, 3) -tetrabenzenacyclododecaphane-4, 10-dione (75)

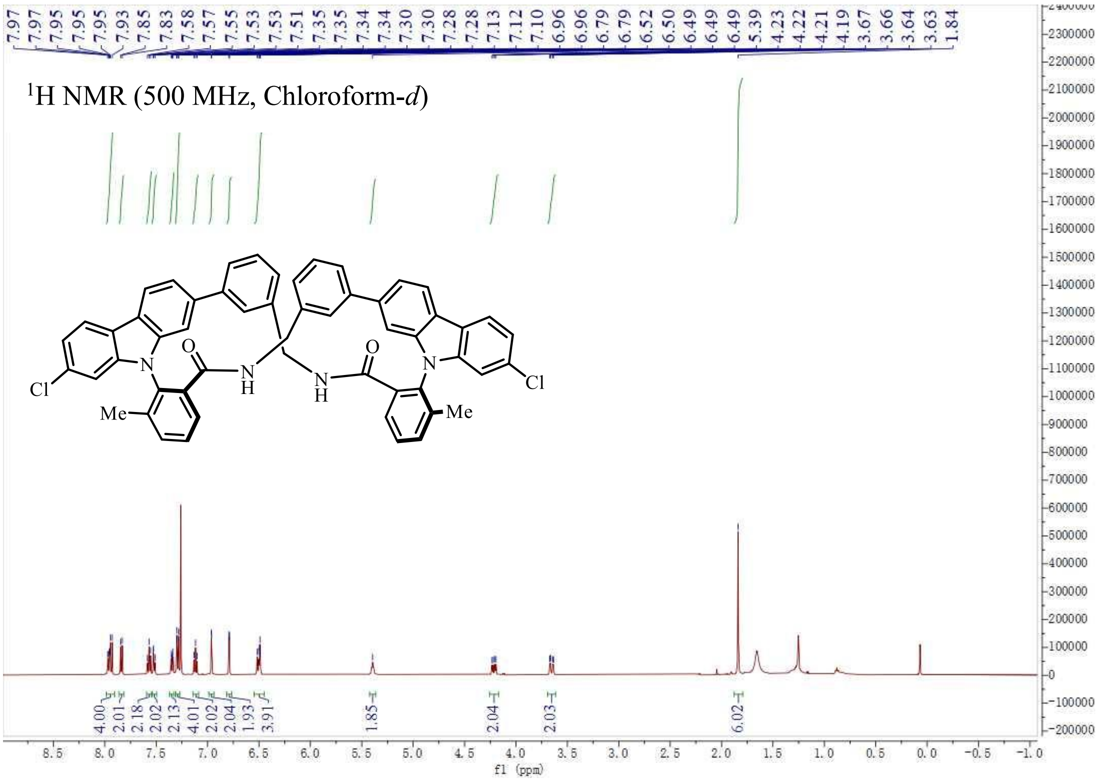

^{13}C NMR (126 MHz, Chloroform-*d*)

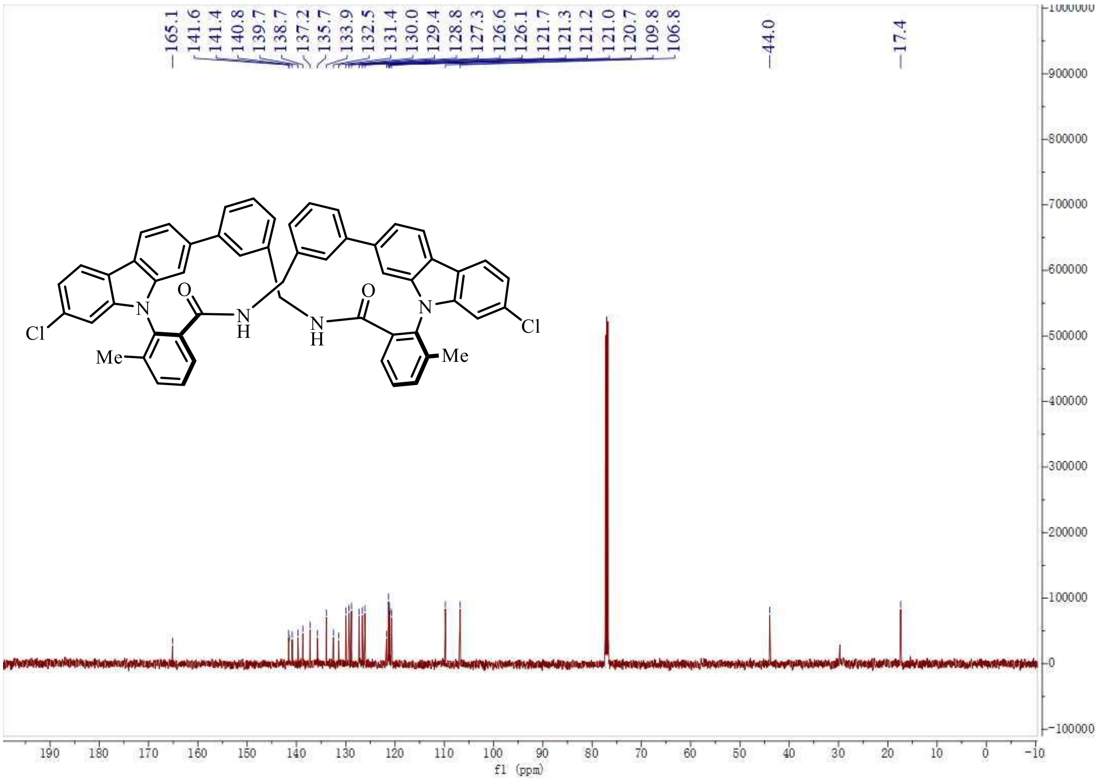

(*R*)-*tert*-Butyl (2-(2-chloro-7-(3-methoxyphenyl)-9*H*-carbazol-9-yl)-3-methylphenyl)carbama-te (76)

^{1}H NMR (500 MHz, Chloroform-*d*)

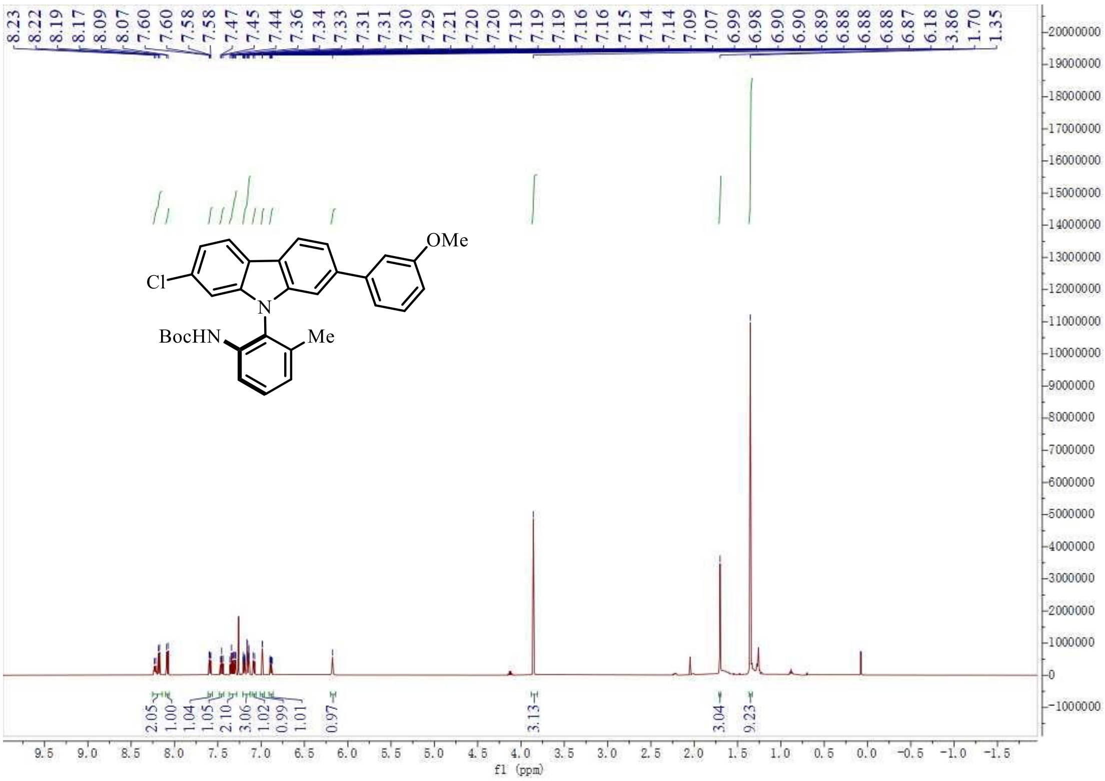

^{13}C NMR (126 MHz, Chloroform-*d*)

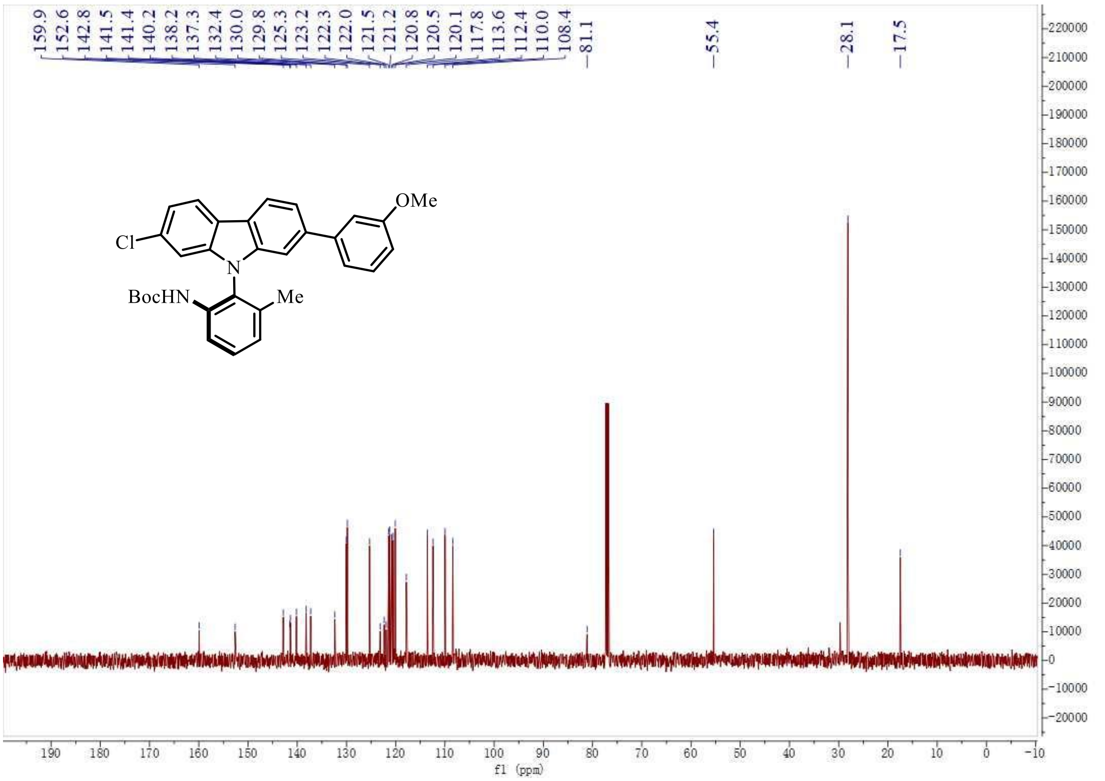

1,3-Bis(2-((*R*)-2-chloro-7-(3-methoxyphenyl)-9*H*-carbazol-9-yl)-3-methylphenyl)urea (77)

^{1}H NMR (400 MHz, Benzene-d_6)

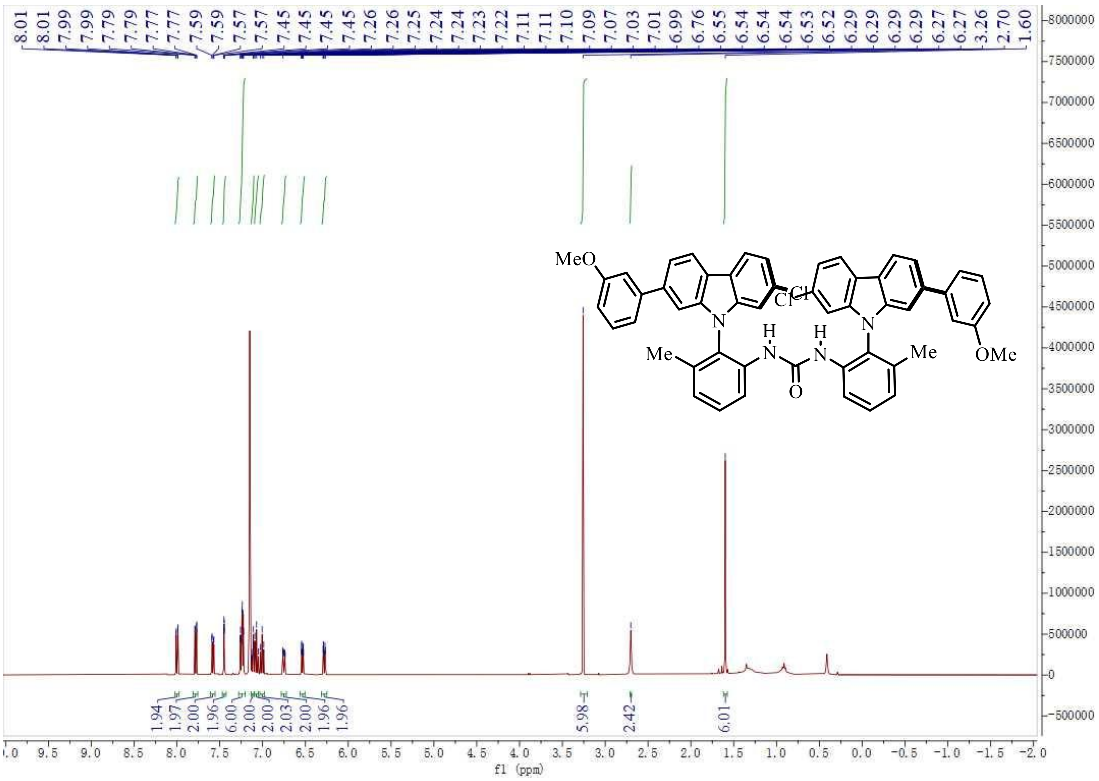

^{13}C NMR (126 MHz, Chloroform-d)

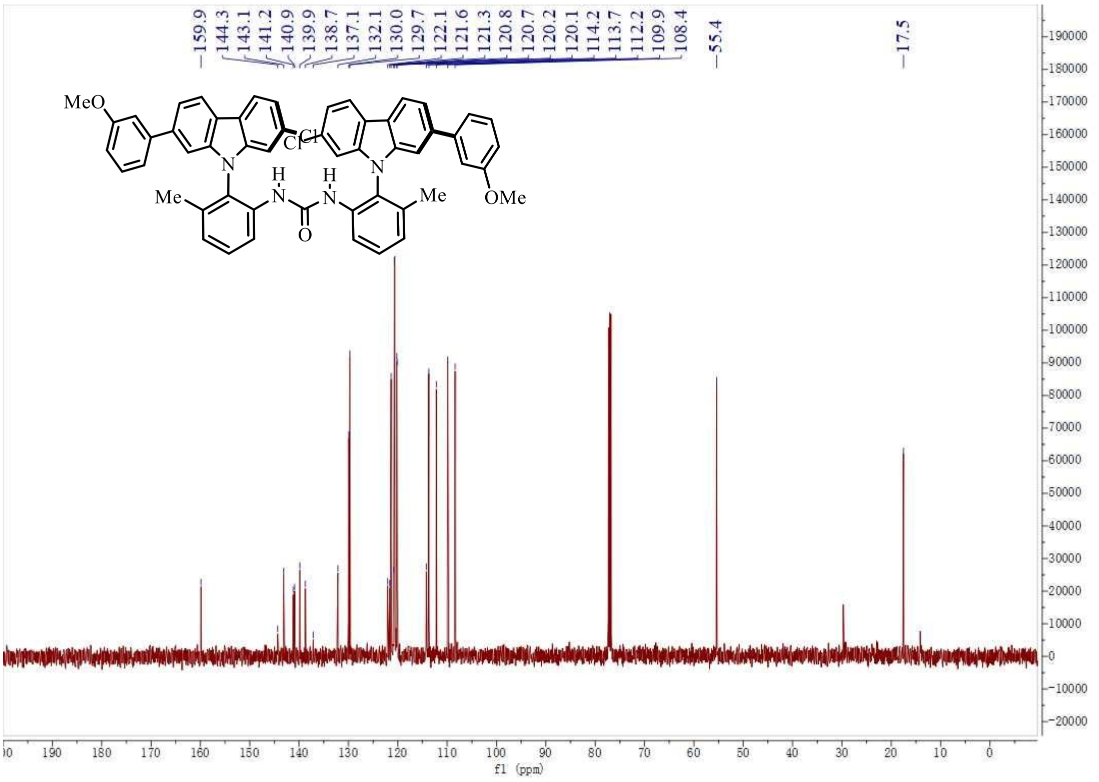

***R*-4-(7-Chloro-9-(2-(4, 6-diphenyl-1, 3, 5-triazin-2-yl)-6-methylphenyl)-9*H*-carbazol-2-yl)-*N*,*N*-diphenylaniline (78)** ^{1}H NMR (500 MHz, Chloroform-*d*)

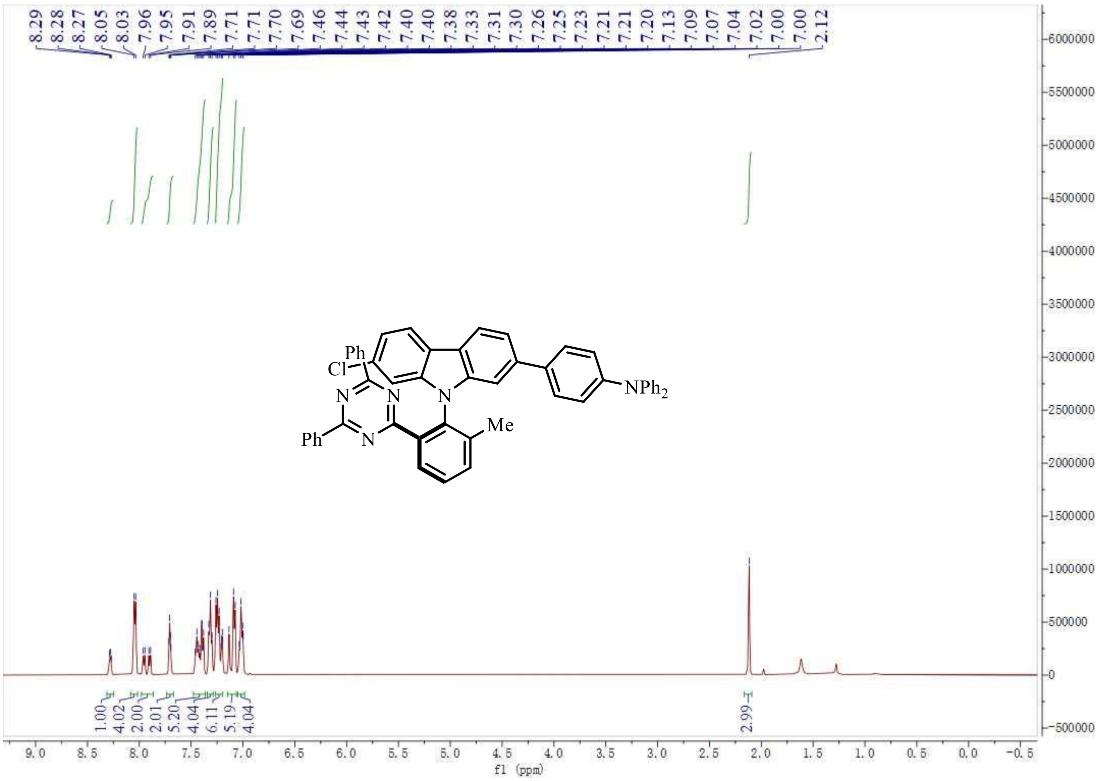

^{13}C NMR (126 MHz, Chloroform-*d*)

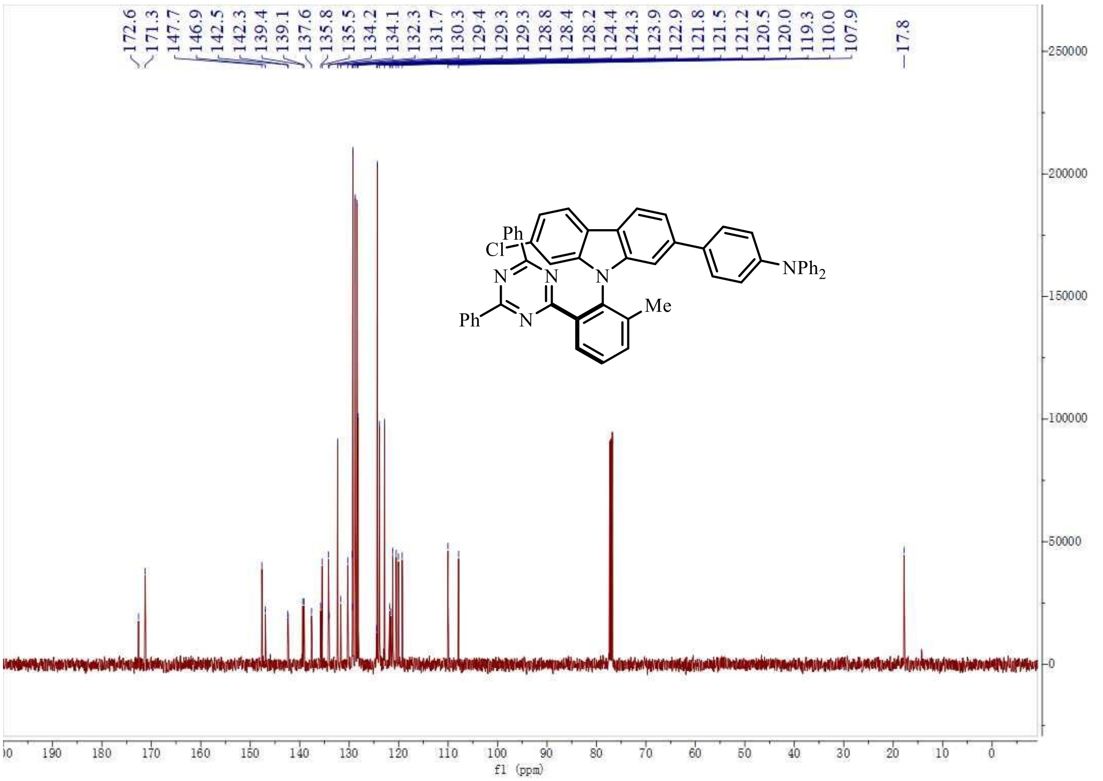

***S*-4-(9-(2-(4, 6-Ciphenyl-1, 3, 5-triazin-2-yl)-6-methylphenyl)-9*H*-carbazol-2-yl)-*N*, *N*-diphenyl -aniline (79)** ^{1}H NMR (500 MHz, Chloroform-*d*)

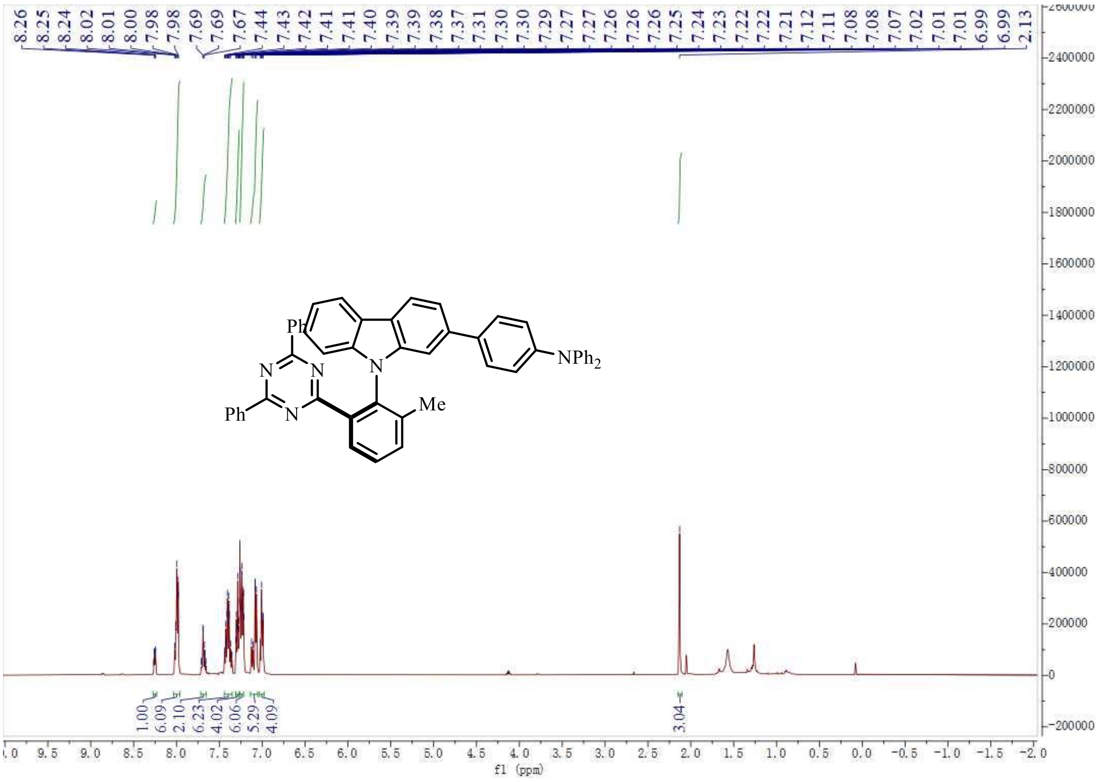

[13]H NMR (126 MHz, Chloroform-*d*)

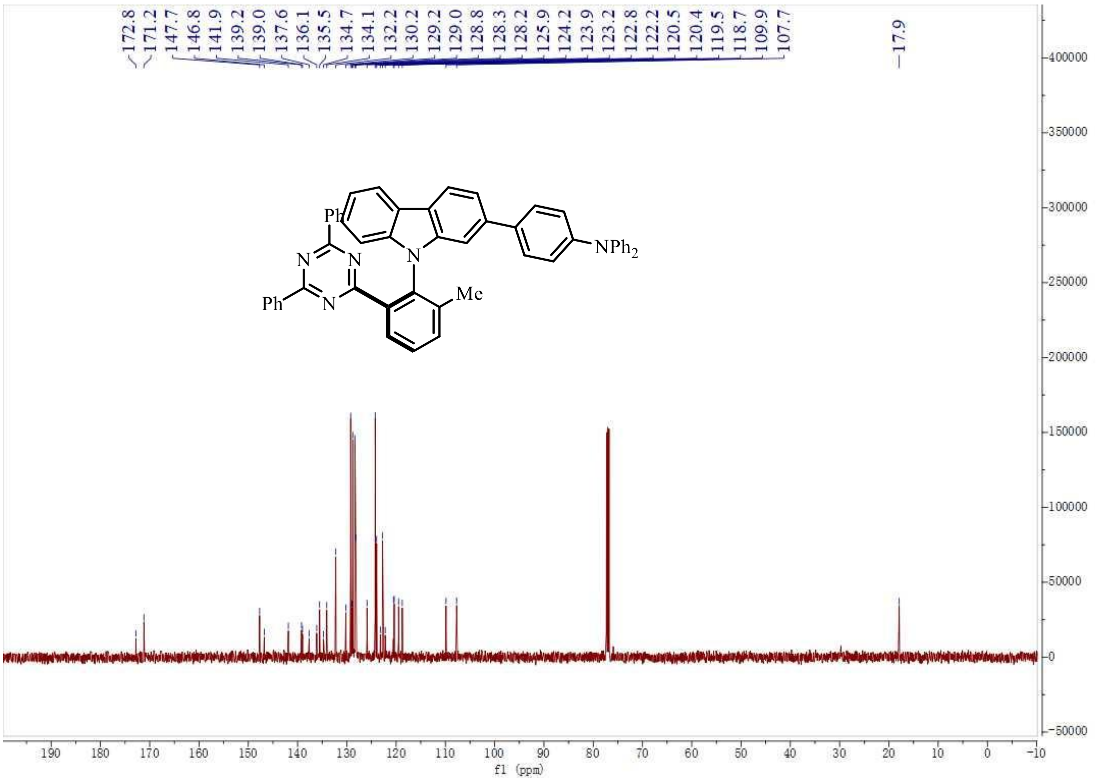

Ethyl (*S*)-2-(2-bromo-7-(4-methoxyphenyl)-9*H*-carbazol-9-yl)-3-methylbenzoate (81)

^{1}H NMR (600 MHz, Chloroform-*d*)

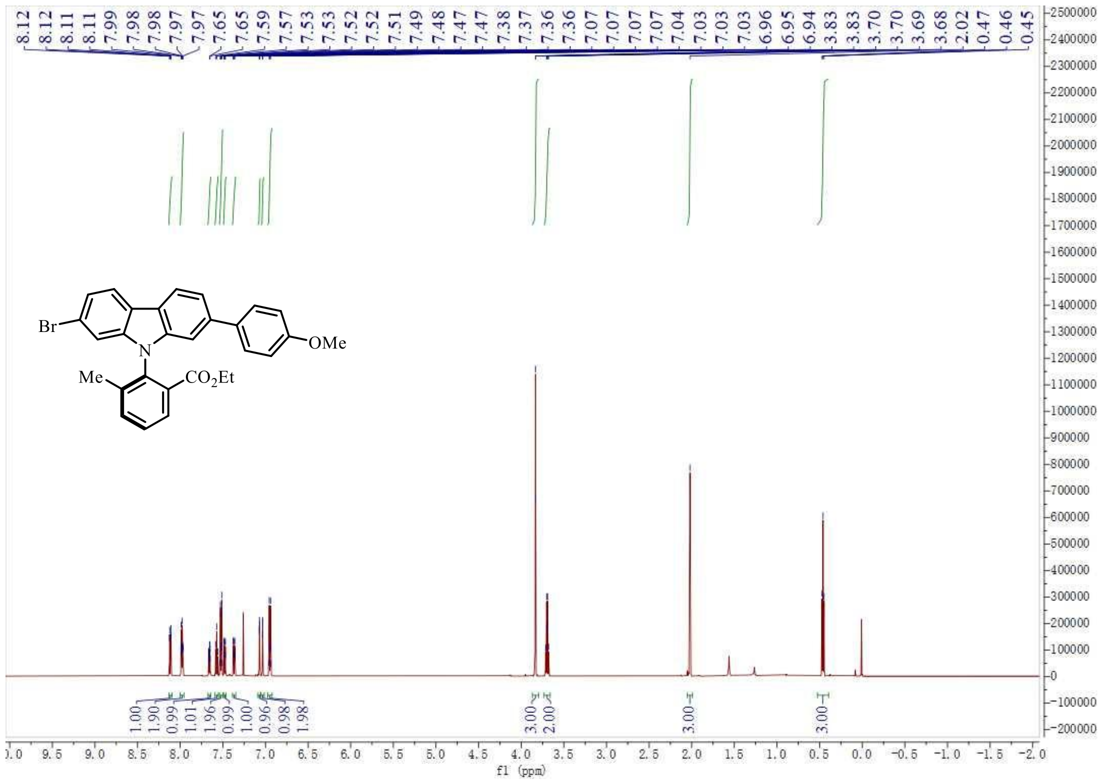

^{13}C NMR (151 MHz, Chloroform-*d*)

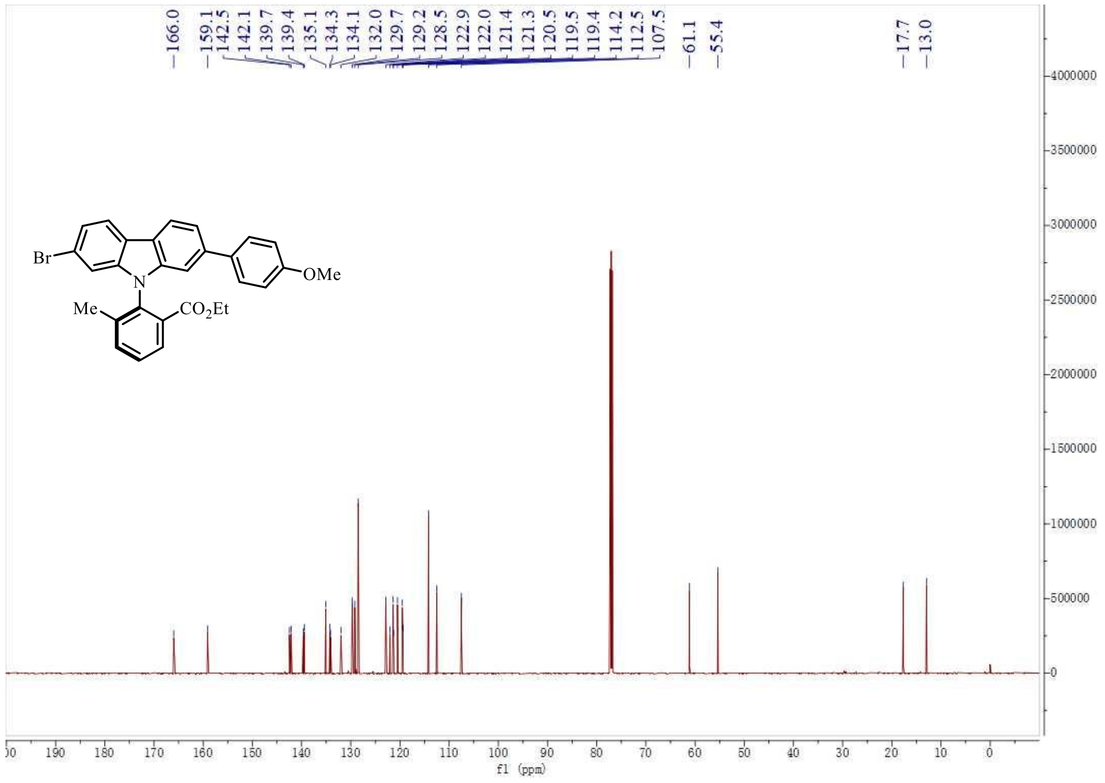

Ethyl 2-(2,7-bis(4-methoxyphenyl)-9*H*-carbazol-9-yl)-3-methylbenzoate (Bis-81)

^{1}H NMR (500 MHz, Chloroform-*d*)

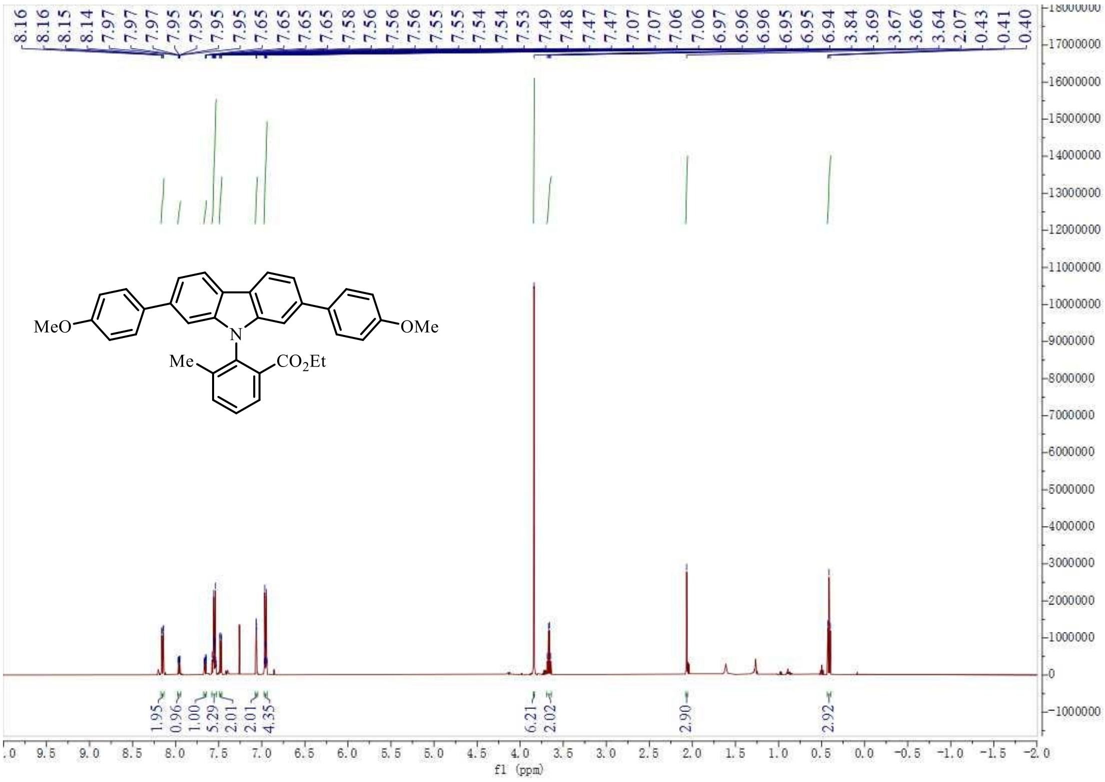

^{13}C NMR (126 MHz, Chloroform-*d*)

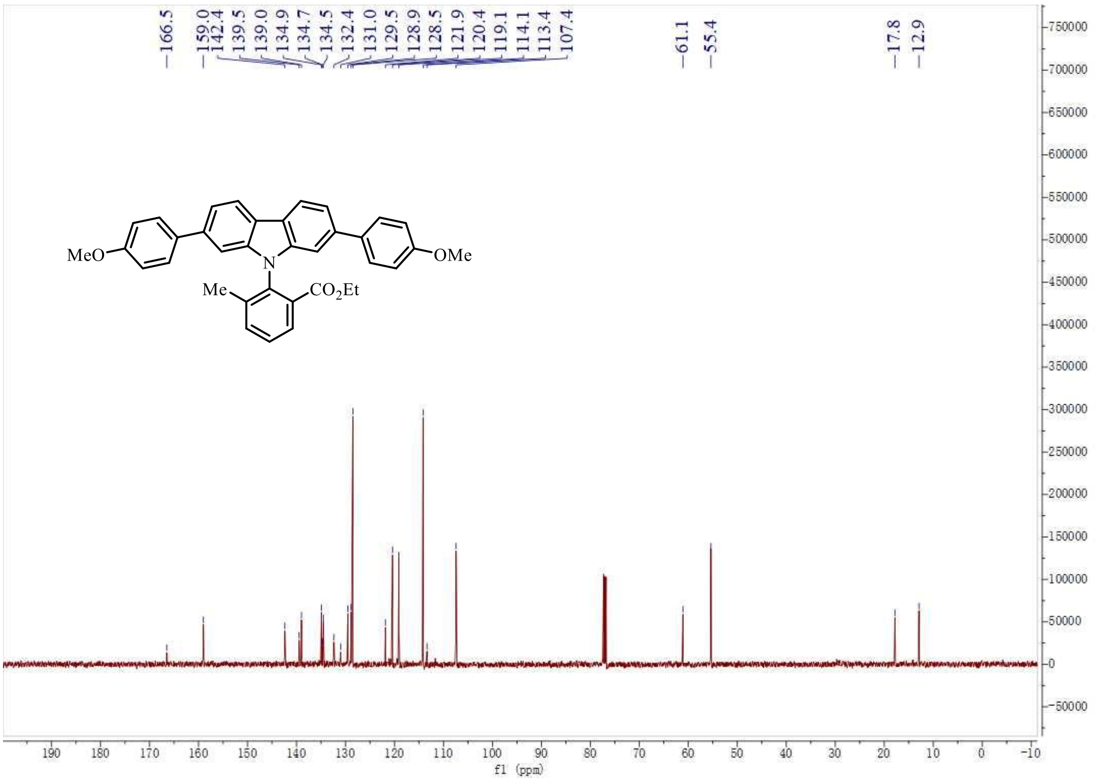

Ethyl (*S*)-2-(2-(dibenzo[b,d]thiophen-4-yl)-7-(4-methoxyphenyl)-9*H*-carbazol-9-yl)-3-methylbenzoate (83) ^{1}H NMR (600 MHz, Chloroform-*d*)

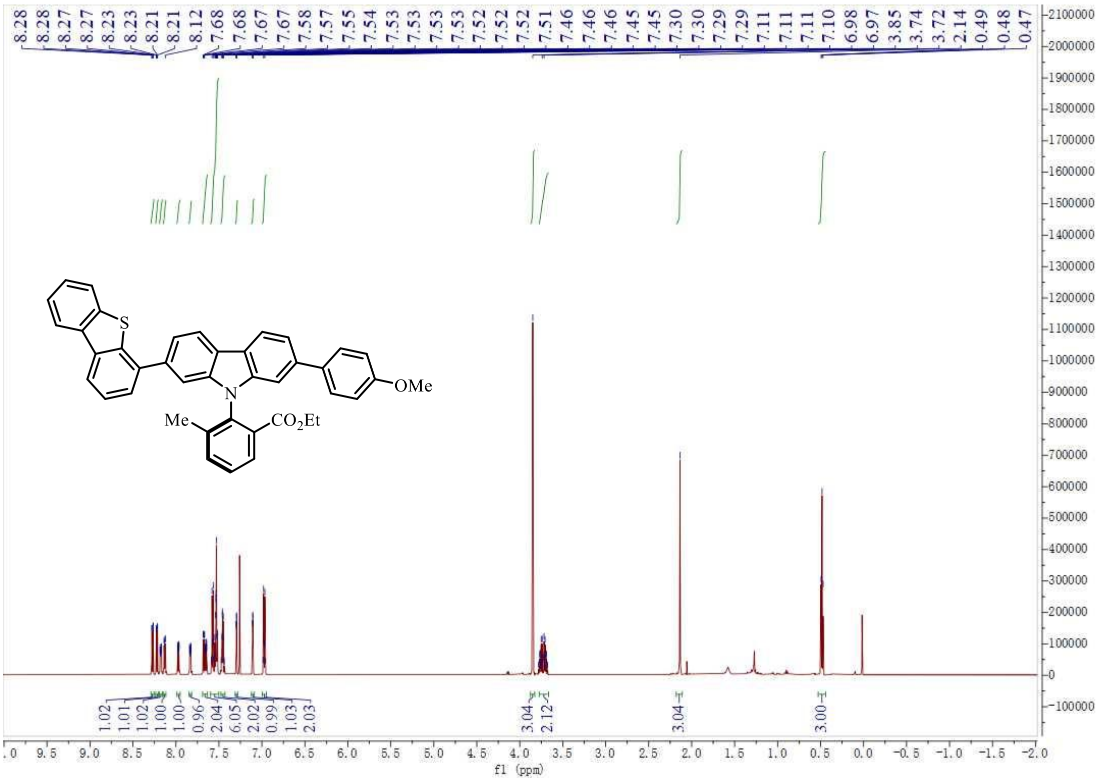

^{13}C NMR (126 MHz, Chloroform-*d*)

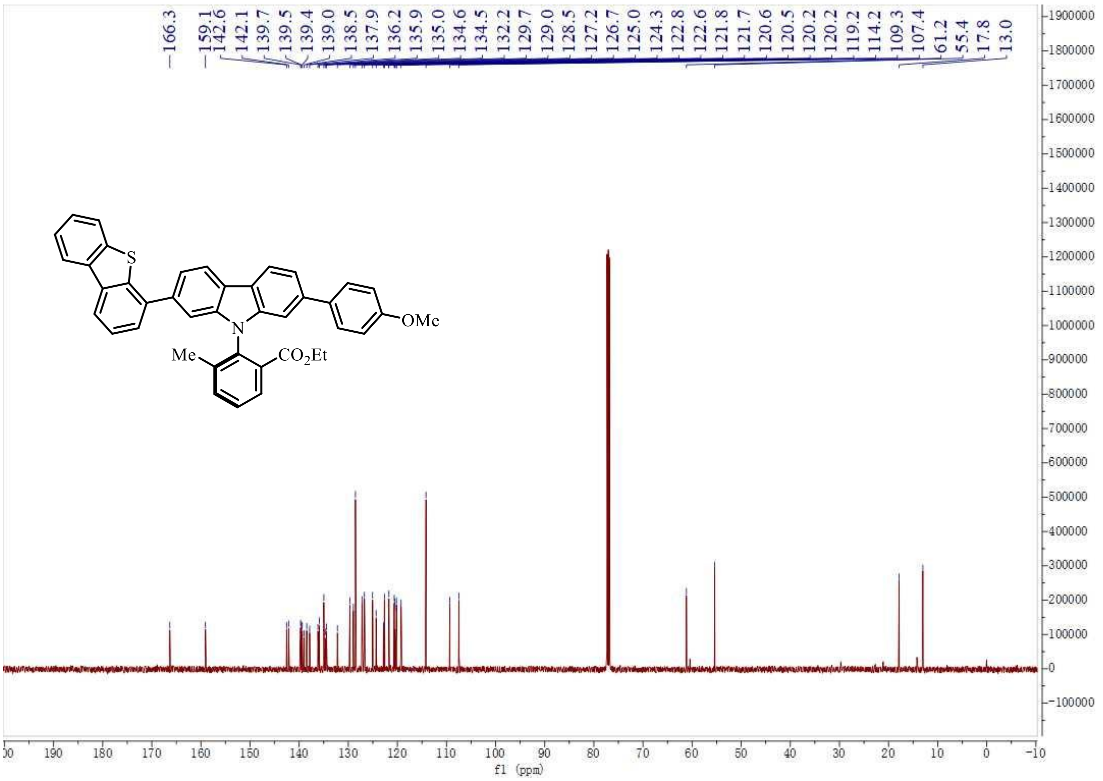

Ethyl (*S*)-2-(3-chloro-6-(4-methoxyphenyl)-9*H*-carbazol-9-yl)-3-methylbenzoate (SI-10)

^{1}H NMR (500 MHz, Chloroform-*d*)

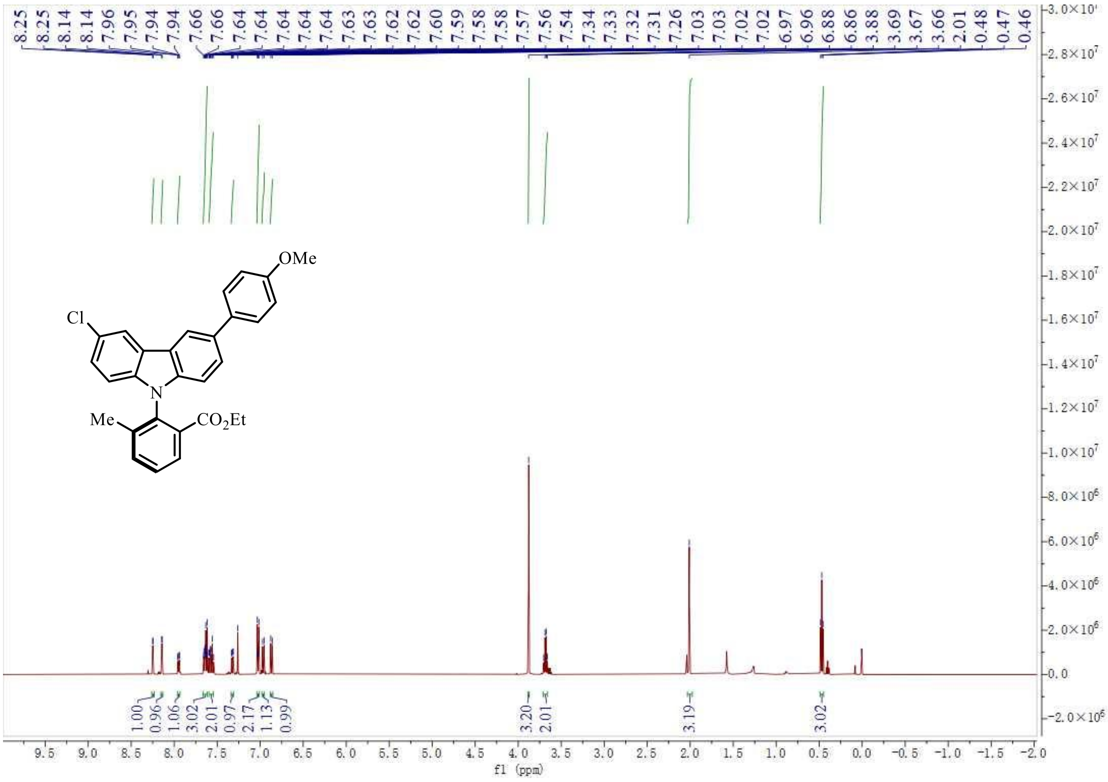

^{13}C NMR (126 MHz, Chloroform-*d*)

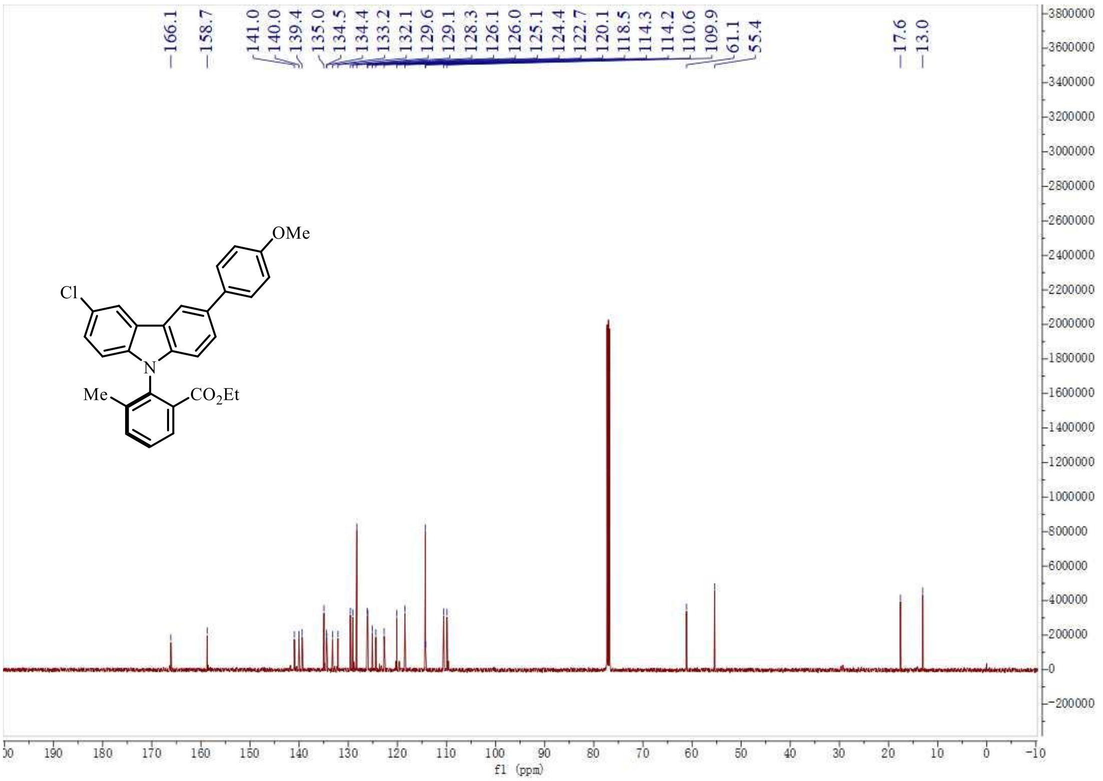

HPLC Traces

2

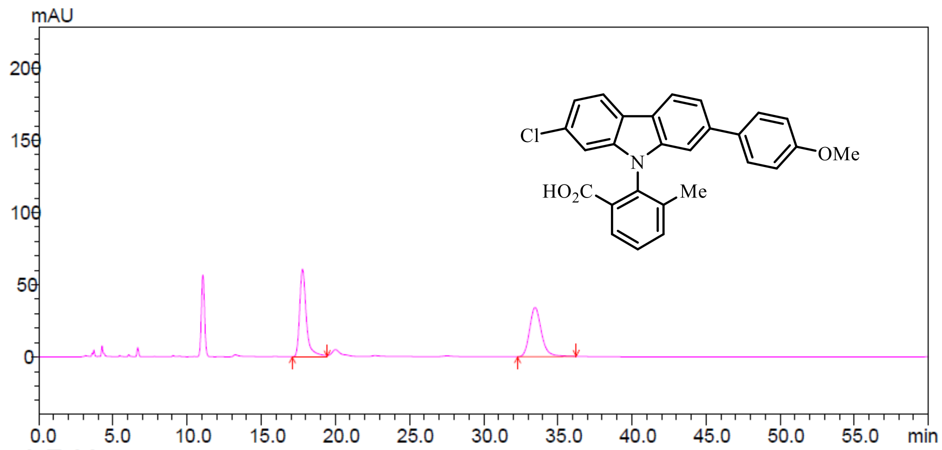

<Peak Table>

PDA Ch1 315nm

Peak#	Ret. Time	Area	Height	Peak Start	Peak End	Area%
1	17.787	1927123	60658	17.088	19.445	50.484
2	33.468	1890163	34063	32.288	36.235	49.516
Total		3817286	94721			100.000

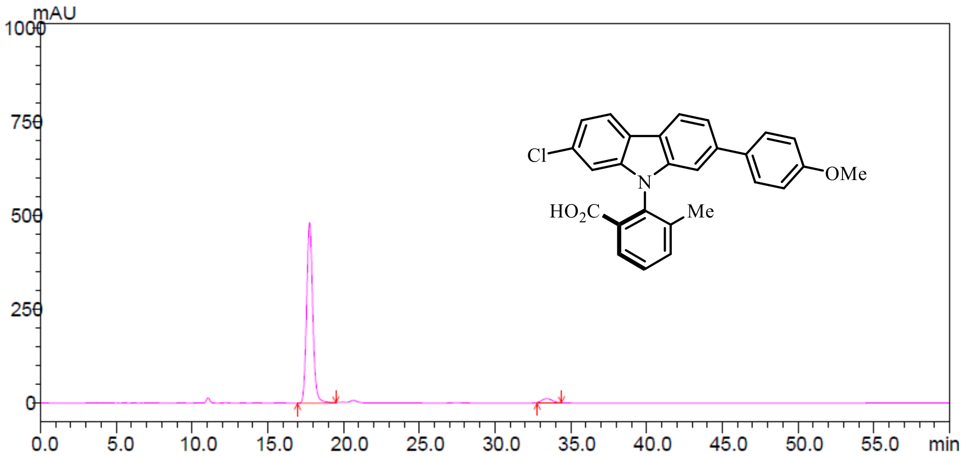

<Peak Table>

PDA Ch1 315nm

Peak#	Ret. Time	Area	Height	Peak Start	Peak End	Area%
1	17.762	13682453	481823	16.960	19.499	95.925
2	33.415	581264	11518	32.768	34.379	4.075
Total		14263717	493341			100.000

Et-ester of 2

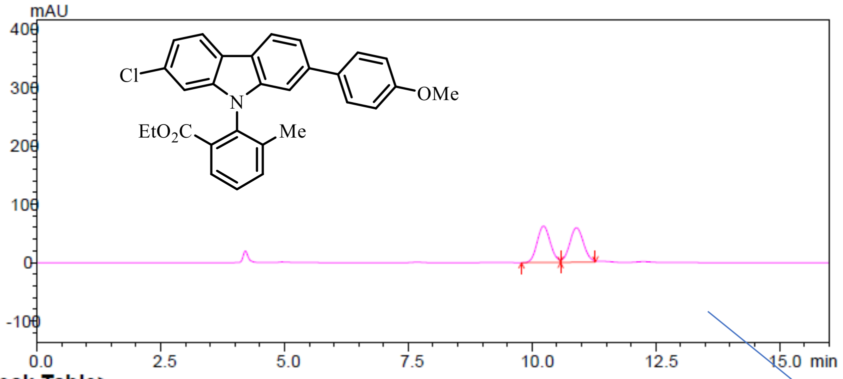

<Peak Table>

PDA Ch1 315nm

Peak#	Ret. Time	Area	Height	Peak Start	Peak End	Area%
1	10.232	1149971	62252	9.787	10.576	49.988
2	10.899	1150517	59274	10.576	11.280	50.012
Total		2300488	121526			100.000

Daicel Chiralpak IC, hexane/*iso*-propanol = 99:1, 0.8 mL/min,

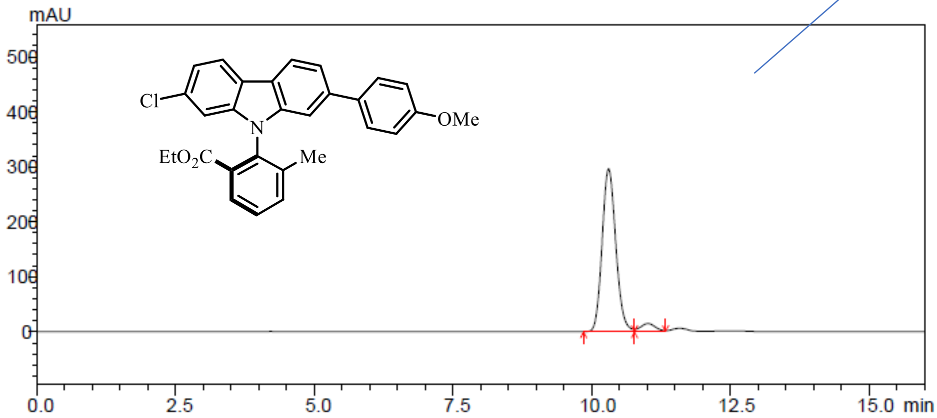

<Peak Table>

PDA Ch1 315nm

Peak#	Ret. Time	Area	Height	Peak Start	Peak End	Area%
1	10.302	5108819	296537	9.867	10.768	96.157
2	11.010	204178	12509	10.768	11.333	3.843
Total		5312997	309046			100.000

3

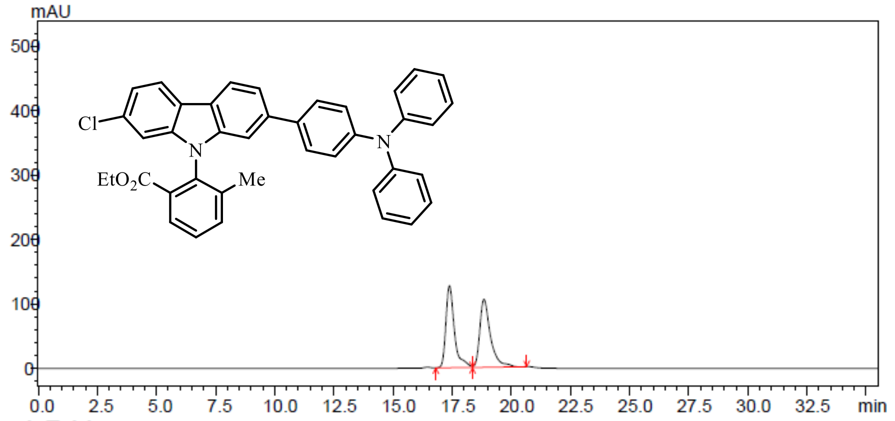

<Peak Table>

PDA Ch1 348nm

Peak#	Ret. Time	Area	Peak Start	Peak End	Area%
1	17.385	3706989	16.661	18.368	49.655
2	18.845	3758488	18.368	20.288	50.345
Total		7465477			100.000

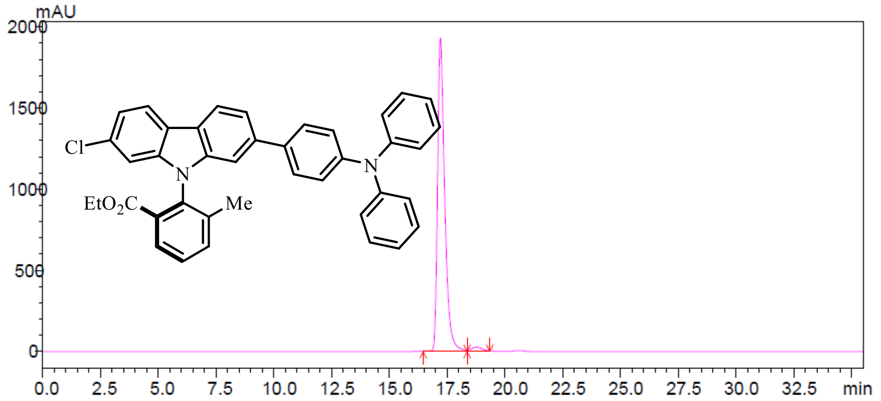

<Peak Table>

PDA Ch1 348nm

Peak#	Ret. Time	Area	Peak Start	Peak End	Area%
1	17.204	41504939	16.459	18.357	97.713
2	18.769	971237	18.357	19.339	2.287
Total		42476176			100.000

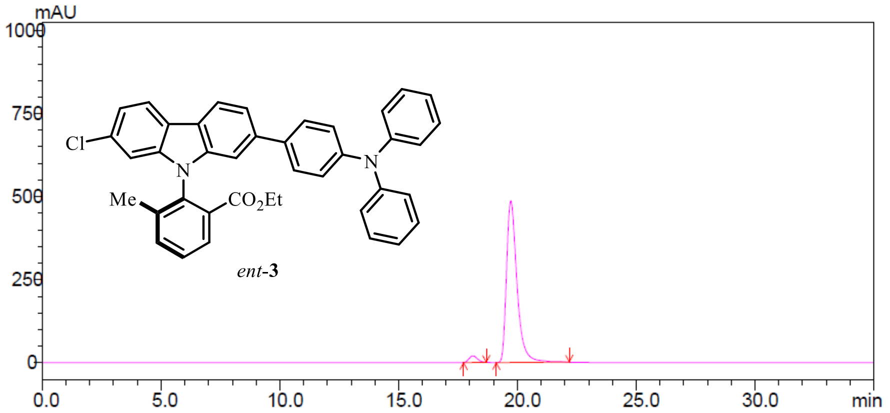

<Peak Table>

PDA Ch1 348nm

Peak#	Ret. Time	Area	Peak Start	Peak End	Area%
1	18.131	482428	17.739	18.688	3.111
2	19.725	15024198	19.115	22.187	96.889
Total		15506625			100.000

4

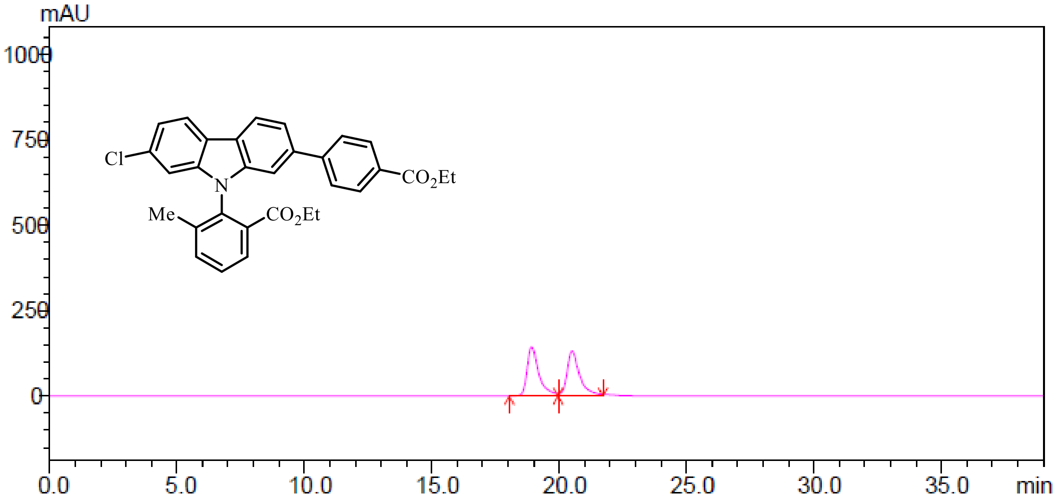

<Peak Table>

PDA Ch1 318nm

Peak#	Ret. Time	Area	Peak Start	Peak End	Area%
1	18.925	4632496	18.048	19.957	49.997
2	20.511	4632989	19.957	21.717	50.003
Total		9265485			100.000

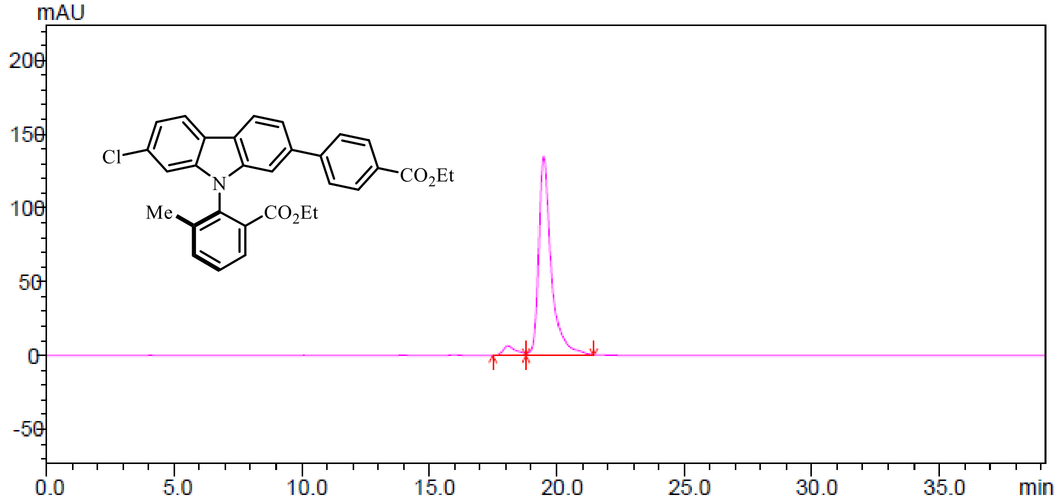

<Peak Table>

PDA Ch1 318nm

Peak#	Ret. Time	Area	Peak Start	Peak End	Area%
1	18.081	240730	17.493	18.795	4.930
2	19.486	4642181	18.795	21.451	95.070
Total		4882911			100.000

5

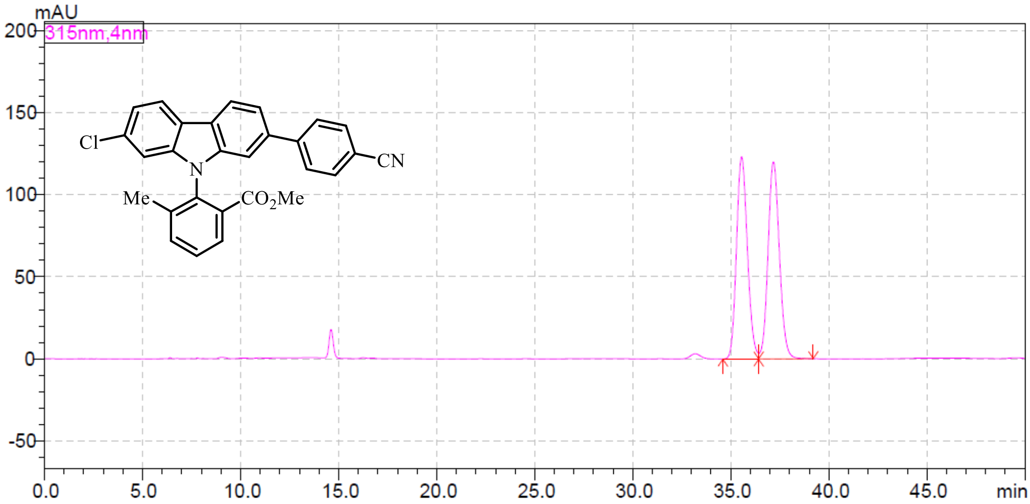

<Peak Table>

PDA Ch1 315nm

Peak#	Ret. Time	Area	Height	Peak Start	Peak End	Area%
1	35.552	4768405	123184	34.592	36.427	49.664
2	37.175	4832955	120060	36.427	39.200	50.336
Total		9601359	243244			100.000

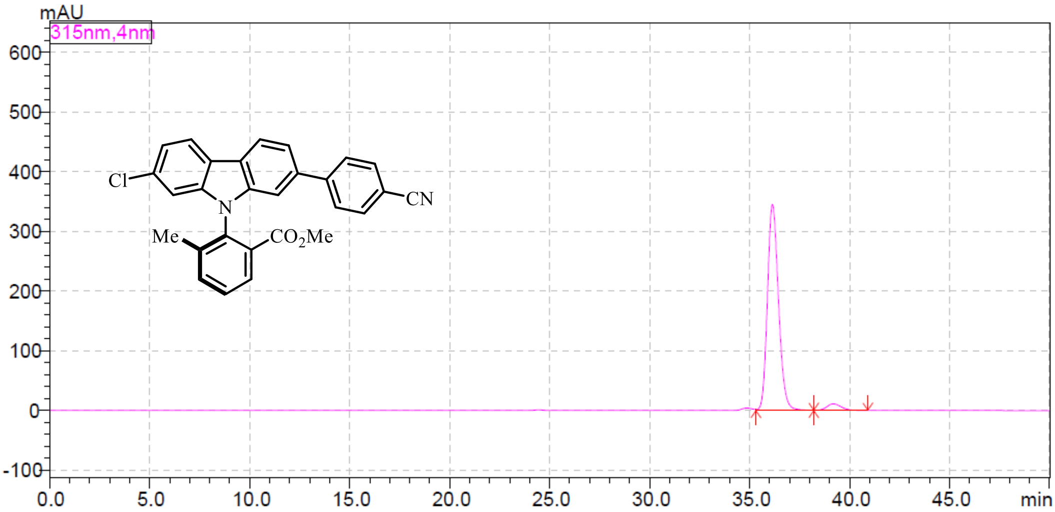

<Peak Table>

PDA Ch1 315nm

Peak#	Ret. Time	Area	Height	Peak Start	Peak End	Area%
1	36.144	13304942	345279	35.317	38.208	96.031
2	39.185	549845	10813	38.208	40.917	3.969
Total		13854787	356092			100.000

6

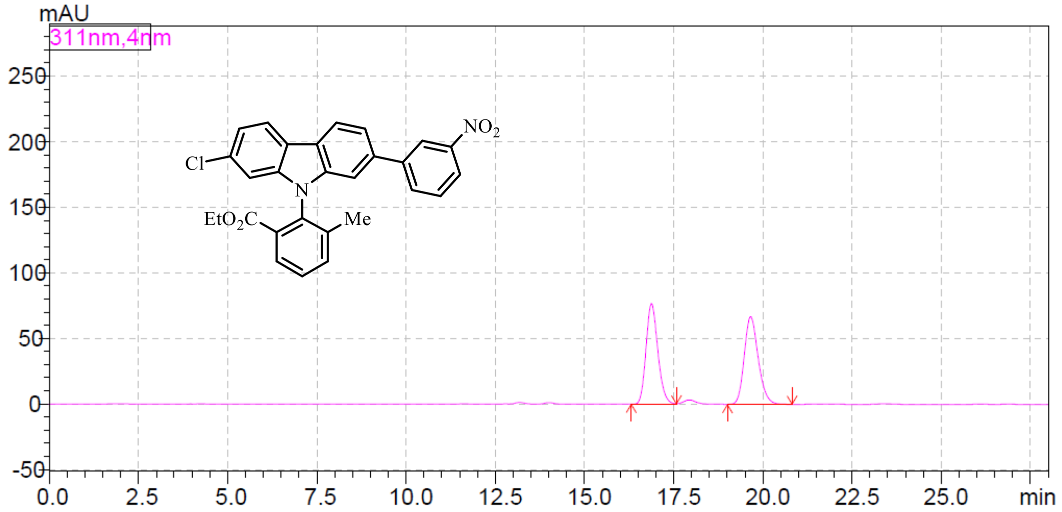

<Peak Table>

PDA Ch1 311nm

Peak#	Ret. Time	Area	Height	Peak Start	Peak End	Area%
1	16.878	1774024	76764	16.309	17.579	49.385
2	19.657	1818203	66809	19.008	20.821	50.615
Total		3592227	143573			100.000

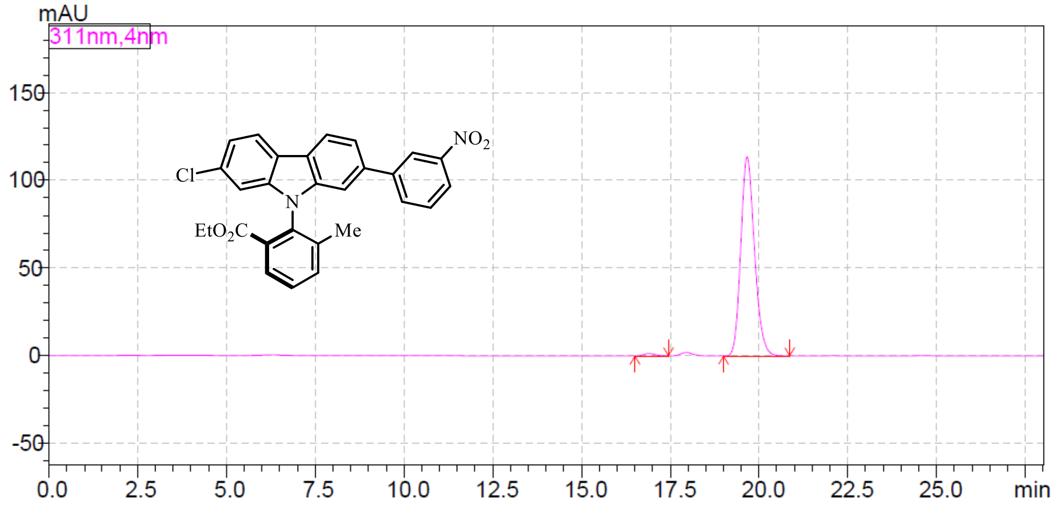

<Peak Table>

PDA Ch1 311nm

Peak#	Ret. Time	Area	Height	Peak Start	Peak End	Area%
1	16.902	30857	1359	16.501	17.451	0.997
2	19.664	3063847	113700	18.997	20.864	99.003
Total		3094703	115059			100.000

7

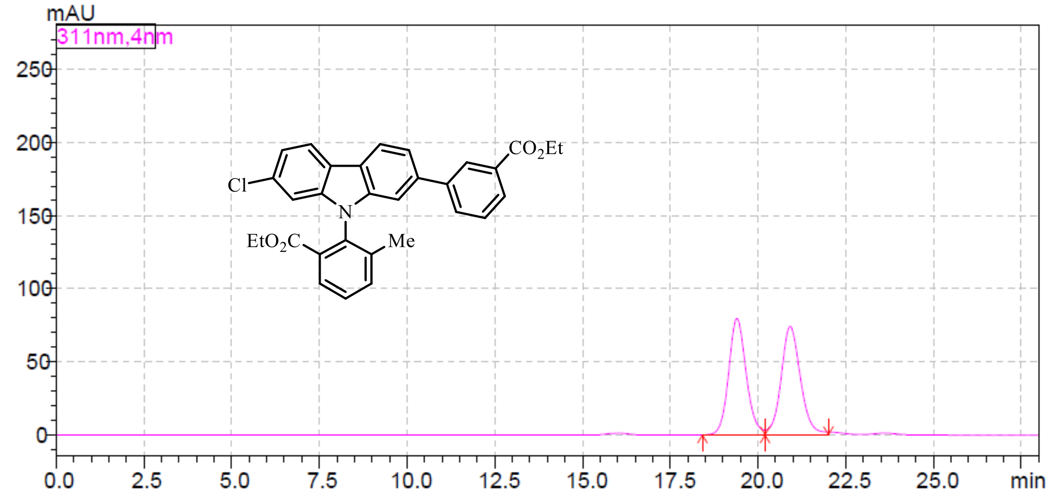

<Peak Table>

PDA Ch1 311nm

Peak#	Ret. Time	Area	Height	Peak Start	Peak End	Area%
1	19.400	2768290	79534	18.421	20.203	49.346
2	20.918	2841696	74083	20.203	22.005	50.654
Total		5609986	153617			100.000

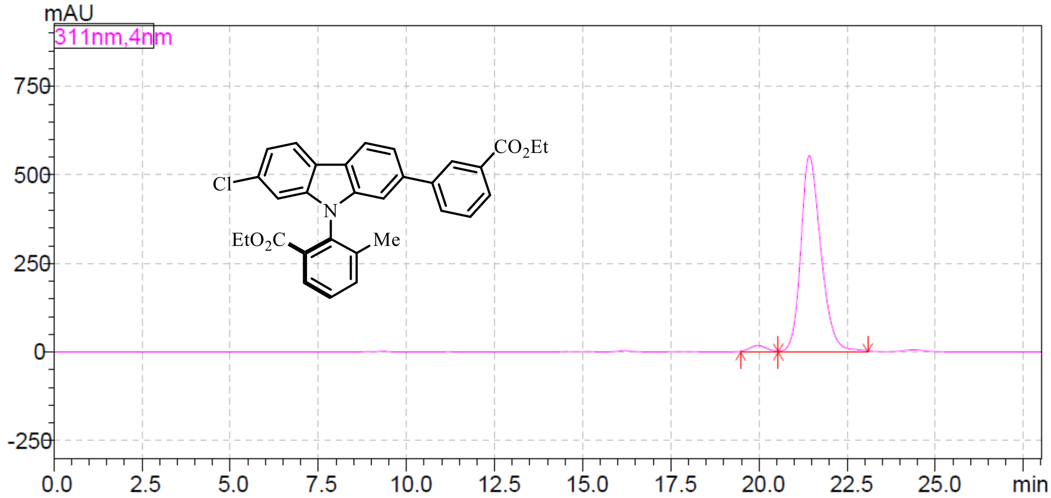

<Peak Table>

PDA Ch1 311nm

Peak#	Ret. Time	Area	Height	Peak Start	Peak End	Area%
1	19.969	635961	18391	19.477	20.544	2.802
2	21.427	22062941	554499	20.544	23.083	97.198
Total		22698902	572889			100.000

8

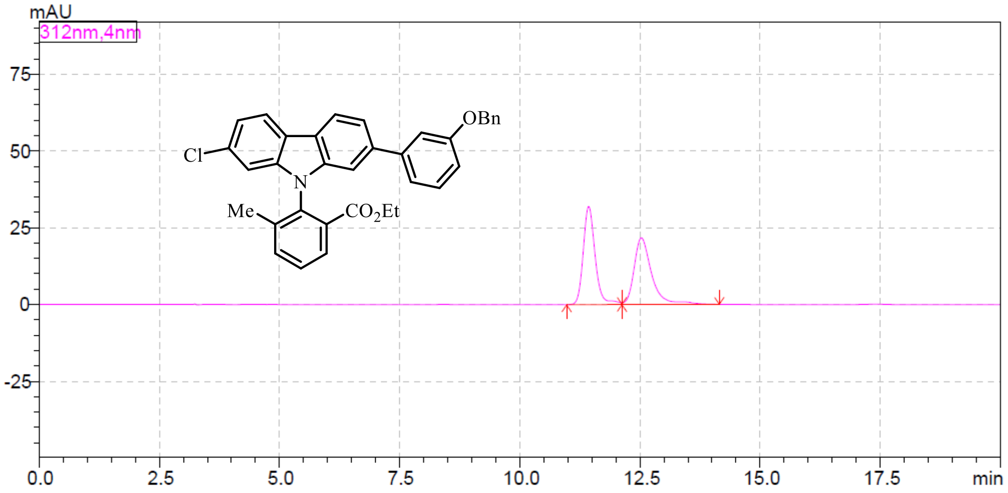

<Peak Table>

PDA Ch1 312nm

Peak#	Ret. Time	Area	Height	Peak Start	Peak End	Area%
1	11.436	553434	31976	10.976	12.128	49.987
2	12.533	553716	21738	12.128	14.155	50.013
Total		1107150	53714			100.000

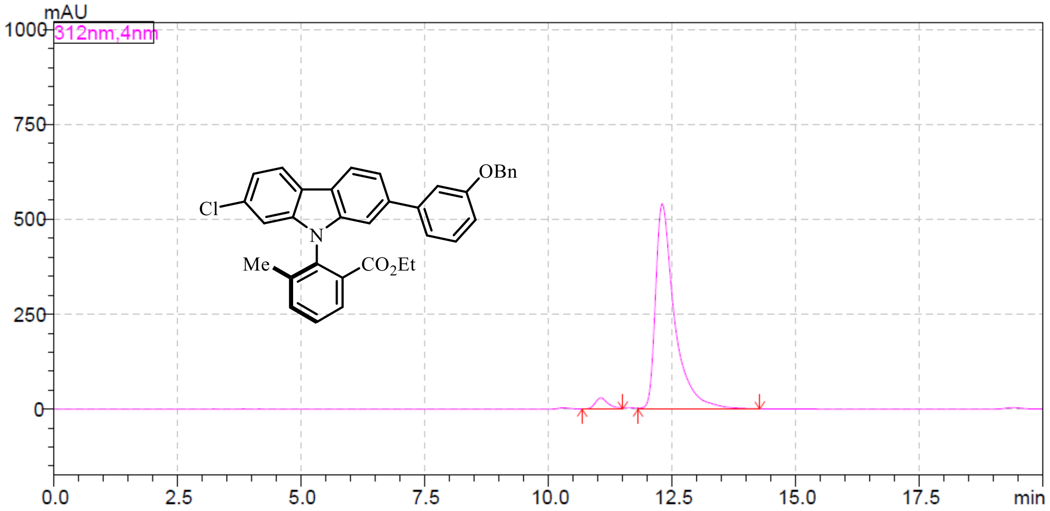

<Peak Table>

PDA Ch1 312nm

Peak#	Ret. Time	Area	Height	Peak Start	Peak End	Area%
1	11.066	555168	29450	10.688	11.509	3.690
2	12.310	14489468	541039	11.819	14.283	96.310
Total		15044637	570489			100.000

9

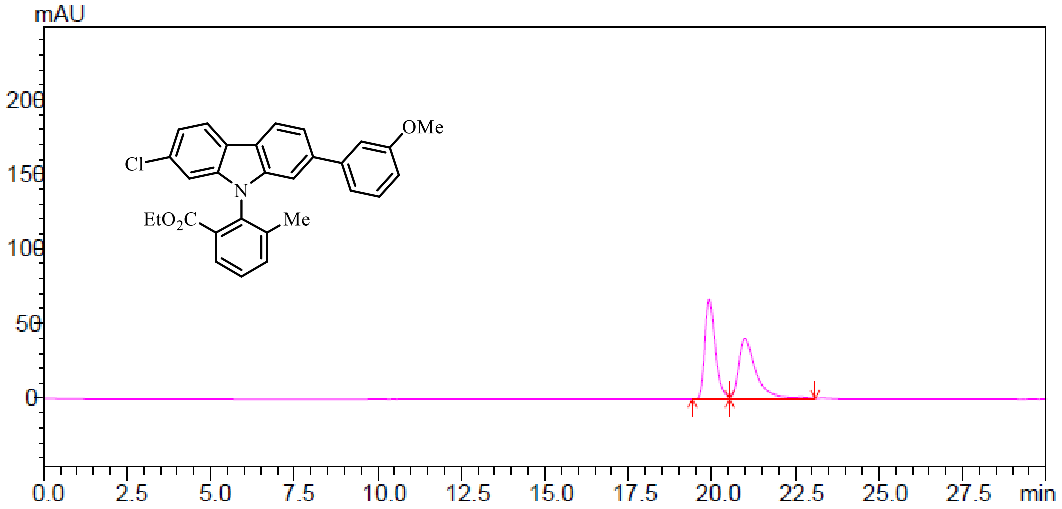

<Peak Table>

PDA Ch1 330nm

Peak#	Ret. Time	Area	Peak Start	Peak End	Area%
1	19.925	1454063	19.424	20.533	49.488
2	20.992	1484147	20.533	23.093	50.512
Total		2938210			100.000

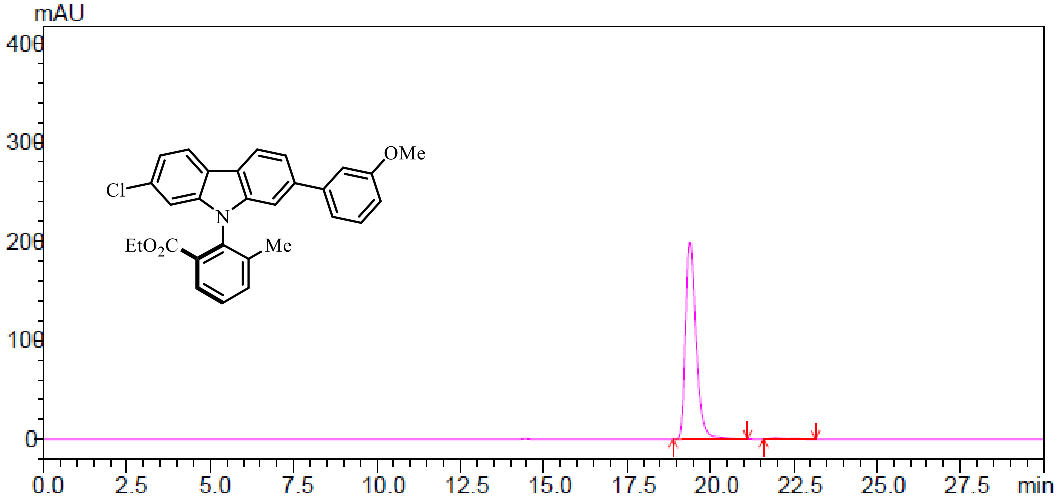

<Peak Table>

PDA Ch1 330nm

Peak#	Ret. Time	Area	Height	Peak Start	Peak End	Area%
1	19.389	4346388	198751	18.880	21.120	98.971
2	21.967	45199	1162	21.600	23.147	1.029
Total		4391587	199913			100.000

10

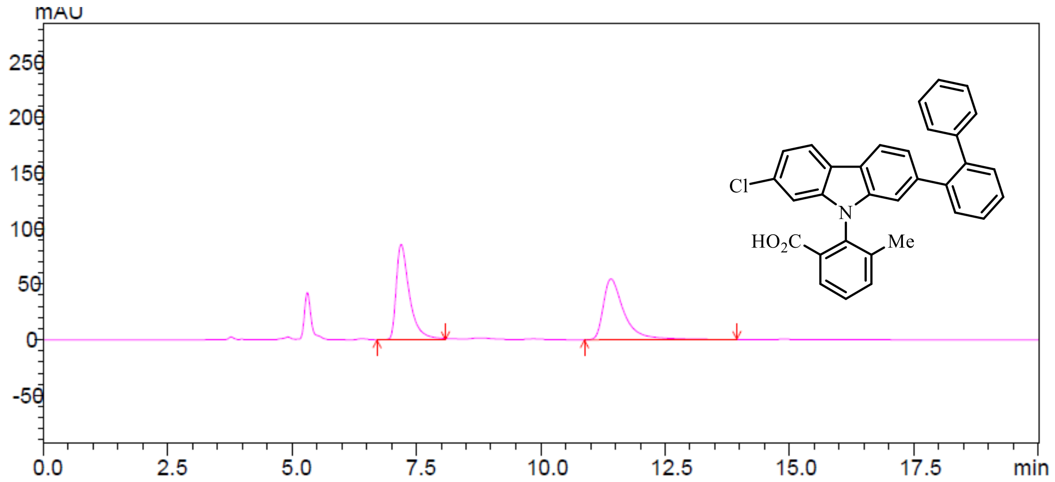

<Peak Table>

PDA Ch1 309nm

Peak#	Ret. Time	Area	Height	Peak Start	Peak End	Area%
1	7.194	1599210	85624	6.709	8.085	50.265
2	11.413	1582377	54696	10.880	13.941	49.735
Total		3181587	140320			100.000

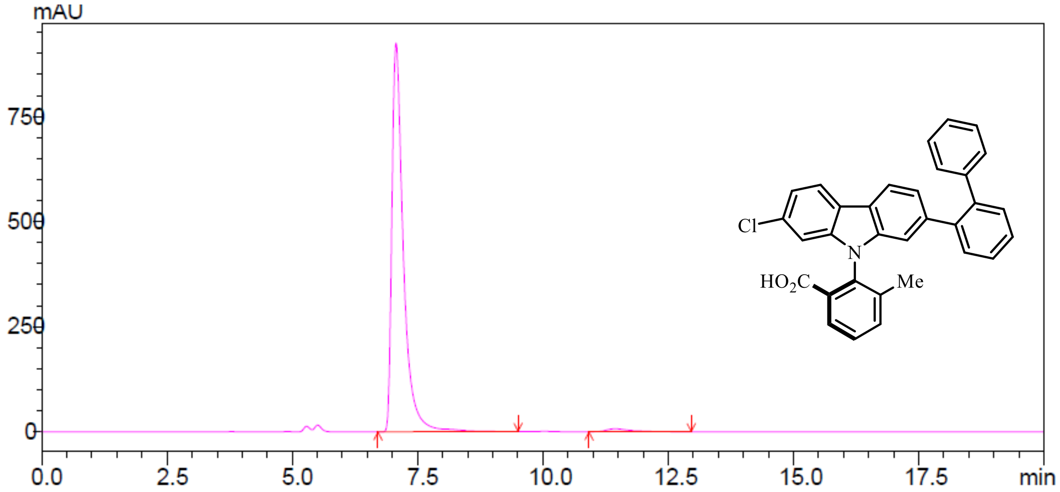

<Peak Table>

PDA Ch1 309nm

Peak#	Ret. Time	Area	Height	Peak Start	Peak End	Area%
1	7.071	14775328	924793	6.709	9.504	98.497
2	11.441	225415	6771	10.923	12.960	1.503
Total		15000743	931564			100.000

11

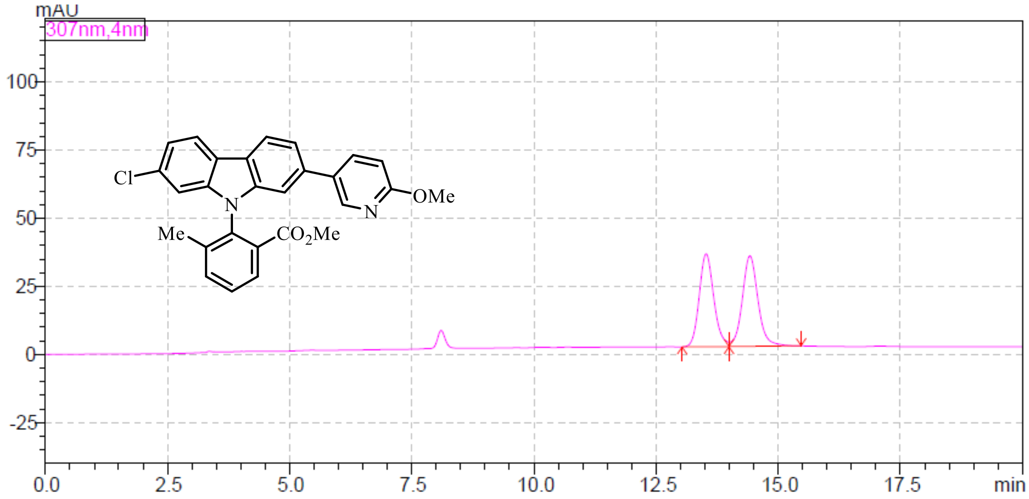

<Peak Table>

PDA Ch1 307nm

Peak#	Ret. Time	Area	Height	Peak Start	Peak End	Area%
1	13.526	706895	34070	13.035	13.995	48.858
2	14.423	739929	33224	13.995	15.467	51.142
Total		1446824	67293			100.000

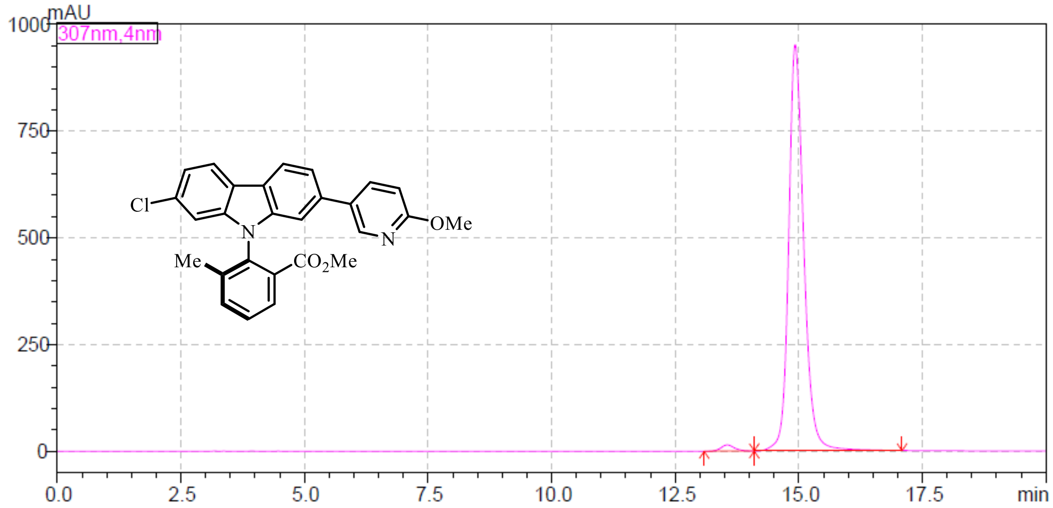

<Peak Table>

PDA Ch1 307nm

Peak#	Ret. Time	Area	Height	Peak Start	Peak End	Area%
1	13.556	276831	14357	13.088	14.101	1.359
2	14.931	20098202	952908	14.101	17.088	98.641
Total		20375033	967264			100.000

12

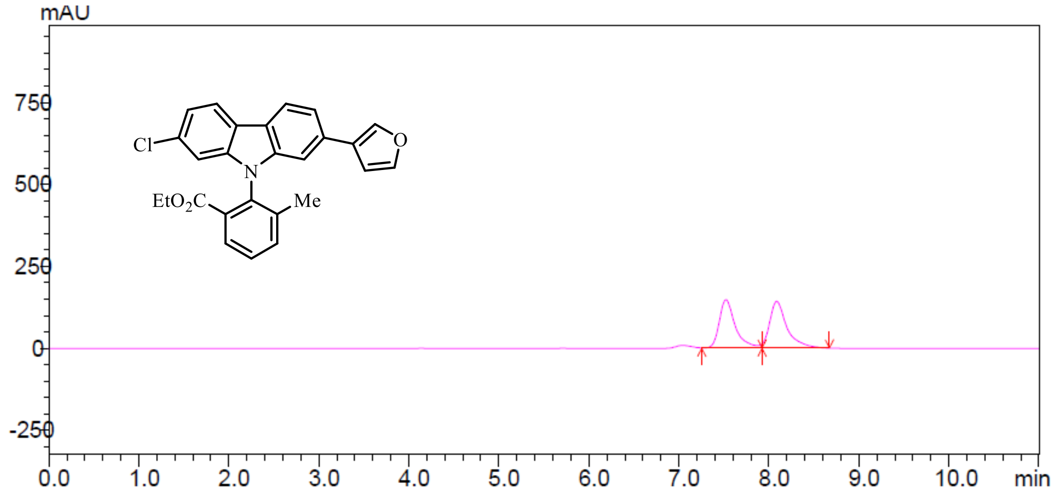

<Peak Table>

PDA Ch1 313nm

Peak#	Ret. Time	Area	Peak Start	Peak End	Area%
1	7.523	1831631	7.253	7.925	49.237
2	8.087	1888380	7.925	8.672	50.763
Total		3720011			100.000

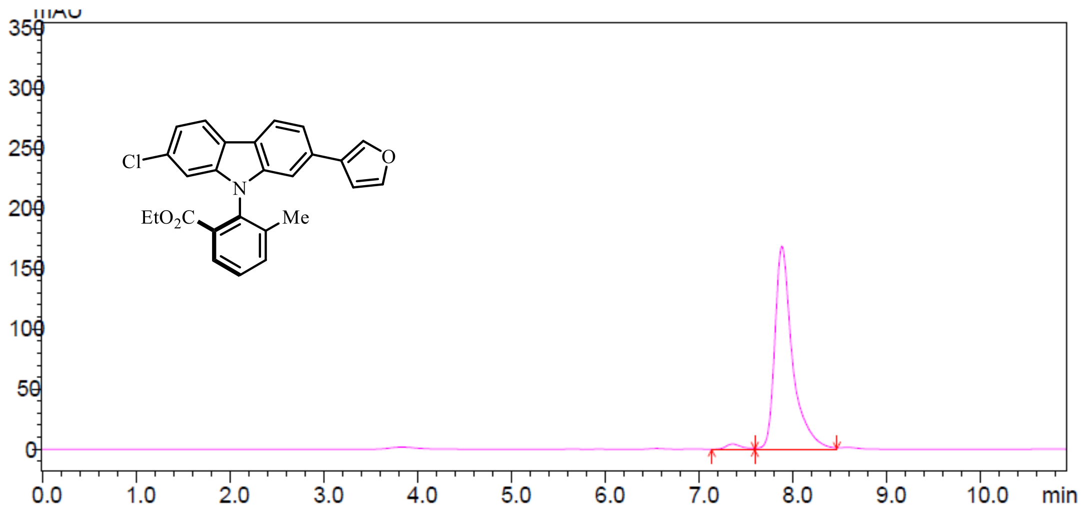

<Peak Table>

PDA Ch1 313nm

Peak#	Ret. Time	Area	Peak Start	Peak End	Area%
1	7.360	54932	7.136	7.595	2.417
2	7.885	2218156	7.595	8.469	97.583
Total		2273088			100.000

13

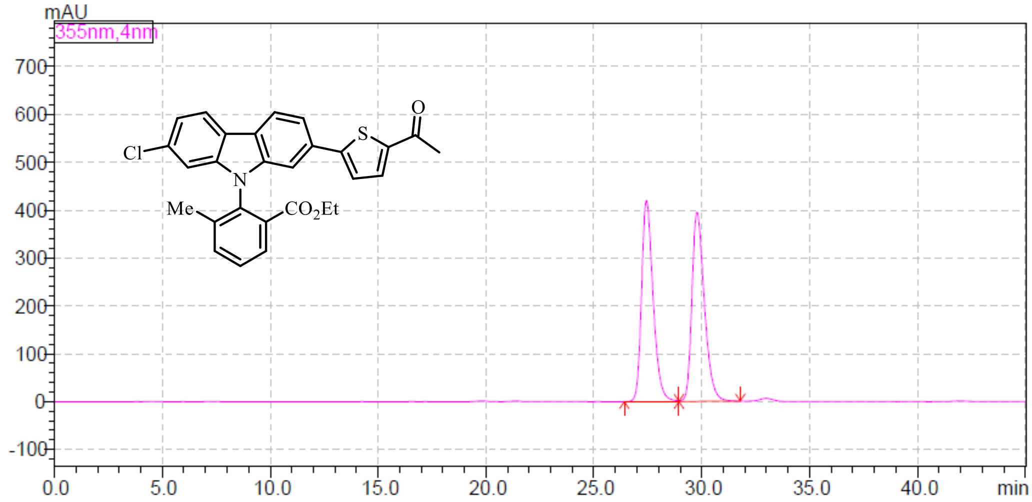

<Peak Table>

PDA Ch1 355nm

Peak#	Ret. Time	Area	Height	Peak Start	Peak End	Area%
1	27.434	15613067	420409	26.411	28.939	49.595
2	29.779	15868181	395162	28.939	31.787	50.405
Total		31481248	815571			100.000

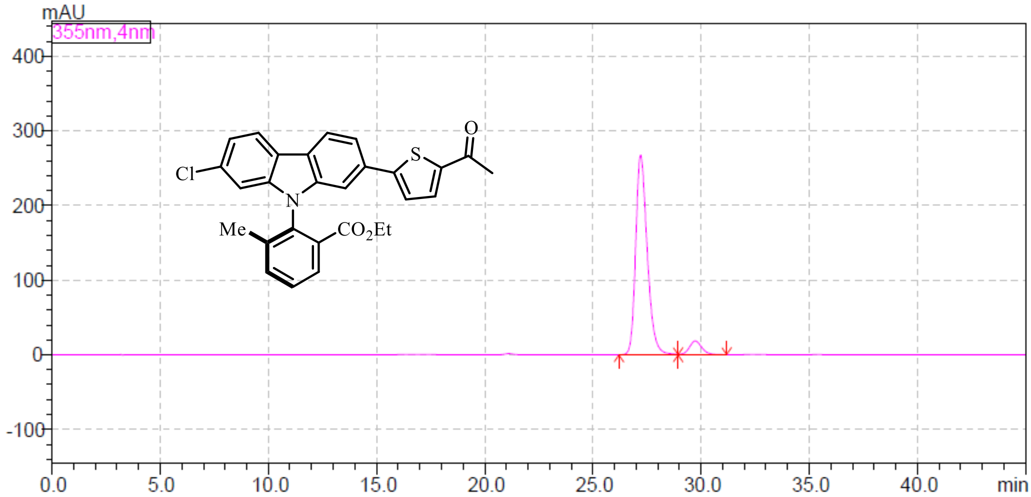

<Peak Table>

PDA Ch1 355nm

Peak#	Ret. Time	Area	Height	Peak Start	Peak End	Area%
1	27.209	9950975	267577	26.208	28.939	93.079
2	29.732	739880	18379	28.939	31.189	6.921
Total		10690854	285956			100.000

14

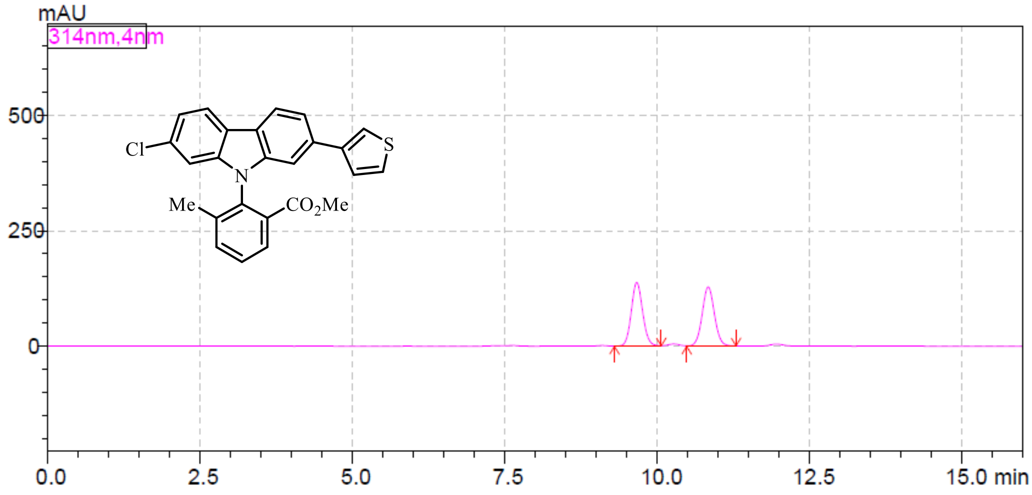

<Peak Table>

PDA Ch1 314nm

Peak#	Ret. Time	Area	Height	Peak Start	Peak End	Area%
1	9.666	1807672	138134	9.301	10.069	49.920
2	10.840	1813471	128543	10.485	11.296	50.080
Total		3621143	266677			100.000

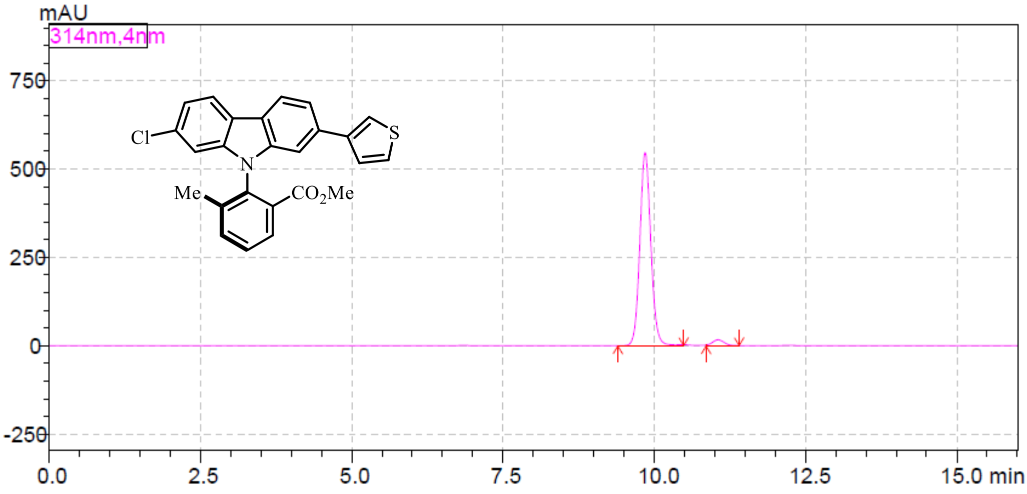

<Peak Table>

PDA Ch1 314nm

Peak#	Ret. Time	Area	Height	Peak Start	Peak End	Area%
1	9.850	6931373	546318	9.397	10.485	96.790
2	11.058	229867	16854	10.859	11.403	3.210
Total		7161240	563172			100.000

15

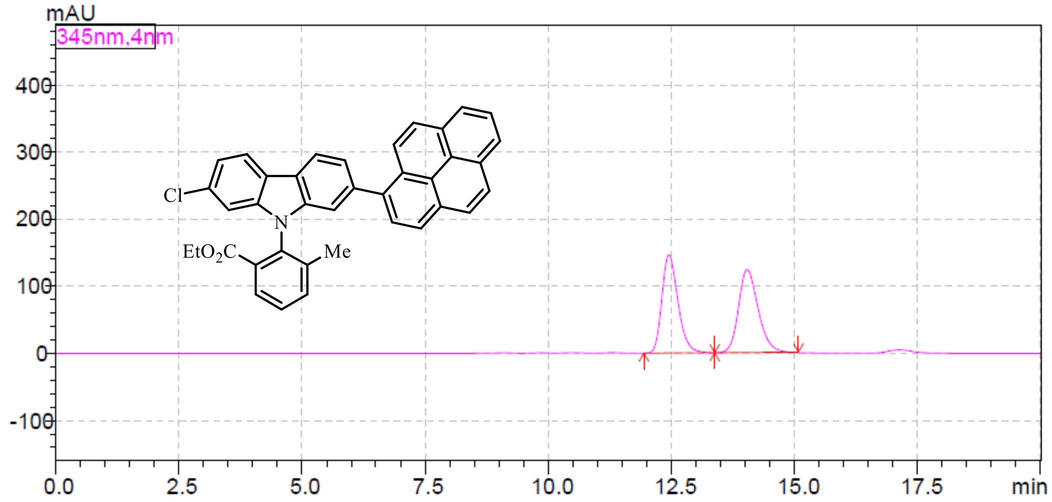

<Peak Table>

PDA Ch1 345nm

Peak#	Ret. Time	Area	Height	Peak Start	Peak End	Area%
1	12.455	3329236	146630	11.947	13.387	49.219
2	14.046	3434835	123838	13.387	15.083	50.781
Total		6764070	270468			100.000

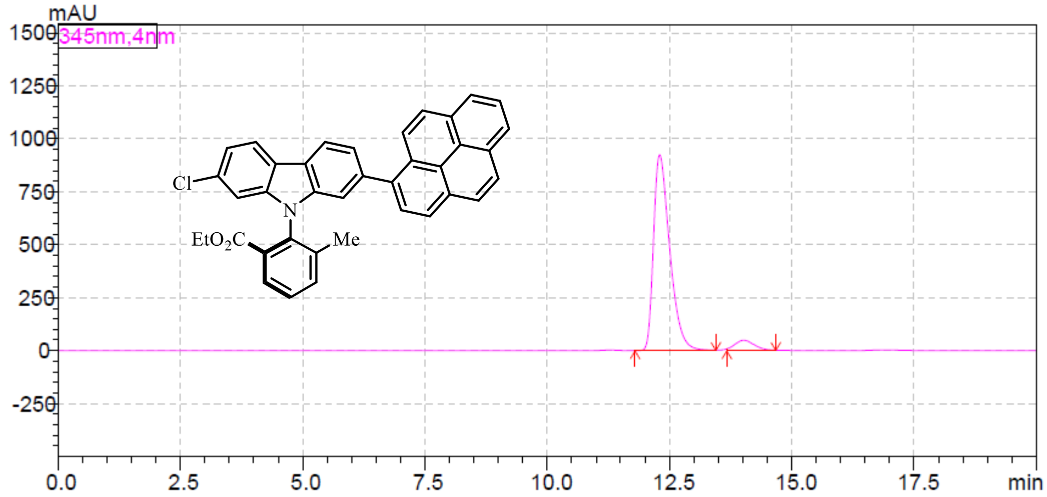

<Peak Table>

PDA Ch1 345nm

Peak#	Ret. Time	Area	Height	Peak Start	Peak End	Area%
1	12.302	21362542	924674	11.797	13.451	93.801
2	14.021	1411867	49115	13.675	14.677	6.199
Total		22774409	973789			100.000

16

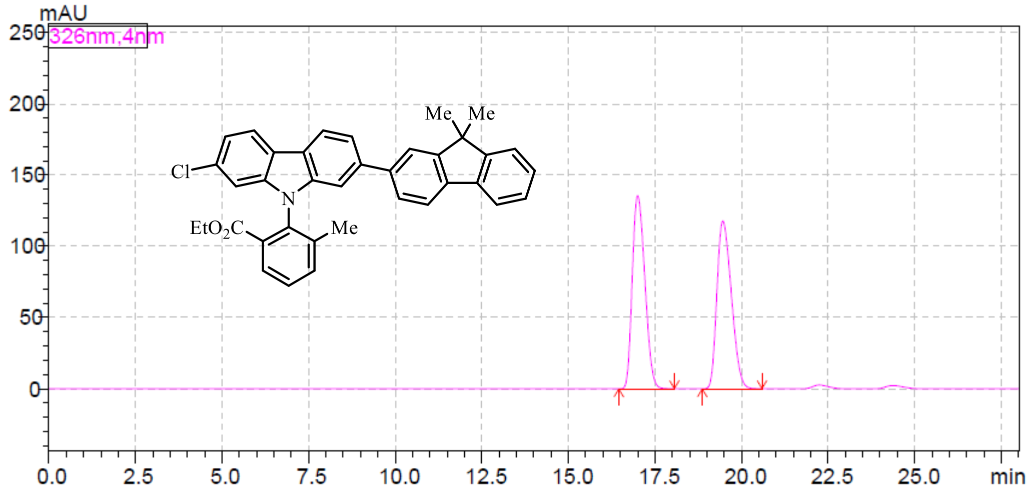

<Peak Table>

PDA Ch1 326nm

Peak#	Ret. Time	Area	Height	Conc.	Peak Start	Peak End	Area%
1	17.001	3430640	135614	0.000	16.459	18.059	50.017
2	19.460	3428355	117790	0.000	18.869	20.587	49.983
Total		6858995	253404				100.000

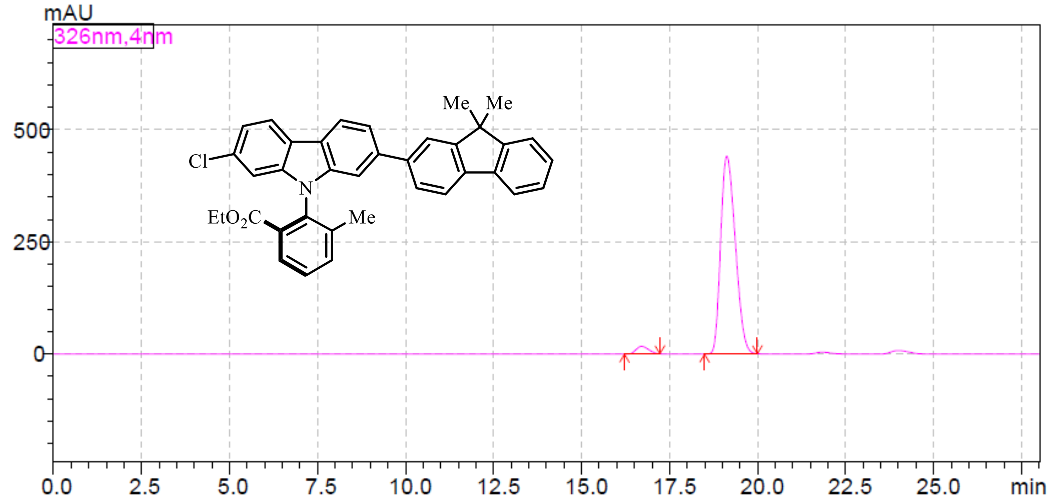

<Peak Table>

PDA Ch1 326nm

Peak#	Ret. Time	Area	Height	Peak Start	Peak End	Area%
1	16.703	428177	16986	16.213	17.227	3.241
2	19.125	12784726	441779	18.496	19.989	96.759
Total		13212903	458765			100.000

17

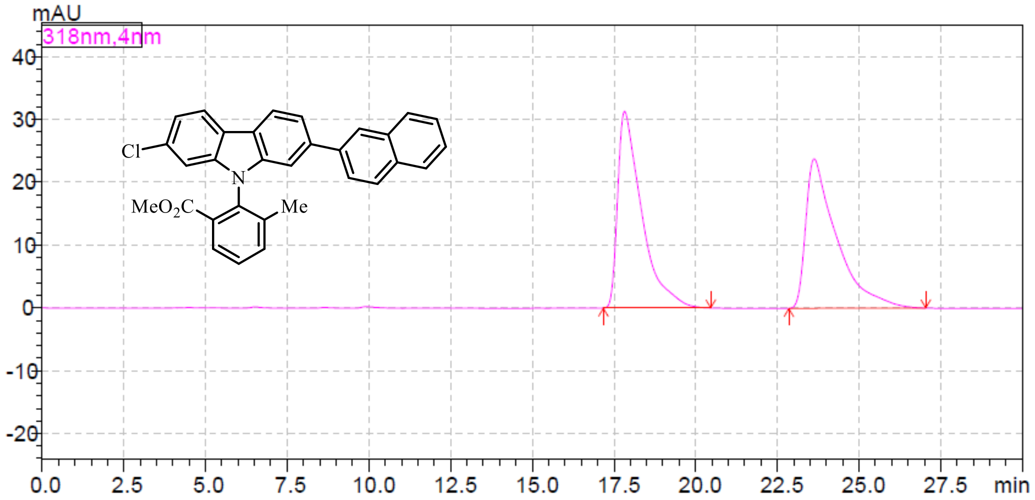

<Peak Table>

PDA Ch1 318nm

Peak#	Ret. Time	Area	Height	Peak Start	Peak End	Area%
1	17.824	1599642	31338	17.173	20.459	50.587
2	23.628	1562490	23778	22.848	27.040	49.413
Total		3162132	55116			100.000

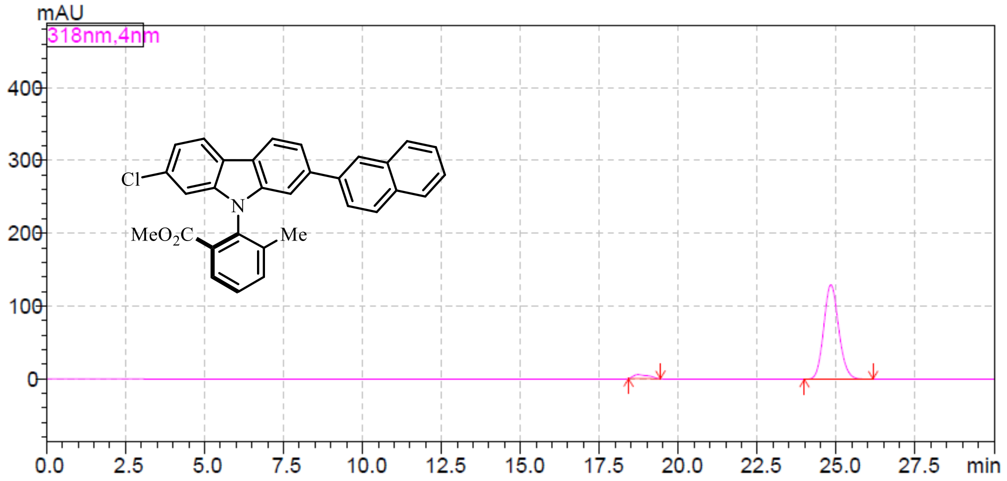

<Peak Table>

PDA Ch1 318nm

Peak#	Ret. Time	Area	Height	Peak Start	Peak End	Area%
1	18.726	207103	5982	18.421	19.435	4.602
2	24.829	4293368	129782	23.989	26.165	95.398
Total		4500471	135765			100.000

18

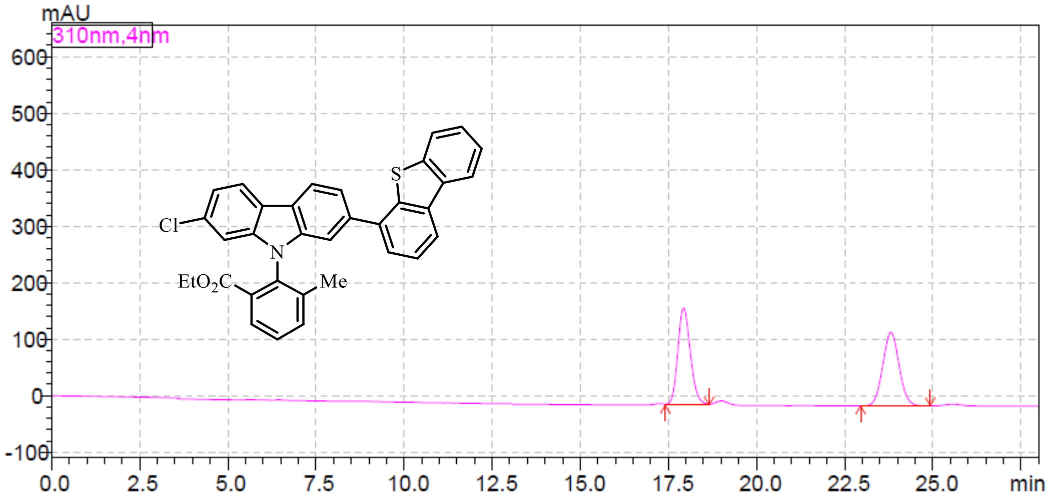

<Peak Table>

PDA Ch1 310nm

Peak#	Ret. Time	Area	Height	Peak Start	Peak End	Area%
1	17.930	4313263	171946	17.408	18.656	50.551
2	23.814	4219287	130319	22.955	24.928	49.449
Total		8532549	302265			100.000

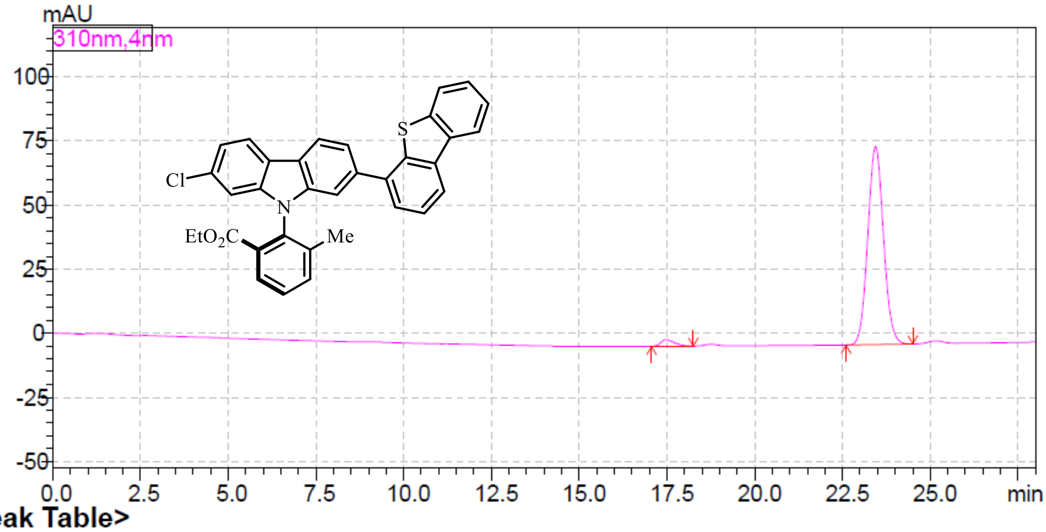

<Peak Table>

PDA Ch1 310nm

Peak#	Ret. Time	Area	Height	Peak Start	Peak End	Area%
1	17.471	69342	2602	17.056	18.251	2.799
2	23.442	2407999	77299	22.603	24.501	97.201
Total		2477342	79901			100.000

19

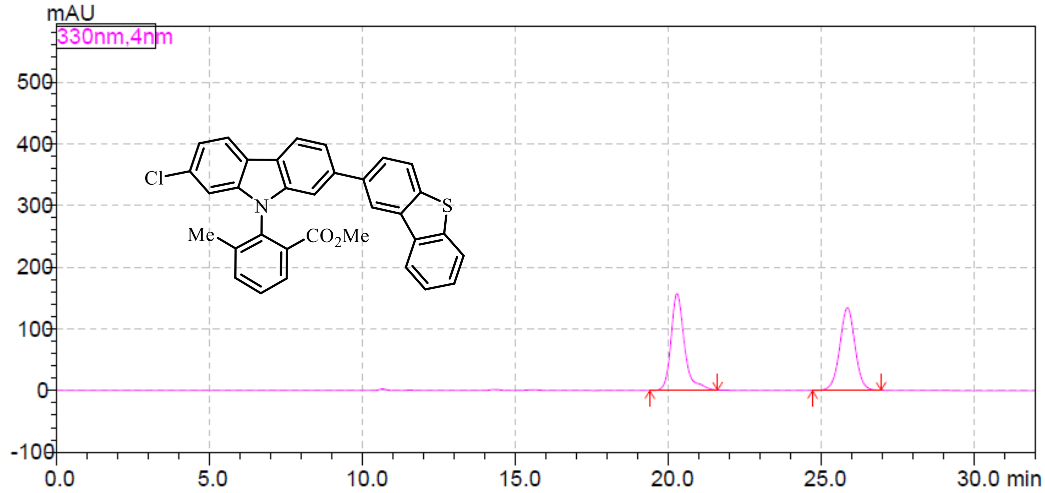

<Peak Table>

PDA Ch1 330nm

Peak#	Ret. Time	Area	Height	Peak Start	Peak End	Area%
1	20.294	4726981	156210	19.371	21.461	50.187
2	25.867	4691805	134243	24.725	26.965	49.813
Total		9418786	290453			100.000

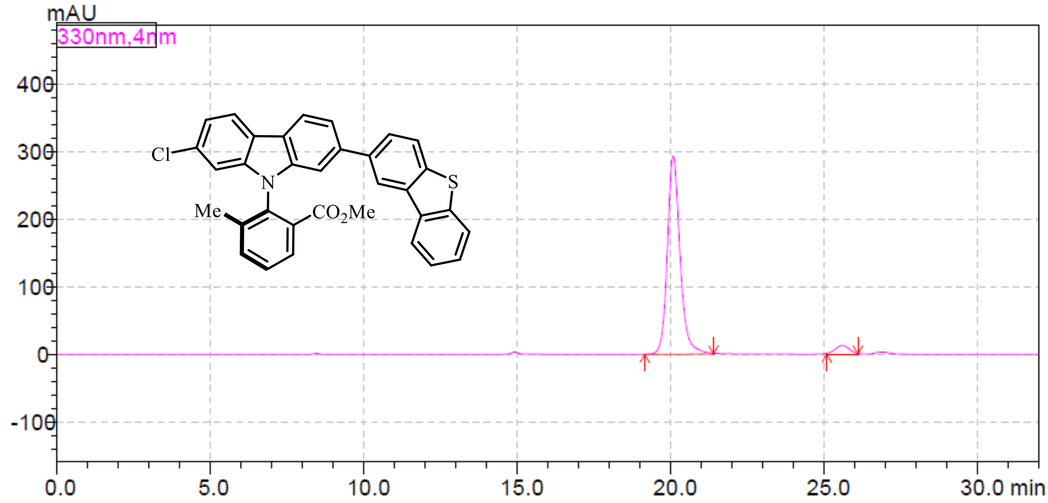

<Peak Table>

PDA Ch1 330nm

Peak#	Ret. Time	Area	Height	Peak Start	Peak End	Area%
1	20.091	8426091	293392	19.168	21.408	95.214
2	25.605	423581	12967	25.099	26.112	4.786
Total		8849672	306359			100.000

20

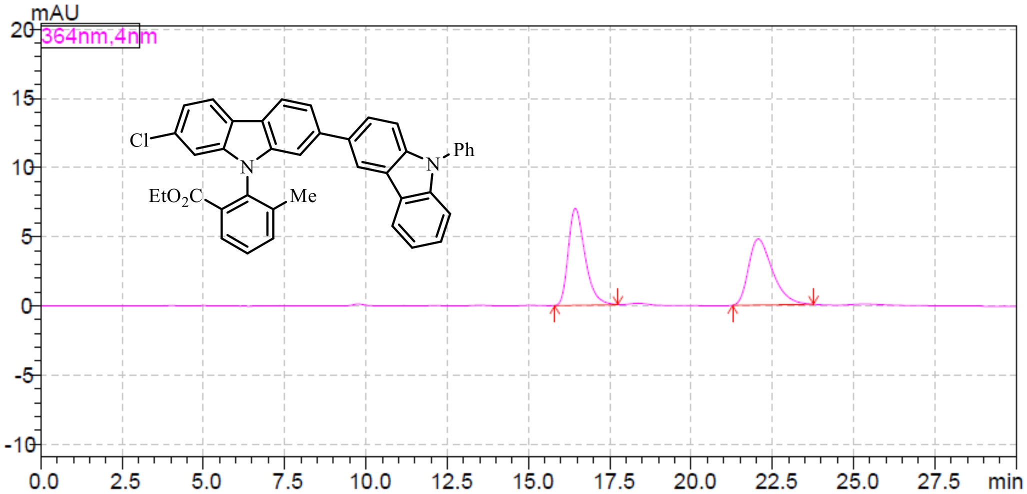

<Peak Table>

PDA Ch1 364nm

Peak#	Ret. Time	Area	Height	Peak Start	Peak End	Area%
1	16.439	251049	7005	15.808	17.749	50.589
2	22.063	245198	4780	21.280	23.765	49.411
Total		496247	11785			100.000

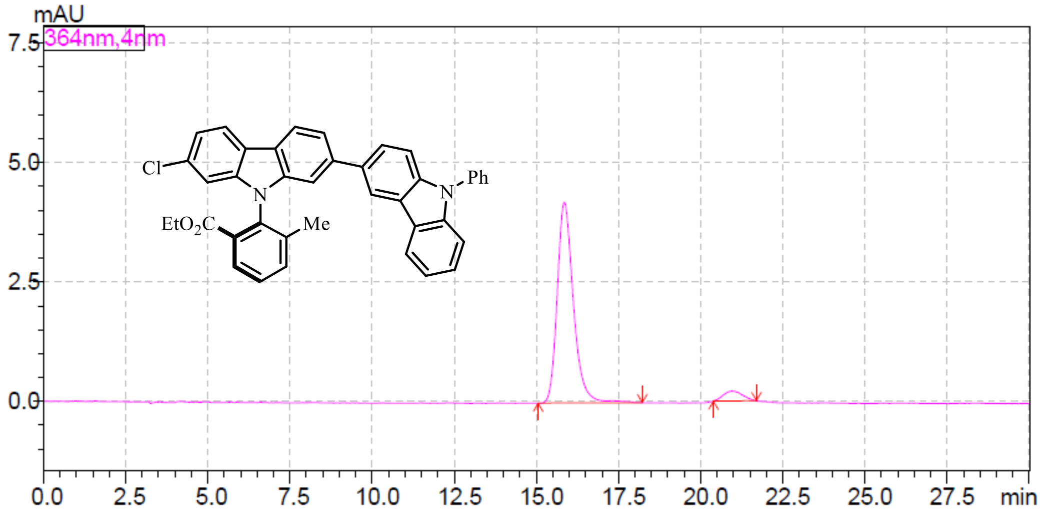

<Peak Table>

PDA Ch1 364nm

Peak#	Ret. Time	Area	Height	Peak Start	Peak End	Area%
1	15.849	144145	4203	15.051	18.219	94.453
2	20.955	8465	206	20.384	21.696	5.547
Total		152610	4409			100.000

21

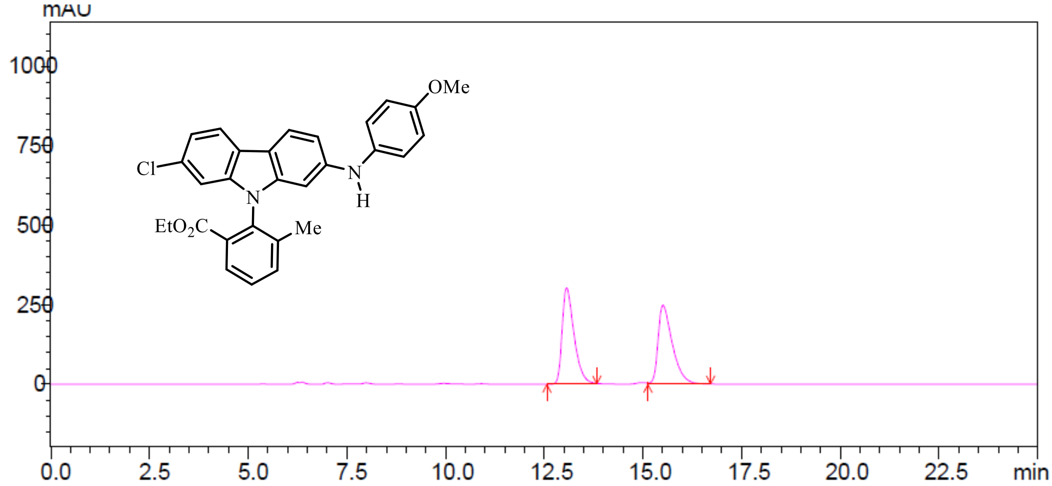

<Peak Table>

PDA Ch1 344nm

Peak#	Ret. Time	Area	Height	Peak Start	Peak End	Area%
1	13.068	6436688	302860	12.565	13.835	50.395
2	15.509	6335808	248726	15.125	16.715	49.605
Total		12772496	551585			100.000

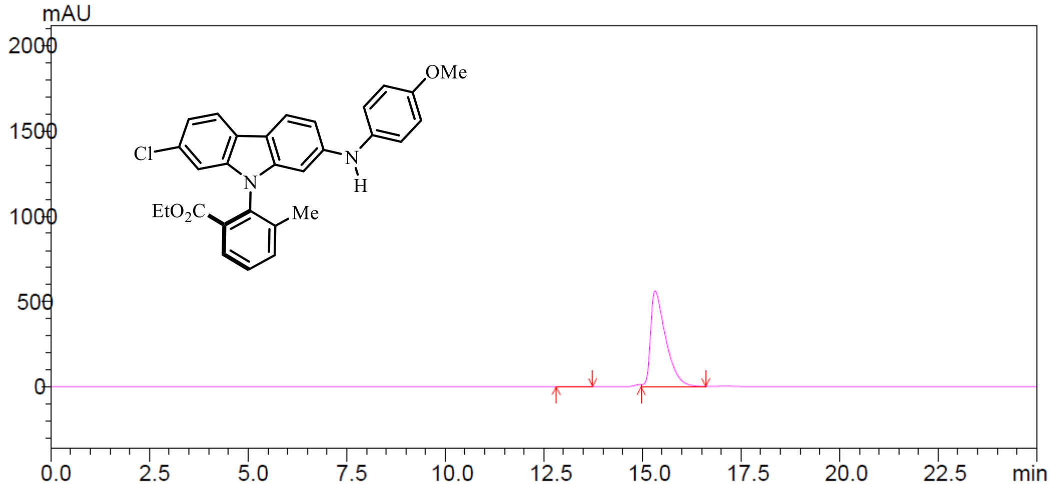

<Peak Table>

PDA Ch1 344nm

Peak#	Ret. Time	Area	Height	Peak Start	Peak End	Area%
1	13.196	28050	1325	12.811	13.739	0.185
2	15.325	15095754	562362	14.965	16.608	99.815
Total		15123805	563687			100.000

22

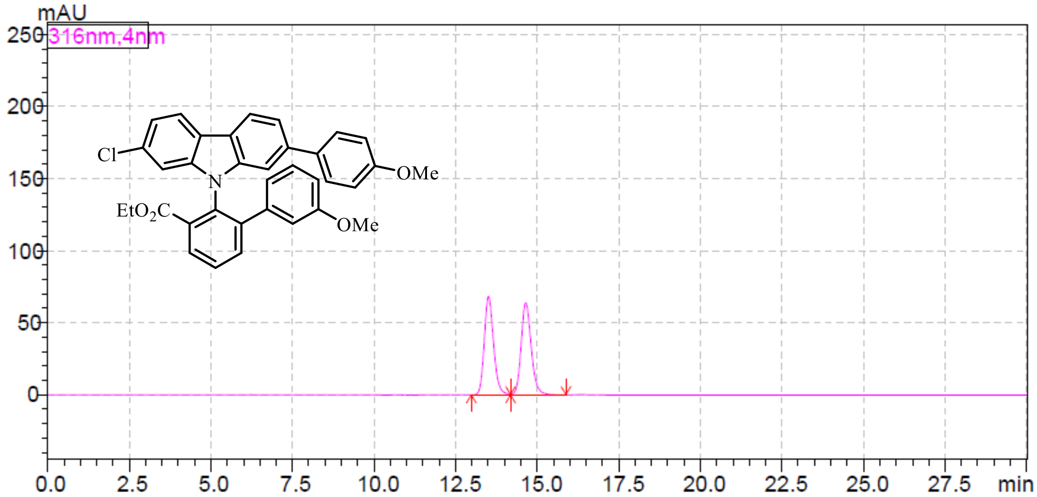

<Peak Table>

PDA Ch1 316nm

Peak#	Ret. Time	Area	Height	Peak Start	Peak End	Area%
1	13.509	1355528	68235	12.981	14.187	49.713
2	14.650	1371182	63793	14.187	15.883	50.287
Total		2726710	132028			100.000

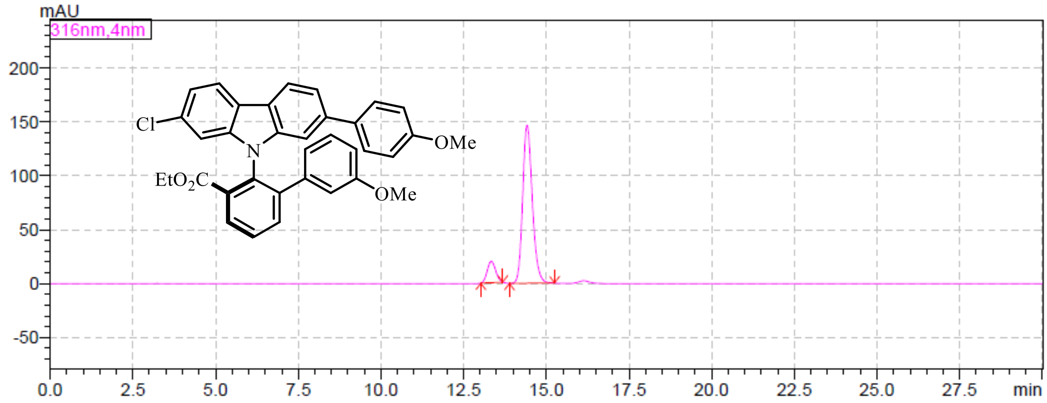

<Peak Table>

PDA Ch1 316nm

Peak#	Ret. Time	Area	Height	Peak Start	Peak End	Area%
1	13.338	347313	19786	13.024	13.685	10.459
2	14.419	2973499	146715	13.888	15.253	89.541
Total		3320813	166501			100.000

23

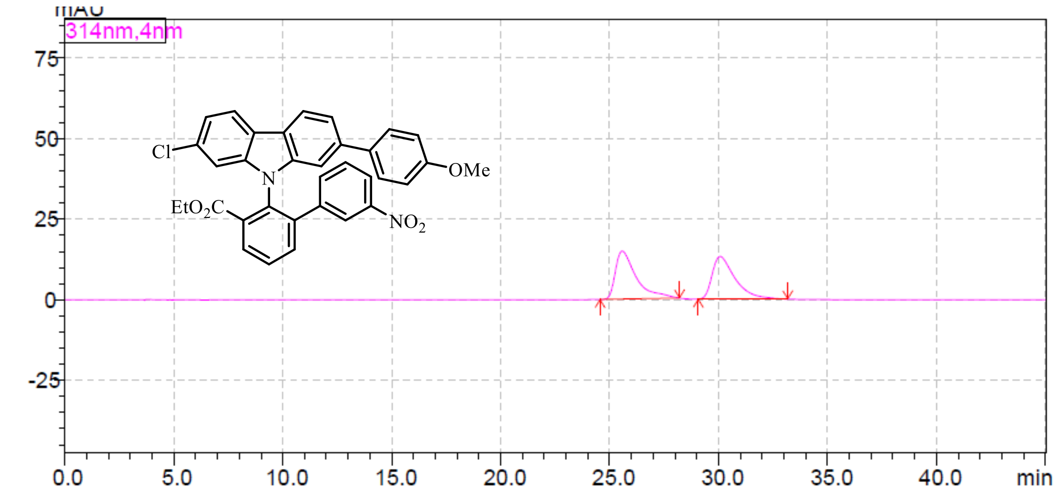

<Peak Table>

PDA Ch1 314nm

Peak#	Ret. Time	Area	Height	Peak Start	Peak End	Area%
1	25.580	988842	14871	24.555	28.203	50.613
2	30.077	964905	13221	29.067	33.173	49.387
Total		1953747	28092			100.000

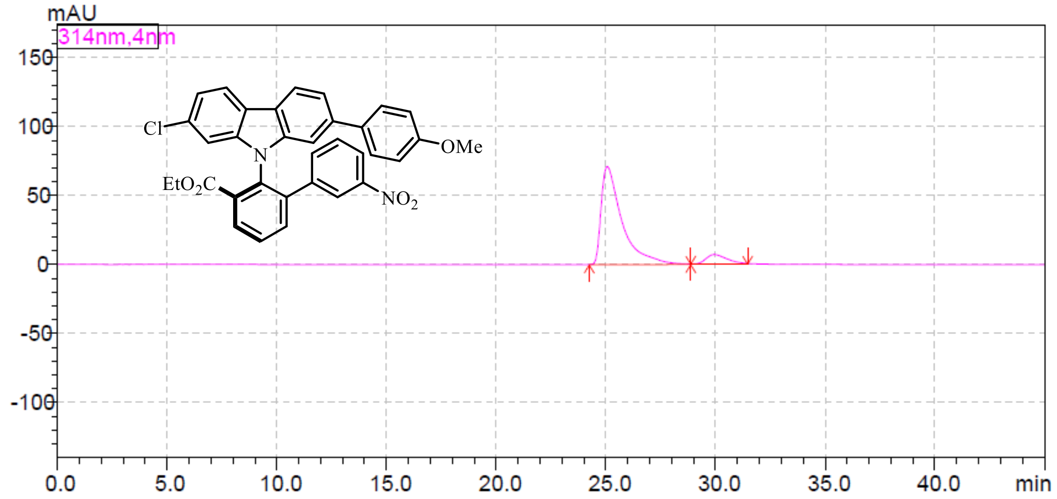

<Peak Table>

PDA Ch1 314nm

Peak#	Ret. Time	Area	Height	Peak Start	Peak End	Area%
1	25.068	4707737	71044	24.245	28.875	90.316
2	29.949	504785	7175	28.875	31.467	9.684
Total		5212521	78219			100.000

24

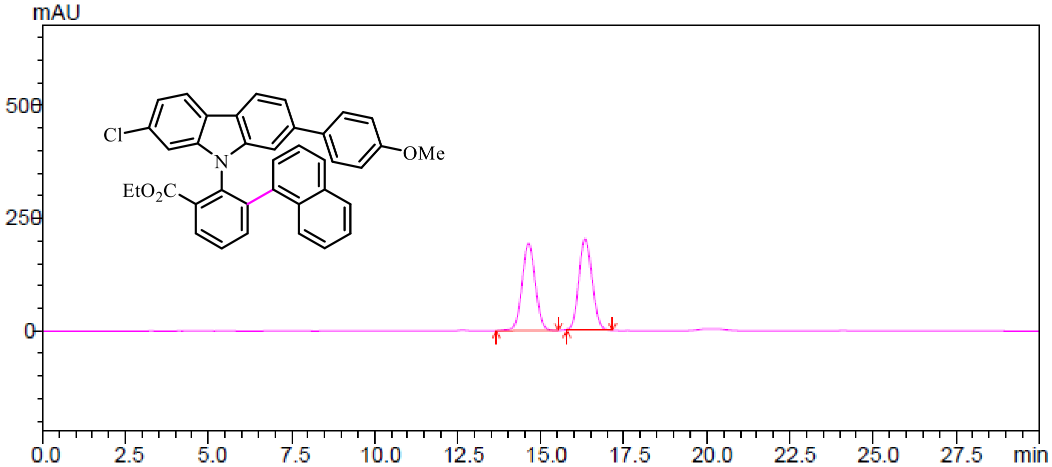

<Peak Table>

PDA Ch1 313nm

Peak#	Ret. Time	Area	Peak Start	Peak End	Area%
1	14.637	5440074	13.659	15.531	49.059
2	16.338	5648859	15.781	17.163	50.941
Total		11088933			100.000

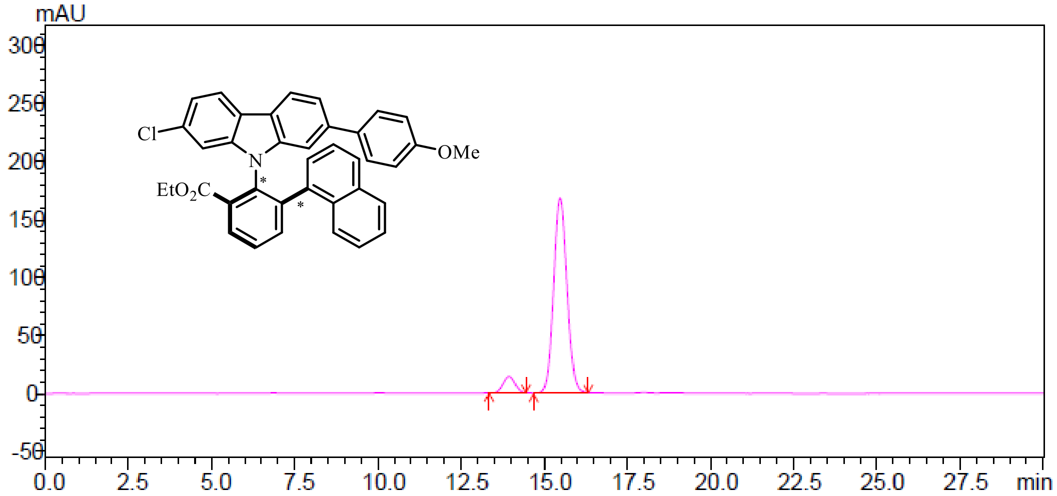

<Peak Table>

PDA Ch1 313nm

Peak#	Ret. Time	Area	Height	Peak Start	Peak End	Area%
1	13.950	366426	14412	13.344	14.453	7.123
2	15.483	4777809	168460	14.688	16.331	92.877
Total		5144234	182872			100.000

25

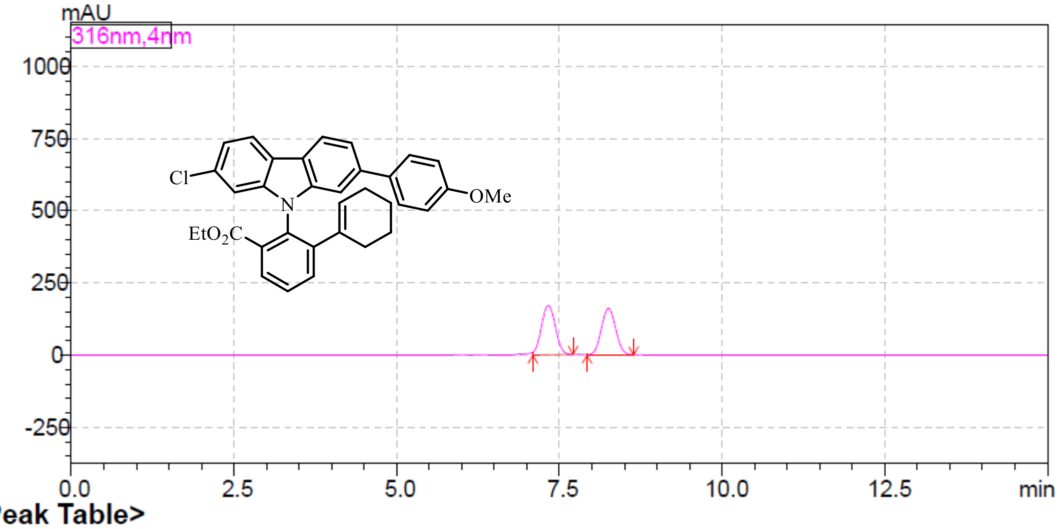

<Peak Table>

PDA Ch1 316nm

Peak#	Ret. Time	Area	Height	Peak Start	Peak End	Area%
1	7.330	2562631	171147	7.093	7.712	50.502
2	8.254	2511664	161997	7.925	8.640	49.498
Total		5074295	333145			100.000

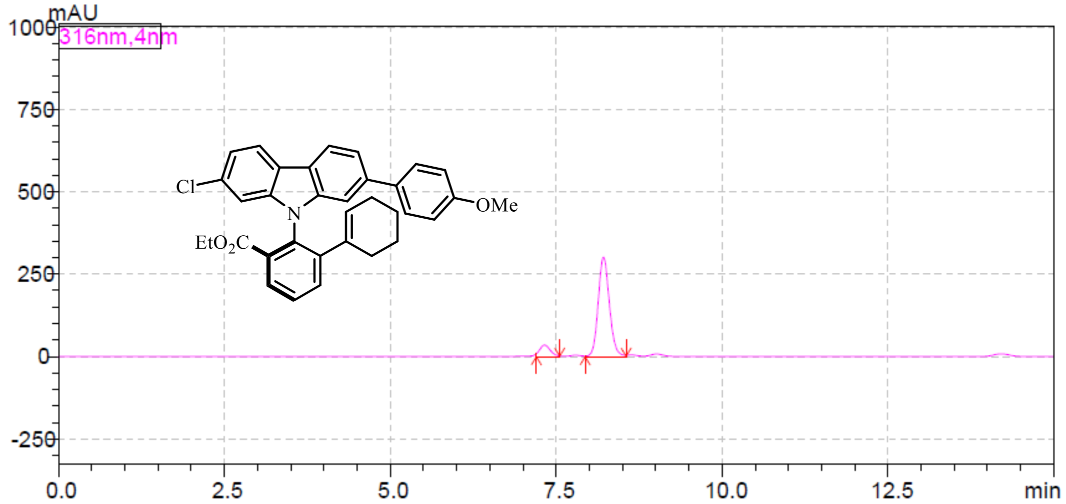

<Peak Table>

PDA Ch1 316nm

Peak#	Ret. Time	Area	Height	Area%	Peak Start	Peak End
1	7.325	381464	34649	9.739	7.200	7.552
2	8.213	3535546	302056	90.261	7.936	8.555
Total		3917009	336705	100.000		

26

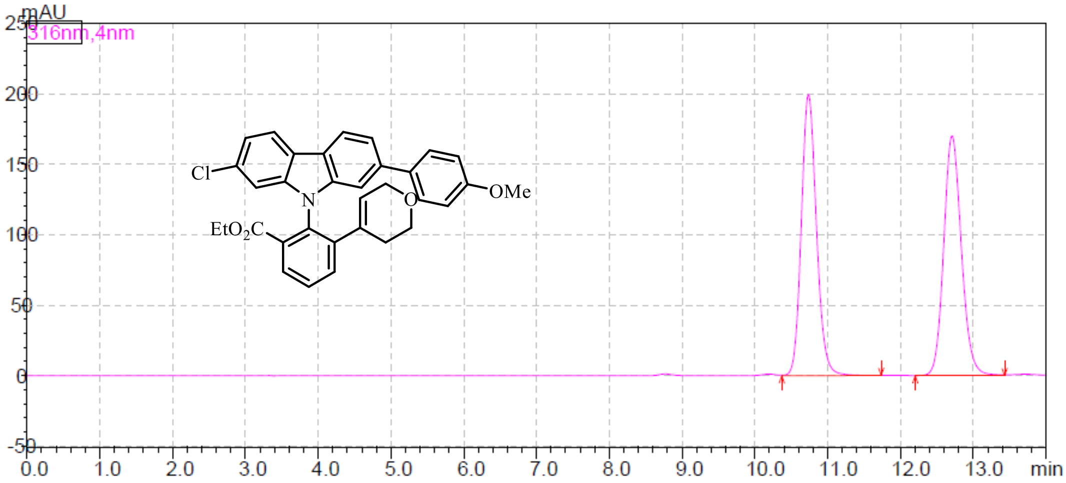

<Peak Table>

PDA Ch1 316nm

Peak#	Ret. Time	Area	Height	Peak Start	Peak End	Area%
1	10.734	2881706	199256	10.368	11.733	50.094
2	12.707	2870888	170005	12.203	13.429	49.906
Total		5752594	369261			100.000

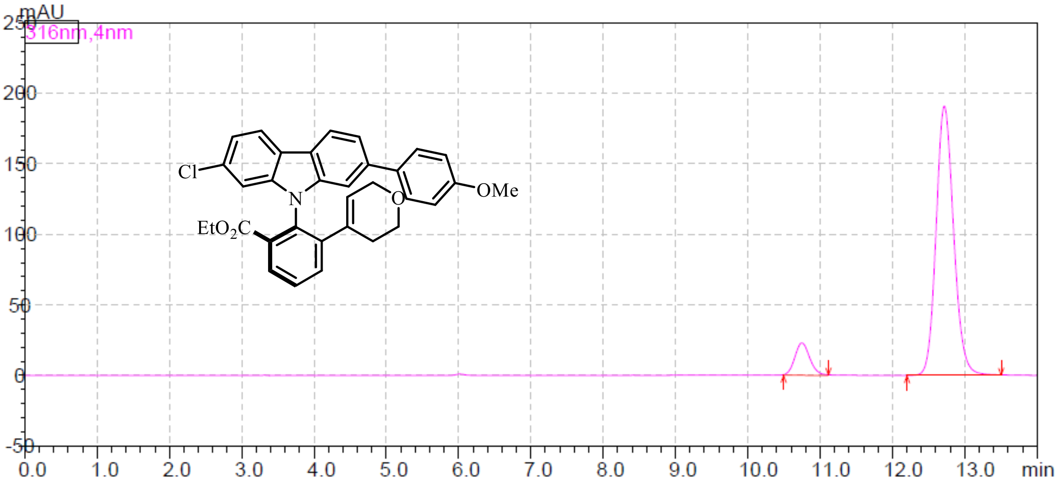

<Peak Table>

PDA Ch1 316nm

Peak#	Ret. Time	Area	Height	Peak Start	Peak End	Area%
1	10.747	331871	22987	10.496	11.115	9.309
2	12.718	3233257	190948	12.203	13.504	90.691
Total		3565129	213935			100.000

27

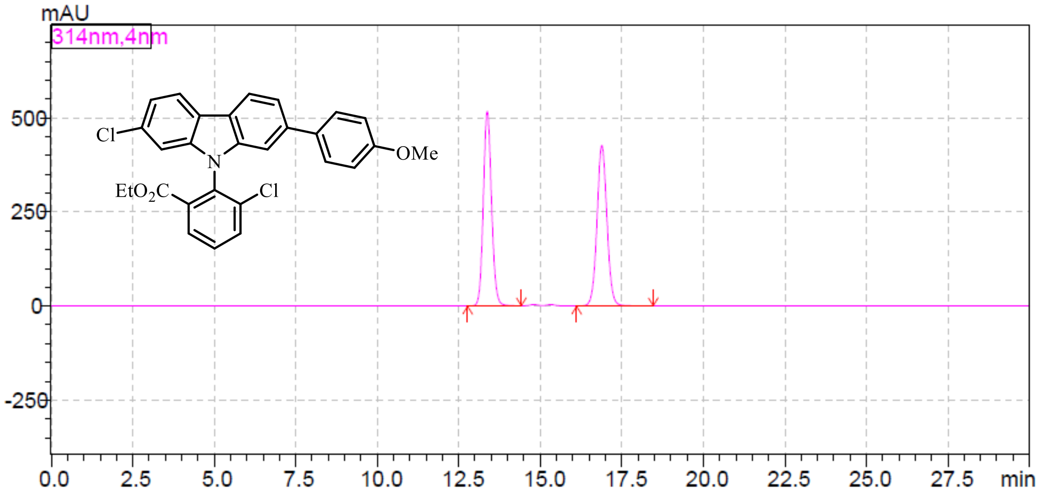

<Peak Table>

PDA Ch1 314nm

Peak#	Ret. Time	Area	Height	Peak Start	Peak End	Area%
1	13.374	9100278	517696	12.768	14.421	50.013
2	16.889	9095446	427036	16.117	18.464	49.987
Total		18195724	944732			100.000

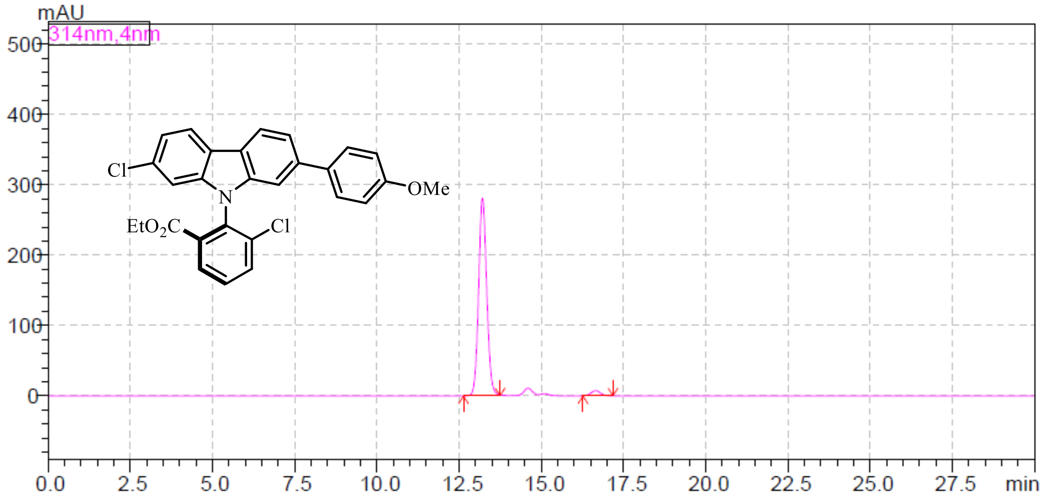

<Peak Table>

PDA Ch1 314nm

Peak#	Ret. Time	Area	Height	Peak Start	Peak End	Area%
1	13.213	4849843	281182	12.629	13.749	96.990
2	16.653	150535	7313	16.267	17.184	3.010
Total		5000378	288495			100.000

SI-**28**

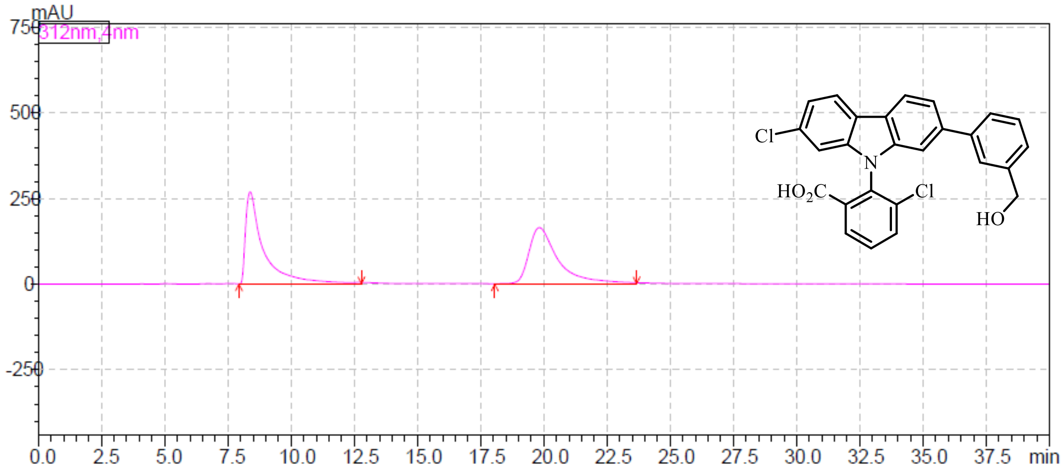

<Peak Table>

PDA Ch1 312nm

Peak#	Ret. Time	Area	Peak Start	Peak End	Area%
1	8.381	13584265	7.936	12.779	50.338
2	19.822	13401938	18.059	23.669	49.662
Total		26986203			100.000

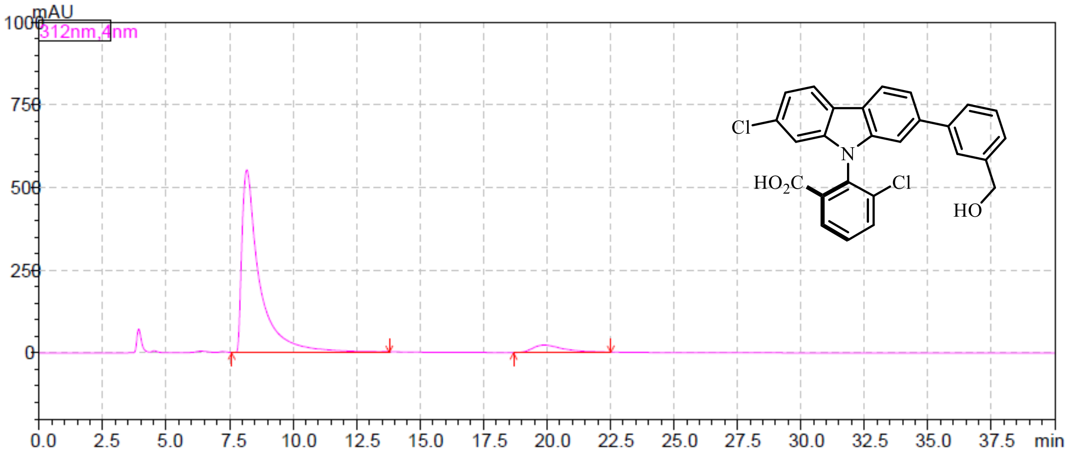

<Peak Table>

PDA Ch1 312nm

Peak#	Ret. Time	Area	Height	Peak Start	Peak End	Area%
1	8.182	27161552	553333	7.584	13.792	93.000
2	19.895	2044298	22718	18.688	22.517	7.000
Total		29205850	576051			100.000

29

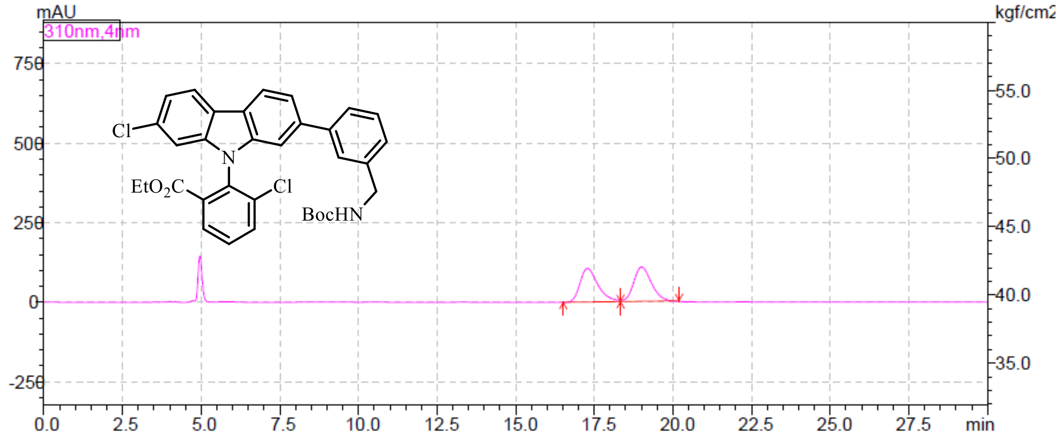

<Peak Table>

PDA Ch1 310nm

Peak#	Ret. Time	Area	Height	Peak Start	Peak End	Area%
1	17.283	4182376	105614	16.512	18.336	50.042
2	19.003	4175393	107729	18.336	20.192	49.958
Total		8357769	213343			100.000

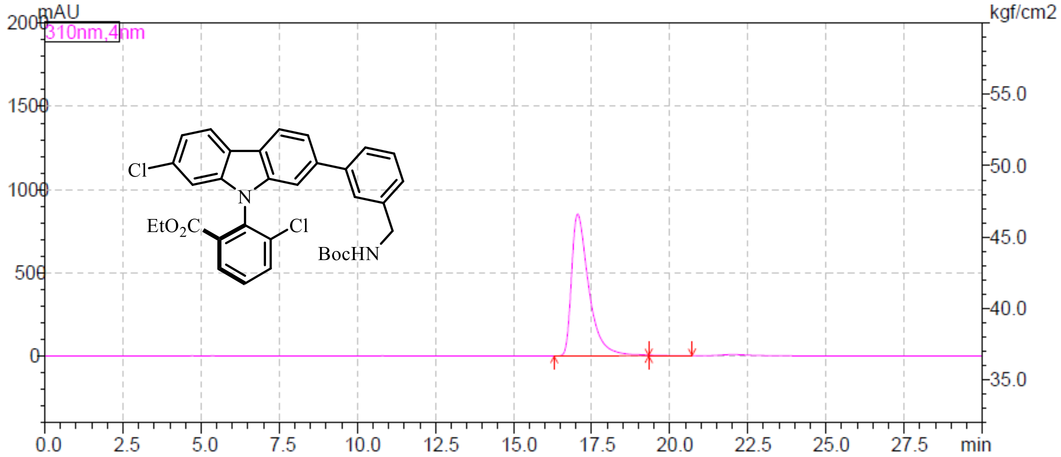

<Peak Table>

PDA Ch1 310nm

Peak#	Ret. Time	Area	Height	Peak Start	Peak End	Area%
1	17.054	32921211	852241	16.299	19.328	99.695
2	19.344	100659	3674	19.328	20.715	0.305
Total		33021870	855914			100.000

30

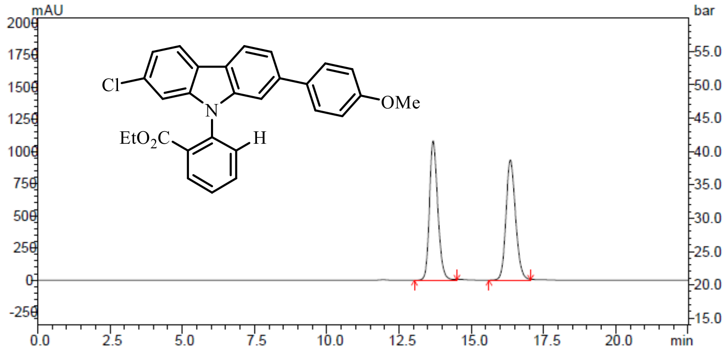

<Peak Table>

PDA Ch1 316nm

Peak#	Ret. Time	Area	Peak Start	Peak End	Area%
1	13.678	21715937	13.035	14.501	50.153
2	16.352	21583724	15.616	17.051	49.847
Total		43299662			100.000

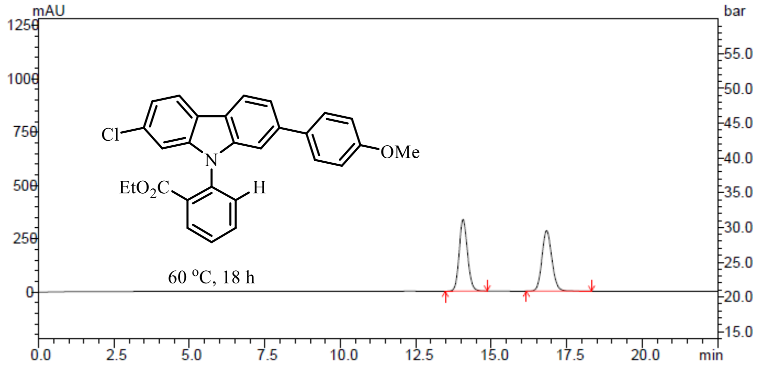

<Peak Table>

PDA Ch1 316nm

Peak#	Ret. Time	Area	Peak Start	Peak End	Area%
1	14.063	8241638	13.461	15.632	50.054
2	16.832	8224011	16.139	18.443	49.946
Total		16465649			100.000

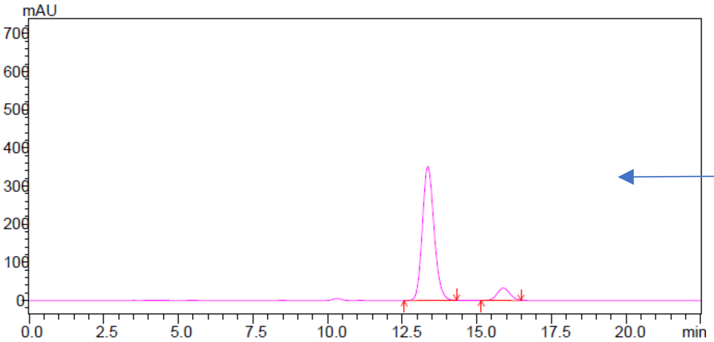

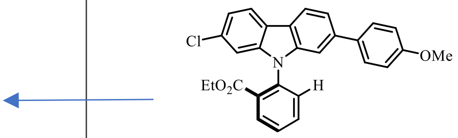

40 °C, 1 h

<Peak Table>

PDA Ch1 316nm

Peak#	Ret. Time	Area	Peak Start	Peak End	Area%
1	13.362	9630556	12.565	14.325	90.936
2	15.891	959871	15.147	16.480	9.064
Total		10590426			100.000

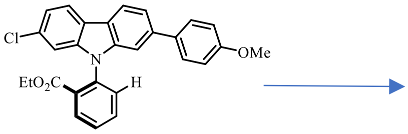

40 °C, 20 h

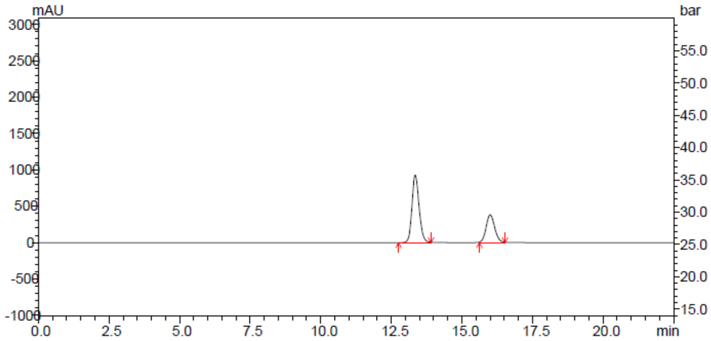

<Peak Table>

PDA Ch1 316nm

Peak#	Ret. Time	Area	Peak Start	Peak End	Area%
1	13.345	16342502	12.757	13.909	66.768
2	16.000	8133932	15.621	16.528	33.232
Total		24476434			100.000

31

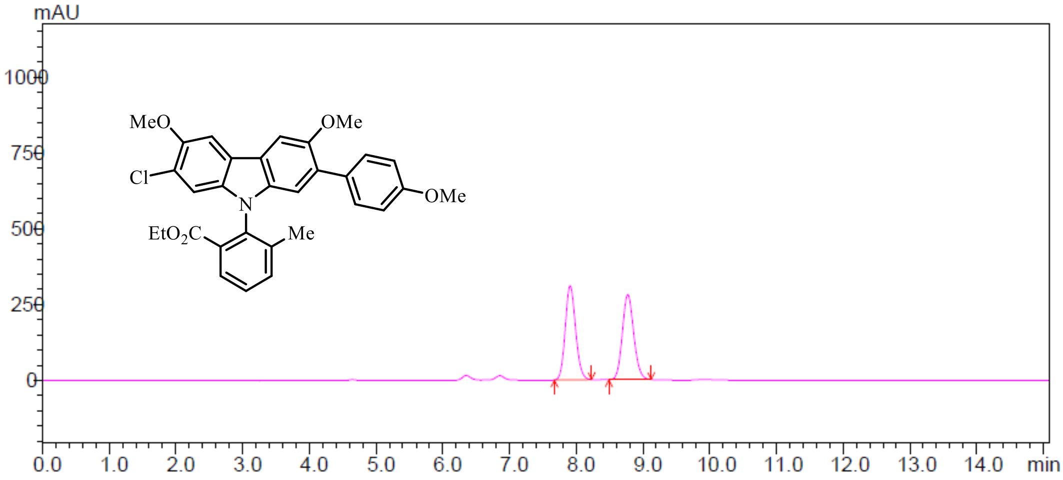

<Peak Table>

PDA Ch1 272nm

Peak#	Ret. Time	Area	Height	Peak Start	Peak End	Area%
1	7.909	3356699	310664	7.675	8.229	50.061
2	8.773	3348538	280678	8.491	9.125	49.939
Total		6705237	591342			100.000

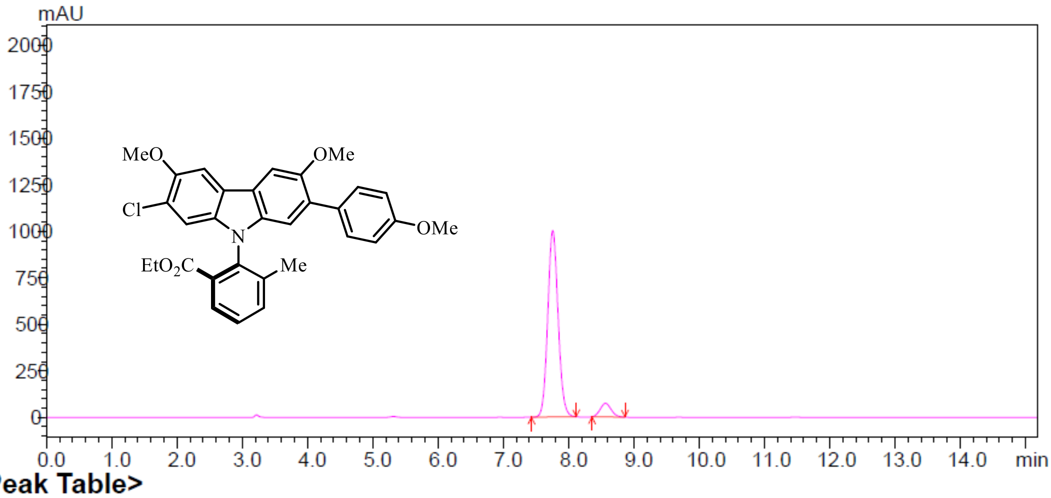

<Peak Table>

PDA Ch1 272nm

Peak#	Ret. Time	Area	Height	Peak Start	Peak End	Area%
1	7.757	11039585	1001433	7.429	8.117	92.817
2	8.565	854313	72908	8.357	8.864	7.183
Total		11893897	1074341			100.000

32

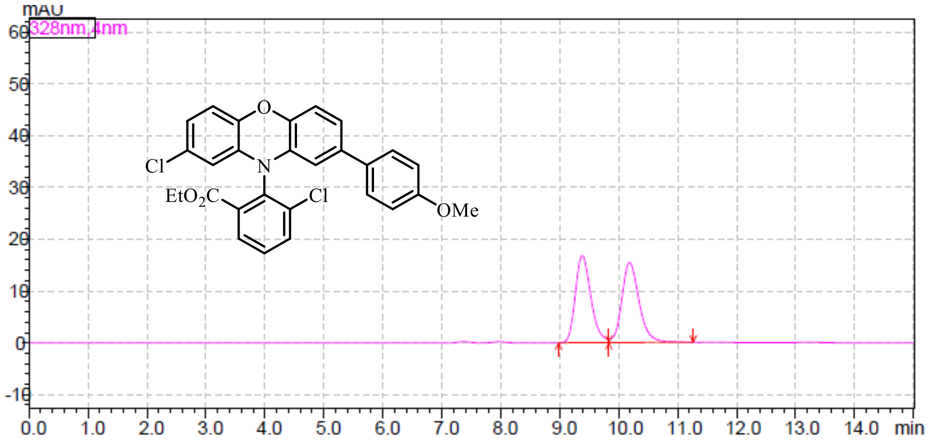

<Peak Table>

PDA Ch1 328nm

Peak#	Ret. Time	Area	Height	Peak Start	Peak End	Area%
1	9.383	308749	16823	8.981	9.835	49.400
2	10.185	316253	15462	9.835	11.264	50.600
Total		625002	32285			100.000

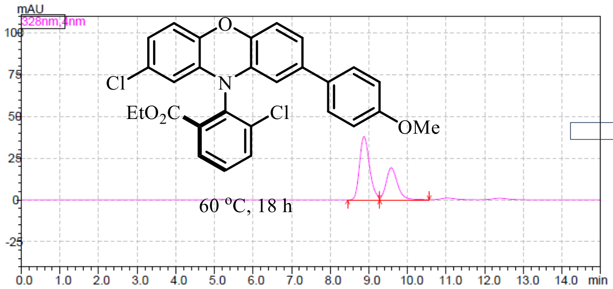

<Peak Table>

PDA Ch1 328nm

Peak#	Ret. Time	Area	Height	Peak Start	Peak End	Area%
1	8.875	686463	37864	8.448	9.280	63.521
2	9.579	394220	19257	9.280	10.560	36.479
Total		1080684	57121			100.000

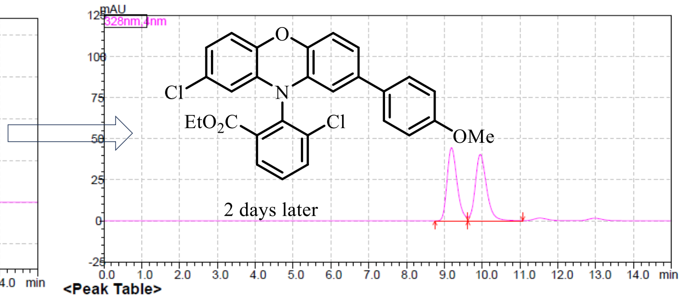

<Peak Table>

PDA Ch1 328nm

Peak#	Ret. Time	Area	Height	Peak Start	Peak End	Area%
1	9.188	819369	44526	8.747	9.621	49.367
2	9.948	840388	40432	9.621	11.072	50.633
Total		1659757	84959			100.000

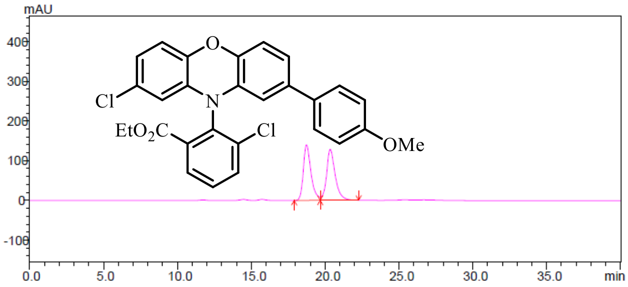

<Peak Table>

PDA Ch1 328nm

Peak#	Ret. Time	Area	Peak Start	Peak End	Area%
1	18.741	5053567	17.909	19.669	49.142
2	20.347	5230006	19.669	22.272	50.858
Total		10283573			100.000

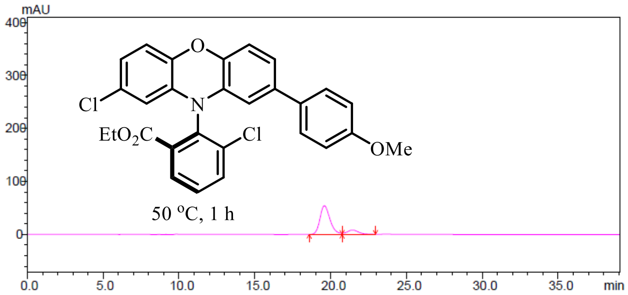

<Peak Table>

PDA Ch1 328nm

Peak#	Ret. Time	Area	Peak Start	Peak End	Area%
1	19.587	2497516	18.581	20.757	85.513
2	21.444	423117	20.757	22.955	14.487
Total		2920633			100.000

33

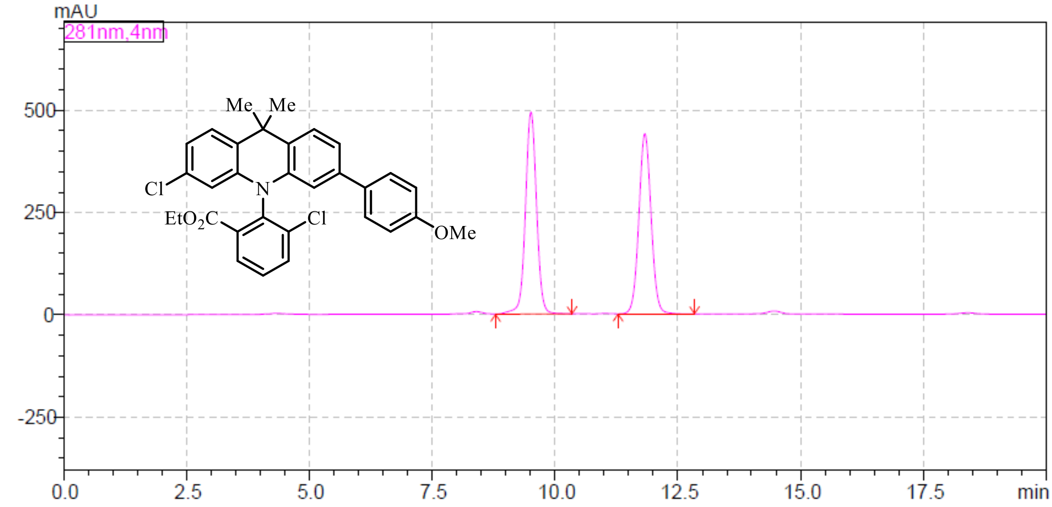

<Peak Table>

PDA Ch1 281nm

Peak#	Ret. Time	Area	Height	Peak Start	Peak End	Area%
1	9.511	7805677	494028	8.789	10.347	50.489
2	11.832	7654373	440559	11.285	12.843	49.511
Total		15460051	934586			100.000

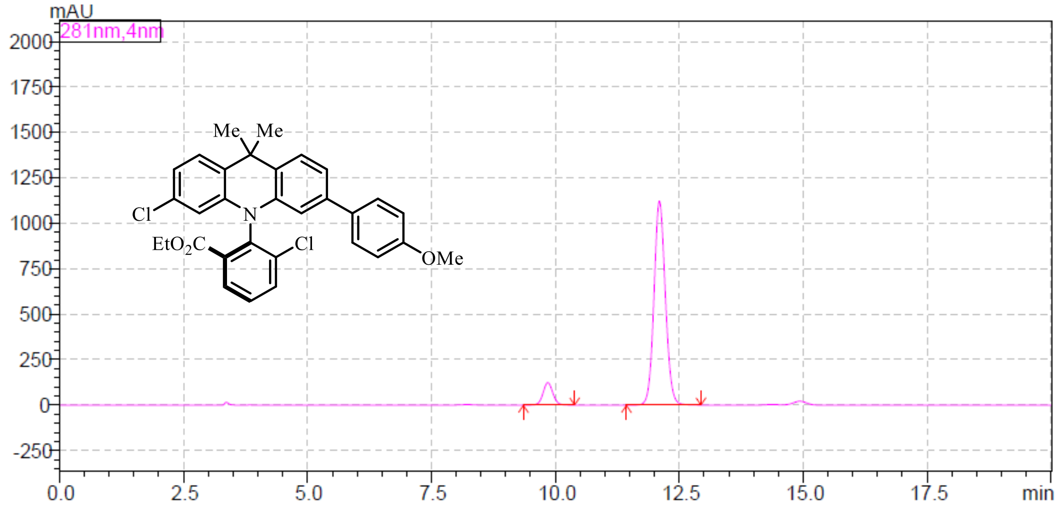

<Peak Table>

PDA Ch1 281nm

Peak#	Ret. Time	Area	Height	Peak Start	Peak End	Area%
1	9.849	1719993	124582	9.355	10.389	8.727
2	12.101	17987872	1122137	11.435	12.939	91.273
Total		19707865	1246719			100.000

35

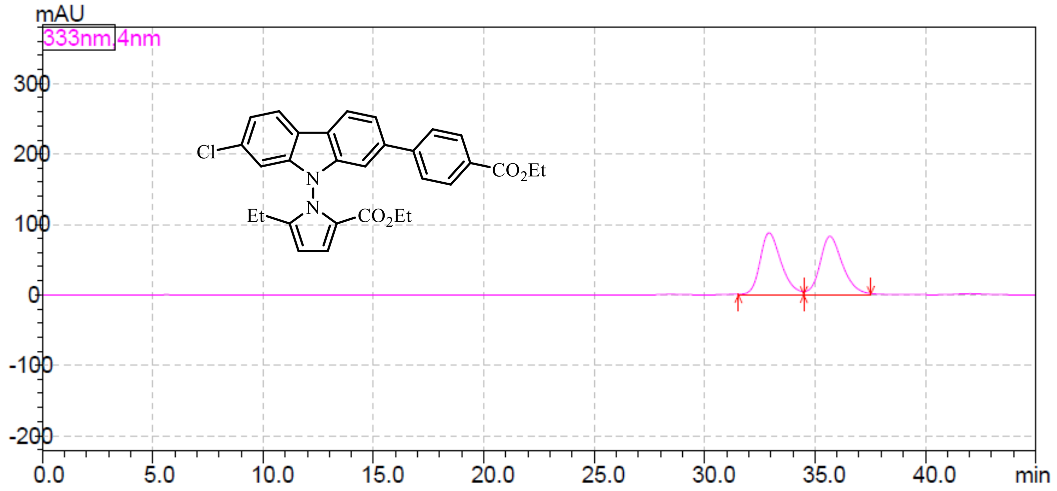

<Peak Table>

PDA Ch1 333nm

Peak#	Ret. Time	Area	Height	Peak Start	Peak End	Area%
1	32.920	5829366	87779	31.531	34.496	49.740
2	35.678	5890368	82911	34.496	37.525	50.260
Total		11719734	170690			100.000

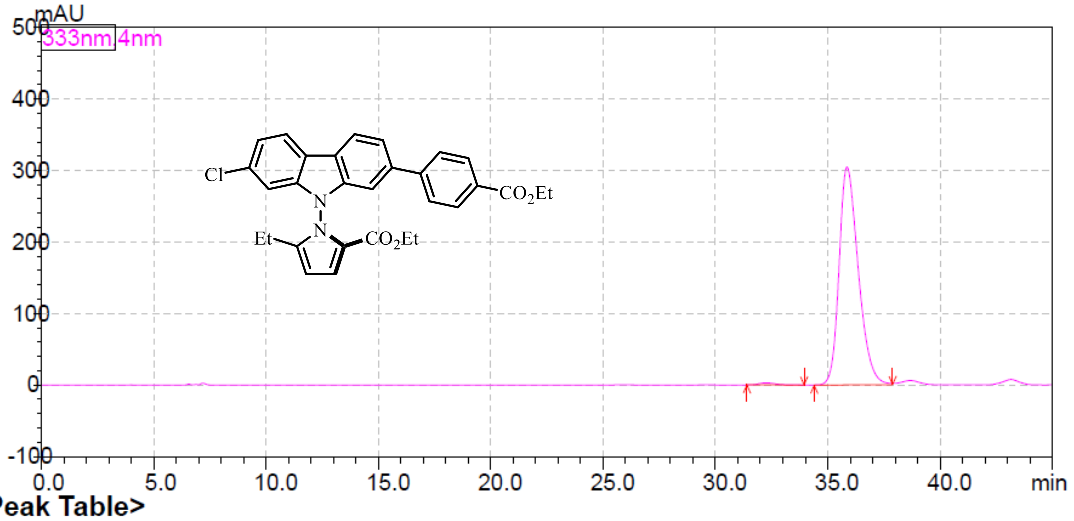

<Peak Table>

PDA Ch1 333nm

Peak#	Ret. Time	Area	Height	Peak Start	Peak End	Area%
1	32.249	146546	2745	31.403	33.952	0.803
2	35.859	18111856	304570	34.400	37.888	99.197
Total		18258402	307315			100.000

36

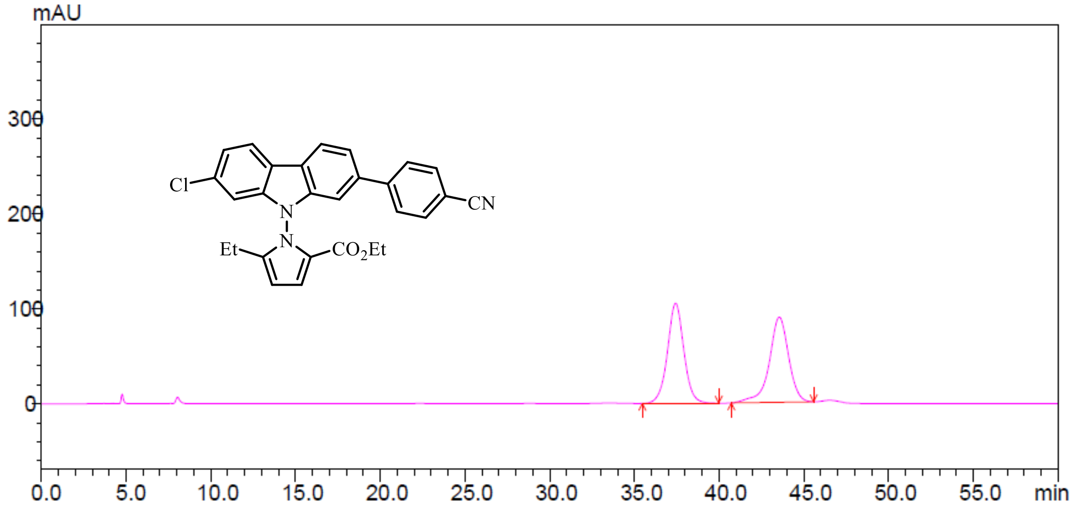

<Peak Table>

PDA Ch1 315nm

Peak#	Ret. Time	Area	Peak Start	Peak End	Area%
1	37.430	7093639	35.467	39.979	49.863
2	43.548	7132569	40.715	45.579	50.137
Total		14226208			100.000

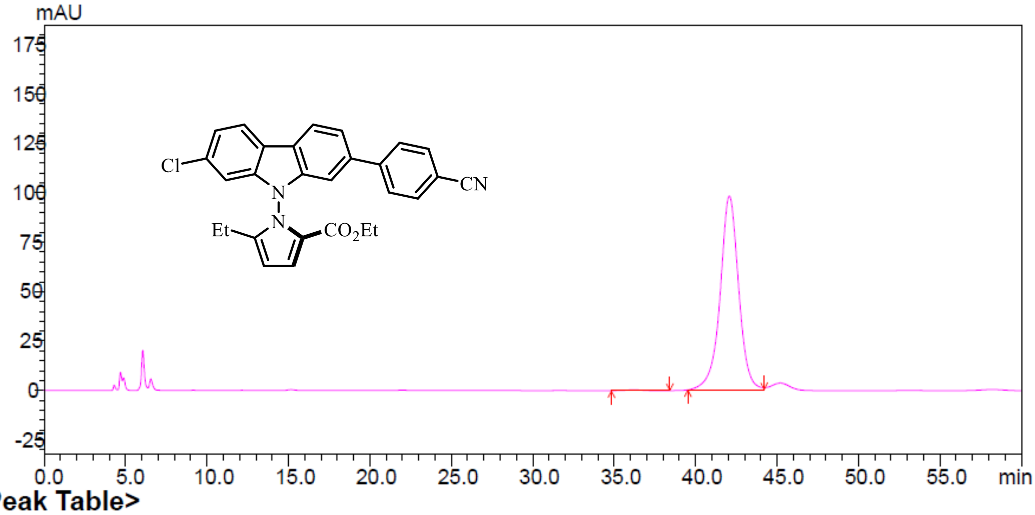

<Peak Table>

PDA Ch1 315nm

Peak#	Ret. Time	Area	Peak Start	Peak End	Area%
1	36.215	40060	34.827	38.411	0.514
2	42.073	7752871	39.520	44.213	99.486
Total		7792931			100.000

37

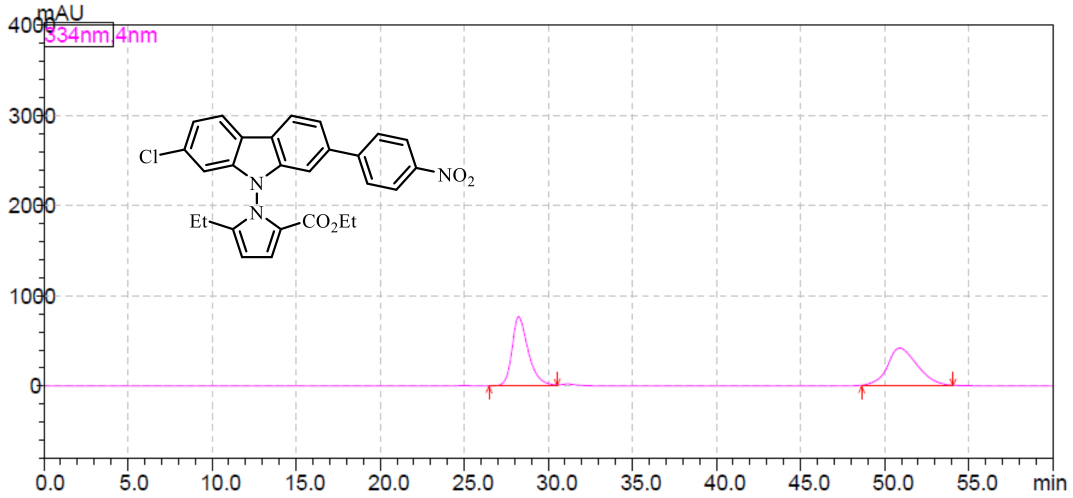

<Peak Table>

PDA Ch1 334nm

Peak#	Ret. Time	Area	Height	Peak Start	Peak End	Area%
1	28.241	51938736	768254	26.475	30.528	49.518
2	50.901	52949968	420415	48.640	54.069	50.482
Total		104888705	1188669			100.000

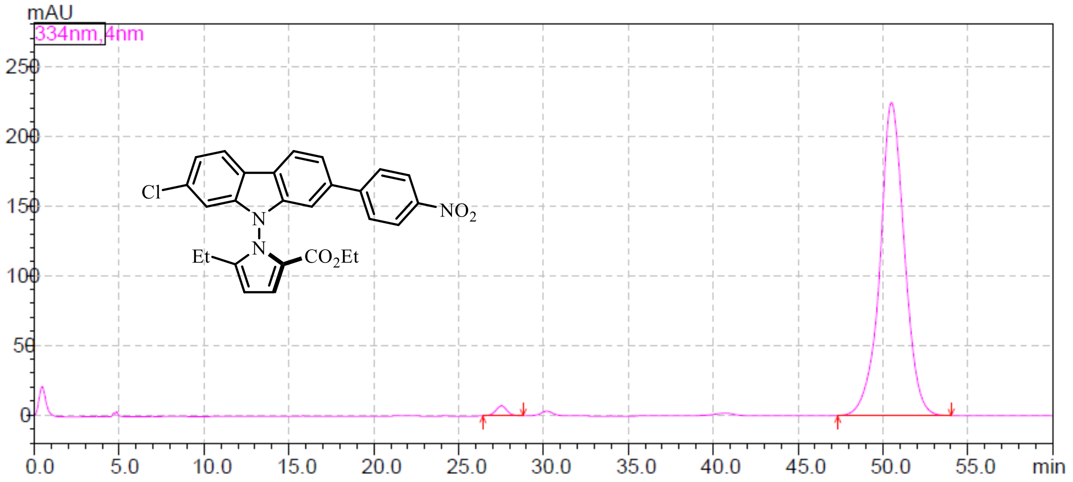

<Peak Table>

PDA Ch1 334nm

Peak#	Ret. Time	Area	Height	Peak Start	Peak End	Area%
1	27.522	340401	7267	26.421	28.800	1.488
2	50.509	22538969	224454	47.339	54.027	98.512
Total		22879370	231721			100.000

38

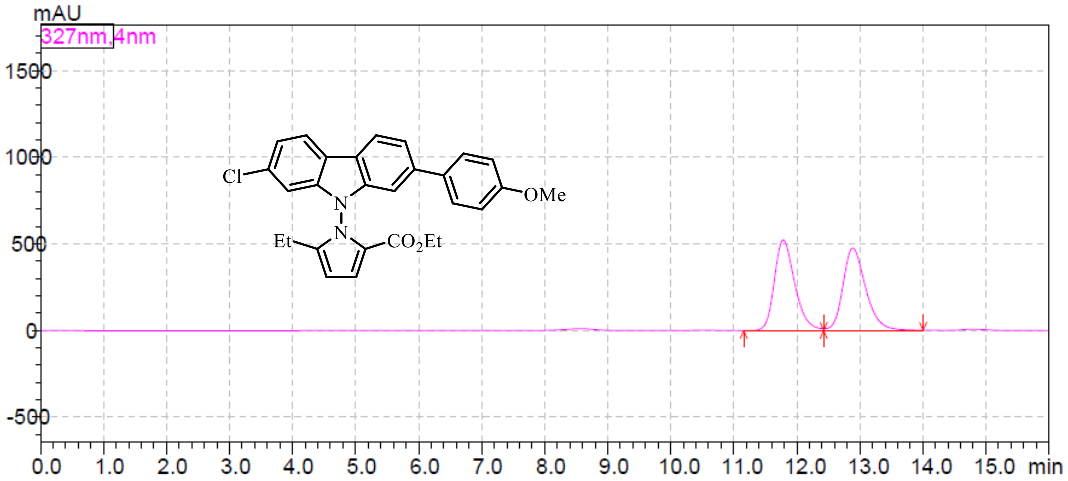

<Peak Table>

PDA Ch1 327nm

Peak#	Ret. Time	Area	Height	Peak Start	Peak End	Area%
1	11.785	11776995	522590	11.157	12.427	49.524
2	12.889	12003339	476439	12.427	14.005	50.476
Total		23780334	999029			100.000

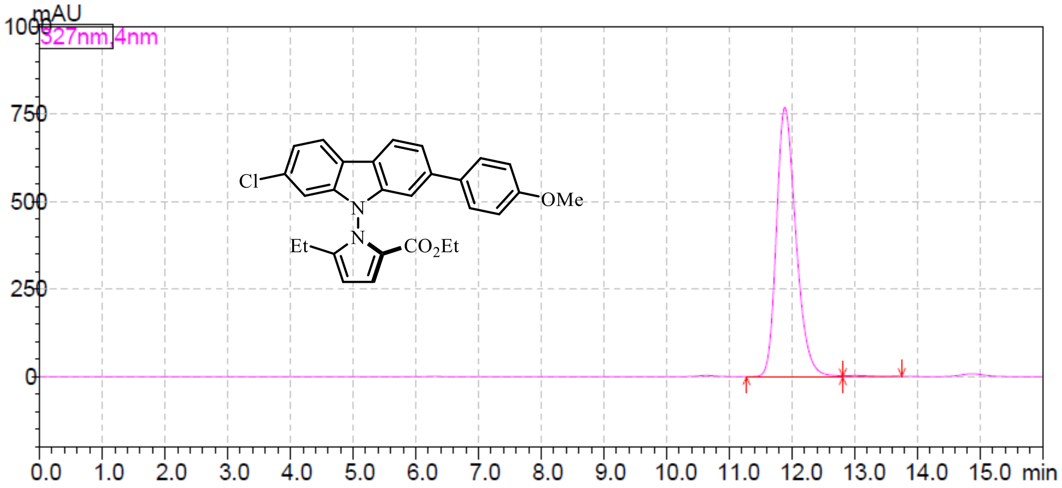

<Peak Table>

PDA Ch1 327nm

Peak#	Ret. Time	Area	Height	Peak Start	Peak End	Area%
1	11.886	16608517	769134	11.275	12.811	99.548
2	13.002	75376	2878	12.811	13.749	0.452
Total		16683893	772012			100.000

39

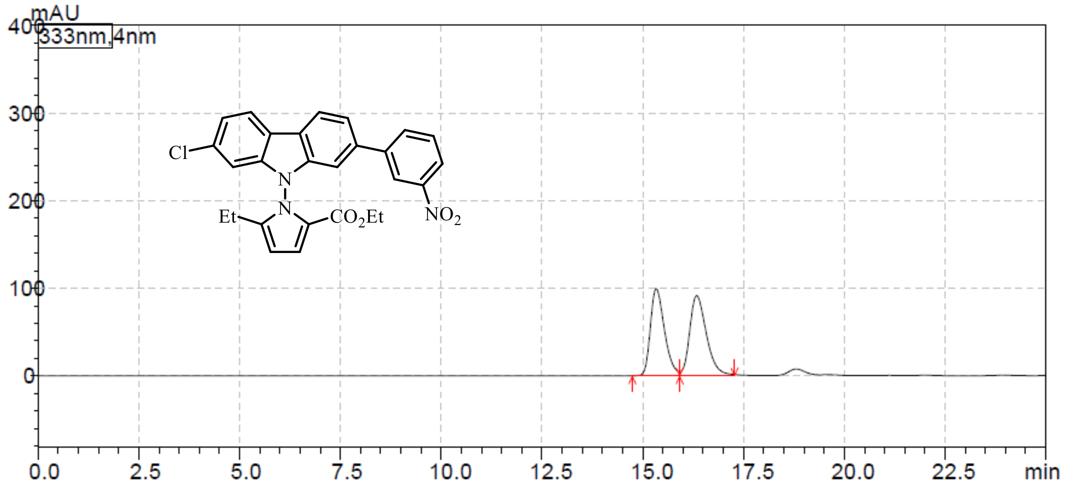

<Peak Table>

PDA Ch1 333nm

Peak#	Ret. Time	Area	Height	Peak Start	Peak End	Area%
1	15.335	2365801	98749	14.805	15.915	49.510
2	16.339	2412660	89389	15.915	16.800	50.490
Total		4778461	188139			100.000

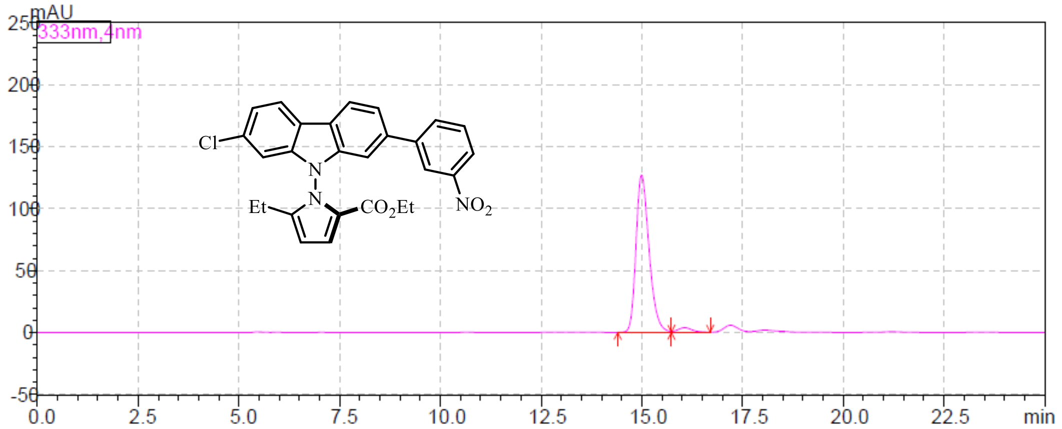

<Peak Table>

PDA Ch1 333nm

Peak#	Ret. Time	Area	Height	Peak Start	Peak End	Area%
1	14.993	2866519	126967	14.400	15.733	96.778
2	16.061	95434	3797	15.733	16.693	3.222
Total		2961953	130764			100.000

40

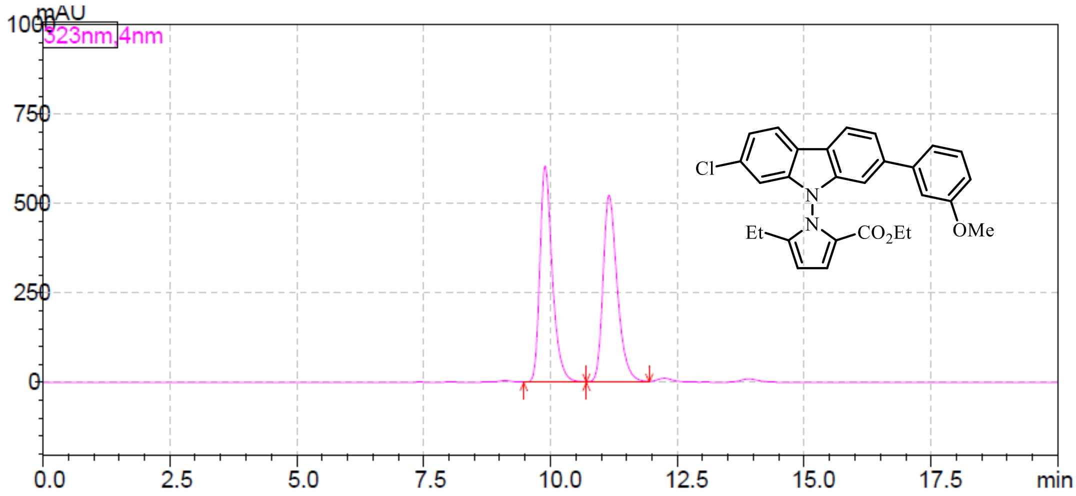

<Peak Table>

PDA Ch1 323nm

Peak#	Ret. Time	Area	Peak Start	Peak End	Area%
1	9.895	10321602	9.472	10.709	50.115
2	11.155	10274284	10.709	11.947	49.885
Total		20595886			100.000

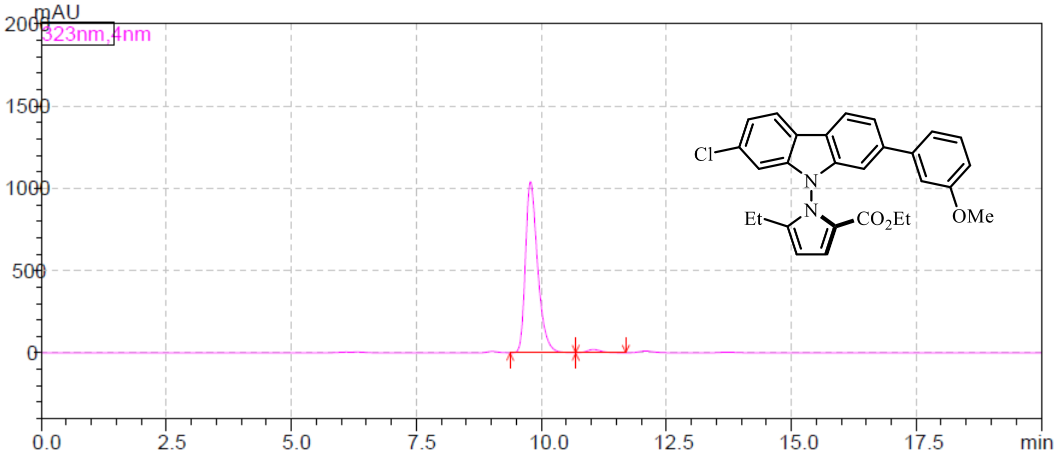

<Peak Table>

PDA Ch1 323nm

Peak#	Ret. Time	Area	Height	Peak Start	Peak End	Area%
1	9.784	17688330	1039640	9.376	10.688	97.750
2	11.042	407238	20381	10.688	11.691	2.250
Total		18095568	1060021			100.000

41

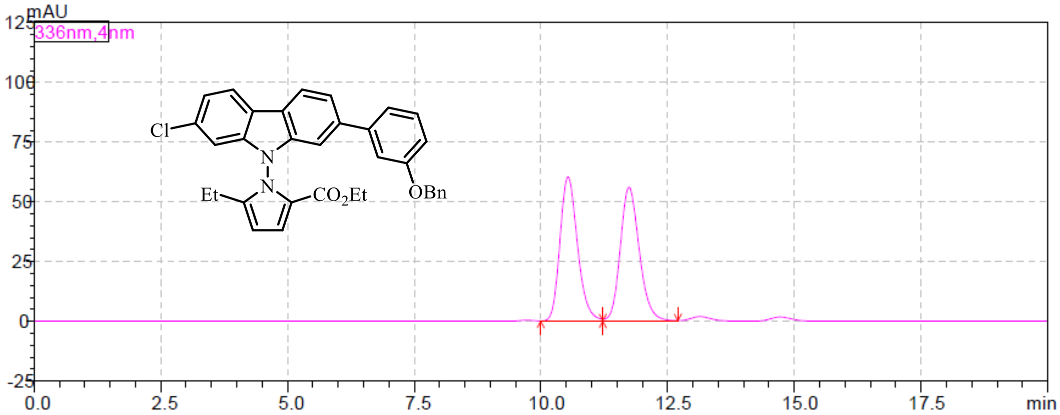

<Peak Table>

PDA Ch1 336nm

Peak#	Ret. Time	Area	Height	Peak Start	Peak End	Area%
1	10.534	1456031	60359	9.995	11.221	49.833
2	11.738	1465795	56028	11.221	12.704	50.167
Total		2921826	116387			100.000

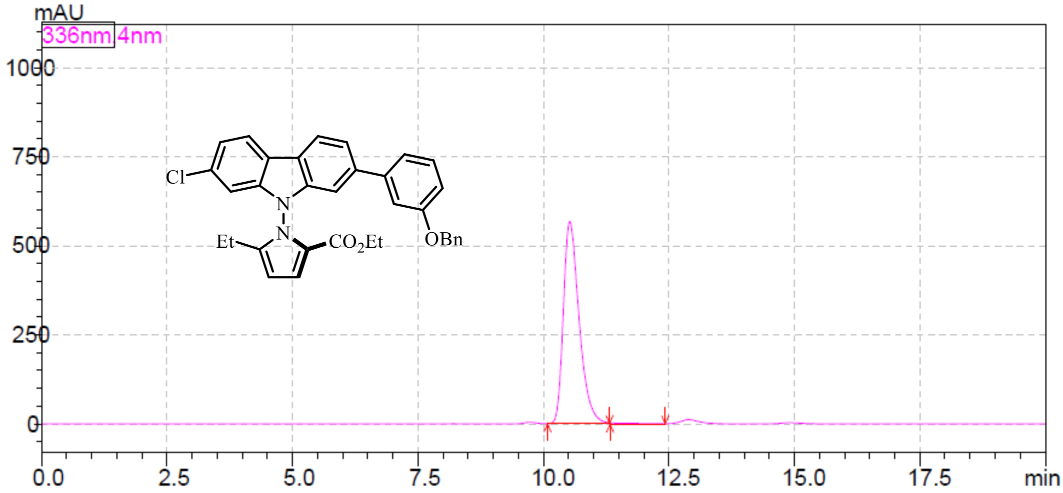

<Peak Table>

PDA Ch1 336nm

Peak#	Ret. Time	Area	Height	Peak Start	Peak End	Area%
1	10.526	12435000	567465	10.080	11.317	99.469
2	11.344	66400	2701	11.328	12.427	0.531
Total		12501401	570167			100.000

42

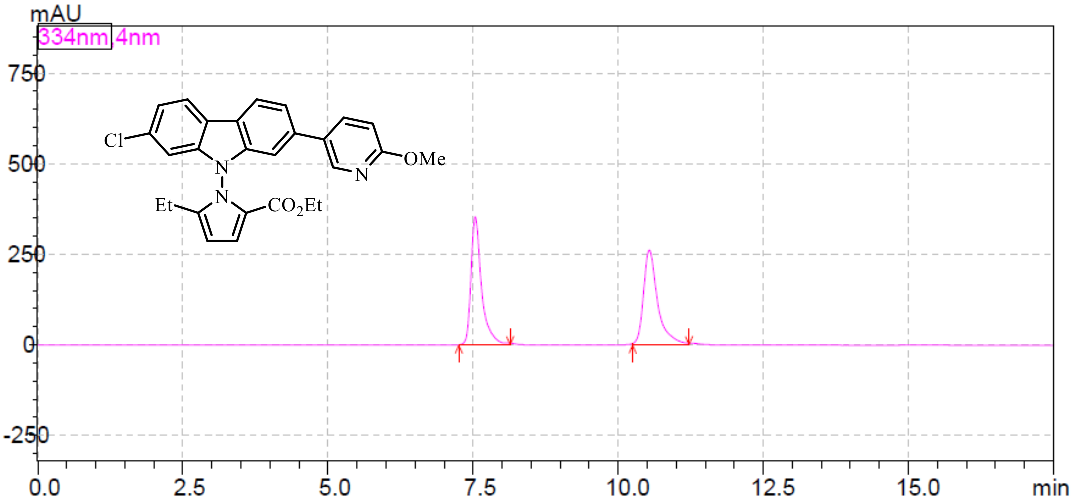

<Peak Table>

PDA Ch1 334nm

Peak#	Ret. Time	Area	Height	Peak Start	Peak End	Area%
1	7.538	4531749	354355	7.253	8.139	50.129
2	10.536	4508381	262315	10.251	11.221	49.871
Total		9040130	616670			100.000

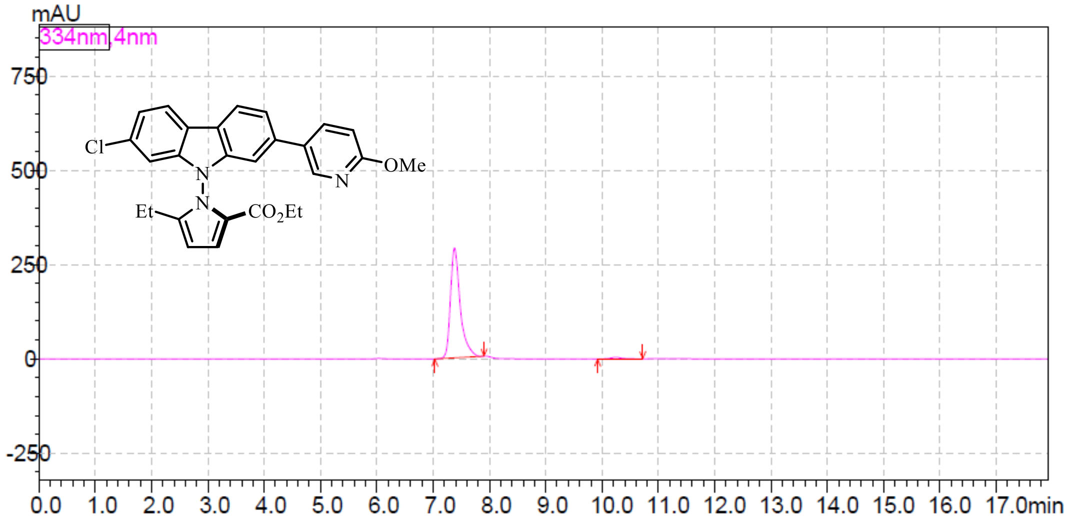

<Peak Table>

PDA Ch1 334nm

Peak#	Ret. Time	Area	Height	Peak Start	Peak End	Area%
1	7.380	3502904	291121	7.029	7.904	98.444
2	10.242	55367	3511	9.920	10.720	1.556
Total		3558271	294632			100.000

43

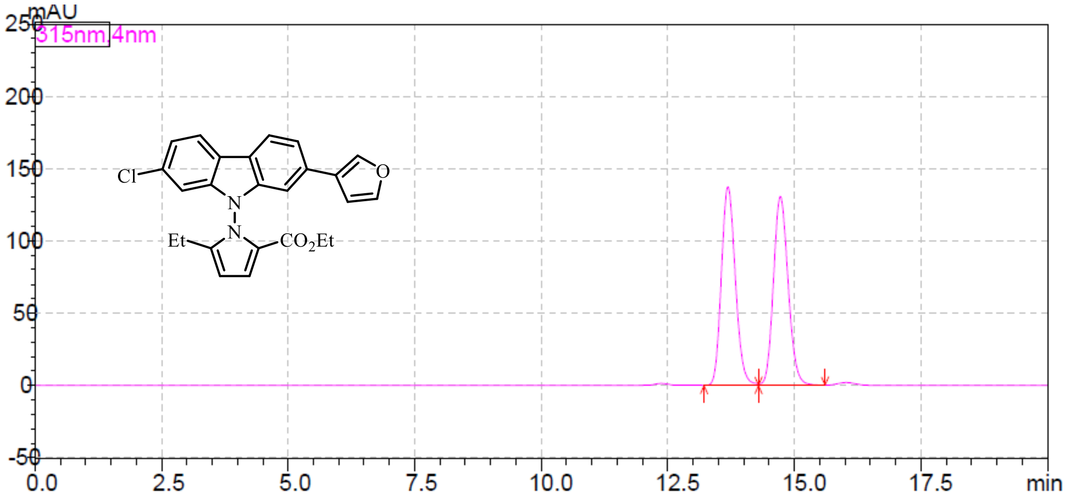

<Peak Table>

PDA Ch1 315nm

Peak#	Ret. Time	Area	Height	Peak Start	Peak End	Area%
1	13.688	2642141	137394	13.216	14.293	49.991
2	14.724	2643139	130650	14.293	15.605	50.009
Total		5285280	268044			100.000

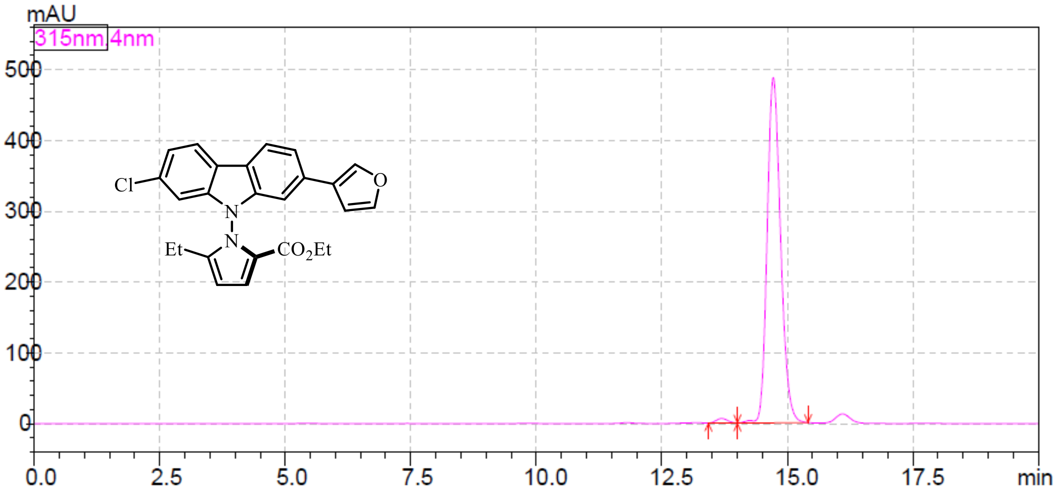

<Peak Table>

PDA Ch1 315nm

Peak#	Ret. Time	Area	Height	Peak Start	Peak End	Area%
1	13.691	99775	6351	13.429	14.005	1.087
2	14.721	9078746	487521	14.005	15.413	98.913
Total		9178521	493872			100.000

44

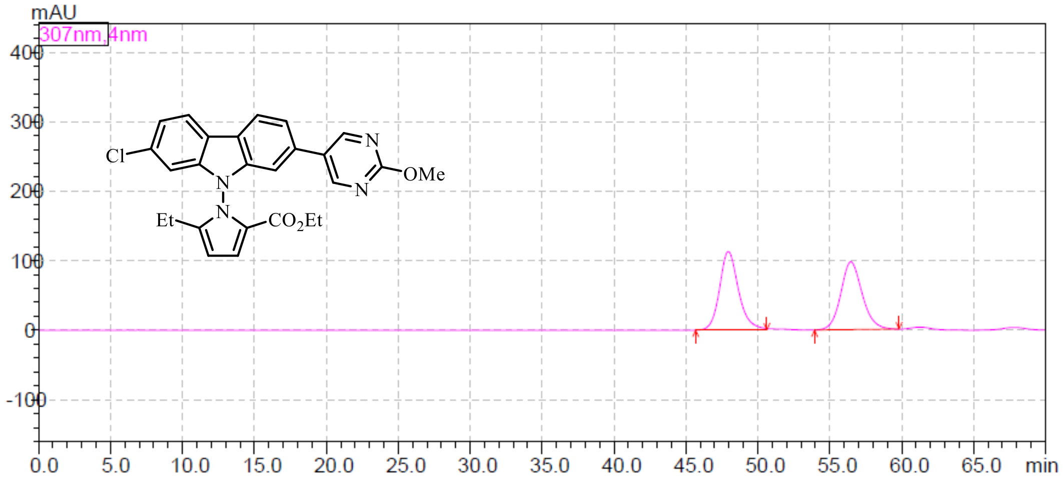

<Peak Table>

PDA Ch1 307nm

Peak#	Ret. Time	Area	Height	Peak Start	Peak End	Area%
1	47.947	10427902	113237	45.675	50.624	50.803
2	56.465	10098393	97483	53.941	59.819	49.197
Total		20526295	210720			100.000

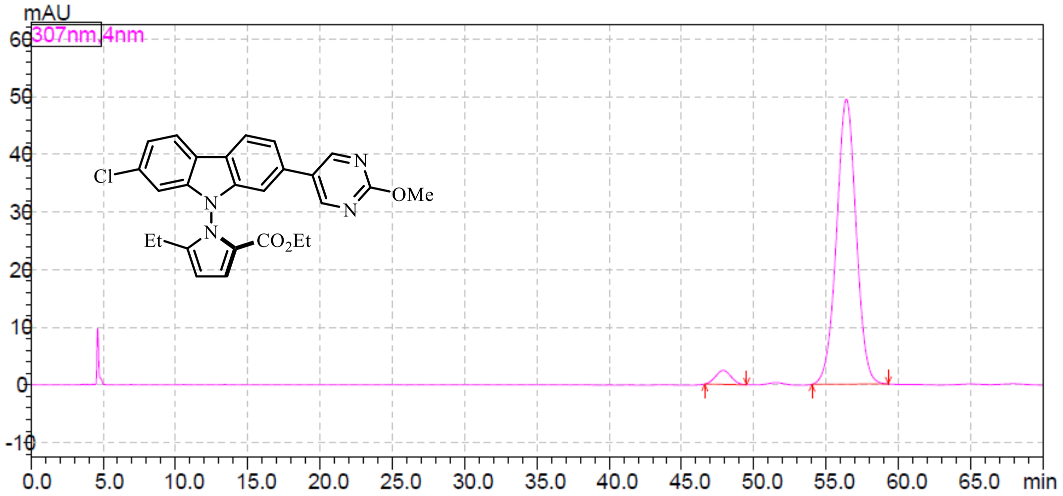

<Peak Table>

PDA Ch1 307nm

Peak#	Ret. Time	Area	Height	Peak Start	Peak End	Area%
1	47.889	182439	2441	46.613	49.515	3.755
2	56.405	4676561	49474	54.027	59.307	96.245
Total		4859001	51914			100.000

45

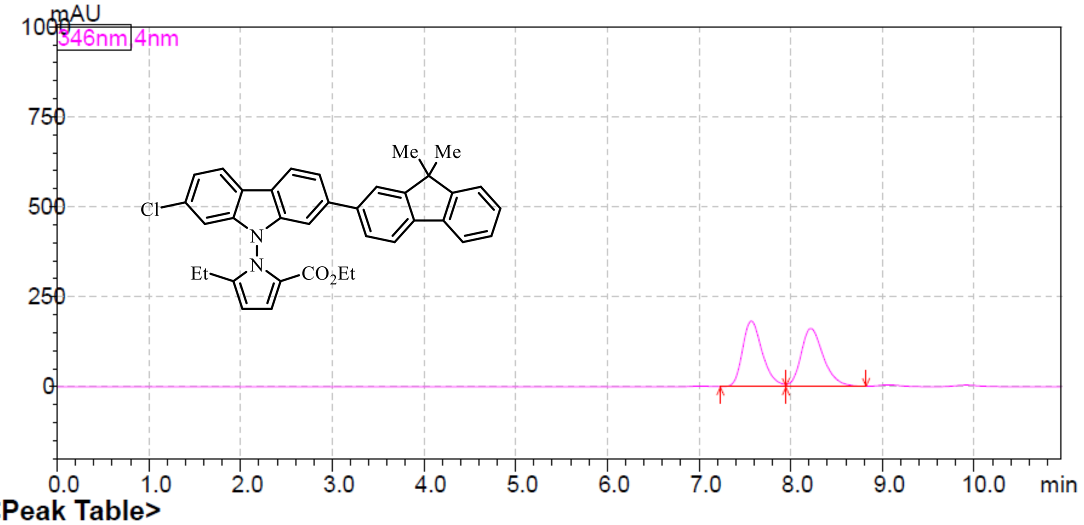

<Peak Table>

PDA Ch1 346nm

Peak#	Ret. Time	Area	Height	Peak Start	Peak End	Area%
1	7.569	2762784	182706	7.232	7.947	49.856
2	8.219	2778712	162168	7.947	8.821	50.144
Total		5541497	344874			100.000

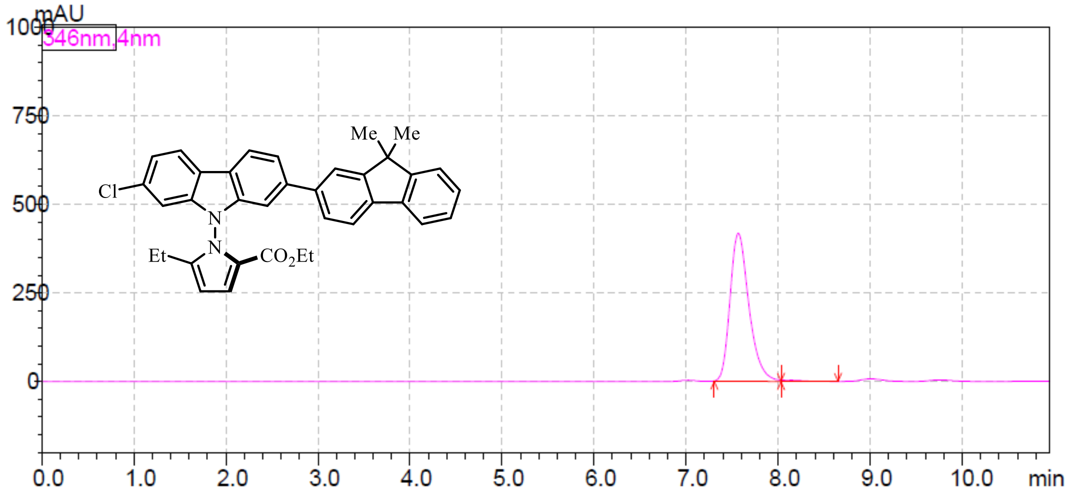

<Peak Table>

PDA Ch1 346nm

Peak#	Ret. Time	Area	Height	Peak Start	Peak End	Area%
1	7.567	5970790	417626	7.307	8.032	99.229
2	8.165	46404	2828	8.032	8.651	0.771
Total		6017195	420453			100.000

46

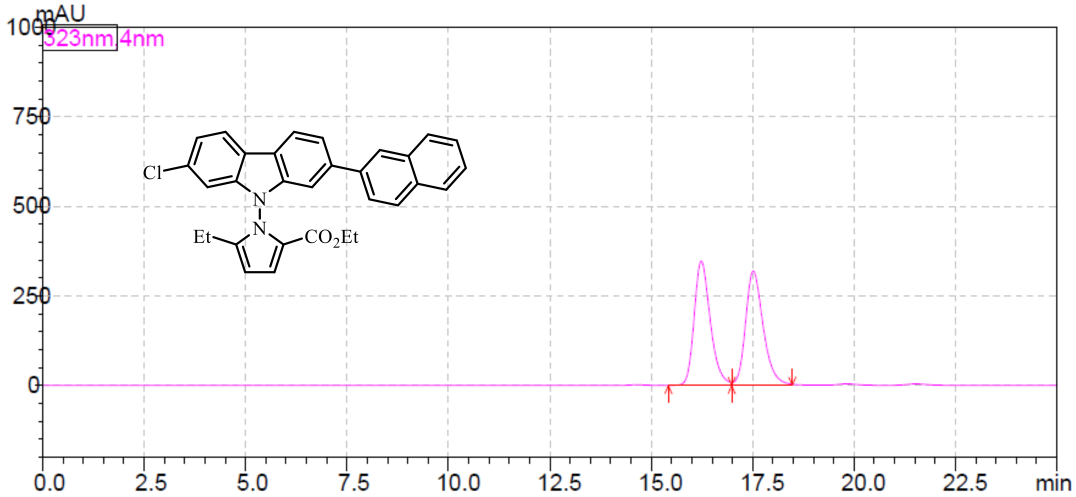

<Peak Table>

PDA Ch1 323nm

Peak#	Ret. Time	Area	Height	Peak Start	Peak End	Area%
1	16.233	9607270	346755	15.424	16.981	49.814
2	17.515	9679196	318902	16.981	18.475	50.186
Total		19286466	665657			100.000

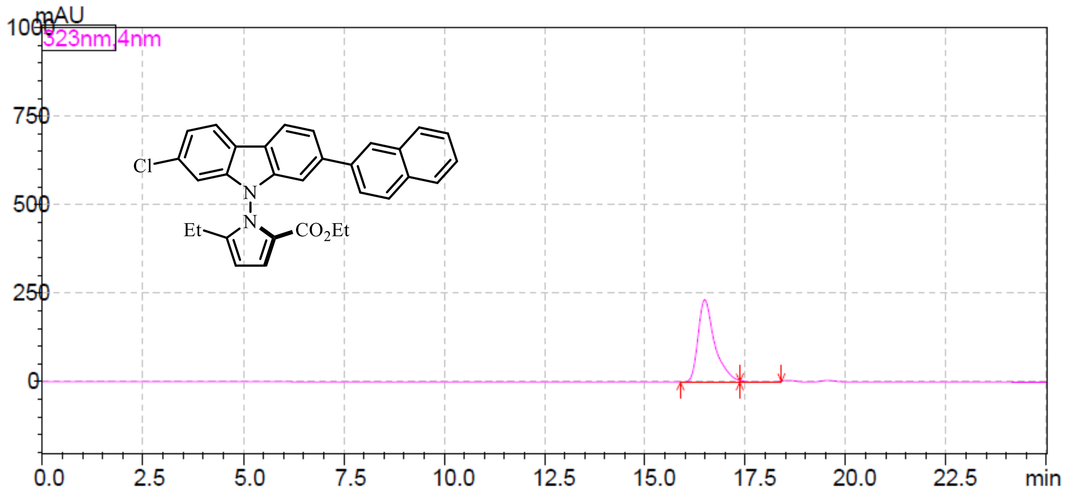

<Peak Table>

PDA Ch1 323nm

Peak#	Ret. Time	Area	Height	Peak Start	Peak End	Area%
1	16.489	6675511	232255	15.883	17.365	99.539
2	17.381	30935	3719	17.365	18.400	0.461
Total		6706446	235974			100.000

47

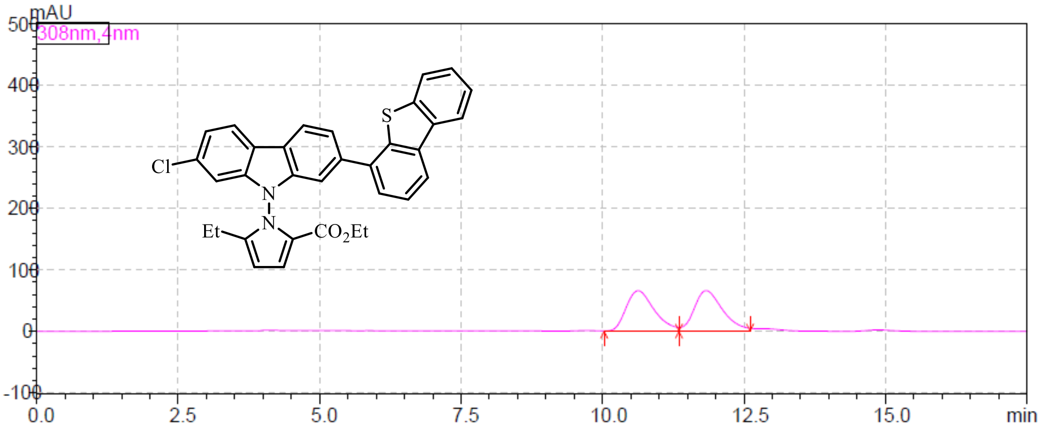

<Peak Table>

PDA Ch1 308nm

Peak#	Ret. Time	Area	Height	Peak Start	Peak End	Area%
1	10.627	2272664	65726	10.027	11.349	49.460
2	11.831	2322255	65977	11.349	12.608	50.540
Total		4594919	131703			100.000

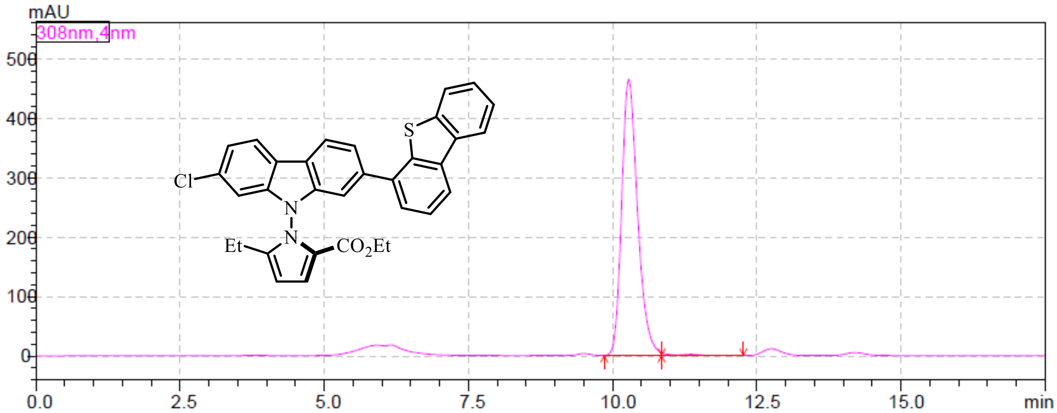

<Peak Table>

PDA Ch1 308nm

Peak#	Ret. Time	Area	Height	Peak Start	Peak End	Area%
1	10.279	8919706	464253	9.856	10.848	99.059
2	10.864	84775	4687	10.848	12.267	0.941
Total		9004481	468940			100.000

48

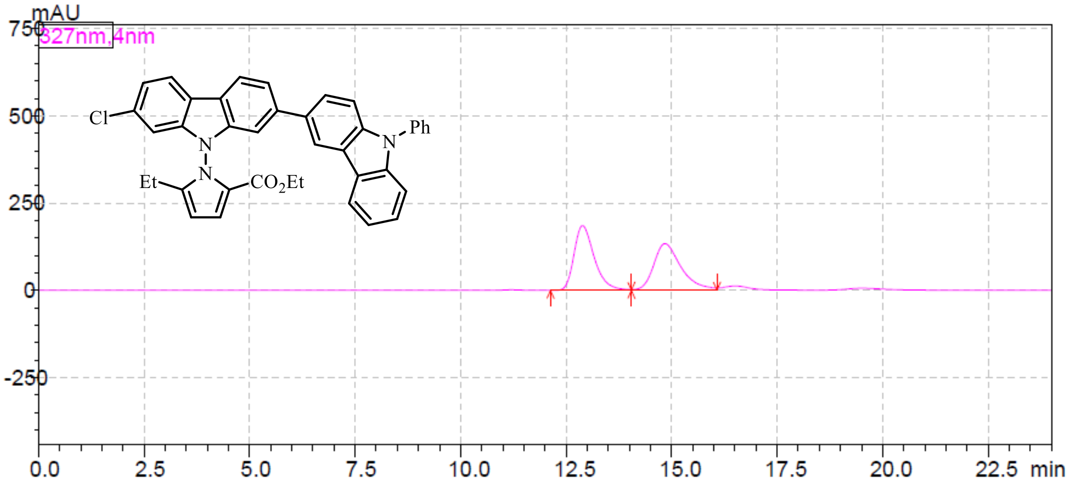

<Peak Table>

PDA Ch1 327nm

Peak#	Ret. Time	Area	Height	Peak Start	Peak End	Area%
1	12.890	6196028	185524	12.128	14.048	50.589
2	14.843	6051674	133789	14.048	16.075	49.411
Total		12247702	319313			100.000

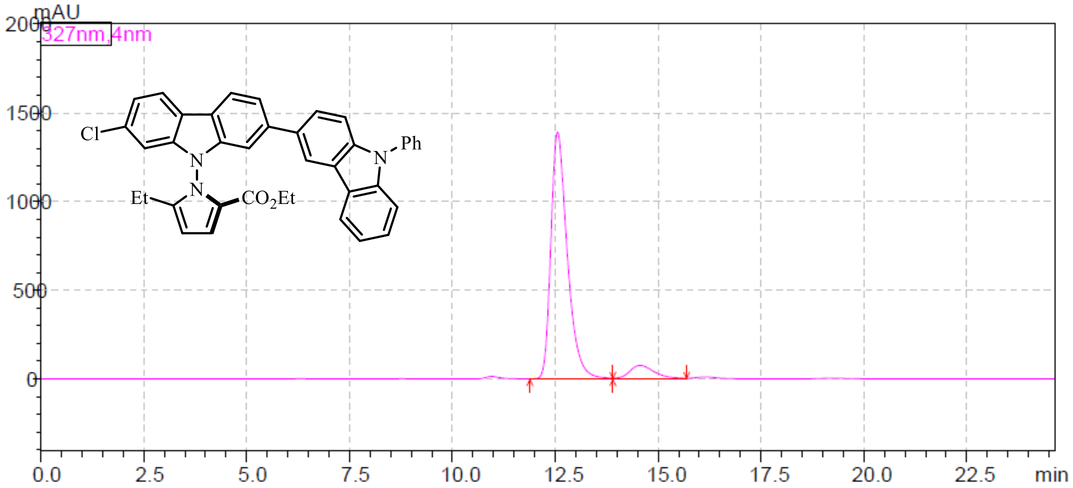

<Peak Table>

PDA Ch1 327nm

Peak#	Ret. Time	Area	Height	Peak Start	Peak End	Area%
1	12.554	38723348	1391035	11.883	13.899	92.052
2	14.560	3343259	77414	13.899	15.691	7.948
Total		42066606	1468449			100.000

49

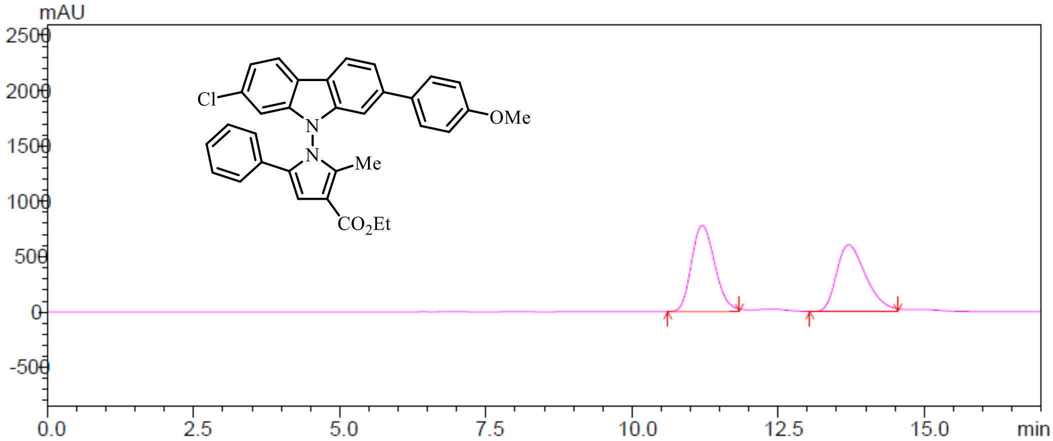

<Peak Table>

PDA Ch1 315nm

Peak#	Ret. Time	Area	Height	Peak Start	Peak End	Area%
1	11.203	21750949	778681	10.613	11.840	50.666
2	13.708	21178961	602891	13.045	14.549	49.334
Total		42929910	1381573			100.000

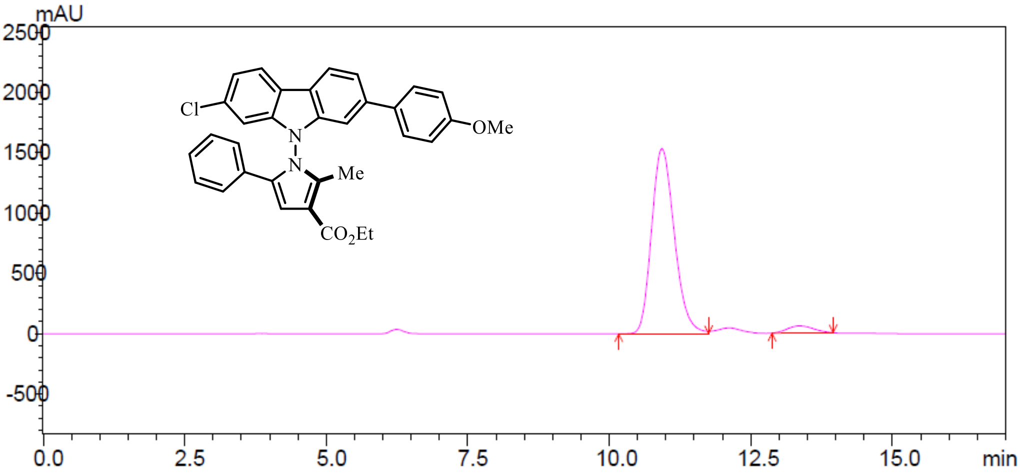

<Peak Table>

PDA Ch1 315nm

Peak#	Ret. Time	Area	Height	Peak Start	Peak End	Area%
1	10.929	43013947	1534182	10.165	11.755	95.884
2	13.363	1846398	58395	12.875	13.952	4.116
Total		44860346	1592577			100.000

50

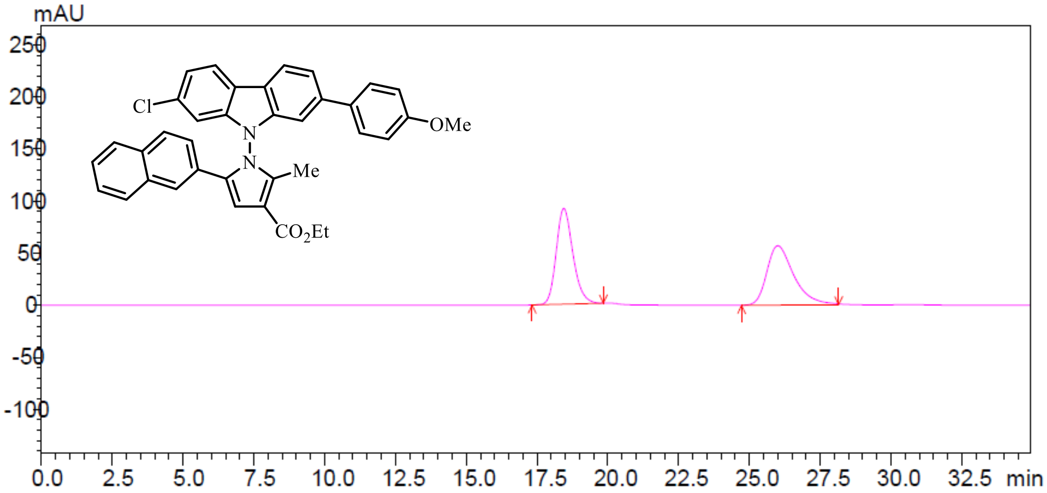

<Peak Table>

PDA Ch1 325nm

Peak#	Ret. Time	Area	Height	Peak Start	Peak End	Area%
1	18.444	3782942	92001	17.323	19.840	49.832
2	26.003	3808450	57095	24.725	28.149	50.168
Total		7591393	149095			100.000

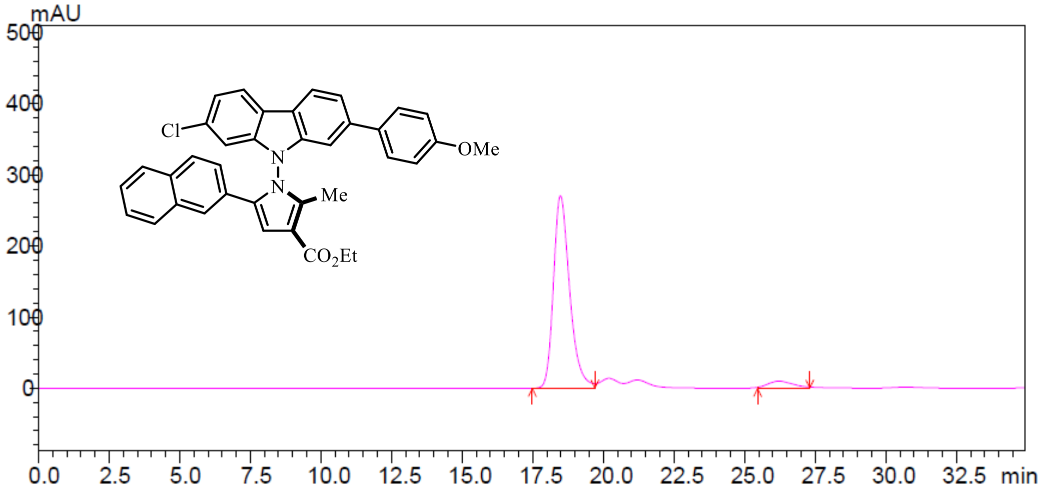

<Peak Table>

PDA Ch1 325nm

Peak#	Ret. Time	Area	Height	Peak Start	Peak End	Area%
1	18.481	10820836	270948	17.483	19.701	94.589
2	26.216	619016	9761	25.461	27.307	5.411
Total		11439851	280710			100.000

51

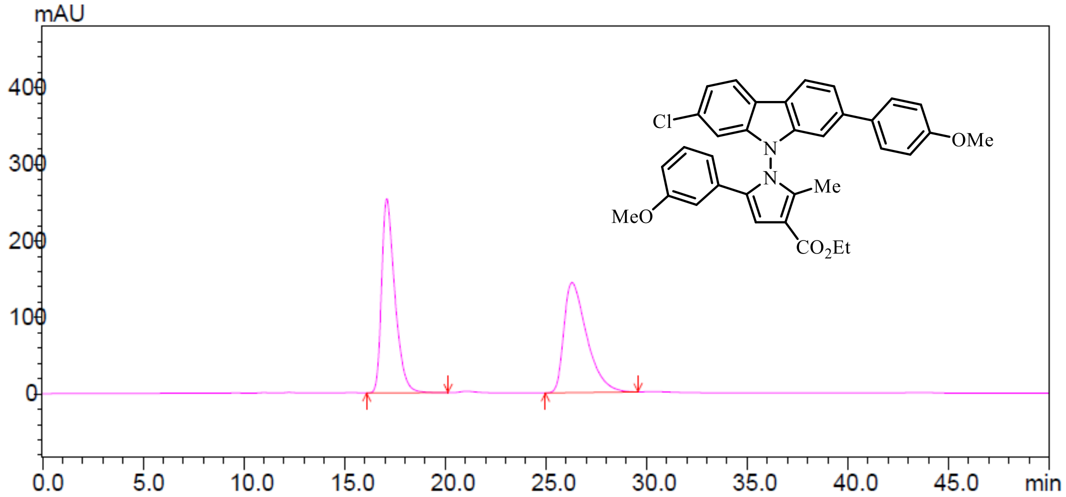

<Peak Table>

PDA Ch1 312nm

Peak#	Ret. Time	Area	Height	Peak Start	Peak End	Area%
1	17.104	11633389	253823	16.107	20.149	50.487
2	26.307	11409179	143844	24.981	29.579	49.513
Total		23042568	397668			100.000

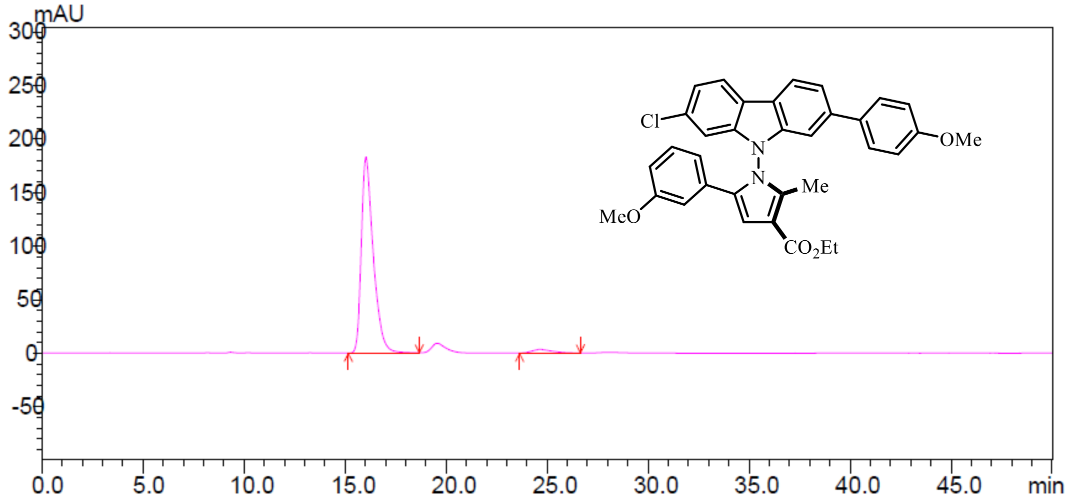

<Peak Table>

PDA Ch1 312nm

Peak#	Ret. Time	Area	Height	Peak Start	Peak End	Area%
1	16.033	7570102	183157	15.157	18.677	96.875
2	24.648	244170	3407	23.637	26.645	3.125
Total		7814272	186565			100.000

52

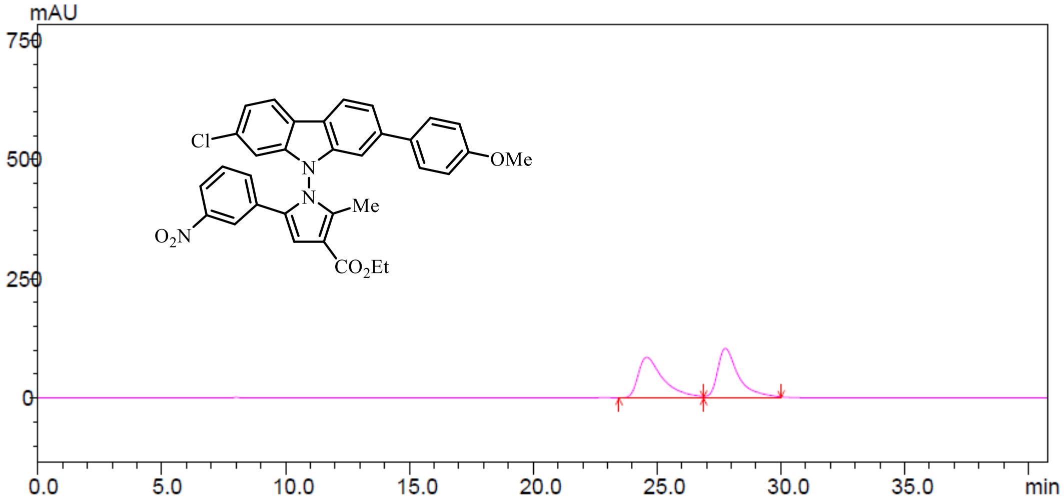

<Peak Table>

PDA Ch1 325nm

Peak#	Ret. Time	Area	Height	Peak Start	Peak End	Area%
1	24.585	6015551	85131	23.467	26.880	49.398
2	27.763	6162164	103790	26.880	30.016	50.602
Total		12177715	188921			100.000

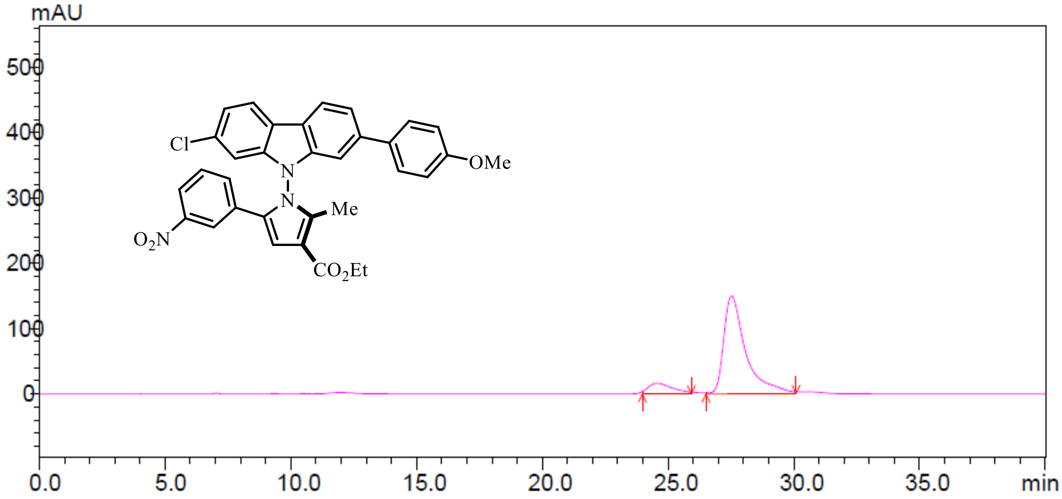

<Peak Table>

PDA Ch1 325nm

Peak#	Ret. Time	Area	Height	Peak Start	Peak End	Area%
1	24.565	1102293	16319	23.979	25.931	10.776
2	27.526	9127108	149822	26.507	30.091	89.224
Total		10229401	166141			100.000

53

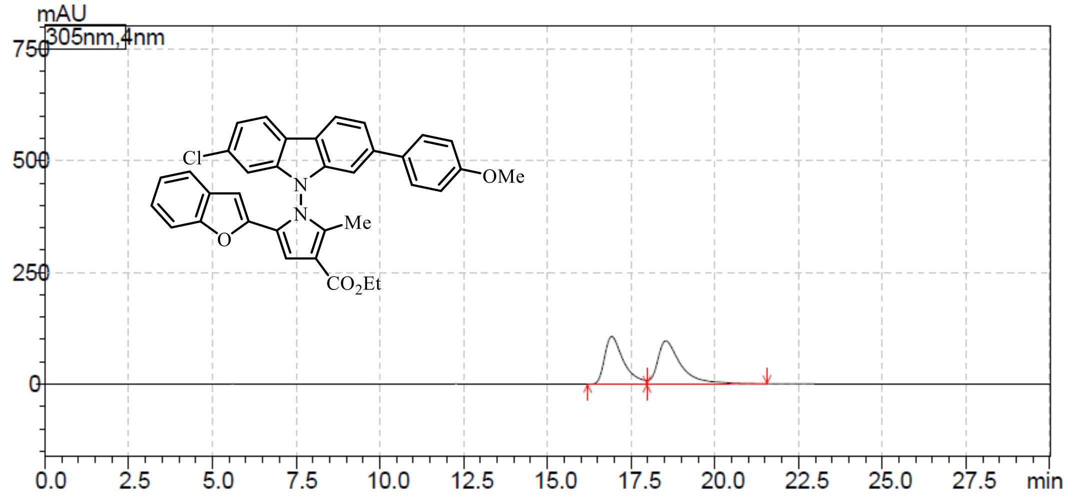

<Peak Table>

PDA Ch1 305nm

Peak#	Ret. Time	Area	Height	Peak Start	Peak End	Area%
1	16.922	4149282	106191	16.235	17.973	49.477
2	18.539	4237016	95511	17.973	19.584	50.523
Total		8386299	201703			100.000

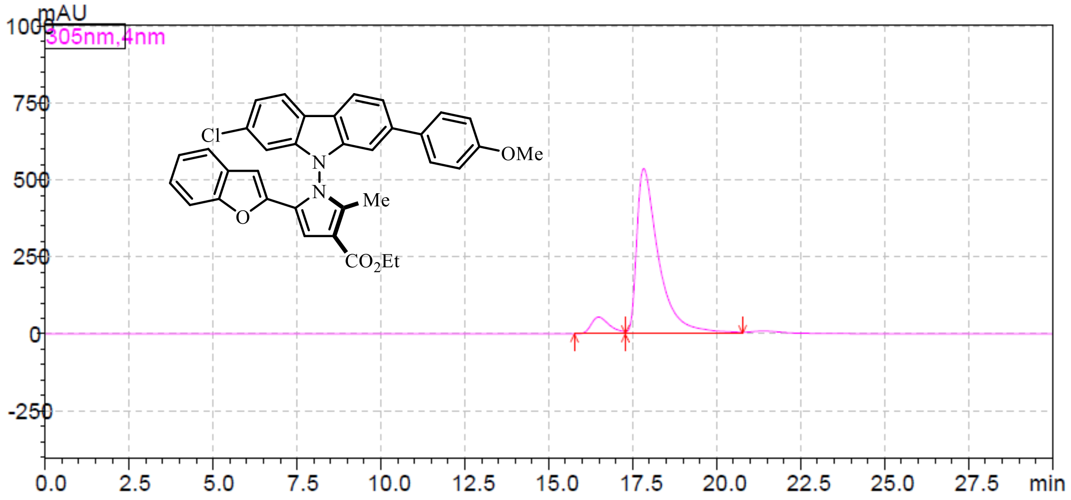

<Peak Table>

PDA Ch1 305nm

Peak#	Ret. Time	Area	Height	Peak Start	Peak End	Area%
1	16.483	2083652	54392	15.765	17.280	7.833
2	17.828	24518967	536707	17.280	20.768	92.167
Total		26602620	591099			100.000

54

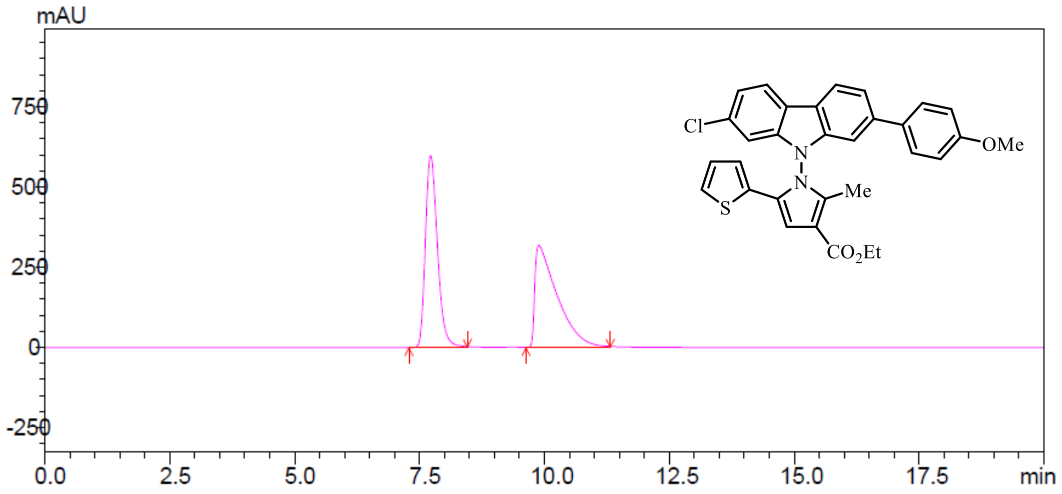

<Peak Table>

PDA Ch1 312nm

Peak#	Ret. Time	Area	Peak Start	Peak End	Area%
1	7.724	10022307	7.285	8.459	50.167
2	9.890	9955408	9.632	11.317	49.833
Total		19977715			100.000

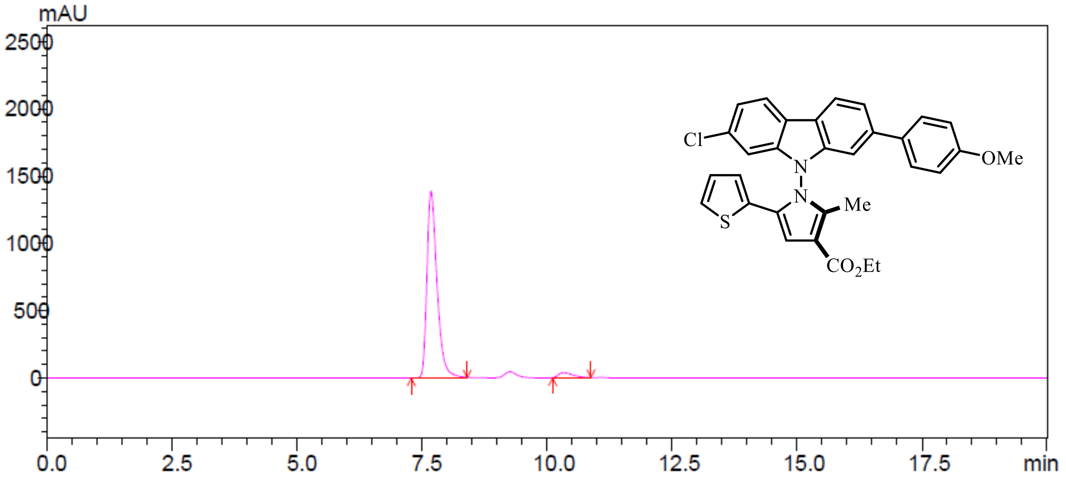

<Peak Table>

PDA Ch1 312nm

Peak#	Ret. Time	Area	Peak Start	Peak End	Area%
1	7.682	19650272	7.285	8.405	96.380
2	10.331	738142	10.112	10.880	3.620
Total		20388414			100.000

55

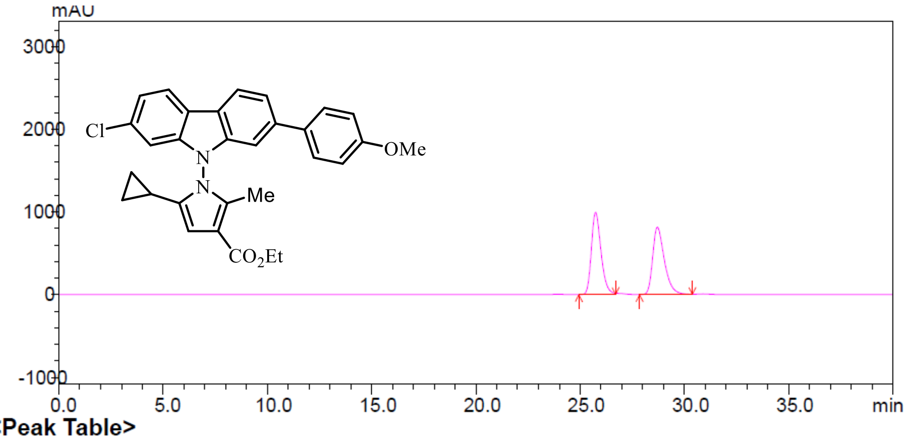

<Peak Table>

PDA Ch1 314nm

Peak#	Ret. Time	Area	Peak Start	Peak End	Area%
1	25.740	32117651	24.939	26.699	50.017
2	28.705	32095609	27.851	30.379	49.983
Total		64213260			100.000

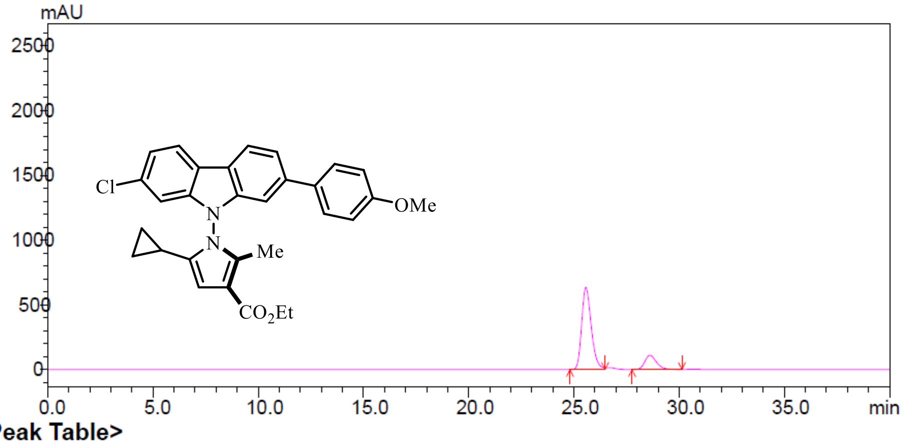

<Peak Table>

PDA Ch1 314nm

Peak#	Ret. Time	Area	Peak Start	Peak End	Area%
1	25.565	20240064	24.811	26.475	82.188
2	28.609	4386470	27.755	30.123	17.812
Total		24626534			100.000

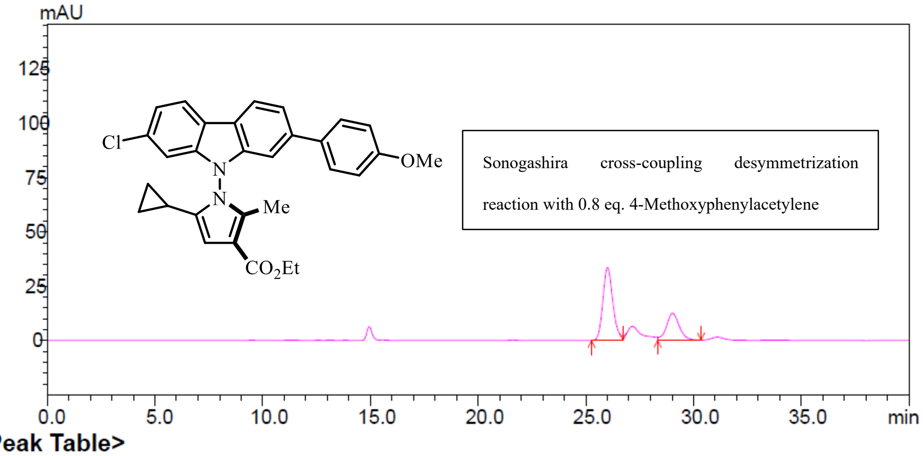

<Peak Table>

PDA Ch1 314nm

Peak#	Ret. Time	Area	Height	Peak Start	Peak End	Area%
1	26.006	1082617	33546	25.259	26.720	67.943
2	29.024	510802	12399	28.331	30.347	32.057
Total		1593418	45944			100.000

56

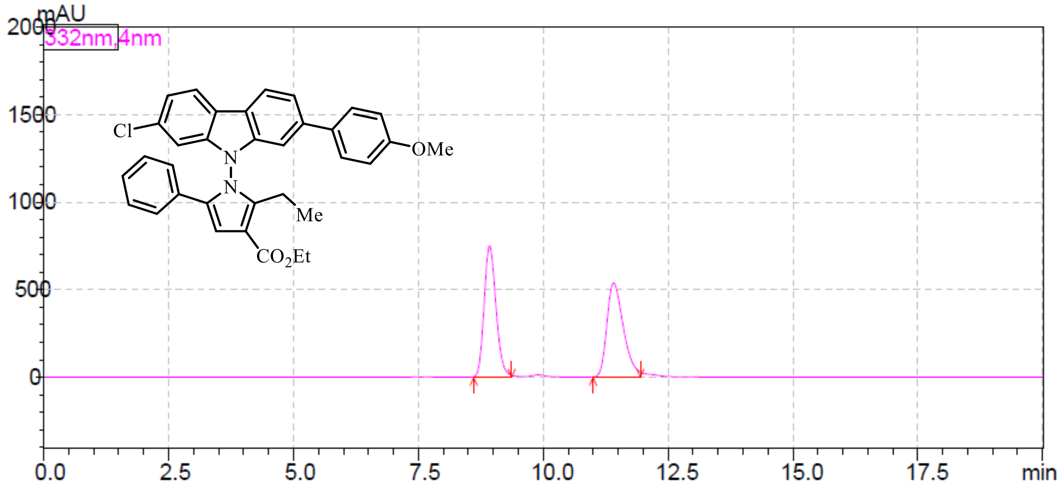

<Peak Table>

PDA Ch1 332nm

Peak#	Ret. Time	Area	Height	Peak Start	Peak End	Area%
1	8.925	12468478	749773	8.608	9.365	50.141
2	11.409	12398382	537827	10.997	11.947	49.859
Total		24866860	1287600			100.000

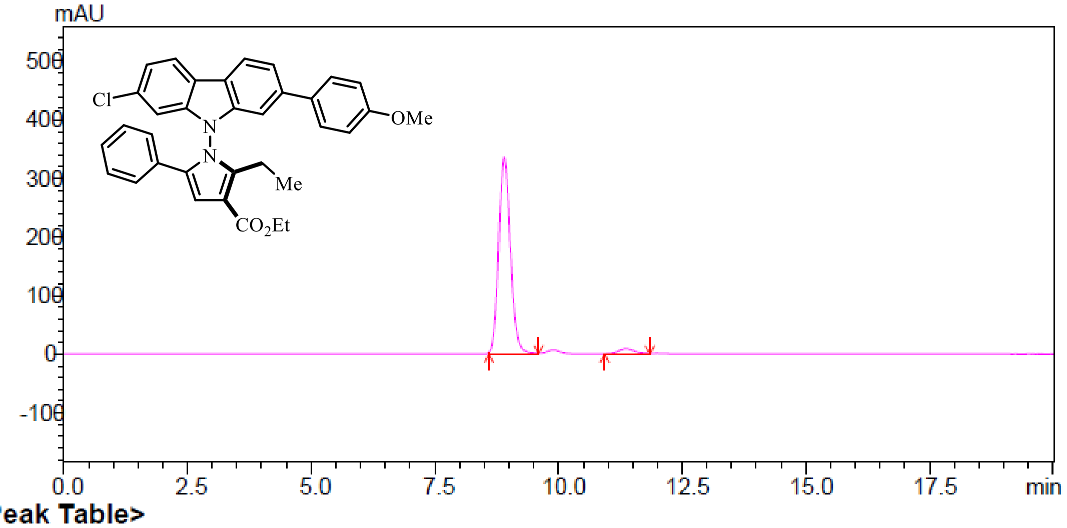

<Peak Table>

PDA Ch1 332nm

Peak#	Ret. Time	Area	Height	Peak Start	Peak End	Area%
1	8.903	5563146	336443	8.597	9.600	96.403
2	11.366	207581	8880	10.933	11.851	3.597
Total		5770727	345322			100.000

57

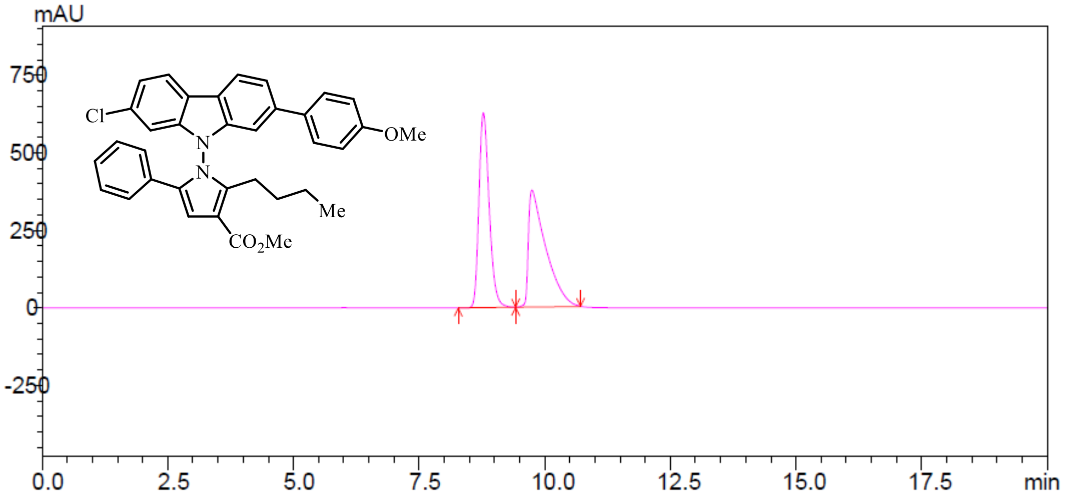

<Peak Table>

PDA Ch1 333nm

Peak#	Ret. Time	Area	Height	Peak Start	Peak End	Area%
1	8.779	8904596	628737	8.277	9.419	49.401
2	9.746	9120500	377300	9.419	10.709	50.599
Total		18025097	1006037			100.000

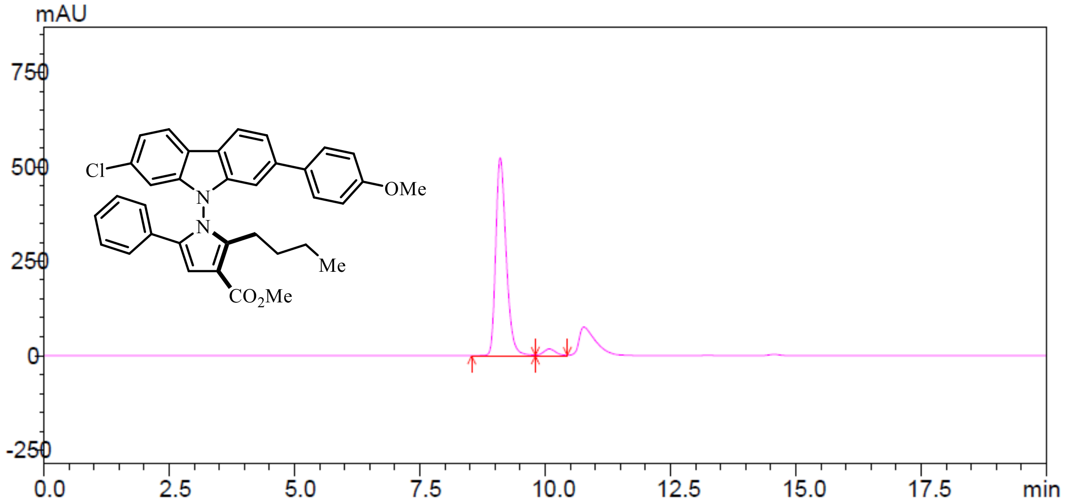

<Peak Table>

PDA Ch1 333nm

Peak#	Ret. Time	Area	Height	Peak Start	Peak End	Area%
1	9.103	7570692	524036	8.533	9.803	96.127
2	10.080	304997	17954	9.803	10.443	3.873
Total		7875690	541989			100.000

58

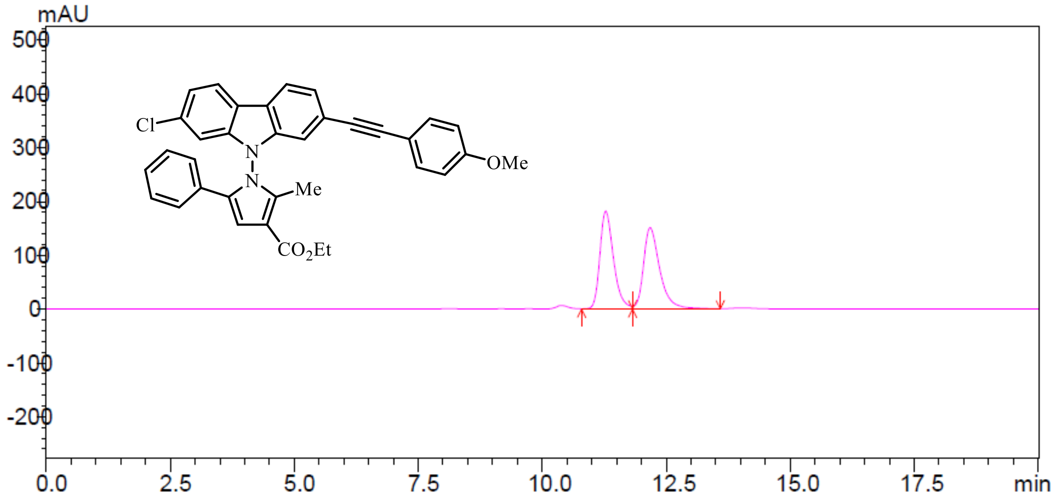

<Peak Table>

PDA Ch1 346nm

Peak#	Ret. Time	Area	Peak Start	Peak End	Area%
1	11.281	3489890	10.795	11.819	50.072
2	12.176	3479851	11.819	13.589	49.928
Total		6969741			100.000

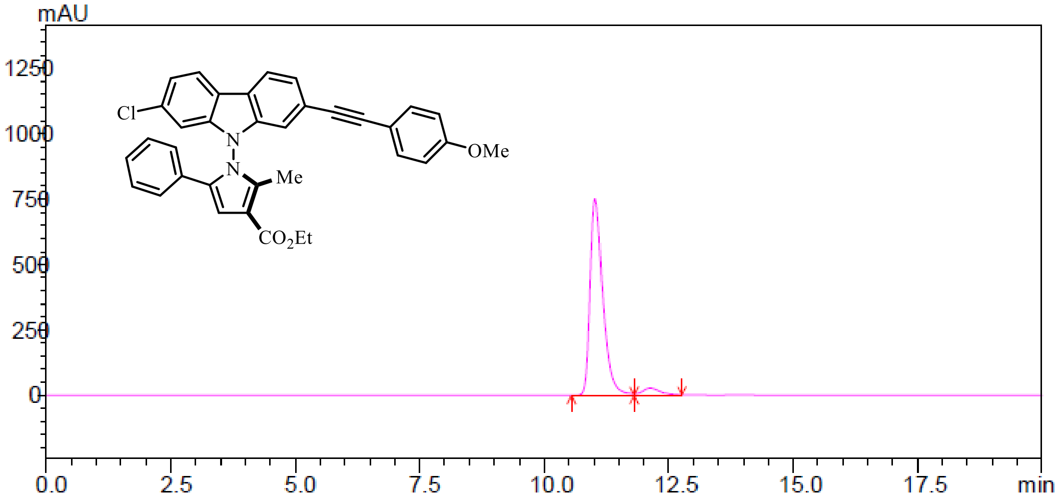

<Peak Table>

PDA Ch1 346nm

Peak#	Ret. Time	Area	Peak Start	Peak End	Area%
1	11.023	13312461	10.549	11.808	94.969
2	12.131	705253	11.808	12.768	5.031
Total		14017714			100.000

59

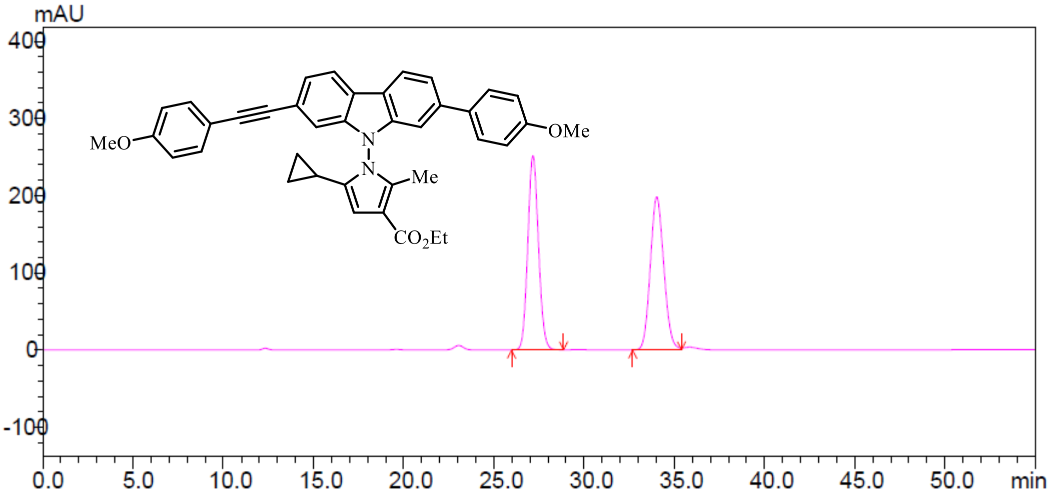

<Peak Table>

PDA Ch1 342nm

Peak#	Ret. Time	Area	Height	Peak Start	Peak End	Area%
1	27.164	10353536	251583	25.973	28.864	49.982
2	34.025	10360821	198488	32.693	35.403	50.018
Total		20714357	450071			100.000

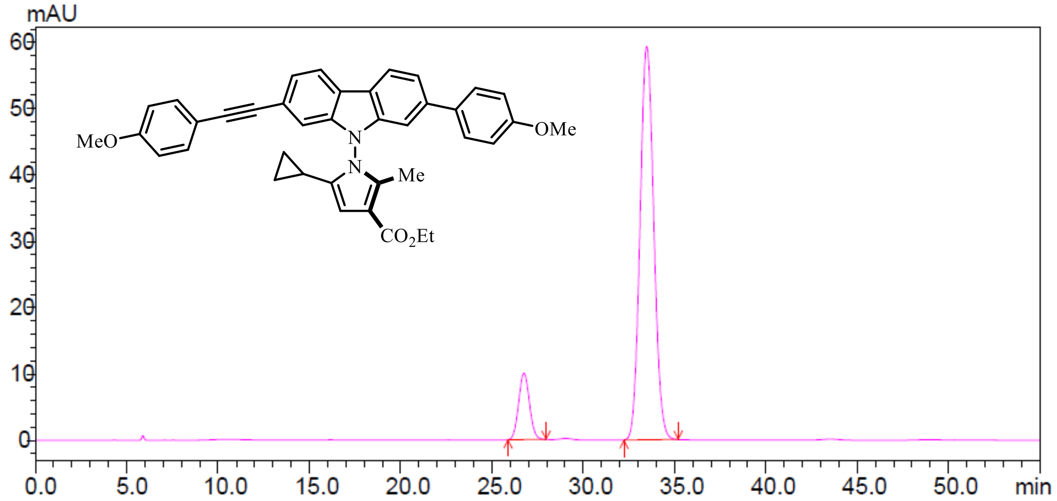

<Peak Table>

PDA Ch1 342nm

Peak#	Ret. Time	Area	Height	Peak Start	Peak End	Area%
1	26.751	414110	10011	25.877	27.957	11.742
2	33.476	3112659	59269	32.235	35.200	88.258
Total		3526769	69280			100.000

Desymmetrization of et-ester

2

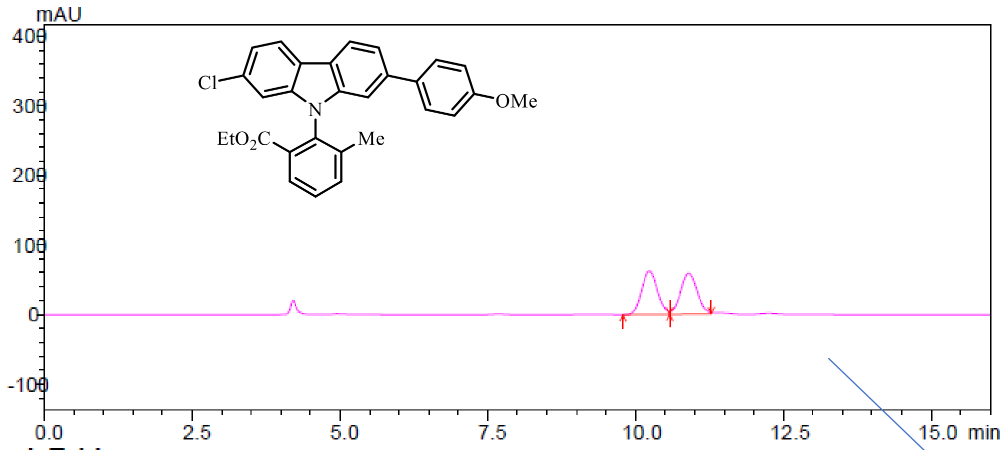

<Peak Table>

PDA Ch1 315nm

Peak#	Ret. Time	Area	Height	Peak Start	Peak End	Area%
1	10.232	1149971	62252	9.787	10.576	49.988
2	10.899	1150517	59274	10.576	11.280	50.012
Total		2300488	121526			100.000

Daicel Chiralpak IC, hexane/*iso*-propanol = 99:1, 0.8 mL/min,

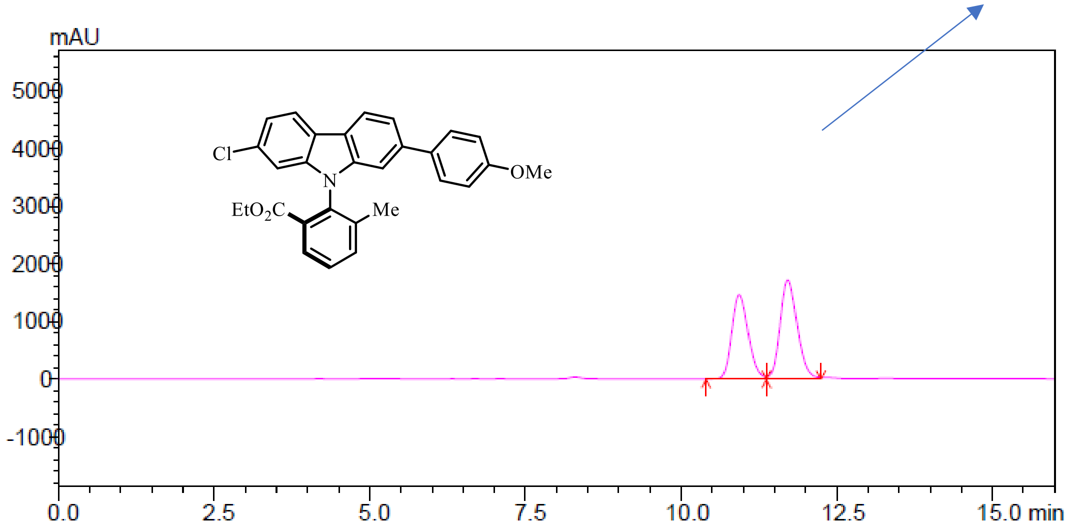

<Peak Table>

PDA Ch1 315nm

Peak#	Ret. Time	Area	Peak Start	Peak End	Area%
1	10.934	27124383	10.400	11.371	44.951
2	11.713	33218075	11.371	12.245	55.049
Total		60342457			100.000

49

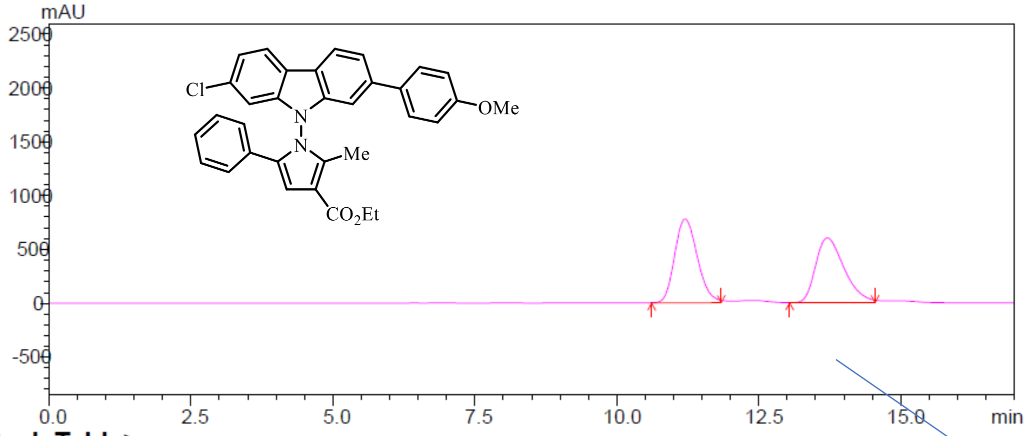

<Peak Table>

PDA Ch1 315nm

Peak#	Ret. Time	Area	Height	Peak Start	Peak End	Area%
1	11.203	21750949	778681	10.613	11.840	50.666
2	13.708	21178961	602891	13.045	14.549	49.334
Total		42929910	1381573			100.000

Daicel Chiralpak IC, hexane/*iso*-propanol = 99:1, 1.0 mL/min,

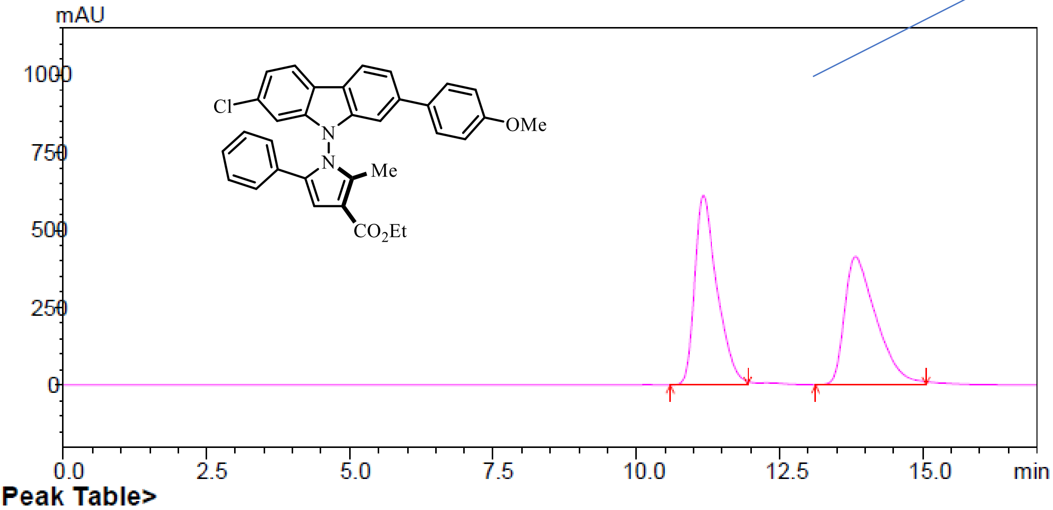

<Peak Table>

PDA Ch1 315nm

Peak#	Ret. Time	Area	Height	Peak Start	Peak End	Area%
1	11.173	16632329	612126	10.592	11.947	50.823
2	13.830	16093514	414701	13.131	15.061	49.177
Total		32725844	1026827			100.000

61

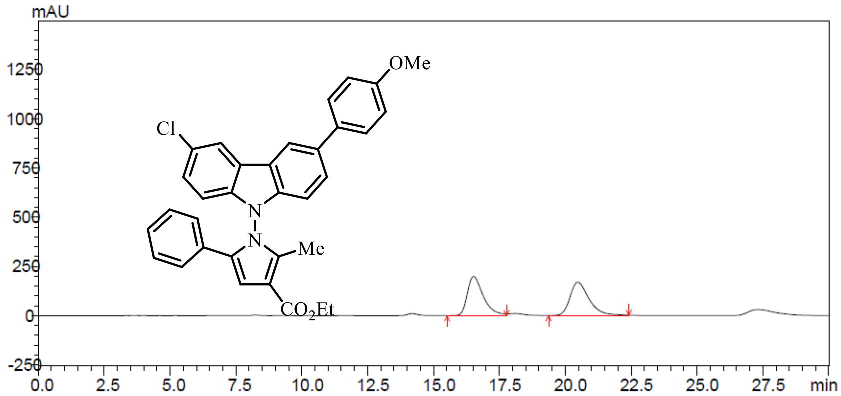

<Peak Table>

PDA Ch1 294nm

Peak#	Ret. Time	Area	Peak Start	Peak End	Area%
1	16.521	12717412	15.584	17.504	49.213
2	20.476	13124338	19.392	21.899	50.787
Total		25841750			100.000

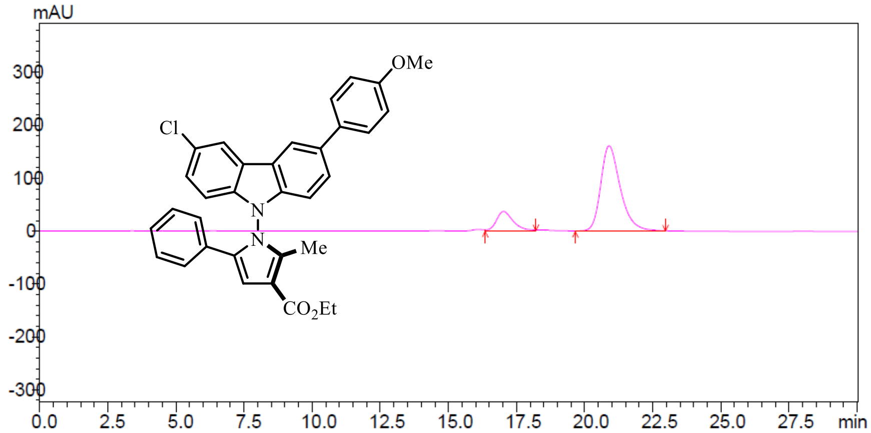

<Peak Table>

PDA Ch1 294nm

Peak#	Ret. Time	Area	Peak Start	Peak End	Area%
1	17.022	1610739	16.352	18.208	16.510
2	20.895	8145121	19.659	22.965	83.490
Total		9755859			100.000

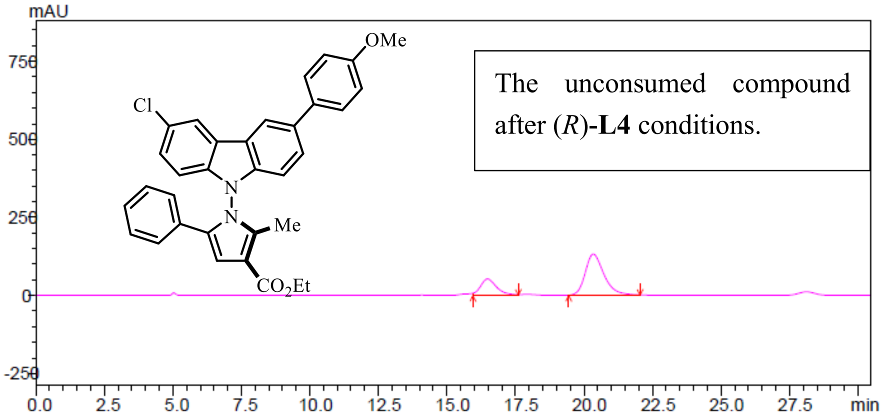

The unconsumed compound after (*R*)-**L4** conditions.

<Peak Table>

PDA Ch1 294nm

Peak#	Ret. Time	Area	Peak Start	Peak End	Area%
1	16.480	2178174	15.947	17.600	25.560
2	20.340	6343590	19.445	22.048	74.440
Total		8521764			100.000

62

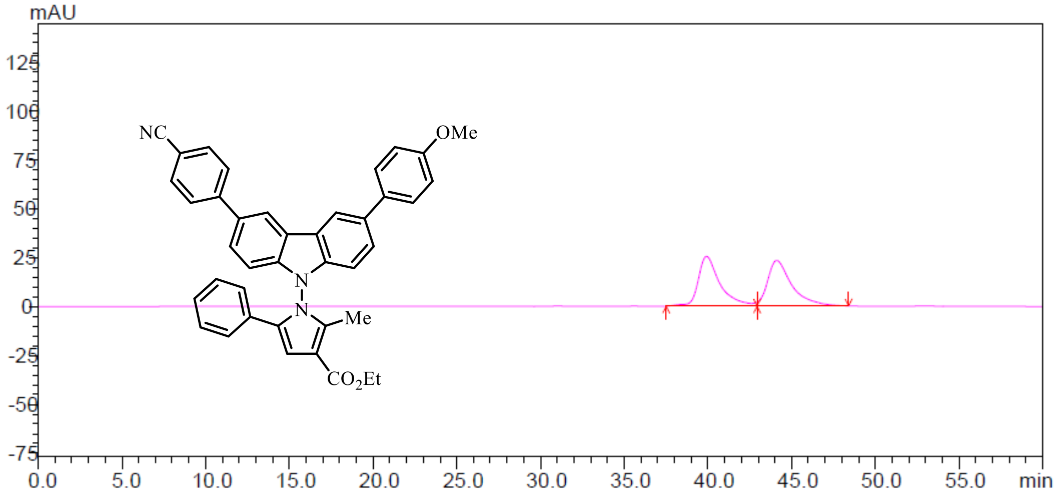

<Peak Table>

PDA Ch1 333nm

Peak#	Ret. Time	Area	Peak Start	Peak End	Area%
1	39.918	2326190	37.515	42.933	49.699
2	44.107	2354413	42.933	48.405	50.301
Total		4680603			100.000

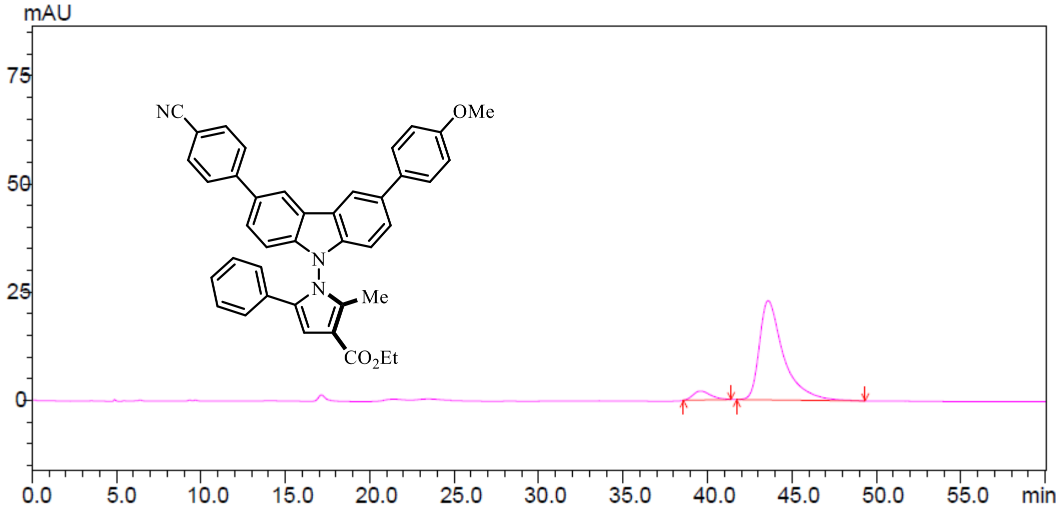

<Peak Table>

PDA Ch1 333nm

Peak#	Ret. Time	Area	Height	Peak Start	Peak End	Area%
1	39.597	152591	2104	38.581	41.397	6.285
2	43.593	2275123	22941	41.739	49.333	93.715
Total		2427714	25045			100.000

64

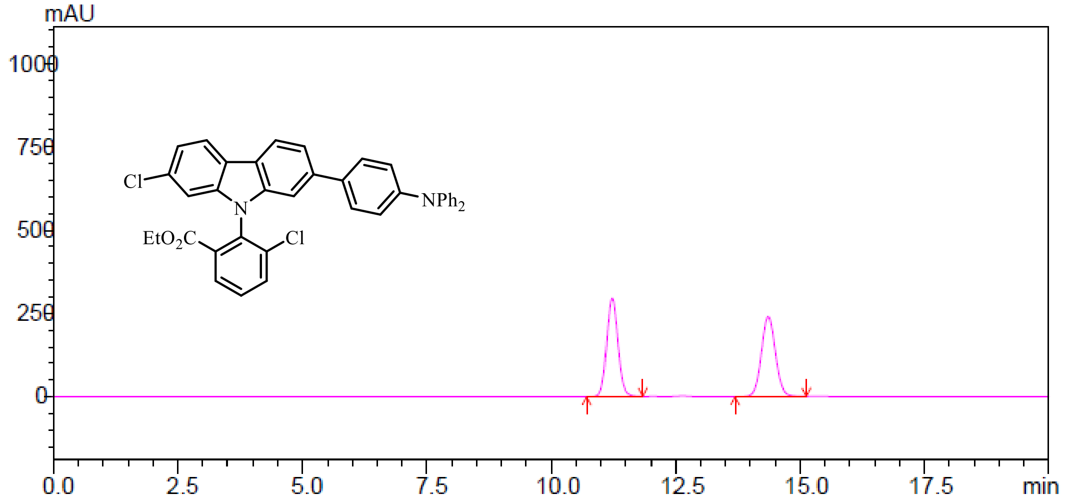

<Peak Table>

PDA Ch1 347nm

Peak#	Ret. Time	Area	Height	Peak Start	Peak End	Area%
1	11.225	4711066	295597	10.709	11.840	49.994
2	14.362	4712213	239403	13.696	15.125	50.006
Total		9423278	535000			100.000

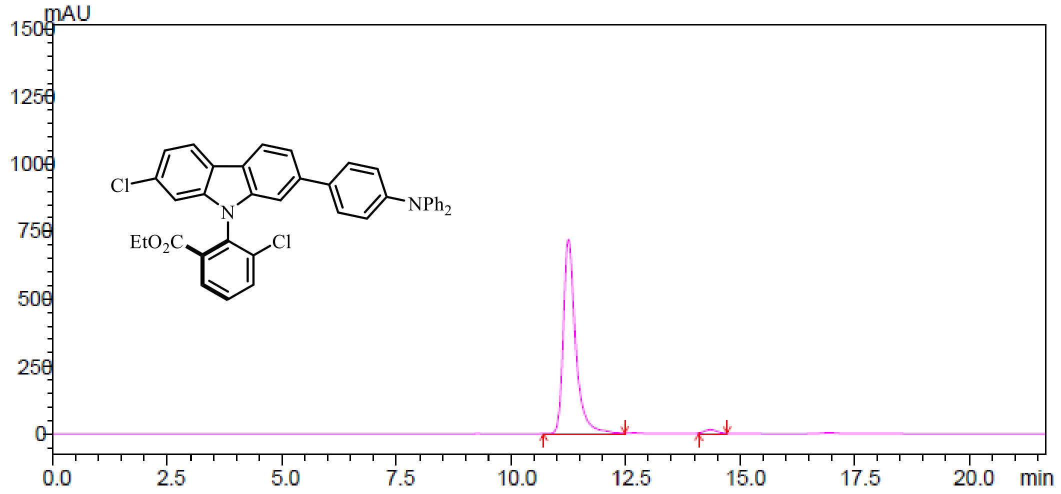

<Peak Table>

PDA Ch1 347nm

Peak#	Ret. Time	Area	Peak Start	Peak End	Area%
1	11.259	13433647	10.709	12.480	97.779
2	14.350	305118	14.101	14.720	2.221
Total		13738765			100.000

65

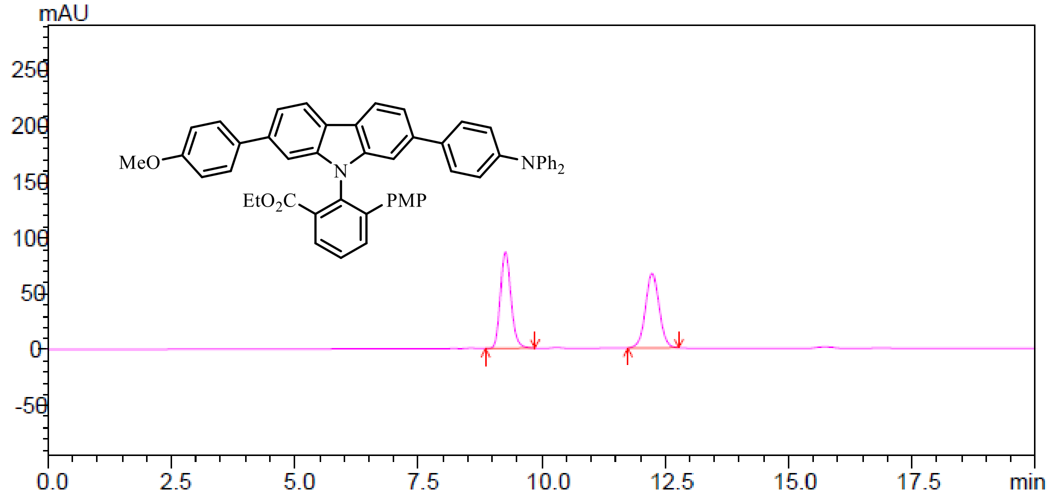

<Peak Table>

PDA Ch1 351nm

Peak#	Ret. Time	Area	Height	Peak Start	Peak End
1	9.264	1277878	86416	8.864	9.867
2	12.240	1283713	66656	11.744	12.779
Total		2561591	153072		

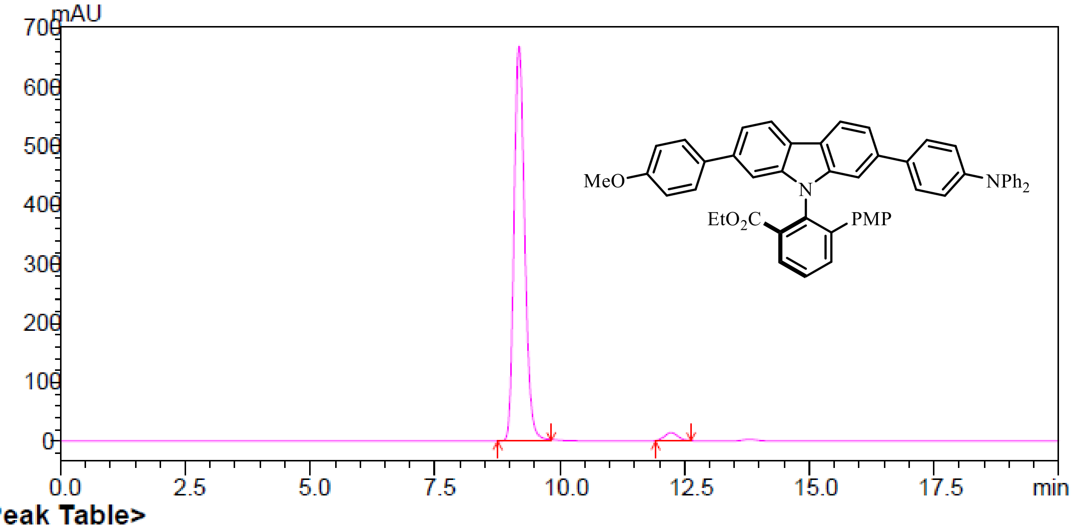

<Peak Table>

PDA Ch1 351nm

Peak#	Ret. Time	Area	Peak Start	Peak End	Area%
1	9.186	9842878	8.757	9.835	97.230
2	12.231	280437	11.925	12.629	2.770
Total		10123315			100.000

66

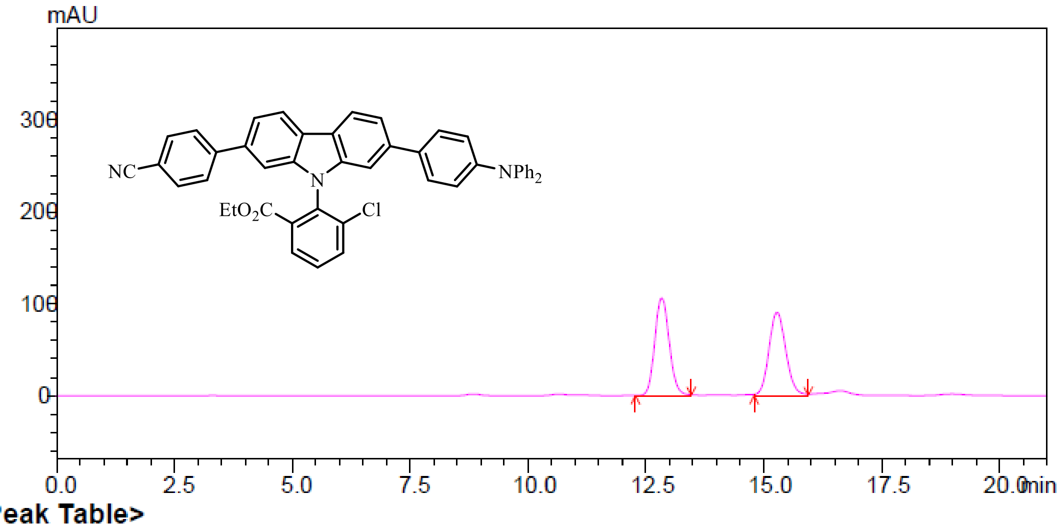

<Peak Table>

PDA Ch1 375nm

Peak#	Ret. Time	Area	Height	Peak Start	Peak End	Area%
1	12.834	2246511	106140	12.277	13.461	50.043
2	15.280	2242663	90226	14.795	15.947	49.957
Total		4489174	196365			100.000

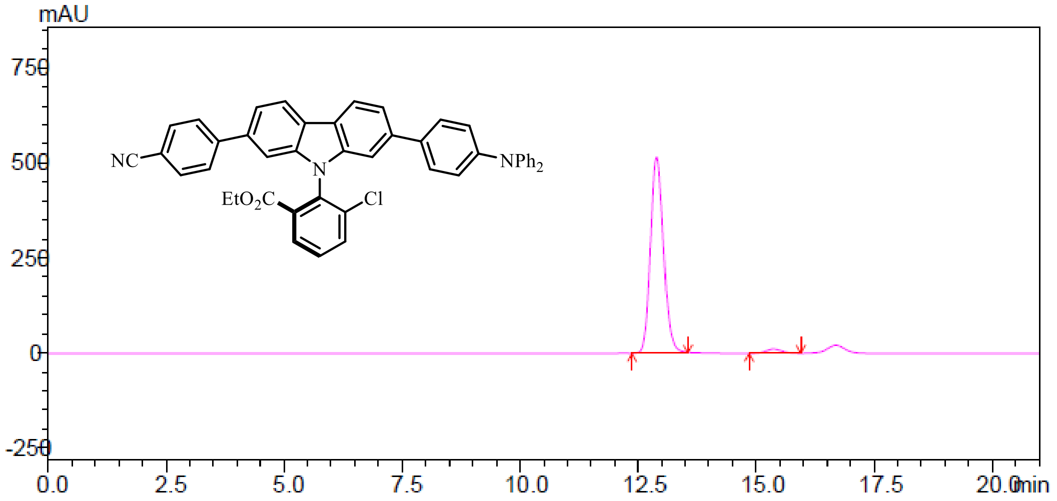

<Peak Table>

PDA Ch1 375nm

Peak#	Ret. Time	Area	Peak Start	Peak End	Area%
1	12.892	10049090	12.373	13.557	97.554
2	15.368	251968	14.848	15.947	2.446
Total		10301058			100.000

67

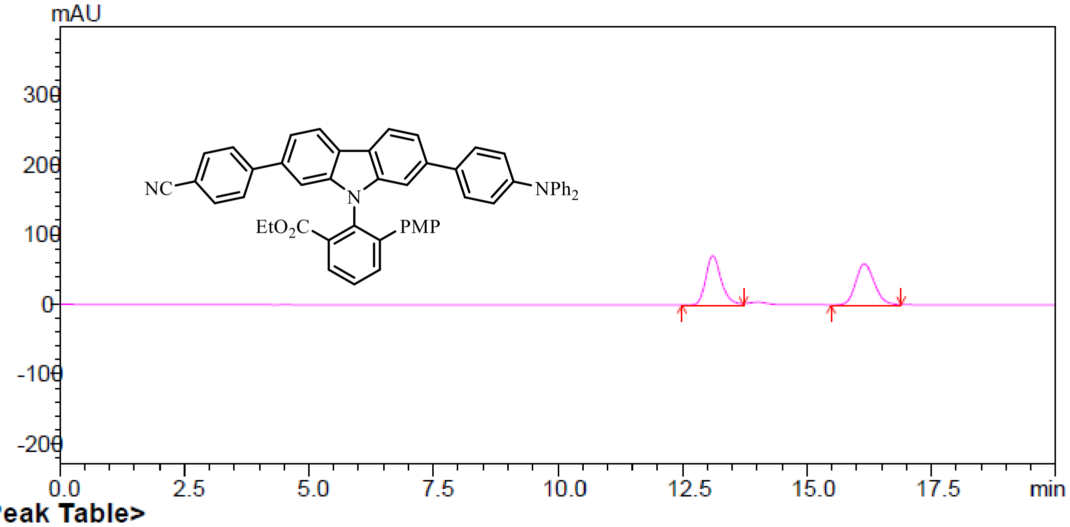

<Peak Table>

PDA Ch1 363nm

Peak#	Ret. Time	Area	Peak Start	Peak End	Area%
1	13.110	1547617	12.491	13.728	49.892
2	16.157	1554342	15.488	16.896	50.108
Total		3101959			100.000

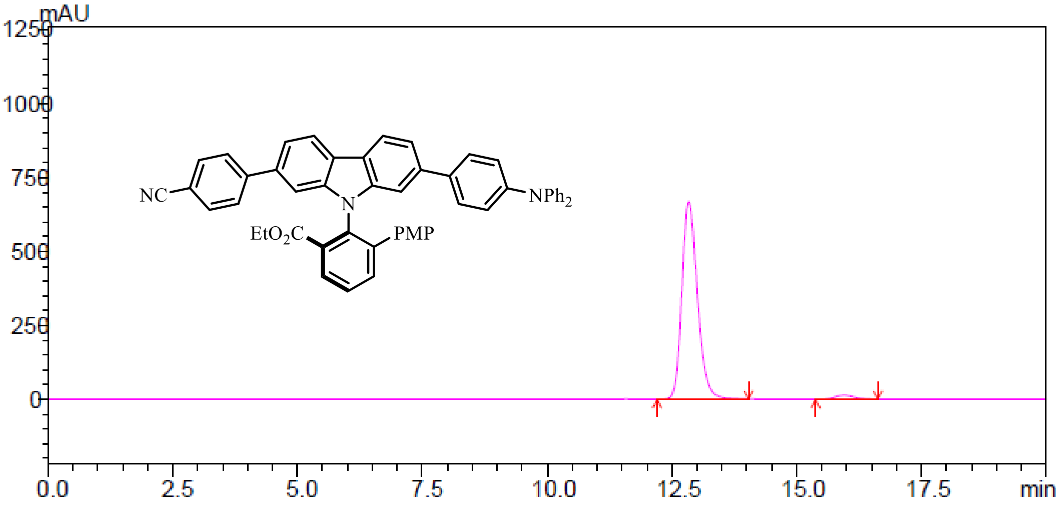

<Peak Table>

PDA Ch1 363nm

Peak#	Ret. Time	Area	Height	Peak Start	Peak End	Area%
1	12.837	14562254	668667	12.203	14.037	97.375
2	15.949	392509	14687	15.381	16.629	2.625
Total		14954763	683353			100.000

70

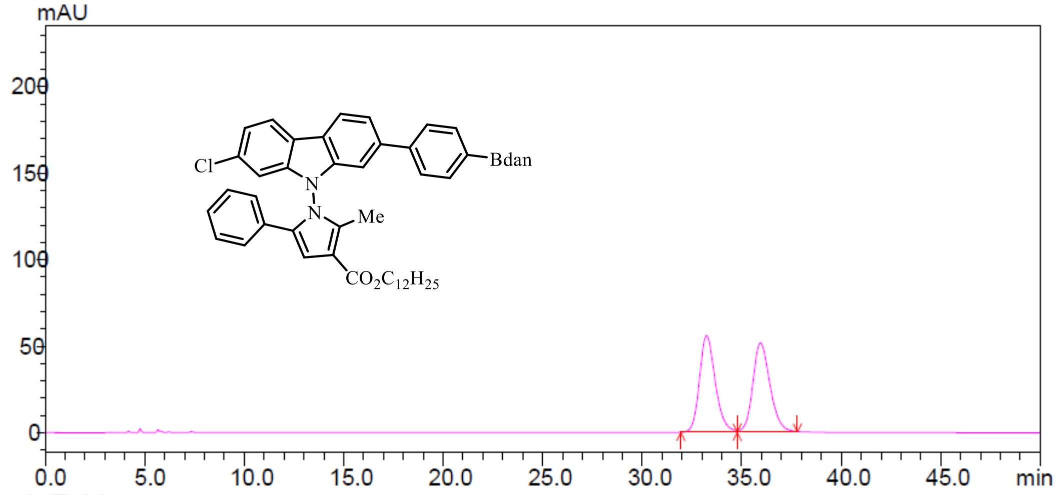

<Peak Table>

PDA Ch1 319nm

Peak#	Ret. Time	Area	Peak Start	Peak End	Area%
1	33.234	3104043	31.936	34.773	49.738
2	35.942	3136772	34.773	37.803	50.262
Total		6240815			100.000

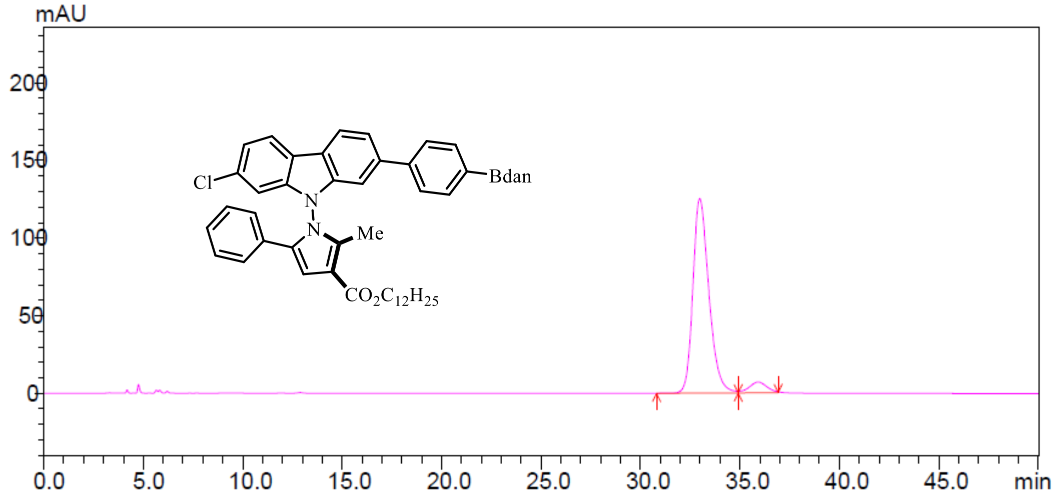

<Peak Table>

PDA Ch1 319nm

Peak#	Ret. Time	Area	Peak Start	Peak End	Area%
1	32.983	7011894	30.805	34.912	94.245
2	35.915	428210	34.912	36.917	5.755
Total		7440104			100.000

71

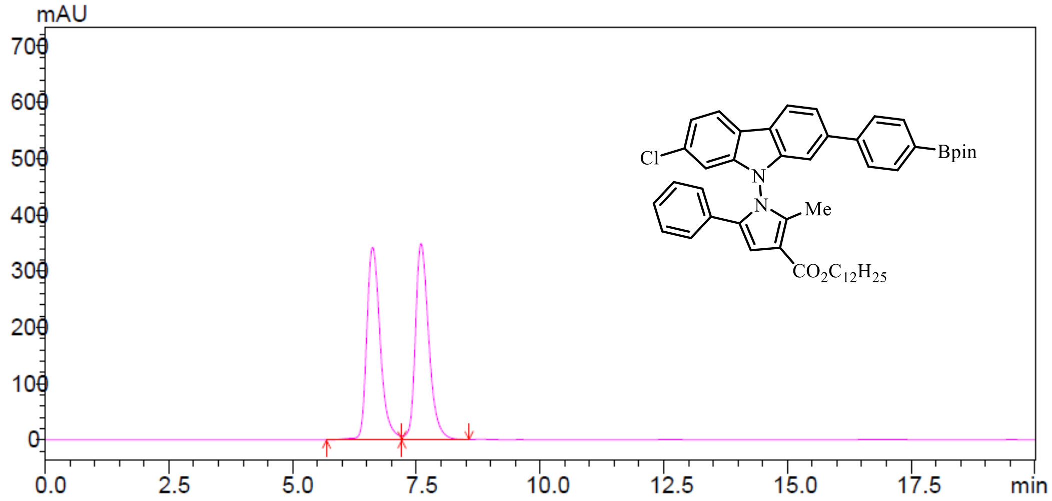

<Peak Table>

PDA Ch1 327nm

Peak#	Ret. Time	Area	Peak Start	Peak End	Area%
1	6.618	6497547	5.685	7.211	49.652
2	7.597	6588693	7.211	8.565	50.348
Total		13086240			100.000

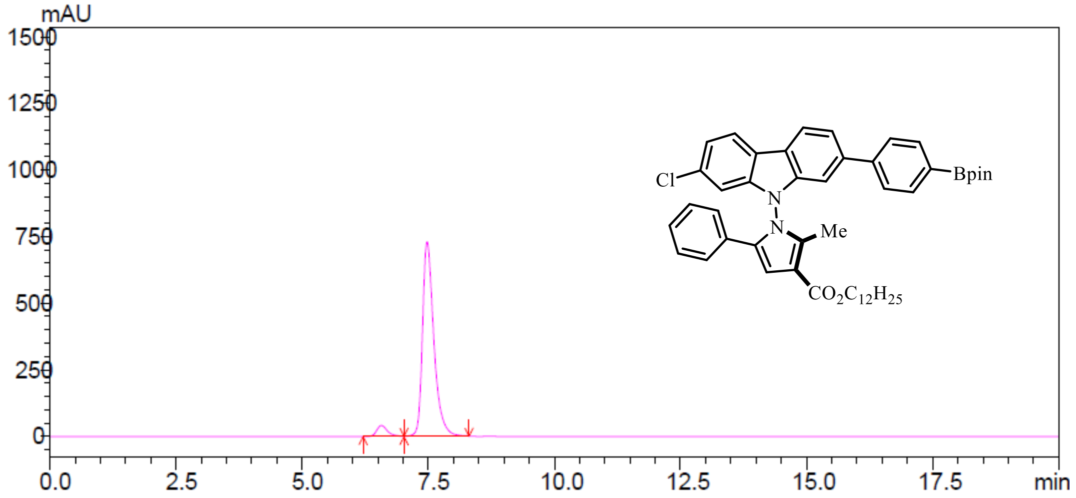

<Peak Table>

PDA Ch1 327nm

Peak#	Ret. Time	Area	Peak Start	Peak End	Area%
1	6.572	628550	6.208	7.019	5.172
2	7.478	11525264	7.019	8.309	94.828
Total		12153814			100.000

SI-**74**

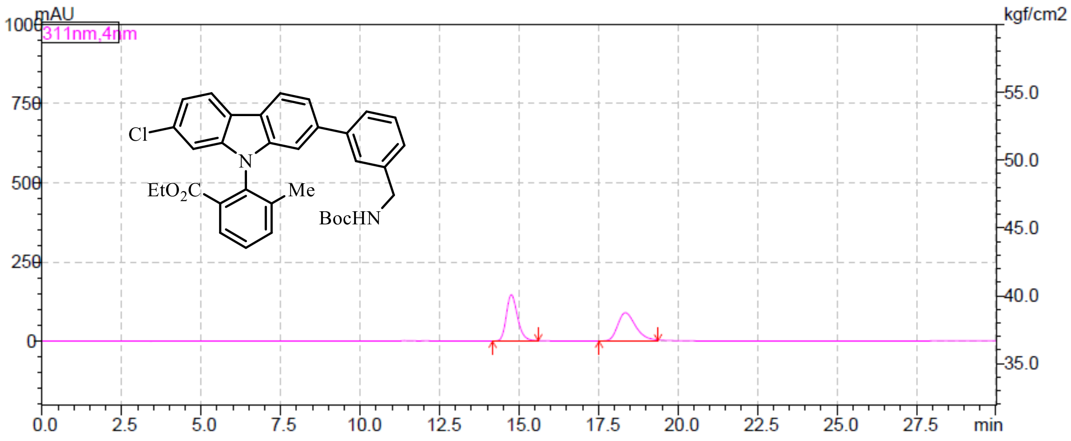

<Peak Table>

PDA Ch1 311nm

Peak#	Ret. Time	Area	Height	Peak Start	Peak End	Area%
1	14.758	3606410	146217	14.165	15.605	50.393
2	18.346	3550215	89360	17.515	19.371	49.607
Total		7156625	235577			100.000

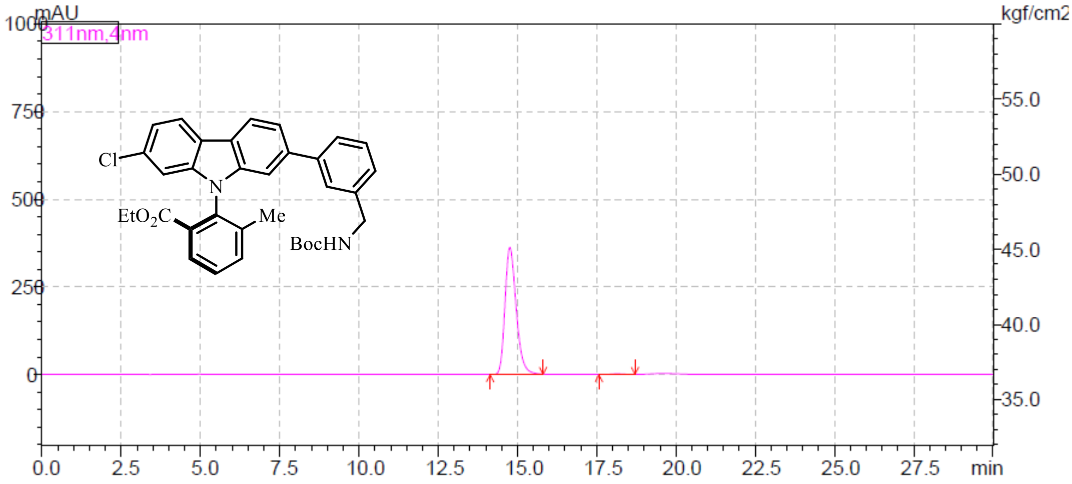

<Peak Table>

PDA Ch1 311nm

Peak#	Ret. Time	Area	Height	Peak Start	Peak End	Area%
1	14.760	8974647	362602	14.133	15.808	99.162
2	18.145	75873	2277	17.568	18.709	0.838
Total		9050520	364878			100.000

75

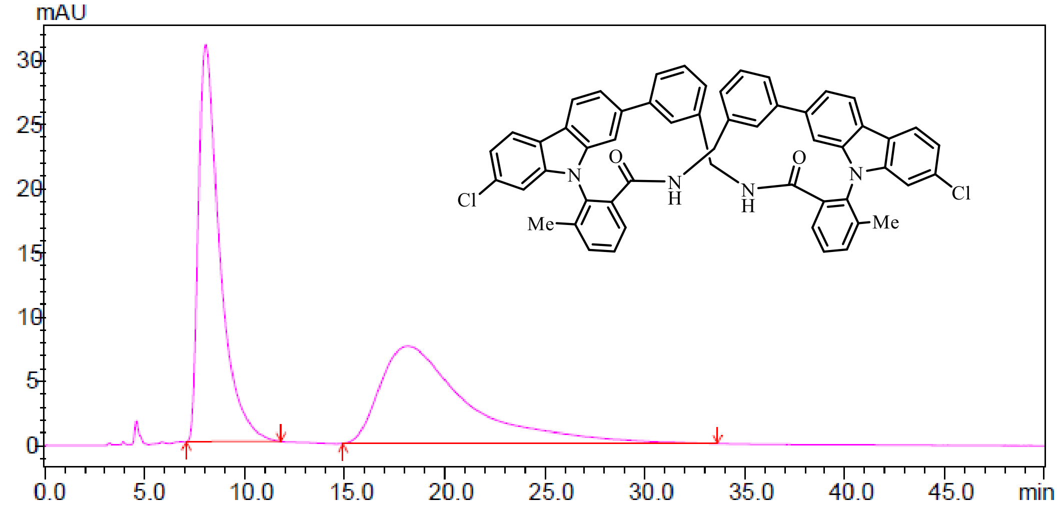

<Peak Table>

PDA Ch1 330nm

Peak#	Ret. Time	Area	Height	Peak Start	Peak End	Area%
1	8.045	2294781	30915	7.072	11.808	50.083
2	18.158	2287199	7592	14.912	33.643	49.917
Total		4581980	38507			100.000

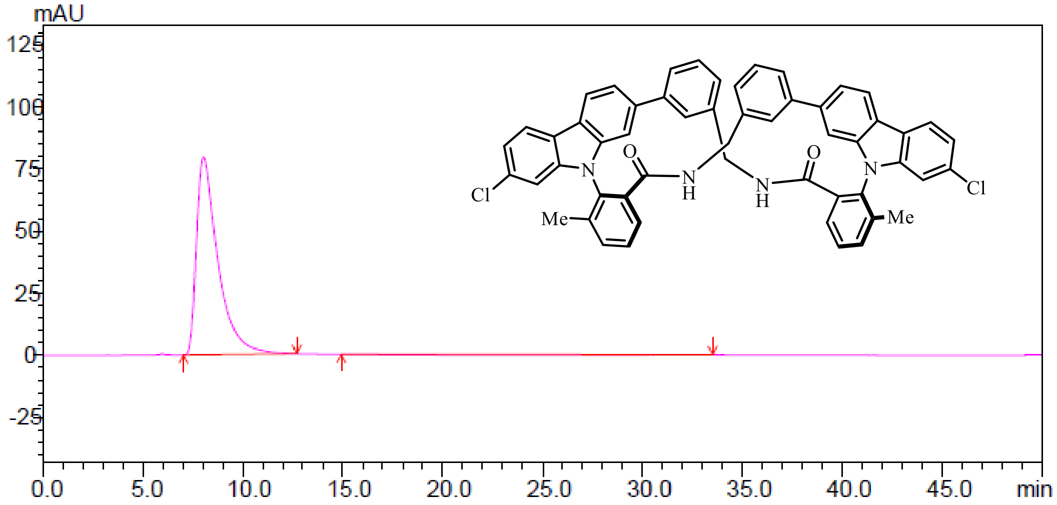

<Peak Table>

PDA Ch1 330nm

Peak#	Ret. Time	Area	Peak Start	Peak End	Area%
1	8.045	5942730	7.040	12.704	101.839
2	14.951	-107318	14.923	33.504	-1.839
Total		5835412			100.000

76

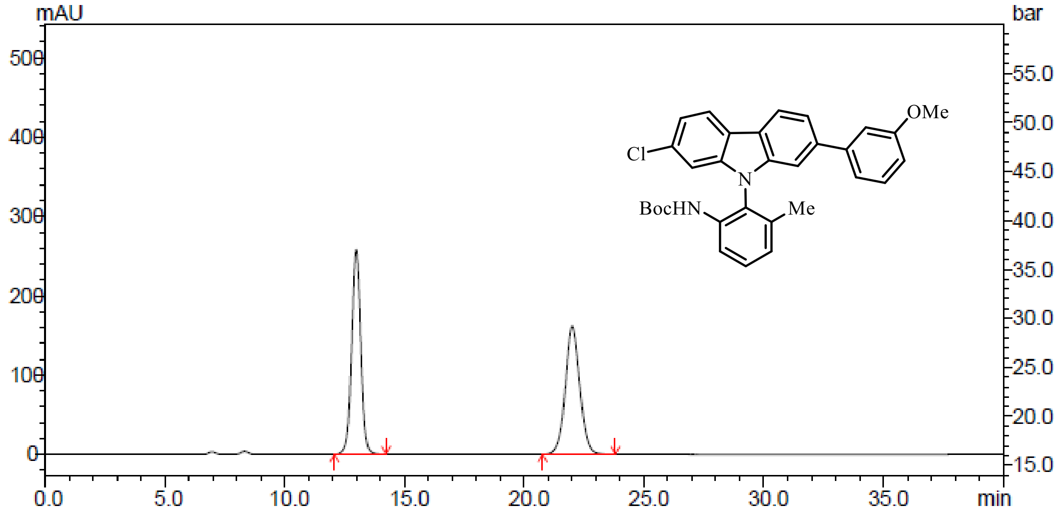

<Peak Table>

PDA Ch1 311nm

Peak#	Ret. Time	Area	Peak Start	Peak End	Area%
1	12.987	6944646	12.075	14.235	50.368
2	22.002	6843294	20.773	23.808	49.632
Total		13787939			100.000

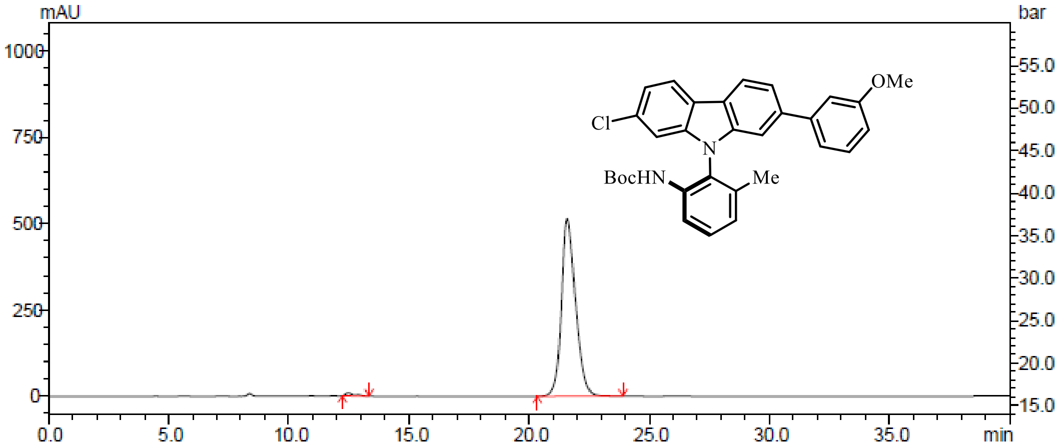

<Peak Table>

PDA Ch1 311nm

Peak#	Ret. Time	Area	Peak Start	Peak End	Area%
1	12.478	221909	12.229	13.333	1.039
2	21.586	21129532	20.347	23.909	98.961
Total		21351441			100.000

77

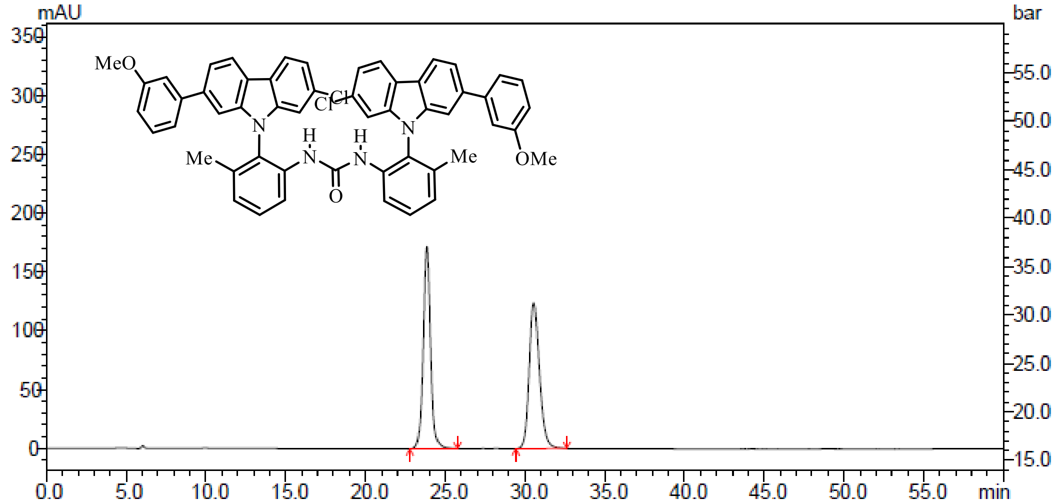

<Peak Table>

PDA Ch1 311nm

Peak#	Ret. Time	Area	Peak Start	Peak End	Area%
1	23.850	5584046	22.789	25.760	50.106
2	30.550	5560379	29.419	32.624	49.894
Total		11144425			100.000

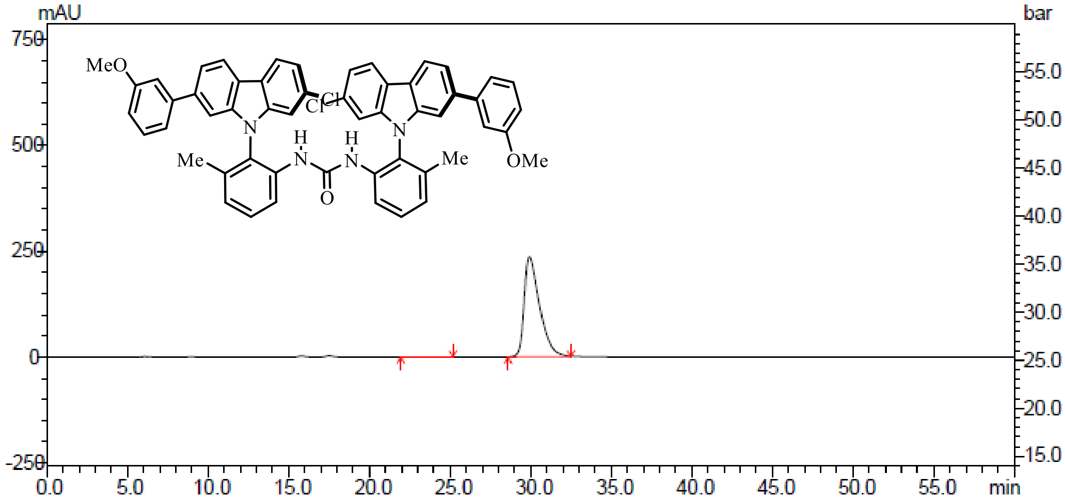

<Peak Table>

PDA Ch1 311nm

Peak#	Ret. Time	Area	Peak Start	Peak End	Area%
1	22.001	-915	21.952	25.173	-0.006
2	29.920	15806681	28.603	32.491	100.006
Total		15805766			100.000

78

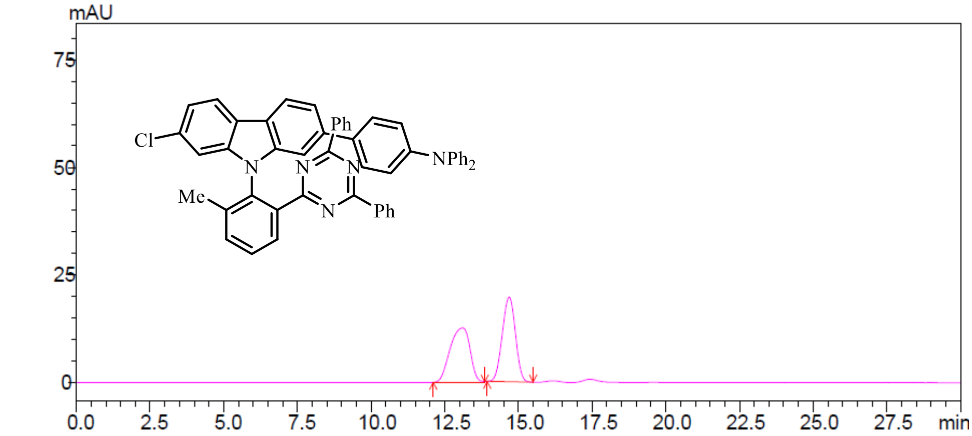

<Peak Table>

PDA Ch1 346nm

Peak#	Ret. Time	Area	Peak Start	Peak End	Area%
1	13.103	594284	12.107	13.856	49.286
2	14.686	611490	13.941	15.499	50.714
Total		1205774			100.000

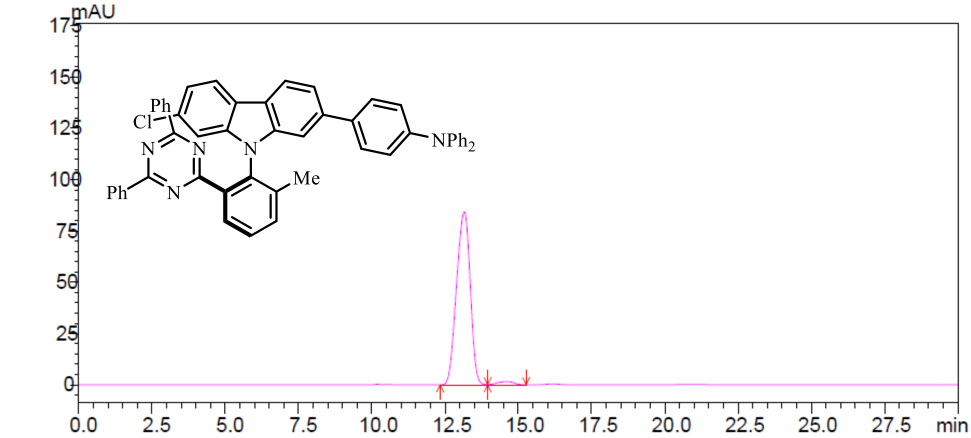

<Peak Table>

PDA Ch1 346nm

Peak#	Ret. Time	Area	Peak Start	Peak End	Area%
1	13.158	2740272	12.331	13.952	97.881
2	14.627	59316	13.952	15.264	2.119
Total		2799588			100.000

ent-**78**

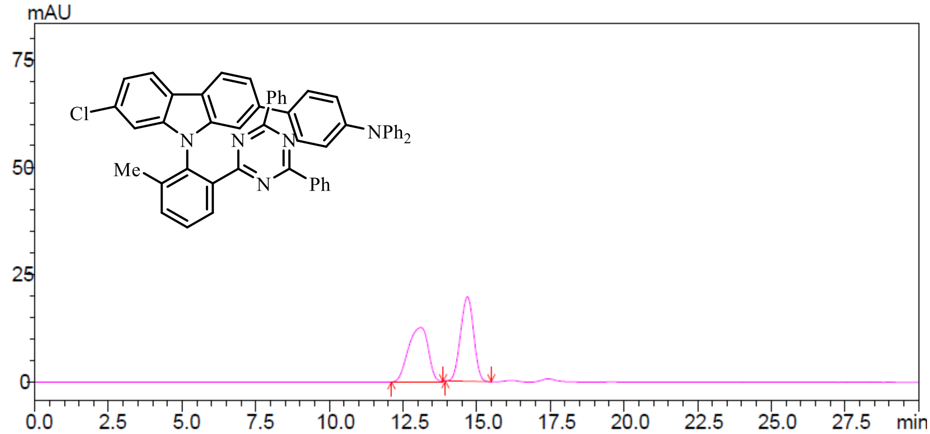

<Peak Table>

PDA Ch1 346nm

Peak#	Ret. Time	Area	Peak Start	Peak End	Area%
1	13.103	594284	12.107	13.856	49.286
2	14.686	611490	13.941	15.499	50.714
Total		1205774			100.000

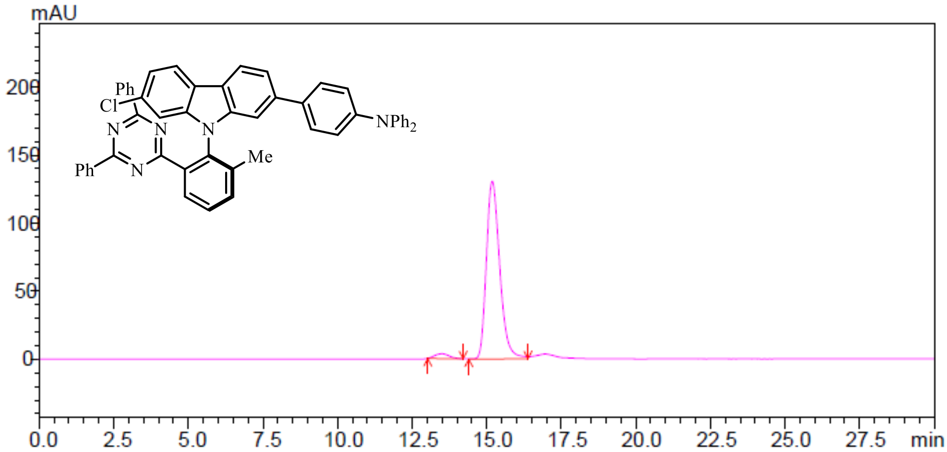

<Peak Table>

PDA Ch1 346nm

Peak#	Ret. Time	Area	Peak Start	Peak End	Area%
1	13.499	119173	13.024	14.208	2.838
2	15.187	4080490	14.400	16.384	97.162
Total		4199663			100.000

79

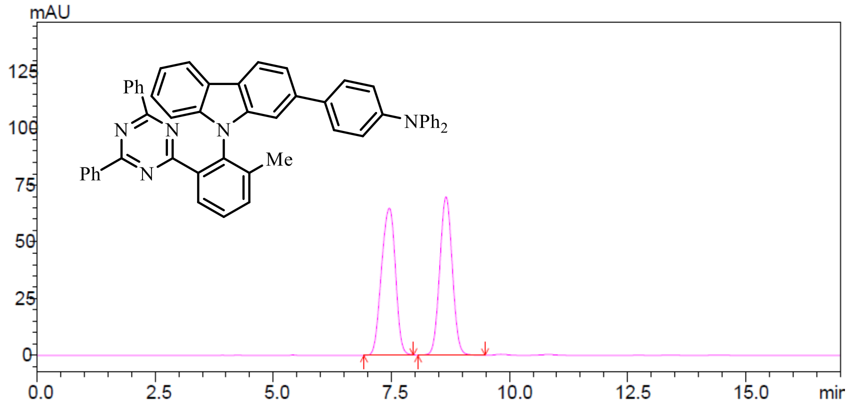

<Peak Table>

PDA Ch1 342nm

Peak#	Ret. Time	Area	Peak Start	Peak End	Area%
1	7.458	1342609	6.923	7.968	50.813
2	8.658	1299651	8.064	9.483	49.187
Total		2642261			100.000

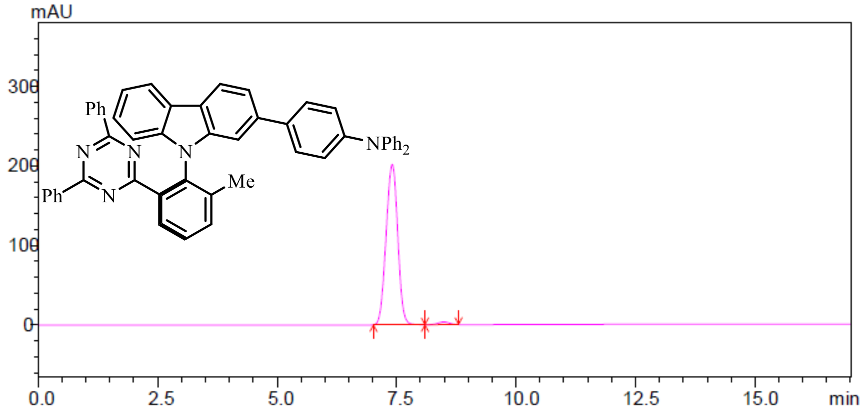

<Peak Table>

PDA Ch1 342nm

Peak#	Ret. Time	Area	Height	Peak Start	Peak End	Area%
1	7.411	3412161	201449	7.019	8.096	98.399
2	8.487	55520	3444	8.096	8.800	1.601
Total		3467680	204893			100.000

81

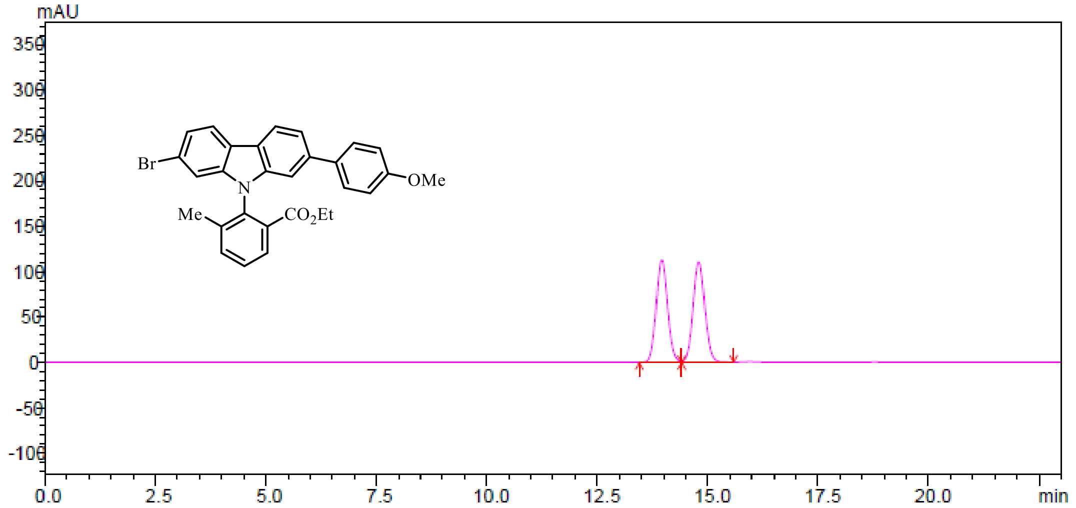

<Peak Table>

PDA Ch1 316nm

Peak#	Ret. Time	Area	Peak Start	Peak End	Area%
1	13.960	2039627	13.451	14.411	49.636
2	14.799	2069514	14.411	15.584	50.364
Total		4109141			100.000

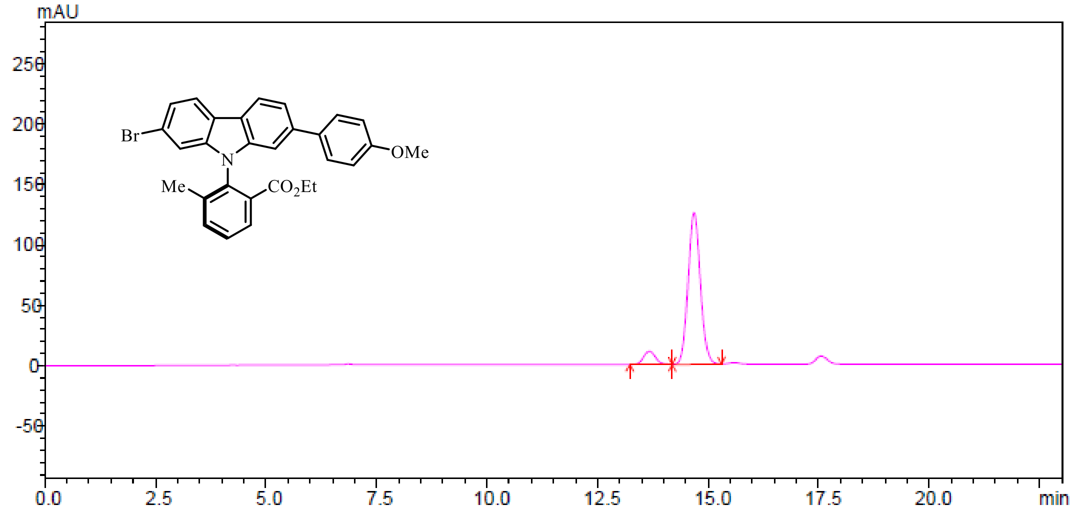

<Peak Table>

PDA Ch1 316nm

Peak#	Ret. Time	Area	Height	Peak Start	Peak End	Area%
1	13.671	199261	10736	13.227	14.187	7.532
2	14.682	2446421	125553	14.187	15.307	92.468
Total		2645682	136288			100.000

83

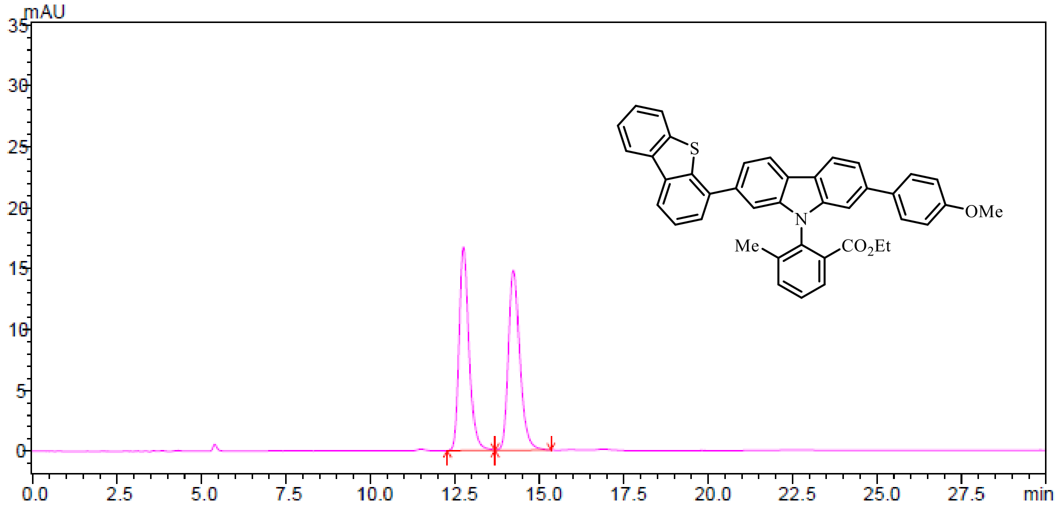

<Peak Table>

PDA Ch1 325nm

Peak#	Ret. Time	Area	Height	Peak Start	Peak End	Area%
1	12.766	347807	16737	12.288	13.696	49.882
2	14.245	349453	14786	13.696	15.349	50.118
Total		697260	31523			100.000

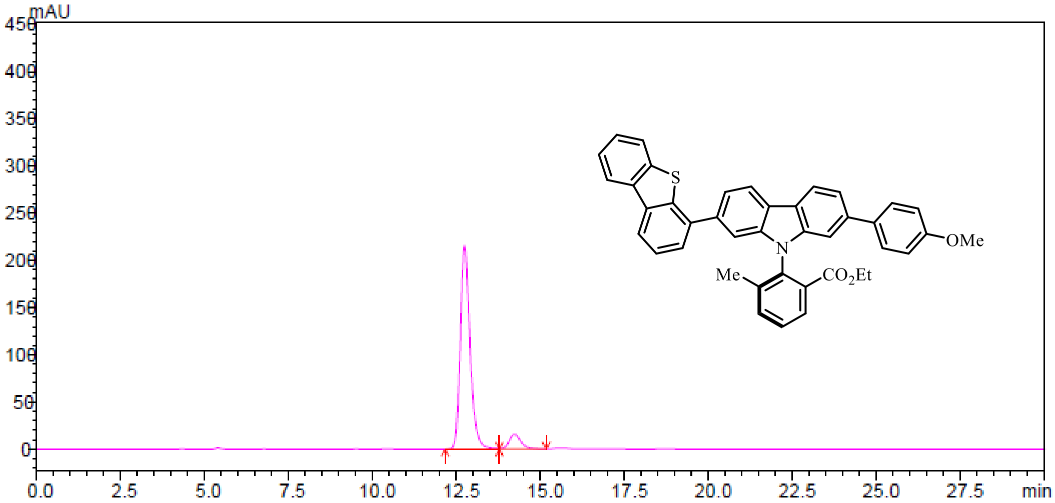

<Peak Table>

PDA Ch1 325nm

Peak#	Ret. Time	Area	Peak Start	Peak End	Area%
1	12.734	4392450	12.160	13.771	92.073
2	14.222	378166	13.771	15.168	7.927
Total		4770615			100.000

SI-**10**

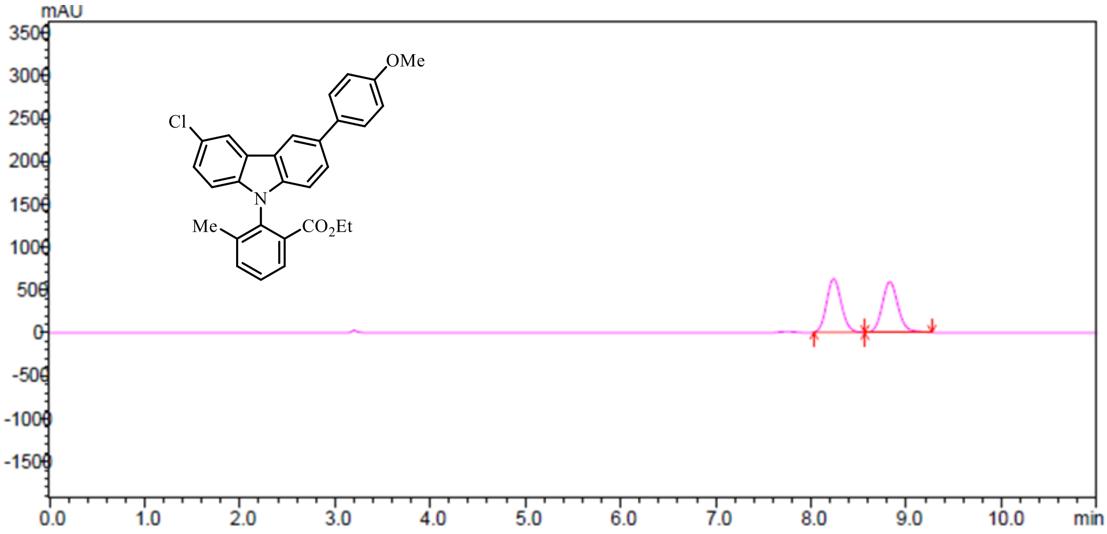

<Peak Table>

PDA Ch1 281nm

Peak#	Ret. Time	Area	Peak Start	Peak End	Area%
1	8.238	6650775	8.032	8.565	49.280
2	8.828	6845045	8.565	9.269	50.720
Total		13495820			100.000

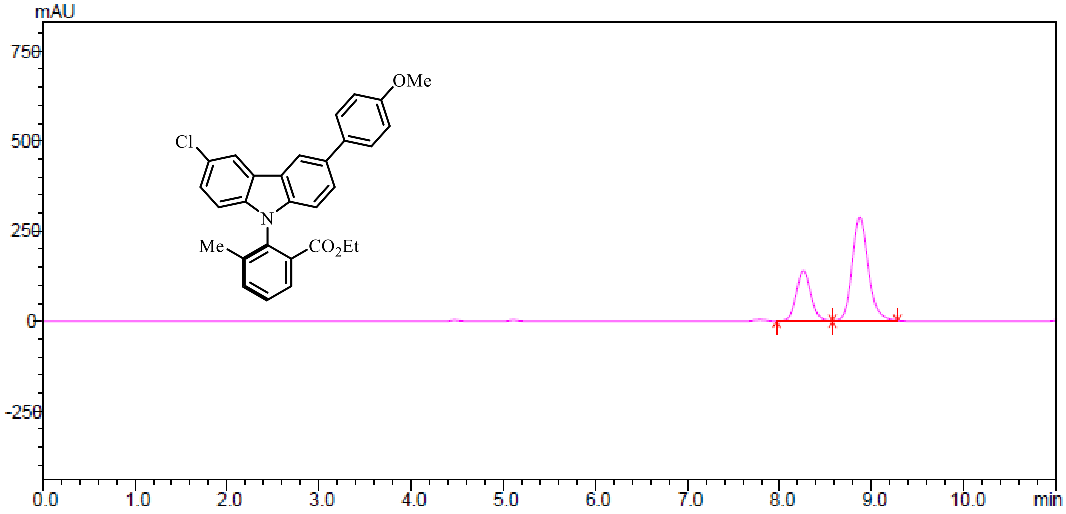

<Peak Table>

PDA Ch1 281nm

Peak#	Ret. Time	Area	Peak Start	Peak End	Area%
1	8.258	1552513	7.968	8.576	30.547
2	8.871	3529892	8.576	9.280	69.453
Total		5082405			100.000

X-Ray Crystallography Data

(S_a, R)-**L6**

The .cif data file is attached as a separate document. CCDC Deposition Number **2390748**

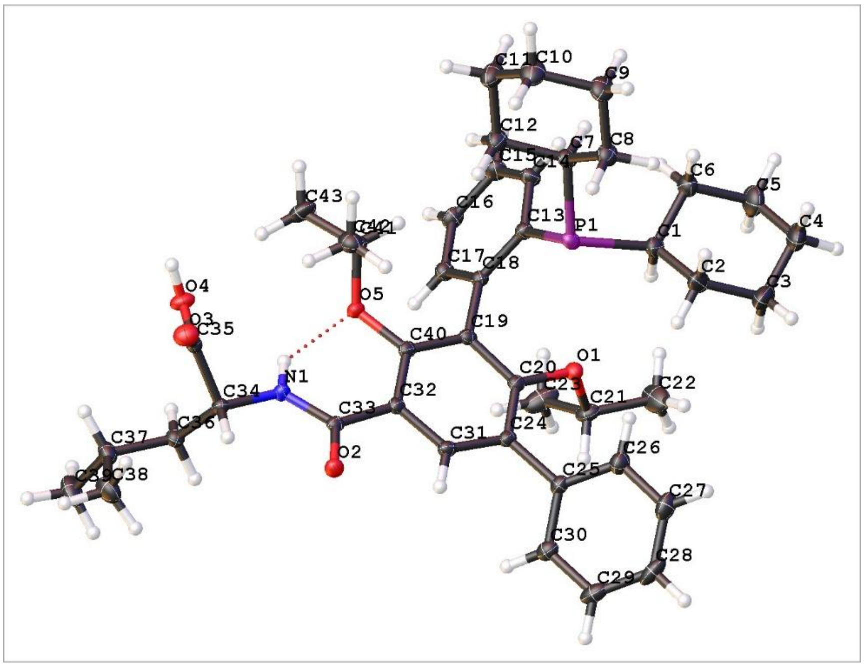

Crystals were obtained by slow evaporation of a solution of the compound in chloroform.

Identification code: o544; Chemical formula: $C_{43}H_{58}NO_5P$; Formula weight: 699.87 g/mol; Temperature: 100 K; Wavelength: 0.71073 Å; Cell: a=9.0732(3) alpha=90, b=13.1703(5) beta=90, c=33.5174(10) gamma=90; Space group P 21 21 21; Volume 4005.2(2); Hall group P 2ac 2ab; Dx, g cm-3: 1.161; Z: 4; Mu (mm-1): 0.112; F000: 1512.0; h,k,lmax:12,18,46; Nref: 11281; Tmin, Tmax: 0.713, 0.743.

2

The .cif data file is attached as a separate document. CCDC Deposition Number **2390746**

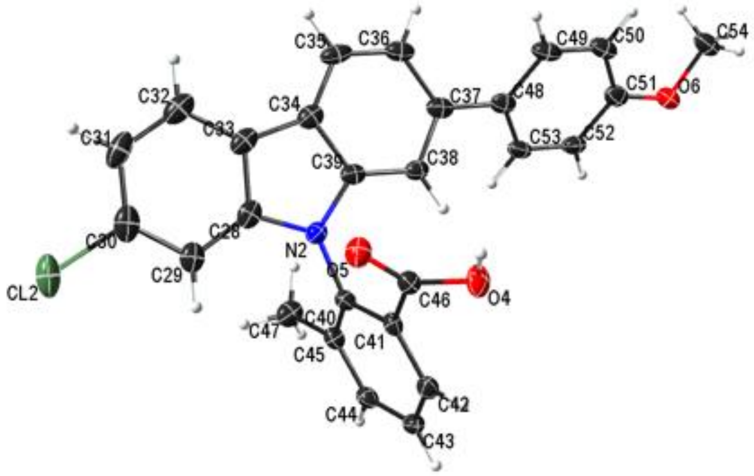

Crystals were obtained by slow evaporation of a solution of the compound in a hexanes–ethyl acetate mixture.

Identification code: N450; Chemical formula: $C_{27}H_{20}ClNO_3$; Formula weight: 441.89 g/mol; Temperature: 100 K; Wavelength: 1.54178 Å; Cell: a=10.5874(5) alpha=90, b=9.3002(4) beta=91.349(1), c=22.1829(10) gamma=90; Volume: 2183.63(17); Space group: P 21; Hall group: P 2yb; Z: 4; Mu (mm-1): 1.789; F000: 920.0; h,k,lmax:13,11,27; Nref: 8519; Tmin, Tmax: 0.632, 0.754.

Et-ester of 44

The .cif data file is attached as a separate document. CCDC Deposition Number **2390747**

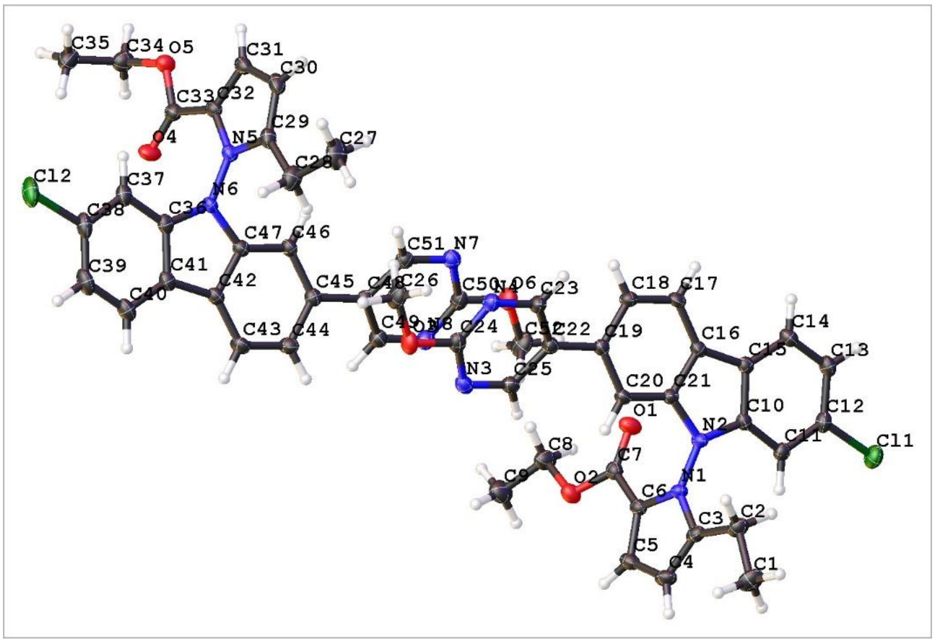

Crystals were obtained by slow evaporation of a solution of the compound in a hexanes–isopropanol mixture.

Identification code: o545; Chemical formula: $C_{26}H_{23}ClN_4O_3$; Formula weight: 474.93 g/mol; Temperature: 100 K; Wavelength: 0.71073 Å; Cell: a=7.3428(6) alpha=102.906(4), b=11.5175(11) beta=97.315(4), c=14.9410(14) gamma=107.868(4); Volume: 1145.82(18); Space group: P 1;Hall group: P 1; Dx, g cm-3: 1.377; Z: 2; Mu (mm-1): 0.204; F000: 496.0; h,k,lmax: 10,16,20; Nref: 12790; Tmin, Tmax: 0.696, 0.746.

References

1. Lou, Y., Wei, J., Li, M., & Zhu, Y. Distal ionic substrate–catalyst interactions enable long-range stereocontrol: access to remote quaternary stereocenters through a desymmetrizing Suzuki–Miyaura reaction. *J. Am. Chem. Soc.* **144**, 123−129 (2022).

2. Wei, J., Gandon, V. & Zhu, Y. Amino acid-derived ionic chiral catalysts enable desymmetrizing cross-coupling to remote acyclic quaternary stereocenters.*J. Am. Chem. Soc.***145**, 16796–16811 (2023).

3. Li, M., Chia, X. L., Tian, C. & Zhu, Y. Mechanically planar chiral rotaxanes through catalytic desymmetrization. *Chem* **8**, 2843–2855 (2022).

4. On, K. W. I., Hong, W. & Zhu, Y. Crossing the ortho-hurdle: ionic stereocontrol enables atroposelective Suzuki-Miyaura coupling. *Chem. Catal.* **3**, 100523 (2023).

5. Kim, B. et al. Distal stereocontrol using guanidinylated peptides as multifunctional ligands: desymmetrization of diarylmethanes via Ullman cross-coupling. *J. Am. Chem. Soc.* **138**, 7939−7945 (2016).

6. Drahomír, V., Ivan, K., Veronika, P. & Vera C. Efficient synthesis of N-alkyl-2,7-dihalocarbazoles by simultaneous carbazole ring closure and N-alkylation. *Tetrahedron* **68**, 5075−5080 (2012).

7. Norifumi, K., Ryohei, K. & Masashi, K. Novel blue light emitting poly(N-arylcarbazol-2,7-ylene) homopolymers: syntheses and properties. *Macromolecules* **39**, 9102−9111 (2006).

8. Trisha, L. A. & Timothy, M. S. Detection of explosives via photolytic cleavage of nitroesters and nitramines. *J. Org. Chem.* **76**, 2976−2993 (2011).

9. Helge, P. et al. 10-(2-oxo-2-Phenylethylidene)-10 *H*-anthracen-9-ones as highly active

antimicrotubule agents: synthesis, antiproliferative activity, and inhibition of tubulin polymerization. *J. Med. Chem.* **54**, 4247−4263 (2011).

10. Yin, S.-Y.; Zhou, Q.; Liu, C.-X.; Gu, Q.; You, S.-L. Enantioselective synthesis of N−N biaryl atropisomers through iridium(I)-catalyzed C−H alkylation with acrylates. *Angew. Chem. Int. Ed.* **62**, e202305067 (2023).

11. Michael, A. C. & Alexander, K. Catalytic, asymmetric, aldol/*o*-conjugate addition sequence for the construction of highly substituted furanoids. *Org. Lett.* **17**, 1385–1388 (2015).

12. Fauchère, J. L., Charton, M., Kier, L. B., Verloop, A. & Pliska, V. Amino acid side chain parameters for correlation studies in biology and pharmacology. *Int. J. Peptide Protein Res.* **32**, 269–278 (1988).
